# A membrane-modulated chemoenzymatic dynamic kinetic resolution for the synthesis of chiral phthalidyl esters

**DOI:** 10.1038/s41467-026-72684-2

**Published:** 2026-05-02

**Authors:** Jun Wu, Donghua He, Yongjin Zhang, Zhendong Feng, Hongxu Liu, Guohua Liu

**Affiliations:** Key Laboratory of Resource Chemistry of the Ministry of Education, Shanghai Engineering Research Center of Green Energy Chemical Engineering, Shanghai Normal University, Shanghai, PR China

**Keywords:** Asymmetric catalysis, Asymmetric synthesis

## Abstract

Chemoenzymatic dynamic kinetic resolution (DKR) offers a powerful bridge between chemocatalysis and biocatalysis for the preparation of chiral molecules. However, its broader application has been limited by the incompatibility between racemization and resolution catalysts, where mutual interference often compromises catalytic activity and/or enantioselectivity. Here, we introduce a membrane-modulated strategy that circumvents the mandatory requirement for strict rate matching, offering a significant conceptual advance in the design of chemoenzymatic DKR systems. By spatially separating racemization and resolution while enabling their collaborative operation within a two-stage, two-step process, this approach dramatically enhances the typically low efficiency of conventional DKR, allowing the efficient synthesis of tetra-substituted 3-hydroxyphthalide esters that are challenging to access by traditional methods, and greatly expanding the scope of chiral phthalide preparation. This membrane-modulated strategy not only streamlines the typically laborious optimization required in conventional DKR for developing an alternative chemoenzymatic DKR approach but also provides a useful platform with the potential for pharmaceutical synthesis.

## Abstract (web summary)

The development of chemoenzymatic dynamic kinetic resolution has long been constrained by principal limitations, which need precise synchronization of racemization and resolution steps for rate matching. Here, the authors introduce a membrane-modulated strategy that circumvents the mandatory requirement for strict rate matching.

## Introduction

**Fig. 1:**
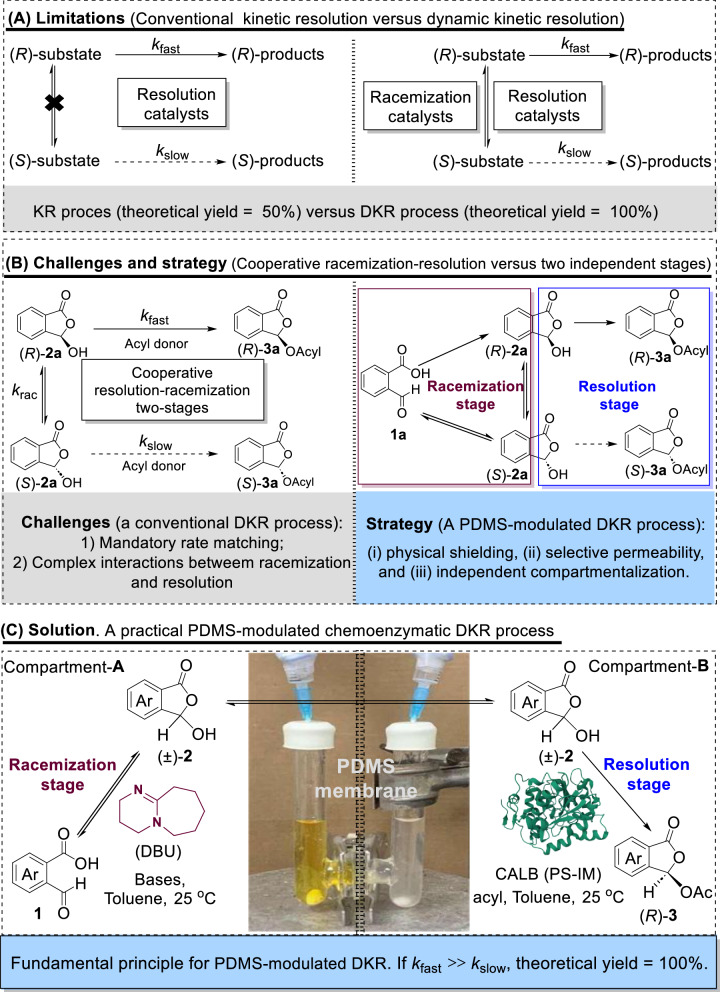
A schematic illustration of limitations, challenges, strategy, and solution for a DKR process. **A** Limitations for the conventional KR versus DKR. **B** Challenges of cooperative racemization–resolution two stages in conventional DKR and a strategy for decoupling racemization and resolution into two independent stages in a PDMS-modulated DKR process. **C** Solution of a practical PDMS-modulated chemoenzymatic DKR process.

Kinetic resolution (KR) is intrinsically limited to a theoretical maximum yield of 50%, whereas dynamic kinetic resolution (DKR) enables complete (theoretical 100%) conversion of racemic substrates into a single enantiomer, as shown in Fig. 1.^1,2^ Over the past decades, the chemoenzymatic DKR has evolved into a powerful platform for the synthesis of a wide range of optically pure pharmaceuticals and fine chemicals, which not only overcomes the intrinsic yield limitation of enzymatic KR but also preserves the enzyme’s exquisite stereoselectivity.^3–8^ However, the fundamental challenge of a conventional chemoenzymatic DKR process lies in achieving compatibility between the two catalytic events of racemization and resolution in accordance with the core principles (Fig. 1A):^9–12^ (i) the KR must display a sufficient enantioselectivity (*E* value = *k*_fast_/k_slow_** ≥** 20), and the rate of racemization (*k*_rac_) must be at least 10 times faster than the enzyme-catalyzed reaction of the slow reacting enantiomer (*k*_slow_) (first limitation), and (ii) the enzyme and the racemization catalyst must be compatible with one another, and the racemization catalyst must not react with the product formed from the resolution (second limitation). The challenge becomes even more pronounced in chemoenzymatic DKR, where additional factors, such as reaction parameters and the nature of the racemization catalyst, can inadvertently cause enzyme deactivation.^13–17^ Therefore, the development of innovative strategies that simultaneously overcome the primary limitation of racemization and resolution rates and address the secondary limitation of chemoenzymatic incompatibility is urgently needed.

Chiral phthalides, a class of bioactive structural motifs widely distributed in natural products, have attracted extensive interest,^18,19^ and recent advances in their asymmetric synthesis have largely centered on purely chemical DKR strategies.^20–25^ These studies primarily focus on elucidating and optimizing the cooperative interactions between racemization and resolution steps in order to enhance both the reactivity and enantioselectivity of DKR processes in accordance with core principles. For example, Smith and co-workers employed an isothiourea catalyst to selectively stabilize the transition state of the (*R*)-isomer via O···S chalcogen bonding, thereby lowering its activation barrier and enabling racemization of the (*S*)-isomer for an efficient DKR process.^20^ In contrast, Zhang’s bicyclic imidazole-based catalyst achieved efficient DKR through conformational control of the transition state via CH–π interactions at exceptionally low temperatures, effectively suppressing non-selective racemization.^22^ Despite these successful examples, achieving an effective DKR process still requires extensive and often laborious optimization to finely control the sensitive racemization step. Therefore, establishing a general strategy that decouples the cooperative racemization-resolution into two independent stages offers a possibility to overcome the limitations of conventional DKR.

Polydimethylsiloxane (PDMS) membranes, as effective physical barriers, have been widely employed in diverse catalytic transformations.^26–28^ The pioneering studies by the Gröger group and others have demonstrated the remarkable utility of PDMS membranes as thimbles in diverse chemoenzymatic two-step sequential enantioselective processes.^29–37^ Although the method is effective, the membrane-mediated DKR process still faces the issue of reduced chiral product conversion due to membrane diffusion limitations. Inspired by the advantage of the continuous-flow DKR approach in achieving quantitative chiral conversion,^38^ and combining the feature of physical separation of enantiomeric products in a membrane-mediated KR process,^39^ a membrane-modulated DKR strategy is expected to decouple racemization and resolution into two independent stages, thereby overcoming the limitations of conventional batch DKR processes. In our design, by decoupling the cooperative racemization–resolution process into two independent stages (blue box versus brown-red box in the strategy of Fig. 1B), the strategy used in a PDMS-modulated process fulfills three collaborative roles to overcome the intrinsic challenges of mandatory rate matching and complex interactions between racemization and resolution of conventional DKR: (i) physical shielding, (ii) selective permeability, and (iii) independent compartmentalization. The shielding function prevents direct contact between racemization chemocatalysts and resolution biocatalysts, thereby eliminating the incompatibility that constitutes the second principal limitation of conventional DKR systems. The selective permeability of the PDMS membrane leverages its dynamic mass-transfer capability, permitting the diffusion of targeted intermediate ((±)−**2a**), while confining catalysts, substrates, and other components to their respective compartments, thereby simplifying the most time-intensive aspect of DKR optimization. The independent compartmentalization establishes two isolated environments and maintains a continuous concentration gradient of intermediates between the two sides of the membrane, thereby driving the cascade forward and overcoming the first principal limitation of DKR systems.

In this work, a practical solution represented by a PDMS-modulated chemoenzymatic DKR process has been established by utilizing the dynamic tautomeric equilibrium between compounds **1** and **2** (Fig. 1C). Compartment-**A** accommodates a base serving as a racemization chemocatalyst, while Compartment-**B** houses a lipase functioning as a resolution biocatalyst. This DKR process ensures complete spatial separation of DBU and CALB while orchestrating the collaborative interplay between DBU-mediated racemization and CALB-mediated resolution. As envisioned, this membrane-modulated DKR strategy furnishes a powerful methodology that grants access to otherwise inaccessible enantioenriched phthalide derivatives, reducing the practical requirement to that of a simple kinetic resolution and thereby achieving high conversion of racemic substrates into a single enantiomer.

## Results and discussion

### Comparative reaction study

To validate the proof-of-concept of the PDMS-modulated strategy, a conventional single-step chemoenzymatic DKR (**I**) was compared with an integrated PDMS-modulated DKR (**II**), in which Compartment-**A** contains a DBU base (DBU: 1,8-diazabicyclo[5.4.0]undec-7-ene)^40–42^ and Compartment-**B** is loaded with CALB lipase (Novozym-435),^43–47^ as summarized in Table 1. To align with the CALB-mediated resolution step, toluene was employed as the reaction medium to evaluate the effect of the racemization step impacted on the catalytic efficiency of the DKR. It was observed that, in the absence of a base, CALB exhibited classical kinetic resolution behavior, affording a 48% yield with 99% *ee* (entry 1).

**Table 1:** Comparative reaction study.^a^


Entry	Type	Catalyst(s)/solvent(s)	%Yield of (*R*)−3a^g^	%*ee*) of (*R*)−3a^g^
1	**I**	CALB/toluene	48	99
2	**I**	CALB/toluene + DBU	68	3
3	**I**	CALB/toluene + pyridine	59	27
4	**I**	CALB/toluene + piperidine	61	13
5	**I**	CALB/toluene + Et_3_N	53	21
6	**I**	CALB/toluene + NaHCO_3_	55	46
7	**I**	CALB/toluene + K_2_CO_3_	58	45
8	**II**	DBU/toluene + CALB/toluene	91	99
9^b^	**II**	NEt_3_/toluene + PS-IM/toluene	88	99
10^c^	**II**	DBU/toluene + CALB/toluene	85	99
11^d^	**II**	DBU/toluene + CALB/toluene	81	99
12^e^	**II**	DBU/toluene + CALB/toluene	83	99
13^f^	**II**	DBU/toluene + CALB/toluene	87	99

^a^Reaction conditions: For reaction (**I),** **1a** (0.20 mmol), base (0.20 mmol), and/or additive in 2.0 mL of solvent (s), 25 °C, 4 h. For reaction (**II**) by using a 0.40 mm-thick PDMS membrane; In Compartment-**A,** **1a** (0.20 mmol), base (0.20 mmol), and/or additive in 2.0 mL of solvent (s), 25 °C, 4 h. In Compartment-**B**, Novozym-435 (CALB) (40.0 mg, 20 mg/0.1 mmol), and acetic anhydride (0.40 mmol, 2.0 equiv.) in 2.0 mL of toluene, 25 °C, 36 h.

^b^Data were obtained using 1.0 equivalents of NEt_3_ instead of DBU in Compartment-**A** and PS-IM (PS-IM: lipases from *Burkholderia* (formerly Pseudomonas) *cepacia*) instead of CALB in Compartment-**B**.

^c^Data were obtained using 1.50 equivalents of DBU in Compartment-**A**.

^d^Data were obtained using 0.50 equivalents of DBU in Compartment-**A**.

^e^Data were obtained using a 0.20 mm thick PDMS membrane.

^f^Data were obtained using a 0.60 mm thick PDMS membrane.

^g^Yields were determined by ^1^H–NMR analysis in Compartment-**B**, and the %*ee* values were determined by chiral HPLC analysis.

Subsequently, a series of different bases were evaluated as additives to probe their influence on the conventional single-step chemoenzymatic DKR (**I**) process (see Supplementary Table 1). Owing to the pronounced dynamic tautomeric equilibrium between 2-formylbenzoic acid (**1a**) and 3-hydroxyisobenzofuran-1(3H)-one (**2a**), with **2a** predominating through a ring-opening pathway under basic conditions, two clear trends were observed. On the one hand, all the tested model reactions with the addition of either organic or inorganic bases afforded yields exceeding 50%. On the other hand, all types of bases significantly reduced the enantioselectivity (entries 2–7). The former observation suggested that the presence of bases induced the DKR behavior, with DBU identified as the optimal racemization catalyst owing to its relatively high yield (entry 2 versus entries 2–7). In contrast, the latter finding indicated that bases might induce folding or structural perturbation of CALB, thereby disrupting its favorable chiral microenvironment and leading to markedly diminished *ee* values.^48–51^ Notably, these comparisons revealed that the conventional two-step cascade DKR process was severely suppressed in the presence of bases due to a deactivation effect of bases impacting CALB, thereby disclosing the incompatible nature of the conventional DKR process that originated from the mismatch between base-mediated racemization and CALB-mediated resolution.

Finally, in the evaluation of an integrated PDMS-modulated DKR (**II**) based on the optimizations (see Supplementary Table 2), it was observed that the model reaction could steadily provide the final (*R*)-**3a** with a 91% yield and 99% *ee* (entry 8), in which the permeation of less than 4.8% DBU from Compartment-**A** to Compartment-**B** and 0.2% acetic anhydride from Compartment-**B** to Compartment-**A** had a negligible impact on the enantioselectivity of the product, as confirmed by GC analysis (see Supplementary Fig. 1). Such a result outperformed the reported outcomes when using triethylamine as the racemization catalyst and PS-IM as the biocatalyst (entry 8 versus entry 9).^25^ Furthermore, this result was also superior to the outcomes obtained with other DBU loadings (entry 8 versus entries 10 and 11). In addition, it was also found that the membrane thickness had a certain impact on the PDMS-modulated DKR process, in which the reaction using a 0.40 mm-thick membrane yielded the best results compared to the reactions with other membrane thicknesses (entry 8 versus entries 12 and 13). These findings demonstrate that the designed membrane-modulated DKR strategy could achieve the anticipated coordination of DBU-mediated racemization with CALB-catalyzed resolution in a two-stage, two-step process, thereby enabling an efficient chemoenzymatic DKR to proceed that was previously incompatible under the conventional one-stage two-step chemoenzymatic conditions.

### Mechanistic studies

**Fig. 2:**
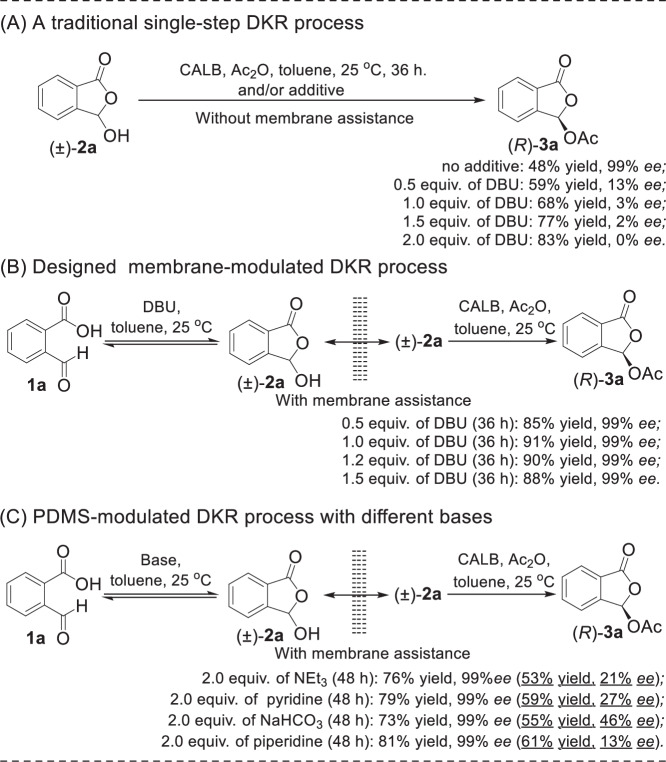
Contrastive experiments. **A** A traditional single-step DKR process. **B** The designed PDMS-modulated DKR process. **C** The PDMS-modulated DKR process with different bases in Compartment-**A**.

To gain deeper insights into the PDMS-modulated chemoenzymatic DKR process, a series of control experiments was systematically conducted to further evaluate the two-stage sequence of DBU-mediated racemization and CALB-catalyzed resolution, as outlined in Fig. 2. In the first type of evaluation, the traditional single-step reaction performed without membrane assistance revealed that, despite extensive optimization, increasing the molar amount of DBU consistently improved the reaction yield but invariably led to a pronounced erosion of enantioselectivity (Fig. 2A). This observation indicates that: (1) DBU influences the catalytic activity and potentially the enantioselectivity of CALB.^48–50^ (2) DBU itself can function as a non-selective catalyst for the esterification reaction.^40^ Supporting evidence was provided by a control experiment, in which omission of CALB and addition of two equivalents of DBU relative to the substrate furnished **3a** in 46% yield but without enantioselectivity. Furthermore, compared to the 91% yield and 99% *ee* achieved by the designed PDMS-modulated DKR process (Entry 8, Table 1), these experimental data conversely confirmed that the efficient DKR could only be attained through spatial compartmentalization of the two catalysts, thereby demonstrating the advantage of the PDMS-modulated strategy. In addition, such an advantage also eliminates the mutual interference and cross-reactivity of DBU with CALB, thereby simplifying the otherwise laborious optimizations required in conventional DKR. This judgment could be further validated by the designed PDMS-modulated control experiments (Fig. 2B). It was observed that, irrespective of the amount of DBU introduced into Compartment-**A**, the reaction consistently proceeded with high yield and an excellent enantioselectivity of 99% *ee*.

To elucidate the advantage of independent compartmentalization in a PDMS-modulated DKR process, a series of bases with intrinsically low racemization activities was subsequently evaluated, as shown in Fig. 2C. It was observed that all four tested bases, under the conditions of increased molar amounts of bases and extended reaction times, when combined with the membrane-modulated strategy, enabled an efficient DKR process. This approach yielded chiral products with significantly higher yields and enantioselectivity compared to traditional one-pot conditions without membrane assistance (data in brackets for entries 3–6 in Table 1). These observations indicated that the continuous consumption of one enantiomer by CALB promoted the persistent racemization of the remaining enantiomer. Remarkably, during this process, CALB-mediated esterification in Compartment-**B** generated a concentration gradient that persistently drove the DKR process forward. As equilibrium was reached, the unreacted enantiomer diffused back into Compartment-**A** for racemization, thereby establishing a self-sustaining cycle of substrate turnover. Thus, the combination of this membrane-mediated device with the dynamic tautomerization facilitated by any racemization base catalyst in Compartment-**A** was sufficient to sustain an efficient DKR process, thereby developing an approach in which a concentration gradient, rather than relative rate control, drives the process.

**Fig. 3:**
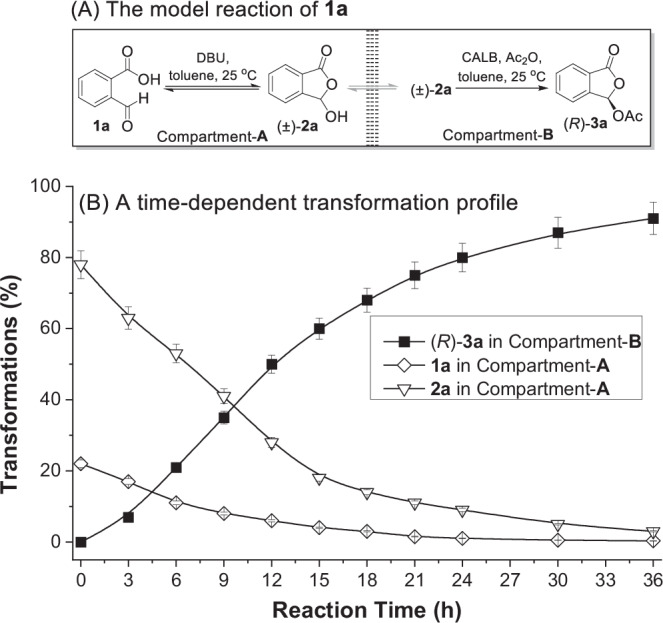
A kinetic investigation of this chemoenzymatic DKR process. **A** The model reaction of **1a**. **B** A time-dependent transformation profile (The error bars represent the standard deviation).

To further explore the nature of the racemization/resolution route, a kinetic investigation of this chemoenzymatic DKR process was conducted, as shown in Fig. 3, which utilized the model reaction of **1a** (Fig. 3A) along with its time-dependent transformation profiles (Fig. 3B). Initially, within the first 12 h, the concentration of **2a** decreased sharply, accompanied by a simultaneous decline in **1a**. During this stage, the mole fraction of **2a** dropped from 78% to 28%, indicating that **2a** predominantly diffused through the membrane into Compartment-**B**. Meanwhile, the CALB-mediated esterification in Compartment-**B** accelerated, delivering (*R*)−**3a** in 50% yield at 12 h. Over the next 6 h (12–18 h), the reaction rate gradually slowed, yet the yield of (*R*)−**3a** continued to rise, reaching 68% at 18 h, accompanied by a gradual decline of **1a** in Compartment-**A**, indicative of the establishment of a dynamic equilibrium of **2a** across the membrane-separated compartments during this stage. Finally, during the subsequent 28 h (18–36 h), the CALB-mediated esterification in Compartment-**B** proceeded more steadily and gradually, implying that the back-diffusion of the unreacted (*S*)-configured **2a** sustained the dynamic equilibrium necessary for the reaction to progress, ultimately affording (*R*)−**3a** in 91% yield.

**Fig. 4:**
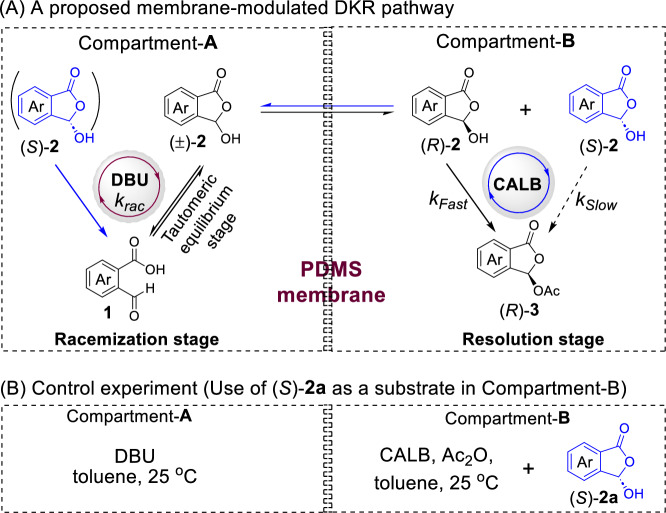
Proposed reaction pathway and control experiment. **A** A proposed membrane-modulated DKR reaction pathway. **B** Control experiment through the use of (*S*)−**2a** as a substrate in Compartment-**B**.

Based on the contrastive experiments and time-course investigations, the proposed membrane-modulated DKR reaction pathway is confirmed by a control experiment, as shown in Fig. 4. The reaction pathway can be delineated as a sequence of three stages: (i) an initial reversible ring-opening/closing equilibrium between **1** and **2** in Compartment-**A** (dynamic tautomeric equilibrium stage), (ii) CALB-mediated selective esterification of (*R*)−**2** in Compartment-**B** with a favorable *k*_fast_ » *k*_slow_ rate relationship (resolution stage), and (iii) DBU-mediated racemization in Compartment-**A** (racemization stage), as illustrated in Fig. 4A. In the initial dynamic tautomeric equilibrium stage, substrates **1** and **2** are maintained in a reversible equilibrium in the presence of DUB conditions within Compartment-**A**. This equilibrium facilitates the transmembrane migration of racemic **2** while retaining **1** in Compartment-**A**, thereby sustaining the balance required to initiate the reaction, as confirmed by the ^1^H NMR analysis.

In the second resolution stage, **2** diffuses through the PDMS membrane into Compartment-**B**, with a diffusion rate of 6.35 × 10⁻^12^ m^2^/s determined via the controlled experiments (see Supplementary Fig. 2).^52,53^ Concurrently, the CALB-mediated selective esterification of (*R*)−**2** occurs in Compartment-**B**, yielding the target product (*R*)-**3**, while the unreacted intermediate (*S*)−**2** (blue-labeled) accumulates. As the reaction proceeds, the continuous consumption of (*R*)−**2** and accumulation of unreacted (*S*)-**2** elevates the local concentration of (*S*)-**2a** in Compartment-**B**. When this concentration surpasses that of racemic **2** in Compartment-**A**, the imbalance generates a concentration gradient that drives the back-diffusion of (*S*)-**2** (blue-labeled) into Compartment-**A**, thereby initiating the third racemization stage. In this process, the DBU-mediated racemization proceeds through a reversible ring-opening/closing mechanism, regenerating racemic **2** for subsequent CALB resolution in Compartment-**B**. Controlled comparative kinetic tests of racemization in Compartment-**A** revealed that the DBU significantly influenced the racemization process. The corresponding rate constant and half-life for the DBU-mediated racemization were determined to be 0.707 h^-1^ and 0.980 h, respectively, calculated using a reported method (see Supplementary Fig. 3).^54–56^ Direct evidence for this reaction pathway was obtained from the control experiment in the membrane-modulated DKR process (Fig. 4B). It was observed that when CALB and (*S*)-**2a** (97% ee) were confined to Compartment-**B** while Compartment-**A** was left substrate-free under standard conditions, (*R*)−**3a** was still obtained in 89% yield with 99% *ee*. This result confirms that (*S*)−**2a** (blue-labeled) back-diffuses into Compartment-**A** for the racemization to racemic **2a**, which subsequently re-enters Compartment-**B** for further CALB-mediated resolution. Moreover, the additional experimental evidence, the significantly higher yields ( > 50%), together with the consistently high *ee* values observed in these experiments (Fig. 2C), also demonstrated that the residual (*S*)−**2a** in Compartment-**B** must undergo reverse transport into Compartment-**A** for DBU-mediated racemization, followed by re-entry into Compartment-**B** for CALB-mediated esterification. Finally, this self-sustaining cycle ultimately enables nearly complete consumption of racemic **2**, achieving an efficient DKR process. Notably, this mechanism effectively bypasses the long-standing challenges of mandatory rate-matching, incompatibility, and laborious racemization optimization. Instead, it establishes a collaborative DKR pathway driven by concentration gradients across compartments, thereby enabling efficient realization of DKR processes that are otherwise incompatible under conventional conditions.

### Substrate scope and utility

**Fig. 5:**
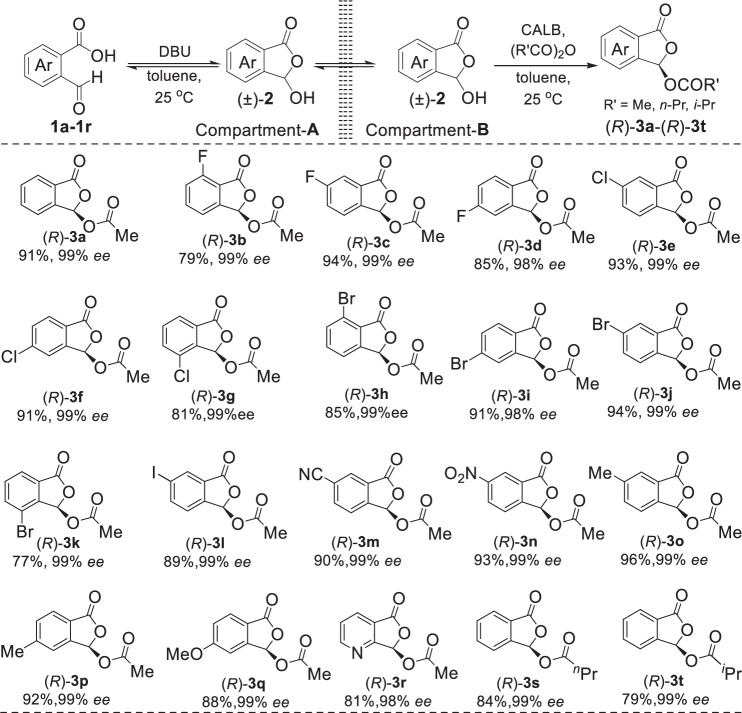
Substrate scope investigation. In compartment-**A,** **1** (0.20 mmol), DBU (0.20 mmol) was introduced into 2.0 mL of toluene. In compartment-**B**, the Novozym-435 (CALB) (40.0 mg, 20 mg/0.1 mmol) and anhydride (0.40 mmol, 2.0 equiv.) were introduced into 2.0 mL of a mixture of toluene. Both reactions in each compartment were stirred at 25 °C for 24–60 h unless otherwise stated. Isolated yields were based on substrate **1**. The *ee* values were determined by HPLC analysis. The absolute configuration of **3a** was determined based on X-ray analysis (Supplementary Tables 3–9), and those of **3b−3t** were tentatively assigned by analogy. Me methyl, ^*n*^Pr *n*-propyl, ^*i*^Pr *iso*-propyl.

After successfully establishing an efficient chemoenzymatic DKR process, a broad range of substrates was examined to elucidate the membrane-modulated strategy, as illustrated in Fig. 5 (see Supplementary HPLC analyzes and characterizations of chiral products). In the series of the tri-substituted 3-hydroxyphthalides, the membrane-modulated strategy exhibited broad generality, enabling efficient conversion of all tested substrates into the corresponding chiral phthalide esters with high yields and excellent enantioselectivity that were difficult to achieve by conventional methods due to their inherent incompatibility. Interestingly, neither the electronic properties nor the steric properties of the substituents on the aromatic ring markedly affected the enantioselectivity owing to the stereoselective nature of CALB, in which both electron-withdrawing and electron-donating substituents afforded products with excellent *ee* values ((*R*)-**3b**–(*R*)-**3n** versus (*R*)-**3o**–(*R*)-**3q**). However, the steric hindrance exerted a pronounced effect on the reaction efficiency. In comparison to substrates with the substituents on the aromatic ring at the 5- or 6-positions, those bearing substituents at the 4- or 7-positions gave considerably lower yields ((*R*)-**3b**, (*R*)-**3g**, (*R*)-**3h**, (*R*)-**3k**). Furthermore, reactions with a heterocycle-substituted substrate also afforded good results ((*R*)-**3r**). Also, the strategy proved applicable to different acylation reagents, delivering the corresponding chiral products in high efficiency ((*R*)-**3s**–(*R*)-**3t**). In addition, the absolute configuration of the chiral product was established by single-crystal X-ray diffraction analysis, which revealed the (*R*)-configuration,^25^ as confirmed by a representative crystal of (*R*)-**3a** obtained from a 1:10 (v/v) mixture of dichloromethane and *n*-pentane (CCDC−2486834, also see Supplementary Tables 3–9).

**Fig. 6:**
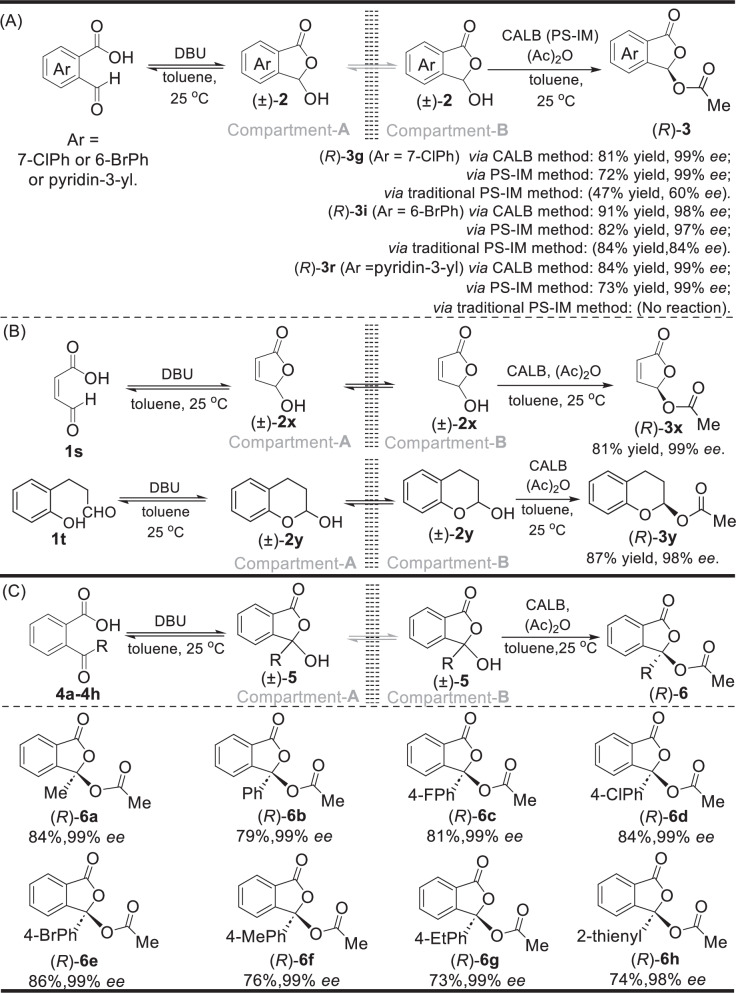
Expanded substrate scope. **A** Comparison of the membrane-modulated CALB method, the membrane-modulated PS-IM method, and the traditional PS-IM method. **B** Expanded other types of substrates. **C** Expanded *tetra*-substituted 3-hydroxyphthalides.

Beyond its general feasibility, the membrane-modulated strategy also demonstrated pronounced advantages over previously reported catalytic systems, as shown in Fig. 6. First, for substrates previously identified as low-reactivity, such as phthalidyl esters bearing chlorine substitution at the 7-position or bromine substitution at the 5-position of the phenyl ring,^25^ the membrane-modulated DKR process consistently delivered the enhanced yields and markedly improved enantioselectivities, irrespective of whether the reported PS-IM enzyme (PS-IM method) or CALB enzyme (CALB method) was employed (Fig. 6A).

This finding suggested that the membrane-modulated strategy could compensate for the substrate limitations in the conventional method, which led to decreased catalytic efficiency due to incompatibility. For heteroaryl substrate **2r**, which could not be transformed by conventional DKR methods, the membrane-modulated process proceeded smoothly with both CALB and PS-IM, affording the desired products in high yield and excellent enantioselectivity (Fig. 6A). Second, it was also found that the PDMS‑modulated DKR process was suitable for other types of substrates, in which a five-membered dihydrofuran derivative (*R*)-**3x** and a six-membered dihydropyran derivative (*R*)-**3y** could be stably obtained in high yield and with excellent enantioselectivity (Fig. 6B).^57^ This finding demonstrates the ability to mitigate activity loss caused by interference from conventional external reaction conditions. Third, for the racemic tetra-substituted 3-hydroxyphthalides (**5a–5h**), which was difficult to access by conventional chemoenzymatic strategy, the membrane-modulated DKR also exhibited remarkable tolerance, consistently furnishing the challenging chiral tetra-substituted 3-hydroxyphthalide esters ((*R*)-**6a**–(*R*)-**6h**) in acceptable yields with excellent enantioselectivities (Fig. 6C) (see Supplementary HPLC analyzes and characterizations of chiral products). Moreover, the electronic and steric effects of alkyl or aryl substituents were found to be negligible on enantioselectivity, as reactions with both electron-withdrawing and electron-donating groups uniformly afforded excellent *ee* values. Therefore, these observations demonstrated the distinct advantages of the membrane-modulated strategy, in which it not only overcame the long-standing challenges of compatibility and rate-matching inherent to conventional DKR processes but also enabled an efficient transformation of previously inaccessible substrate classes, thereby substantially broadening the synthetic scope of chiral phthalidyl esters.

**Fig. 7:**
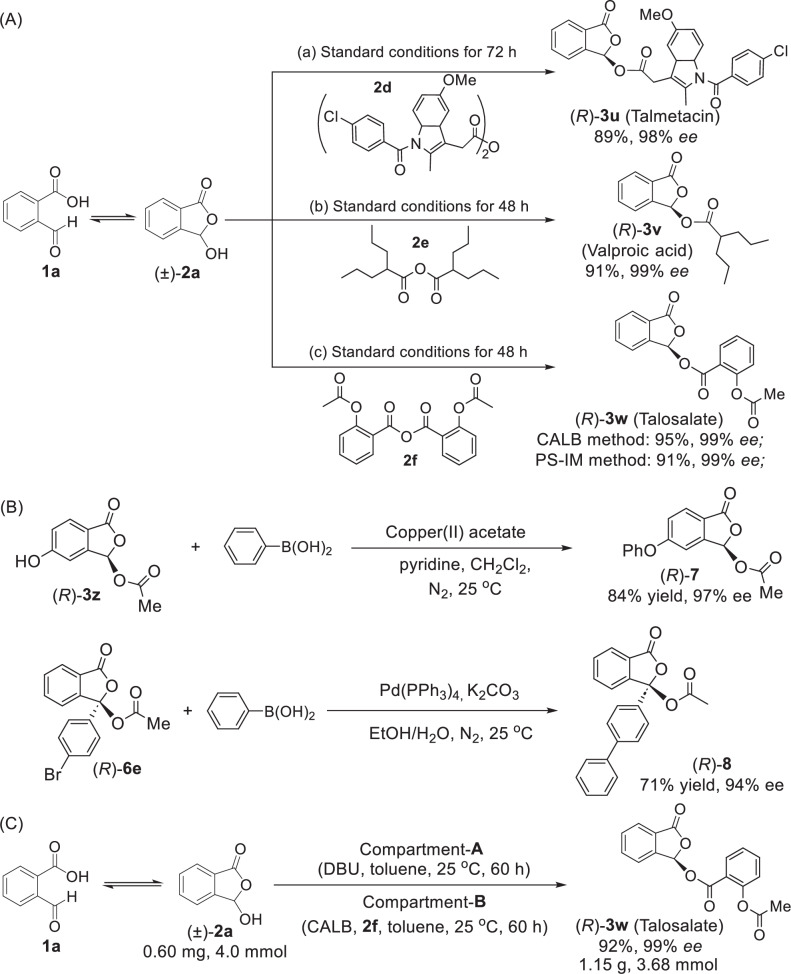
Practical utility of the membrane-modulated DKR process. **A** Preparations of Talmetacin, Valproic acid, and Talosalate. **B** Derivatization of chiral products. **C** Gram-scale synthesis of Talosalate.

To further explore the practical utility of the membrane-modulated DKR process, chiral phthalide prodrugs of important bioactive molecules were prepared (see Supplementary HPLC analyzes and characterizations of chiral phthalide prodrugs), as shown in Fig. 7.

It was found that three prodrugs, talmetacin (89% yield), valproic acid (an antiepileptic drug; 91% yield), and talosalate (95% yield), were obtained with excellent optical purities (Fig. 7A), in which the 99% *ee* values could be difficult to access using conventional chemical approaches.^21–23^ In addition to the preparation of chiral phthalide prodrugs, the obtained chiral products could also be further derivatized to synthesize their analogues (Fig. 7B). It was found that (*R*)-**3z** could afford product (*R*)-**7** through an etherification reaction, while (*R*)-**6e** yielded the corresponding coupling product (*R*)-**8** through a coupling reaction (see Supplementary HPLC analyzes and characterizations of chiral phthalide prodrugs).^58–60^

Furthermore, to validate the generality of the membrane-modulated DKR strategy, the use of PS-IM as a resolution catalyst also afforded talosalate in 91% yield with 99% ee (the CALB method via the PS-IM method in Fig. 7B). More importantly, this strategy not only underscored its potential as a broadly applicable platform but also demonstrated high practicality for the industrial production of enantioenriched pharmaceuticals through a straightforward gram-scale synthesis. It was observed that the DKR of **2a** (4.0 mmol) delivered 1.15 g of the optically pure talosalate (*R*)-**3k** in 92% yield with 99% ee upon extended reaction time (Fig. 7C). Furthermore, it was also found that the PDMS membrane could be reused five times while still maintaining 81% yield and 97% ee, as confirmed by the SEM images (see Supplementary Table 10 and Fig. 4).

In conclusion, we have established a PDMS membrane–modulated strategy that overcomes the intrinsic constraints of conventional chemoenzymatic DKR. The PDMS membrane unites three collaborative functions, physical shielding, selective permeability, and independent compartmentalization, which together eliminate the long-standing challenges of catalyst incompatibility and kinetic matching in conventional DKR. Physical shielding prevents direct contact between racemization and resolution catalysts, eliminating mutual deactivation. Whereas selective permeability enables dynamic mass transfer of cyclic substrates while confining catalysts to their respective domains, thus bypassing the laborious rate-ratio optimization. The independent compartmentalization creates mutually isolated environments and maintains a concentration gradient of intermediates across the membrane, thereby enabling the DKR to proceed continuously and effectively. Mechanistic studies reveal a collaborative two-stage pathway wherein chemocatalytic racemization is seamlessly coupled to enzymatic resolution. This work introduces a generalizable PDMS-modulated platform for integrating incompatible chemo- and biocatalytic processes into a single operational step, providing both a conceptual and practical advance over conventional chemoenzymatic DKR approaches.

## Methods

### The general procedure for the PDMS-modulated DKR

In compartment-**A,** **1** (0.20 mmol), DBU (0.20 mmol) was introduced into 2.0 mL of toluene. In compartment-**B**, the Novozym-435 (CALB) (40.0 mg, 20 mg/0.1 mmol) and anhydride (0.40 mmol, 2.0 equiv.) were introduced into 2.0 mL of a mixture of toluene. A balloon filled with air (*P* = 1 atm) was connected to the top of both compartments. Both reactions in each compartment were stirred at 25 °C for 24–60 h. After this, the resulting solution in Compartment-**B** was filtered. The resulting toluene from the combined filtrates of Compartment-**B** and the solution in Compartment-**A** was removed using a rotary evaporator. The remaining solution was extracted with EtOAc (3 × 5 mL), and the collected organic phase was rinsed with brine (10 mL). After drying the organic layer over anhydrous Na_2_SO_4_, filtration of the drying agent, and evaporation of the solvent under vacuum, the crude product was purified by silica gel flash column chromatography, affording the chiral products **3**. The enantiomeric excess (*ee*) values were determined using HPLC analysis with a Photo-Diode Array detector and a Daicel chiral cell column (Φ 0.46 × 25 cm).

## Supplementary information

Supplementary information

Transparent Peer Review file

## Data Availability

The authors declare that the data supporting the findings of this study are available within the article and Supplementary Information file, or from the corresponding author upon request. The X-ray crystallographic coordinates for structures reported in this study have been deposited at the Cambridge Crystallographic Data Centre (CCDC), under deposition numbers of CCDC 2486834 [(*R*)-**3a**: (*R*)-3-oxo-1,3-dihydroisobenzofuran-1-yl acetate in Supplementary Tables 3–9]. These data can be obtained free of charge from The Cambridge Crystallographic Data Centre via https://www.ccdc.cam.ac. uk/structures/.
