# Supplementary material for: A membrane-modulated chemoenzymatic dynamic kinetic resolution for the synthesis of chiral phthalidyl esters
Source: Nat Commun. 2026 May 2;17:5950. doi: 10.1038/s41467-026-72684-2 (PMC13342490; doi:10.1038/s41467-026-72684-2)
Supplement: Supplementary file 1 — Supplementary information [file 41467_2026_72684_MOESM1_ESM.pdf]

Supplementary Information

A Membrane-Modulated Chemoenzymatic Dynamic Kinetic Resolution for the Synthesis of Chiral Phthalidyl Esters

Jun Wu[1], Donghua He[1], Yongjin Zhang[1], Zhendong Feng[1], Hongxu Liu[1], and Guohua Liu[1]*

[1]Key Laboratory of Resource Chemistry of the Ministry of Education, Shanghai Engineering Research Center of Green Energy Chemical Engineering, Shanghai Normal University, No.100 Guilin Rd, Shanghai, 200234, PR China. *E-mail: ghliu@shnu.edu.cn

CONTENTS

Supplementary Experimental. S2
Supplementary Table 1. Optimizing reaction conditions for the traditional DKR of **2a** S19
Supplementary Table 2. Optimizing reaction conditions for the PDMS-modulated DKR of **2a** ... S20
Supplementary Table 3. Methods and crystal data, and structure refinement for (*R*)-**3a** S21
Supplementary Table 4. Fractional atomic coordinates ($\times10^4$) and equivalent isotropic displacement parameters ($Å^2\times10^3$) for (*R*)-**3a** S23
Supplementary Table 5. Anisotropic displacement parameters ($Å^2\times10^3$) for (*R*)-**3a** S24
Supplementary Table 6. Bond lengths for (*R*)-**3a** S25
Supplementary Table 7. Bond angles for (*R*)-**3a** S25
Supplementary Table 8. Torsion angles for (*R*)-**3a** S26
Supplementary Table 9. Hydrogen atom coordinates ($Å\times10^4$) and isotropic displacement parameters ($Å^2\times10^3$) for (*R*)-**3a** S27
Supplementary Figure 1. The permeation analyses of DBU and acetic anhydride S28
Supplementary Figure 2. Measurement of the diffusion coefficient through PDMS S30
Supplementary Figure 3. Measurement of the DBU-racemization kinetic parameters S32
HPLC analyses of chiral products ((*R*)-**3a**-(*R*)-**3t**) S36
Characterizations of chiral products ((*R*)-**3a**-(*R*)-**3t**) S56
HPLC analyses of chiral products ((*R*)-**6a**-(*R*)-**6h**) S79
Characterizations of chiral products ((*R*)-**6a**-(*R*)-**6h**) S87
HPLC analyses of chiral phthalide prodrugs ((*R*)-**3u**-(*R*)-**3w**) S96
Characterizations of of chiral phthalide prodrugs ((*R*)-**3u**-(*R*)-**3w**) S104
Supplementary Table 10. Reusability of the PDMS membrane S112
Supplementary Figure 4. The SEM image of the PDMS membrane before and after cycle S113
Supplementary References S119

Supplementary Experimental

1. General.

All reactions involving air- or moisture-sensitive reagents or intermediates were carried out in oven-dried glassware using standard Schlenk techniques. Ketones are commercially available and were used without additional purification and/or pretreatment. Deuterated solvents were purchased commercially and were degassed and stored over activated 4 Å molecular sieves. Chromatography grade *n*-hexane and 2-propanol (2-PrOH) used in high-performance liquid chromatography (HPLC) were purchased from Sigma−Aldrich Company Ltd and used as received. All other commercially available reagents were purchased from Sigma-Aldrich, TCI Chemicals, Alfa Aesar, Acros Organics, or ABCR in the highest purity grade and used without further purification. The unit of the optimal enzymatic activity of lipase CALB coming from Novozym-435 is defined as the amount of enzyme that can release 1 μmol of butyric acid per minute at pH 8.0 and 40 °C (Tributyrin caprate is the substrate). The unit of the optimal enzymatic activity of lipase PS-IM is defined as the amount of enzyme that can reduce 1 μmol of butyric acid per minute at pH 7.5 and 30 °C (Tributyrin caprate is the substrate). The membrane was purchased from Zhongke Materials Company (The membrane has a polymerization degree of 10:1, dimensions of 15×15 cm, thicknesses of 200 μm/400 μm/600 μm/1 mm/2 mm/3 mm, an operating temperature range of -40 to 200 °C, and is non-toxic to biological systems)

2. Characterization.

The ^{1}H NMR, ^{13}C NMR, and/or ^{19}F NMR spectra were performed on a Bruker Avance DPX-400 spectrometer in $CDCl_3$ solutions. Chemical shifts are given in parts per million (δ units) downfield from tetramethylsilane using the residual solvent signal ($CHCl_3$, δ 7.26) as an internal standard. ^{1}H NMR information is given in the following format: multiplicity (s, singlet; d, doublet; t, triplet; q, quartet; qui, quintet; sept, septet; m, multiplet), coupling constant(s) (*J*) in Hertz (Hz), and the number of protons. The prefix app is occasionally applied when the true signal multiplicity is unresolved, and br indicates the signal in question is broadened. High-resolution mass spectrometry (HRMS) spectra were obtained on a micro TOF-QII Instrument. HPLC analyses were carried out on a Hewlett-Packard Model HP 1200 instrument. The enantiomeric excesses (*ee*) were determined using a Daicel Chiralcel® column AD-H or OD-H, or OZ-H with the above HPLC setup.

3. The synthesis of substrates.

3.1. General procedure for preparation of aryl-substituted 3-hydroxyisobenzofuranones.[1]

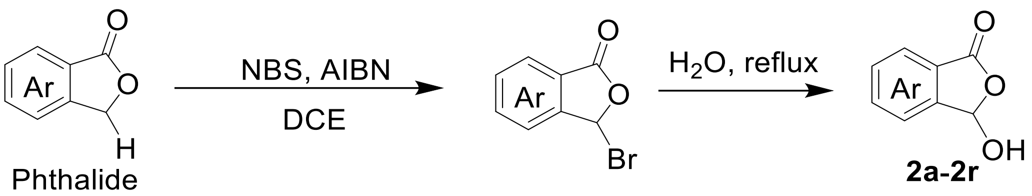

A typical procedure was as follows. Phthalide (0.20 mol), NBS (2.4-3 equiv.), and benzoyl peroxide (15.0 mg) were suspended in 10 mL of DCE. The mixture was heated to reflux with stirring under an argon atmosphere for 2-10 h, cooled to room temperature, and filtered. The residue was washed with DCE (2 × 5 mL), and the filtrate was concentrated in vacuo. After 5.0 mL of water was added, the resulting mixture was heated to reflux with stirring for 4-12 h. After cooling to room temperature, the mixture was acidified with $NaHSO_4$ and extracted with EtOAc (3 × 5 mL). The combined organic layers and the collected organic phase were rinsed with brine (5 mL). After drying the organic layer over anhydrous Na_2SO_4, filtration of the drying agent, and evaporation of the solvent under vacuum, the crude product was purified by silica gel flash column chromatography, affording the products **2a-2r** in 71-85% isolated yields.

3.2. General procedure for the Synthesis of keto acids *via* a Friedel-Crafts reaction.[2]

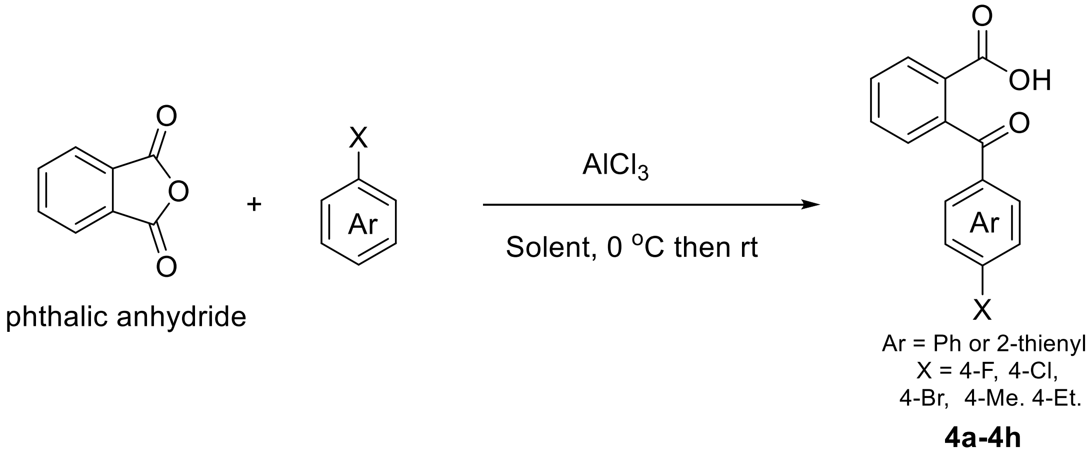

A typical procedure was as follows. To a flame-dried round-bottom flask, phthalic anhydride (1 equiv.) was dissolved in 10 mL of anhydrous THF. Aluminum chloride powder (2.5 equiv.) was then added at 0 °C, followed by the addition of the corresponding arenes (1.5 equiv.). The reaction mixture was stirred at room temperature for 12 h. Upon complete consumption of the starting material, the reaction mixture was poured into crushed ice water. Concentrated hydrochloric acid was added until the pH reached a range of 1-3. The organic layer was extracted with EtOAc (3 × 5 mL). The combined organic layers and the collected organic phase were rinsed with brine (5 mL). After drying the organic layer over anhydrous Na_2SO_4, filtration of the drying agent, and evaporation of the solvent under vacuum, the crude product was purified by silica gel flash column chromatography, affording the products **4a-4h** in 81-89% isolated yields.

3.3. General procedure for synthesis of anhydride.[3]

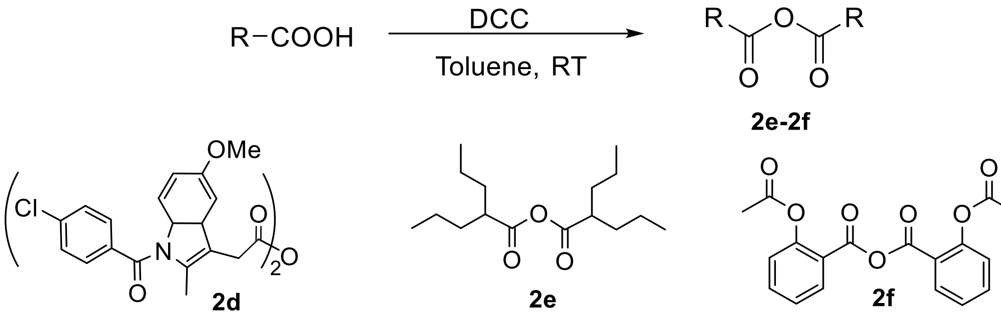

A typical procedure was as follows. To a flame-dried round-bottom flask, dicyclohexyl carbodiimide (DCC) (0.5 equiv.) was dissolved in 10 mL of anhydrous toluene. The corresponding carboxylic acid (1.0 equiv.) was then added at 25 °C. The reaction mixture was stirred at room temperature for 12 h. Upon complete consumption of the starting material, the reaction mixture was poured into crushed ice water. The sodium hydroxide solution (1.0 M) was added until the pH reached a range of 7. The organic layer was extracted with EtOAc (3 × 5 mL). The combined organic layers and the collected organic phase were rinsed with brine (5 mL). After drying the organic layer over anhydrous Na_2SO_4, filtration of the drying agent, and evaporation of the solvent under vacuum, the crude product was purified by silica gel flash column chromatography, affording the products **2d-2f** in 73-85% isolated yields.

4. The general procedure for the PDMS-modulated DKR.

4.1 CALB method (The general procedure for the PDMS-modulated DKR of **1**).

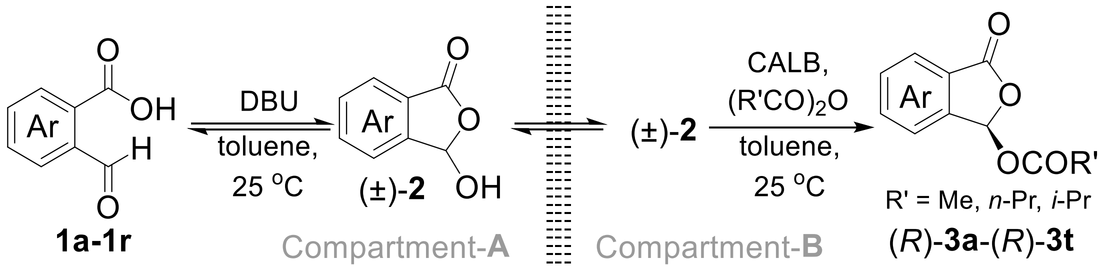

A typical procedure for a CALB method was as follows. In compartment-**A**, **1** (0.20 mmol), DBU (0.20 mmol) was introduced into 2.0 mL of toluene. In compartment-**B**, the Novozym-435 (CALB) (40.0 mg, 20 mg/0.1 mmol) and anhydride (0.40 mmol, 2.0 equiv.) were introduced into 2.0 mL of a mixture of toluene. A balloon filled with air (P = 1 atm) was connected to the top of both compartments. Both reactions in each compartment were stirred at 25 °C for 24-60 h. After this, the resulting solution in Compartment-**B** was filtered. The resulting toluene from the combined filtrates of Compartment-**B** and the solution in Compartment-**A** was removed using a rotary evaporator. The remaining solution was extracted with EtOAc (3 × 5 mL), and the collected organic phase was rinsed with brine (10 mL). After drying the organic layer over anhydrous Na_2SO_4, filtration of the drying agent, and evaporation of the solvent under vacuum, the crude product was purified by silica gel flash column chromatography,

affording the products ((*R*)-**3a**–(*R*)-**3t)** in 77-96% isolated yields. The enantiomeric excess (*ee*) values were determined using HPLC analysis with a Photo-Diode Array detector and a Daicel chiral cell column (Φ 0.46 × 25 cm).

4.2 PS-IM method (The general procedure for the PDMS-modulated DKR of **1**).

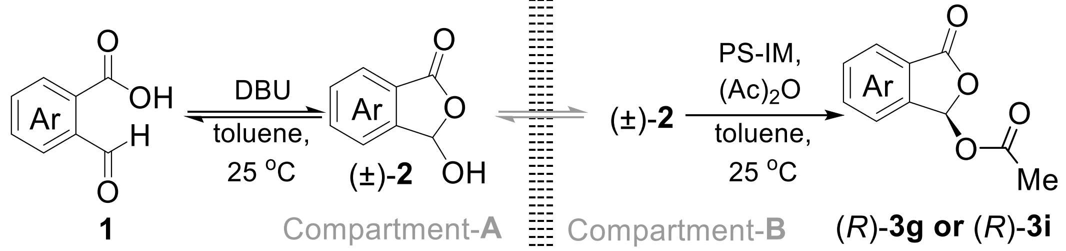

A typical procedure for a PS-IM method was as follows. In compartment-**A, 1** (0.20 mmol), DBU (0.20 mmol) was introduced into 2.0 mL of toluene. In compartment-**B**, the PS-IM (40.0 mg, 20 mg/0.1 mmol) and acetic anhydride (0.40 mmol, 2.0 equiv.) were introduced into 2.0 mL of a mixture of toluene. A balloon filled with air (P = 1 atm) was connected to the top of both compartments. Both reactions in each compartment were stirred at 25 °C for 72 h. After this, the resulting solution in Compartment-**B** was filtered. The resulting toluene from the combined filtrates of Compartment-**B** and the solution in Compartment-**A** was removed using a rotary evaporator. The remaining solution was extracted with EtOAc (3 × 5 mL), and the collected organic phase was rinsed with brine (10 mL). After drying the organic layer over anhydrous Na_2SO_4, filtration of the drying agent, and evaporation of the solvent under vacuum, the crude product was purified by silica gel flash column chromatography, affording the products in 73-82% isolated yields. The enantiomeric excess (*ee*) values were determined using HPLC analysis with a Photo-Diode Array detector and a Daicel chiral cell column (Φ 0.46 × 25 cm).

4.3 Traditional method (The general procedure for the PDMS-modulated DKR of **1**).

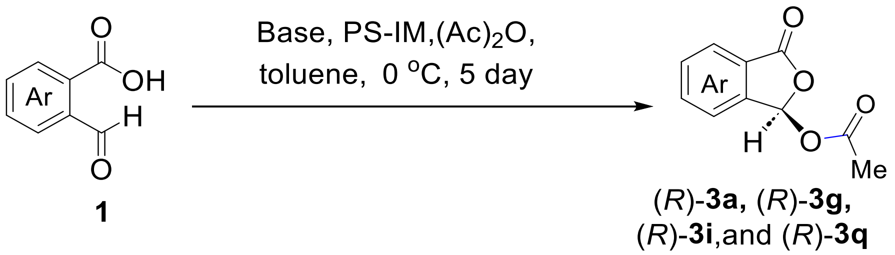

A typical procedure for a traditional DKR method was as follows. In a typical experiment, a mixture of compound **1** (1.0 equiv, 0.10 mmol), DBU (1.0 equiv, 0.10 mmol), and acetic anhydride (3.6 equiv, 0.36 mmol) in dry toluene (0.1 M) was added to a sealed-cap vial (1.75 mL) containing the CALB (40.0 mg, 20 mg/0.1 mmol) or the PS-IM (40.0 mg, 20 mg/0.1 mmol). The reaction mixture was kept and stirred at 0 °C for 72-120 h, at which time the reaction mixture was filtered and condensed under

vacuum. The crude product was purified by silica gel column chromatography to afford the products. The enantiomeric excess (*ee*) values were determined using HPLC analysis with a Photo-Diode Array detector and a Daicel chiral cell column (Φ 0.46 × 25 cm).

4.4 CALB method (The general procedure for the PDMS-modulated DKR of **4**).

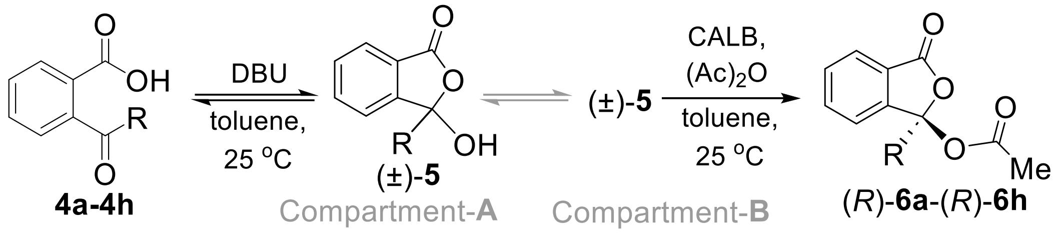

A typical procedure for a CALB method was as follows. In compartment-**A**, **4** (0.20 mmol), DBU (0.20 mmol) was introduced into 2.0 mL of toluene. In compartment-**B**, the Novozym-435 (CALB) (40.0 mg, 20 mg/0.1 mmol) and acetic anhydride (0.40 mmol, 2.0 equiv.) were introduced into 2.0 mL of a mixture of toluene. A balloon filled with air (P = 1 atm) was connected to the top of both compartments. Both reactions in each compartment were stirred at 25 °C for 48-72 h. After this, the resulting solution in Compartment-**B** was filtered. The resulting toluene from the combined filtrates of Compartment-**B** and the solution in Compartment-**A** was removed using a rotary evaporator. The remaining solution was extracted with EtOAc (3 × 5 mL), and the collected organic phase was rinsed with brine (10 mL). After drying the organic layer over anhydrous Na_2SO_4, filtration of the drying agent, and evaporation of the solvent under vacuum, the crude product was purified by silica gel flash column chromatography, affording the products ((*R*)-**6a**–(*R*)-**6h)** in 73-86% isolated yields. The enantiomeric excess (*ee*) values were determined using HPLC analysis with a Photo-Diode Array detector and a Daicel chiral cell column (Φ 0.46 × 25 cm).

5. General procedure for the time course investigation in the PDMS-modulated DKR of 1a

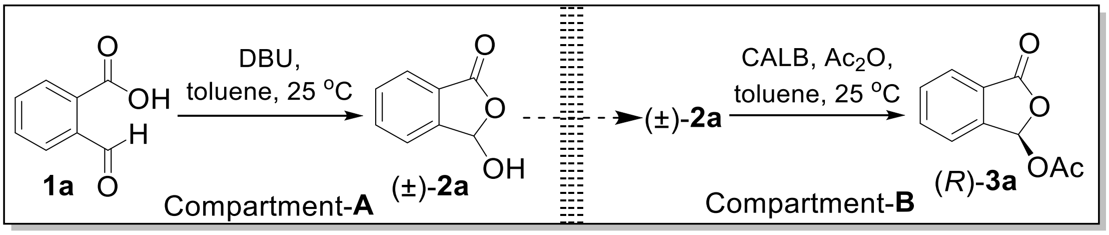

A typical procedure was as follows. In compartment-**A**, **1a** (2.0 mmol), DBU (2.0 mmol) was introduced into 10.0 mL of toluene. In compartment-**B**, the Novozym-435 (CALB) (400.0 mg, 20 mg/0.1 mmol) and acetic anhydride (4.0 mmol, 2.0 equiv.) were introduced into 10.0 mL of a mixture of toluene. A balloon filled with air (P = 1 atm) was connected to the top of both compartments. Both reactions in each compartment were stirred at 25 °C. During the 36 h reaction, the samples from both

Compartment-**A** and Compartment-**B** were individually collected at defined time intervals, analyzed by HPLC with an internal standard to determine the molar contents of **1a**, **2a**, and/or (*R*)-**3a**.

6. General procedure for the preparation of chiral phthalide prodrugs.

6.1 CALB method for the preparation of chiral phthalide prodrugs

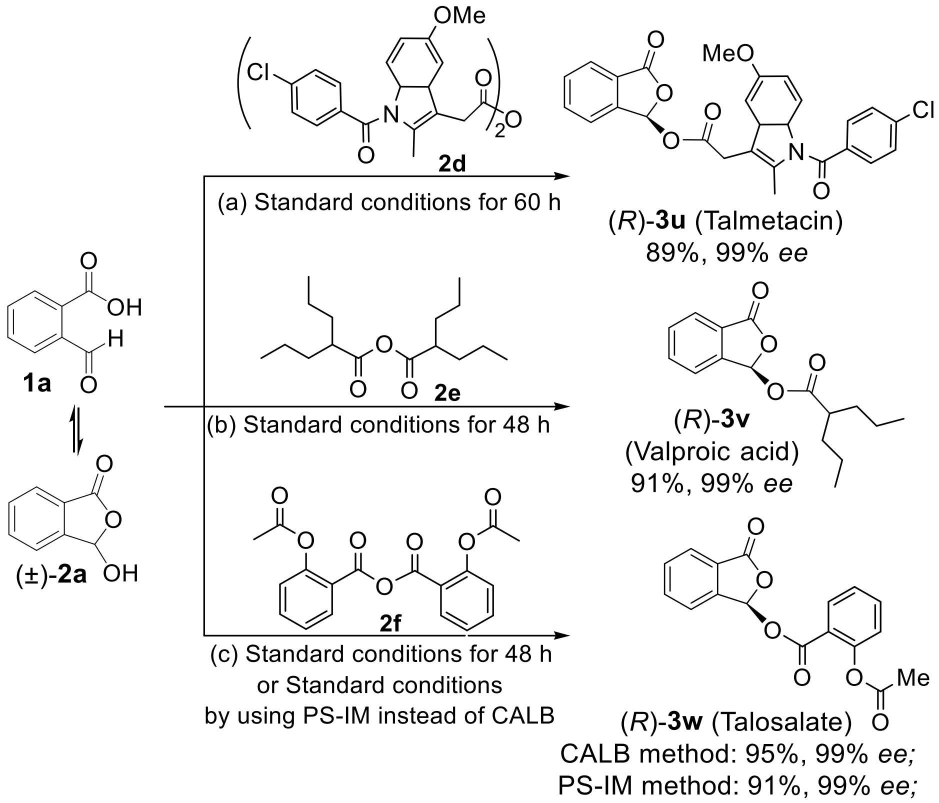

A typical procedure for a CALB method was as follows. In compartment-**A**, **1a** (0.20 mmol), DBU (0.20 mmol) was introduced into 2.0 mL of toluene. In compartment-**B**, the Novozym-435 (CALB) (40.0 mg, 20 mg/0.1 mmol) and anhydride (0.40 mmol, 2.0 equiv.) were introduced into 2.0 mL of a mixture of toluene. A balloon filled with air (P = 1 atm) was connected to the top of both compartments. Both reactions in each compartment were stirred at 25 °C for 48-72 h. After this, the resulting solution in Compartment-**B** was filtered. The resulting toluene from the combined filtrates of Compartment-**B** and the solution in Compartment-**A** was removed using a rotary evaporator. The remaining solution was extracted with EtOAc (3 × 5 mL), and the collected organic phase was rinsed with brine (10 mL). After drying the organic layer over anhydrous Na_2SO_4, filtration of the drying agent, and evaporation of the solvent under vacuum, the crude product was purified by silica gel flash column chromatography, affording the products ((*R*)-**3u**–(*R*)-**3w)** in 89-95% isolated yields. The enantiomeric excess (*ee*) values were determined using HPLC analysis with a Photo-Diode Array detector and a Daicel chiral cell column (Φ 0.46 × 25 cm).

6.2 PS-IM method for the preparation of chiral phthalide prodrugs

A typical procedure for a PS-IM method was as follows. In compartment-**A**, **1a** (0.20 mmol), DBU (0.20 mmol) was introduced into 2.0 mL of toluene. In compartment-**B**, the PS-IM (40.0 mg, 20 mg/0.1 mmol) and anhydride (0.60 mmol, 3.0 equiv.) were introduced into 2.0 mL of a mixture of toluene. A balloon filled with air (P = 1 atm) was connected to the top of both compartments. Both reactions in each compartment were stirred at 25 °C for 48 h. After this, the resulting solution in Compartment-**B** was filtered. The resulting toluene from the combined filtrates of Compartment-**B** and the solution in Compartment-**A** was removed using a rotary evaporator. The remaining solution was extracted with EtOAc (3 × 5 mL), and the collected organic phase was rinsed with brine (10 mL). After drying the organic layer over anhydrous Na_2SO_4, filtration of the drying agent, and evaporation of the solvent under vacuum, the crude product was purified by silica gel flash column chromatography, affording the products in 91% isolated yields. The enantiomeric excess (*ee*) values were determined using HPLC analysis with a Photo-Diode Array detector and a Daicel chiral cell column (Φ 0.46 × 25 cm).

6.3 General procedure for the preparation of product (*R*)-**7** and (*R*)-**8**.

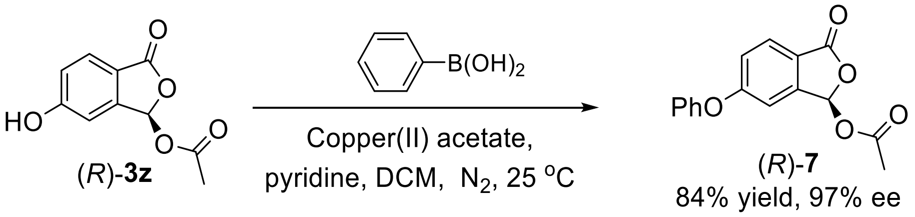

Under nitrogen protection, (*R*)-**3z** (0.20 mol), phenylboronic acid (0.40 mol), pyridine (0.80 mol), and copper(II) acetate monohydrate (0.20 mol) were successively added to dichloromethane (10 mL). The mixture was stirred at 25 °C for 48 h. After completion, the reaction was quenched with 2 N dilute hydrochloric acid, and the solids were removed by filtration. The combined organic phases were extracted with CH_2Cl_2 (5 mL × 3), dried over anhydrous magnesium sulfate, filtered, and concentrated under reduced pressure. The crude product was purified by silica gel column chromatography to afford the target product (*R*)-**7** (48.3 mg, 0.17 mmol) in 84% yield.

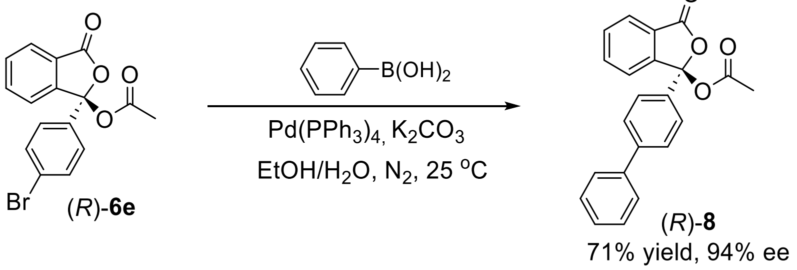

Under nitrogen protection, (*R*)-**6e** (0.20 mmol), phenylboronic acid (0.40 mmol), $Pd(PPh_3)_4$ (0.06 mmol), and potassium carbonate (0.20 mmol) were added to a mixture of ethanol/water (1:3, v/v, 10 mL). The reaction was stirred at 25 °C for 48 h. After completion, the mixture was poured into water

and extracted with CH_2Cl_2 (5 mL × 3). The combined organic phases were dried over anhydrous $MgSO_4$, filtered, and concentrated under reduced pressure. The crude product was purified by silica gel column chromatography to afford the target product (*R*)-**8** (48.8 mg, 0.14 mmol) in 71% yield.

6.4 General procedure for the preparation of (*S*)-**2x**.

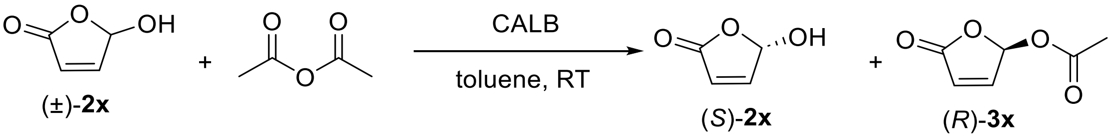

At room temperature, (±)-2x (0.20 mmol), acetic anhydride (0.25 mmol), and CALB (40 mg) were added to toluene (4.0 mL), and the resulting mixture was stirred for 5 h. After this, the solution was extracted with EtOAc (3 × 5 mL), and the collected organic phase was rinsed with brine (10 mL). After drying the organic layer over anhydrous Na_2SO_4, filtration of the drying agent, and evaporation of the solvent under vacuum, the crude product was purified by silica gel flash column chromatography, affording (*S*)-**2x** in 42% isolated yields. The enantiomeric excess (*ee*) values were determined using HPLC analysis with a Photo-Diode Array detector and a Daicel chiral cell column (Φ 0.46 × 25 cm).

7. General procedure for the reusability of the PDMS membrane.

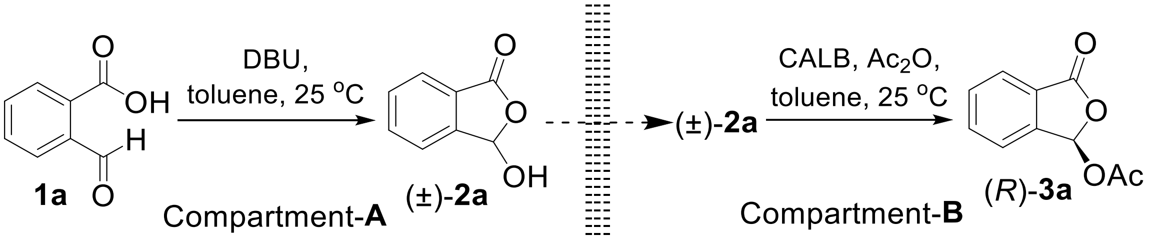

A typical procedure was as follows. In compartment-**A**, **1a** (0.20 mmol) and DBU (0.20 mmol) were introduced into 2.0 mL of toluene. In compartment-**B**, Novozym-435 (CALB) (40.0 mg, 20 mg/0.1 mmol) and acetic anhydride (0.40 mmol, 2.0 equiv.) were introduced into 2.0 mL of a mixture of toluene. A balloon filled with air (P = 1 atm) was connected to the top of both compartments. Both reactions in each compartment were stirred at 25 °C for 36 h. After this, the resulting solution in both compartments was removed, and the membrane in the reaction device was reused for the next cycle. The resulting solution was filtered, and toluene was removed using a rotary evaporator. The remaining solution was extracted with EtOAc (3 × 3 mL), and the collected organic phase was rinsed with brine (5 mL). After drying the organic layer over anhydrous Na_2SO_4, filtration of the drying agent, and evaporation of the solvent under vacuum, the crude product was purified by silica gel flash column chromatography, affording (*R*)-**3a**. The enantiomeric excess (*ee*) values were determined using HPLC analysis with a Photo-Diode Array detector and a Daicel chiral cell column (Φ 0.46 × 25 cm).

8. General procedure for the gram-scale preparation of (*R*)-3w.

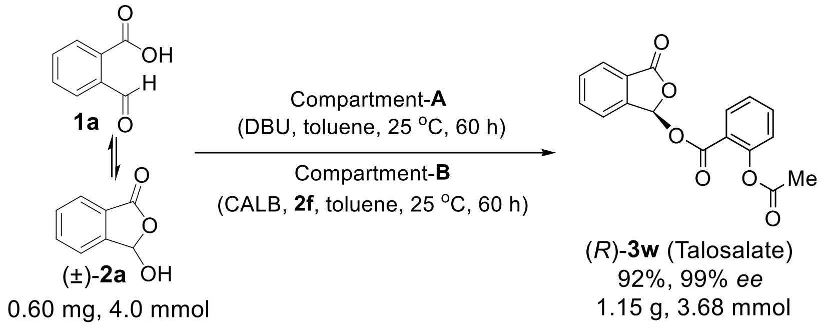

A typical procedure for a CALB method was as follows. In Compartment-**A**, **1a** (4.0 mmol), DBU (4.0 mmol) was introduced into 30.0 mL of toluene. In Compartment-**B**, the Novozym-435 (CALB) (0.80 g, 20 mg/0.1 mmol) and anhydride (**2f**) (8.0 mmol, 2.0 equiv.) were introduced into 30.0 mL of a mixture of toluene. A balloon filled with air (P = 1 atm) was connected to the top of both compartments. Both reactions in each compartment were stirred at 25 °C for 60 h. After this, the resulting solution in Compartment-**B** was filtered. The resulting toluene from the combined filtrates of Compartment-**B** and the solution in Compartment-**A** was removed using a rotary evaporator. The remaining solution was extracted with EtOAc (3 × 5 mL), and the collected organic phase was rinsed with brine (10 mL). After drying the organic layer over anhydrous Na_2SO_4, filtration of the drying agent, and evaporation of the solvent under vacuum, the crude product was purified by silica gel flash column chromatography, affording (*R*)-**3w** in 92% isolated yields. The enantiomeric excess (*ee*) values were determined using HPLC analysis with a Photo-Diode Array detector and a Daicel chiral cell column (Φ 0.46 × 25 cm).

9. Data of chiral products.

(*R*)-3a: (*R*)-3-oxo-1,3-dihydroisobenzofuran-1-yl acetate. White solid, 91% yield, 99%ee. ^{1}H NMR (400 MHz, Chloroform-d) δ 7.89-7.87 (d, *J* = 7.6 Hz, 1H), 7.75-7.71 (t, *J* = 7.5 Hz, 1H), 7.64–7.57 (m, 2H), 7.39 (s, 1H), 2.16 (s, 3H). ^{13}C NMR (101 MHz, Chloroform-d) δ 169.44, 167.87, 144.25, 134.88, 131.29, 126.40, 125.72, 123.61, 92.63, 20.79. HRMS (ESI): m/z [M+Na]$^+$ calcd for $C_{10}H_8O_4Na$: 215.0320; found 215.0306. HPLC (Chiralpak IC, elute: Hexanes/i-PrOH = 90/10, detector: 210 nm, flow rate: 1.0 mL/min, 25 °C).

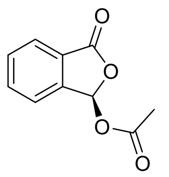

(*R*)-3b: (*R*)-4-fluoro-3-oxo-1,3-dihydroisobenzofuran-1-yl acetate. White solid, 79% yield, 99%*ee*. ^{1}H NMR (400 MHz, Chloroform-d) δ 7.78–7.73 (m, 1H), 7.40–7.37 (m, 2H), 7.29-7.25 (t, *J* = 8.5 Hz, 1H), 2.17 (s, 3H). ^{13}C NMR (101 MHz, Chloroform-d) δ 169.24, 160.60, 157.96, 146.57, 137.71, 137.64, 119.72, 119.68, 118.60, 118.41, 92.00, 20.74. ^{19}F NMR (376 MHz, Chloroform-d) δ-112.76. HRMS (ESI): m/z [M+Na]$^+$ calcd for $C_{10}H_7FO_4Na$: 233.0226; found: 233.0220. HPLC (Chiralpak OD, elute: Hexanes/*i*-PrOH = 90/10, detector: 210 nm, flow rate: 1.0 mL/min, 25 °C).

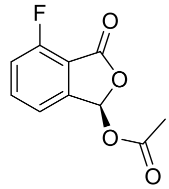

(*R*)-3c: (*R*)-5-fluoro-3-oxo-1,3-dihydroisobenzofuran-1-yl acetate. White solid, 94% yield, 99%*ee*. ^{1}H NMR (400 MHz, Chloroform-d) δ 7.60-7.55 (td, *J* = 10.0, 9.2, 5.6 Hz, 2H), 7.47-7.42 (m, 1H), 7.39 (s, 1H), 2.18 (s, 3H). ^{13}C NMR (101 MHz, Chloroform-d) δ 169.34, 166.55, 165.66, 163.15, 139.88, 139.86, 125.59, 125.50, 122.82, 122.58, 112.54, 112.30, 92.44, 20.77. ^{19}F NMR (376 MHz, Chloroform-d) δ -100.98. HRMS (ESI): m/z [M+Na]$^+$ calcd for $C_{10}H_7FO_4Na$: 233.1502; found: 233.1500. HPLC (Chiralpak IC, elute: Hexanes/*i*-PrOH = 80/20, detector: 210 nm, flow rate: 1.0 mL/min, 25 °C).

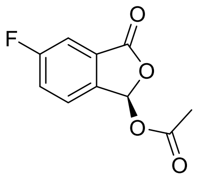

(*R*)-3d: (*R*)-6-fluoro-3-oxo-1,3-dihydroisobenzofuran-1-yl acetate. White oil, 85% yield, 98%*ee*. ^{1}H NMR (400 MHz, Chloroform-d) δ 7.90-7.87 (dd, *J* = 8.4, 4.6 Hz, 1H), 7.34 (s, 1H), 7.32-7.26 (m, *J* = 13.2, 8.0, 2.2 Hz, 2H), 2.17 (s, 3H). ^{13}C NMR (101 MHz, Chloroform-d) δ 169.28, 168.04, 166.61, 165.48, 147.12, 147.02, 128.21, 128.11, 122.48, 122.46, 119.52, 119.29, 111.37, 111.12, 91.83, 91.80, 20.70. ^{19}F NMR (376 MHz, Chloroform-d) δ -100.98. HRMS (ESI): m/z [M+Na]$^+$ calcd for $C_{10}H_7FO_4Na$: 233.1288; found: 210.1281. HPLC (Chiralpak IC, elute: Hexanes/*i*-PrOH = 80/20, detector: 210 nm, flow rate: 1.0 mL/min, 25 °C).

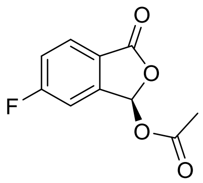

(*R*)-3e: (*R*)-5-chloro-3-oxo-1,3-dihydroisobenzofuran-1-yl acetate. Colorless oil, 93% yield, 99%*ee*.

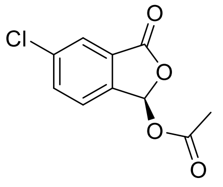

^{1}H NMR (400 MHz, Chloroform-d) δ 7.86 (d, *J* = 2.0 Hz, 1H), 7.71-7.69 (dd, *J* = 8.1, 1.9 Hz, 1H), 7.55-7.53 (d, *J* = 8.1 Hz, 1H), 7.38 (s, 1H), 2.18 (s, 3H). ^{13}C NMR (101 MHz, Chloroform-d) δ 169.29, 166.43, 142.42, 137.77, 135.12, 128.32, 125.74, 92.40, 20.77. HRMS (ESI): m/z [M+Na]$^+$ calcd for $C_{10}H_7ClO_4Na$: 248.9931; found: 248.9928. HPLC (Chiralpak IC, elute: Hexanes/*i*-PrOH = 80/20, detector: 254 nm, flow rate: 1.0 mL/min, 25 °C).

(*R*)-3f: (*R*)-6-chloro-3-oxo-1,3-dihydroisobenzofuran-1-yl acetate. White solid, 91% yield, 99%*ee*.

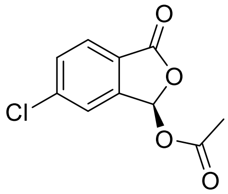

^{1}H NMR (400 MHz, Chloroform-d) δ 7.87-7.84 (d, *J* = 10.1 Hz, 1H), 7.63-7.59 (d, *J* = 16.7 Hz, 2H), 7.37 (s, 1H), 2.20 (s, 3H). ^{13}C NMR (101 MHz, Chloroform-d) δ 169.26, 166.70, 145.91, 141.62, 132.00, 126.96, 124.93, 124.13, 91.98, 20.77. HRMS (ESI): m/z [M+Na]$^+$ calcd for $C_{10}H_7ClO_4Na$: 248.8762; found: 248.8760. HPLC (Chiralpak IC, elute: Hexanes/*i*-PrOH = 80/20, detector: 254 nm, flow rate: 1.0 mL/min, 25 °C).

(*R*)-3g: (R)-7-chloro-3-oxo-1,3-dihydroisobenzofuran-1-yl acetate. White solid, 81% yield, 99%*ee*.

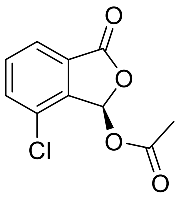

^{1}H NMR (400 MHz, Chloroform-d) δ 7.82-7.80 (d, *J* = 8.4 Hz, 1H), 7.69-7.67 (d, *J* = 6.9 Hz, 1H), 7.63-7.59 (t, *J* = 7.7 Hz, 1H), 7.48 (s, 1H), 2.18 (s, 3H). ^{13}C NMR (101 MHz, Chloroform-d) δ 168.84, 166.72, 141.48, 135.09, 132.96, 130.06, 128.77, 124.16, 90.93, 20.50. HRMS (ESI): m/z [M+Na]$^+$ calcd for $C_{10}H_7ClO_4Na$: 248.0448; found:248.0442. HPLC (Chiralpak IC, elute: Hexanes/*i*-PrOH = 80/20, detector: 210 nm, flow rate: 1.0 mL/min, 25 °C).

(*R*)-3h: (*R*)-4-bromo-3-oxo-1,3-dihydroisobenzofuran-1-yl acetate. White solid, 85% yield, 99%*ee*.

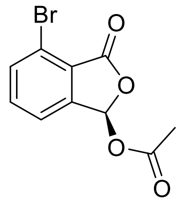

^{1}H NMR (400 MHz, Chloroform-d) δ 7.75-7.73 (d, *J* = 7.4 Hz, 1H), 7.59–7.53 (m, 2H), 7.30 (s, 1H), 2.15 (s, 3H). ^{13}C NMR (101 MHz, Chloroform-d) δ 169.31, 165.27, 146.59, 135.92, 124.75, 122.76, 120.79, 90.95, 20.77. HRMS (ESI): m/z [M+Na]$^+$ calcd for $C_{10}H_7BrO_4Na$: 292.9425; found:292.9420. HPLC (Chiralpak AD-H, elute: Hexanes/*i*-PrOH = 90/10, detector: 210 nm, flow rate: 1.0 mL/min, 25 °C).

(*R*)-3i: (*R*)-6-bromo-3-oxo-1,3-dihydroisobenzofuran-1-yl acetate. White solid, 91% yield, 98%*ee*.

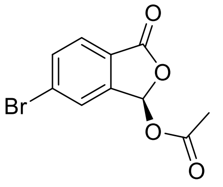

^{1}H NMR (400 MHz, Chloroform-d) δ 7.87-7.85 (d, *J* = 8.1 Hz, 1H), 7.64 –7.59 (m, 2H), 7.38 (s, 1H), 2.20 (s, 3H). ^{13}C NMR (101 MHz, Chloroform-d) δ 169.26, 145.91, 141.63, 132.01, 126.97, 124.94, 124.13, 91.98, 20.78. HRMS (ESI): m/z [M+Na]$^+$ calcd for $C_{10}H_7BrO_4Na$: 292.9619; found: 292.9617. HPLC (Chiralpak IC, elute: Hexanes/*i*-PrOH = 80/20, detector: 254 nm, flow rate: 1.0 mL/min, 25 °C).

(*R*)-3j: (*R*)-5-bromo-3-oxo-1,3-dihydroisobenzofuran-1-yl acetate. Colorless oil, 94% yield, 99%*ee*.

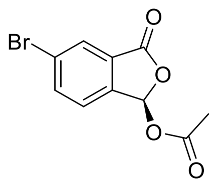

^{1}H NMR (400 MHz, Chloroform-d) δ 8.00 (s, 1H), 7.85-7.83 (dd, *J* = 8.1, 1.8 Hz, 1H), 7.49-7.47 (d, *J* = 8.1 Hz, 1H), 7.35 (s, 1H), 2.16 (s, 3H). ^{13}C NMR (101 MHz, Chloroform-d) δ 169.28, 166.31, 142.93, 137.94, 128.73, 128.47, 125.52, 125.18, 92.45, 20.76. HRMS (ESI): m/z [M+Na]$^+$ calcd for $C_{10}H_7BrO_4Na$: 292.6413; found: 292.6410. HPLC (Chiralpak IC, elute: Hexanes/*i*-PrOH = 80/20, detector: 210 nm, flow rate: 1.0 mL/min, 25 °C).

(*R*)-3k: (*R*)-7-bromo-3-oxo-1,3-dihydroisobenzofuran-1-yl acetate. White solid, 77% yield, 99%*ee*.

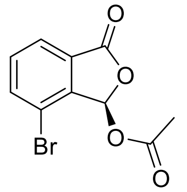

^{1}H NMR (400 MHz, Chloroform-d) δ 7.86-7.83 (dd, *J* = 7.7, 4.9 Hz, 2H), 7.53-7.51 (t, *J* = 7.7 Hz, 1H), 7.38 (s, 1H), 2.18 (s, 3H). ^{13}C NMR (101 MHz, Chloroform-d) δ 168.89, 166.75, 143.52, 133.05, 128.78, 124.71, 117.73, 91.74, 20.49. HRMS (ESI): m/z [M+Na]+ calcd for $C_{10}H_7BrO_4Na$: 292.9177; found: 292.9372. HPLC (Chiralpak IC, elute: Hexanes/i-PrOH = 80/20, detector: 210 nm, flow rate: 1.0 mL/min, 25 °C).

(*R*)-3l: (*R*)-5-iodo-3-oxo-1,3-dihydroisobenzofuran-1-yl acetate. White solid, 89% yield, 99%*ee*.

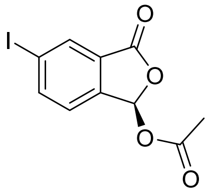

^{1}H NMR (400 MHz, Chloroform-d) δ 8.22-8.21 (d, *J* = 1.5 Hz, 1H), 8.05-8.03 (dd, *J* = 8.0, 1.5 Hz, 1H), 7.36-7.35 (d, *J* = 8.2 Hz, 2H), 2.17 (s, 3H). ^{13}C NMR (101 MHz, Chloroform-d) δ 169.28, 166.17, 143.65, 143.59, 134.74, 125.22, 96.78, 92.53, 20.79. HRMS (ESI): m/z [M+Na]$^+$ calcd for $C_{10}H_7IO_4Na$: 340.9288; found: 340.9280. HPLC (Chiralpak IC, elute: Hexanes/*i*-PrOH = 80/20, detector: 210 nm, flow rate: 1.0 mL/min, 25 °C).

(*R*)-3m: (*R*)-5-cyano-3-oxo-1,3-dihydroisobenzofuran-1-yl acetate. White solid, 90% yield, 99%*ee*.

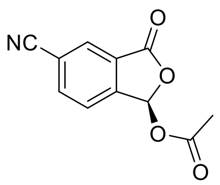

^{1}H NMR (400 MHz, Chloroform-d) δ 8.18 (s, 1H), 8.04-8.01 (dd, *J* = 7.9, 1.5 Hz, 1H), 7.79-7.77 (d, *J* = 7.9 Hz, 1H), 7.44 (s, 1H), 2.18 (s, 3H). ^{13}C NMR (101 MHz, Chloroform-d) δ 169.08, 165.67, 147.84, 138.09, 129.72, 127.76, 125.10, 116.97, 115.75, 92.31, 20.70. HRMS (ESI): m/z [M+Na]$^+$ calcd for $C_{10}H_7CNO_4Na$: 240.0276; found: 240.0272. HPLC (Chiralpak IC, elute: Hexanes/i-PrOH = 80/20, detector: 210 nm, flow rate: 1.0 mL/min, 25 °C).

(*R*)-3n: (*R*)-5-nitro-3-oxo-1,3-dihydroisobenzofuran-1-yl acetate. White solid, 93% yield, 99%*ee*.

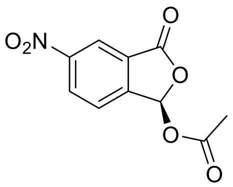

^{1}H NMR (400 MHz, Chloroform-d) δ 8.74 (s, *J* = 2.0 Hz, 1H), 8.63–8.61 (m, 1H), 7.83-7.81 (d, *J* = 8.3 Hz, 1H), 7.50 (s, 1H), 2.22 (s, 3H). ^{13}C NMR (101 MHz, Chloroform-d) δ 168.99, 165.35, 150.39, 149.23, 129.74, 128.47, 125.19, 121.34, 92.17, 20.72. HRMS (ESI): m/z [M+Na]$^+$ calcd for $C_{10}H_7NO_6Na$: 260.0171; found: 260.0170. HPLC (Chiralpak IC, elute: Hexanes/i-PrOH = 80/20, detector: 210 nm, flow rate: 1.0 mL/min, 25 °C).

(*R*)-3o: (*R*)-6-methyl-3-oxo-1,3-dihydroisobenzofuran-1-yl acetate. White solid, 96% yield, 99%*ee*.

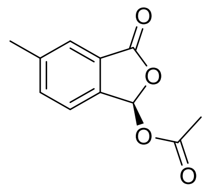

^{1}H NMR (400 MHz, Chloroform-d) δ 7.68 (s, 1H), 7.54-7.53 (d, *J* = 7.8 Hz, 1H), 7.46-7.44 (d, *J* = 7.9 Hz, 1H), 7.35 (s, 1H), 2.47 (s, 3H), 2.16 (s, 3H). ^{13}C NMR (101 MHz, Chloroform-d) δ 169.53, 168.04, 141.93, 135.90, 135.23, 125.78, 123.26, 121.83, 92.66, 21.41, 20.83. HRMS (ESI): m/z [M+Na]$^+$ calcd for $C_{11}H_{10}O_4Na$: 229.0477; found: 229.0471. HPLC (Chiralpak IC, elute: Hexanes/*i*-PrOH = 80/20, detector: 210 nm, flow rate: 1.0 mL/min, 25 °C).

(*R*)-3p: (*R*)-6-methyl-3-oxo-1,3-dihydroisobenzofuran-1-yl acetate. White solid, 92% yield, 99%*ee*.

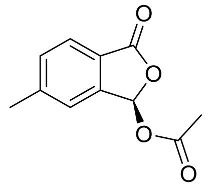

^{1}H NMR (400 MHz, Chloroform-d) δ 7.83 - 7.81 (d, J = 7.8 Hz, 1H), 7.47 - 7.45 (d, J = 7.8 Hz, 1H), 7.39 (s, 2H), 2.53 (s, 3H), 2.20 (s, 3H). ^{13}C NMR (101 MHz, Chloroform-d) δ 169.48, 167.85, 146.32, 144.83, 125.57, 123.86, 22.07, 20.83. HRMS (ESI): m/z [M+Na]$^+$ calcd for $C_{11}H_{10}O_4Na$: 229.0477; found: 229.0471. HPLC (Chiralpak IC, elute: Hexanes/*i*-PrOH = 80/20, detector: 210 nm, flow rate: 1.0 mL/min, 25 °C).

(*R*)-3q: (*R*)-6-methoxy-3-oxo-1,3-dihydroisobenzofuran-1-yl acetate. White solid, 88% yield,

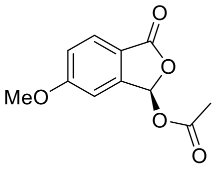

99%*ee*. ^{1}H NMR (400 MHz, Chloroform-d) δ 7.74-7.72 (d, *J* = 8.5 Hz, 1H), 7.27-7.26 (d, *J* = 2.5 Hz, 1H), 7.08-7.06 (d, *J* = 8.5 Hz, 1H), 6.99 (d, *J* = 2.3 Hz, 1H), 3.88 (s, 3H), 2.14 (s, 3H). ^{13}C NMR (101 MHz, Chloroform-d) δ 169.45, 167.56, 165.29, 147.09, 127.15, 118.53, 118.51, 92.03, 56.06, 20.77. HRMS (ESI): m/z [M+Na]$^+$ calcd for Chemical Formula: $C_{11}H_{10}O_5Na$: 245.0426; found: 245.0420. HPLC (Chiralpak IC, elute: Hexanes/*i*-PrOH = 80/20, detector: 254 nm, flow rate: 1.0 mL/min, 25 °C).

(*R*)-3r: (*R*)-5-oxo-5,7-dihydrofuro[3,4-b]pyridin-7-yl acetate. White solid, 81% yield, 98%*ee*. ^{1}H

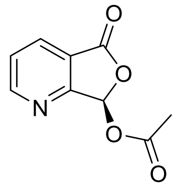

NMR (400 MHz, Chloroform-d) δ 8.95 - 8.93 (d, *J* = 4.9 Hz, 1H), 8.25 - 8.23 (d, *J* = 7.8 Hz, 1H), 7.62 - 7.59 (dd, *J* = 7.8, 4.9 Hz, 1H), 7.39 (s, 1H), 2.21 (s, 3H). ^{13}C NMR (101 MHz, Chloroform-d) δ 168.92, 165.88, 163.48, 156.00, 134.24, 125.79, 120.95, 91.82, 20.66. HRMS (ESI): m/z [M+Na]$^+$ calcd for $C_{11}H_{10}O_4Na$: 229.0477; found: 229.0471. HPLC (Chiralpak IC, elute: Hexanes/*i*-PrOH = 80/20, detector: 210 nm, flow rate: 1.0 mL/min, 25 °C).

(*R*)-3s: (*R*)-3-oxo-1,3-dihydroisobenzofuran-1-yl butyrate. White solid, 84% yield, 99%*ee*. ^{1}H

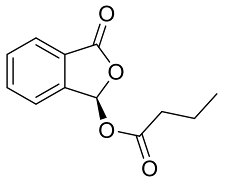

NMR (400 MHz, Chloroform-d) δ 7.90-7.88 (d, *J* = 7.6 Hz, 1H), 7.75-7.71 (t, *J* = 7.5 Hz, 1H), 7.65–7.56 (m, 2H), 7.42 (s, 1H), 2.40-2.36 (t, *J* = 7.4 Hz, 2H), 1.70-1.65 (q, *J* = 7.3 Hz, 2H), 0.97-0.93 (t, *J* = 7.5 Hz, 3H). ^{13}C NMR (101 MHz, Chloroform-d) δ 172.10, 167.93, 144.41, 134.85, 131.23, 126.46, 125.72, 123.53, 92.56, 35.81, 18.06, 13.50. HRMS (ESI): m/z [M+Na]$^+$ calcd for $C_{12}H_{12}O_4Na$: 243.0633; found:

243.0629. HPLC (Chiralpak IC, elute: Hexanes/i-PrOH = 80/20, detector: 210 nm, flow rate: 1.0 mL/min, 25 °C).

(*R*)-3t: (*R*)-3-oxo-1,3-dihydroisobenzofuran-1-yl isobutyrate. White solid, 79% yield, 99%*ee*. ^{1}H NMR (400 MHz, Chloroform-d) δ 7.88-7.86 (d, *J* = 7.6 Hz, 1H), 7.74-7.70 (t, *J* = 7.5 Hz, 1H), 7.63-7.55 (dd, *J* = 26.9, 7.1 Hz, 2H), 7.39 (s, 1H), 2.64-2.57 (p, *J* = 7.0 Hz, 1H), 1.18-1.16 (dd, *J* = 7.0, 2.9 Hz, 6H). ^{13}C NMR (101 MHz, Chloroform-d) δ 175.52, 167.97, 144.45, 134.89, 131.23, 126.43, 125.68, 123.50, 92.63, 33.85, 18.58. HRMS (ESI): m/z [M+Na]$^{+}$ calcd for $C_{12}H_{12}O_4Na$: 243.0512; found: 243.0512. HPLC (Chiralpak IC, elute: Hexanes/i-PrOH = 80/20, detector: 210 nm, flow rate: 1.0 mL/min, 25 °C).

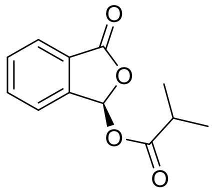

(*R*)-3u: (*R*)-3-oxo-1,3-dihydroisobenzofuran-1-yl 2-(1-(4-chlorobenzoyl)-5-methoxy-2-methyl-3a,7a-dihydro-1H-indol-3-yl)acetate. Yellow solid, 89% yield, 98%*ee*. ^{1}H NMR (400 MHz, Chloroform-d) δ 7.95 - 7.93 (d, *J* = 7.5 Hz, 1H), 7.74-7.70 (t, *J* = 7.5 Hz, 1H), 7.67 - 7.65 (d, *J* = 8.5 Hz, 3H), 7.48 - 7.46 (d, *J* = 8.5 Hz, 3H), 7.42 (s, 1H), 6.91 - 6.87 (d, *J* = 13.7 Hz, 2H), 6.69 (d, *J* = 9.0 Hz, 1H), 3.78 (s, 2H), 3.77 (s, 3H), 2.36 (s, 3H). ^{13}C NMR (101 MHz, Chloroform-d) δ 169.36, 168.29, 167.72, 156.13, 144.16, 139.46, 136.31, 134.85, 133.74, 131.37, 131.22, 130.83, 130.22, 126.47, 125.88, 123.53, 115.03, 111.21, 101.13. HRMS (ESI): m/z [M+Na]$^{+}$ calcd for $C_{27}H_{22}ClO_4Na$: 514.1033; found: 514.1011. HPLC (Chiralpak IC, elute: Hexanes/*i*-PrOH = 80/20, detector: 210 nm, flow rate: 1.0 mL/min, 25 °C).

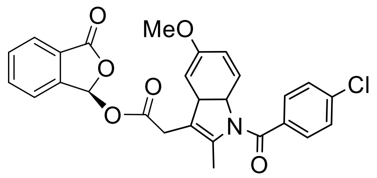

(*R*)-3v: (*R*)-3-oxo-1,3-dihydroisobenzofuran-1-yl 2-propylpentanoate. White solid, 91% yield, 99%*ee*. ^{1}H NMR (400 MHz, Chloroform-d) δ 7.88-7.87 (d, *J* = 7.6 Hz, 1H), 7.74 - 7.70 (t, *J* = 7.5 Hz, 1H), 7.63-7.60 (t, *J* = 7.5 Hz, 1H), 7.53-7.51 (d, *J* = 7.6 Hz, 1H), 7.42 (s, 1H), 2.46-7.39 (dt, *J* = 9.2, 5.1 Hz, 1H), 1.65-1.56 (m, 2H), 1.44 - 1.40 (m, 2H), 1.32 - 1.29 (m, 4H), 0.88 - 0.83 (td, *J* = 7.3, 4.0 Hz, 6H). ^{13}C NMR (101 MHz, Chloroform-d) δ 174.93, 167.95, 144.55, 134.83, 131.18, 126.51, 125.68, 123.38, 92.53, 45.06, 34.23, 34.19, 20.49, 20.44, 13.89, 13.87. HRMS (ESI): m/z [M+Na]$^{+}$ calcd for $C_{16}H_{20}O_4Na$: 229.1259; found: 229.1241. HPLC (Chiralpak IC, elute: Hexanes/*i*-PrOH = 80/20, detector: 210 nm, flow rate: 1.0 mL/min, 25 °C).

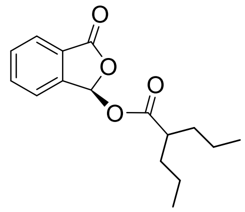

(*R*)-3w: (*R*)-3-oxo-1,3-dihydroisobenzofuran-1-yl 2-acetoxybenzoate. White solid, 95% yield, 99%*ee*. ^{1}H NMR (400 MHz, Chloroform-d) δ 8.01 - 7.93 (dd, *J* = 24.4, 7.7 Hz, 2H), 7.77 - 7.74 (m, 1H), 7.68 - 7.58 (m, 4H), 7.31 - 7.27 (t, *J* = 7.7 Hz, 1H), 7.12 - 7.10 (d, *J* = 8.1 Hz, 1H), 2.19 (s, 3H). ^{13}C NMR (101 MHz, Chloroform-d) δ 169.51, 167.77, 162.87, 151.19, 144.26, 135.08, 135.01, 132.17, 131.45, 126.43, 126.19, 125.82, 124.15, 123.93, 121.60, 93.19, 20.79. HRMS (ESI):

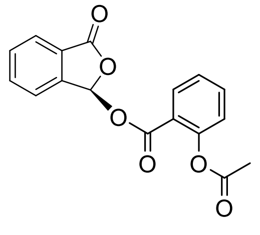

m/z [M+Na]+ calcd for $C_{17}H_{12}O_4Na$: 335.0532; found: 335.0541. HPLC (Chiralpak IC, elute: Hexanes/*i*-PrOH = 80/20, detector: 210 nm, flow rate: 1.0 mL/min, 25 °C).

(*R*)-3x: (*R*)-5-oxo-2,5-dihydrofuran-2-yl acetate. Pale yellow oil, 81% yield, 99%*ee*. ^{1}H NMR (400 MHz, Chloroform-d) δ 7.33–7.26 (m, 1H), 6.94 (s, 1H), 6.29-6.27 (dd, *J* = 5.6, 1.3 Hz, 1H), 2.12 (s, 3H). ^{13}C NMR (101 MHz, Chloroform-d) δ 169.78, 168.95, 149.95, 125.09, 93.81, 20.59. HRMS (ESI): m/z [M+H]+ calcd for $C_6H_6O_4Na$: 165.0164; found: 165.0158. HPLC (Chiralpak IC, elute: Hexanes/i-PrOH = 90/10, detector: 210 nm, flow rate: 1.0 mL/min, 25 °C).

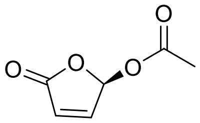

(*R*)-3y: (R)-chroman-2-yl acetate. Pale yellow oil, 87% yield, 98%*ee*. ^{1}H NMR (400 MHz, Chloroform-d) δ 7.16 - 7.08 (m, 2H), 6.95 - 6.87 (m, 2H), 6.53 (s, 1H), 3.04 - 2.95 (m, 1H), 2.75 - 2.69 (ddd, *J* = 16.3, 5.8, 2.9 Hz, 1H), 2.16 - 2.10 (dq, *J* = 14.0, 3.0 Hz, 1H), 2.08 (s, 3H), 2.06 - 2.01 (m, 1H). ^{13}C NMR (101 MHz, Chloroform-d) δ 169.91, 151.52, 129.26, 127.63, 121.69, 121.42, 117.10, 90.23, 25.06, 21.23, 19.66. HRMS (ESI): m/z [M+H]+ calcd for $C_{11}H_{12}O_3Na$: 215.0664; found: 215.0658. HPLC (Chiralpak IC, elute: Hexanes/i-PrOH = 98/02, detector: 210 nm, flow rate: 1.0 mL/min, 25 °C).

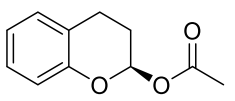

(*R*)-3z: (*R*)-6-hydroxy-3-oxo-1,3-dihydroisobenzofuran-1-yl acetate. Pale yellow oil, 85% yield, 97%*ee*. ^{1}H NMR (400 MHz, Chloroform-d) δ 7.92 - 7.87 (m, 1H), 7.54 - 7.51 (m, 2H), 7.44 - 7.39 (t, *J* = 8.5 Hz, 1H), 2.32 (s, 3H). ^{13}C NMR (101 MHz, Chloroform-d) δ 169.41, 166.54, 142.54, 137.88, 135.24, 128.43, 125.85, 125.02, 92.52, 20.88. HRMS (ESI): m/z [M+H]+ calcd for $C_{10}H_8O_5Na$: 231.0269; found: 231.0238. HPLC (Chiralpak IC, elute: Hexanes/i-PrOH = 90/10, detector: 210 nm, flow rate: 1.0 mL/min, 25 °C).

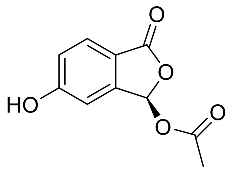

(*R*)-6a: (*R*)-1-methyl-3-oxo-1,3-dihydroisobenzofuran-1-yl acetate. Pale yellow oil, 84% yield, 99%*ee*. ^{1}H NMR (400 MHz, Chloroform-d) δ 7.76-7.74 (d, *J* = 7.6 Hz, 1H), 7.65-7.61 (t, *J* = 7.5 Hz, 1H), 7.53–7.47 (m, 2H), 1.91 (s, 3H), 1.86 (s, 3H). ^{13}C NMR (101 MHz, Chloroform-d) δ 168.22, 167.60, 147.84, 134.68, 130.53, 126.75, 125.19, 121.79, 105.01, 25.15, 21.53. HRMS (ESI): m/z [M+Na]+ calcd for $C_{11}H_{10}O_4Na$: 229.0477; found: 229.0473. HPLC (Chiralpak IC, elute: Hexanes/i-PrOH = 80/20, detector: 210 nm, flow rate: 1.0 mL/min, 25 °C).

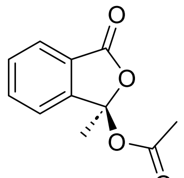

(*R*)-6b: (*R*)-3-oxo-1-phenyl-1,3-dihydroisobenzofuran-1-yl acetate. White solid, 79% yield, 99%*ee*. ^{1}H NMR (400 MHz, Chloroform-d) δ 7.93-7.91 (d, *J* = 7.6 Hz, 1H), 7.68-7.64 (t, *J* = 7.5 Hz, 1H), 7.59-7.51 (m, 4H), 7.41-7.39 (dd, *J* = 5.2, 2.1 Hz, 3H), 2.12 (s, 3H). ^{13}C NMR (101 MHz, Chloroform-d) δ 168.01, 167.93, 147.84, 137.00, 134.74, 130.60, 129.64, 128.84, 126.51, 125.60, 125.15, 122.98, 105.02, 21.75. HRMS (ESI): m/z [M+Na]+

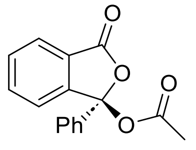

calcd for $C_{16}H_{12}O_4Na$: 291.0633; found: 291.0621. HPLC (Chiralpak IC, elute: Hexanes/*i*-PrOH = 80/20, detector: 210 nm, flow rate: 1.0 mL/min, 25 °C).

(*R*)-6c: (*R*)-1-(4-fluorophenyl)-3-oxo-1,3-dihydroisobenzofuran-1-yl acetate. White solid, 81% yield, 99%*ee*. ^{1}H NMR (400 MHz, Chloroform-d) δ 7.93-7.91 (d, *J* = 7.5 Hz, 1H), 7.70-7.66 (t, *J* = 8.1 Hz, 1H), 7.60-7.49 (m, 4H), 7.10-7.05 (t, *J* = 8.7 Hz, 2H), 2.11 (s, 3H). ^{13}C NMR (101 MHz, Chloroform-d) δ 167.92, 167.67, 164.57, 162.09, 147.53, 134.84, 133.03, 133.00, 130.75, 127.41, 127.33, 126.53, 125.67, 122.92, 115.94, 115.72, 104.64, 21.72. ^{19}F NMR (376 MHz, Chloroform-d) δ -111.44. HRMS (ESI): m/z $[M+Na]^+$ calcd for $C_{16}H_{11}FO_4Na$: 309.0539; found: 309.0512. HPLC (Chiralpak IC, elute: Hexanes/*i*-PrOH = 80/20, detector: 210 nm, flow rate: 1.0 mL/min, 25 °C).

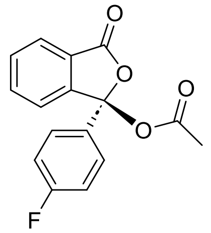

(*R*)-6d: (*R*)-1-(4-chlorophenyl)-3-oxo-1,3-dihydroisobenzofuran-1-yl acetate. White solid, 84% yield, 99%*ee*. ^{1}H NMR (400 MHz, Chloroform-d) δ 7.92-7.90 (d, *J* = 7.6 Hz, 1H), 7.69-7.65 (t, *J* = 7.5 Hz, 1H), 7.59-7.56 (t, *J* = 7.5 Hz, 1H), 7.51-7.48 (m, 3H), 7.37-7.34 (d, *J* = 8.7 Hz, 2H), 2.11 (s, 3H). ^{13}C NMR (101 MHz, Chloroform-d) δ 167.90, 167.64, 147.42, 135.69, 134.95, 130.83, 129.07, 126.70, 126.37, 125.69, 122.91, 104.49, 21.70. HRMS (ESI): m/z $[M+Na]^+$ calcd for $C_{16}H_{11}ClO_4Na$: 325.0244; found: 325.0252. HPLC (Chiralpak IC, elute: Hexanes/*i*-PrOH = 80/20, detector: 210 nm, flow rate: 1.0 mL/min, 25 °C).

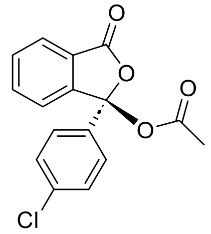

(*R*)-6e: (*R*)-1-(4-bromophenyl)-3-oxo-1,3-dihydroisobenzofuran-1-yl acetate. White solid, 86% yield, 99%*ee*. ^{1}H NMR (400 MHz, Chloroform-d) δ 7.92-7.90 (d, *J* = 7.5 Hz, 1H), 7.69 -7.65 (m, 1H), 7.70-7.49 (dt, *J* = 29.7, 7.9 Hz, 4H), 7.43-7.41 (d, *J* = 8.7 Hz, 2H), 2.11 (s, 3H). ^{13}C NMR (101 MHz, Chloroform-d) δ 167.88, 167.63, 147.37, 136.21, 134.95, 132.05, 130.85, 126.95, 126.34, 125.72, 123.95, 122.90, 104.51, 21.72. HRMS (ESI): m/z $[M+Na]^+$ calcd for $C_{16}H_{11}BrO_4Na$: 368.9738; found: 368.9710. HPLC (Chiralpak IC, elute: Hexanes/*i*-PrOH = 80/20, detector: 210 nm, flow rate: 1.0 mL/min, 25 °C).

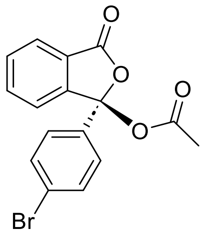

(*R*)-6f: (R)-3-oxo-1-(p-tolyl)-1,3-dihydroisobenzofuran-1-yl acetate. White solid, 76% yield, 99%*ee*. ^{1}H NMR (400 MHz, Chloroform-d) δ 7.92-7.90 (d, *J* = 7.6 Hz, 1H), 7.68-7.64 (d, *J* = 8.6 Hz, 1H), 7.58-7.51 (m, 2H), 7.45-7.43 (d, *J* = 8.3 Hz, 2H), 7.22-7.20 (d, *J* = 8.1 Hz, 2H), 2.35 (s, 3H), 2.11 (s, 3H). ^{13}C NMR (101 MHz, Chloroform-d) δ 168.05, 167.97, 147.96, 139.70, 134.70, 134.10, 130.52, 129.50, 126.55, 125.54, 125.11, 122.99, 105.20, 21.77, 21.19. HRMS (ESI): m/z $[M+Na]^+$ calcd for $C_{17}H_{14}O_4Na$: 305.0790; found: 305.0771. HPLC (Chiralpak IC, elute: Hexanes/*i*-PrOH = 80/20, detector: 210 nm, flow rate: 1.0 mL/min, 25 °C).

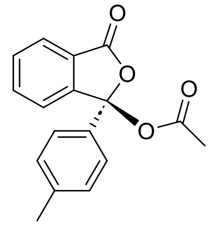

(*R*)-6g: (*R*)-1-(4-ethylphenyl)-3-oxo-1,3-dihydroisobenzofuran-1-yl acetate. White solid, 73% yield, 99%*ee*. ^{1}H NMR (400 MHz, Chloroform-d) δ 7.92-7.90 (d, *J* = 7.5 Hz, 1H), 7.68-7.64 (t, *J* = 7.5 Hz, 1H), 7.58-7.52 (m, 2H), 7.48-7.46 (d, *J* = 8.3 Hz, 2H), 7.26-7.23 (t, *J* = 6.9 Hz, 2H), 2.68-7.62 (q, *J* = 7.5 Hz, 2H), 2.11 (s, 3H), 1.24-1.20 (t, J = 7.6 Hz, 3H). ^{13}C NMR (101 MHz, Chloroform-d) δ 168.06, 167.97, 147.94, 145.94, 134.68, 134.27, 130.51, 128.31, 126.58, 125.53, 125.20, 123.00, 105.22, 28.55, 21.77, 15.33. HRMS (ESI): m/z [M+Na]$^+$ calcd for $C_{18}H_{16}O_4Na$: 319.0946; found: 319.0921. HPLC (Chiralpak IC, elute: Hexanes/*i*-PrOH = 80/20, detector: 210 nm, flow rate: 1.0 mL/min, 25 °C).

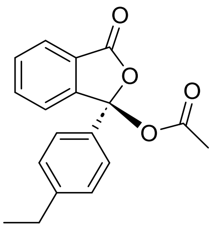

(*R*)-6h: (*R*)-3-oxo-1-(thiophen-2-yl)-1,3-dihydroisobenzofuran-1-yl acetate. Yellow solid, 74% yield, 98%*ee*. ^{1}H NMR (400 MHz, Chloroform-d) δ 7.93-7.91 (d, *J* = 7.5 Hz, 1H), 7.72 - 7.61 (m, 3H), 7.39 - 7.38 (m, 1H), 7.11 - 6.98 (m, 2H), 2.10 (s, 3H). ^{13}C NMR (101 MHz, Chloroform-d) δ 189.16, 188.19, 167.81, 167.12, 146.92, 139.81, 134.68, 130.93, 127.47, 126.88, 126.65, 125.66, 123.08, 21.71. HRMS (ESI): m/z [M+Na]$^+$ calcd for $C_{14}H_{10}SO_4Na$: 297.0197; found: 297.0157. HPLC (Chiralpak IC, elute: Hexanes/*i*-PrOH = 80/20, detector: 210 nm, flow rate: 1.0 mL/min, 25 °C).

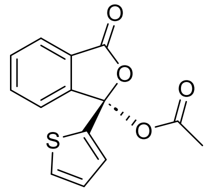

(*R*)-7: (*R*)-3-oxo-1-(thiophen-2-yl)-1,3-dihydroisobenzofuran-1-yl acetate. Yellow oil, 84% yield, 97%*ee*. ^{1}H NMR (400 MHz, Chloroform-d) δ 7.82 - 7.80 (d, *J* = 8.4 Hz, 1H), 7.45 - 7.40 (m, 2H), 7.29 - 7.22 (m, 2H), 7.18 - 7.16 (dd, *J* = 8.4, 2.2 Hz, 1H), 7.10 - 7.08 (d, *J* = 7.5 Hz, 2H), 7.03 (d, *J* = 2.1 Hz, 1H), 2.15 (s, 3H). ^{13}C NMR (101 MHz, Chloroform-d) δ 169.34, 167.27, 164.01, 154.71, 146.86, 130.41, 127.58, 125.49, 120.64, 120.55, 120.04, 111.38, 91.86, 20.81. HRMS (ESI): m/z [M+Na]$^+$ calcd for $C_{16}H_{12}O_5Na$: 307.0887; found: 307.0852. HPLC (Chiralpak IC, elute: Hexanes/*i*-PrOH = 90/10, detector: 210 nm, flow rate: 1.0 mL/min, 25 °C).

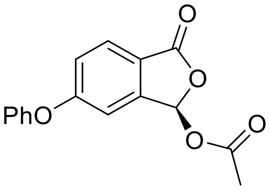

(*R*)-8: (*R*)-3-oxo-1-(thiophen-2-yl)-1,3-dihydroisobenzofuran-1-yl acetate. Yellow solid, 71% yield, 94%*ee*. ^{1}H NMR (400 MHz, Chloroform-d) δ 7.96 - 7.94 (d, *J* = 7.6 Hz, 1H), 7.71 - 7.68 (t, *J* = 7.5 Hz, 1H), 7.62 (s, 5H), 7.60 - 7.56 (d, *J* = 6.9 Hz, 3H), 7.47 - 7.43 (m, 2H), 7.39 - 7.35 (d, *J* = 7.3 Hz, 1H), 2.15 (s, 3H). ^{13}C NMR (101 MHz, Chloroform-d) δ 168.00, 147.77, 142.61, 140.13, 135.84, 134.72, 130.65, 130.63, 128.89, 127.81, 127.56, 127.18, 126.59, 125.70, 125.65, 123.00, 21.82. HRMS (ESI): m/z [M+Na]$^+$ calcd for $C_{14}H_{10}SO_4Na$: 367.0946; found: 367.0944. HPLC (Chiralpak IC, elute: Hexanes/*i*-PrOH = 90/10, detector: 210 nm, flow rate: 1.0 mL/min, 25 °C).

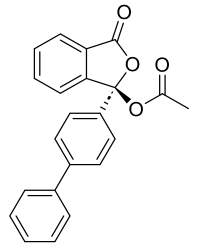

Supplementary Table 1. Optimizing reaction conditions for the traditional DKR of 2a. [a]

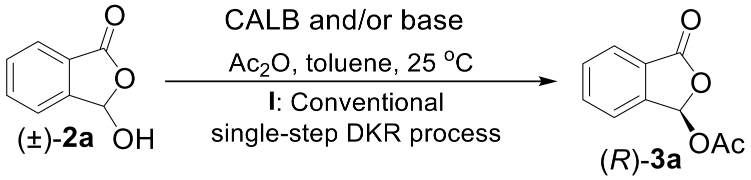

Entry	Base(equiv.)	Time (h)	%Yield of (*R*)-**3a**[b]	%*ee* of (*R*)-**3a**[b]
1	DBU (1)	36	68	3
2	K_2CO_3 (1)	36	58	45
3	$NaHCO_3$ (1)	36	55	46
4	*i*-Pr_2Et_3N (1)	36	56	29
5	Et_3N (1)	36	53	21
6	pyridine (1)	36	59	27
7	piperidine	36	61	13
8	DBU (0.2)	36	54	22
9	DBU (0.5)	36	59	13
10	DBU (1.5)	36	77	2
11	DBU (2.0)	36	83	0

[a] Reaction conditions. **2a** (0.20 mmol), base (0.2-2.0 equiv.), Novozym-435 (CALB) (40.0 mg, 20 mg/0.1 mmol), and acetic anhydride (0.40 mmol, 2.0 equiv.) were introduced into 4.0 mL of a mixture of toluene. [b] Yields were determined by ^{1}H–NMR analysis, and the %*ee* values were determined by chiral HPLC analysis.

Supplementary Table 2. Optimizing reaction conditions for the PDMS-modulated DKR of 2a.

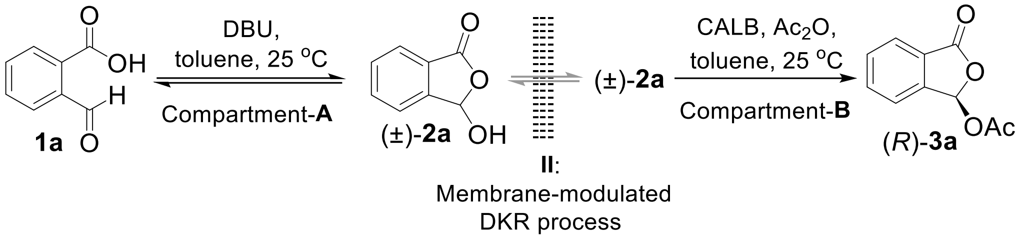

Entry	Base/(equiv.) in Compartment-**A**	CALB (mg) in Compartment-**B**	Time (h)	%Yield of (*R*)-**3a**[b]	%*ee* of (*R*)-**3a**[b]
1	DBU (2)	CALB (40)	48	82	99
2	Et_3N (2)	CALB (40)	48	76	99
3	Pyridine (2)	CALB (40)	48	79	99
4	$NaHCO_3$ (2)	CALB (40)	48	73	99
5	piperidine (2)	CALB (40)	48	81	99
6	DBU (1)	CALB (40)	36	91	99
7	DBU (1.5)	CALB (40)	36	88	99
8	DBU (1.2)	CALB (40)	36	90	99
9	DBU (0.8)	CALB (40)	36	89	99
10	DBU (1)	CALB (40)	38	91	99
11	DBU (1)	CALB (40)	34	87	99
12	DBU (1)	CALB (35)	36	88	99
13	DBU (1)	CALB (45)	36	91	99
14[c]	DBU (1)	CALB (40)	36	83	99
15[d]	DBU (1)	CALB (40)	36	87	99

[a] Reaction conditions. In compartment-**A**, **1a** (0.20 mmol), base (0.8-2.0 equiv.) was introduced into 2.0 mL of toluene. In compartment-**B**, the Novozym-435 (CALB) (35.0-45.0 mg, 20 mg/0.1 mmol) and acetic anhydride (0.40 mmol, 2.0 equiv.) were introduced into 2.0 mL of a mixture of toluene. [b] Yields were determined by ^{1}H–NMR analysis, and the %*ee* values were determined by chiral HPLC analysis. [c] Data were obtained by using a 0.20 mm thick PDMS membrane. [d] Data were obtained by using a 0.60 mm thick PDMS membrane.

Supplementary Table 3. Methods and crystal data, and structure refinement for (*R*)-3a (CCDC-2486834 for datablock(s) 20250910wj_auto).

Methods. The crystals of compound **3a** were grown using the solvent evaporation method with a mixed solvent consisting of a 1:10 mixture of dichloromethane and *n*-pentane. First, the purified compound **3a** (15 mg) was dissolved in a minimal volume (approximately 1 mL) of the solvent mixture at room temperature (20-25°C) in a clean glass vial. The resulting solution was then filtered through a 0.22 μm syringe filter. The filtered solution was transferred to a clean, sealed vial, and the vial was loosely capped to allow for slow evaporation. The vial was left undisturbed in a dark, stable environment at ambient temperature (20-25°C) for 2 days, during which the solvent gradually evaporated. The vial was monitored daily, and the crystals of **3a** were obtained after 2 days. The single-crystal X-ray diffraction data were collected on a Bruker D8 VENTURE Photon III diffractometer with a helios MX multilayer monochromator Cu Kα radiation (λ = 1.54178 Å) at 100 K in the Instrumental Analysis Center of Shanghai Jiao Tong University. Data collection, unit cell refinement, and data reduction were performed using APEX5 v2023.9-2. The structure was solved by the Intrinsic Phasing method and refined by full-matrix least-squares on F2 with anisotropic displacement parameters for the non-H atoms using the SHELXTL program package.

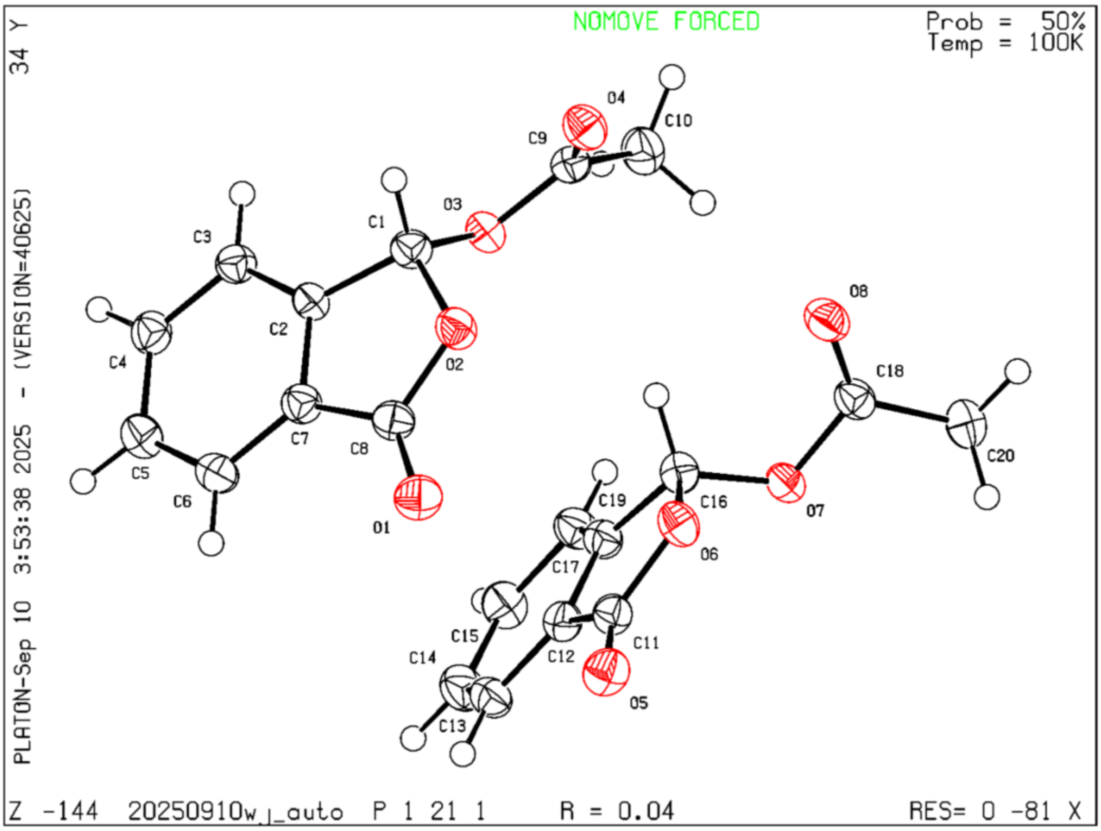

Crystal data and structure refinement for (*R*)-3a (CCDC-2486834. Datablock: 20250910wj_auto).

Identification code	CCDC-2486834
Empirical formula	$C_{10}H_8O_4$
Formula weight	192.16
Temperature/K	100.00(10)
Crystal system	monoclinic
Space group	$P2_1$
a/Å	8.4479(4)
b/Å	11.2745(6)
c/Å	9.5742(5)
α/°	90
β/°	90.338(4)
γ/°	90
Volume/Å^3	911.89(8)
Z	4
ρ_{calc}g/cm^3	1.400
μ/mm^{-1}	0.930
F(000)	400.0
Crystal size/mm^3	0.14 × 0.12 × 0.1
Radiation	Cu Kα (λ = 1.54184)
2θ range for data collection/°	9.238 to 151.374
Index ranges	$-10 \le h \le 10$, $-13 \le k \le 13$, $-11 \le l \le 11$
Reflections collected	9678
Independent reflections	3300 [R_{int} = 0.0395, R_{sigma} = 0.0441]
Data/restraints/parameters	3300/1/256
Goodness-of-fit on F^2	1.017
Final R indexes [I>=2σ (I)]	R_1 = 0.0410, wR_2 = 0.1052
Final R indexes [all data]	R_1 = 0.0483, wR_2 = 0.1103
Largest diff. peak/hole / e Å^{-3}	0.23/-0.21
Flack parameter	-0.1(2)

Supplementary Table 4. Fractional atomic coordinates (×10^4) and equivalent isotropic displacement parameters (Å^2×10^3) for (*R*)-3a (CCDC-2486834. Datablock: 20250910wj_auto. U_{eq} is defined as 1/3 of the trace of the orthogonalised U_{IJ} tensor).

Atom	*x*	*y*	*z*	U(eq)
C8	9895(4)	5329(3)	3051(3)	26.1(7)
O8	5662(3)	5759(2)	7505(2)	32.6(5)
C9	8857(4)	3540(3)	6778(3)	25.9(7)
C18	4431(4)	5840(3)	6892(3)	26.5(7)
C2	10566(3)	3355(3)	3305(3)	22.5(6)
O2	9935(3)	5112.5(19)	4466(2)	27.7(5)
C4	11056(4)	1976(3)	1488(3)	28.2(7)
O4	9713(3)	4194(2)	7407(2)	30.4(5)
C7	10306(4)	4232(3)	2317(3)	25.8(7)
O7	4322(3)	5779(2)	5461(2)	27.9(5)
C17	5511(4)	5137(3)	3345(3)	25.0(7)
C1	10328(4)	3886(3)	4732(3)	25.2(6)
O1	9565(3)	6303(2)	2610(2)	33.0(6)
C16	5792(4)	5707(3)	4746(3)	26.3(7)
C15	4759(4)	3740(3)	1622(4)	31.9(8)
C14	5118(4)	4561(3)	579(4)	33.3(8)
C13	5667(4)	5686(3)	914(3)	28.9(7)
C12	5850(4)	5957(3)	2322(3)	25.5(7)
C11	6310(4)	7082(3)	2996(3)	26.3(7)
C6	10415(4)	4006(3)	889(3)	29.9(7)
O6	6306(3)	6891(2)	4426(2)	28.6(5)
C5	10796(4)	2863(3)	489(3)	31.7(7)
O5	6607(3)	8040(2)	2522(2)	32.9(5)
C3	10945(4)	2208(3)	2910(3)	25.5(7)
O3	9011(3)	3327(2)	5368(2)	25.6(5)
C19	4950(4)	4010(3)	3035(3)	29.2(7)
C20	2824(4)	6029(3)	7507(3)	32.3(8)
C10	7457(4)	2900(3)	7346(3)	33.2(7)

Supplementary Table 5. Anisotropic displacement parameters (Å^2×10^3) for (*R*)-3a (CCDC-2486834.Datablock: 20250910wj_auto. The Anisotropic displacement factor exponent takes the form: $-2\pi^2[h^2a^{*2}U_{11}+2hka^*b^*U_{12}+\ldots]$).

Atom	U_{11}	U_{22}	U_{33}	U_{23}	U_{13}	U_{12}
C8	27.1(16)	25.2(16)	26.0(16)	1.8(13)	-0.7(12)	-1.7(12)
O8	32.7(12)	41.2(14)	23.9(11)	0.1(10)	-1.4(9)	-1.9(11)
C9	28.9(15)	24.7(16)	24.1(15)	1.7(12)	-0.8(12)	4.8(13)
C18	32.7(17)	23.9(16)	22.8(15)	-1.1(13)	0.6(13)	-1.1(14)
C2	24.4(14)	23.4(15)	19.7(14)	0.5(12)	1.4(11)	-0.6(12)
O2	35.5(12)	23.3(12)	24.4(11)	-0.9(9)	1.8(9)	1.5(9)
C4	28.2(16)	28.7(16)	27.6(16)	-4.4(13)	-0.3(13)	1.1(13)
O4	34.7(12)	29.4(13)	27.2(11)	-1.9(9)	1.1(9)	-3.1(10)
C7	26.9(15)	25.2(16)	25.3(15)	0.7(12)	0.3(12)	-0.1(13)
O7	27.7(11)	35.7(12)	20.2(10)	0.7(10)	0.9(8)	1.6(10)
C17	27.5(15)	24.3(16)	23.3(15)	1.0(12)	1.0(11)	1.2(13)
C1	29.3(15)	20.7(16)	25.6(15)	2.3(12)	0.2(12)	-0.6(12)
O1	37.9(14)	24.8(12)	36.3(13)	4.8(10)	1.5(10)	-0.1(10)
C16	26.7(15)	27.3(16)	24.9(16)	-1.4(13)	1.7(12)	0.3(13)
C15	38.3(18)	25.4(17)	32.0(18)	-1.7(13)	2.0(14)	-2.7(14)
C14	40(2)	32.9(18)	27.0(16)	-5.4(14)	-0.6(13)	0.2(15)
C13	33.3(17)	30.1(17)	23.5(15)	3.3(14)	2.3(12)	0.6(14)
C12	26.0(15)	25.0(17)	25.4(16)	-1.2(12)	0.5(12)	1.5(12)
C11	24.3(15)	29.0(17)	25.5(16)	-0.6(13)	0.7(12)	0.9(12)
C6	34.5(17)	31.7(18)	23.4(16)	1.7(14)	-0.7(12)	2.1(15)
O6	33.2(12)	28.6(12)	24.1(11)	-3.8(9)	1.9(9)	-3.9(10)
C5	34.4(17)	35.4(19)	25.4(16)	-2.8(14)	1.0(13)	1.2(15)
O5	34.9(13)	24.3(12)	39.5(13)	2.3(10)	2.7(10)	-0.8(10)
C3	27.0(15)	24.3(16)	25.2(16)	0.2(12)	-1.2(12)	0.6(12)
O3	28.6(11)	27.1(12)	21.2(10)	-0.2(9)	2.0(8)	-1.6(9)
C19	35.4(17)	26.5(17)	25.6(16)	1.7(14)	0.1(13)	-0.9(14)
C20	31.6(17)	35.1(19)	30.2(17)	-1.8(15)	6.3(13)	-2.4(14)
C10	33.6(18)	34.7(18)	31.5(17)	0.8(14)	5.3(14)	-1.1(14)

Supplementary Table 6. Bond lengths for (*R*)-3a (CCDC-2486834. Datablock: 20250910wj_auto).

Atom	Atom	Length/Å	Atom	Atom	Length/Å
C8	O2	1.377(4)	C7	C6	1.395(4)
C8	C7	1.465(4)	O7	C16	1.424(3)
C8	O1	1.209(4)	C17	C16	1.505(4)
O8	C18	1.194(4)	C17	C12	1.379(4)
C9	O4	1.193(4)	C17	C19	1.387(5)
C9	O3	1.378(4)	C1	O3	1.420(4)
C9	C10	1.491(5)	C16	O6	1.437(4)
C18	O7	1.375(3)	C15	C14	1.396(5)
C18	C20	1.498(4)	C15	C19	1.395(5)
C2	C7	1.385(4)	C14	C13	1.388(5)
C2	C1	1.506(4)	C13	C12	1.390(4)
C2	C3	1.385(4)	C12	C11	1.474(5)
O2	C1	1.444(4)	C11	O6	1.386(4)
C4	C5	1.400(5)	C11	O5	1.199(4)
C4	C3	1.391(4)	C6	C5	1.382(5)

Supplementary Table 7. Bond angles for (*R*)-3a (CCDC-2486834).

Atom	Atom	Atom	Angle/°	Atom	Atom	Atom	Angle/°
O2	C8	C7	108.5(3)	O2	C1	C2	104.7(2)
O1	C8	O2	120.6(3)	O3	C1	C2	108.8(3)
O1	C8	C7	130.9(3)	O3	C1	O2	108.7(3)
O4	C9	O3	122.8(3)	O7	C16	C17	108.6(3)
O4	C9	C10	126.5(3)	O7	C16	O6	108.3(3)
O3	C9	C10	110.7(3)	O6	C16	C17	104.7(3)
O8	C18	O7	122.6(3)	C19	C15	C14	121.7(3)
O8	C18	C20	127.3(3)	C13	C14	C15	120.9(3)
O7	C18	C20	110.1(3)	C14	C13	C12	117.4(3)
C7	C2	C1	108.3(3)	C17	C12	C13	121.3(3)
C3	C2	C7	121.1(3)	C17	C12	C11	108.8(3)
C3	C2	C1	130.6(3)	C13	C12	C11	129.9(3)
C8	O2	C1	110.3(2)	O6	C11	C12	107.3(3)
C3	C4	C5	121.6(3)	O5	C11	C12	131.6(3)
C2	C7	C8	108.2(3)	O5	C11	O6	121.1(3)
C2	C7	C6	121.8(3)	C5	C6	C7	117.3(3)
C6	C7	C8	129.9(3)	C11	O6	C16	110.9(2)
C18	O7	C16	115.4(2)	C6	C5	C4	120.8(3)
C12	C17	C16	108.3(3)	C2	C3	C4	117.3(3)
C12	C17	C19	122.3(3)	C9	O3	C1	115.0(2)
C19	C17	C16	129.3(3)	C17	C19	C15	116.4(3)

Supplementary Table 8. Torsion angles for (*R*)-3a (CCDC-2486834. Datablock: 20250910wj_auto).

A	B	C	D	Angle/°	A	B	C	D	Angle/°
C8	O2	C1	C2	1.1(3)	C16	C17	C12	C13	-178.7(3)
C8	O2	C1	O3	-115.0(3)	C16	C17	C12	C11	-1.7(3)
C8	C7	C6	C5	-178.5(3)	C16	C17	C19	C15	177.9(3)
O8	C18	O7	C16	-4.8(5)	C15	C14	C13	C12	0.3(5)
C18	O7	C16	C17	156.0(3)	C14	C15	C19	C17	0.1(5)
C18	O7	C16	O6	-90.8(3)	C14	C13	C12	C17	0.4(5)
C2	C7	C6	C5	0.0(5)	C14	C13	C12	C11	-175.9(3)
C2	C1	O3	C9	166.8(2)	C13	C12	C11	O6	179.4(3)
O2	C8	C7	C2	1.0(3)	C13	C12	C11	O5	1.7(6)
O2	C8	C7	C6	179.6(3)	C12	C17	C16	O7	115.6(3)
O2	C1	O3	C9	-79.8(3)	C12	C17	C16	O6	0.0(3)
O4	C9	O3	C1	3.8(4)	C12	C17	C19	C15	0.7(5)
C7	C8	O2	C1	-1.3(3)	C12	C11	O6	C16	-2.8(3)
C7	C2	C1	O2	-0.5(3)	C5	C4	C3	C2	0.0(5)
C7	C2	C1	O3	115.6(3)	O5	C11	O6	C16	175.3(3)
C7	C2	C3	C4	0.1(5)	C3	C2	C7	C8	178.7(3)
C7	C6	C5	C4	0.1(5)	C3	C2	C7	C6	-0.1(5)
O7	C16	O6	C11	-114.0(3)	C3	C2	C1	O2	-179.3(3)
C17	C16	O6	C11	1.7(3)	C3	C2	C1	O3	-63.2(4)
C17	C12	C11	O6	2.7(3)	C3	C4	C5	C6	-0.1(5)
C17	C12	C11	O5	-175.0(3)	C19	C17	C16	O7	-61.9(4)
C1	C2	C7	C8	-0.3(3)	C19	C17	C16	O6	-177.5(3)
C1	C2	C7	C6	-179.0(3)	C19	C17	C12	C13	-1.0(5)
C1	C2	C3	C4	178.7(3)	C19	C17	C12	C11	176.1(3)
O1	C8	O2	C1	179.0(3)	C19	C15	C14	C13	-0.6(6)
O1	C8	C7	C2	-179.3(3)	C20	C18	O7	C16	174.6(3)
O1	C8	C7	C6	-0.7(6)	C10	C9	O3	C1	-179.1(3)

Supplementary Table 9. Hydrogen atom coordinates (Å×10^4) and isotropic displacement parameters (Å^2×10^3) for (*R*)-3a (CCDC-2486834. Datablock: 20250910wj_auto).

**Atom**	***x***	***y***	***z***	**U(eq)**
H4	11313	1197	1186	34
H1	11304	3813	5322	30
H16	6604	5266	5305	32
H15	4376	2978	1364	38
H14	4984	4346	-374	40
H13	5907	6250	209	35
H6	10235	4612	218	36
H5	10882	2678	-476	38
H3	11123	1603	3585	31
H19	4709	3453	3747	35
H20A	2197	5303	7404	48
H20B	2293	6682	7018	48
H20C	2933	6225	8500	48
H10A	6541	3433	7354	50
H10B	7688	2635	8301	50
H10C	7221	2210	6758	50

Supplementary Figure 1. The permeation analyses of DBU and acetic anhydride.

For the GC analysis of DBU in the control experiment (before reaction at Compartment-**A** *versus* after reaction for 36 h at Compartment-**B**. Conditions. In compartment-**A**, DBU (0.20 mmol) in 2.0 mL of toluene, 25 °C, 36 h. In Compartment-**B**, 2.0 mL of toluene, 25 °C, 36 h.)

The calculated content ratio after reaction (the peak area of DBU after reaction for 36 h divided by the peak area of DBU before the reaction**)** = 5614366/116754976 = 4.8%).

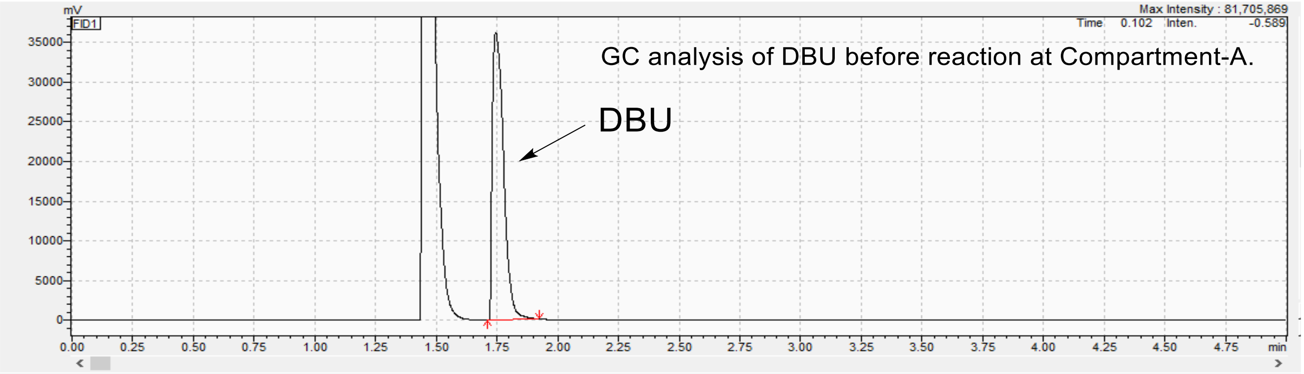

结果视图 － 峰表

峰表 化合物 组 校准曲线

峰号	保留时间	面积	高度	标记	浓度	浓度单位	化合物ID号	化合物名	面积%
1	1. 745	116754976	36120607	M	100. 000				100. 000
总计		116754976	36120607		100. 000				100. 000

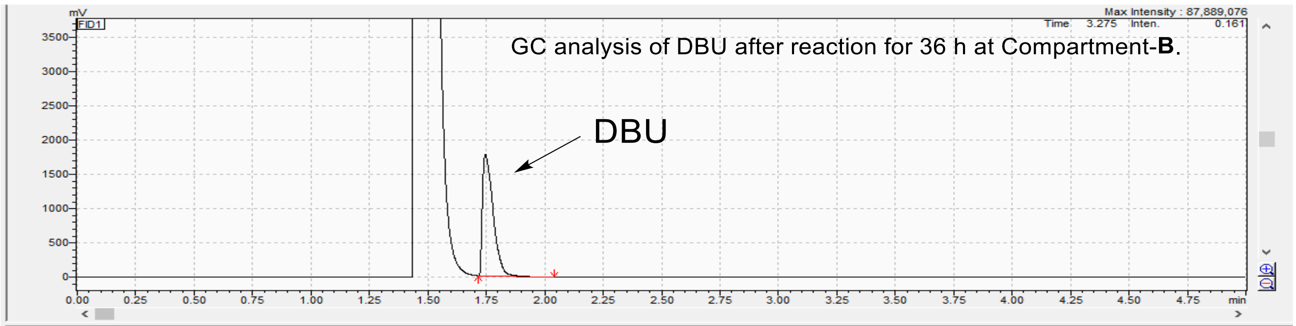

结果视图 － 峰表

峰表 化合物 组 校准曲线

峰号	保留时间	面积	高度	标记	浓度	浓度单位	化合物ID号	化合物名	面积%
1	1. 746	5614366	1756202	M	100. 000				100. 000
总计		5614366	1756202		100. 000				100. 000

For the GC/MS analysis of acetic anhydride in the control experiment (before reaction at Compartment-**B** *versus* the GC/MS analysis after reaction for 36 h at Compartment-**A**. Conditions. In compartment-**A**, 2.0 mL of toluene, 25 °C, 36 h. In compartment-**B**, Novozym-435 (CALB) (40.0 mg), acetic anhydride (0.40 mmol) in 2.0 mL of toluene, 25 °C, 36 h.)

The content ratio after reaction (the peak area of acetic anhydride after reaction for 36 h at Compartment-**A** divided by the peak area of acetic anhydride before the reaction at Compartment-**B)** = 24848/10039269 = 0.2%.)

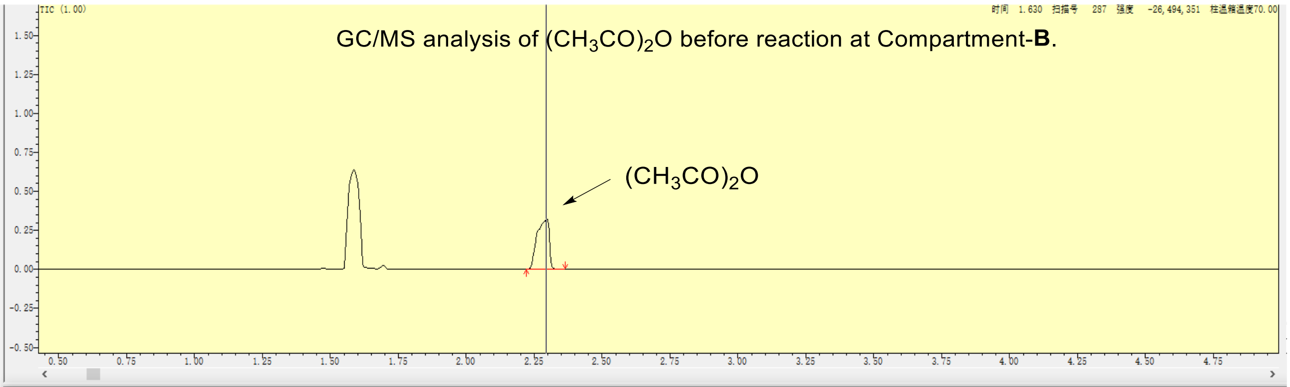

定性表

打印(P) 编辑(E) 视图(V) 相似度检索(S)

峰号	保留时间	开始时间	结束时间	m/z	峰面积	峰面积%	峰高	峰高%	A/H	标记	名称	CAS #	SI
1	2.296	2.220	2.365	TIC	10039269	100.00	31969043	100.00	3.14	MI		0-0-0	0

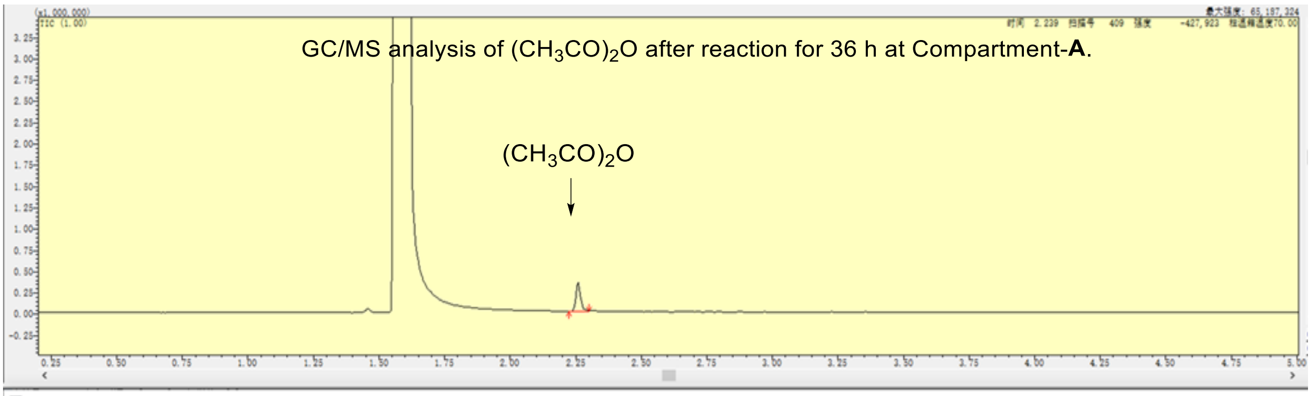

定性表

打印(P) 编辑(E) 视图(V) 相似度检索(S)

峰号	保留时间	开始时间	结束时间	m/z	峰面积	峰面积%	峰高	峰高%	A/H	标记	名称	CAS #	SI
1	2.259	2.225	2.300	TIC	24848	100.00	335138	100.00	1.27	MI		0-0-0	0

Supplementary Figure 2. Measurement of the diffusion coefficient of 2a through PDMS.

1.1 Measurement principle of the diffusion rate.[4-5]

The Time-Lag Method is a classical technique for determining the diffusion coefficient (D) by analyzing the transient diffusion data of a substance permeating through a thin film. The theoretical basis of this method lies in the fact that the diffusion of a substance through a film requires a transition period from initiation to the establishment of a steady state. This transition time is directly related to the diffusion coefficient and the film thickness. The formula for calculating the diffusion coefficient (D) is:

$D = L^2 / 6\theta$

Where:

- D is the diffusion coefficient (m^2/s).
- L is the film thickness (m).
- θ is the time-lag, which is the time required from the start of diffusion until the steady-state diffusion stage is reached (s).

1.2 Determination of the diffusion rate of the intermediate **2a**

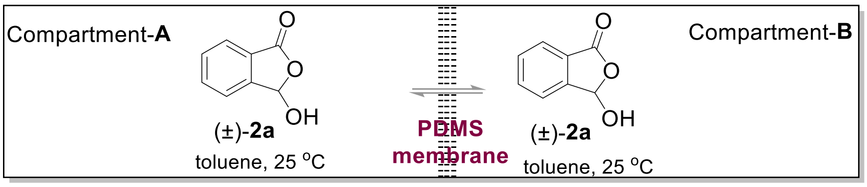

Objective: To quantify the diffusion coefficient (D) of the key intermediate compound **2a** through a defined PDMS, providing essential kinetic parameters for understanding its mass transport behavior.

Step 1: Conduct the diffusion experiment and collect data

The experimental setup referenced from the article is used. A PDMS membrane is securely fixed between the two compartments (Compartment-**A:** 2.0 mL of toluene containing **2a** (0.20 mmol, 30.0 mg), and Compartment-**B:** 2.0 mL of pure toluene). The experiment is conducted under constant temperature (25°C) with stirring at a fixed speed of 150 rpm to minimize boundary layer effects. At specific time intervals, a 10 μL sample is withdrawn from Compartment-**B** and analyzed by Gas Chromatography (GC/MS) to quantify the mass of **2a** that has diffused across the membrane.

The collected data were summarized in a table as shown below.

**Time (h)**	**Mass of 2a (mg) in Compartment-B**
0	0.00
20	0.00
40	0.00
60	0.00
70	0.0
75	0.1
80	0.43
100	0.81
120	1.34
140	1.78

160	2.33
180	2.93
200	3.41
220	3.94
240	4.50

Step 2: Plot the diffusion profile

Figure. Mass of **2a** diffused to the Compartment-**B** versus time.

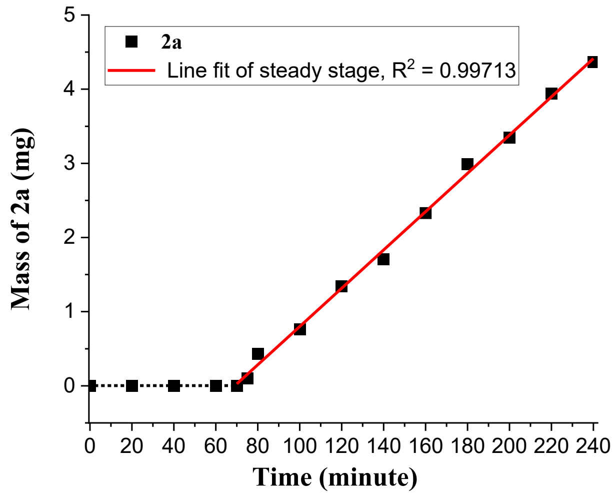

Step 3: Determine the time-lag (θ) from the Figure

The plotted curve typically shows two phases. (1) Initial Transient Phase: As the compound penetrates and diffuses through the membrane, the mass in the receptor compartment is very low. The curve is initially flat and close to the time axis. (2) Steady-State Phase**:** Diffusion reaches a steady state, and the mass in the receptor compartment increases linearly with time. The linear portion of the curve from the steady-state phase is extrapolated backwards until it intersects the time axis. The value of time at this intersection point is defined as the Time-Lag (θ) (defined as the time required from the start of diffusion until the steady-state diffusion stage is reached).

For the curve corresponding to compound **2a** (above Figure), the time-lag (θ) is estimated to be 70 minutes (1.17 h).

Step 4: Calculate the diffusion coefficient (D)

The obtained time-lag (θ) and the known membrane thickness (L) are substituted directly into the time-lag equation:

$D = L^2 / 6\theta$

Sample 2a Calculation:

- $L = 400\ \mu m = 400 \times 10^{-6}\ m = 4 \times 10^{-4}\ m$
- $\theta = 70\ (minute) \times 60 = 4200\ s$

$D = (4 \times 10^{-4}\ m)^2 / (6 \times 4200\ s) = (1.6 \times 10^{-7}\ m^2) / (25200\ s) \approx 6.35 \times 10^{-12}\ m^2/s$.

Supplementary Figure 3. Measurement of the DBU-racemization kinetic parameters.

1.1 Reaction modeling principle.[6-8]

The DBU-catalyzed racemization is modeled as a first-order irreversible reaction: (*S*) → Racemate. The kinetic analysis relies on monitoring the decrease in enantiomeric excess (*ee*) over time. Its kinetic parameters are solved using the following mathematical model:

(1) Calculation of enantiomeric excess (ee%):

The concentrations of the R- and S-enantiomers are determined separately using HPLC. The ee% is calculated using the formula:

$ee\% = (([R]-[S])/([R]+[S])) \times 100$

(2) Determination of rate constant (k_{rac}) and half-life ($t_{1/2rac}$):

A plot of ln(ee) versus time is constructed. The slope of this linear plot gives the racemization rate constant, k_{rac}. The half-life of racemization is then calculated from the rate constant using the equation:

$t_{1/2rac} = (\ln 2)/k_{rac}$

1.2 Determination of DBU-induced racemization kinetic parameters.

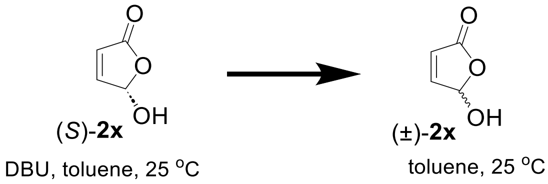

Objective: To quantitatively determine the kinetic parameters (rate constant k_{rac} and half-life $t_{1/2rac}$) of the DBU-catalyzed racemization using compound **2x** as a model substrate, establishing a standard protocol for evaluating chiral stability under basic conditions.

Step 1: Reaction system configuration and collect data

(1) Base: 1,8-Diazabicyclo[5.4.0]undec-7-ene (DBU)

(2) Chiral Substrate: Compound 2x (0.20 mmol, 30.0 mg)

(3) Solvent: 2.0 mL toluene

(4) Conditions: Constant temperature (25°C) with stirring at 150 rpm.

Step 2: Calculation of enantiomeric excess (ee%)

(1) Solution preparation: Precisely weigh and dissolve 0.20 mmol of compound 2x in 2.0 mL of toluene.

(2) Reaction initiation: Add an equimolar amount of the base DBU (1,8-diazabicyclo[5.4.0]undec-7-ene) to the solution to initiate racemization. Start the timer immediately and maintain a constant stirring speed.

(3) Sampling and analysis: At specific, predetermined time intervals, withdraw a 100 μL aliquot from the reaction mixture.

(4) HPLC analysis: Analyze each aliquot using High-Performance Liquid Chromatography (HPLC) with a chiral stationary phase to separate and quantify the R- and S-enantiomers of 2x.

(5) Calculation: For each time point, calculate the enantiomeric excess (ee%) using the formula based on the concentrations ([R] and [S]) determined by HPLC:

a) ee% = [([R] - [S]) / ([R] + [S])] × 100

The collected data were summarized in a table as shown below.

Time (h)	*%ee* of 2x
0.0	98
0.5	83
1.0	56
2.0	28
3.0	12
4.0	8

(*S*)-3x: (*S*)-5-hydroxyfuran-2(5H)-one (HPLC: Chiralpak OJ-H, detected at 210 nm, eluent: n-hexane/2-propanol = 95/5, flow rate = 1.0 mL/min, 25°C).

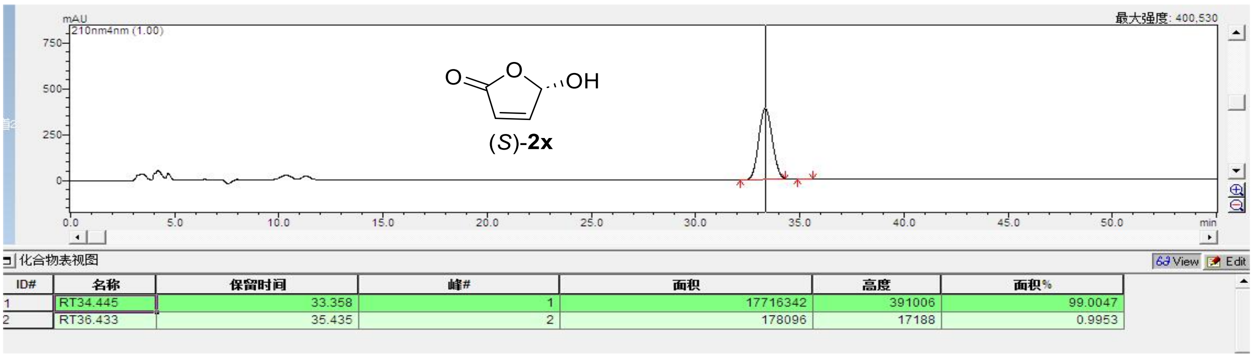

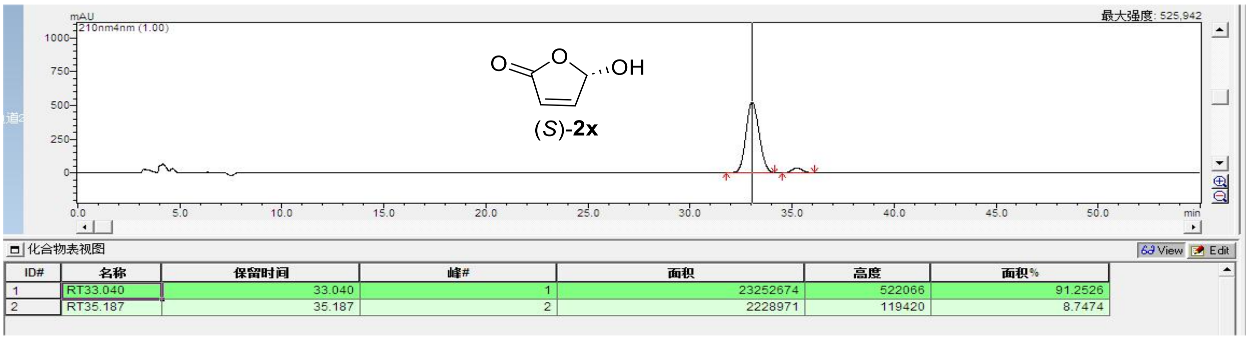

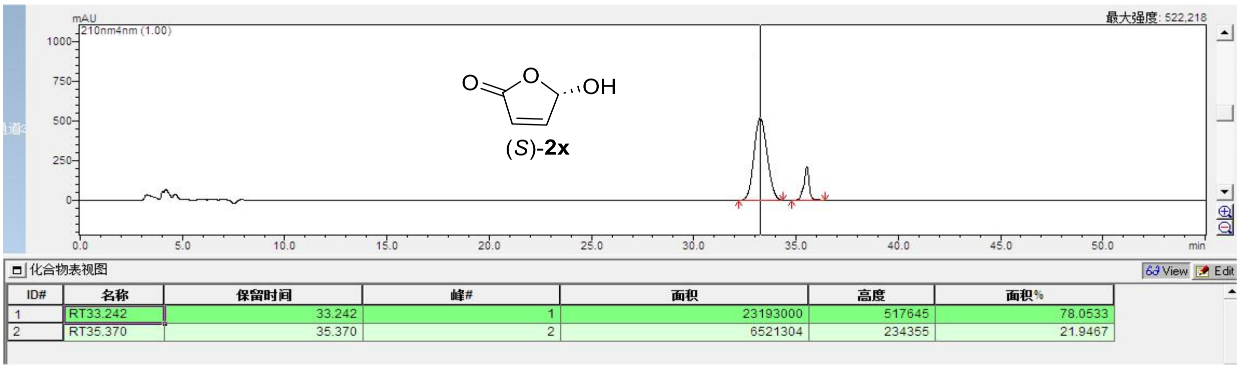

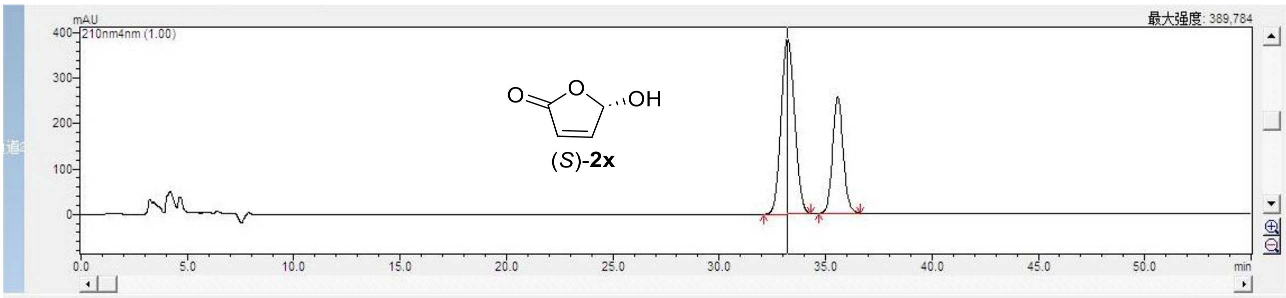

化合物表视图

ID#	名称	保留时间	峰#	面积	高度	面积%
1	RT33.209	33.209	1	17079772	386695	64.3601
2	RT35.357	35.357	2	9458038	264278	35.6399

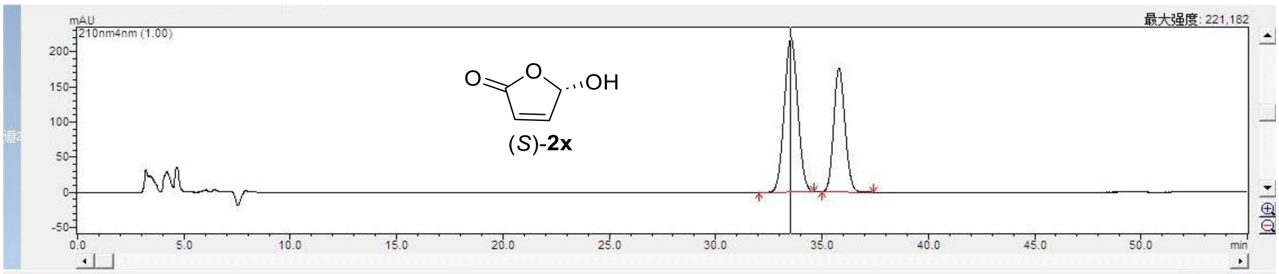

化合物表视图

ID#	名称	保留时间	峰#	面积	高度	面积%
1	RT33.533	33.533	1	9671812	219547	56.0636
2	RT35.671	35.671	2	7579683	181736	43.9364

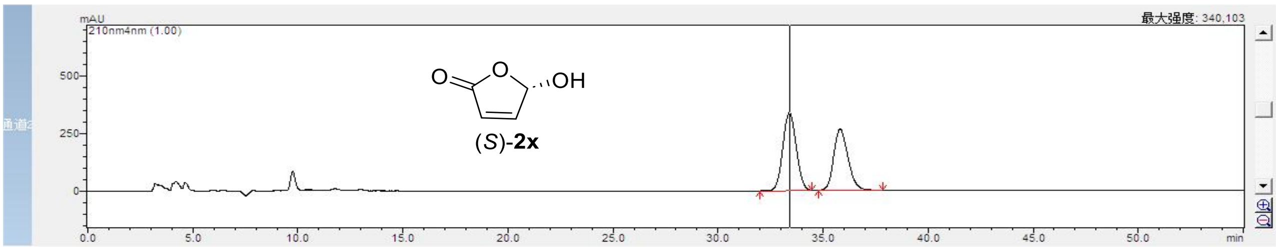

化合物表视图

ID#	名称	保留时间	峰#	面积	高度	面积%
1	RT33.389	33.389	1	14924001	336473	54.1481
2	RT35.523	35.523	2	12637466	289903	45.8519

<u>Translation of all characters (Chinese) in the above two frameworks to English is as follows:</u>

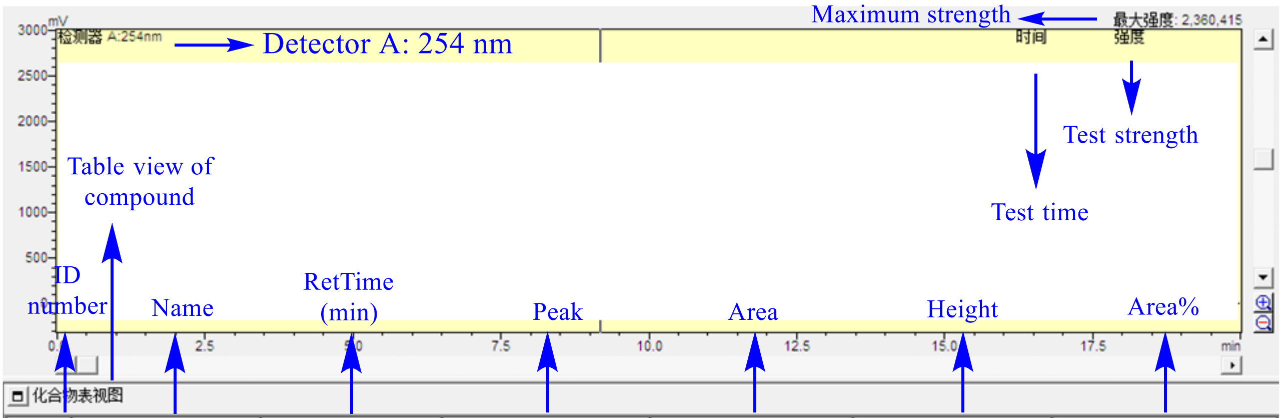

ID#	名称	保留时间	峰#	面积	高度	面积%
1	RT9.190	9.190	1	39625295	2360461	47.0509
2	RT11.859	11.859	2	44592689	2144002	52.9491

Step 3: Determination of the racemization rate constant (k_{rac})

(1) Data transformation: Calculate the natural logarithm (ln) of the ee% values obtained in Step 2 for each corresponding time point.

(2) Plot generation: Construct a graph of ln(ee) versus time.

(3) Linear regression: Perform a linear fit on the data points. The racemization is modeled as a first-order reaction, and the data should fit the equation:

$\ln(ee) = -k_{rac} \times t$

where t is time, and k_{rac} is a rate constant.

(4) Slope analysis: The slope of the resulting straight line is equal to the negative value of the racemization rate constant, $-k_{rac}$.

Figure. Influence of DBU on the rate constant of the racemization of **2x** at 25 °C, with time on the x-axis and ln(ee) on the y-axis.

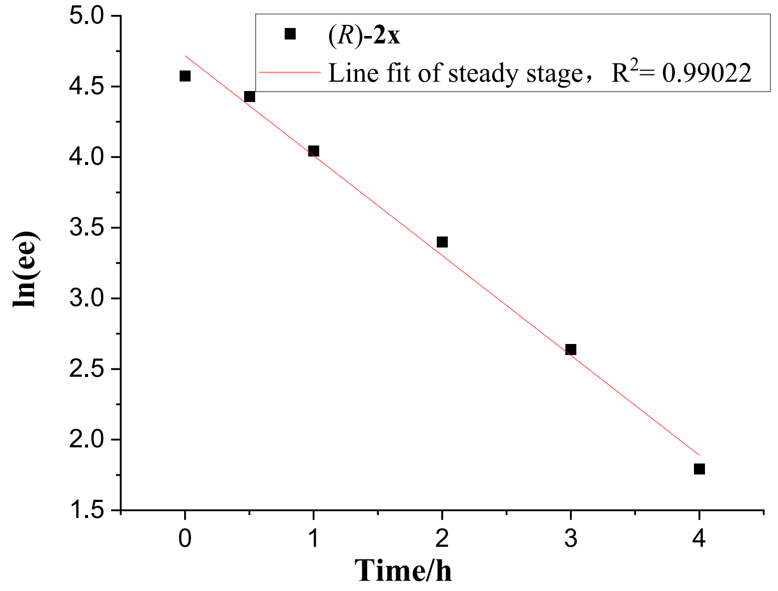

For the rate constant corresponding to compound **2x** (above Figure), the $\boldsymbol{k_{rac}}$ is estimated to be 0.70703 (h^{-1}).

Step 4: Calculation of the half-life ($t_{1/2rac}$)

(1) Parameter application: Once the racemization rate constant (k_{rac}) is determined from the slope in Step 3, it is used in the standard first-order kinetics half-life formula.

(2) Half-Life calculation: Calculate the half-life of racemization, which is the time required for the enantiomeric excess (ee%) to decrease to 50% of its initial value, using the equation:

$t_{1/2rac} = \ln 2/k_{rac}$

Sample calculation:

- $k_{rac} = 0.70703\ h^{-1}$

$t_{1/2\ rac} = \ln 2/k_{rac} = 0.693147/0.70703\ h^{-1} \approx 0.980\ h$

HPLC analyses of chiral products ((*R*)-3a-(*R*)-3t).

(*R*)-3a: (*R*)-3-oxo-1,3-dihydroisobenzofuran-1-yl acetate (HPLC: Chiralpak IC, detected at 210 nm, eluent: n-hexane/2-propanol = 90/10, flow rate = 1.0 mL/min, 25°C).

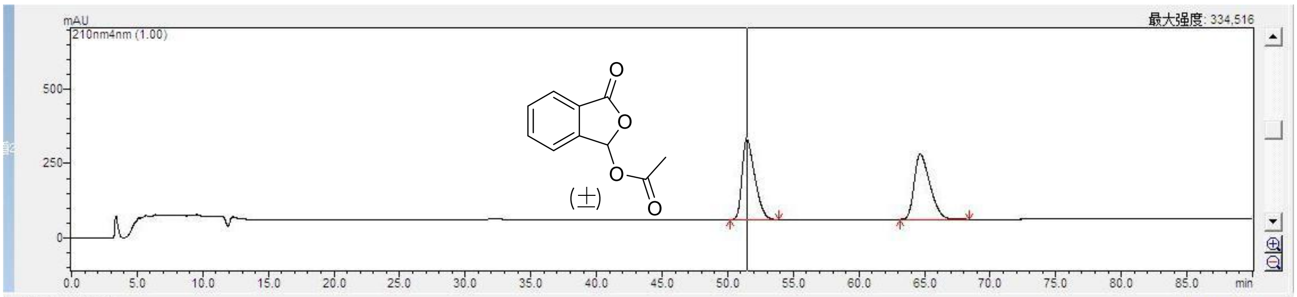

化合物表视图

ID#	名称	保留时间	峰#	面积	高度	面积%
1	RT51.446	51.446	1	18177709	272592	49.8495
2	RT64.668	64.668	2	18287499	220029	50.1505

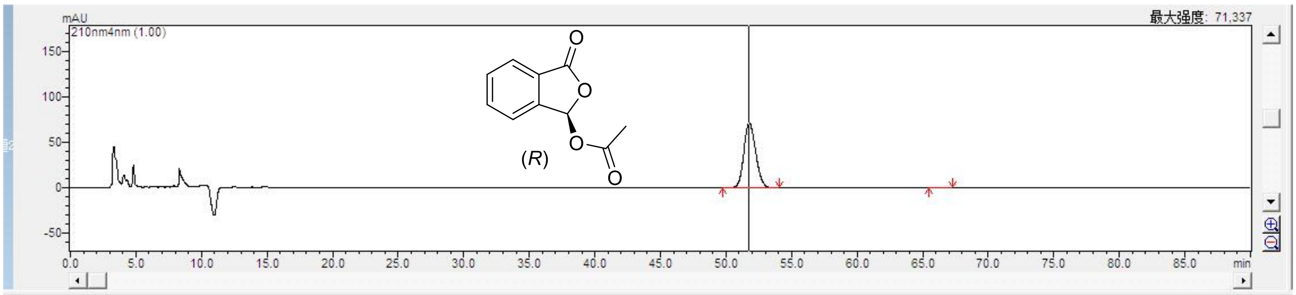

化合物表视图

ID#	名称	保留时间	峰#	面积	高度	面积%
1	RT51.762	51.762	1	4483681	71691	99.4712
2	RT66.770	66.770	2	23835	365	0.5288

Translation of all characters (Chinese) in the above two frameworks to English is as follows:

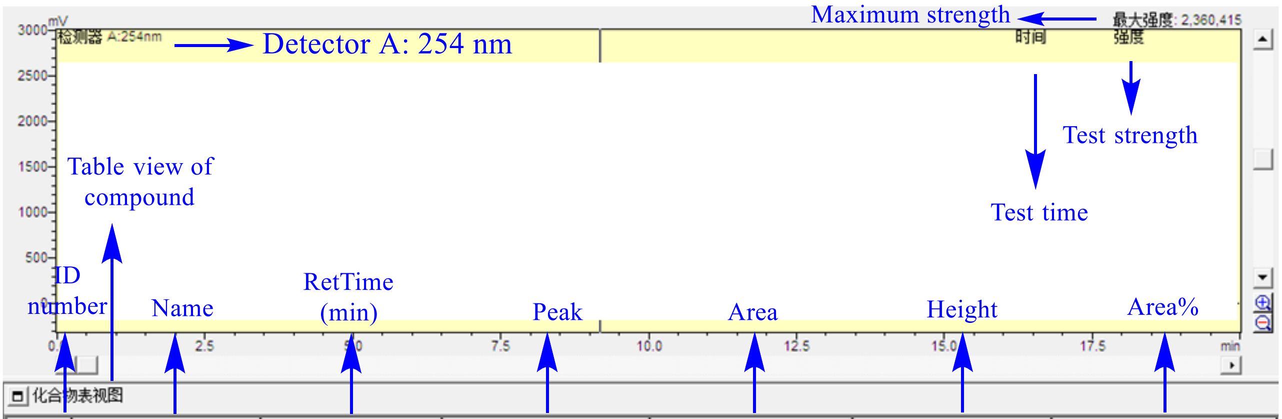

ID#	名称	保留时间	峰#	面积	高度	面积%
1	RT9.190	9.190	1	39625295	2360461	47.0509
2	RT11.859	11.859	2	44592689	2144002	52.9491

(*R*)-3b: (*R*)-4-fluoro-3-oxo-1,3-dihydroisobenzofuran-1-yl acetate (HPLC: Chiralpak OD, detected at 210 nm, eluent: n-hexane/2-propanol = 90/10, flow rate = 1.0 mL/min, 25°C).

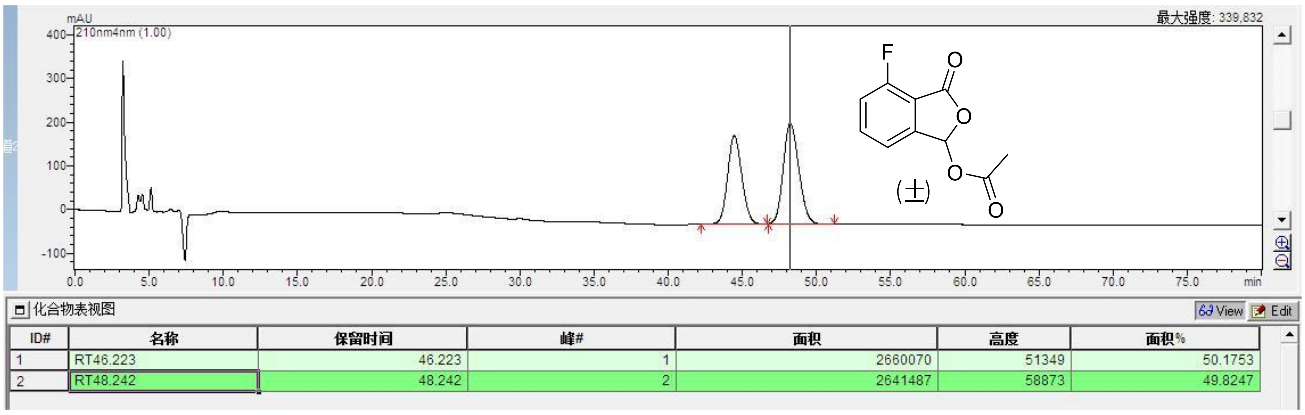

ID#	名称	保留时间	峰#	面积	高度	面积%
1	RT46.223	46.223	1	2660070	51349	50.1753
2	RT48.242	48.242	2	2641487	58873	49.8247

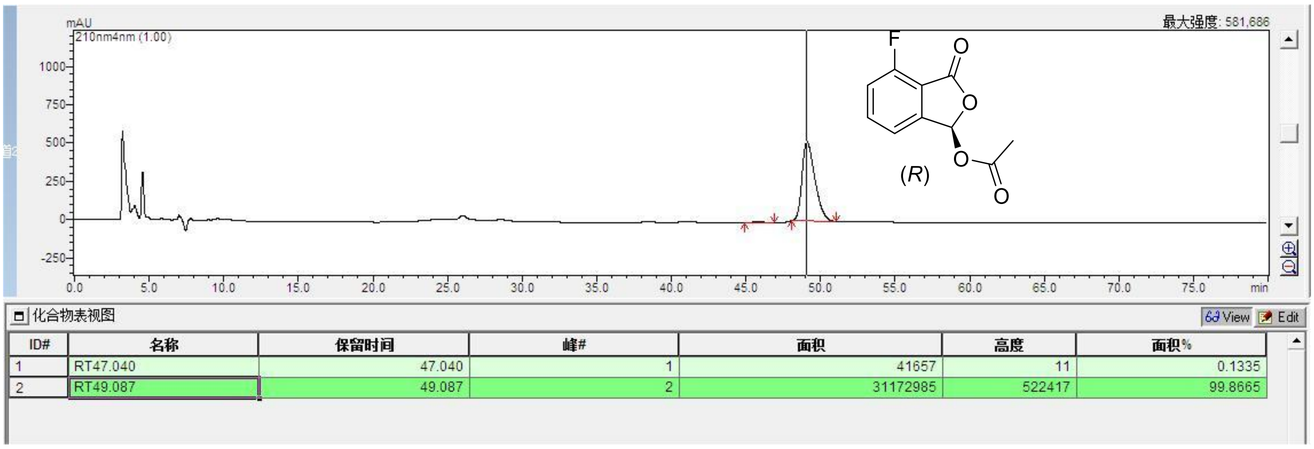

ID#	名称	保留时间	峰#	面积	高度	面积%
1	RT47.040	47.040	1	41657	11	0.1335
2	RT49.087	49.087	2	31172985	522417	99.8665

<u>**Translation of all characters (Chinese) in the above two frameworks to English is as follows:**</u>

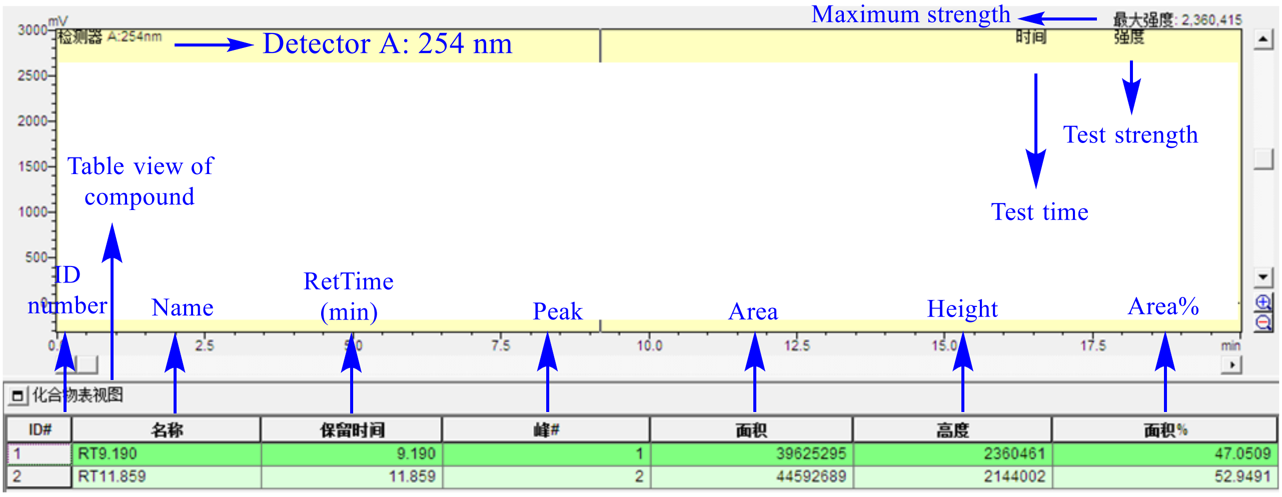

ID#	名称	保留时间	峰#	面积	高度	面积%
1	RT9.190	9.190	1	39625295	2360461	47.0509
2	RT11.859	11.859	2	44592689	2144002	52.9491

(*R*)-3c: (*R*)-5-fluoro-3-oxo-1,3-dihydroisobenzofuran-1-yl acetate (HPLC: Chiralpak IC, detected at 210 nm, eluent: n-hexane/2-propanol = 80/20, flow rate = 1.0 mL/min, 25°C).

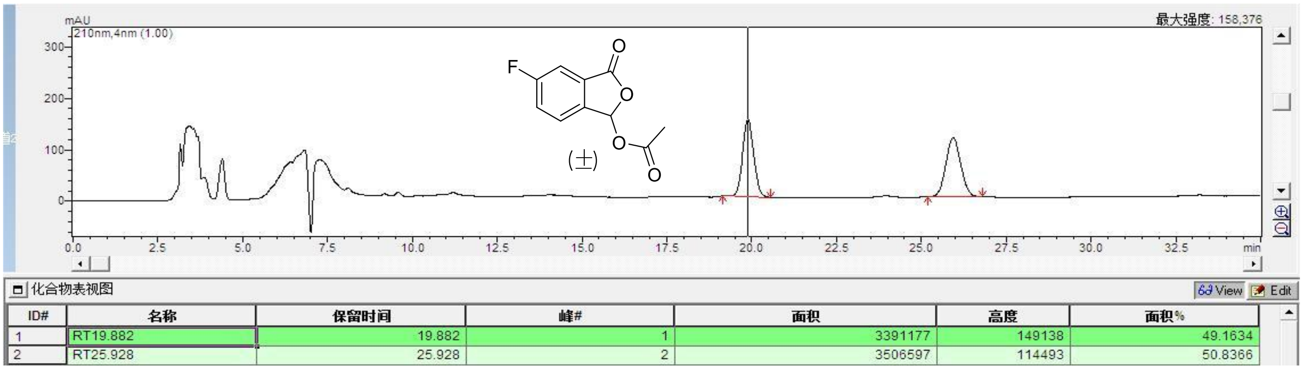

ID#	名称	保留时间	峰#	面积	高度	面积%
1	RT19.882	19.882	1	3391177	149138	49.1634
2	RT25.928	25.928	2	3506597	114493	50.8366

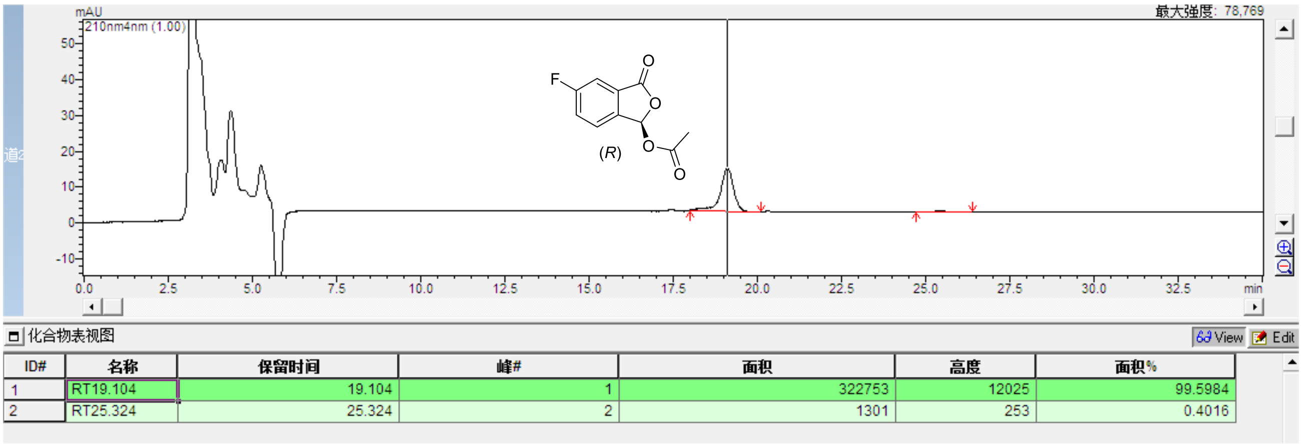

ID#	名称	保留时间	峰#	面积	高度	面积%
1	RT19.104	19.104	1	322753	12025	99.5984
2	RT25.324	25.324	2	1301	253	0.4016

__**Translation of all characters (Chinese) in the above two frameworks to English is as follows:**__

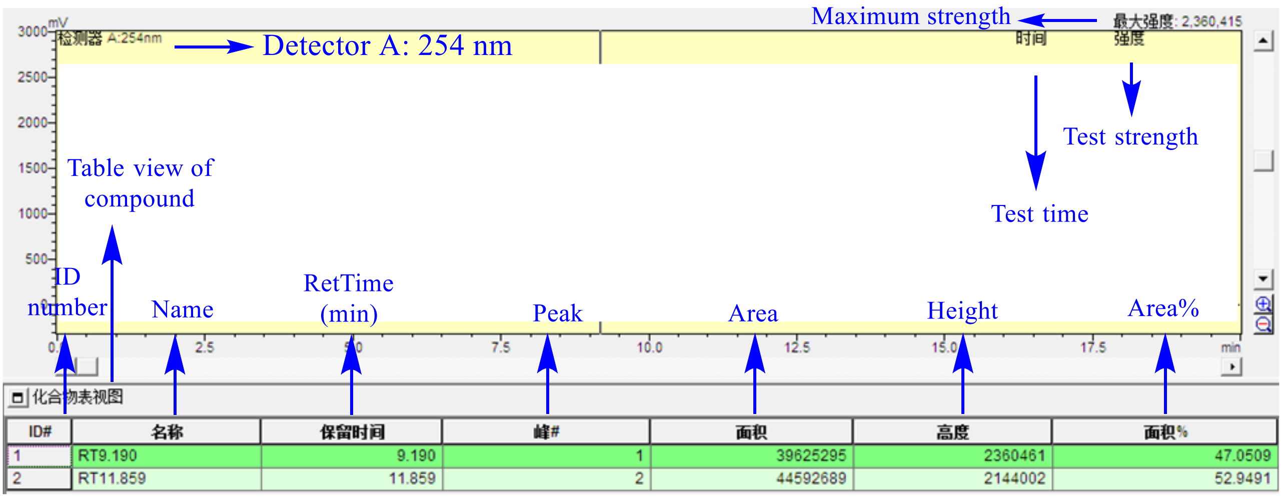

ID#	名称	保留时间	峰#	面积	高度	面积%
1	RT9.190	9.190	1	39625295	2360461	47.0509
2	RT11.859	11.859	2	44592689	2144002	52.9491

(*R*)-3d: (*R*)-6-fluoro-3-oxo-1,3-dihydroisobenzofuran-1-yl acetate (HPLC: Chiralpak IC, detected at 210 nm, eluent: n-hexane/2-propanol = 80/20, flow rate = 1.0 mL/min, 25°C).

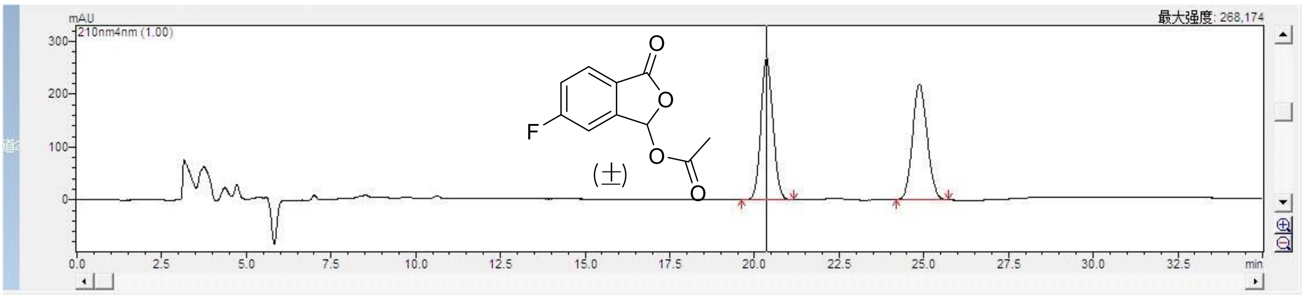

化合物表视图 View Edit

ID#	名称	保留时间	峰#	面积	高度	面积%
1	RT20.352	20.352	1	6662352	267945	50.1703
2	RT24.863	24.863	2	6617125	219275	49.8297

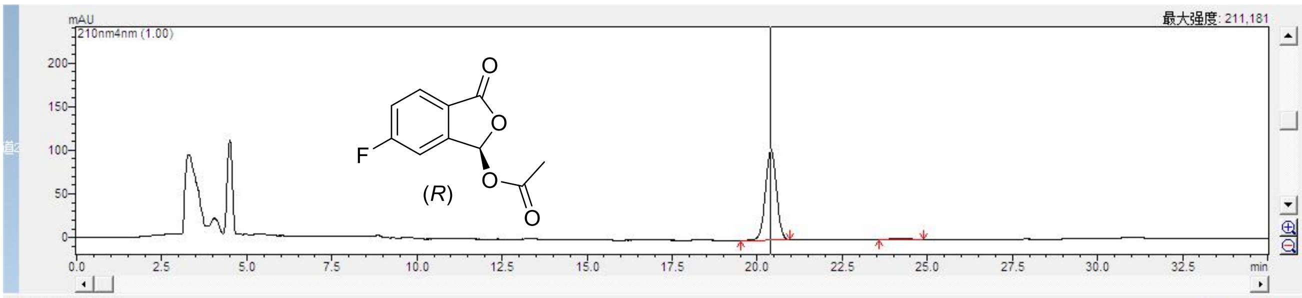

化合物表视图 View Edit

ID#	名称	保留时间	峰#	面积	高度	面积%
1	RT20.399	20.399	1	2343499	104178	98.8198
2	RT24.106	24.106	2	27988	2364	1.1802

<u>Translation of all characters (Chinese) in the above two frameworks to English is as follows:</u>

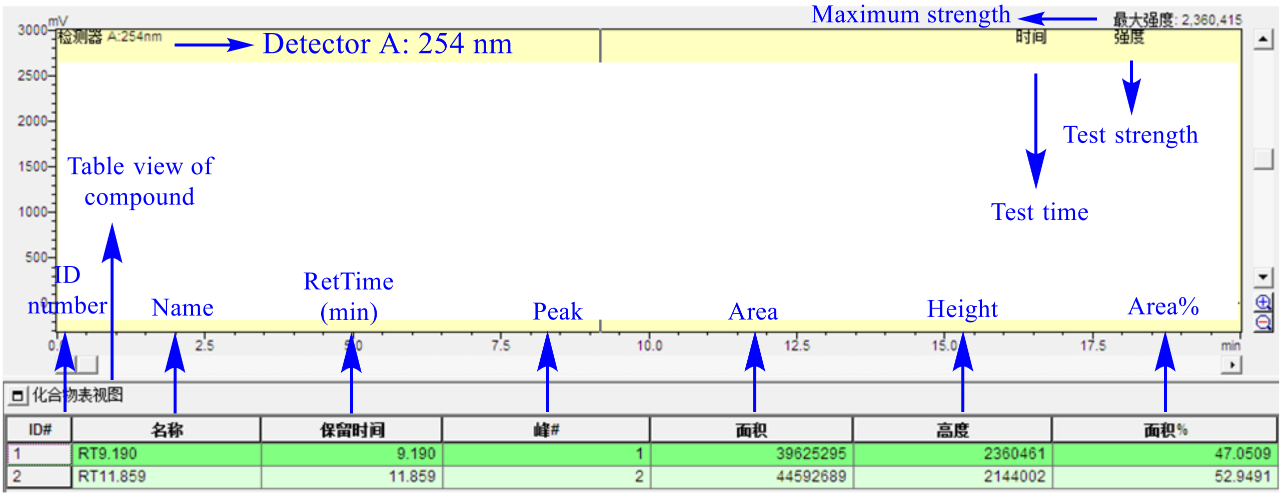

ID#	名称	保留时间	峰#	面积	高度	面积%
1	RT9.190	9.190	1	39625295	2360461	47.0509
2	RT11.859	11.859	2	44592689	2144002	52.9491

(*R*)-3e: (*R*)-5-chloro-3-oxo-1,3-dihydroisobenzofuran-1-yl acetate (HPLC: Chiralpak IC, detected at 254 nm, eluent: n-hexane/2-propanol = 80/20, flow rate = 1.0 mL/min, 25°C).

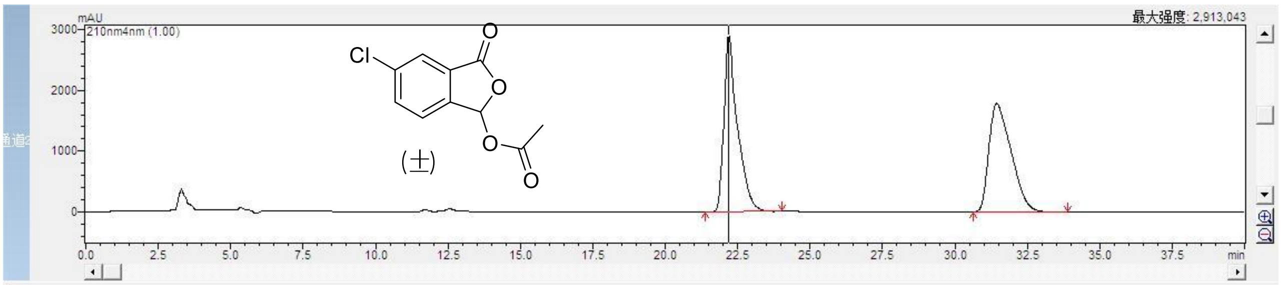

化合物表视图

ID#	名称	保留时间	峰#	面积	高度	面积%
1	RT22.184	22.184	1	93839046	2914316	49.4973
2	RT31.449	31.449	2	95745235	1792948	50.5027

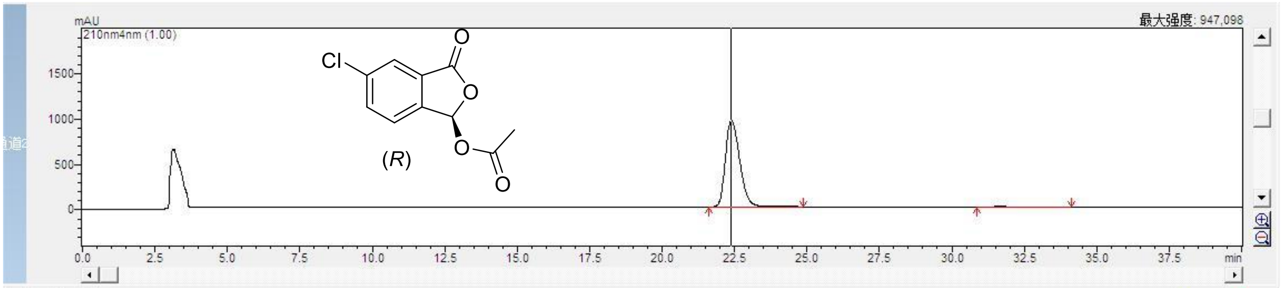

化合物表视图

ID#	名称	保留时间	峰#	面积	高度	面积%
1	RT22.390	22.390	1	32757886	950509	99.3534
2	RT31.755	31.755	2	213195	24420	0.6466

<u>**Translation of all characters (Chinese) in the above two frameworks to English is as follows:**</u>

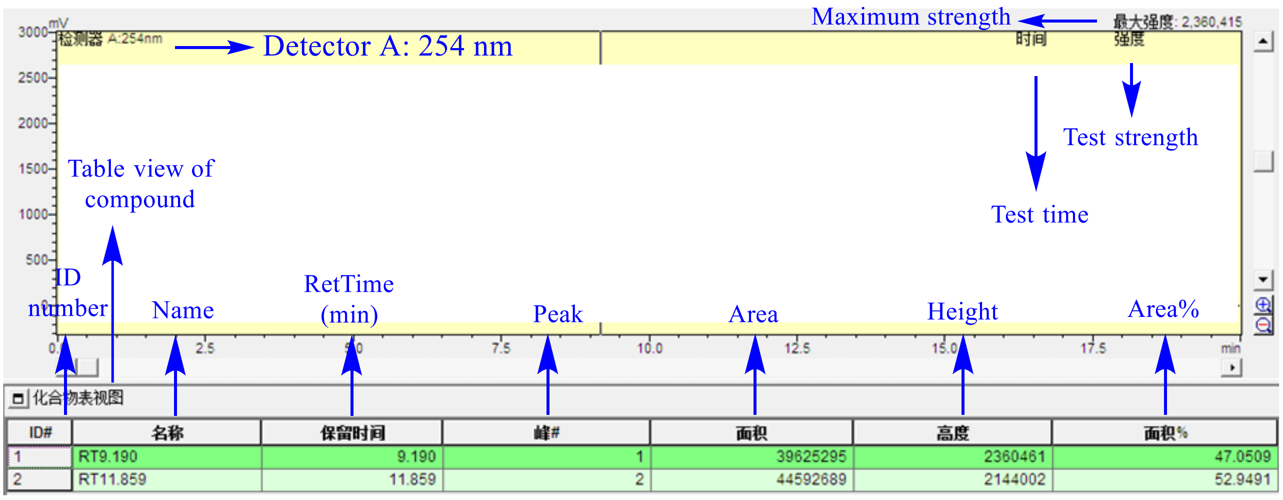

化合物表视图

ID#	名称	保留时间	峰#	面积	高度	面积%
1	RT9.190	9.190	1	39625295	2360461	47.0509
2	RT11.859	11.859	2	44592689	2144002	52.9491

(*R*)-3f: (*R*)-6-chloro-3-oxo-1,3-dihydroisobenzofuran-1-yl acetate (HPLC: Chiralpak IC, detected at 254 nm, eluent: n-hexane/2-propanol = 80/20, flow rate = 1.0 mL/min, 25°C).

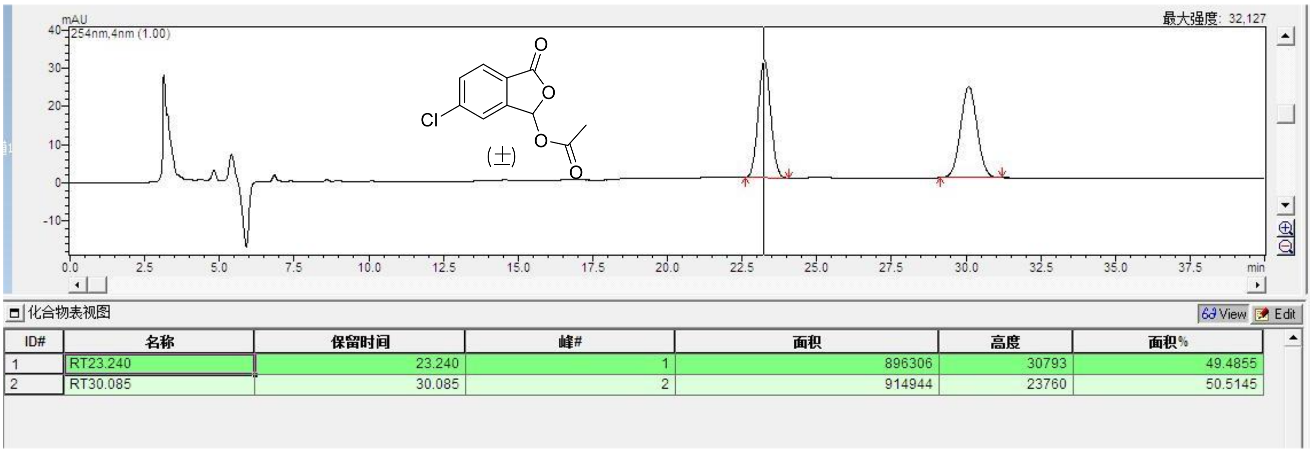

ID#	名称	保留时间	峰#	面积	高度	面积%
1	RT23.240	23.240	1	896306	30793	49.4855
2	RT30.085	30.085	2	914944	23760	50.5145

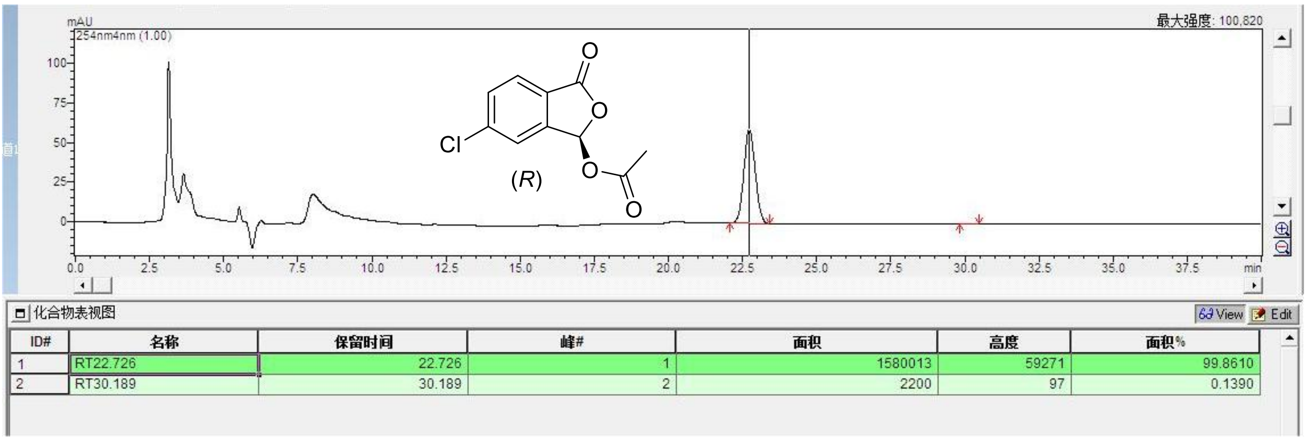

ID#	名称	保留时间	峰#	面积	高度	面积%
1	RT22.726	22.726	1	1580013	59271	99.8610
2	RT30.189	30.189	2	2200	97	0.1390

<u>Translation of all characters (Chinese) in the above two frameworks to English is as follows:</u>

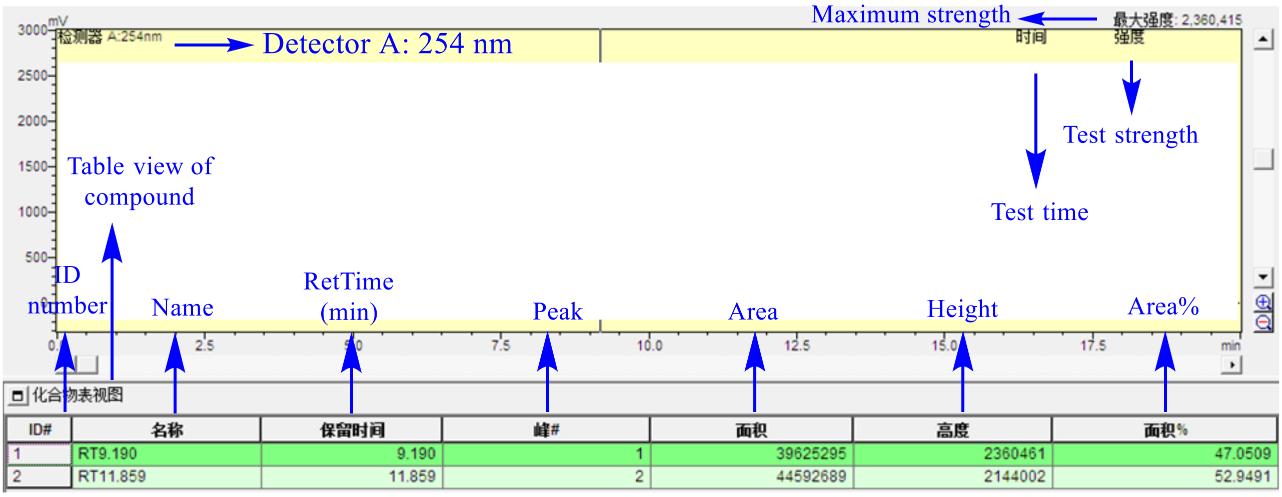

ID#	名称	保留时间	峰#	面积	高度	面积%
1	RT9.190	9.190	1	39625295	2360461	47.0509
2	RT11.859	11.859	2	44592689	2144002	52.9491

(*R*)-3g: (*R*)-7-chloro-3-oxo-1,3-dihydroisobenzofuran-1-yl acetate (HPLC: Chiralpak IC, detected at 210 nm, eluent: n-hexane/2-propanol = 80/20, flow rate = 1.0 mL/min, 25°C).

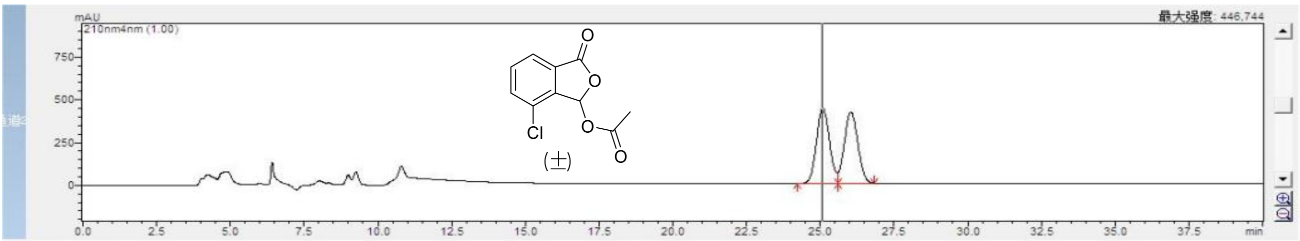

化合物表视图

ID#	名称	保留时间	峰#	面积	高度	面积%
1	RT25.084	25.084	1	13608276	440890	50.4488
2	RT26.038	26.038	2	13366156	418315	49.5512

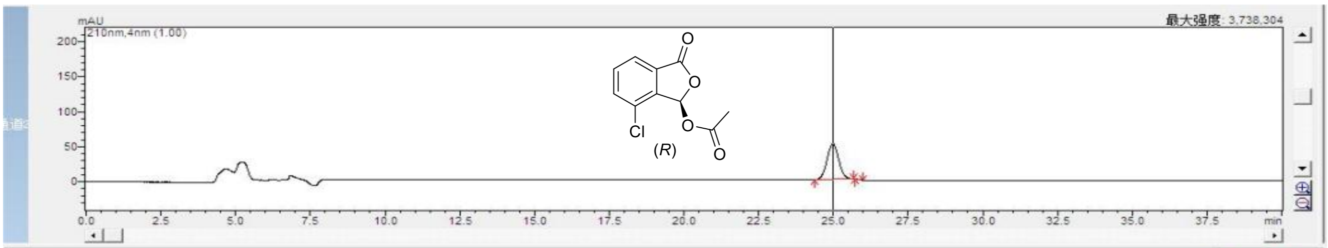

化合物表视图

ID#	名称	保留时间	峰#	面积	高度	面积%
1	RT24.978	24.978	1	1368732	50544	99.9757
2	RT25.717	25.717	2	332	12	0.0243

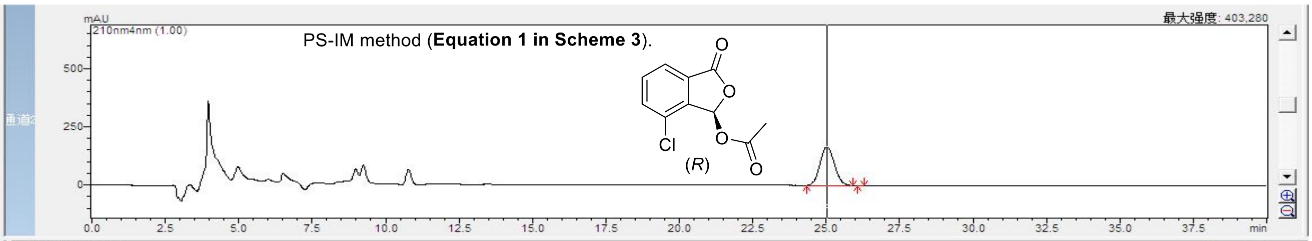

化合物表视图

ID#	名称	保留时间	峰#	面积	高度	面积%
1	RT25.013	25.013	1	9486358	356786	99.7550
2	RT25.957	25.957	2	23294	6658	0.2450

<u>Translation of all characters (Chinese) in the above two frameworks to English is as follows:</u>

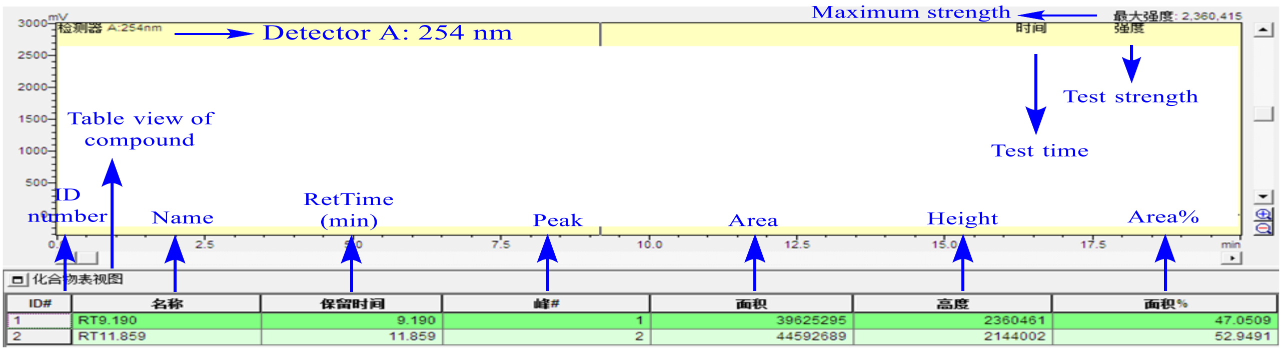

(*R*)-3h: (*R*)-4-bromo-3-oxo-1,3-dihydroisobenzofuran-1-yl acetate (HPLC: Chiralpak AD-H, detected at 210 nm, eluent: n-hexane/2-propanol = 90/10, flow rate = 1.0 mL/min, 25°C).

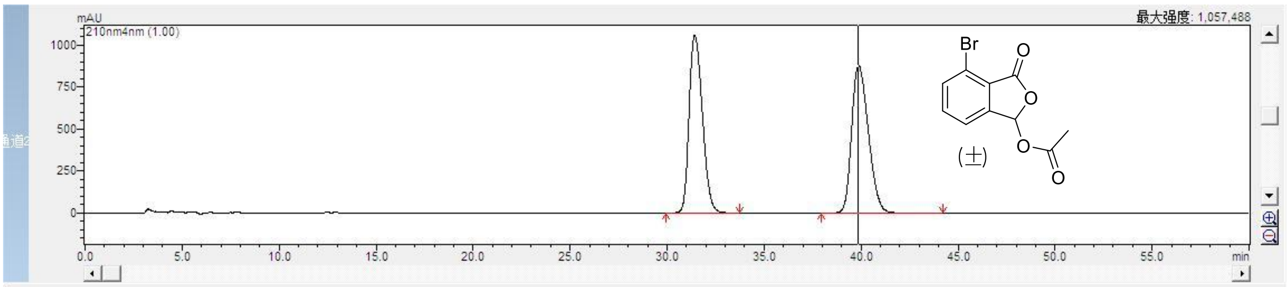

化合物表视图

ID#	名称	保留时间	峰#	面积	高度	面积%
1	RT31.422	31.422	1	50367960	1057871	48.9299
2	RT39.870	39.870	2	52570962	878427	51.0701

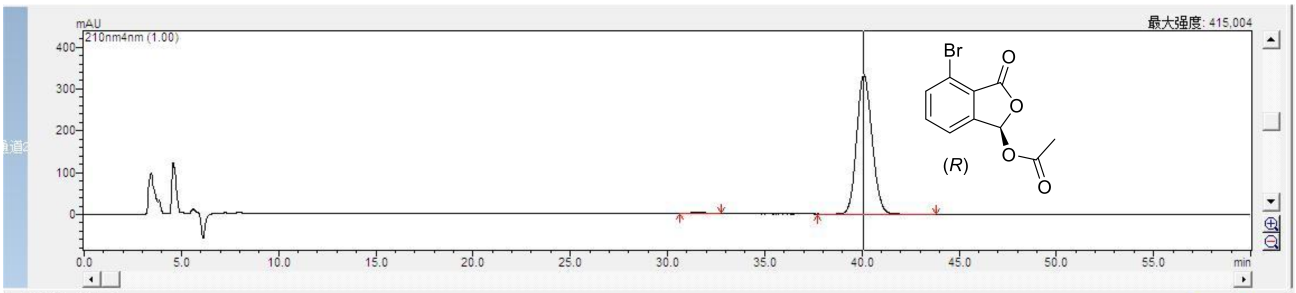

化合物表视图

ID#	名称	保留时间	峰#	面积	高度	面积%
1	RT31.625	31.625	1	61045	10888	0.3327
2	RT40.098	40.098	2	18284970	333228	99.6673

<u>Translation of all characters (Chinese) in the above two frameworks to English is as follows:</u>

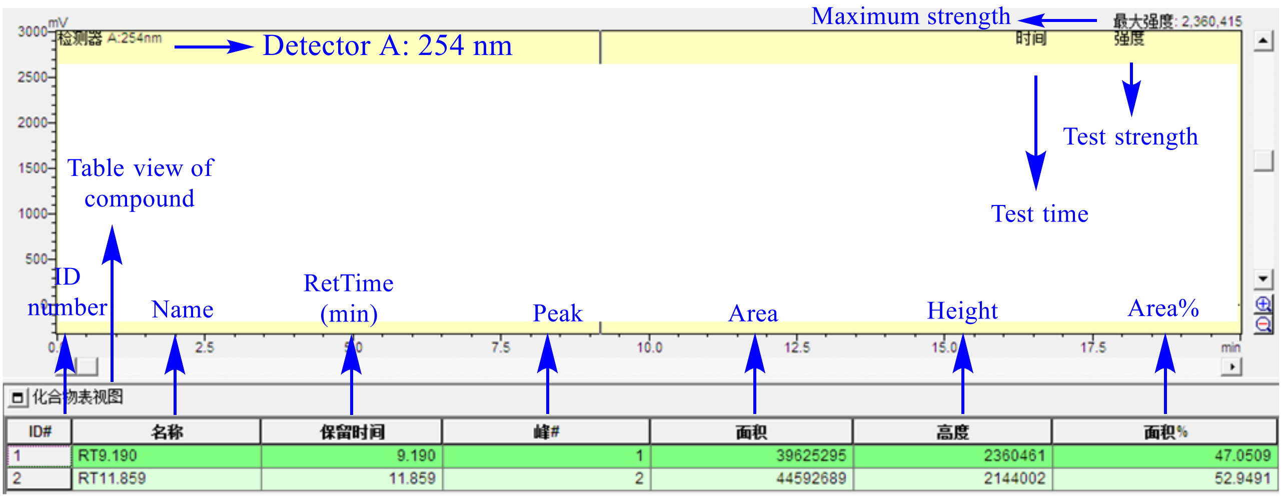

(*R*)-3i: (*R*)-5-bromo-3-oxo-1,3-dihydroisobenzofuran-1-yl acetate (HPLC: Chiralpak IC, detected at 210 nm, eluent: n-hexane/2-propanol = 80/20, flow rate = 1.0 mL/min, 25°C).

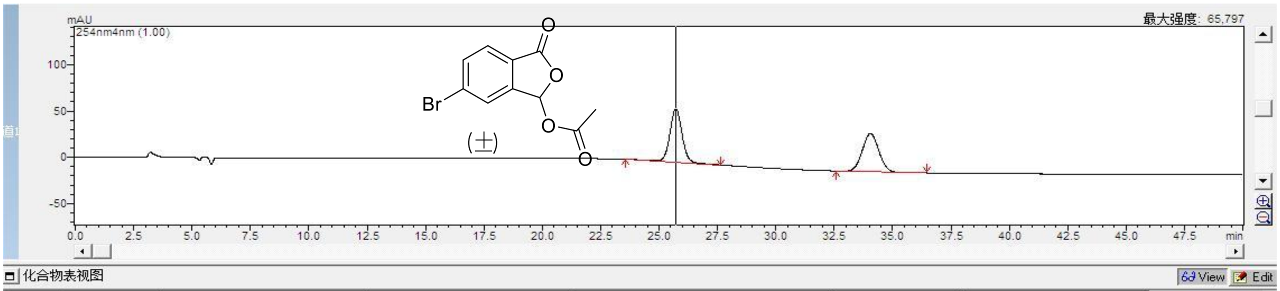

ID#	名称	保留时间	峰#	面积	高度	面积%
1	RT25.710	25.710	1	2103762	57804	51.0145
2	RT34.045	34.045	2	2020090	41099	48.9855

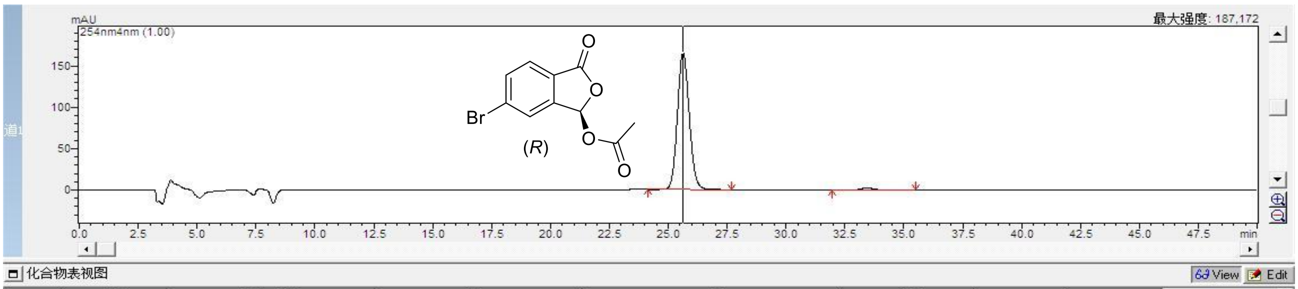

ID#	名称	保留时间	峰#	面积	高度	面积%
1	RT25.624	25.624	1	5792132	166085	98.9649
2	RT33.344	33.344	2	60579	5956	1.0351

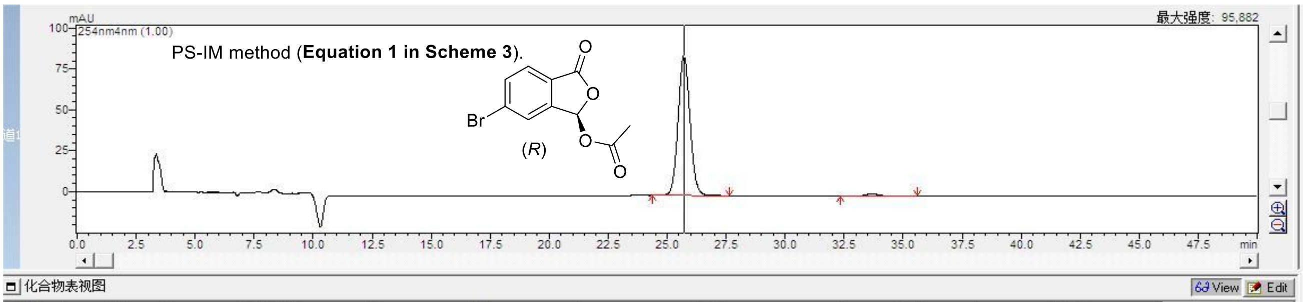

ID#	名称	保留时间	峰#	面积	高度	面积%
1	RT25.703	25.703	1	2987290	85394	98.3092
2	RT33.471	33.471	2	51377	5193	1.6908

<u>Translation of all characters (Chinese) in the above two frameworks to English is as follows:</u>

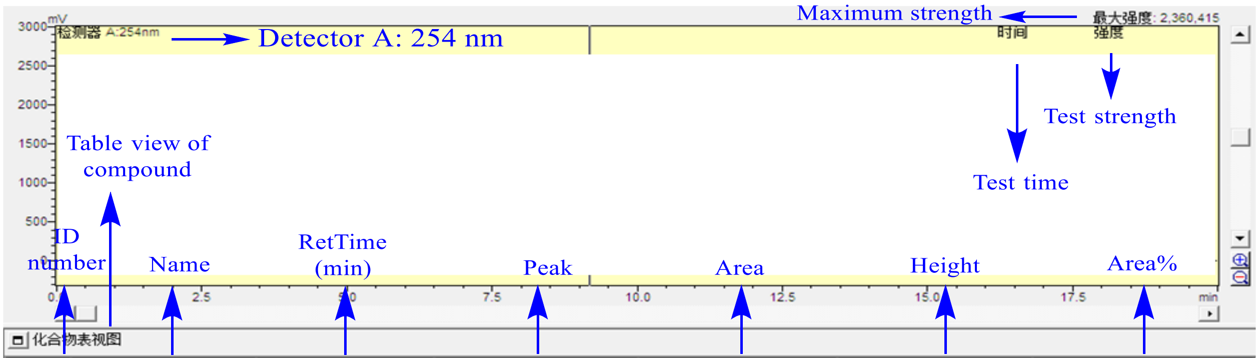

ID#	名称	保留时间	峰#	面积	高度	面积%
1	RT9.190	9.190	1	39625295	2360461	47.0509
2	RT11.859	11.859	2	44592689	2144002	52.9491

(*R*)-3j: (*R*)-5-bromo-3-oxo-1,3-dihydroisobenzofuran-1-yl acetate (HPLC: Chiralpak IC, detected at 210 nm, eluent: n-hexane/2-propanol = 80/20, flow rate = 1.0 mL/min, 25°C).

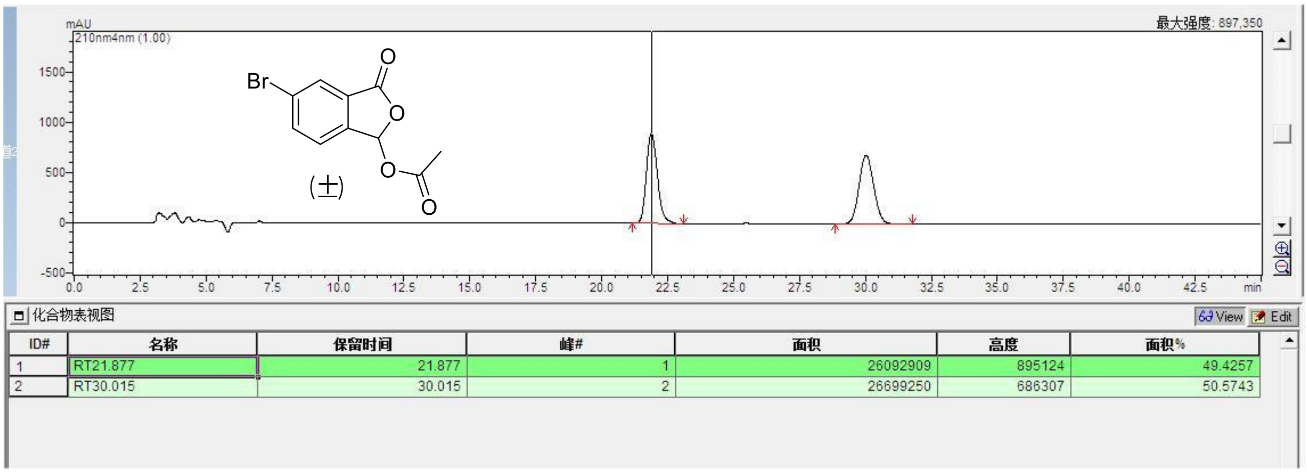

ID#	名称	保留时间	峰#	面积	高度	面积%
1	RT21.877	21.877	1	26092909	895124	49.4257
2	RT30.015	30.015	2	26699250	686307	50.5743

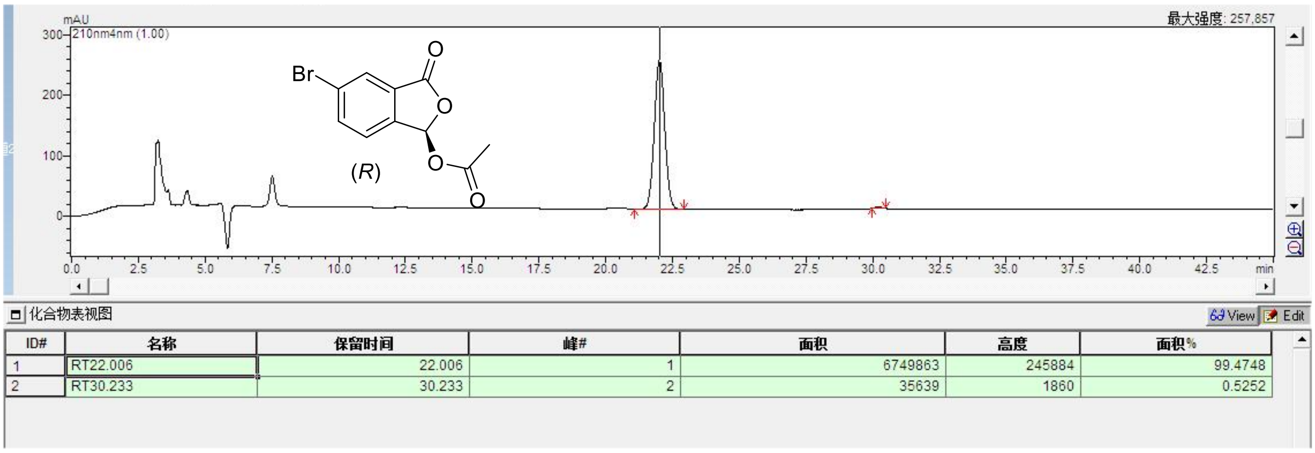

ID#	名称	保留时间	峰#	面积	高度	面积%
1	RT22.006	22.006	1	6749863	245884	99.4748
2	RT30.233	30.233	2	35639	1860	0.5252

<u>**Translation of all characters (Chinese) in the above two frameworks to English is as follows:**</u>

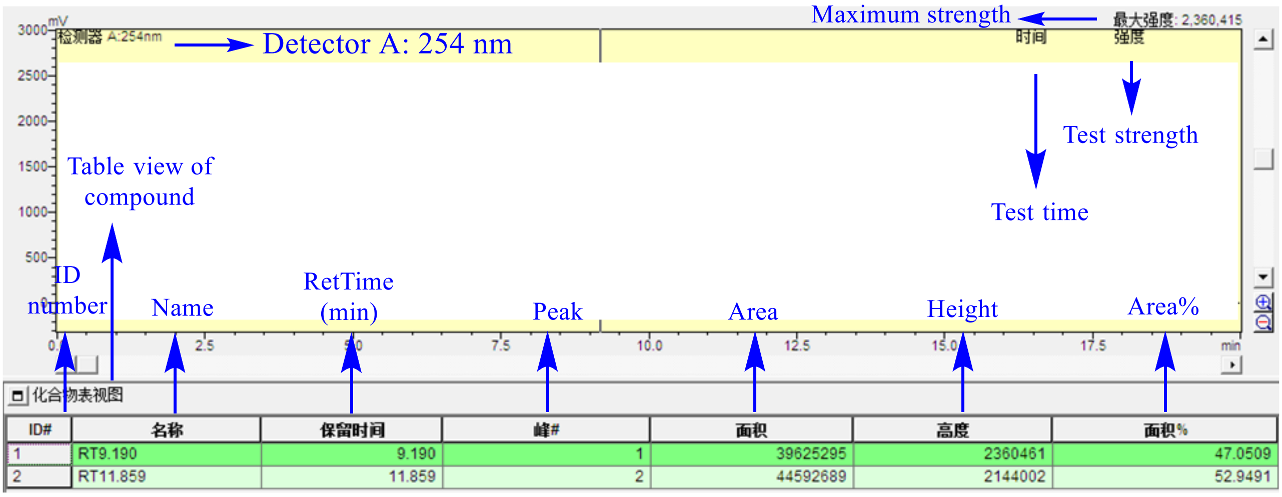

ID#	名称	保留时间	峰#	面积	高度	面积%
1	RT9.190	9.190	1	39625295	2360461	47.0509
2	RT11.859	11.859	2	44592689	2144002	52.9491

(*R*)-3k: (*R*)-7-bromo-3-oxo-1,3-dihydroisobenzofuran-1-yl acetate (HPLC: Chiralpak IC, detected at 210 nm, eluent: n-hexane/2-propanol = 80/20, flow rate = 1.0 mL/min, 25°C).

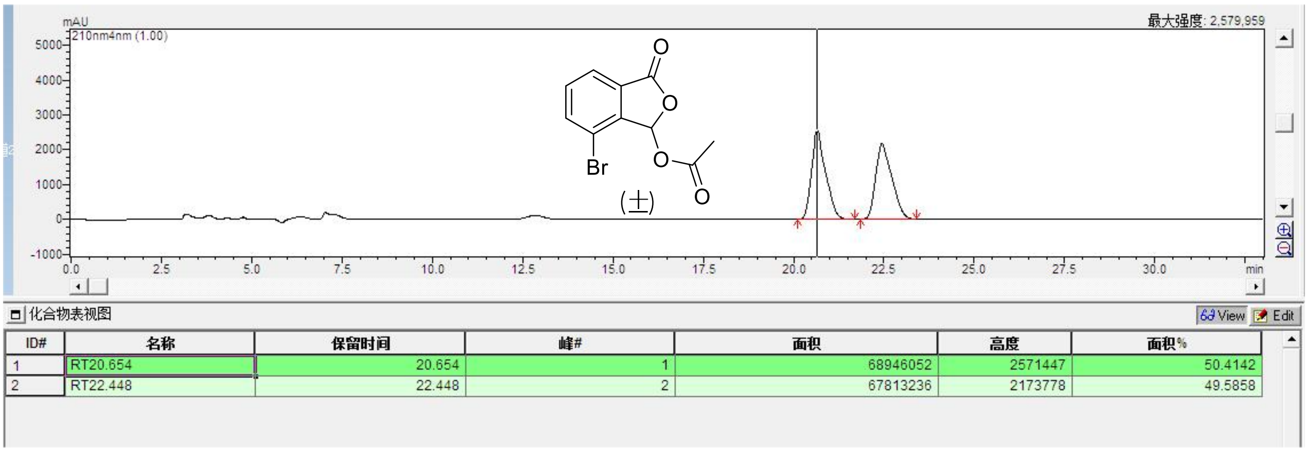

ID#	名称	保留时间	峰#	面积	高度	面积%
1	RT20.654	20.654	1	68946052	2571447	50.4142
2	RT22.448	22.448	2	67813236	2173778	49.5858

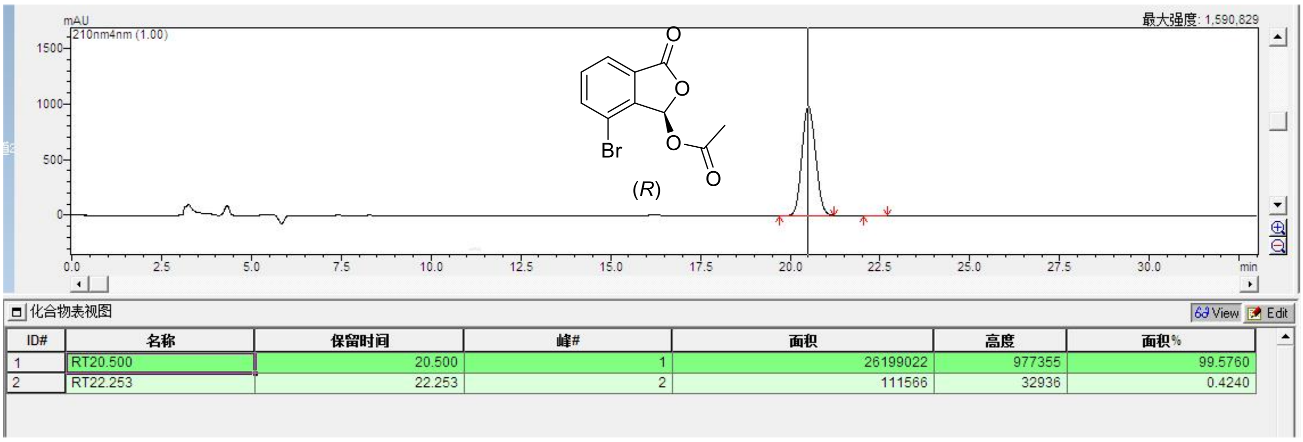

ID#	名称	保留时间	峰#	面积	高度	面积%
1	RT20.500	20.500	1	26199022	977355	99.5760
2	RT22.253	22.253	2	111566	32936	0.4240

<u>Translation of all characters (Chinese) in the above two frameworks to English is as follows:</u>

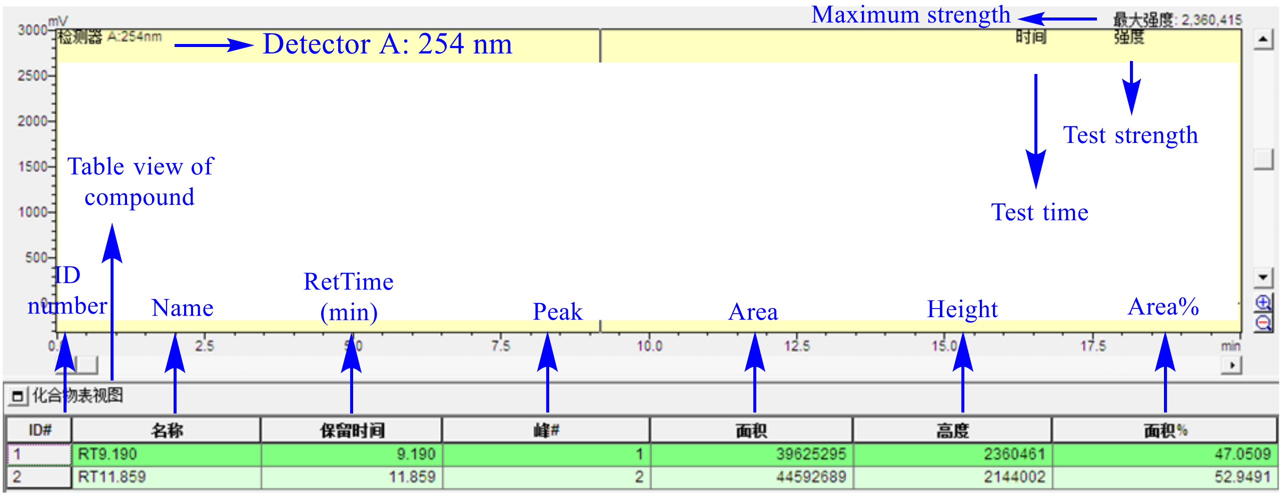

ID#	名称	保留时间	峰#	面积	高度	面积%
1	RT9.190	9.190	1	39625295	2360461	47.0509
2	RT11.859	11.859	2	44592689	2144002	52.9491

(*R*)-3l: (*R*)-5-iodo-3-oxo-1,3-dihydroisobenzofuran-1-yl acetate (HPLC: Chiralpak IC, detected at 210 nm, eluent: n-hexane/2-propanol = 80/20, flow rate = 1.0 mL/min, 25°C).

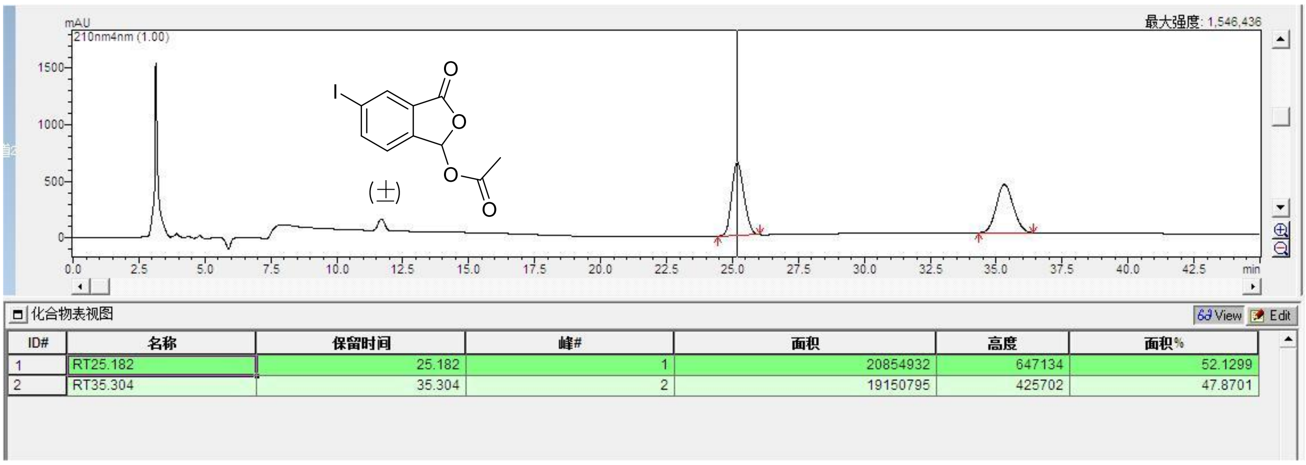

ID#	名称	保留时间	峰#	面积	高度	面积%
1	RT25.182	25.182	1	20854932	647134	52.1299
2	RT35.304	35.304	2	19150795	425702	47.8701

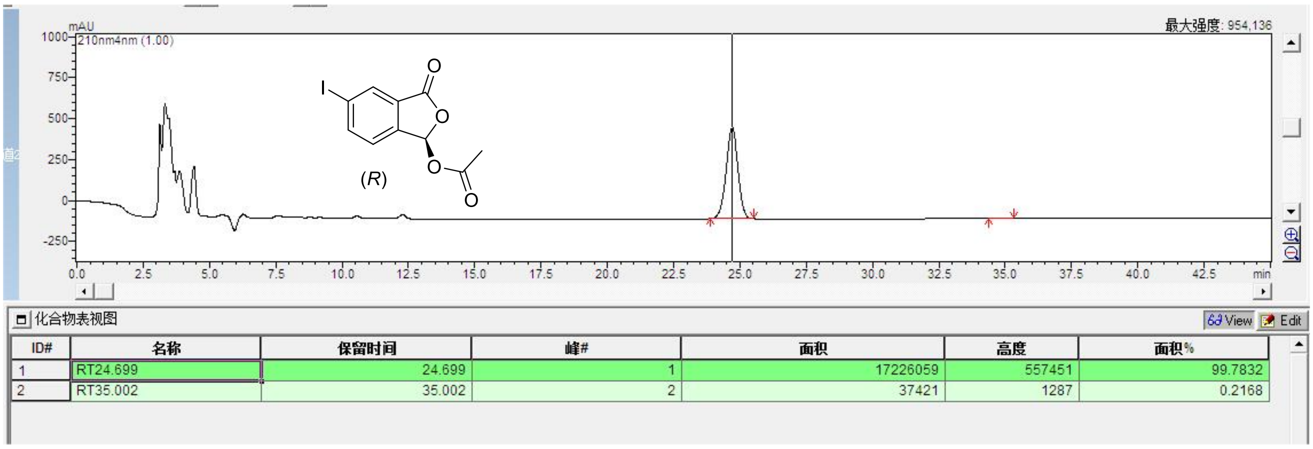

ID#	名称	保留时间	峰#	面积	高度	面积%
1	RT24.699	24.699	1	17226059	557451	99.7832
2	RT35.002	35.002	2	37421	1287	0.2168

Translation of all characters (Chinese) in the above two frameworks to English is as follows:

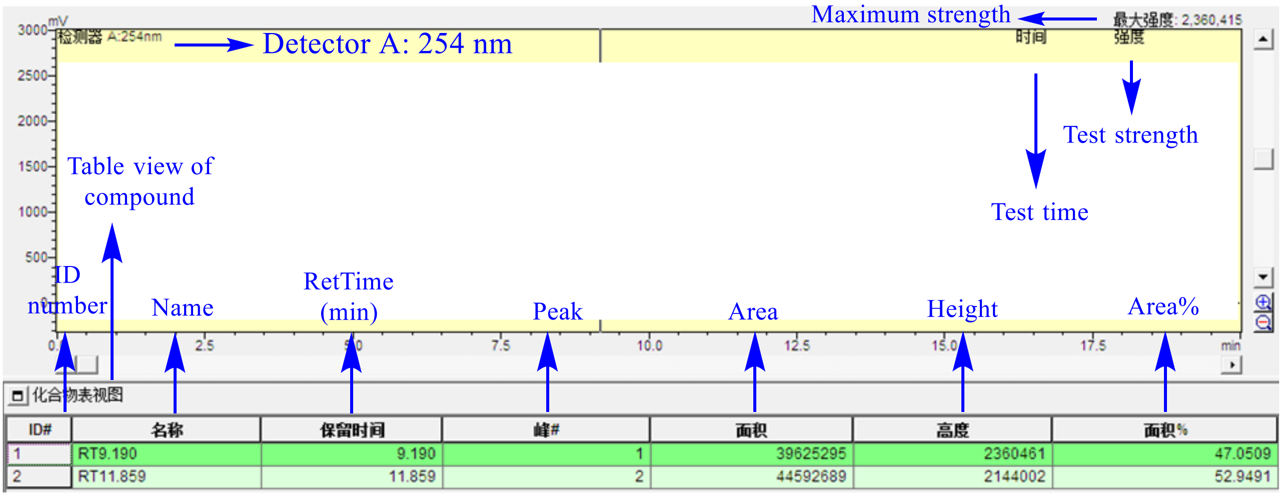

ID#	名称	保留时间	峰#	面积	高度	面积%
1	RT9.190	9.190	1	39625295	2360461	47.0509
2	RT11.859	11.859	2	44592689	2144002	52.9491

(*R*)-3m: (*R*)-5-cyano-3-oxo-1,3-dihydroisobenzofuran-1-yl acetate (HPLC: Chiralpak IC, detected at 210 nm, eluent: n-hexane/2-propanol = 80/20, flow rate = 1.0 mL/min, 25°C).

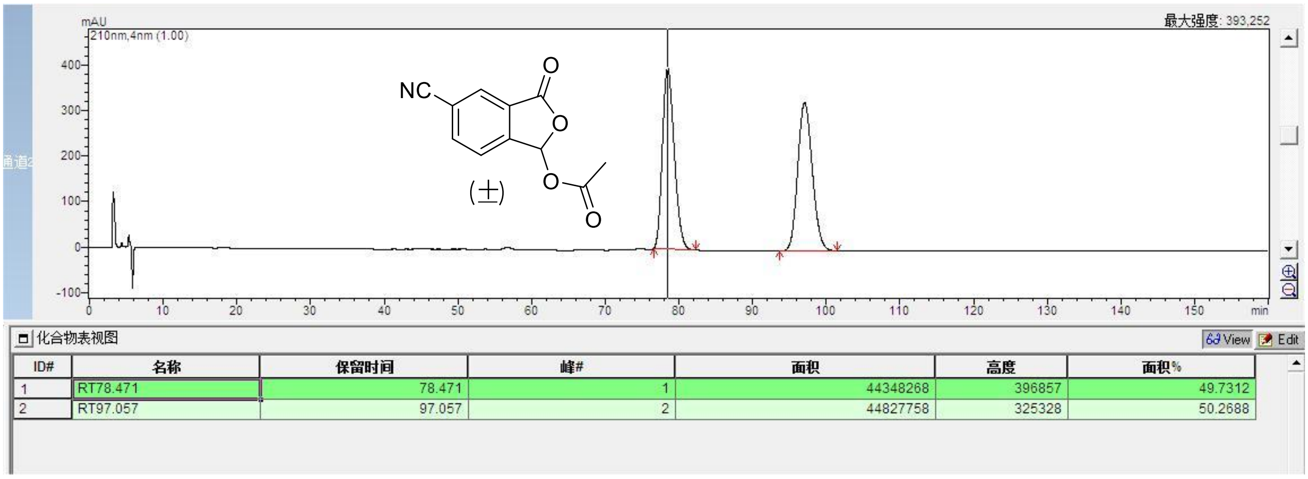

ID#	名称	保留时间	峰#	面积	高度	面积%
1	RT78.471	78.471	1	44348268	396857	49.7312
2	RT97.057	97.057	2	44827758	325328	50.2688

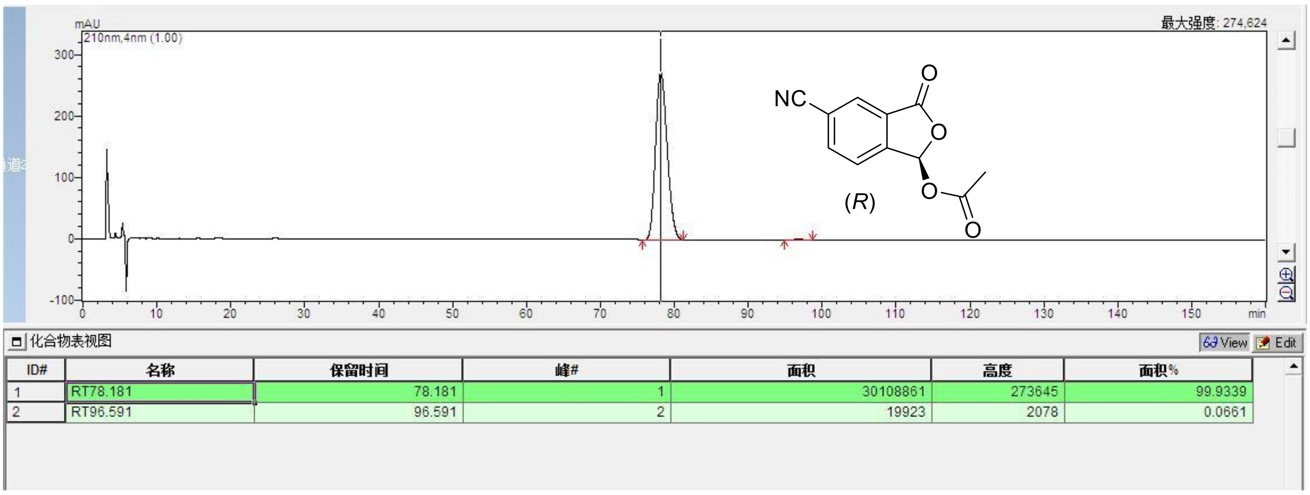

ID#	名称	保留时间	峰#	面积	高度	面积%
1	RT78.181	78.181	1	30108861	273645	99.9339
2	RT96.591	96.591	2	19923	2078	0.0661

Translation of all characters (Chinese) in the above two frameworks to English is as follows:

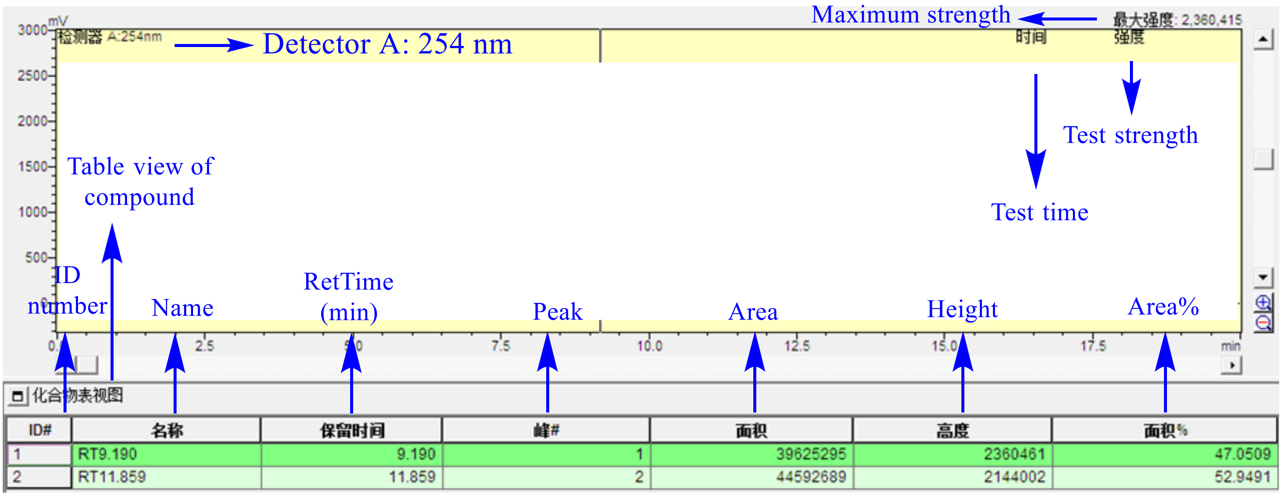

ID#	名称	保留时间	峰#	面积	高度	面积%
1	RT9.190	9.190	1	39625295	2360461	47.0509
2	RT11.859	11.859	2	44592689	2144002	52.9491

(*R*)-3n: (*R*)-5-nitro-3-oxo-1,3-dihydroisobenzofuran-1-yl acetate (HPLC: Chiralpak IC, detected at 210 nm, eluent: n-hexane/2-propanol = 80/20, flow rate = 1.0 mL/min, 25°C).

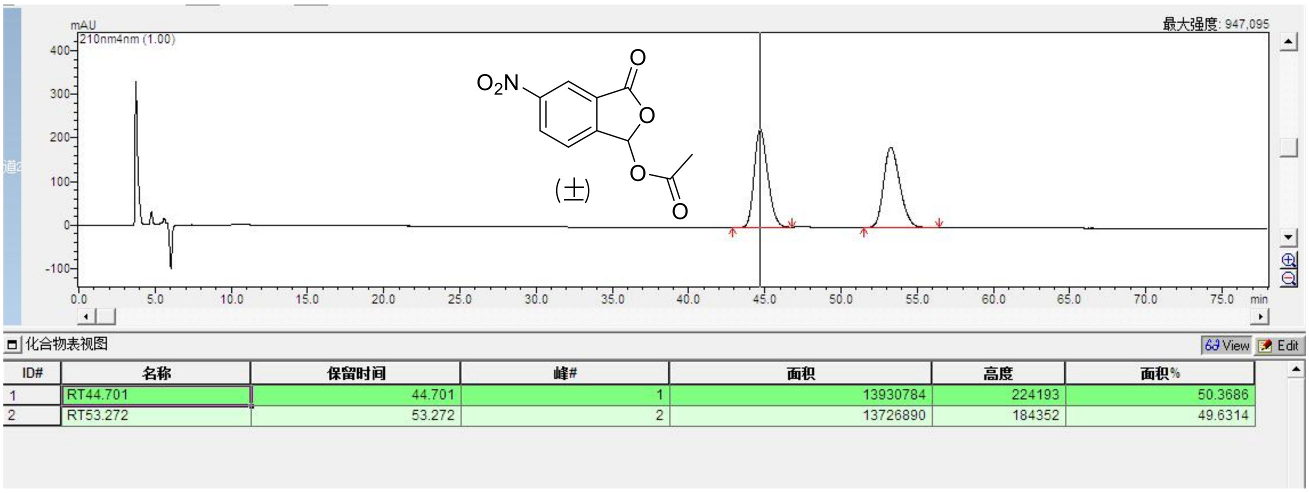

ID#	名称	保留时间	峰#	面积	高度	面积%
1	RT44.701	44.701	1	13930784	224193	50.3686
2	RT53.272	53.272	2	13726890	184352	49.6314

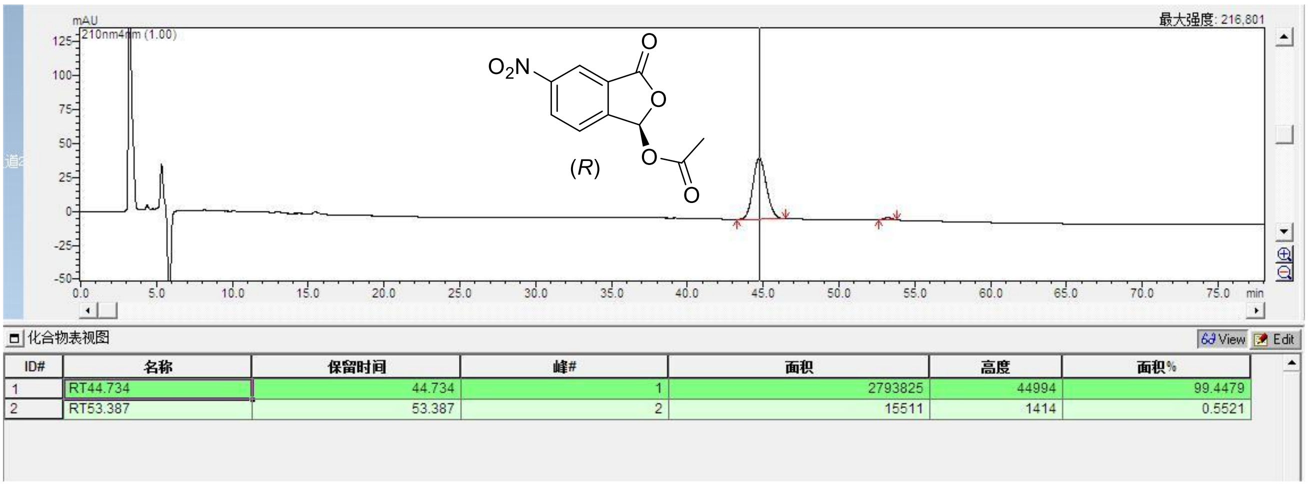

ID#	名称	保留时间	峰#	面积	高度	面积%
1	RT44.734	44.734	1	2793825	44994	99.4479
2	RT53.387	53.387	2	15511	1414	0.5521

<u>Translation of all characters (Chinese) in the above two frameworks to English is as follows:</u>

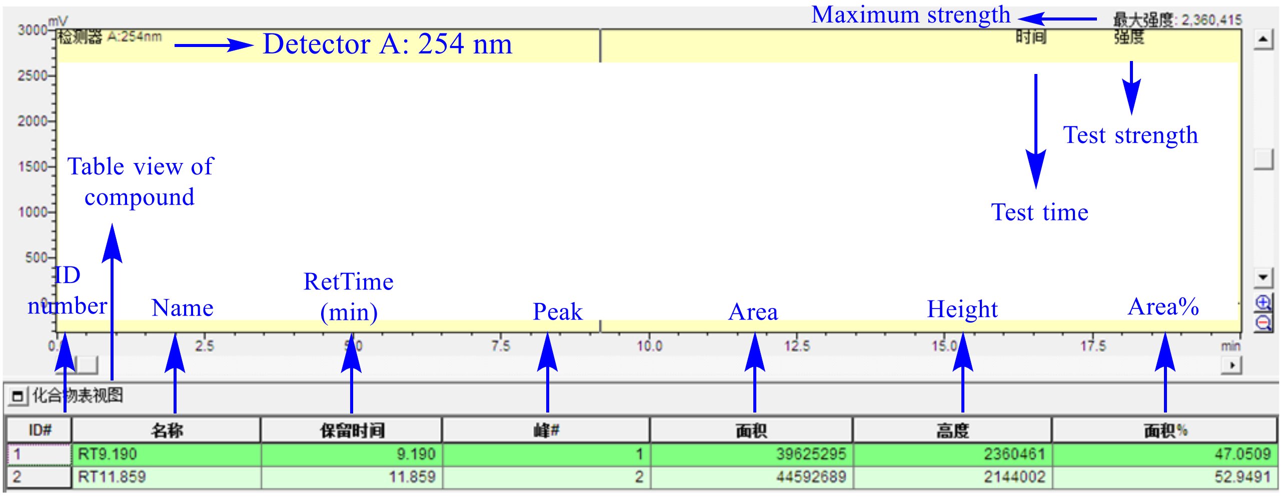

ID#	名称	保留时间	峰#	面积	高度	面积%
1	RT9.190	9.190	1	39625295	2360461	47.0509
2	RT11.859	11.859	2	44592689	2144002	52.9491

(*R*)-3o: (*R*)-5-methyl-3-oxo-1,3-dihydroisobenzofuran-1-yl acetate (HPLC: Chiralpak IC, detected at 210 nm, eluent: n-hexane/2-propanol = 80/20, flow rate = 1.0 mL/min, 25°C).

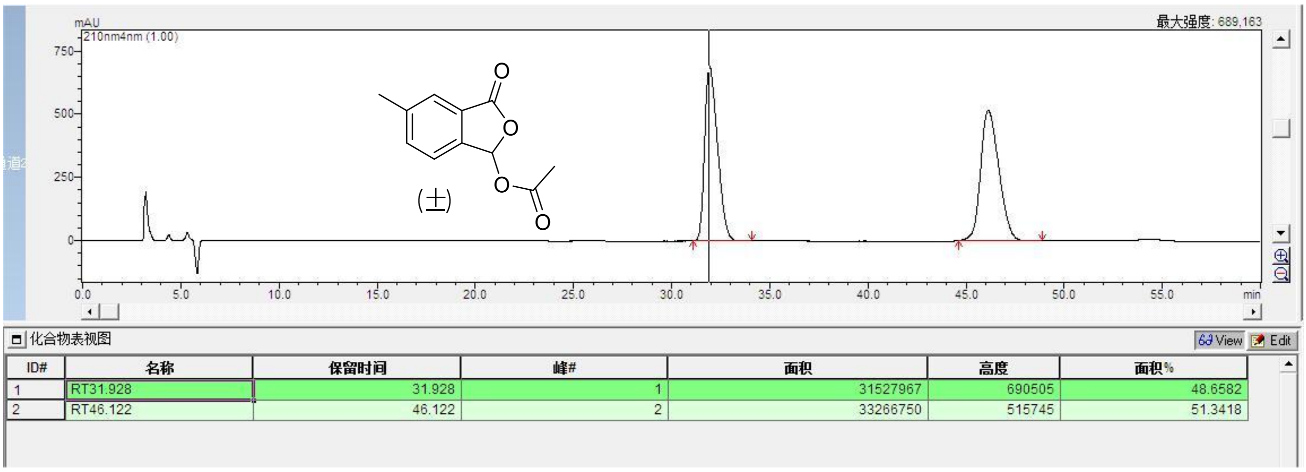

ID#	名称	保留时间	峰#	面积	高度	面积%
1	RT31.928	31.928	1	31527967	690505	48.6582
2	RT46.122	46.122	2	33266750	515745	51.3418

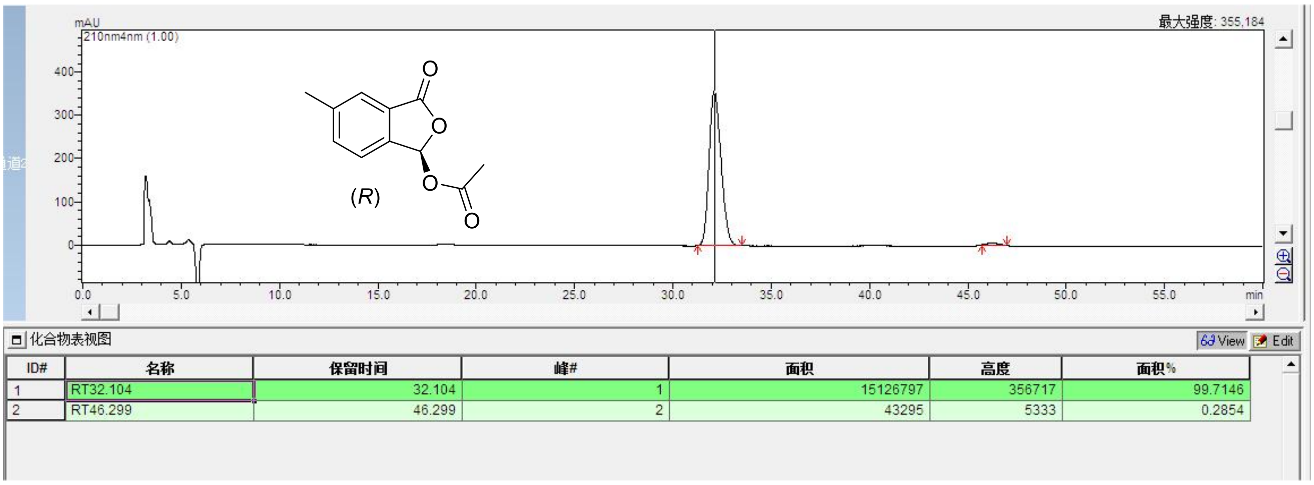

ID#	名称	保留时间	峰#	面积	高度	面积%
1	RT32.104	32.104	1	15126797	356717	99.7146
2	RT46.299	46.299	2	43295	5333	0.2854

Translation of all characters (Chinese) in the above two frameworks to English is as follows:

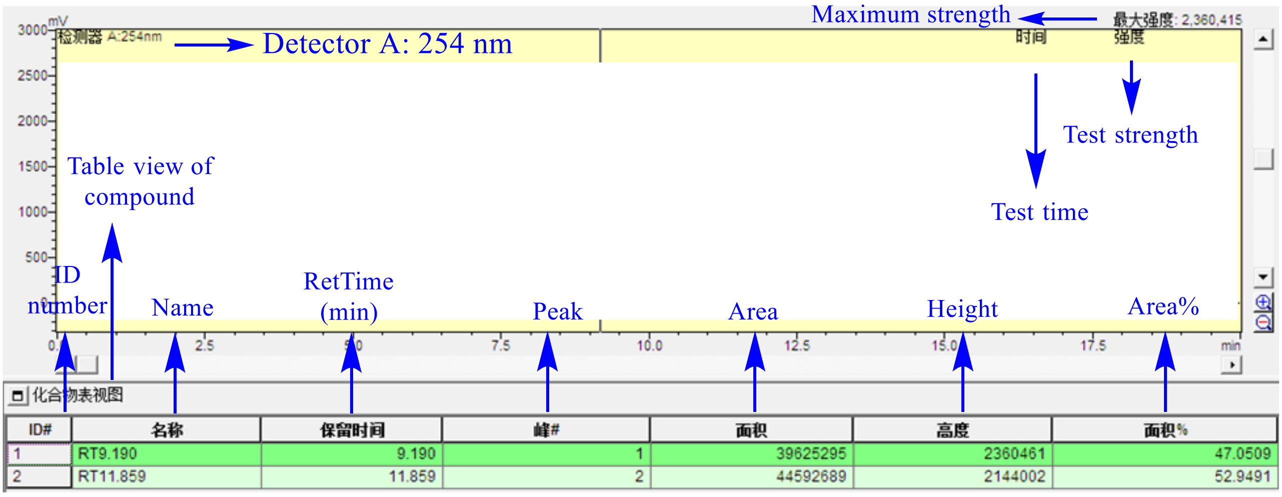

ID#	名称	保留时间	峰#	面积	高度	面积%
1	RT9.190	9.190	1	39625295	2360461	47.0509
2	RT11.859	11.859	2	44592689	2144002	52.9491

(*R*)-3p: (*R*)-6-methyl-3-oxo-1,3-dihydroisobenzofuran-1-yl acetate. (HPLC: Chiralpak AD-H, detected at 254 nm, eluent: n-hexane/2-propanol = 80/20, flow rate = 1.0 mL/min, 25°C).

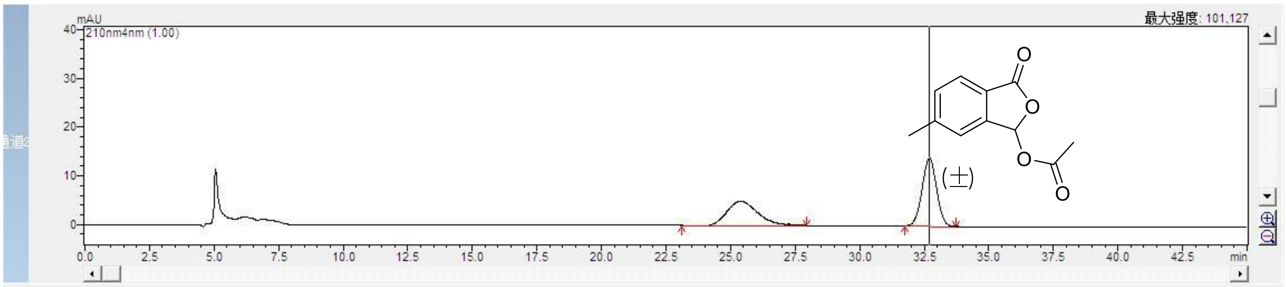

化合物表视图

ID#	名称	保留时间	峰#	面积	高度	面积%
1	RT25.424	25.323	1	432387	5116	49.9207
2	RT32.696	32.186	2	433761	12579	50.0793

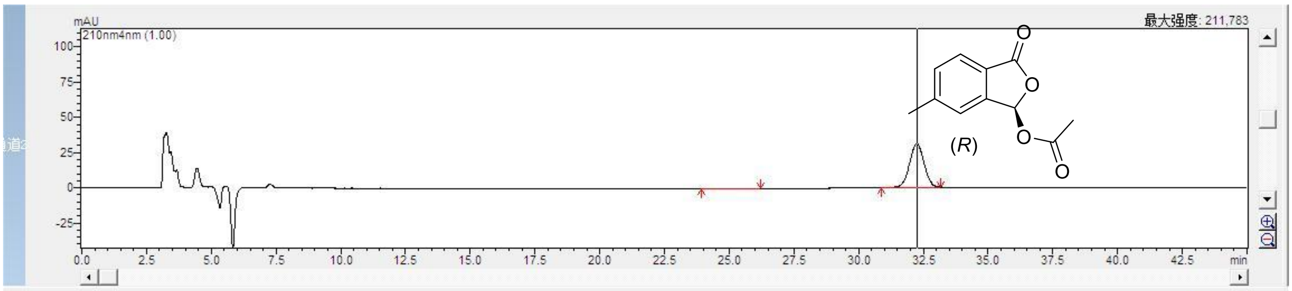

化合物表视图

ID#	名称	保留时间	峰#	面积	高度	面积%
1	RT25.213	25.213	1	6627	285	0.5551
2	RT32.249	32.249	2	1187153	30785	99.4449

__**Translation of all characters (Chinese) in the above two frameworks to English is as follows:**__

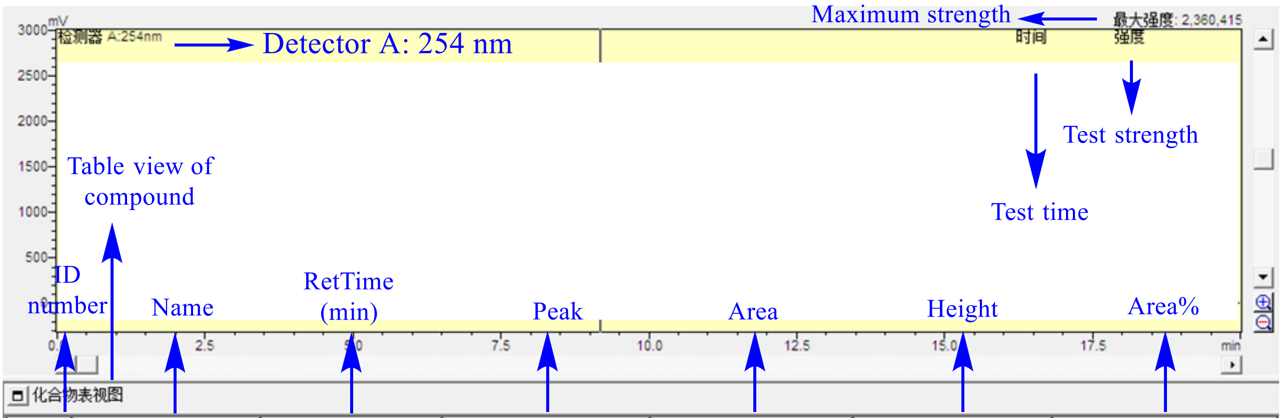

ID#	名称	保留时间	峰#	面积	高度	面积%
1	RT9.190	9.190	1	39625295	2360461	47.0509
2	RT11.859	11.859	2	44592689	2144002	52.9491

(R)-3q: (*R*)-6-methoxy-3-oxo-1,3-dihydroisobenzofuran-1-yl acetate (HPLC: Chiracel IC, detected at 254 nm, eluent: n-hexane/2-propanol = 80/20, flow rate = 1.0 mL/min, 25°C).

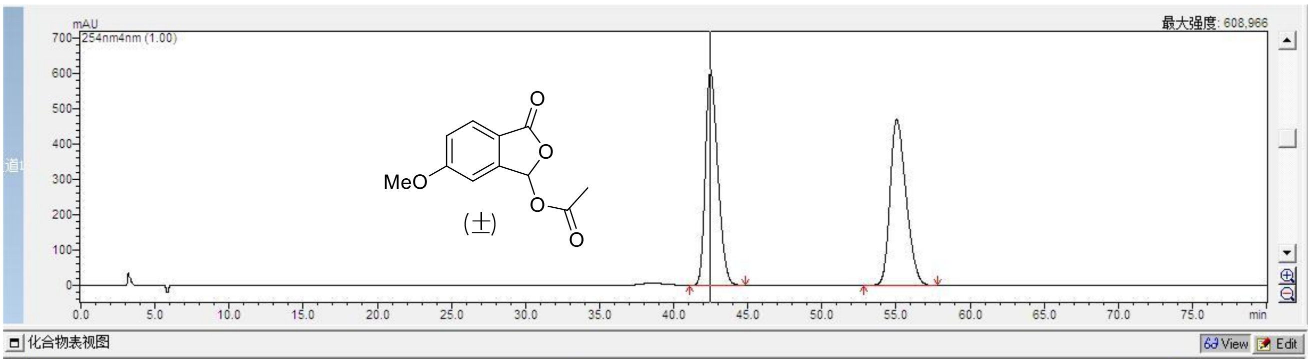

ID#	名称	保留时间	峰#	面积	高度	面积%
1	RT42.479	42.479	1	35423000	608537	49.9225
2	RT55.044	55.044	2	35532955	469794	50.0775

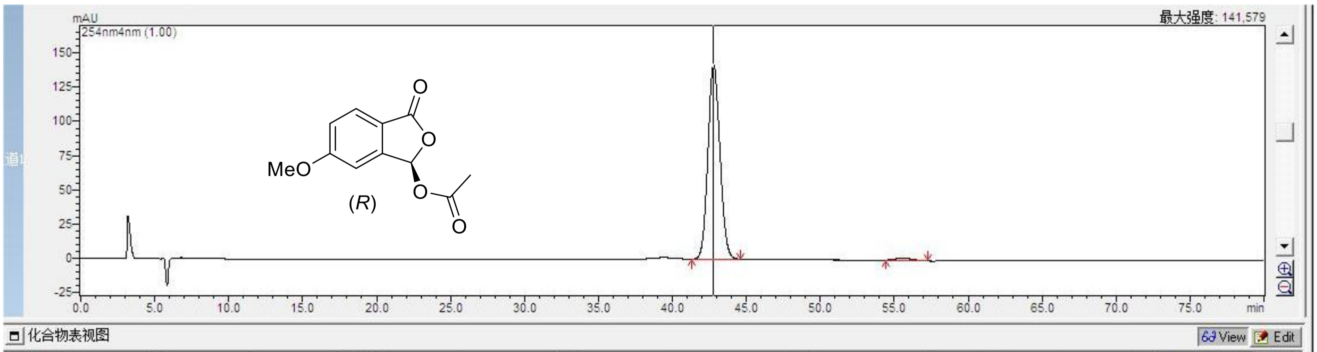

ID#	名称	保留时间	峰#	面积	高度	面积%
1	RT42.785	42.785	1	8041495	142129	99.7056
2	RT55.494	55.494	2	23741	2644	0.2944

<u>Translation of all characters (Chinese) in the above two frameworks to English is as follows:</u>

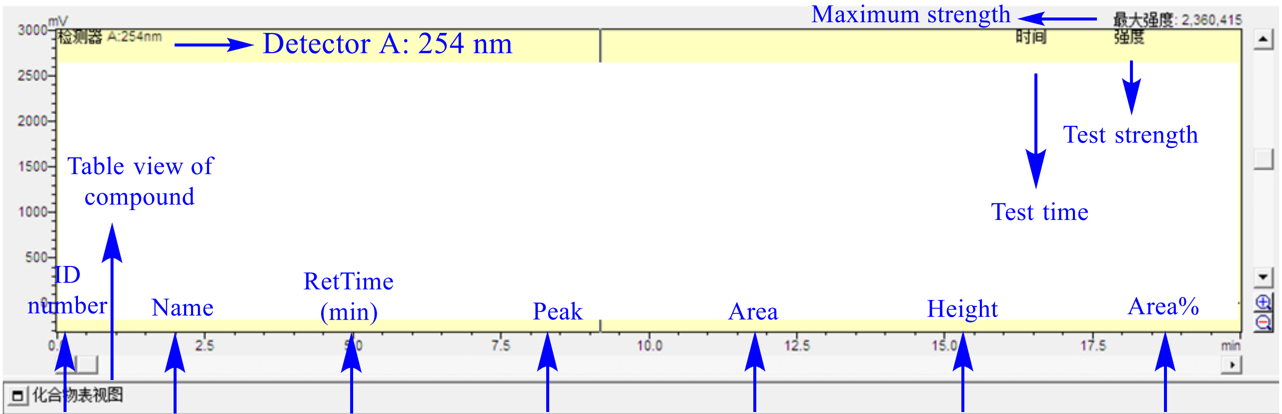

ID#	名称	保留时间	峰#	面积	高度	面积%
1	RT9.190	9.190	1	39625295	2360461	47.0509
2	RT11.859	11.859	2	44592689	2144002	52.9491

(*R*)-3r: (*R*)-5-oxo-5,7-dihydrofuro[3,4-b]pyridin-7-yl acetate. (HPLC: Chiralpak IC, detected at 210 nm, eluent: n-hexane/2-propanol = 80/20, flow rate = 1.0 mL/min, 25°C).

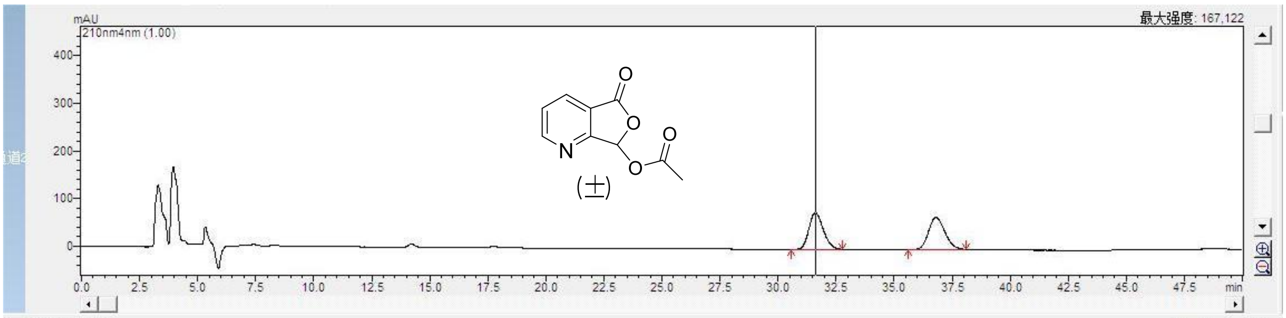

化合物表视图

ID#	名称	保留时间	峰#	面积	高度	面积%
1	RT31.602	31.602	1	3324834	77029	50.2326
2	RT36.804	36.804	2	3294042	67179	49.7674

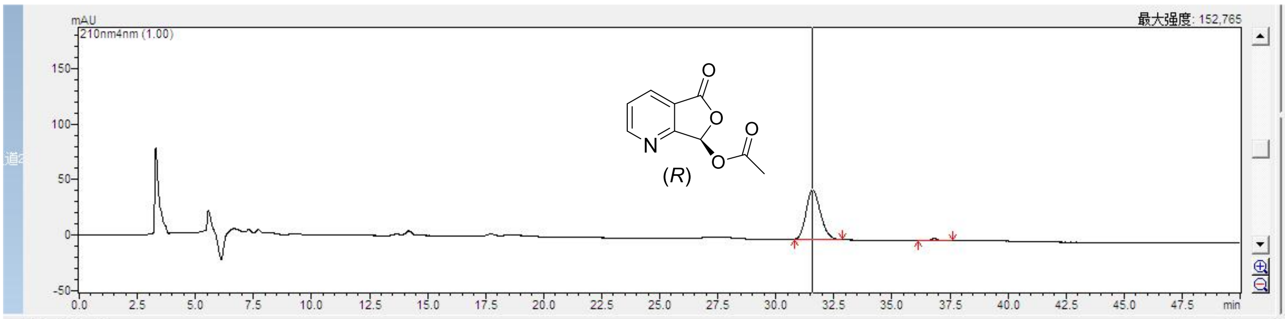

化合物表视图

ID#	名称	保留时间	峰#	面积	高度	面积%
1	RT31.582	31.582	1	1922469	44900	98.9584
2	RT36.769	36.769	2	20235	1928	1.0416

<u>Translation of all characters (Chinese) in the above two frameworks to English is as follows:</u>

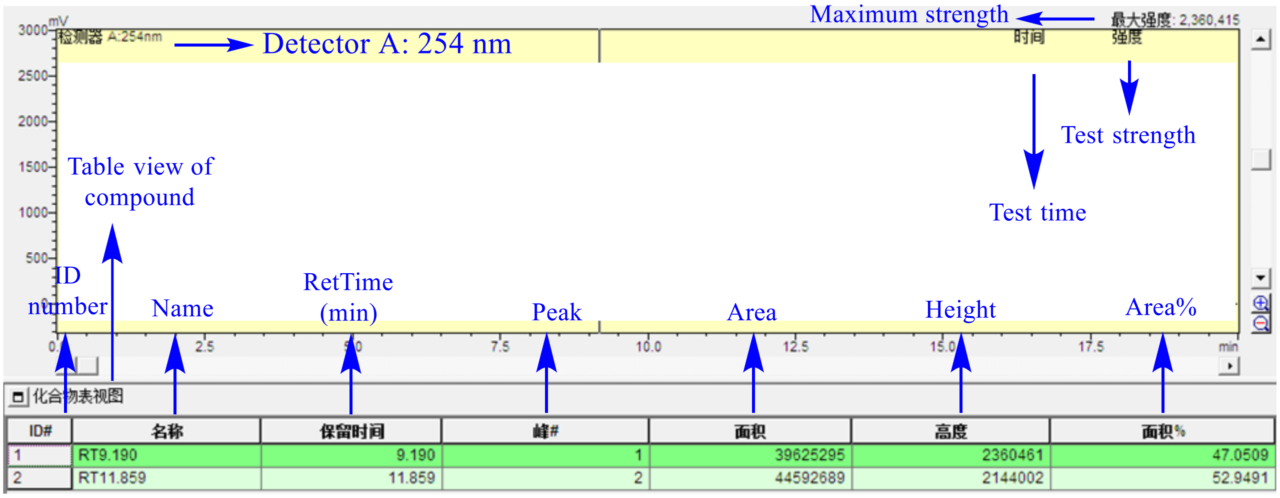

ID#	名称	保留时间	峰#	面积	高度	面积%
1	RT9.190	9.190	1	39625295	2360461	47.0509
2	RT11.859	11.859	2	44592689	2144002	52.9491

(*R*)-3s: (*R*)-3-oxo-1,3-dihydroisobenzofuran-1-yl butyrate (HPLC: Chiralpak IC, detected at 210 nm, eluent: n-hexane/2-propanol = 80/20, flow rate = 1.0 mL/min, 25°C).

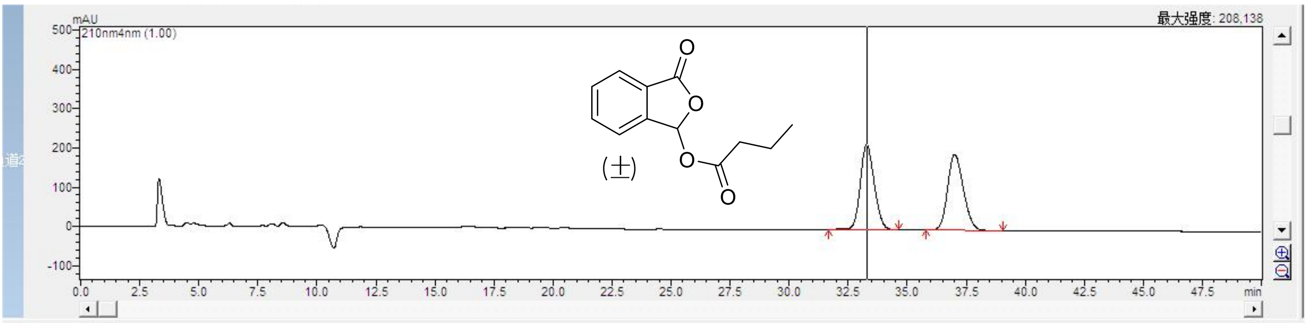

化合物表视图

ID#	名称	保留时间	峰#	面积	高度	面积%
1	RT33.279	33.279	1	8986735	216935	50.2987
2	RT37.007	37.007	2	8880003	193271	49.7013

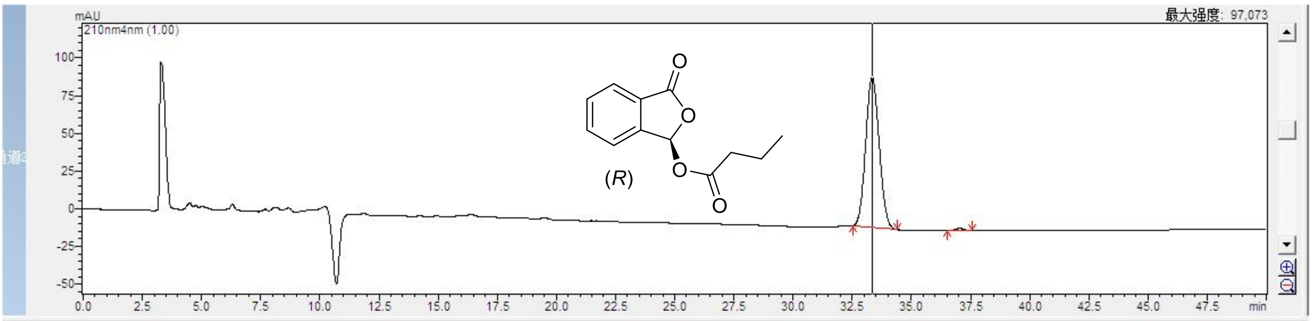

化合物表视图

ID#	名称	保留时间	峰#	面积	高度	面积%
1	RT33.336	33.336	1	3966450	99607	99.4605
2	RT37.071	37.071	2	21513	3210	0.5395

<u>Translation of all characters (Chinese) in the above two frameworks to English is as follows:</u>

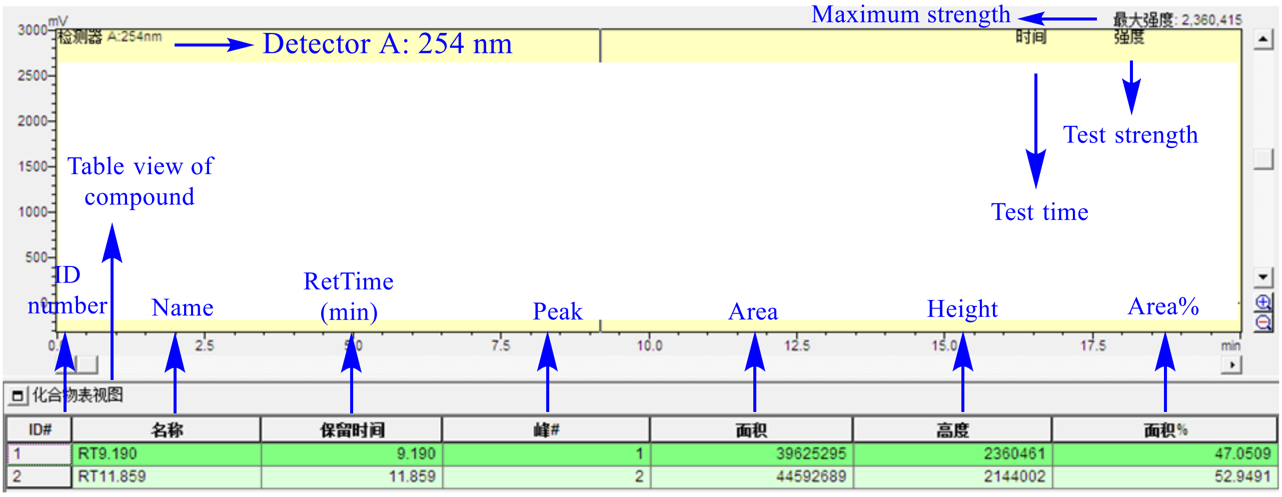

(***R***)**-3t: (*****R*****)-3-oxo-1,3-dihydroisobenzofuran-1-yl isobutyrate** (HPLC: Chiralpak IC, detected at 210 nm, eluent: n-hexane/2-propanol = 80/20, flow rate = 1.0 mL/min, 25°C).

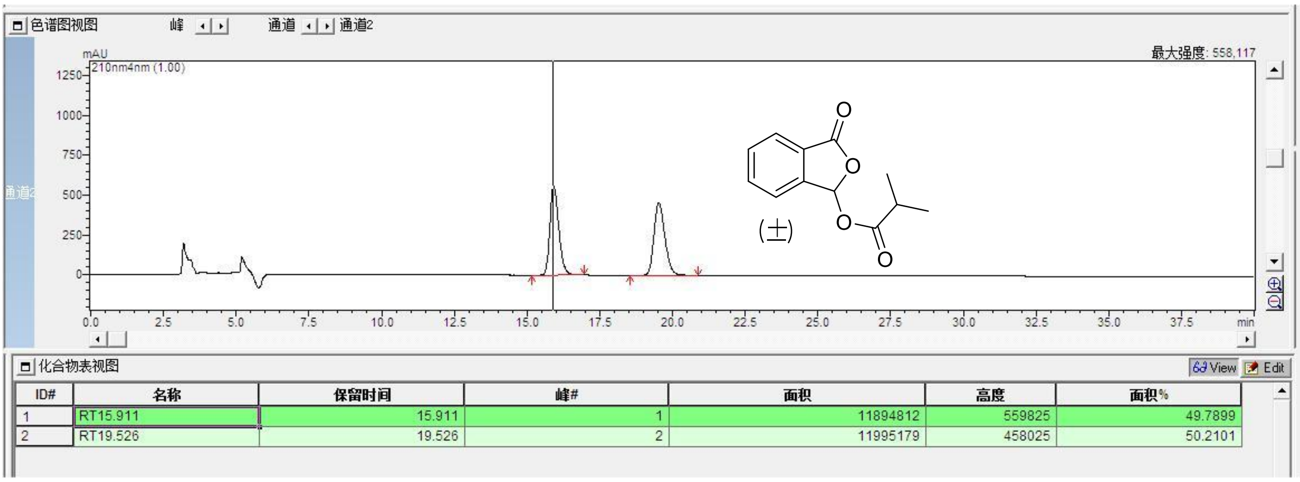

ID#	名称	保留时间	峰#	面积	高度	面积%
1	RT15.911	15.911	1	11894812	559825	49.7899
2	RT19.526	19.526	2	11995179	458025	50.2101

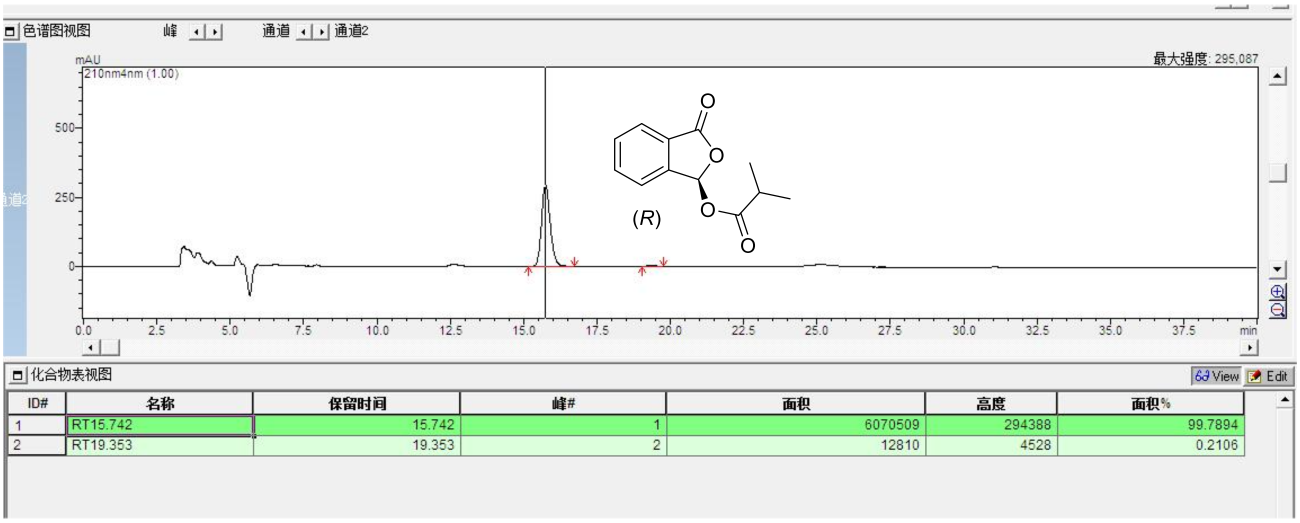

ID#	名称	保留时间	峰#	面积	高度	面积%
1	RT15.742	15.742	1	6070509	294388	99.7894
2	RT19.353	19.353	2	12810	4528	0.2106

Translation of all characters (Chinese) in the above two frameworks to English is as follows:

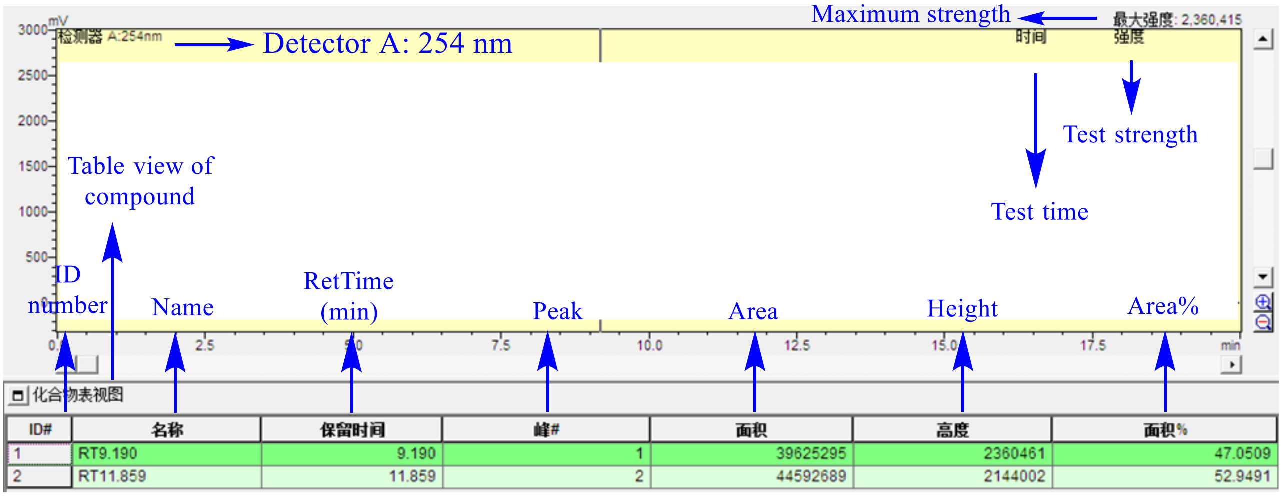

ID#	名称	保留时间	峰#	面积	高度	面积%
1	RT9.190	9.190	1	39625295	2360461	47.0509
2	RT11.859	11.859	2	44592689	2144002	52.9491

Characterizations of chiral products ((*R*)-3a-(*R*)-3t).

(*R*)-3a: (*R*)-3-oxo-1,3-dihydroisobenzofuran-1-yl acetate.

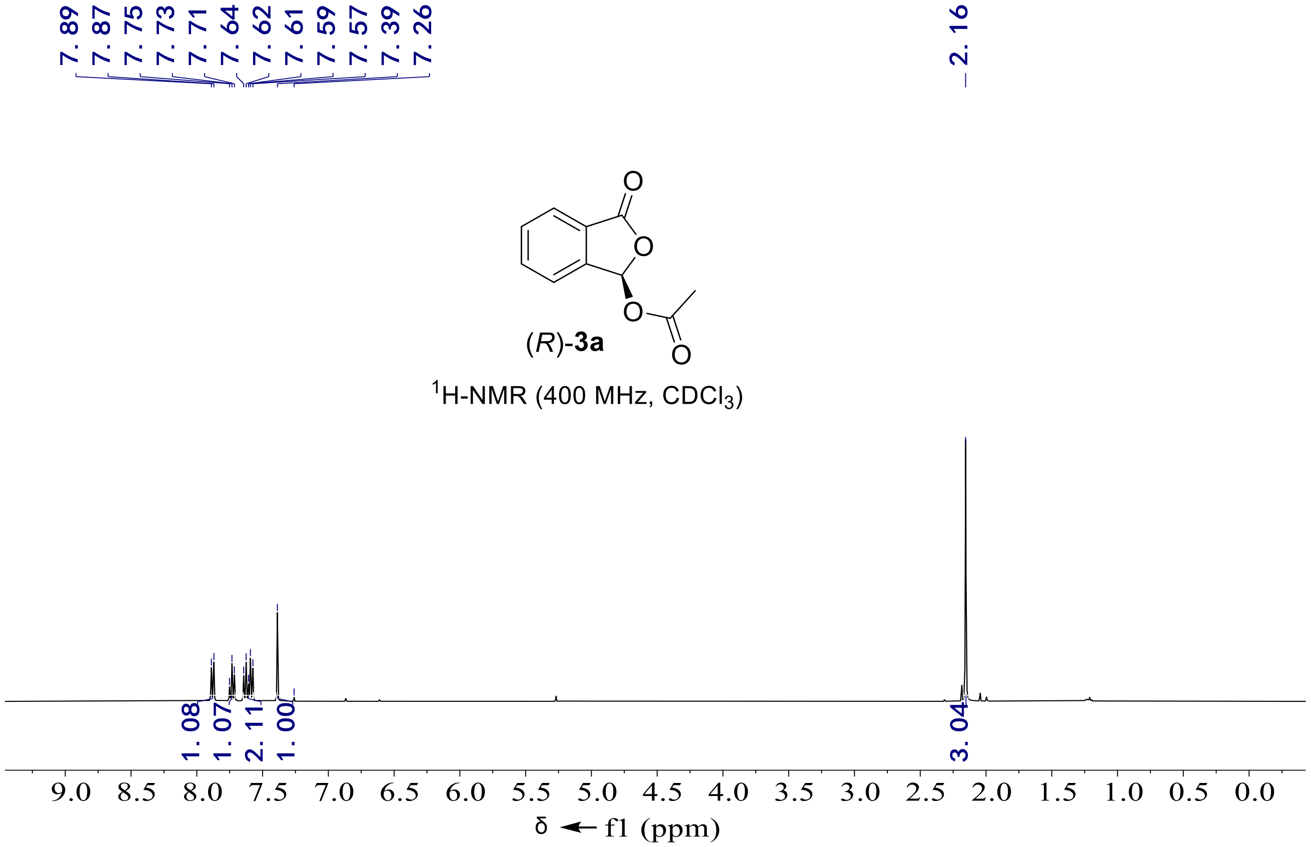

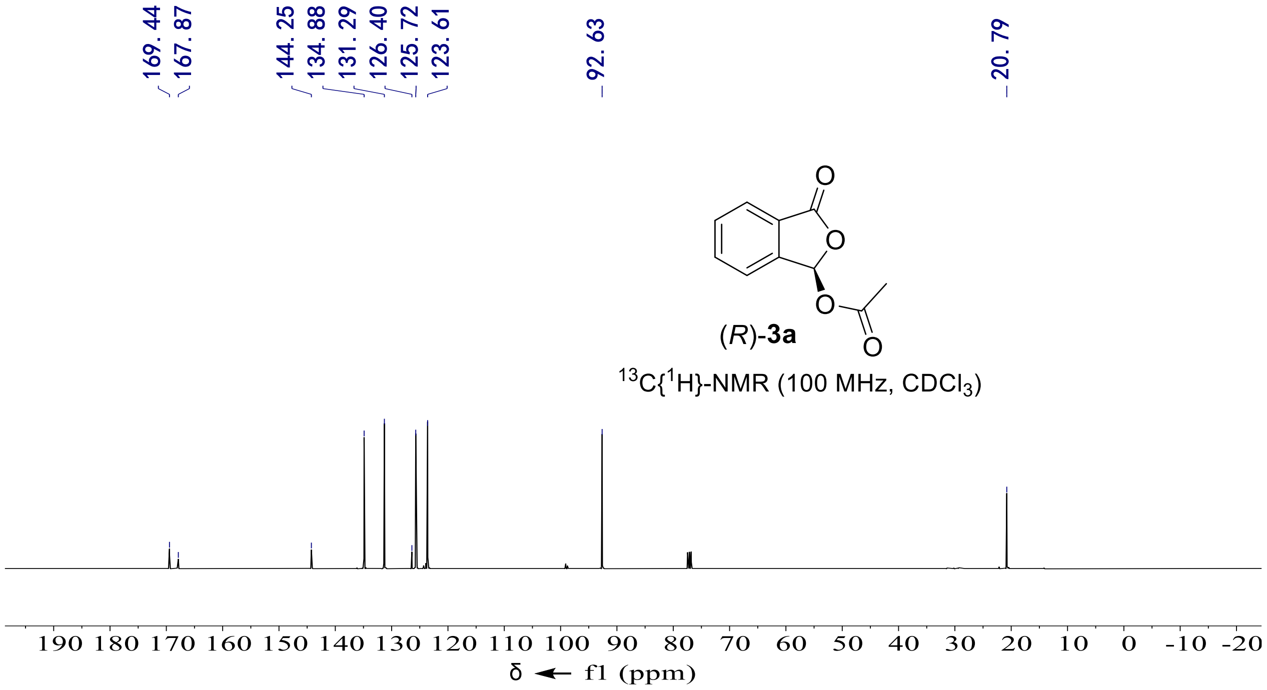

(*R*)-3b: (*R*)-4-fluoro-3-oxo-1,3-dihydroisobenzofuran-1-yl acetate.

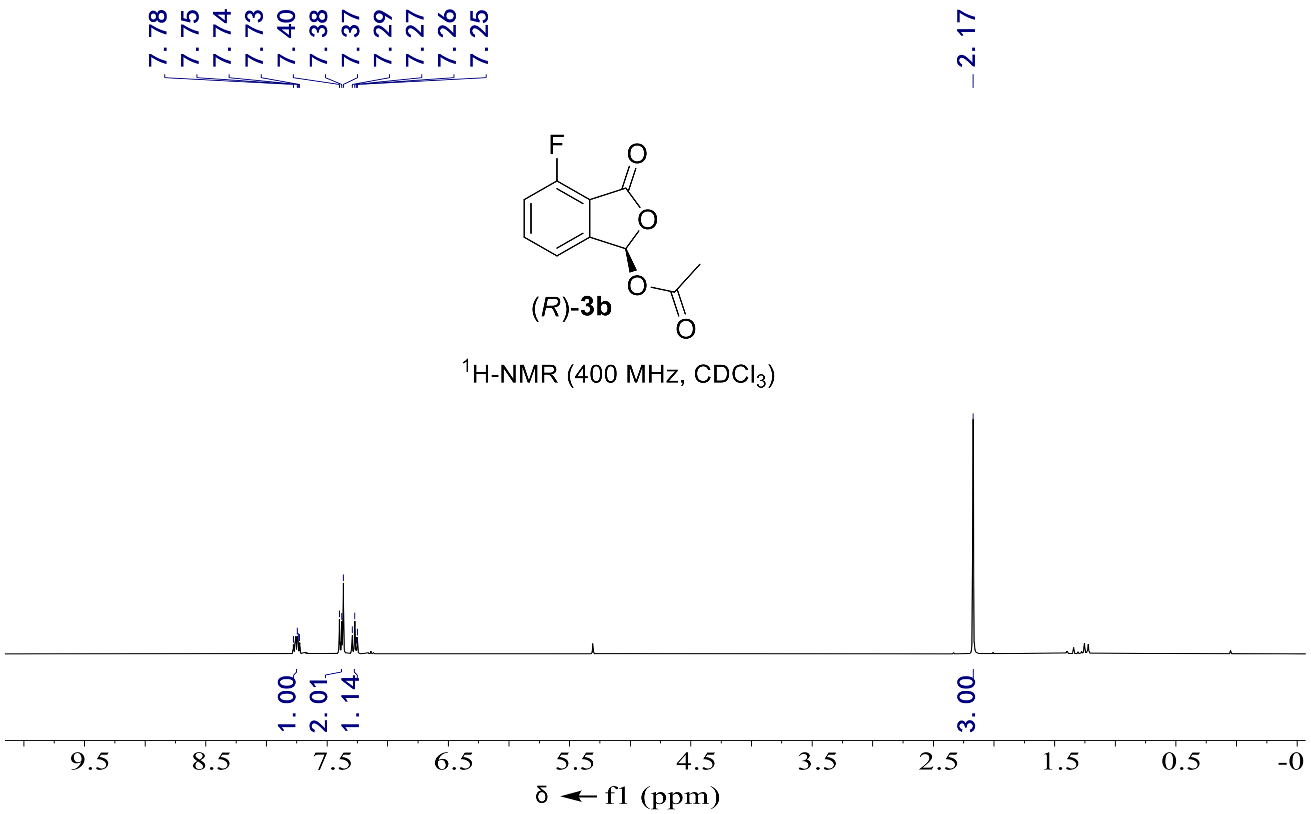

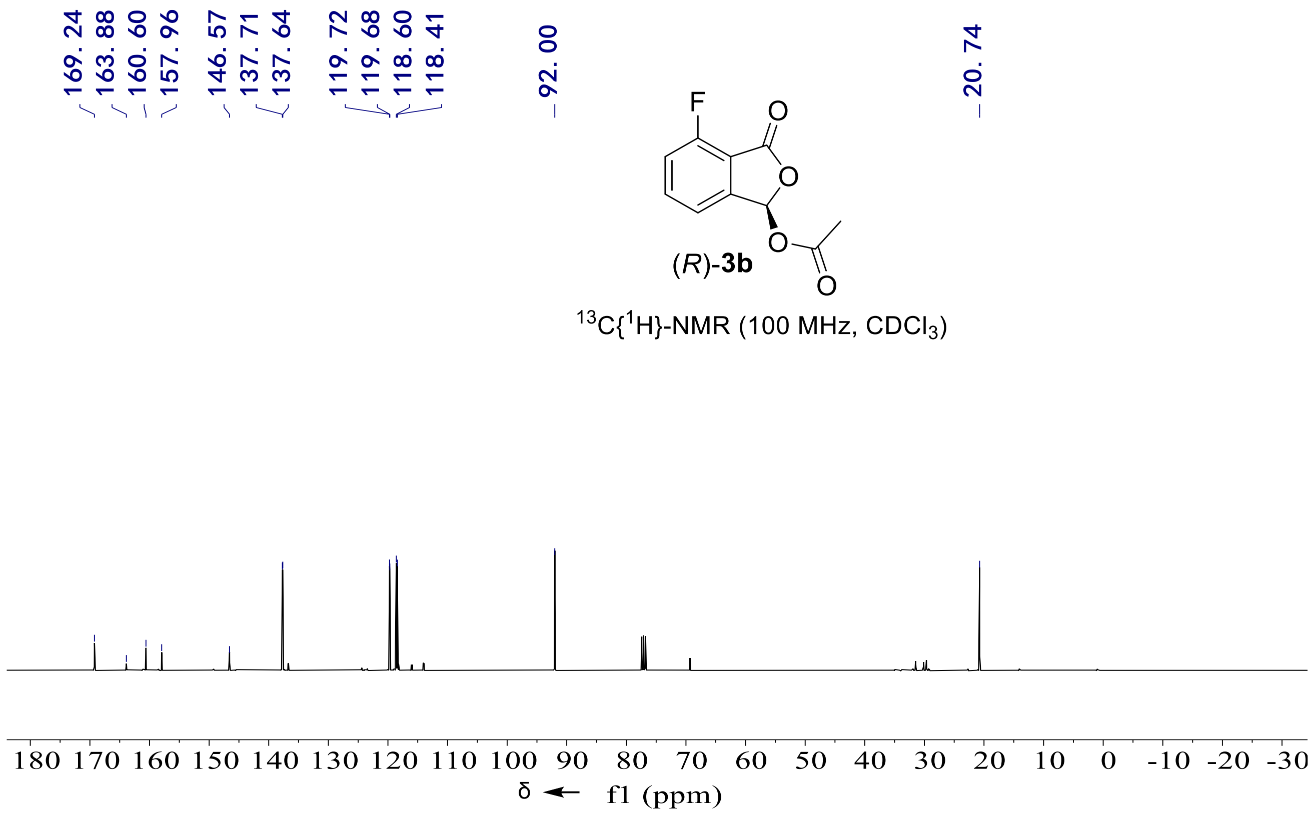

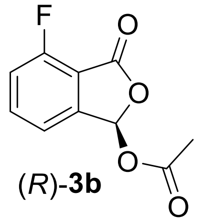

(*R*)-**3b**

^{19}F-NMR (376 MHz, $CDCl_3$)

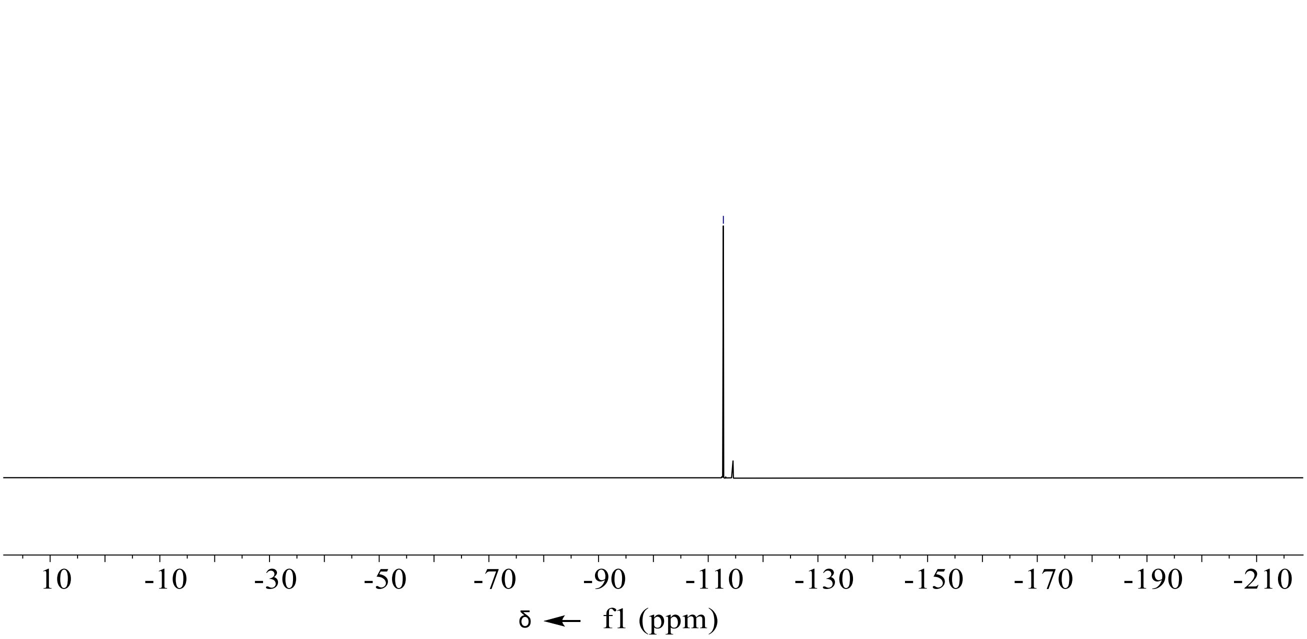

(*R*)-3c: (*R*)-5-fluoro-3-oxo-1,3-dihydroisobenzofuran-1-yl acetate.

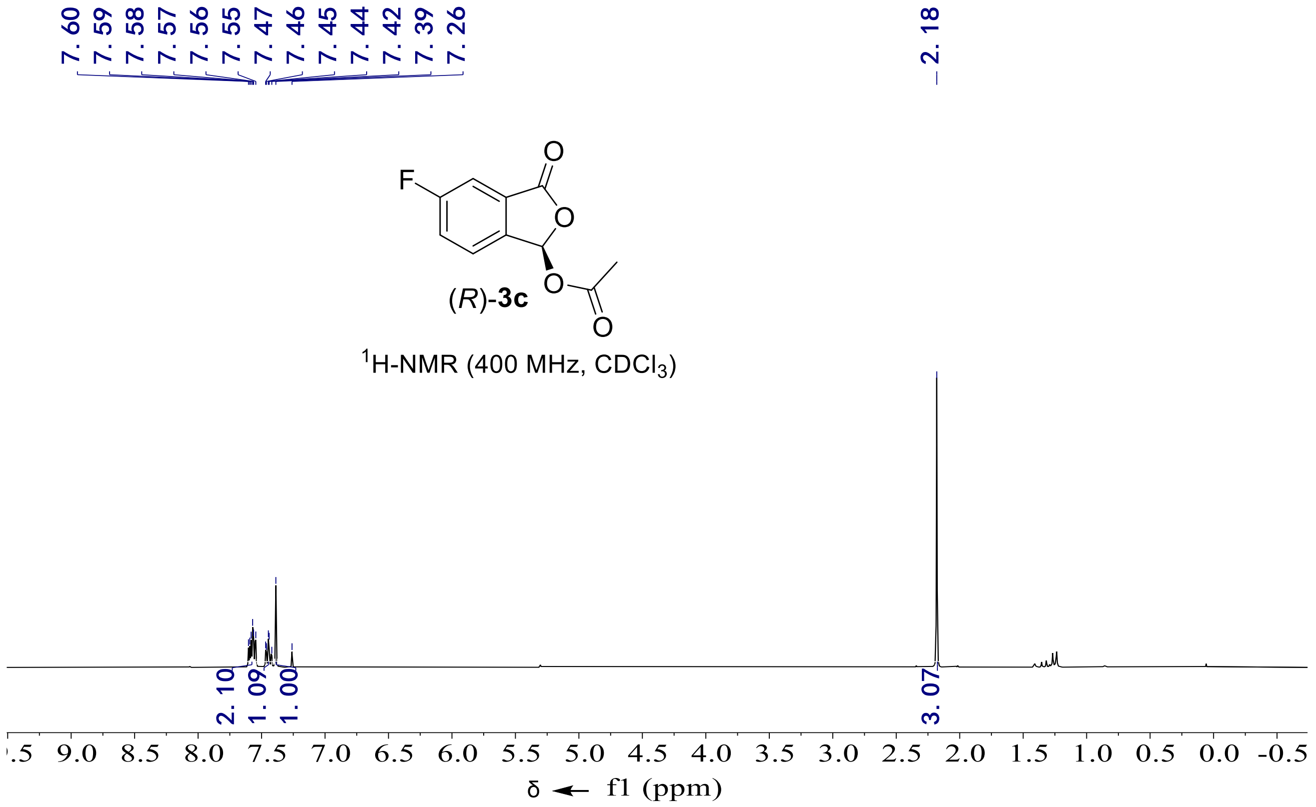

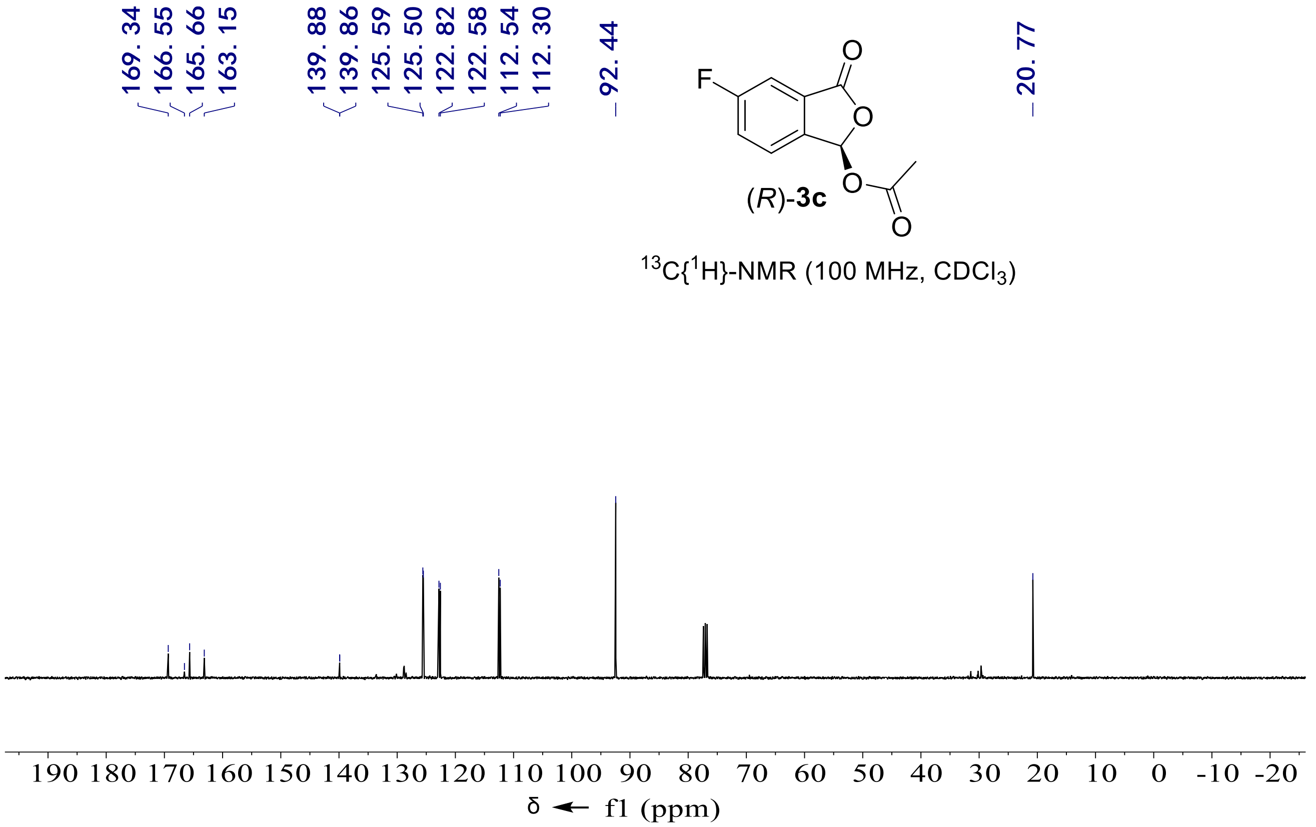

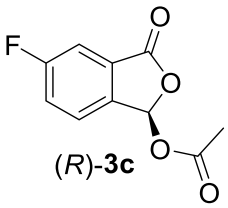

(*R*)-**3c**

^{19}F-NMR (376 MHz, $CDCl_3$)

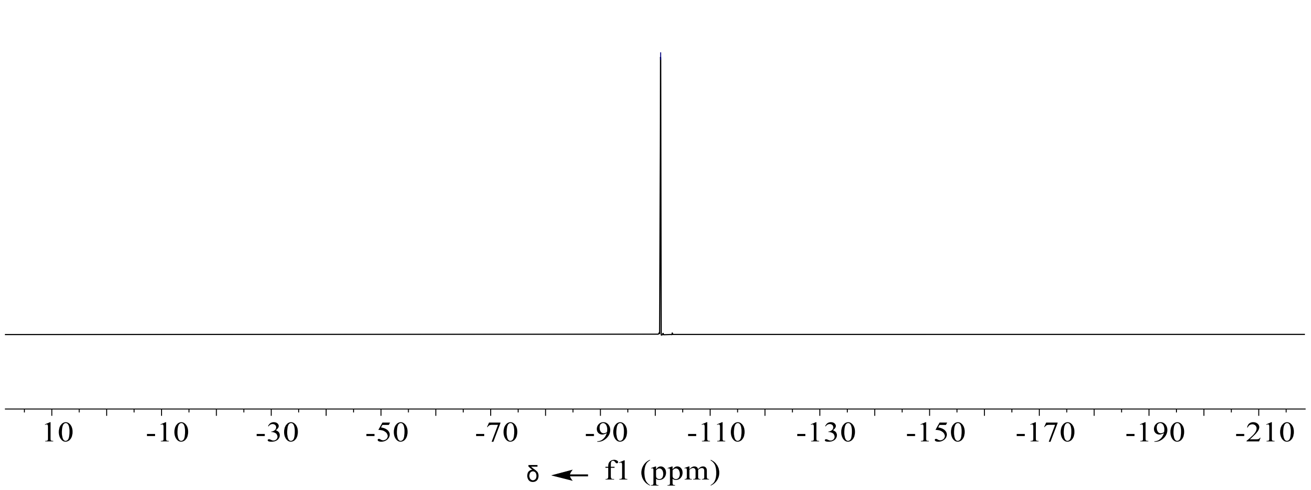

(*R*)-3d: (*R*)-6-fluoro-3-oxo-1,3-dihydroisobenzofuran-1-yl acetate.

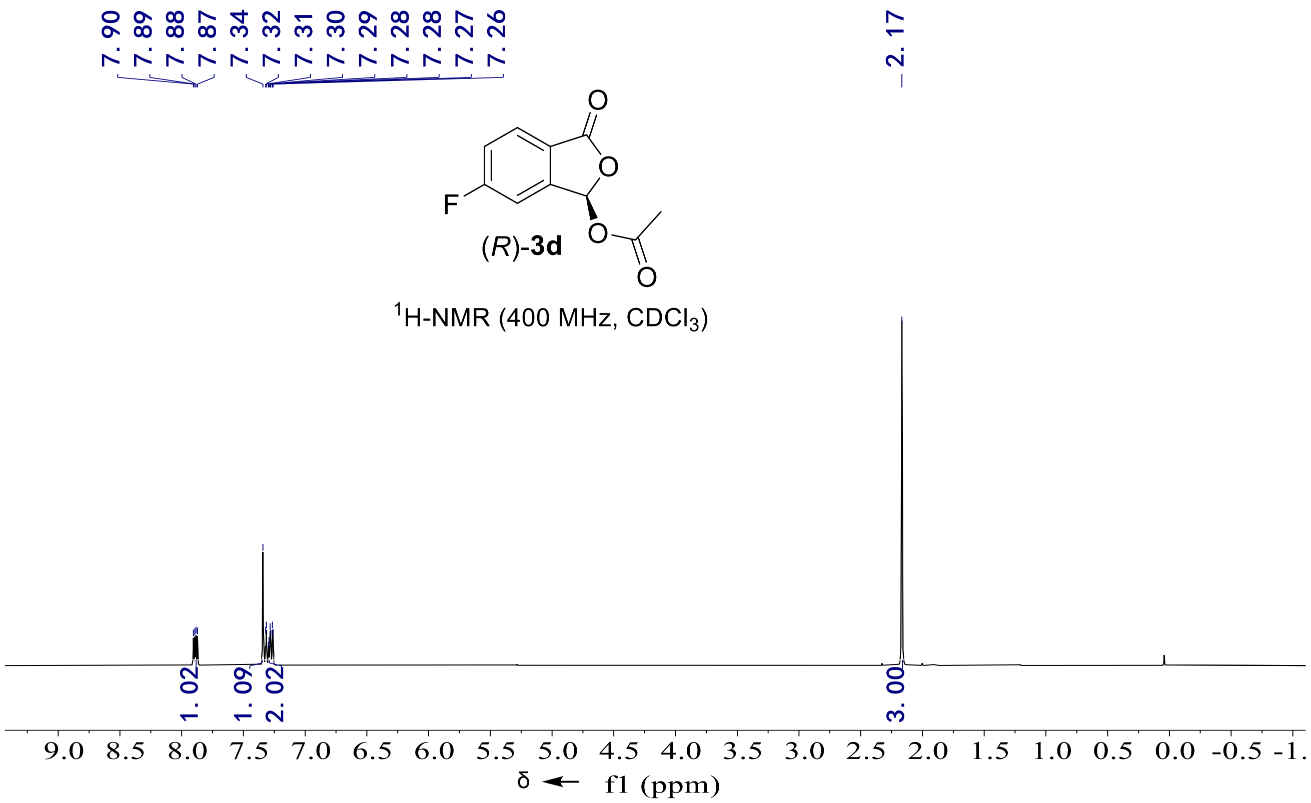

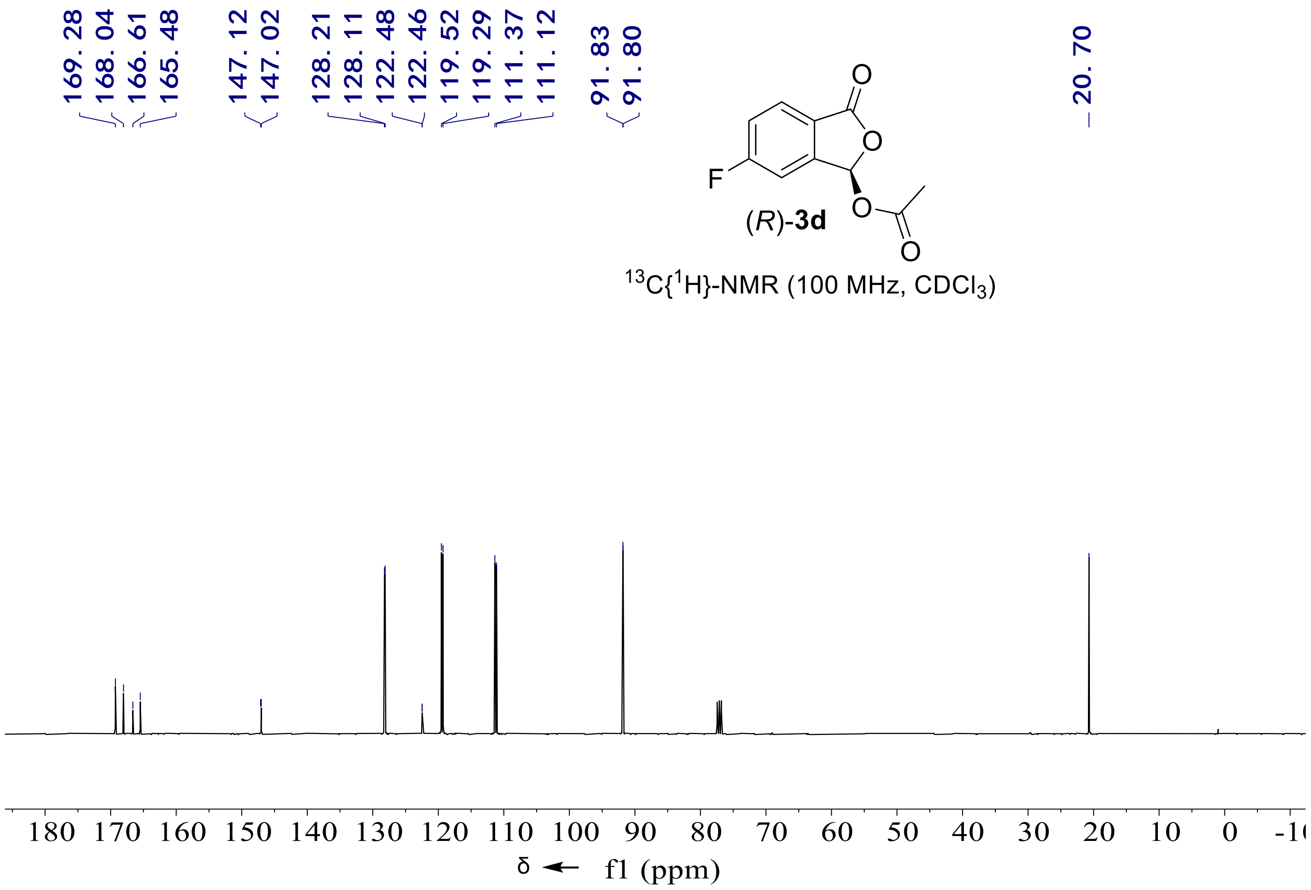

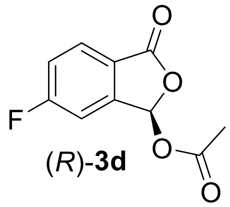

(*R*)-**3d**

^{19}F-NMR (376 MHz, $CDCl_3$)

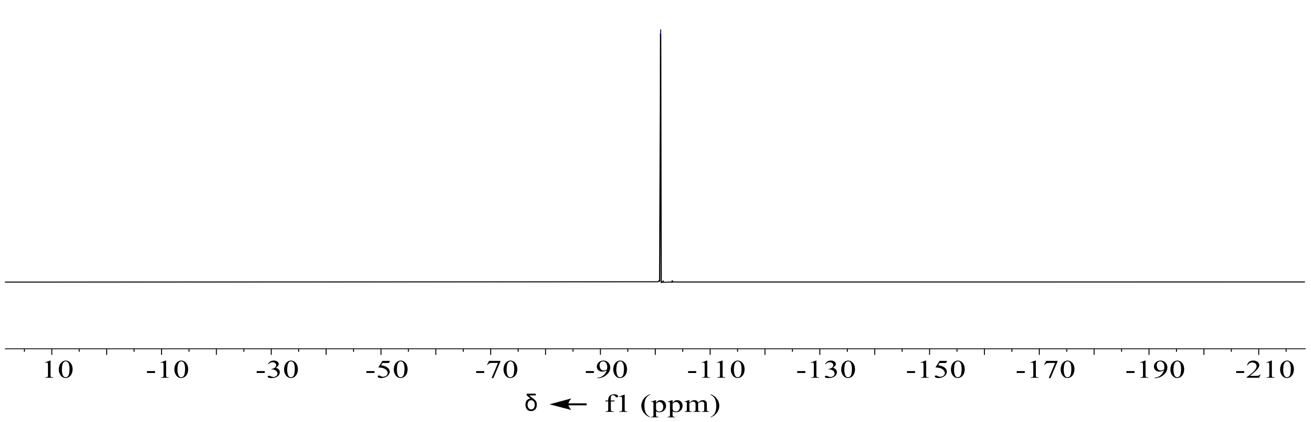

(*R*)-3e: (*R*)-5-chloro-3-oxo-1,3-dihydroisobenzofuran-1-yl acetate.

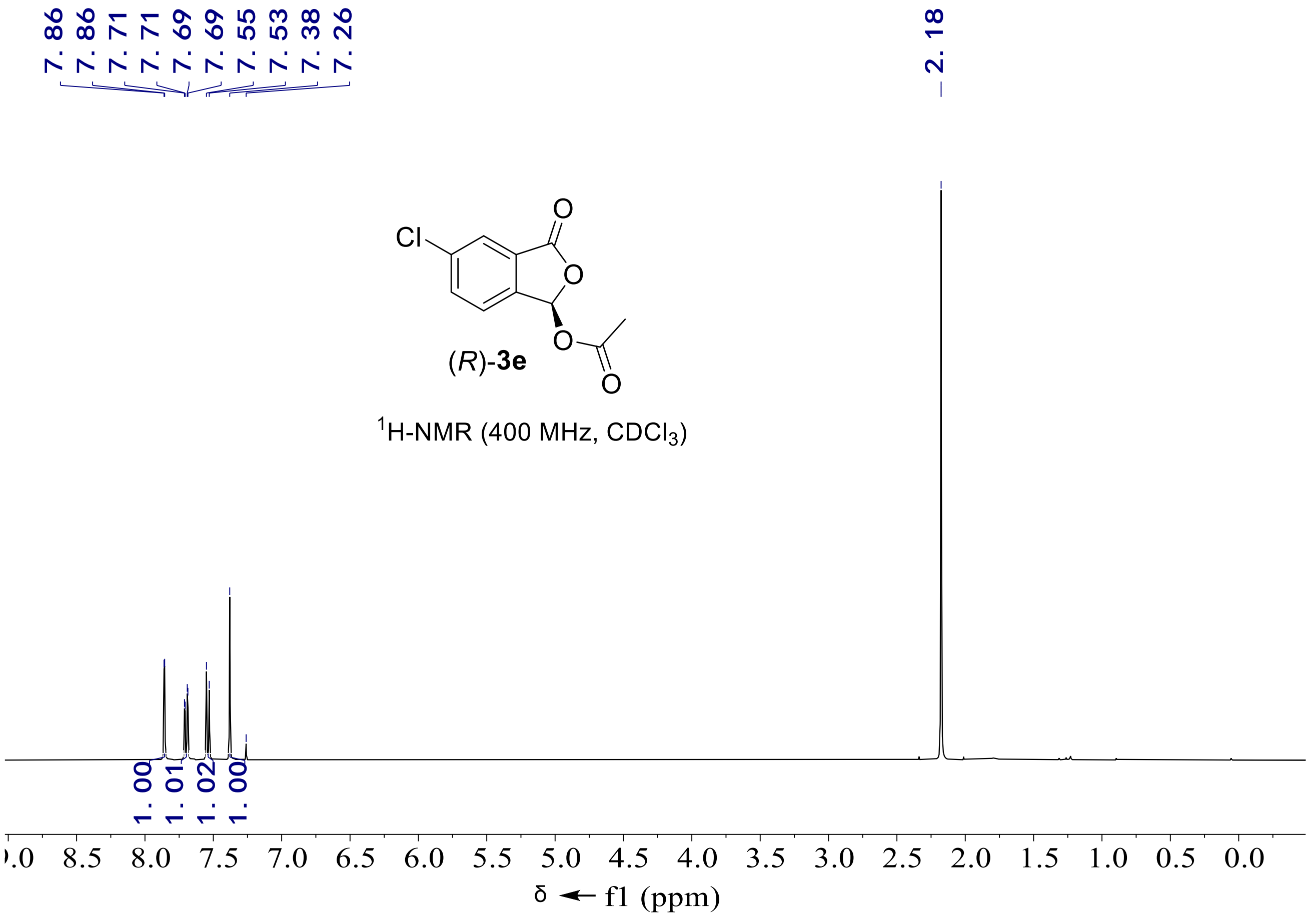

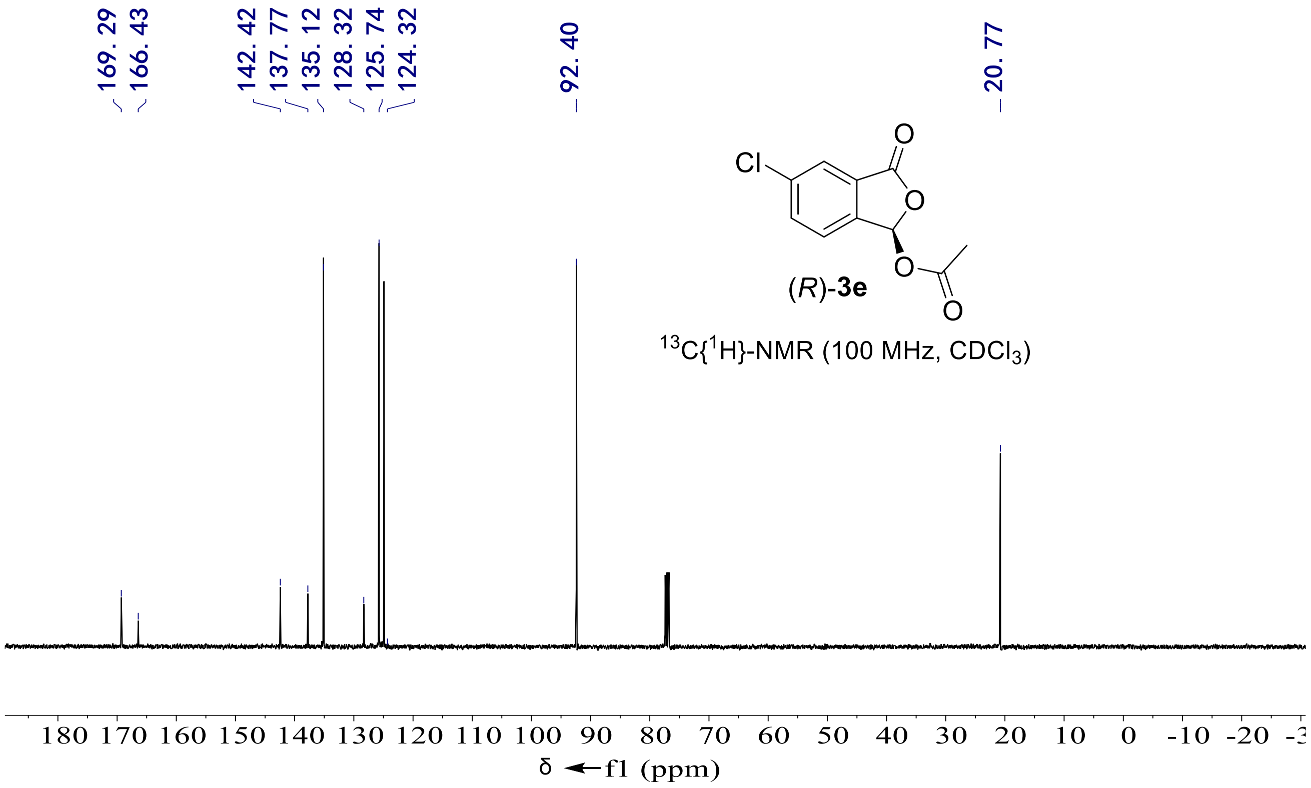

(*R*)-3f: (*R*)-6-chloro-3-oxo-1,3-dihydroisobenzofuran-1-yl acetate.

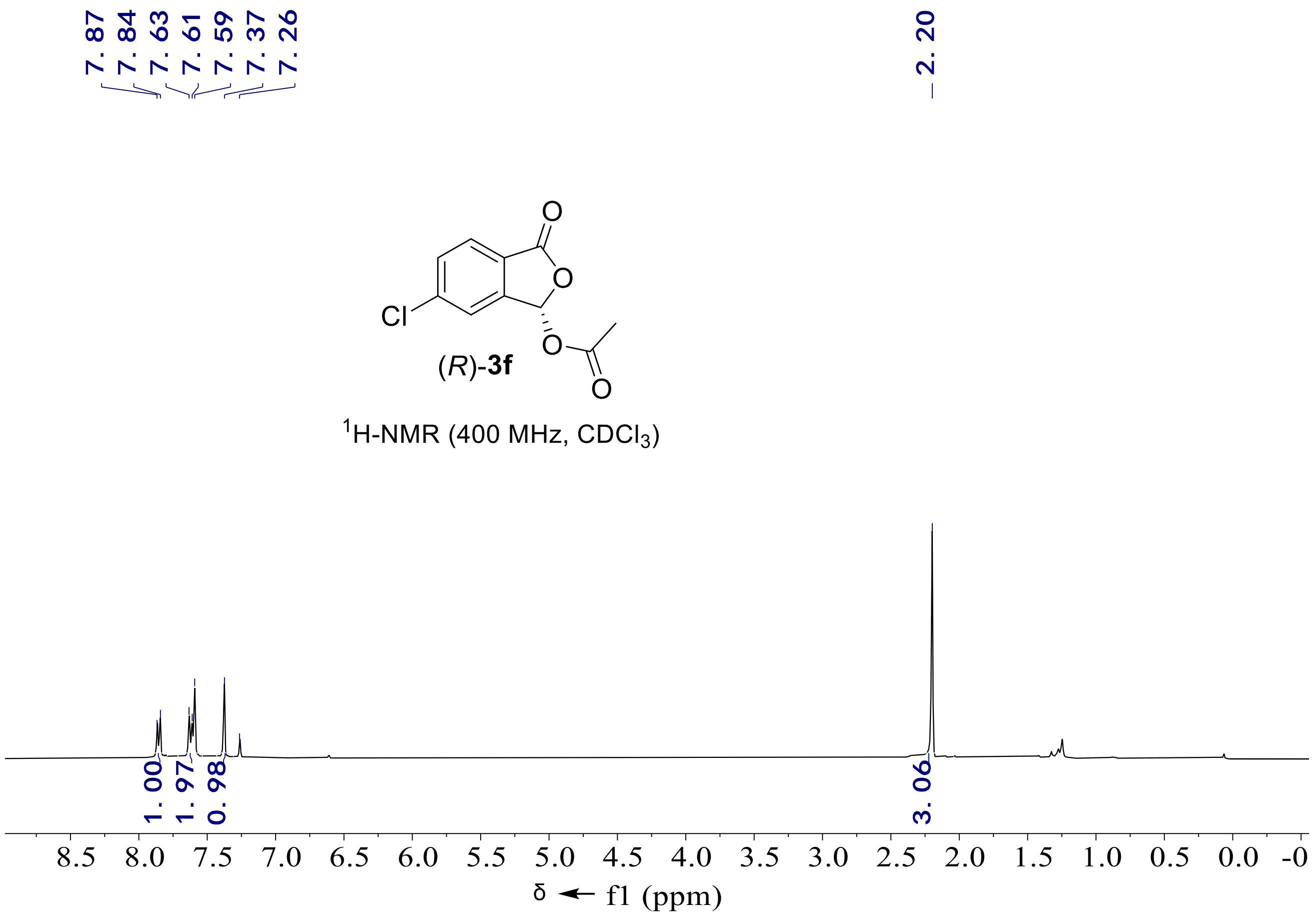

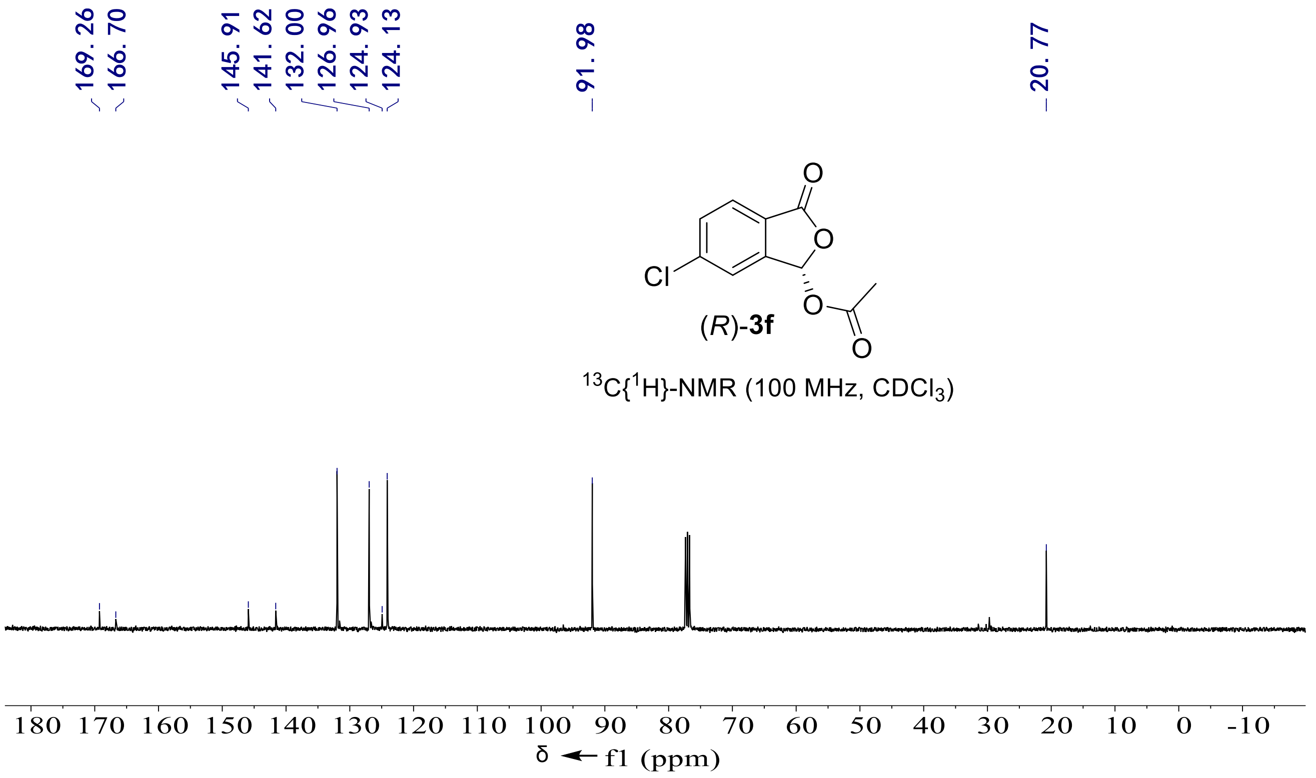

(*R*)-3g: (*R*)-7-chloro-3-oxo-1,3-dihydroisobenzofuran-1-yl acetate.

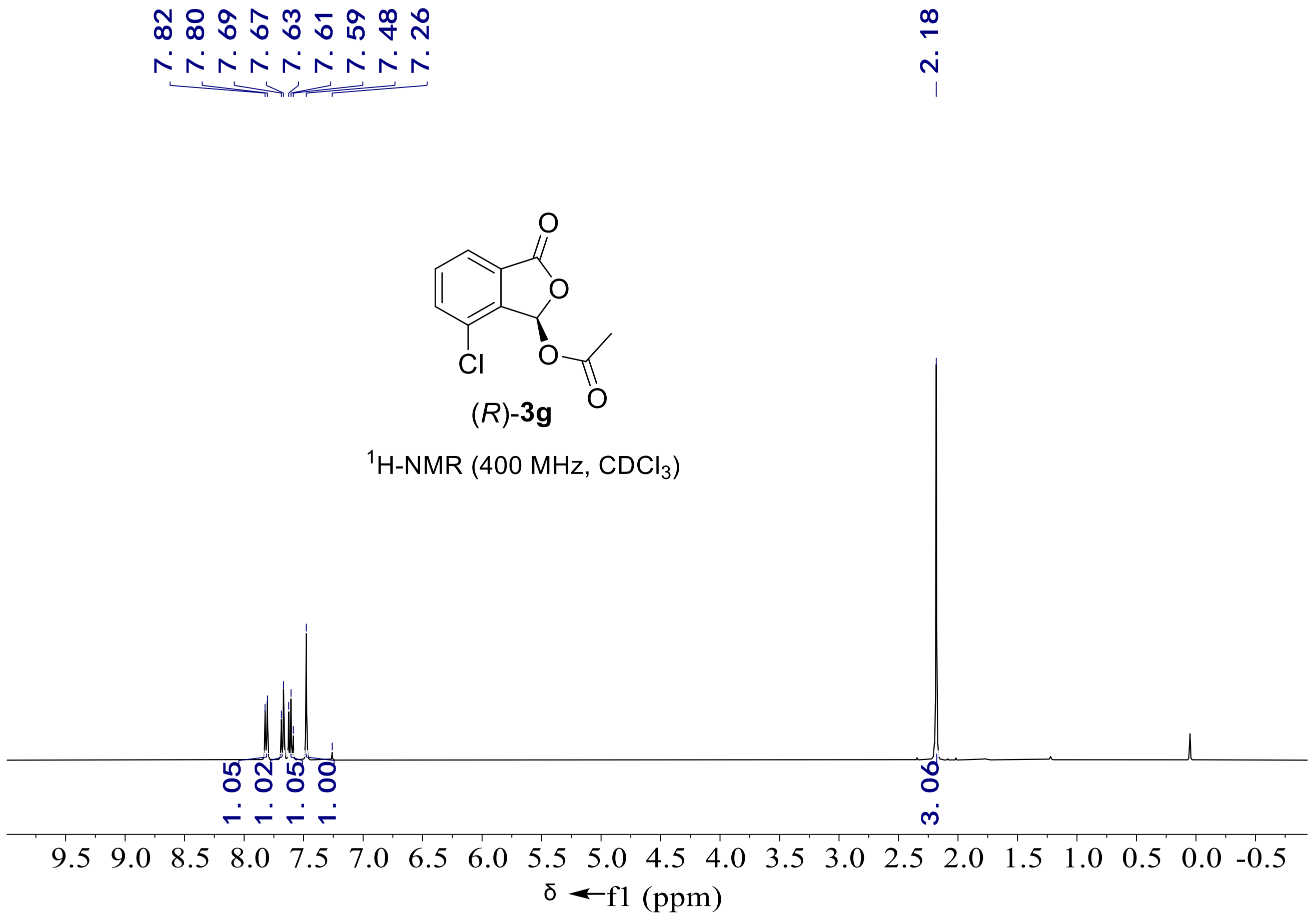

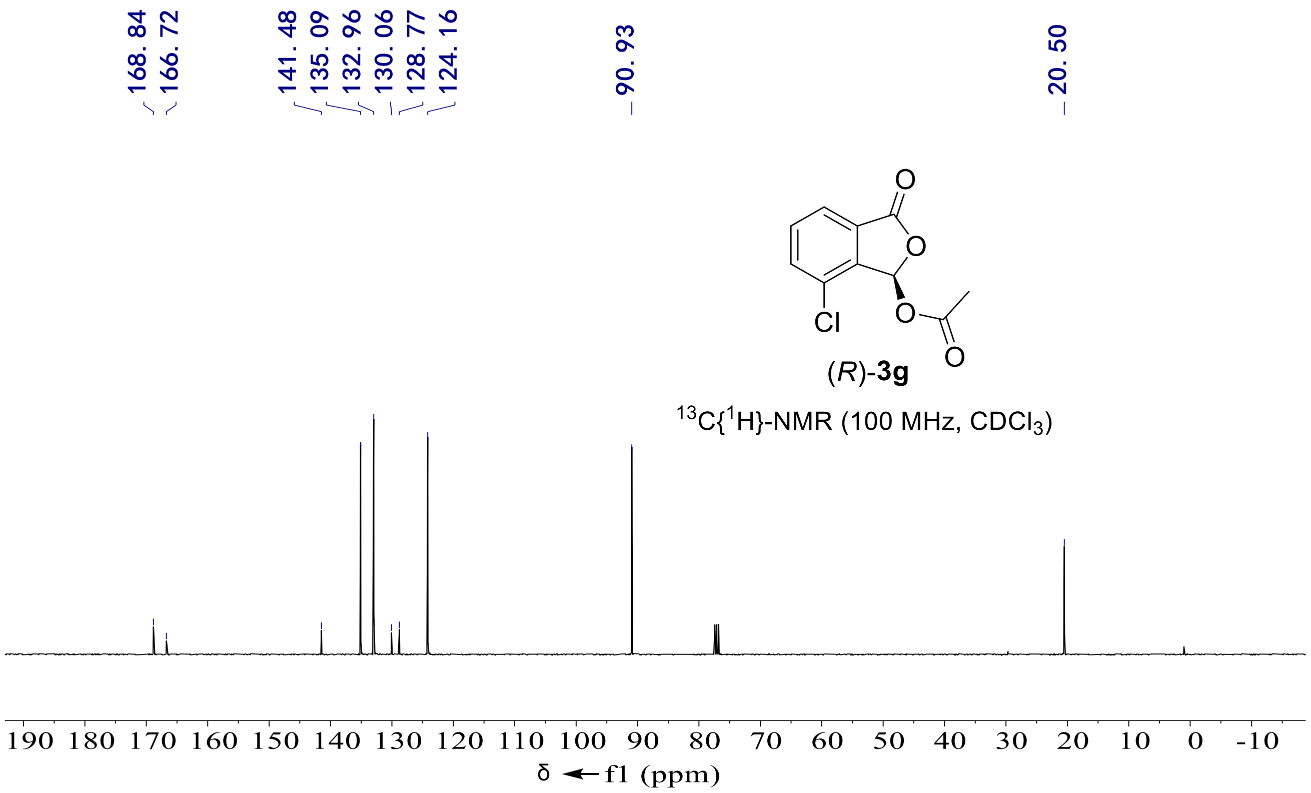

(*R*)-3h: (*R*)-4-bromo-3-oxo-1,3-dihydroisobenzofuran-1-yl acetate.

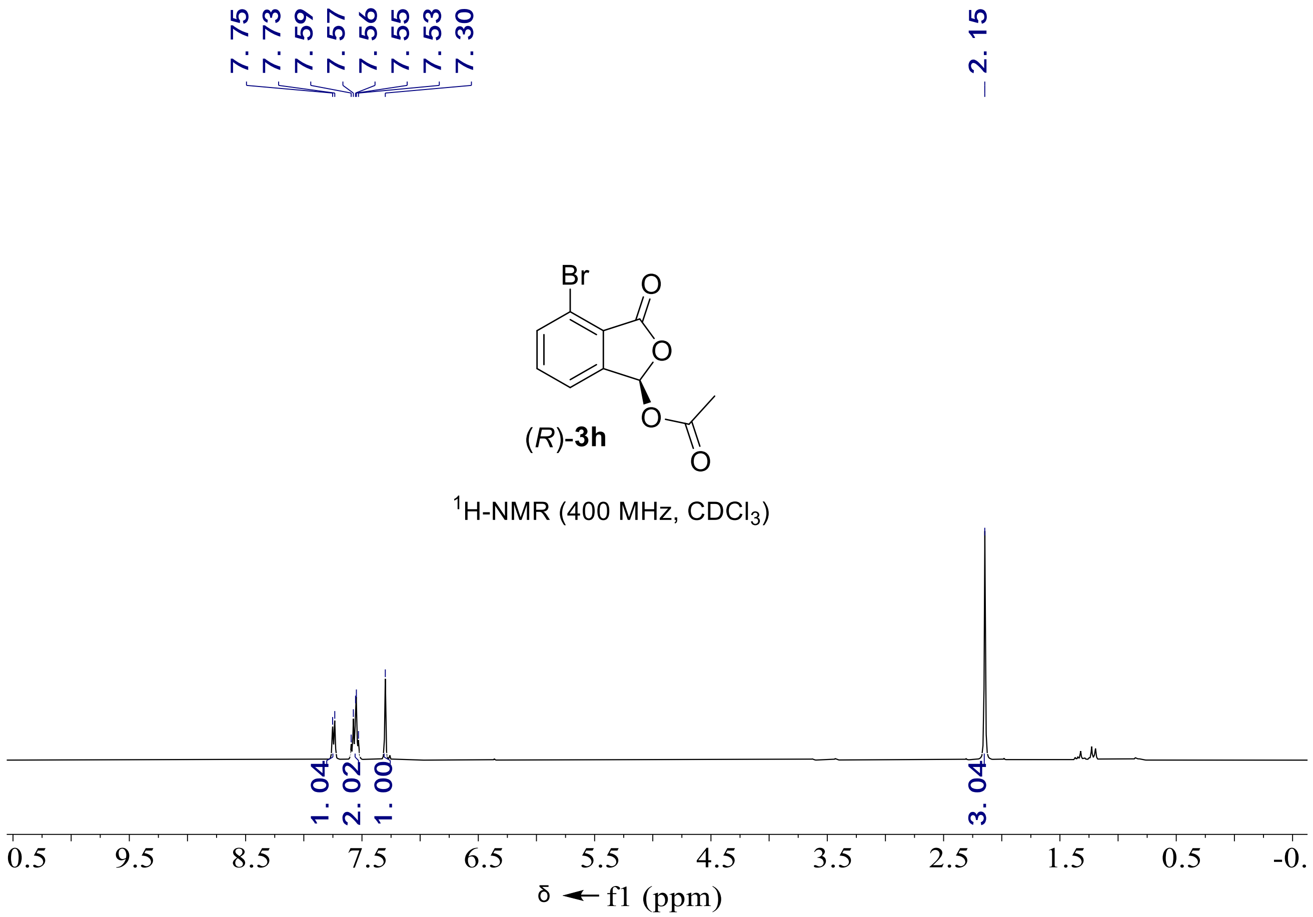

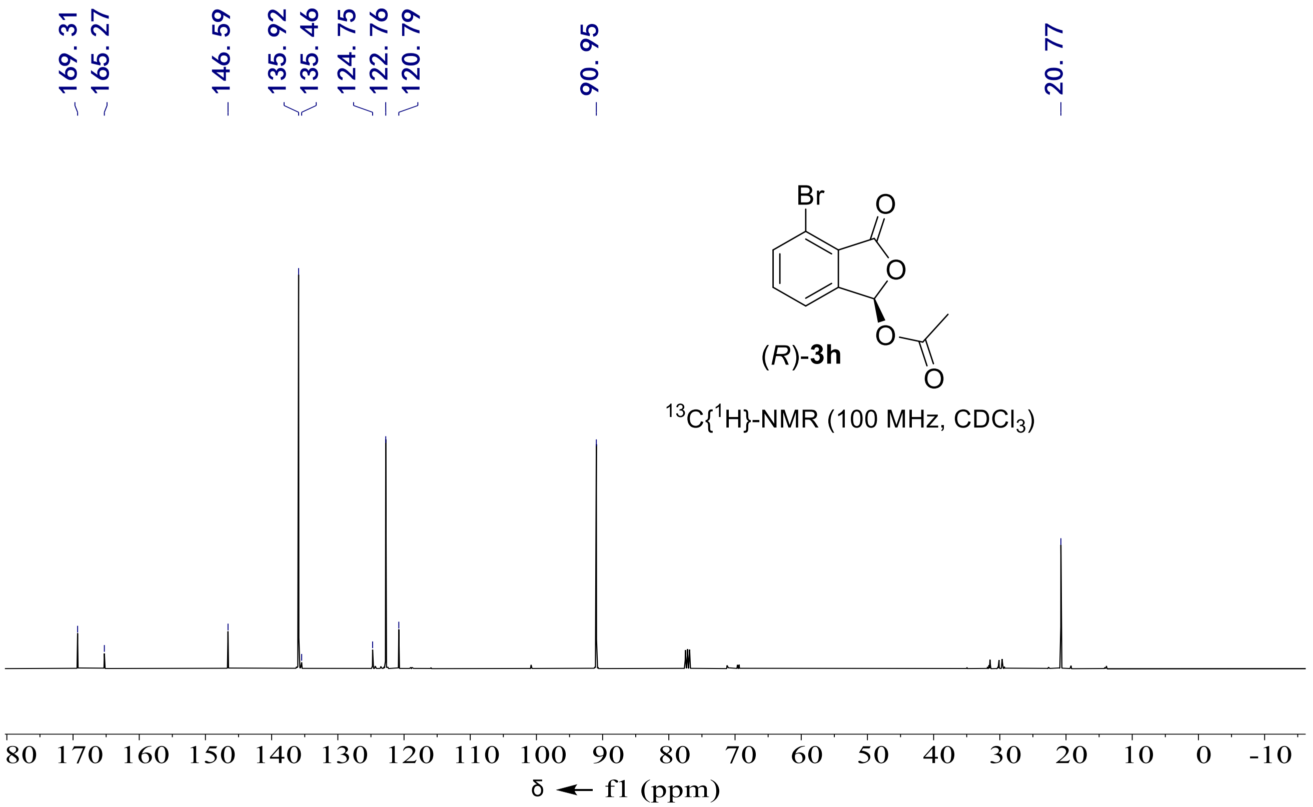

(*R*)-3i: (*R*)-5-bromo-3-oxo-1,3-dihydroisobenzofuran-1-yl acetate.

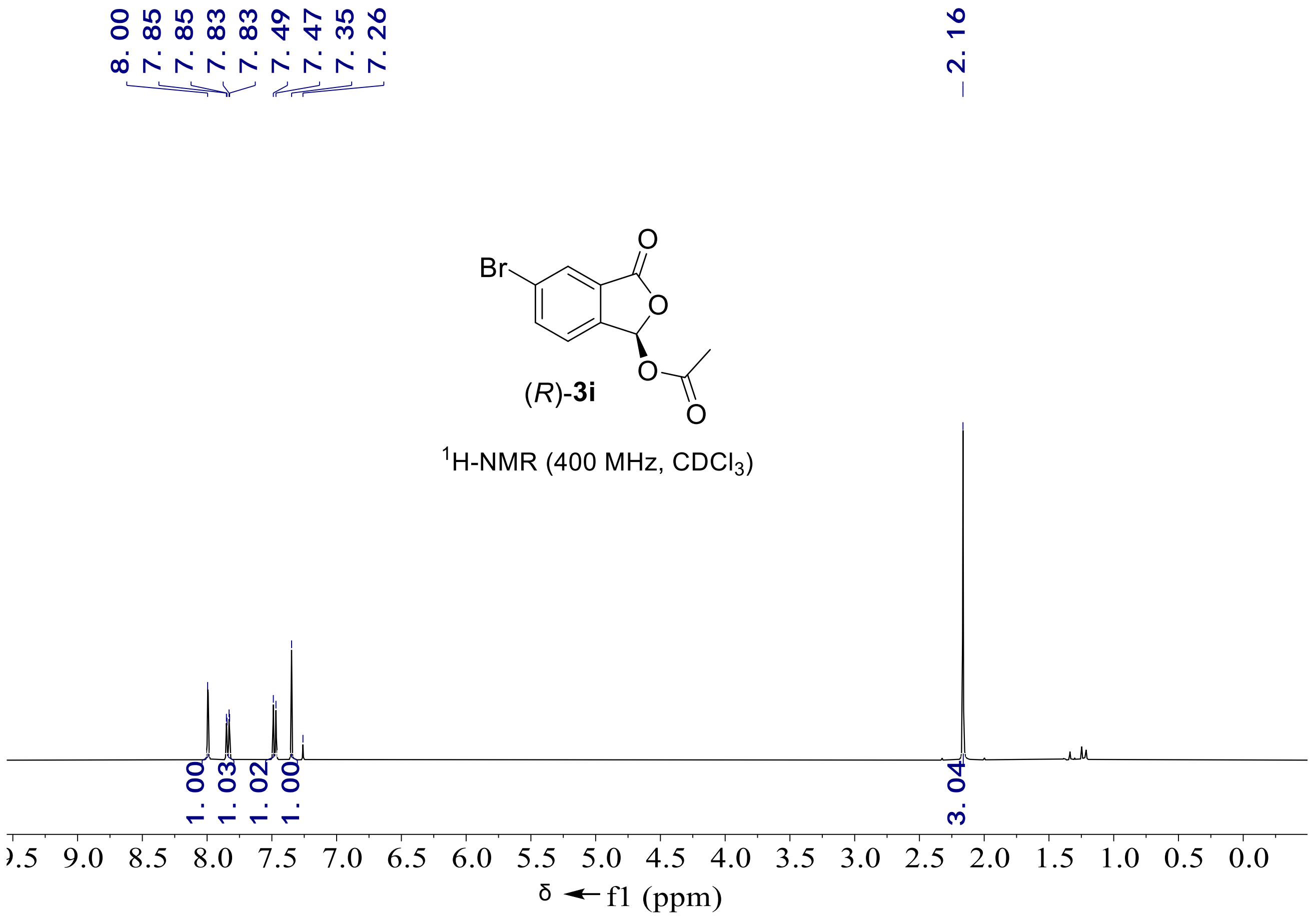

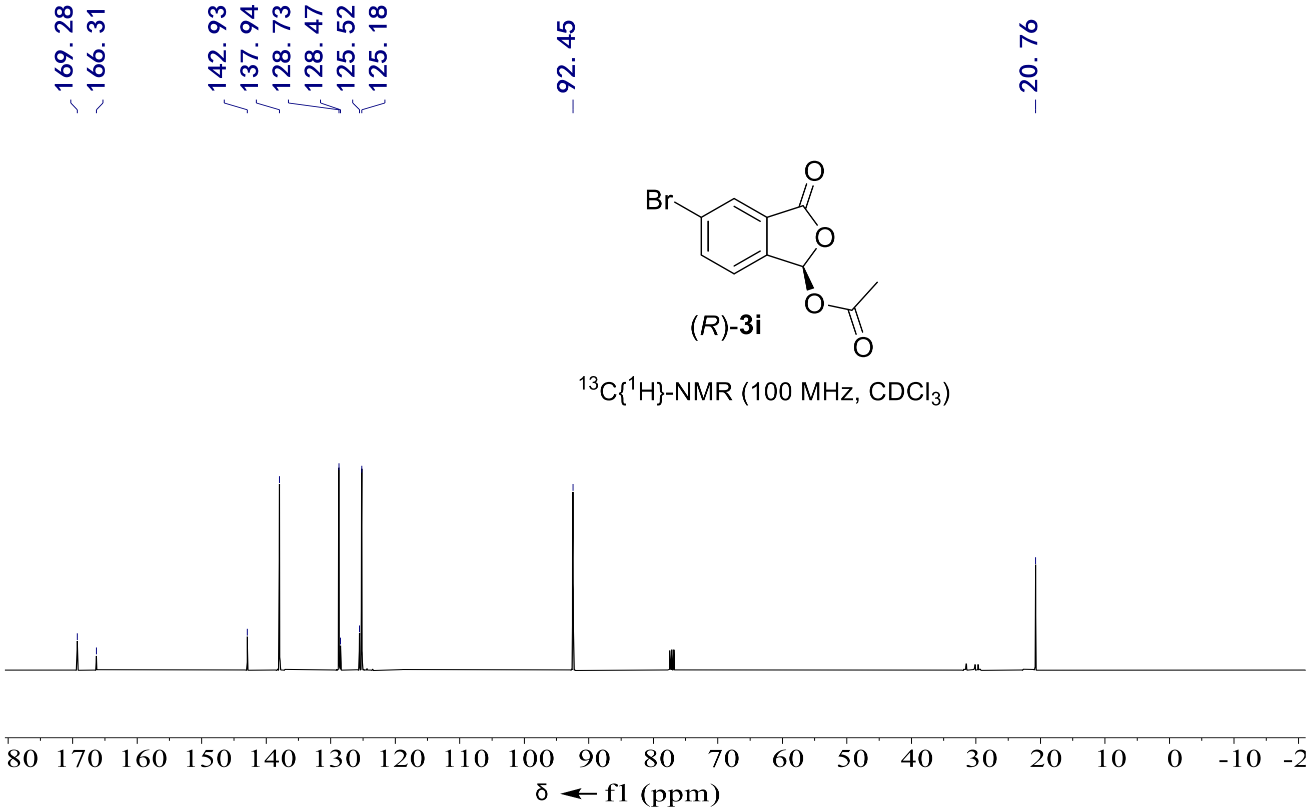

(*R*)-3j: (*R*)-6-bromo-3-oxo-1,3-dihydroisobenzofuran-1-yl acetate.

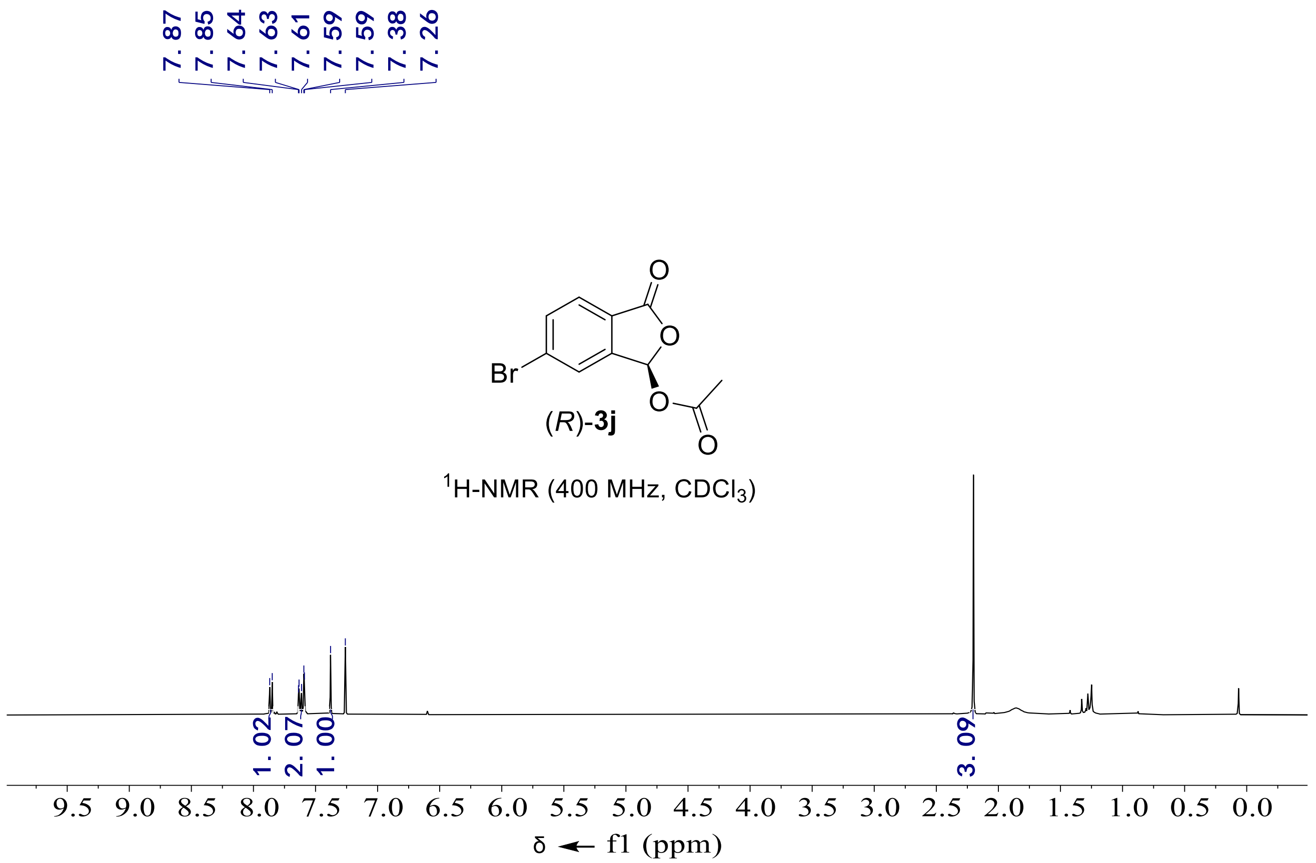

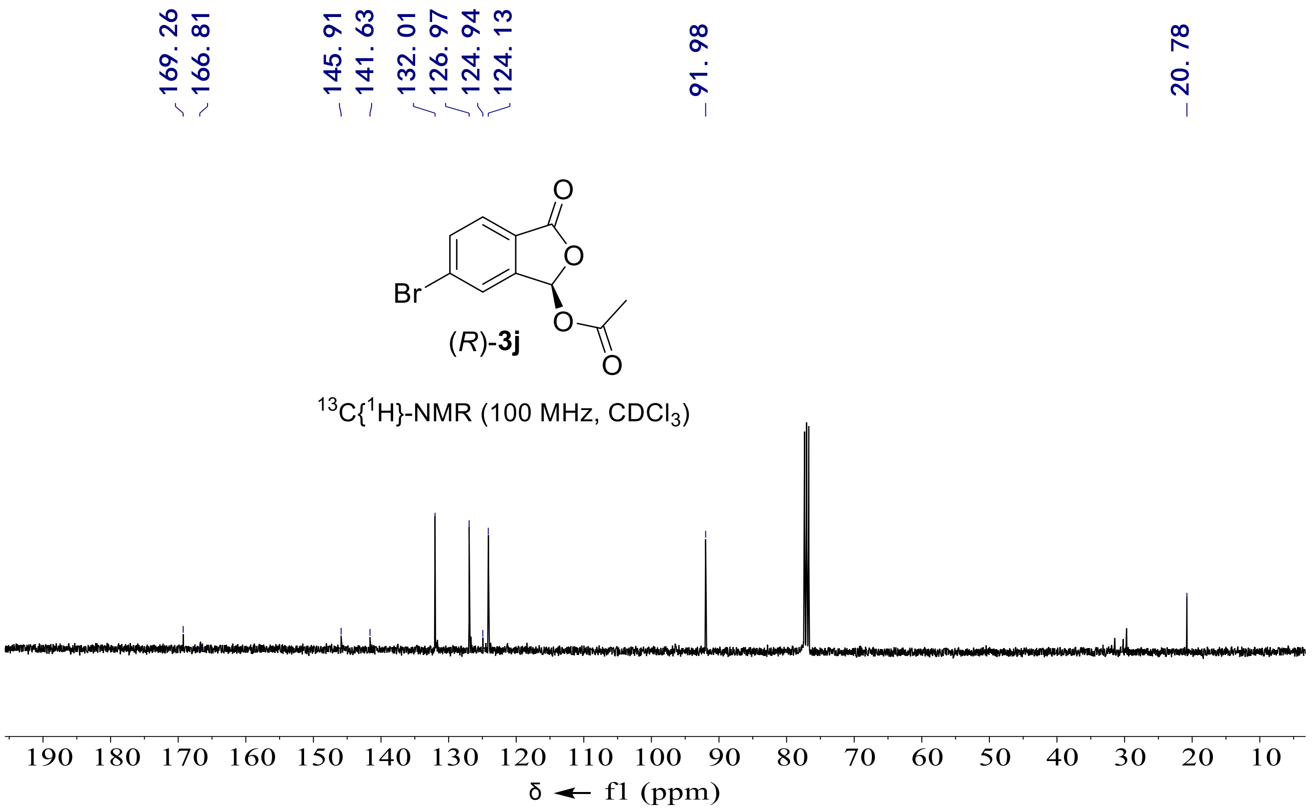

(*R*)-3k: (*R*)-7-bromo-3-oxo-1,3-dihydroisobenzofuran-1-yl acetate.

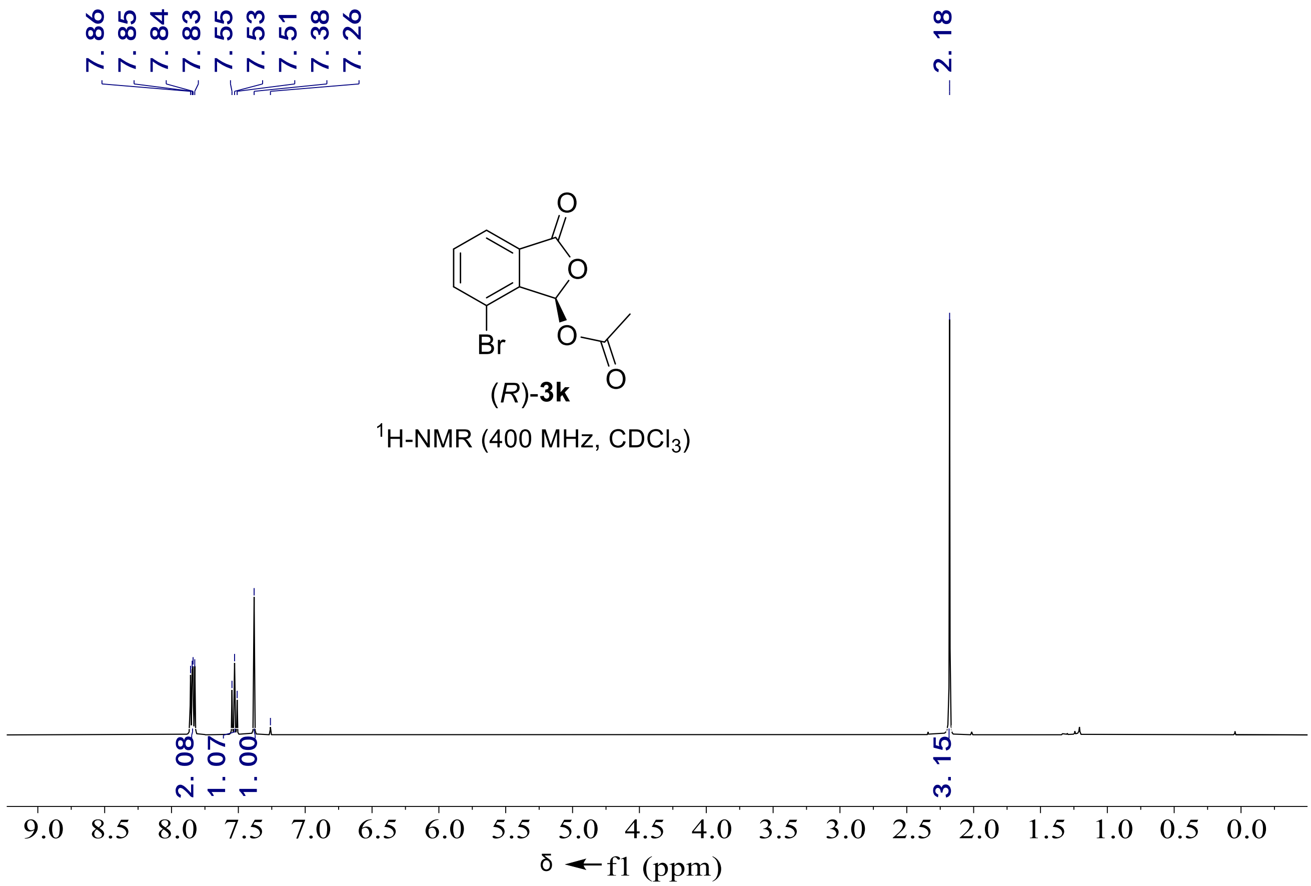

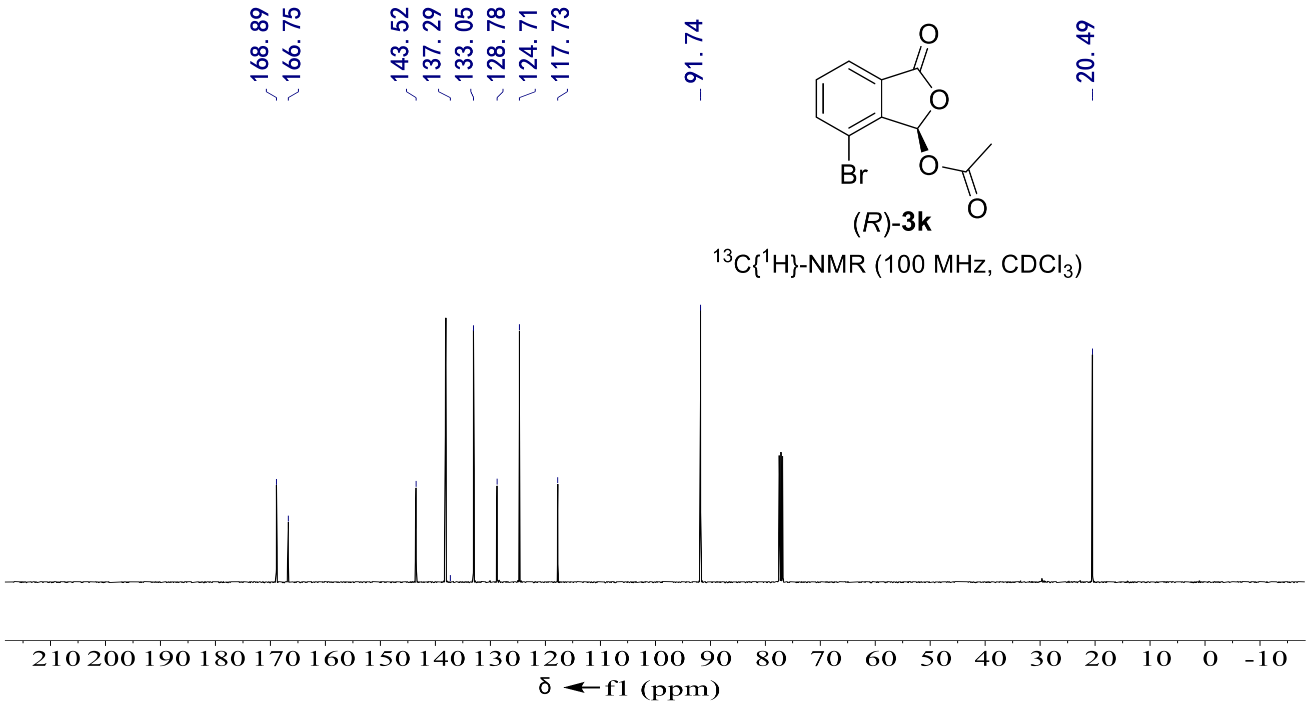

(*R*)-3l: (*R*)-5-iodo-3-oxo-1,3-dihydroisobenzofuran-1-yl acetate.

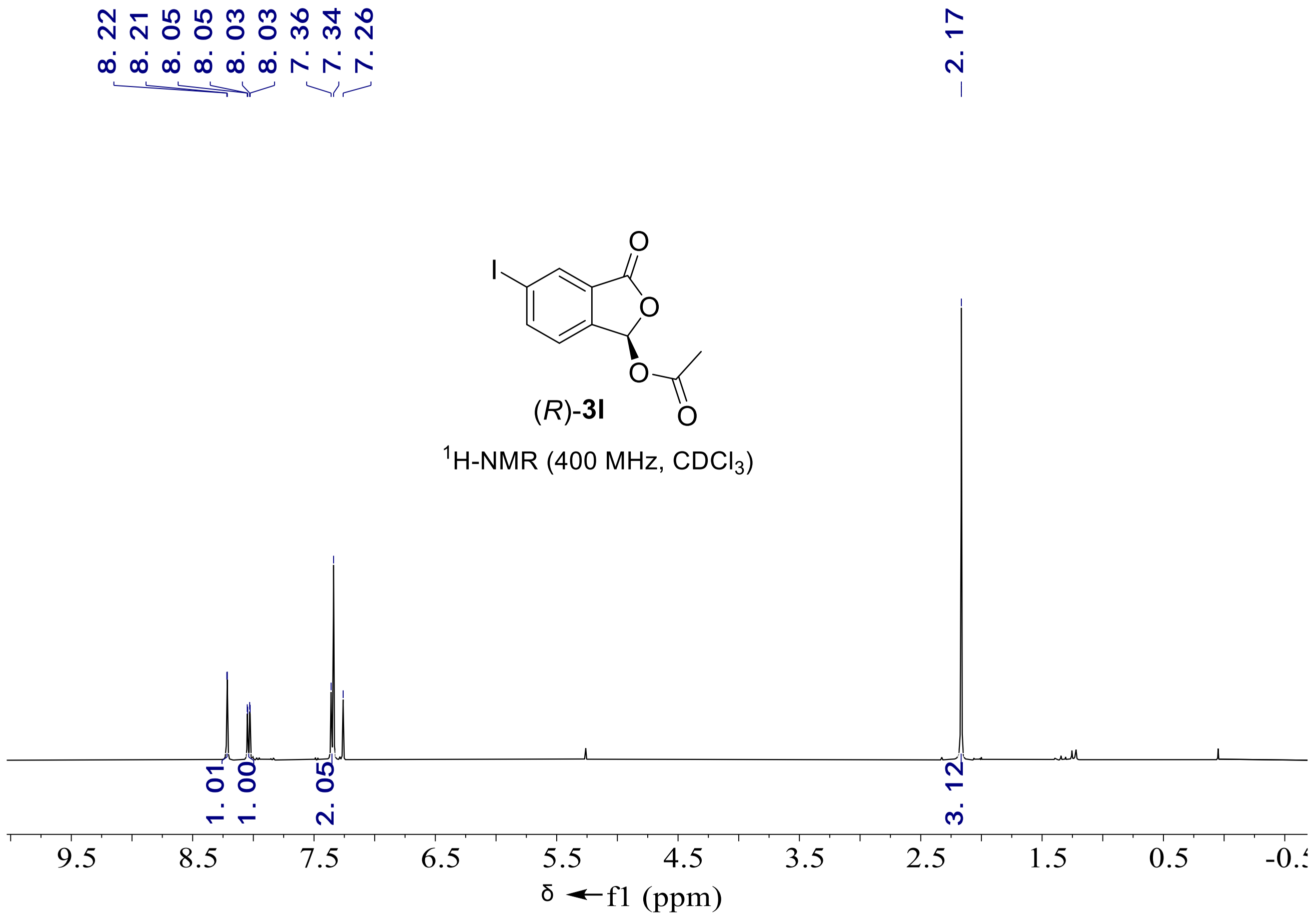

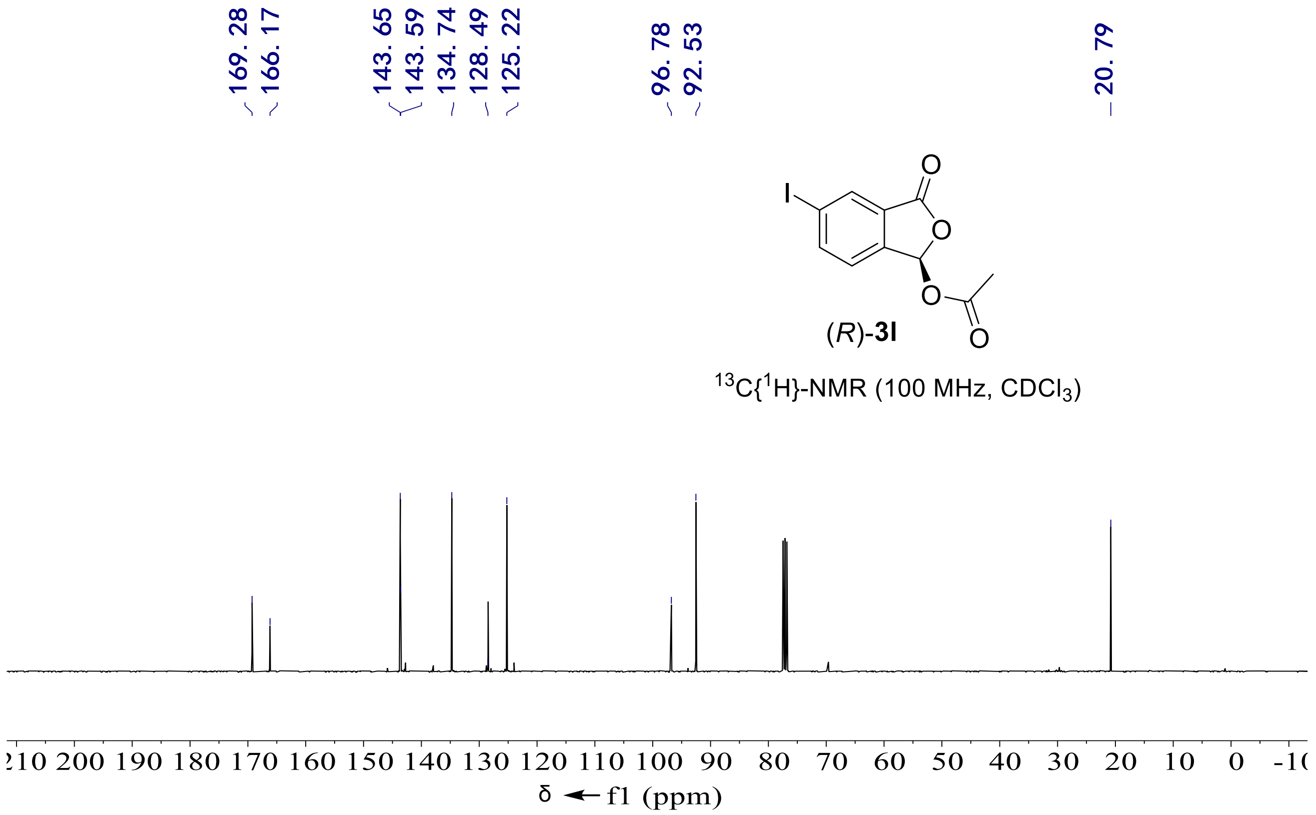

(*R*)-3m: (*R*)-5-cyano-3-oxo-1,3-dihydroisobenzofuran-1-yl acetate.

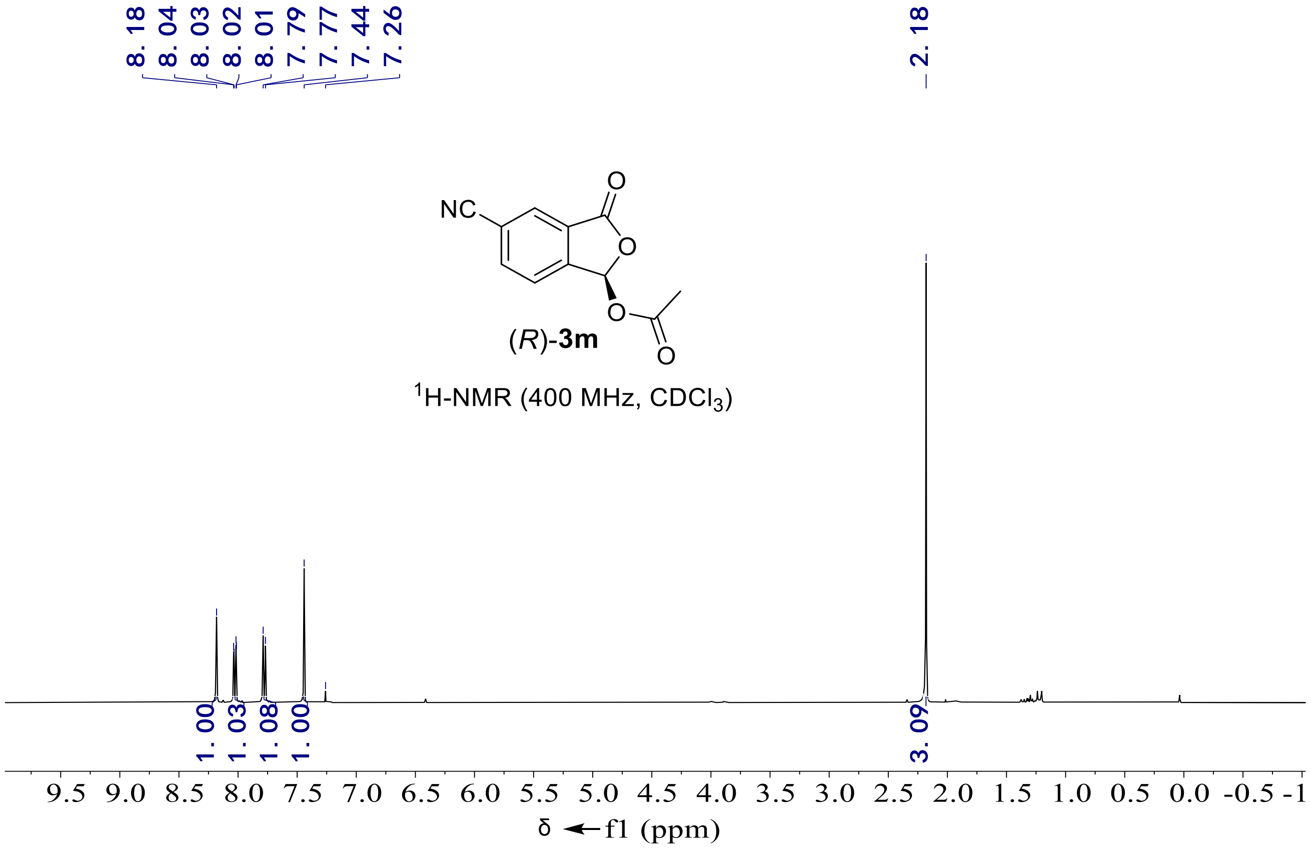

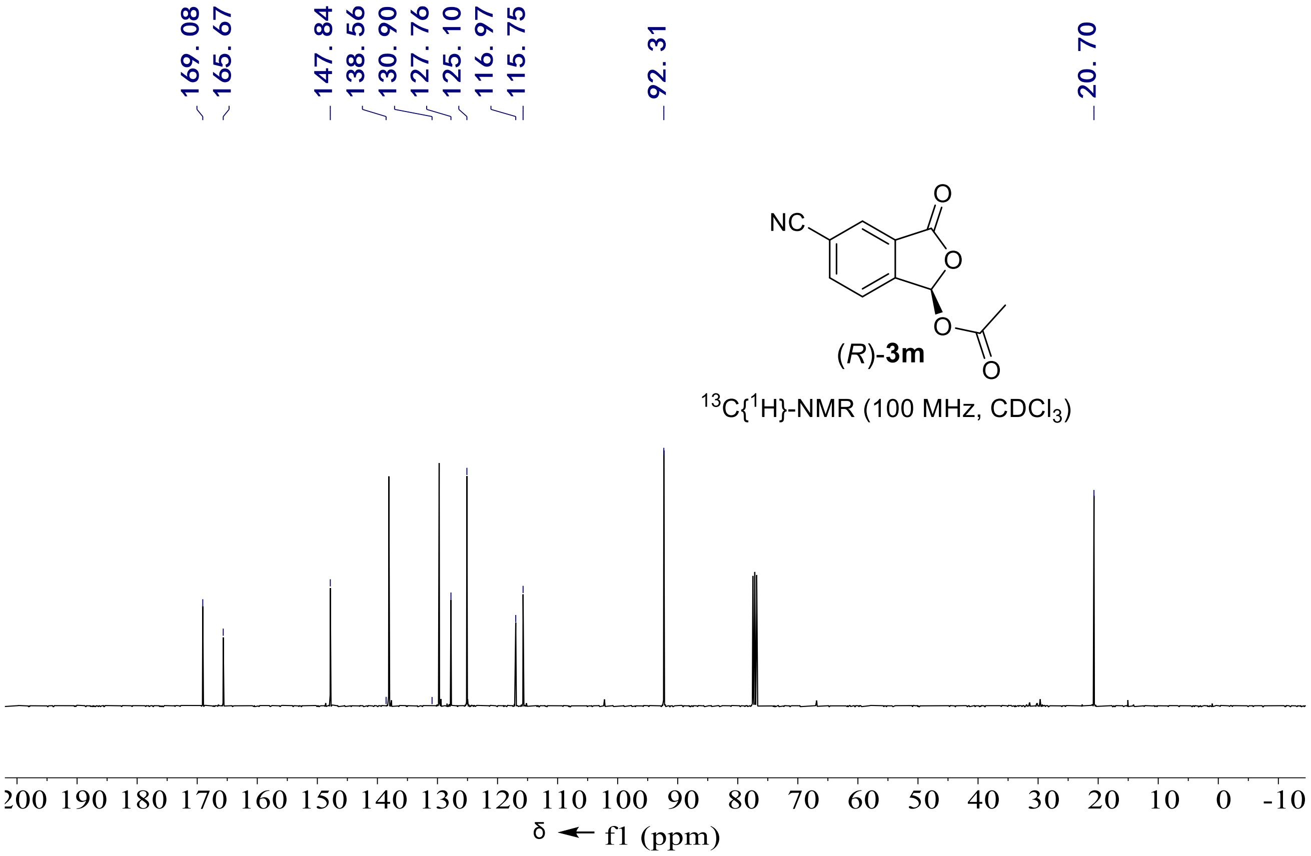

(R)-3n: (*R*)-5-nitro-3-oxo-1,3-dihydroisobenzofuran-1-yl acetate.

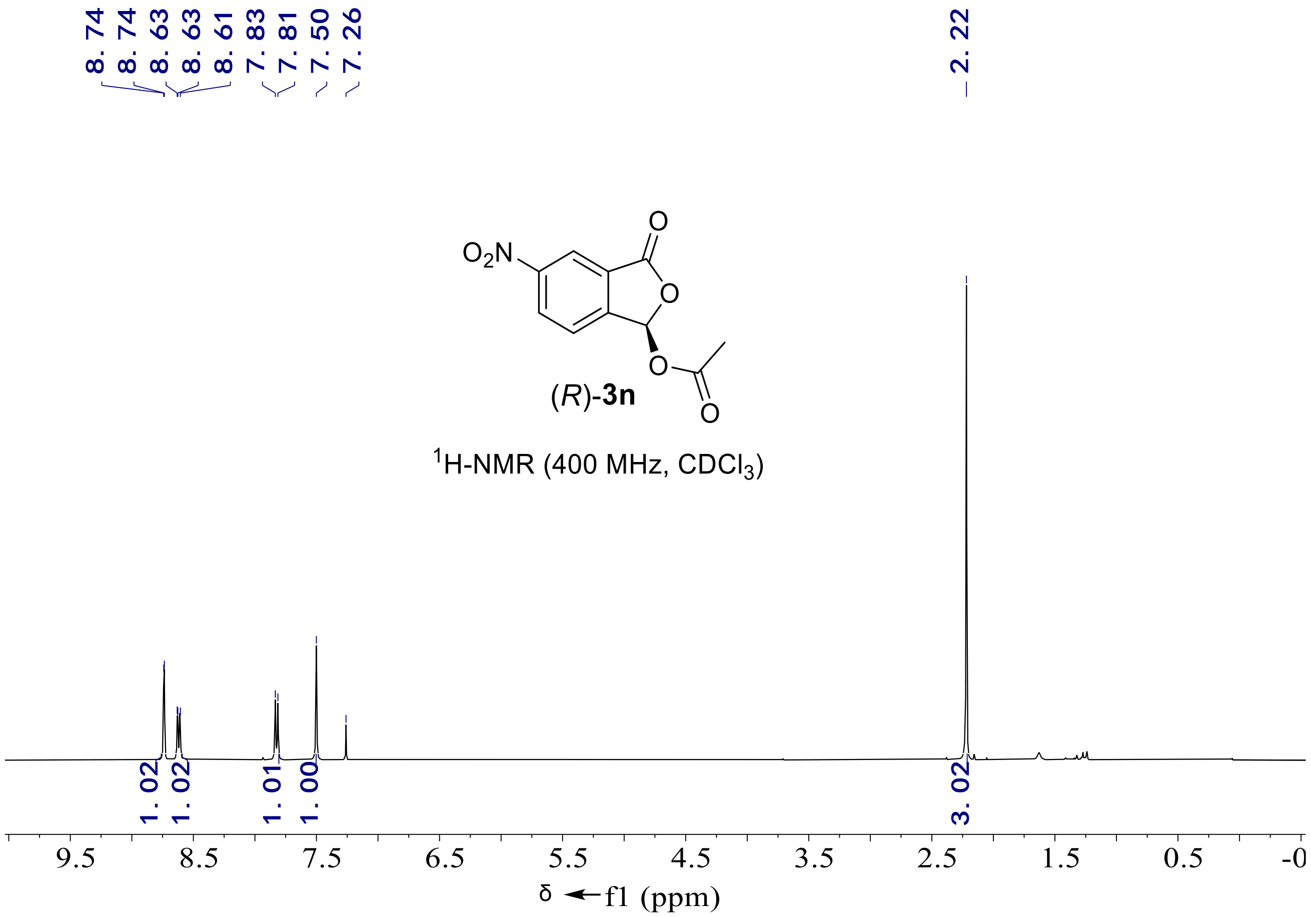

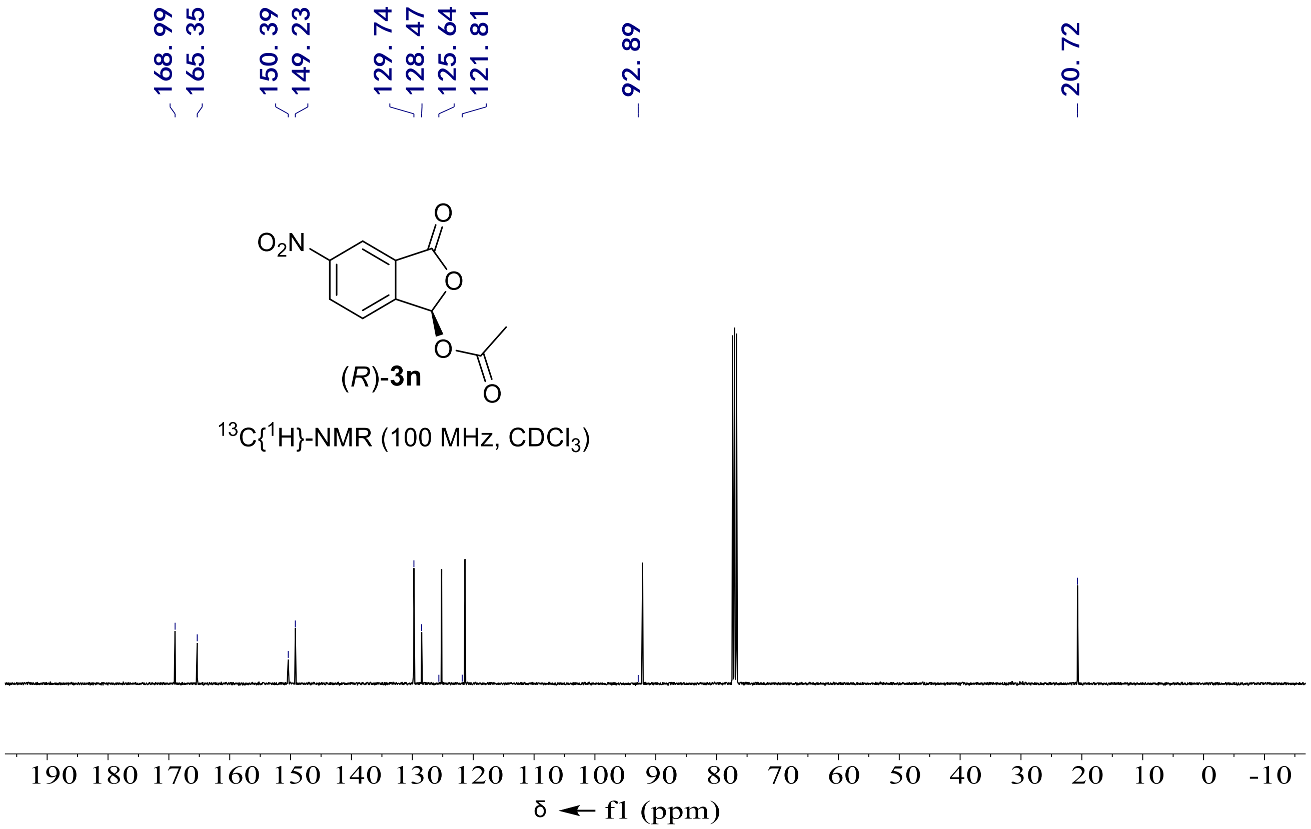

(*R*)-3o: (*R*)-5-methyl-3-oxo-1,3-dihydroisobenzofuran-1-yl acetate.

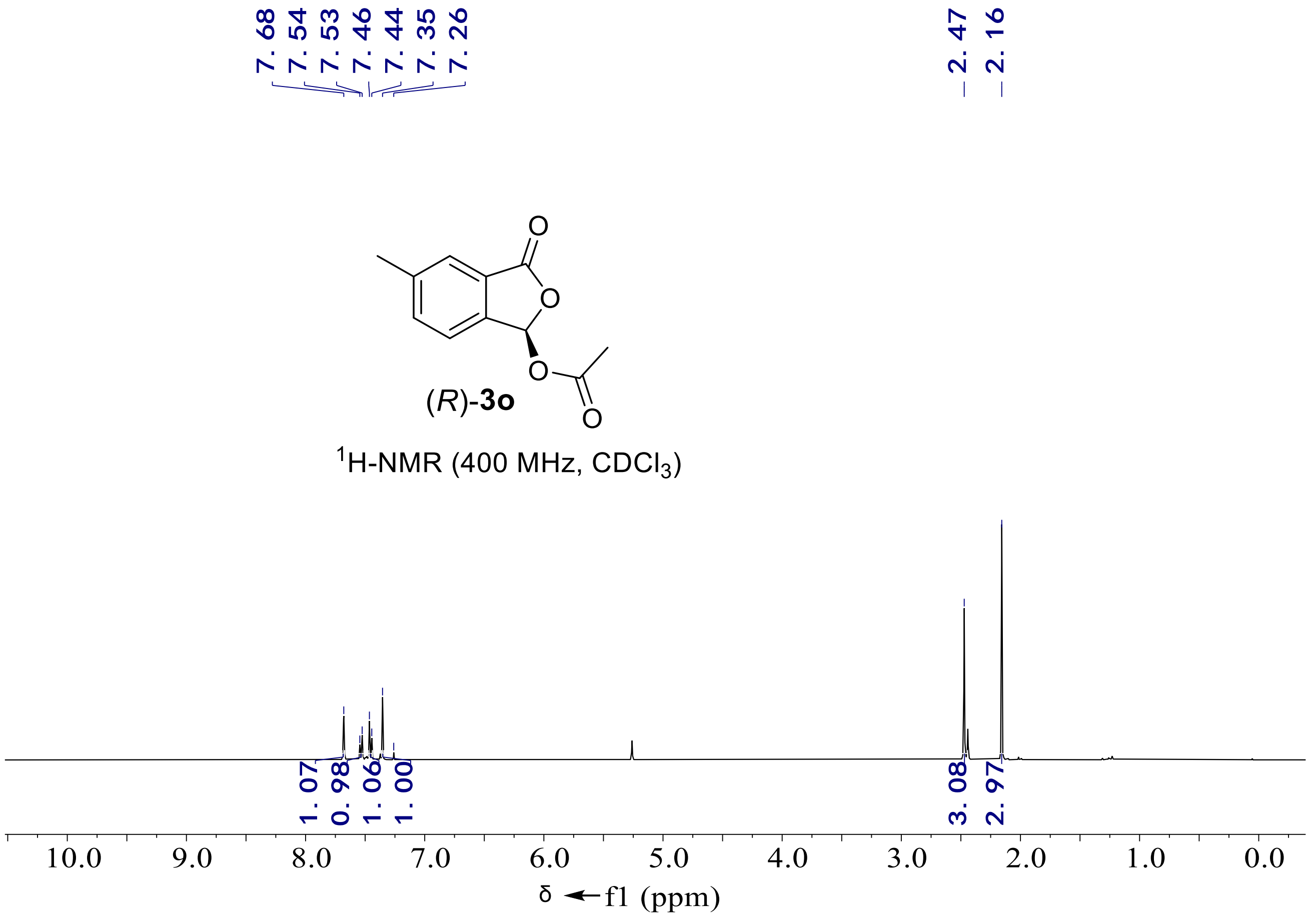

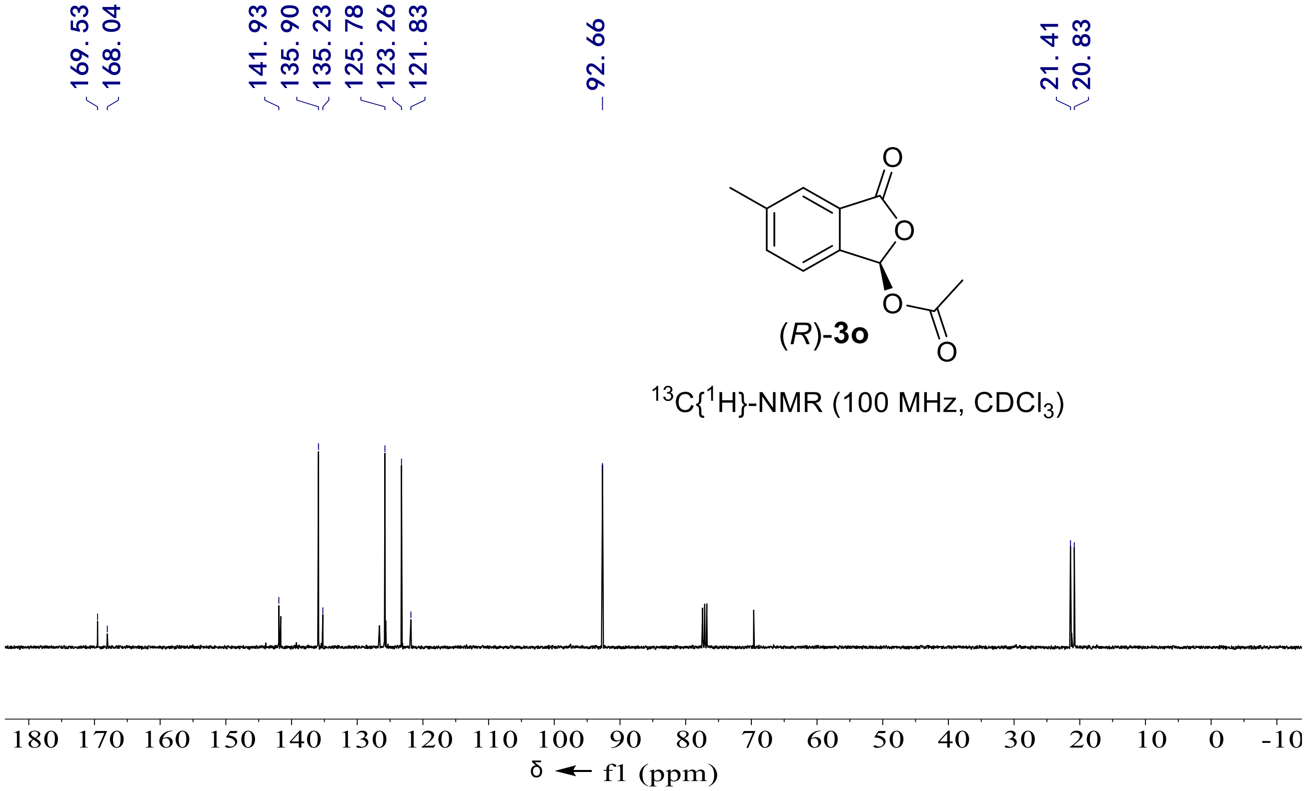

(*R*)-3p: (*R*)-6-methyl-3-oxo-1,3-dihydroisobenzofuran-1-yl acetate.

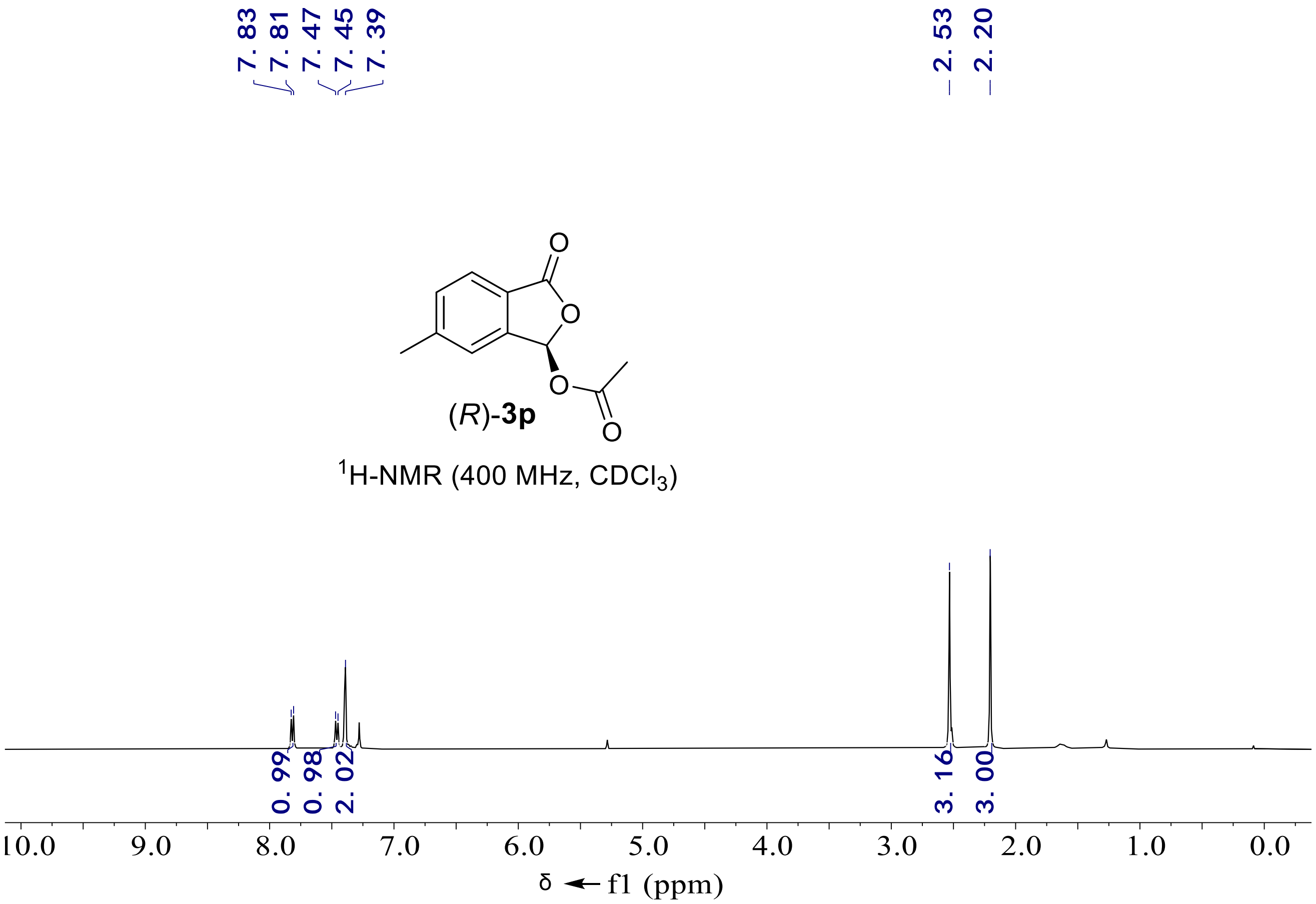

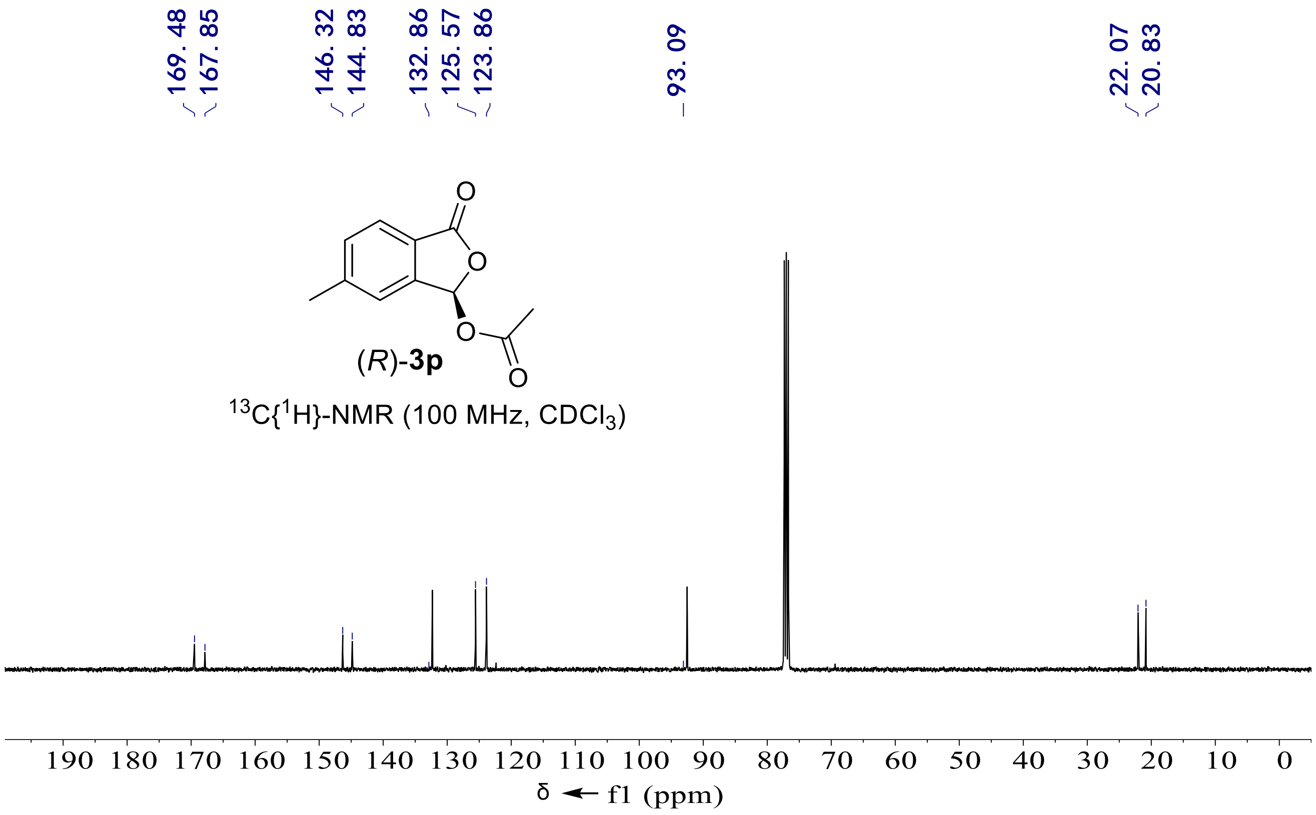

(*R*)-3q: (*R*)-6-methoxy-3-oxo-1,3-dihydroisobenzofuran-1-yl acetate.

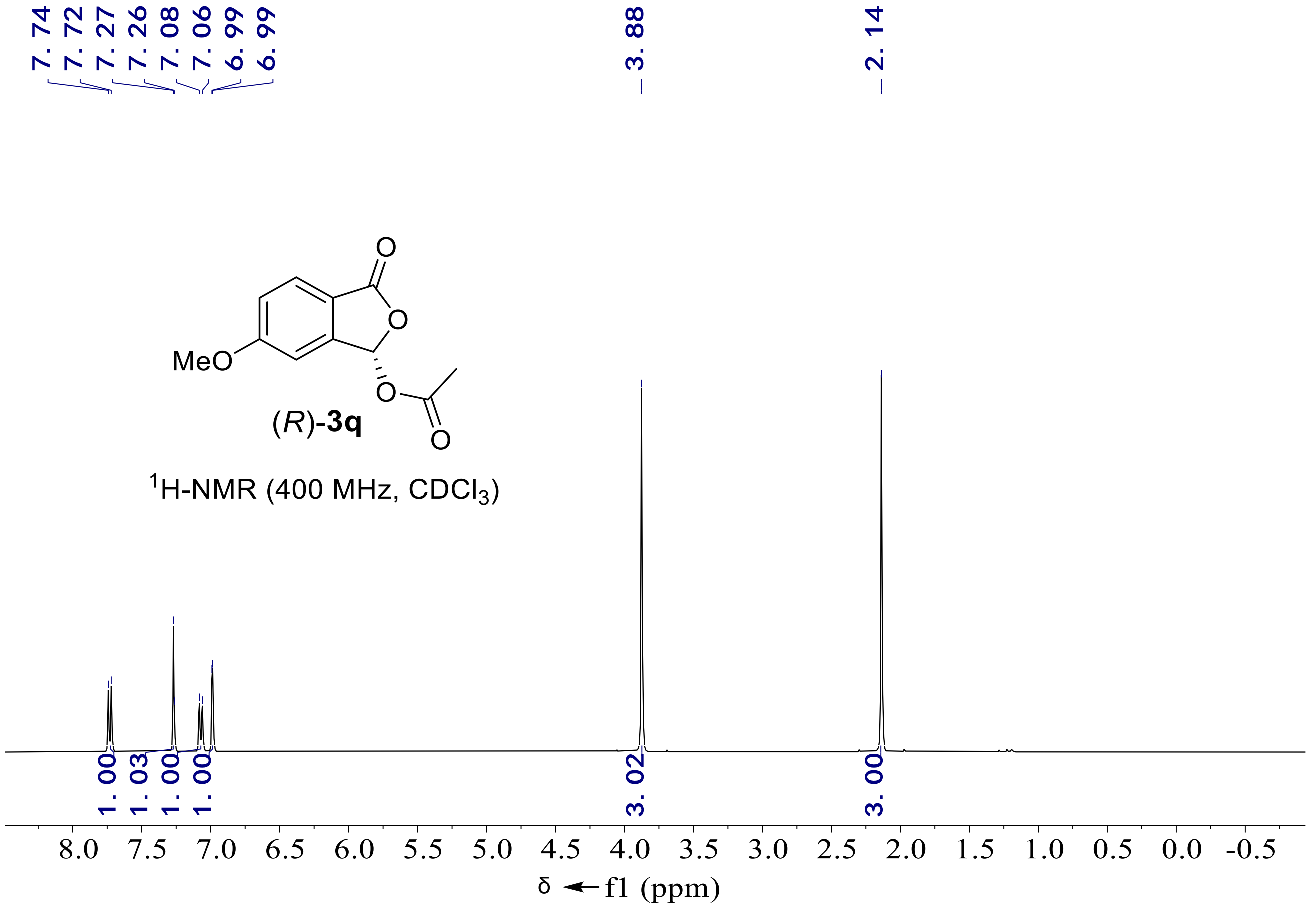

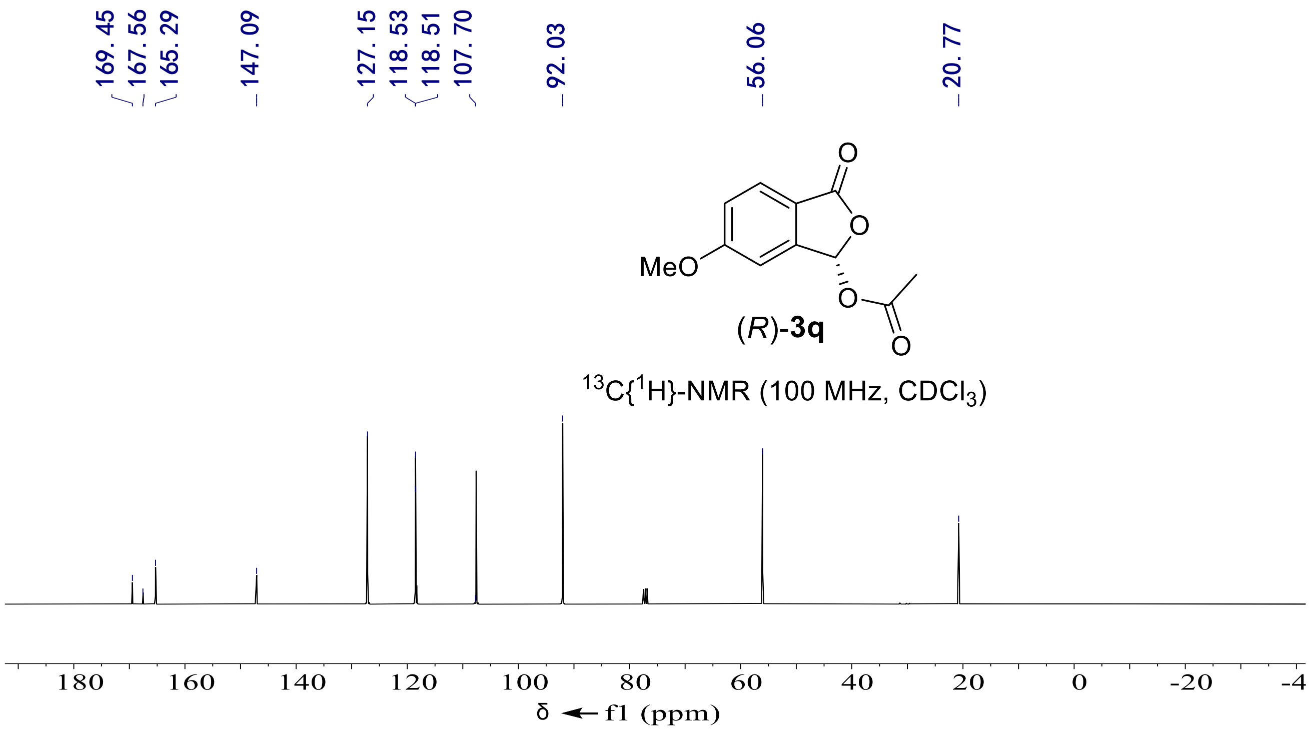

(*R*)-3r: (*R*)-6-methoxy-3-oxo-1,3-dihydroisobenzofuran-1-yl acetate.

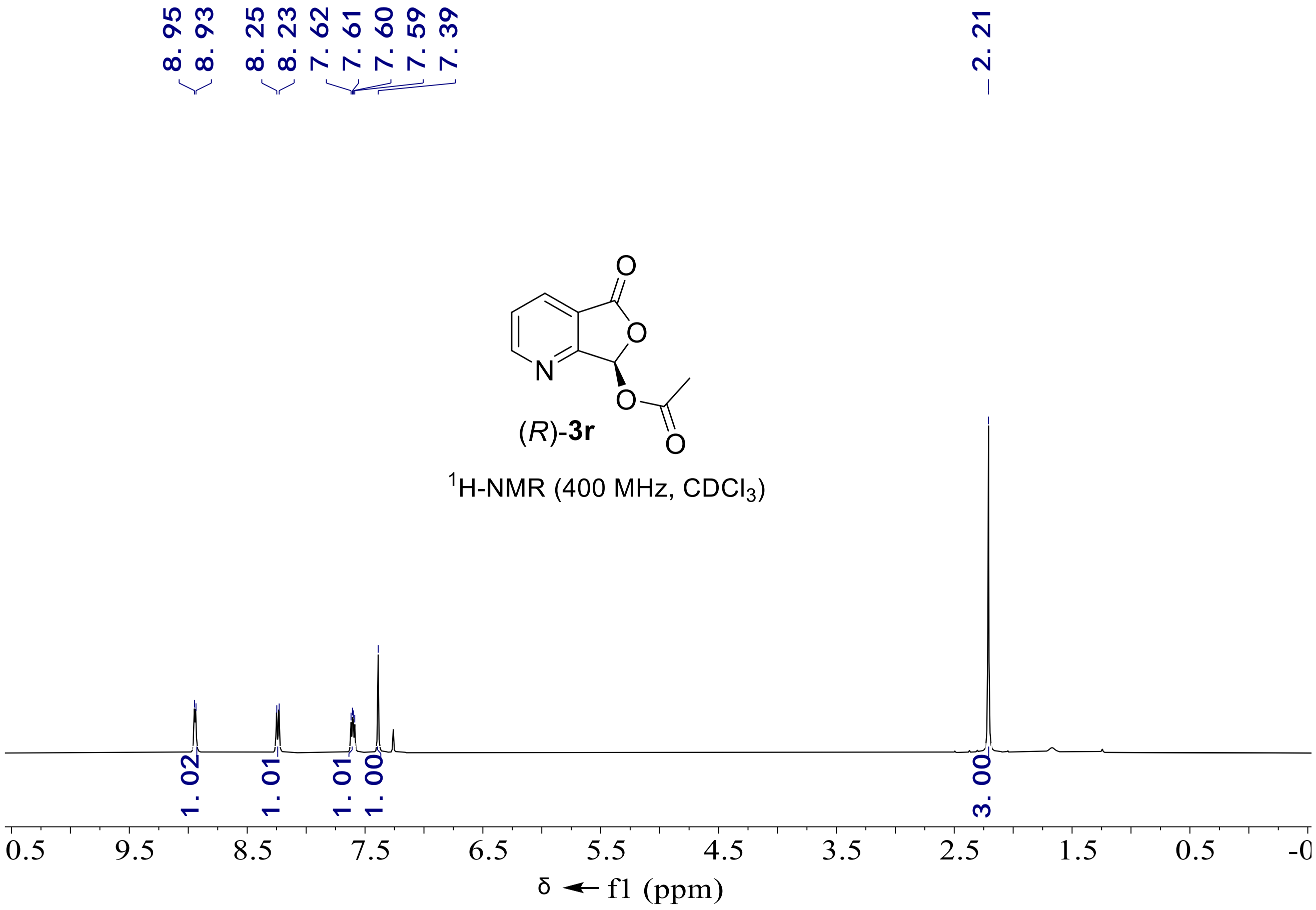

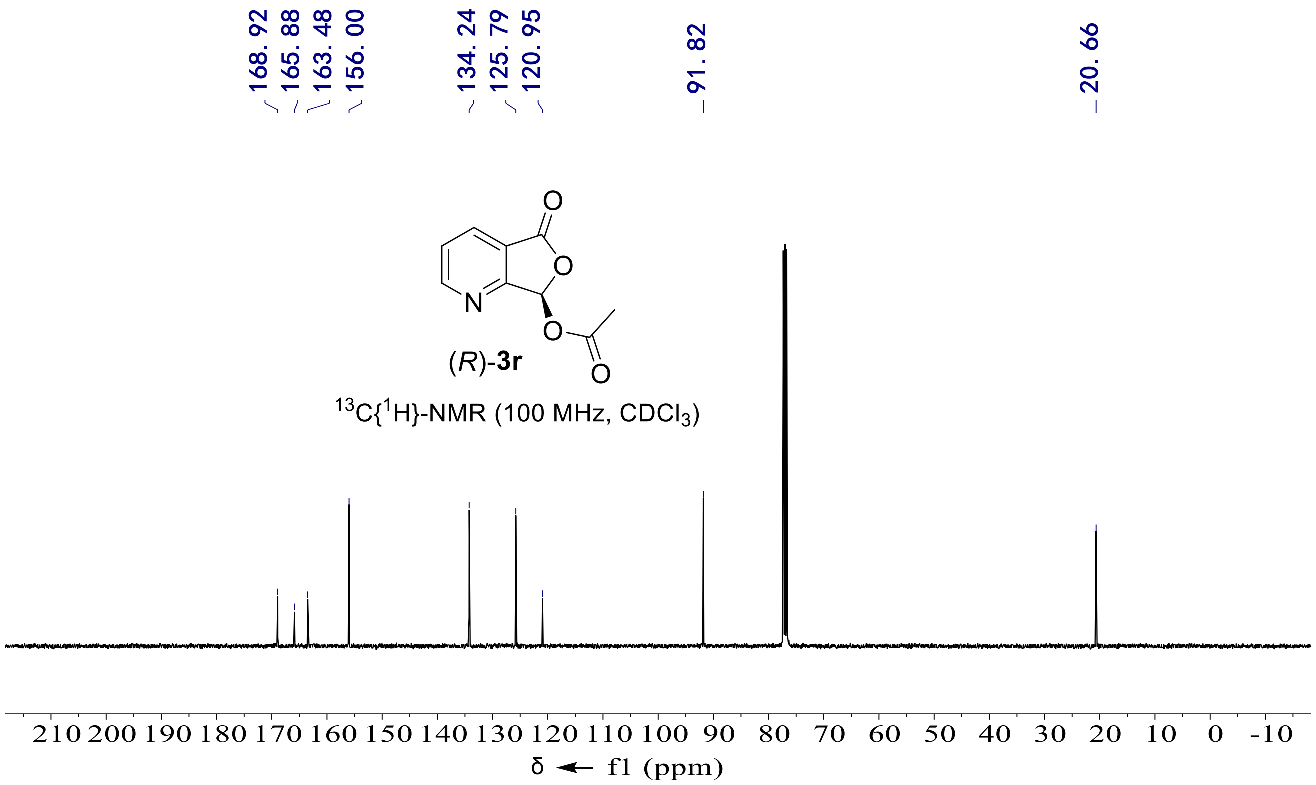

(R)-3s: (*R*)-3-oxo-1,3-dihydroisobenzofuran-1-yl butyrate.

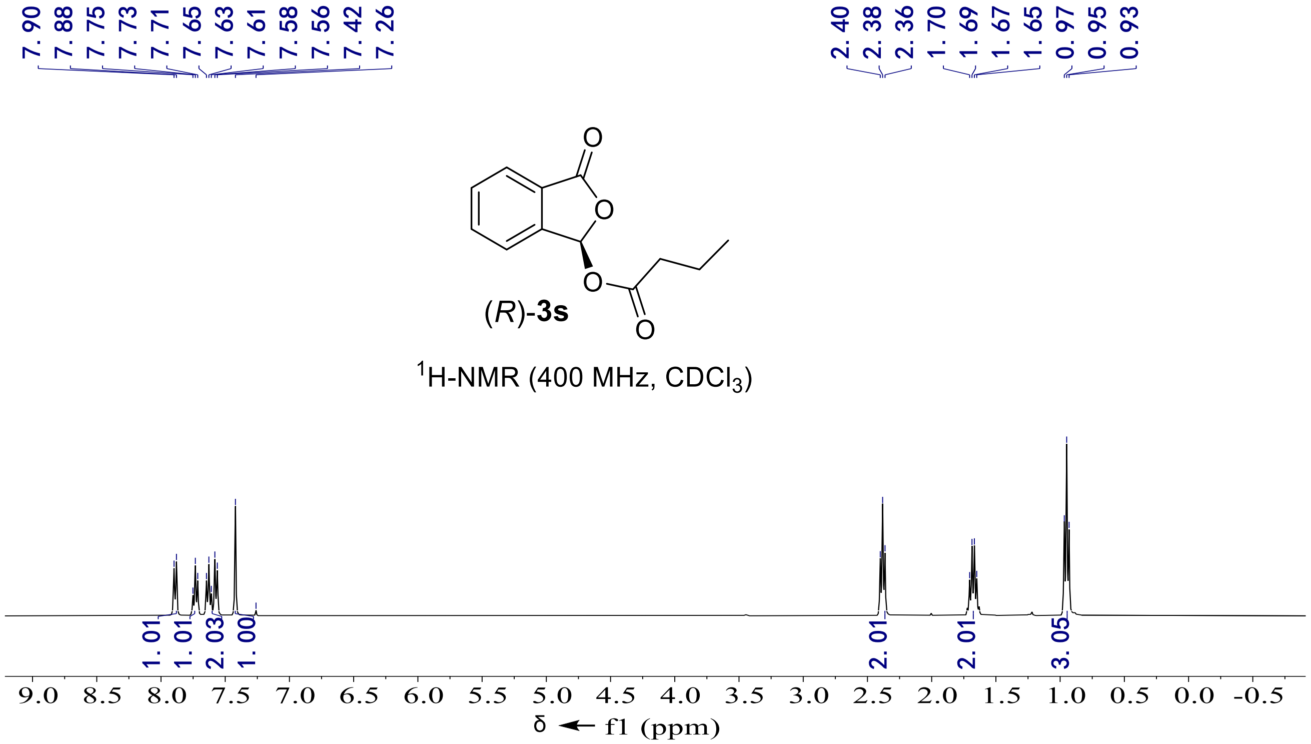

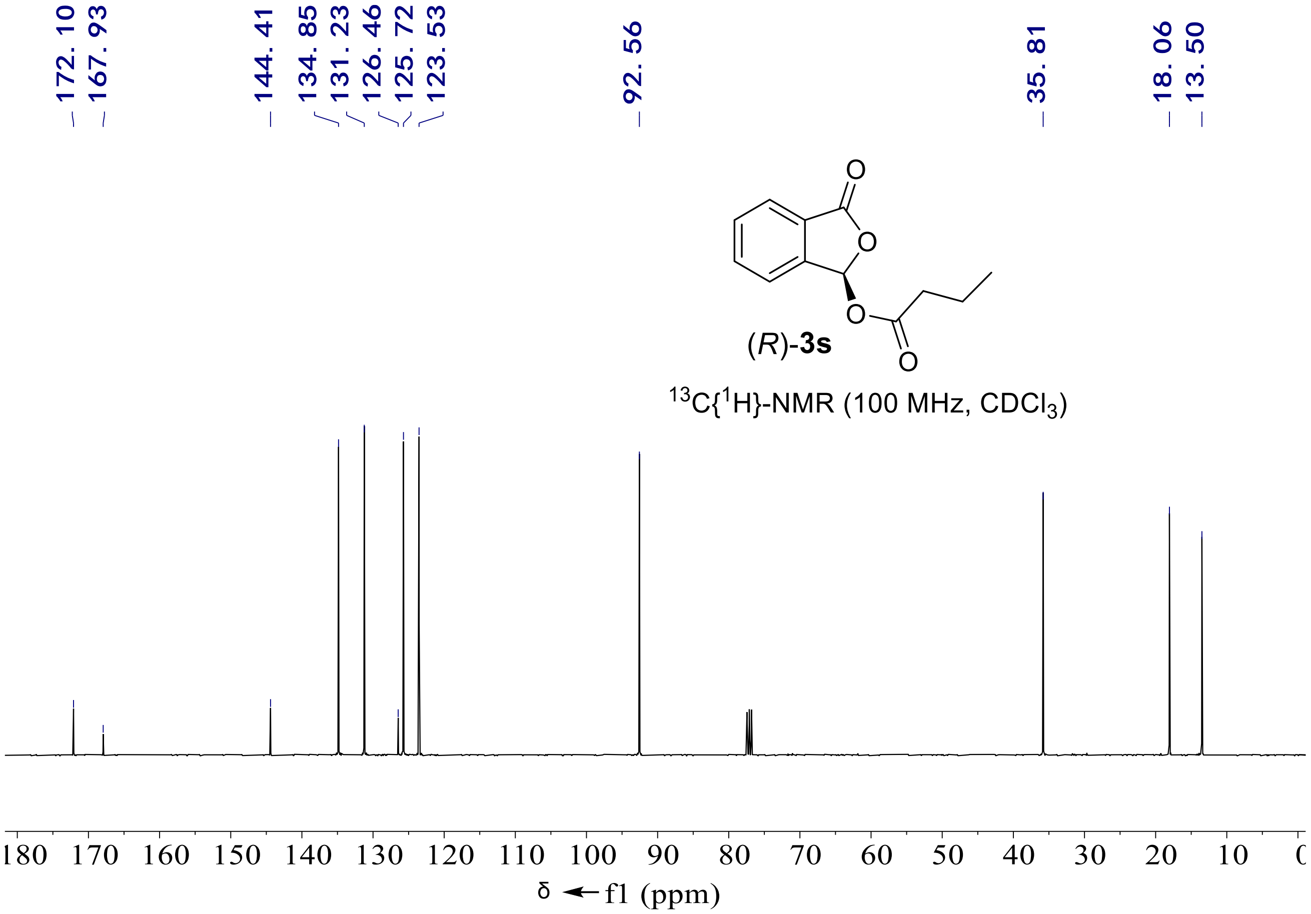

(*R*)-3t: (*R*)-3-oxo-1,3-dihydroisobenzofuran-1-yl butyrate.

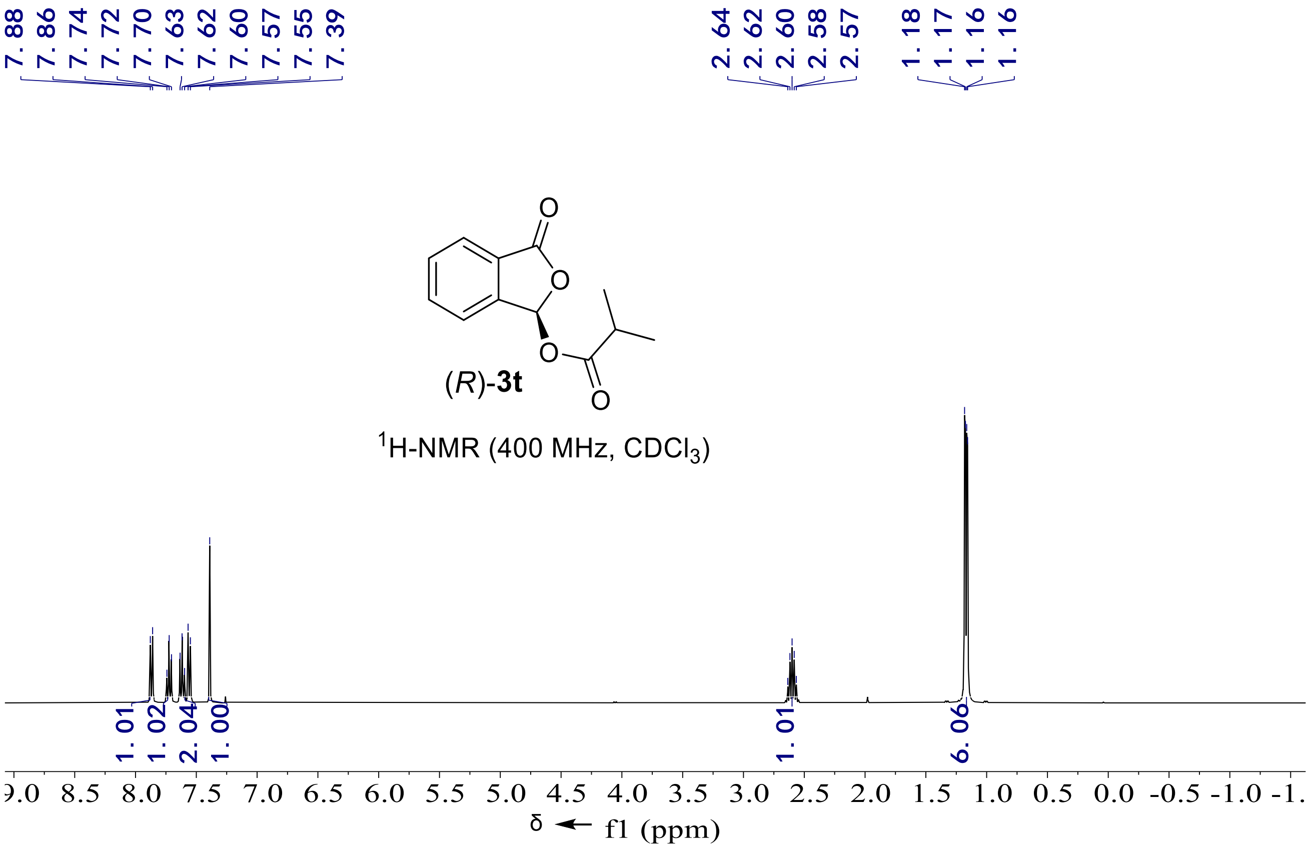

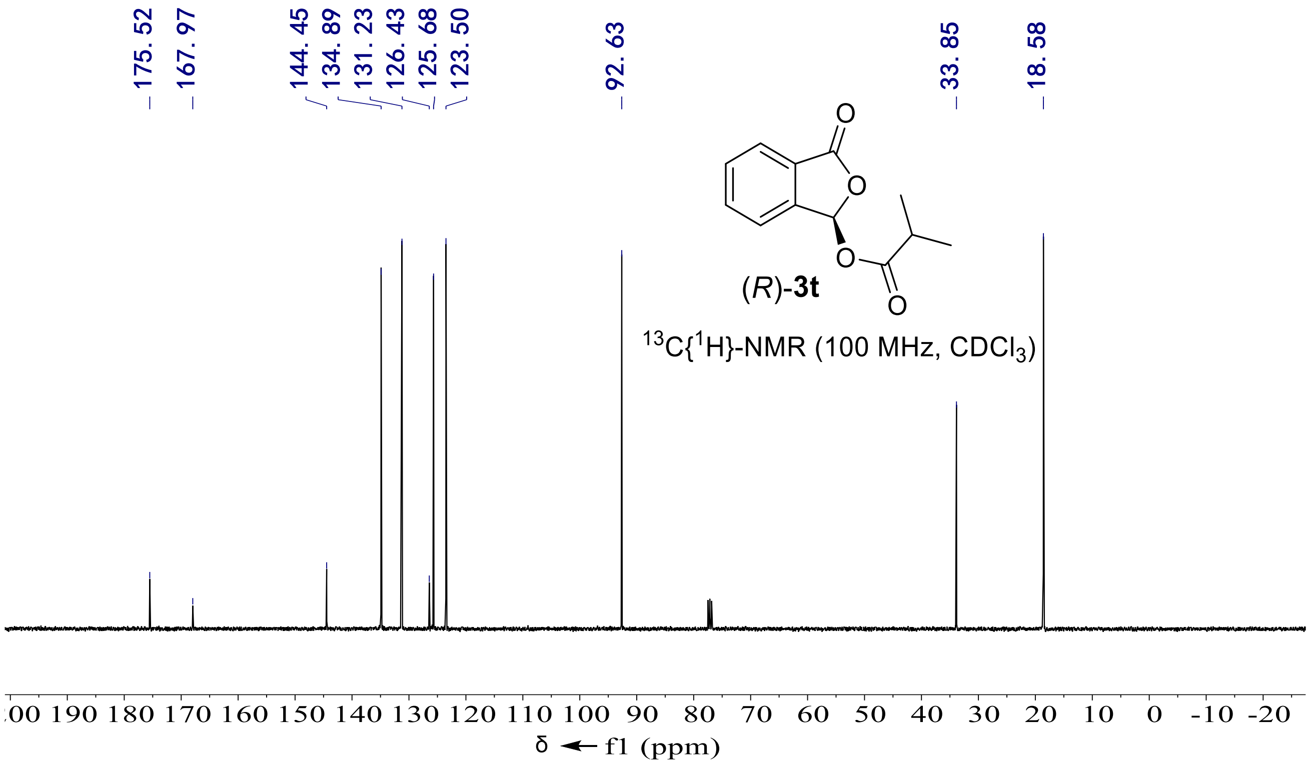

HPLC analyses of chiral products ((*R*)-6a-(*R*)-6h).

(*R*)-6a: (*R*)-1-methyl-3-oxo-1,3-dihydroisobenzofuran-1-yl acetate (HPLC: Chiralpak IC, detected at 210 nm, eluent: n-hexane/2-propanol = 80/20, flow rate = 1.0 mL/min, 25°C).

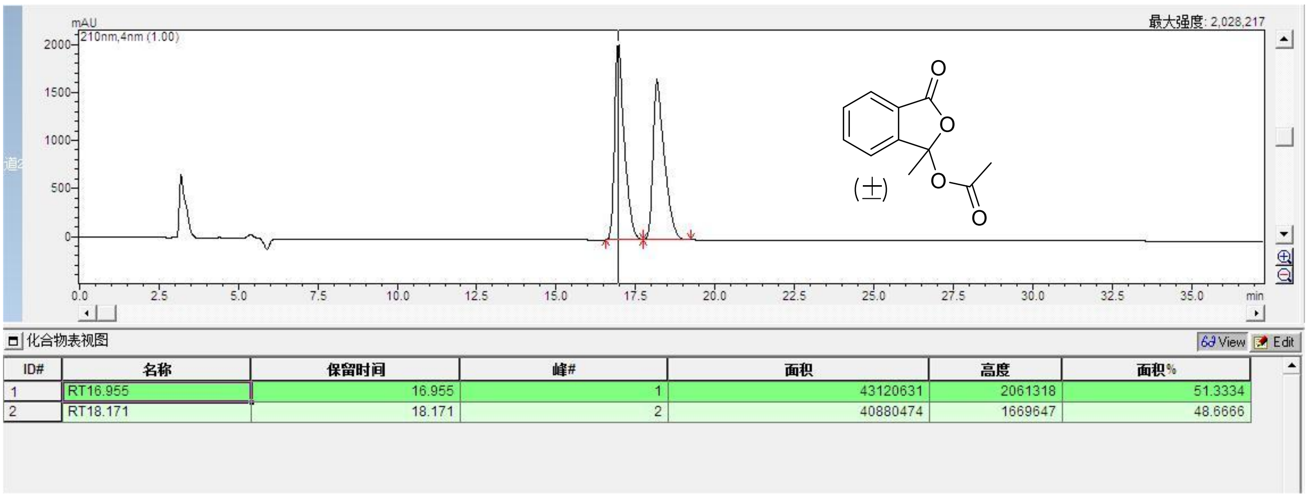

ID#	名称	保留时间	峰#	面积	高度	面积%
1	RT16.955	16.955	1	43120631	2061318	51.3334
2	RT18.171	18.171	2	40880474	1669647	48.6666

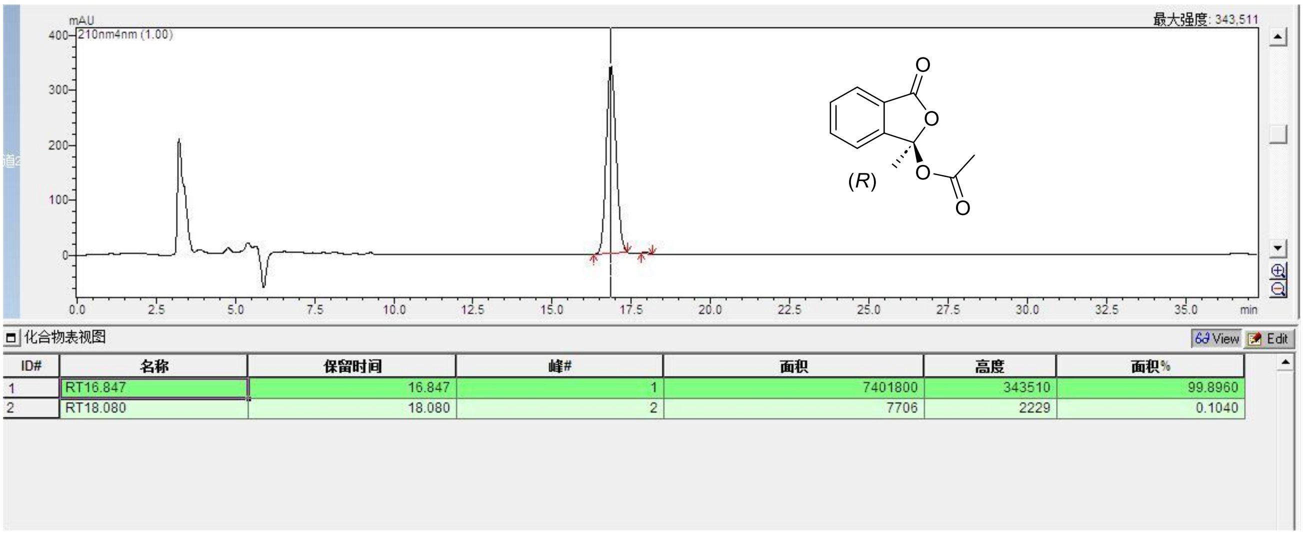

ID#	名称	保留时间	峰#	面积	高度	面积%
1	RT16.847	16.847	1	7401800	343510	99.8960
2	RT18.080	18.080	2	7706	2229	0.1040

<u>Translation of all characters (Chinese) in the above two frameworks to English is as follows:</u>

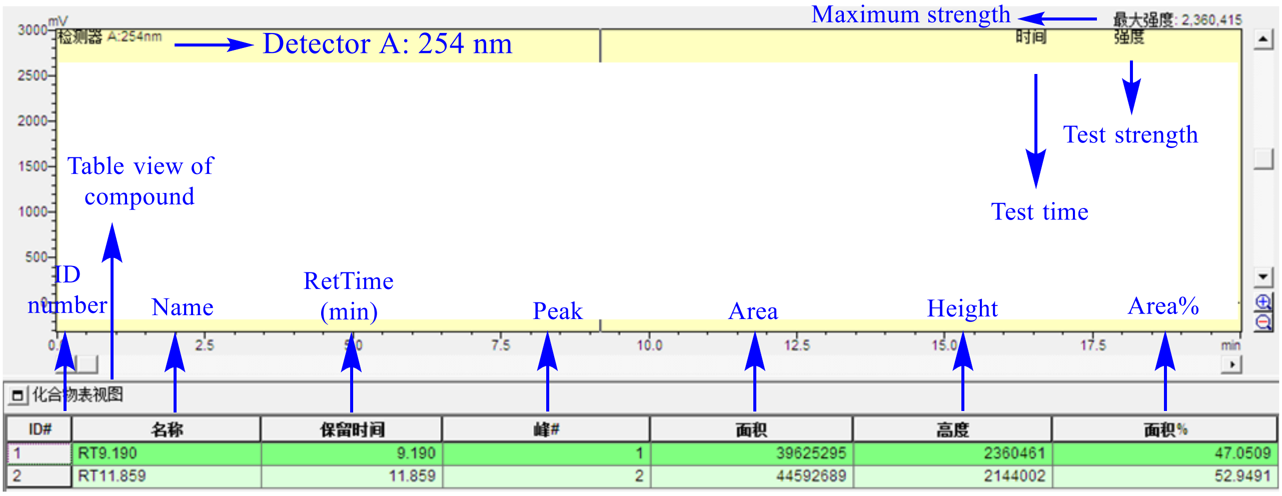

ID#	名称	保留时间	峰#	面积	高度	面积%
1	RT9.190	9.190	1	39625295	2360461	47.0509
2	RT11.859	11.859	2	44592689	2144002	52.9491

(*R*)-6b: (R)-3-oxo-1-phenyl-1,3-dihydroisobenzofuran-1-yl acetate. (HPLC: Chiralpak IC, detected at 210 nm, eluent: n-hexane/2-propanol = 80/20, flow rate = 1.0 mL/min, 25°C).

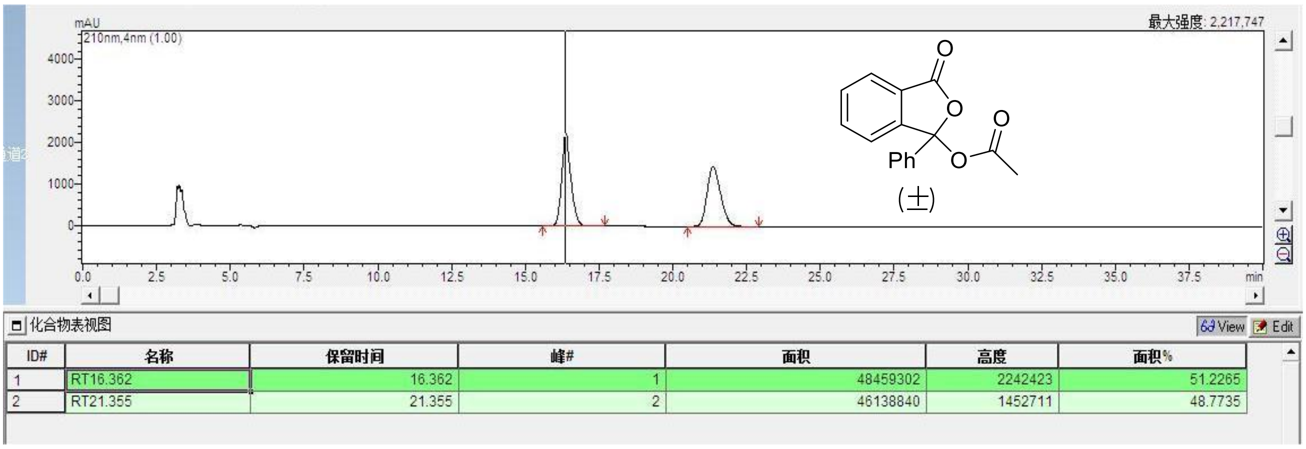

ID#	名称	保留时间	峰#	面积	高度	面积%
1	RT16.362	16.362	1	48459302	2242423	51.2265
2	RT21.355	21.355	2	46138840	1452711	48.7735

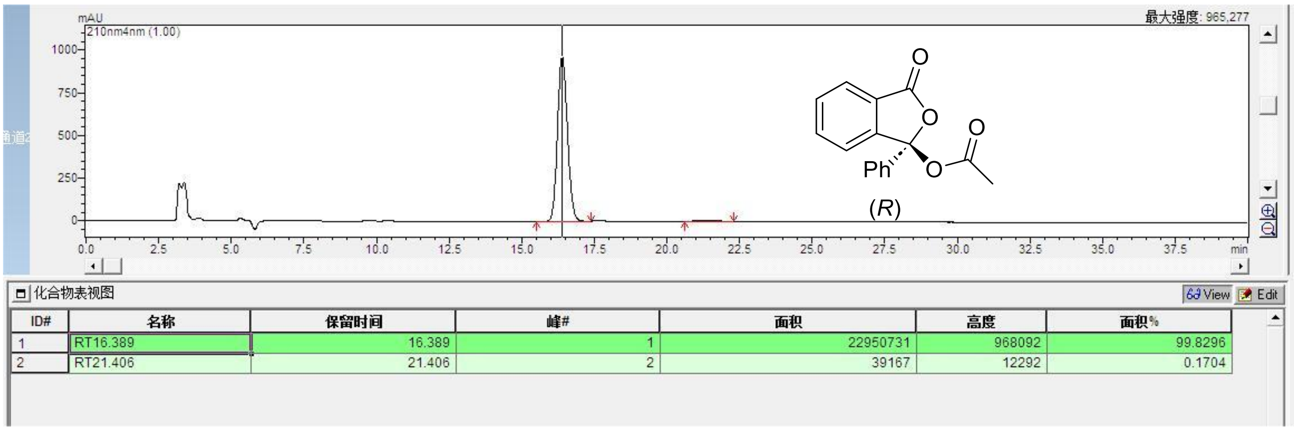

ID#	名称	保留时间	峰#	面积	高度	面积%
1	RT16.389	16.389	1	22950731	968092	99.8296
2	RT21.406	21.406	2	39167	12292	0.1704

Translation of all characters (Chinese) in the above two frameworks to English is as follows:

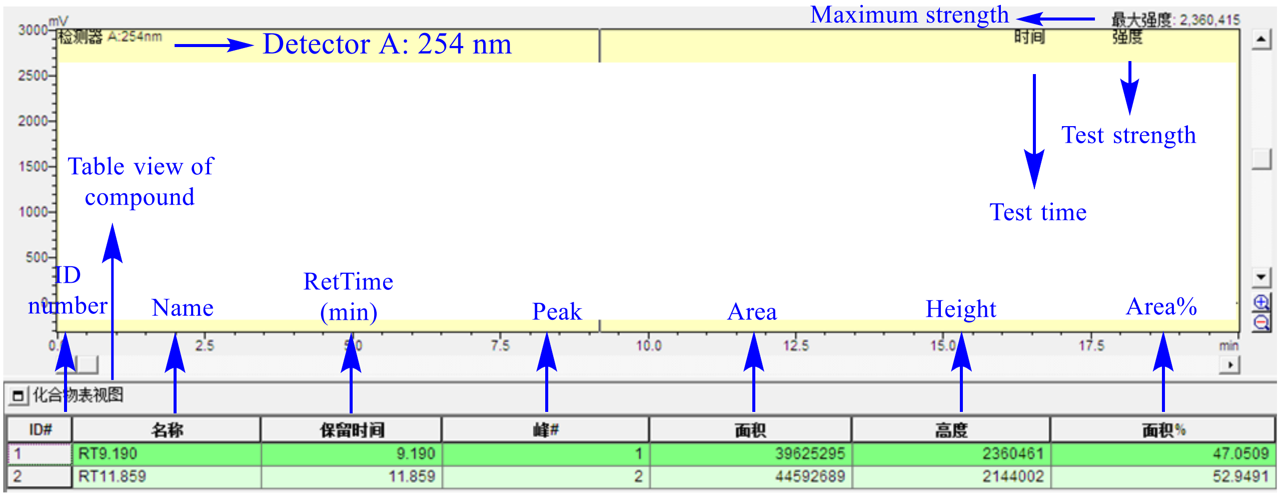

ID#	名称	保留时间	峰#	面积	高度	面积%
1	RT9.190	9.190	1	39625295	2360461	47.0509
2	RT11.859	11.859	2	44592689	2144002	52.9491

(*R*)-6c: (R)-1-(4-fluorophenyl)-3-oxo-1,3-dihydroisobenzofuran-1-yl acetate. (HPLC: Chiralpak IC, detected at 210 nm, eluent: n-hexane/2-propanol = 80/20, flow rate = 1.0 mL/min, 25°C).

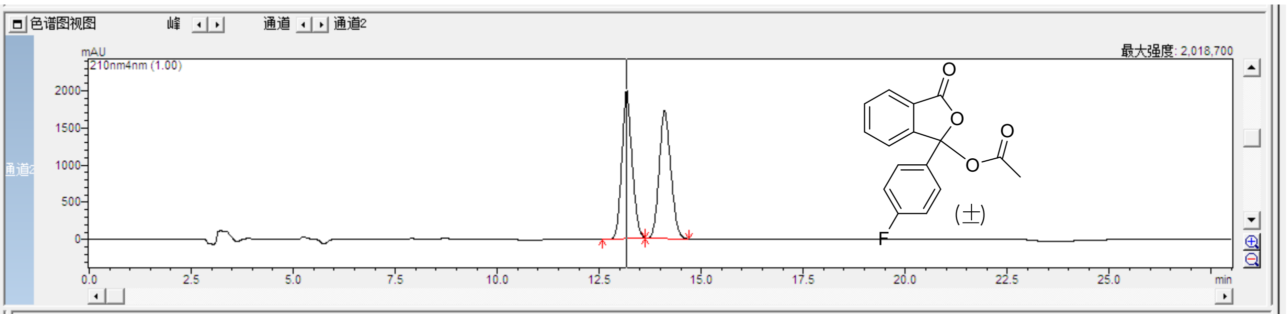

ID#	名称	保留时间	峰#	面积	高度	面积%
1	RT13.163	13.163	1	34284068	2005867	50.4584
2	RT14.094	14.094	2	33661081	1720273	49.5416

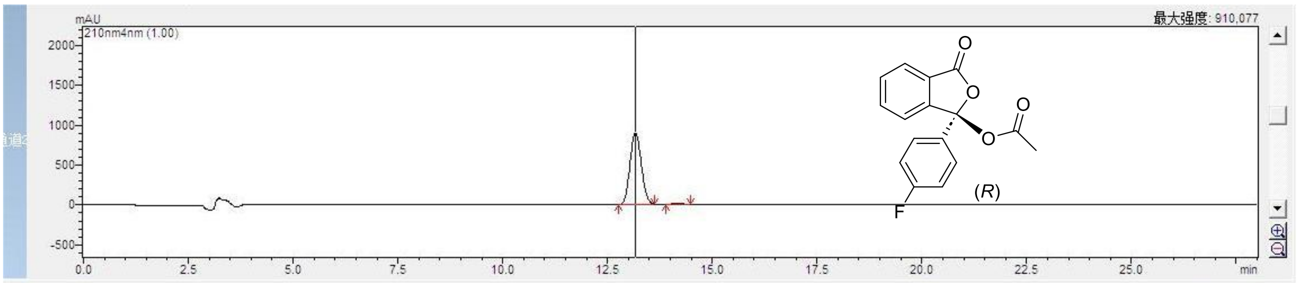

ID#	名称	保留时间	峰#	面积	高度	面积%
1	RT13.163	13.163	1	16845217	902410	99.4472
2	RT14.091	14.091	2	93642	31346	0.5528

<u>Translation of all characters (Chinese) in the above two frameworks to English is as follows:</u>

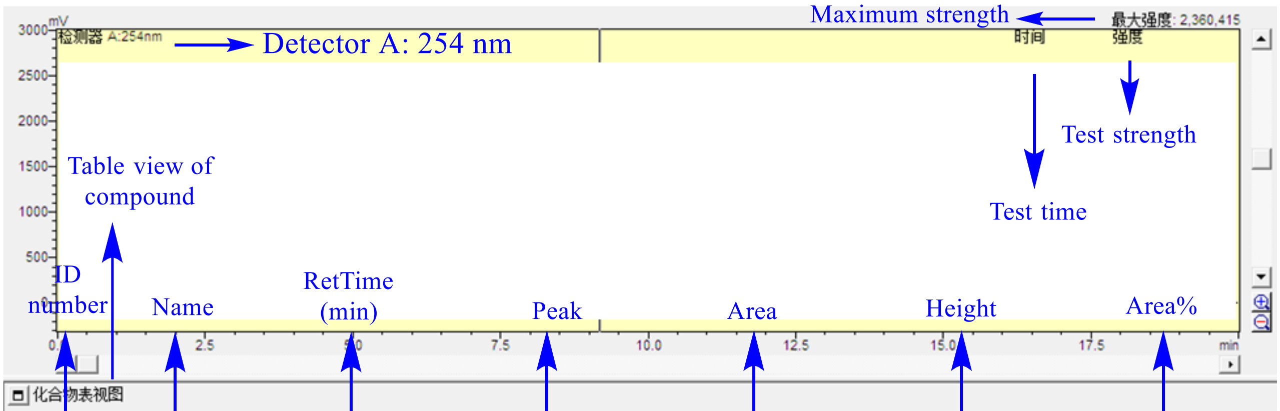

ID#	名称	保留时间	峰#	面积	高度	面积%
1	RT9.190	9.190	1	39625295	2360461	47.0509
2	RT11.859	11.859	2	44592689	2144002	52.9491

(*R*)-6d: (*R*)-1-(4-chlorophenyl)-3-oxo-1,3-dihydroisobenzofuran-1-yl acetate. (HPLC: Chiralpak OD-H, detected at 210 nm, eluent: n-hexane/2-propanol = 90/10, flow rate = 1.0 mL/min, 25°C).

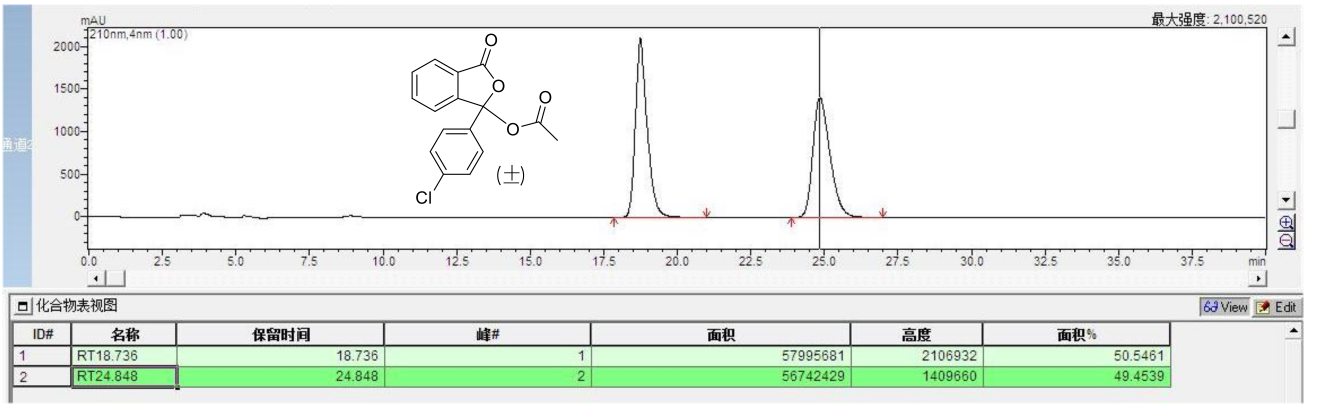

ID#	名称	保留时间	峰#	面积	高度	面积%
1	RT18.736	18.736	1	57995681	2106932	50.5461
2	RT24.848	24.848	2	56742429	1409660	49.4539

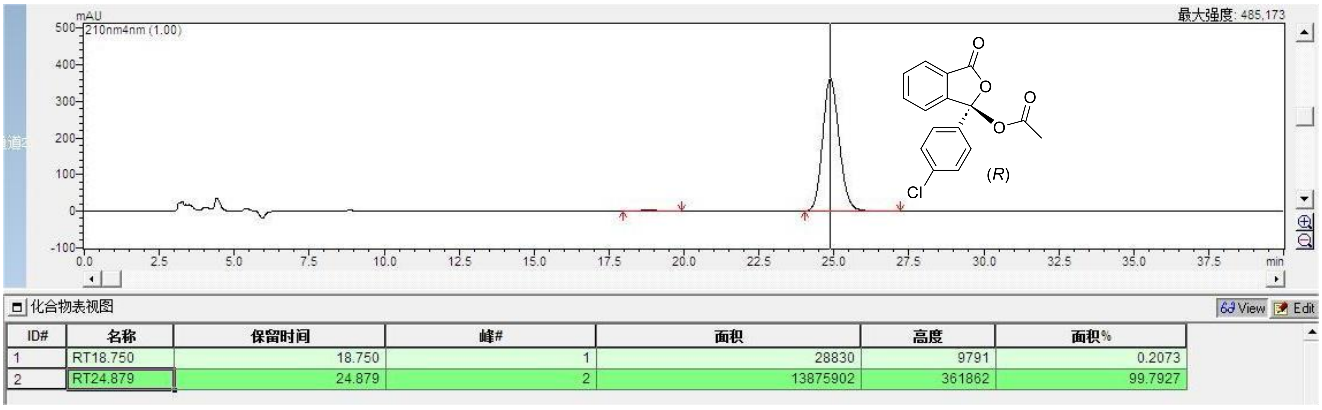

ID#	名称	保留时间	峰#	面积	高度	面积%
1	RT18.750	18.750	1	28830	9791	0.2073
2	RT24.879	24.879	2	13875902	361862	99.7927

<u>Translation of all characters (Chinese) in the above two frameworks to English is as follows:</u>

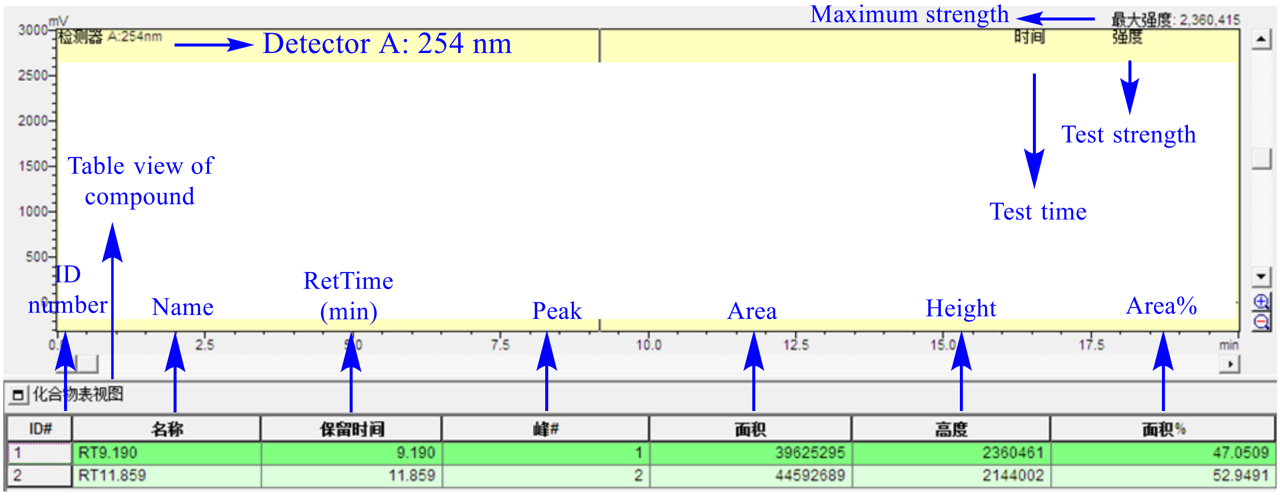

ID#	名称	保留时间	峰#	面积	高度	面积%
1	RT9.190	9.190	1	39625295	2360461	47.0509
2	RT11.859	11.859	2	44592689	2144002	52.9491

(*R*)-6e: (R)-1-(4-bromophenyl)-3-oxo-1,3-dihydroisobenzofuran-1-yl acetate. (HPLC: Chiralpak IC, detected at 210 nm, eluent: n-hexane/2-propanol = 95/5, flow rate = 1.0 mL/min, 25°C).

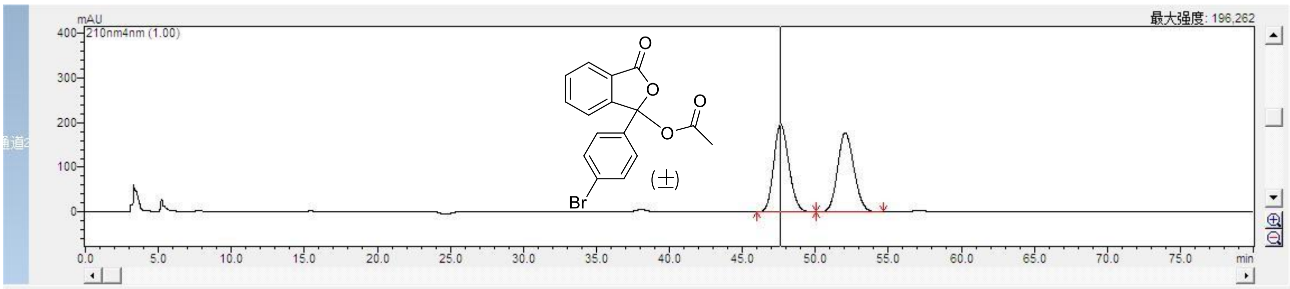

化合物表视图

ID#	名称	保留时间	峰#	面积	高度	面积%
1	RT47.619	47.619	1	14160042	196268	50.4830
2	RT52.048	52.048	2	13889077	177266	49.5170

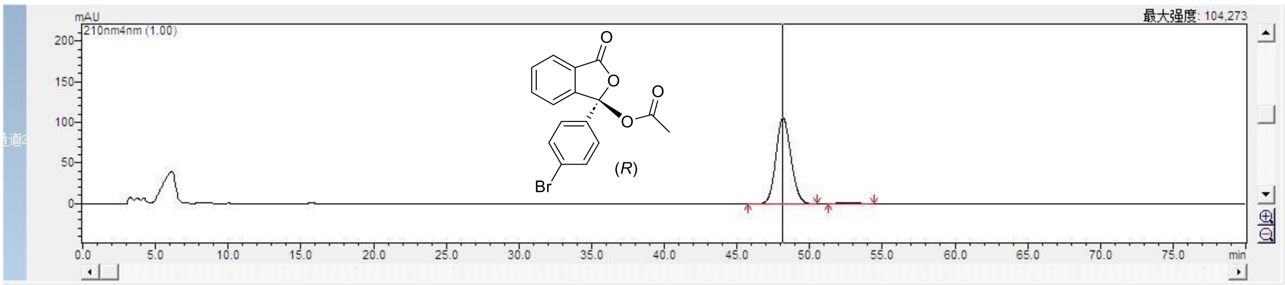

化合物表视图

ID#	名称	保留时间	峰#	面积	高度	面积%
1	RT48.161	48.161	1	7627053	106084	99.5445
2	RT52.623	52.623	2	34898	3247	0.4555

<u>Translation of all characters (Chinese) in the above two frameworks to English is as follows:</u>

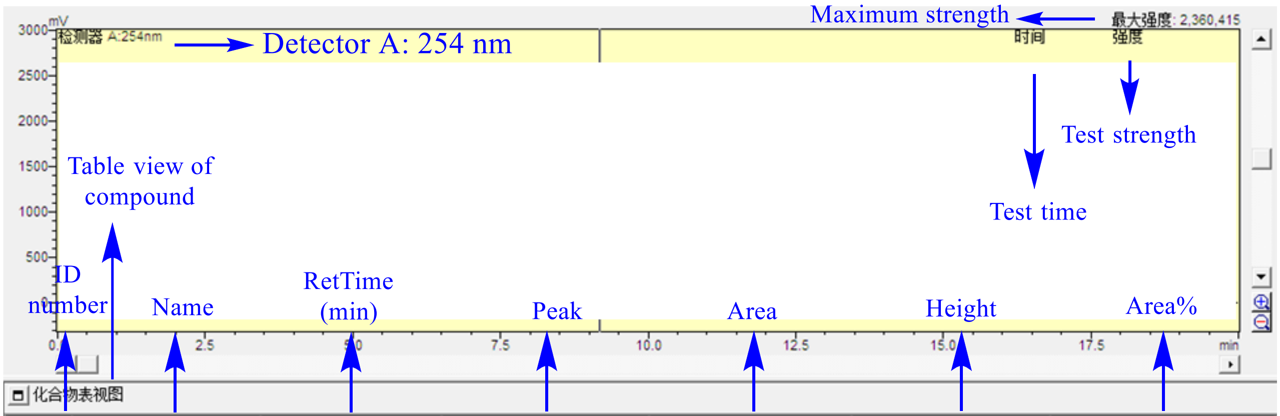

ID#	名称	保留时间	峰#	面积	高度	面积%
1	RT9.190	9.190	1	39625295	2360461	47.0509
2	RT11.859	11.859	2	44592689	2144002	52.9491

(*R*)-6f: (*R*)-3-oxo-1-(p-tolyl)-1,3-dihydroisobenzofuran-1-yl acetate. (HPLC: Chiralpak IC, detected at 210 nm, eluent: n-hexane/2-propanol = 80/20, flow rate = 1.0 mL/min, 25°C).

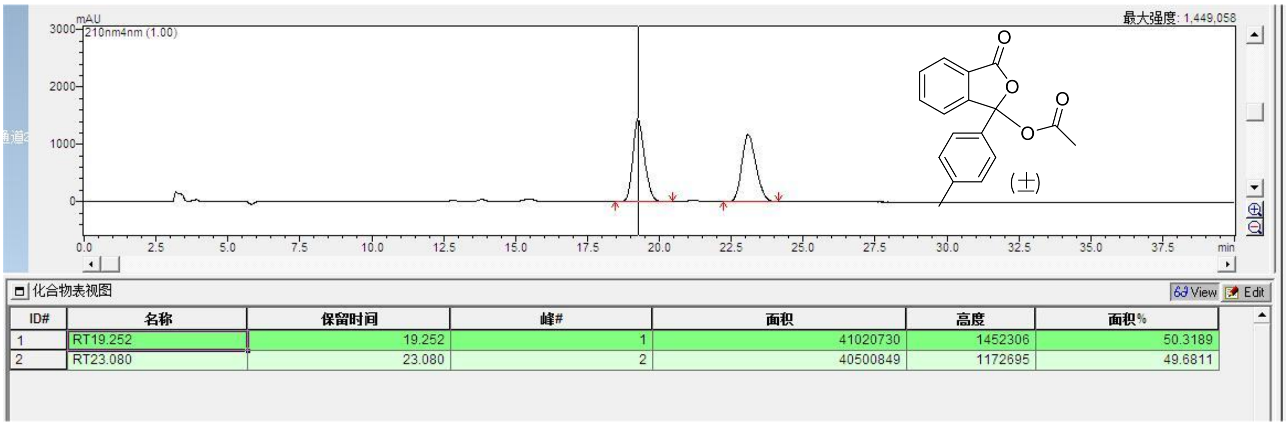

ID#	名称	保留时间	峰#	面积	高度	面积%
1	RT19.252	19.252	1	41020730	1452306	50.3189
2	RT23.080	23.080	2	40500849	1172695	49.6811

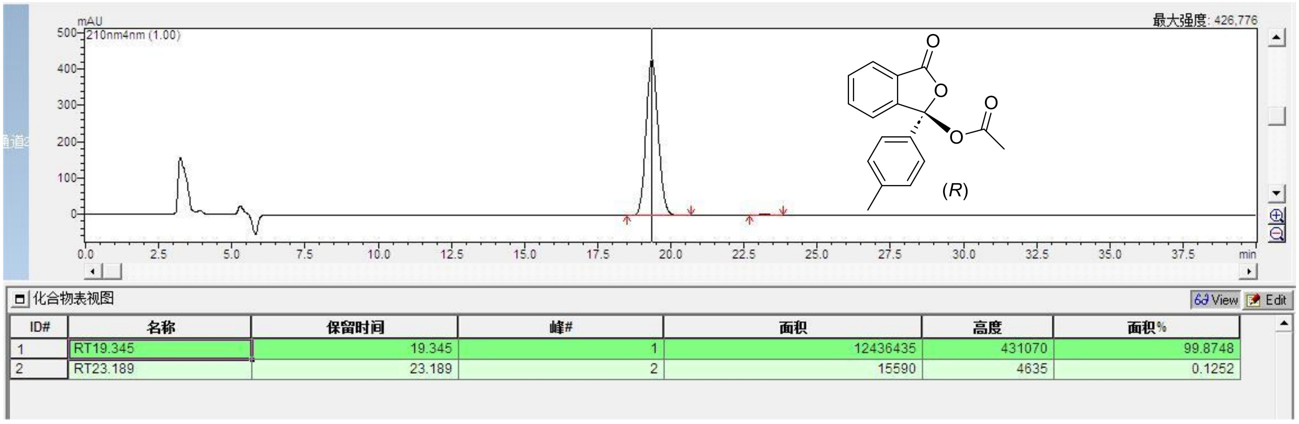

ID#	名称	保留时间	峰#	面积	高度	面积%
1	RT19.345	19.345	1	12436435	431070	99.8748
2	RT23.189	23.189	2	15590	4635	0.1252

<u>Translation of all characters (Chinese) in the above two frameworks to English is as follows:</u>

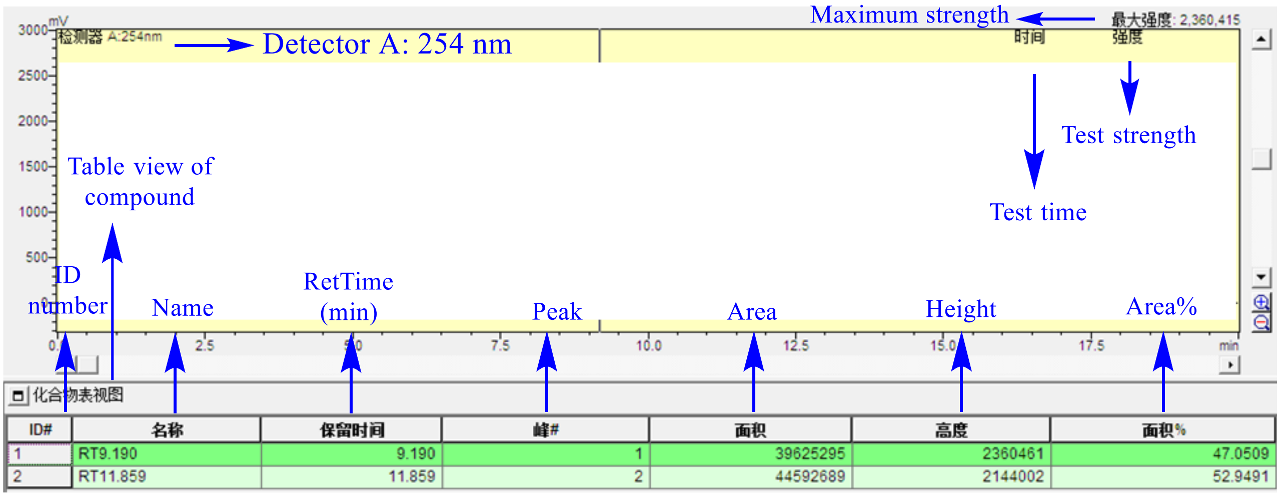

ID#	名称	保留时间	峰#	面积	高度	面积%
1	RT9.190	9.190	1	39625295	2360461	47.0509
2	RT11.859	11.859	2	44592689	2144002	52.9491

(*R*)-6g: (*R*)-1-(4-ethylphenyl)-3-oxo-1,3-dihydroisobenzofuran-1-yl acetate. (HPLC: Chiralpak IC, detected at 210 nm, eluent: n-hexane/2-propanol = 95/5, flow rate = 1.0 mL/min, 25°C).

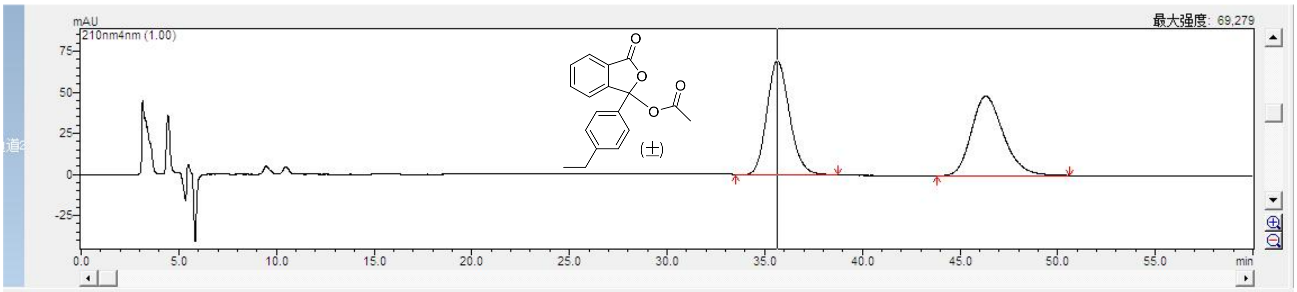

化合物表视图

ID#	名称	保留时间	峰#	面积	高度	面积%
1	RT35.661	35.661	1	5593101	69493	50.2864
2	RT46.319	46.319	2	5529393	48330	49.7136

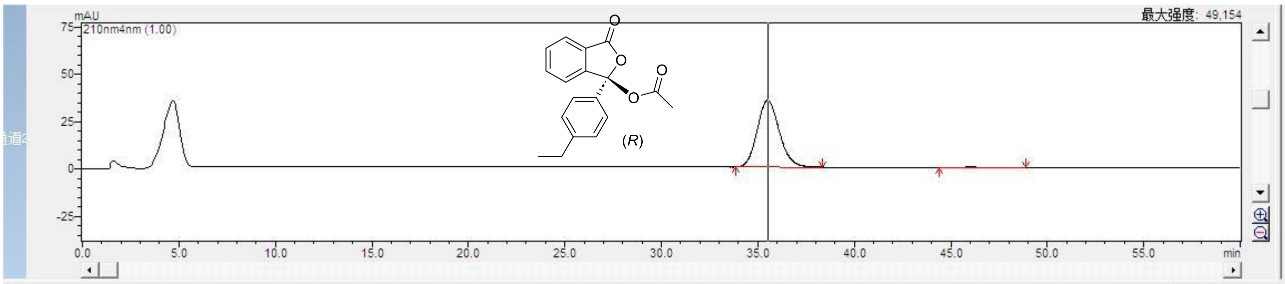

化合物表视图

ID#	名称	保留时间	峰#	面积	高度	面积%
1	RT35.505	35.505	1	2819595	35321	99.5058
2	RT46.177	46.177	2	14003	889	0.4942

Translation of all characters (Chinese) in the above two frameworks to English is as follows:

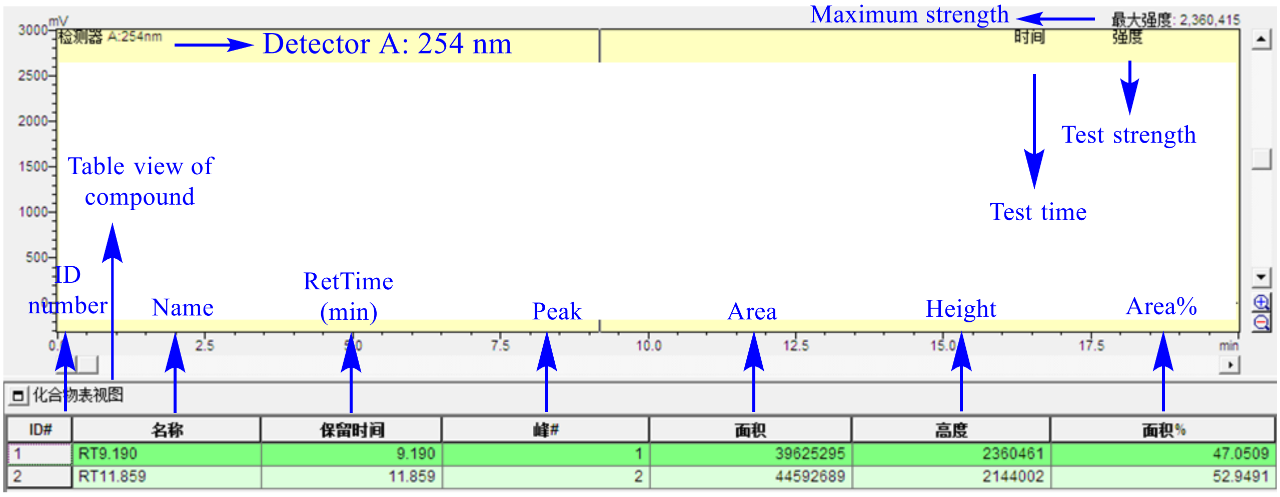

(*R*)-6h: (*R*)-3-oxo-1-(thiophen-2-yl)-1,3-dihydroisobenzofuran-1-yl acetate. (HPLC: Chiralpak IC, detected at 210 nm, eluent: n-hexane/2-propanol = 80/20, flow rate = 1.0 mL/min, 25°C).

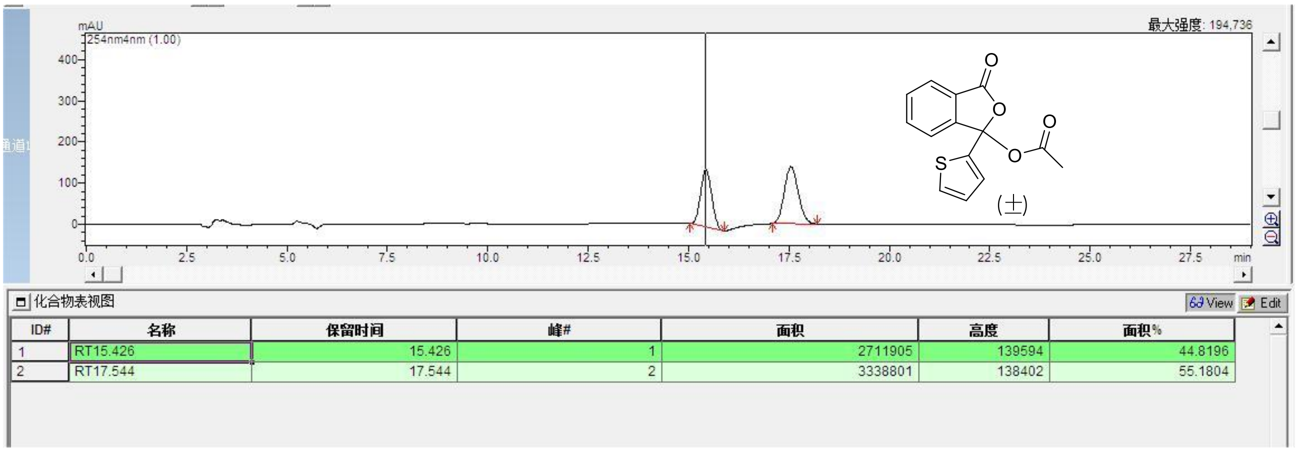

ID#	名称	保留时间	峰#	面积	高度	面积%
1	RT15.426	15.426	1	2711905	139594	44.8196
2	RT17.544	17.544	2	3338801	138402	55.1804

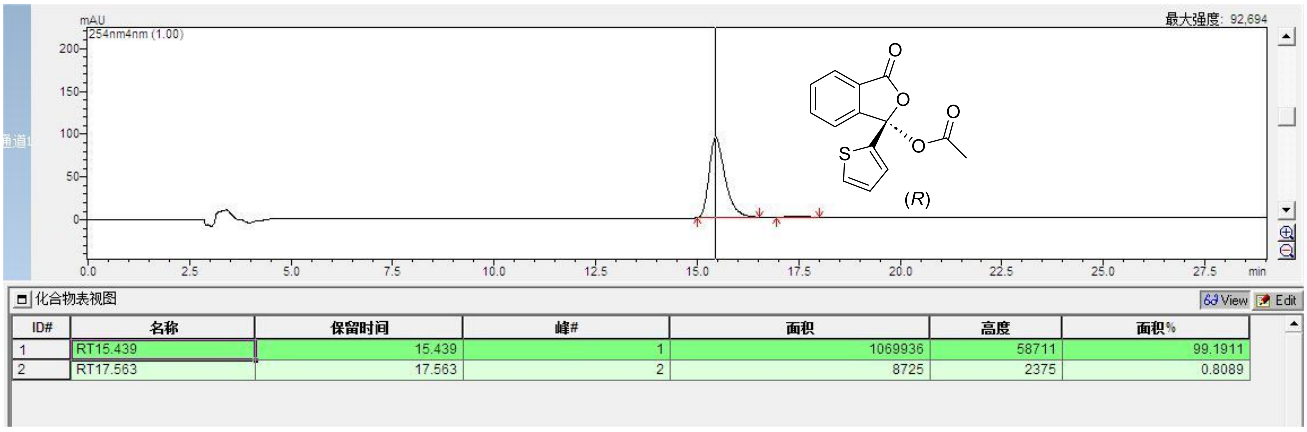

ID#	名称	保留时间	峰#	面积	高度	面积%
1	RT15.439	15.439	1	1069936	58711	99.1911
2	RT17.563	17.563	2	8725	2375	0.8089

<u>Translation of all characters (Chinese) in the above two frameworks to English is as follows:</u>

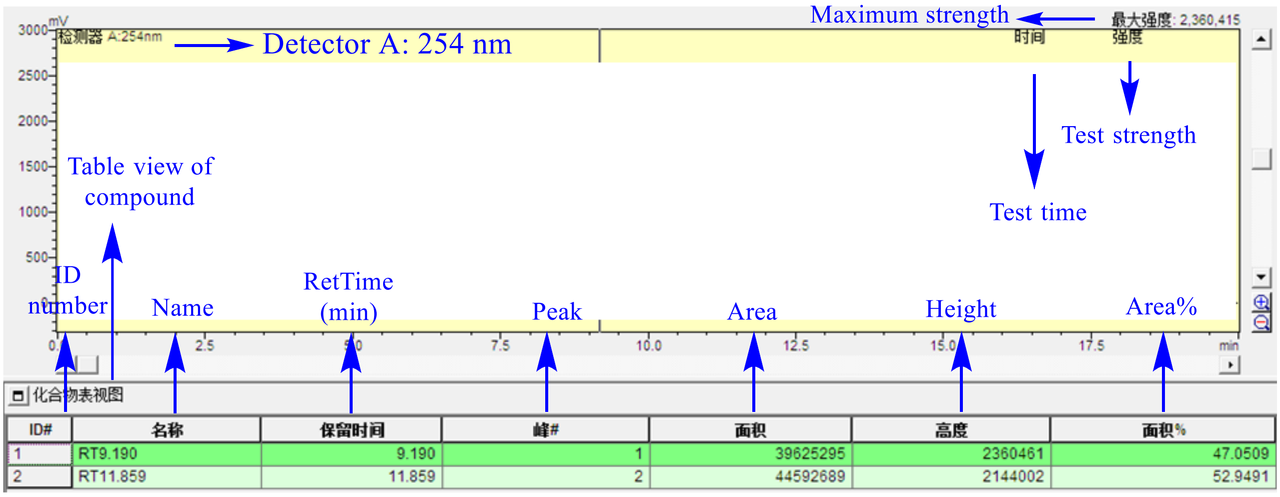

ID#	名称	保留时间	峰#	面积	高度	面积%
1	RT9.190	9.190	1	39625295	2360461	47.0509
2	RT11.859	11.859	2	44592689	2144002	52.9491

Characterizations of chiral products ((*R*)-6a-(*R*)-6h).

(*R*)-6a: (*R*)-1-methyl-3-oxo-1,3-dihydroisobenzofuran-1-yl acetate.

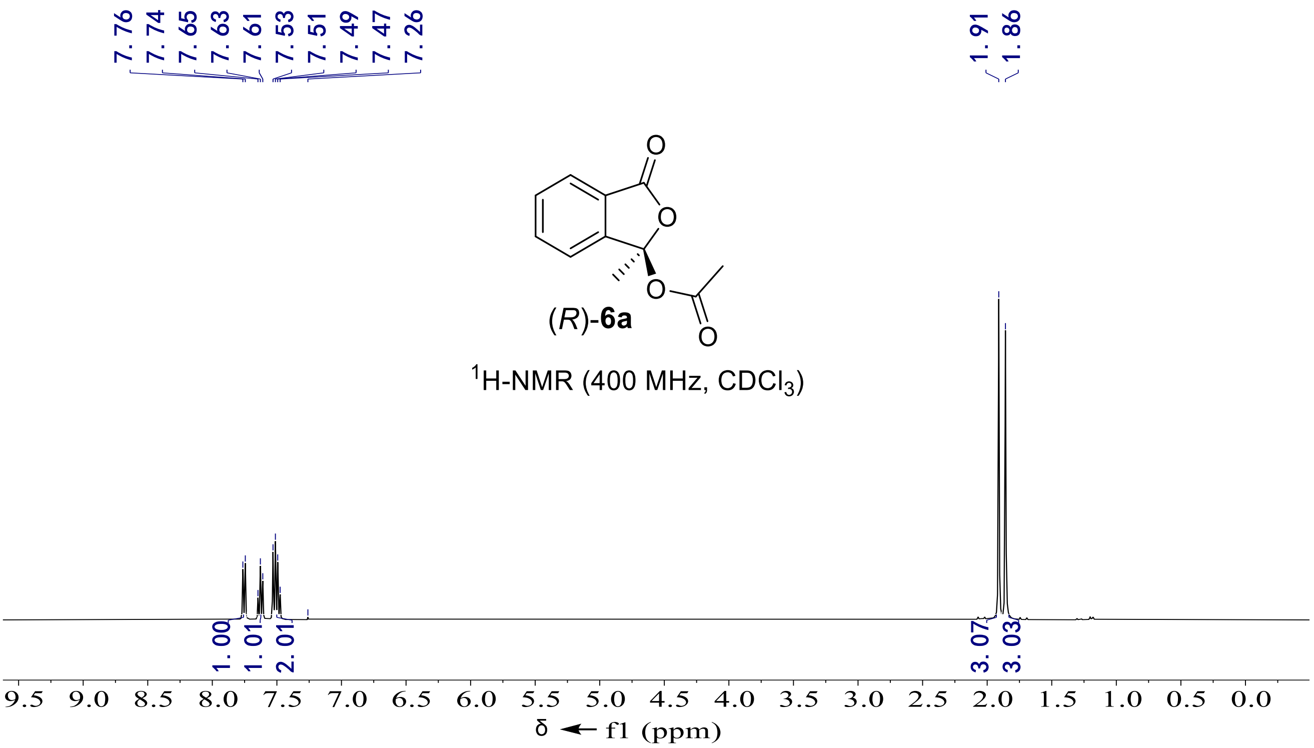

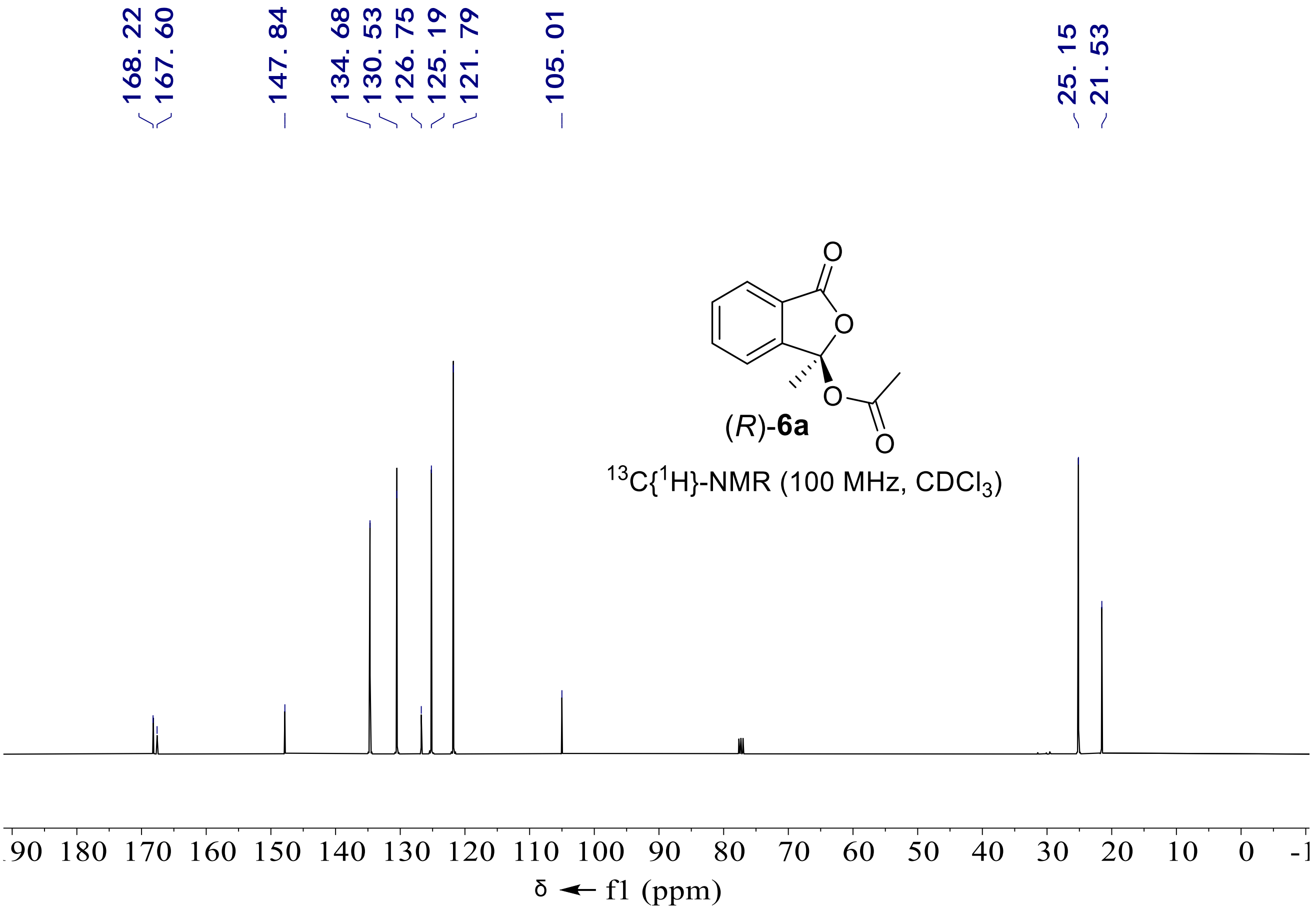

(*R*)-6b: (*R*)-3-oxo-1-phenyl-1,3-dihydroisobenzofuran-1-yl acetate.

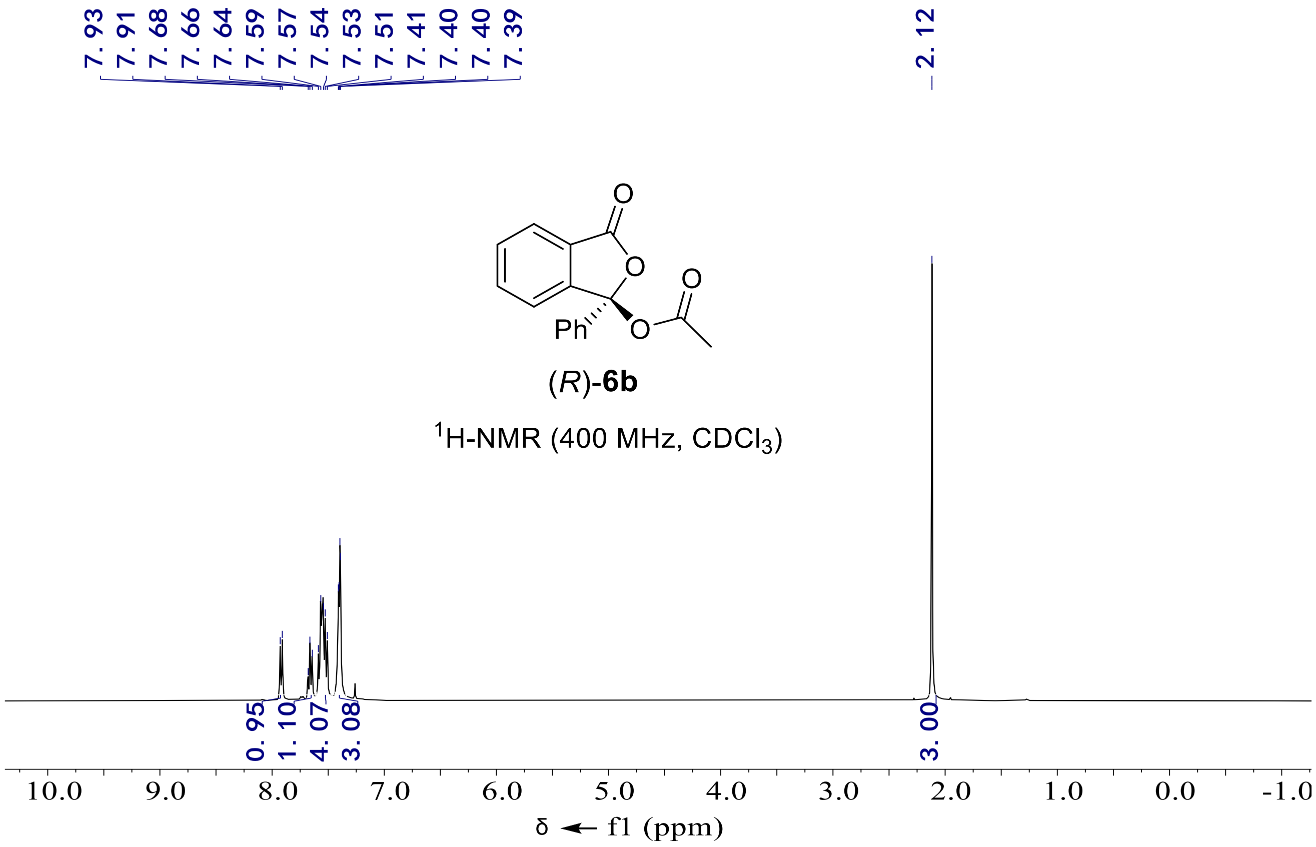

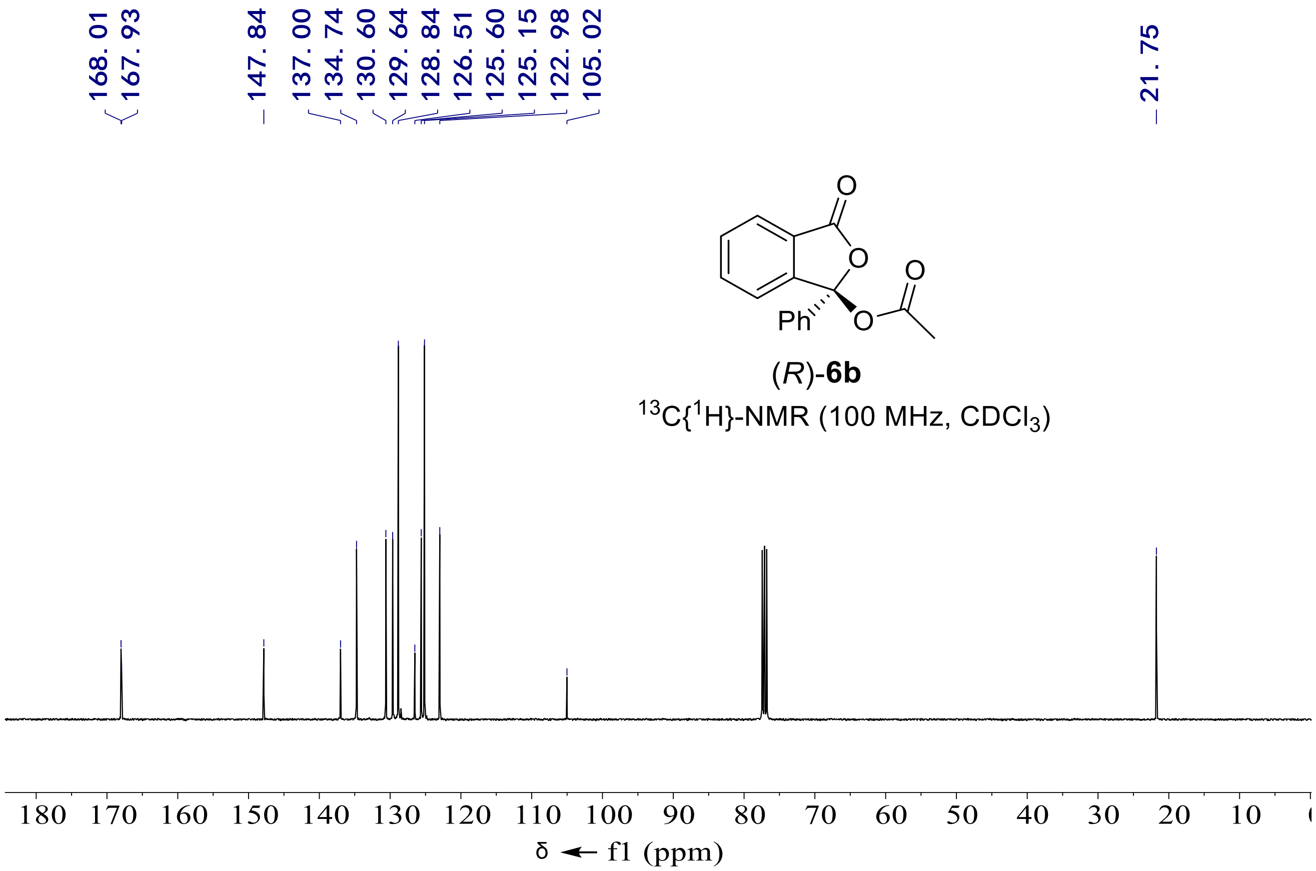

(*R*)-6c: (*R*)-1-(4-fluorophenyl)-3-oxo-1,3-dihydroisobenzofuran-1-yl acetate.

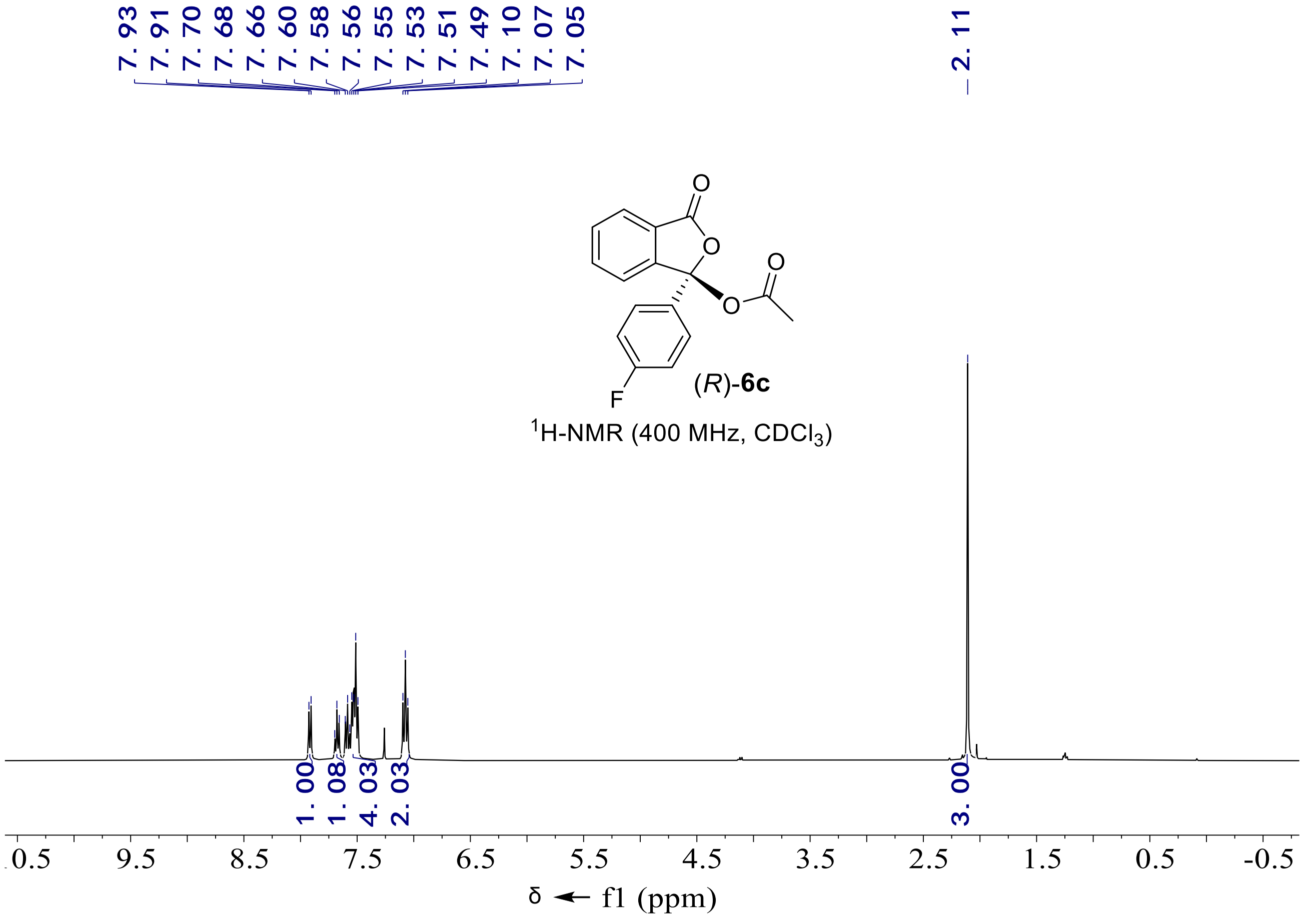

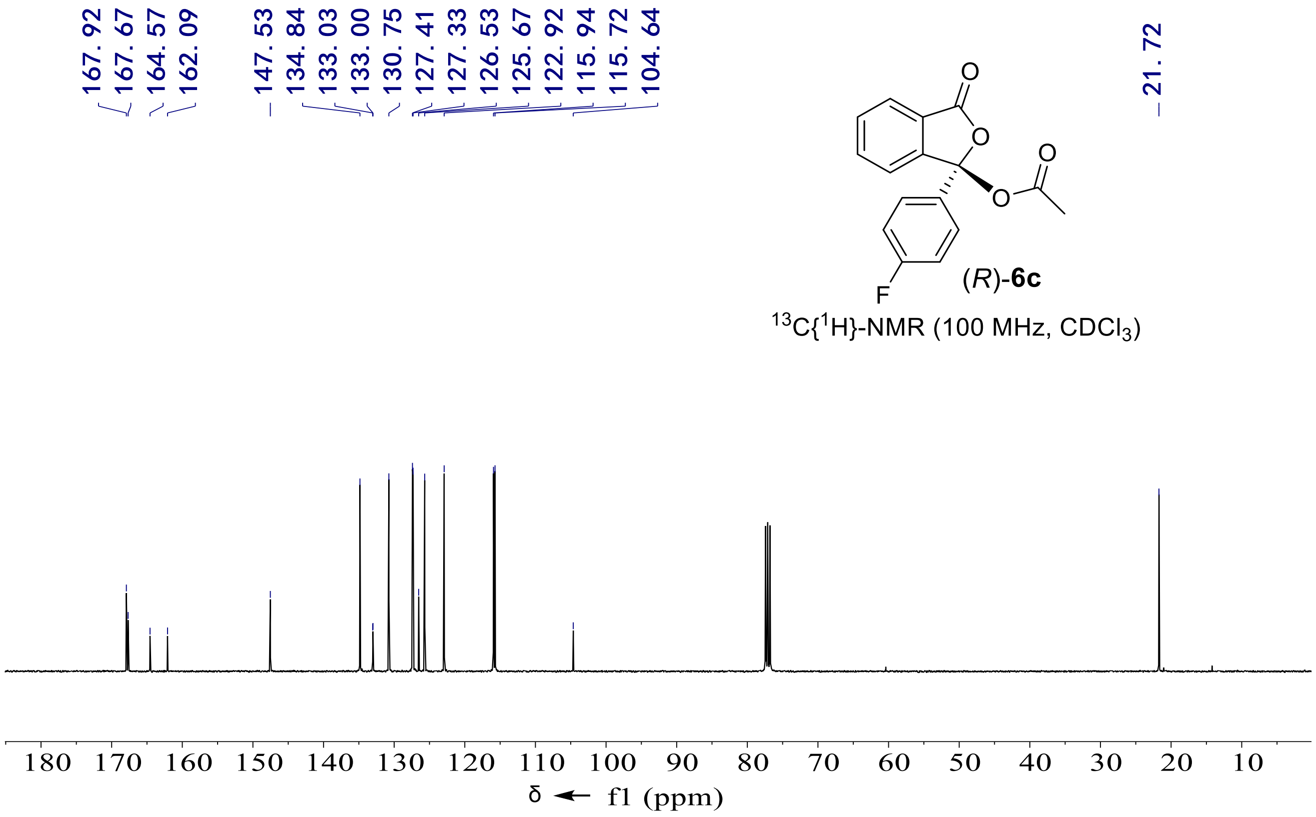

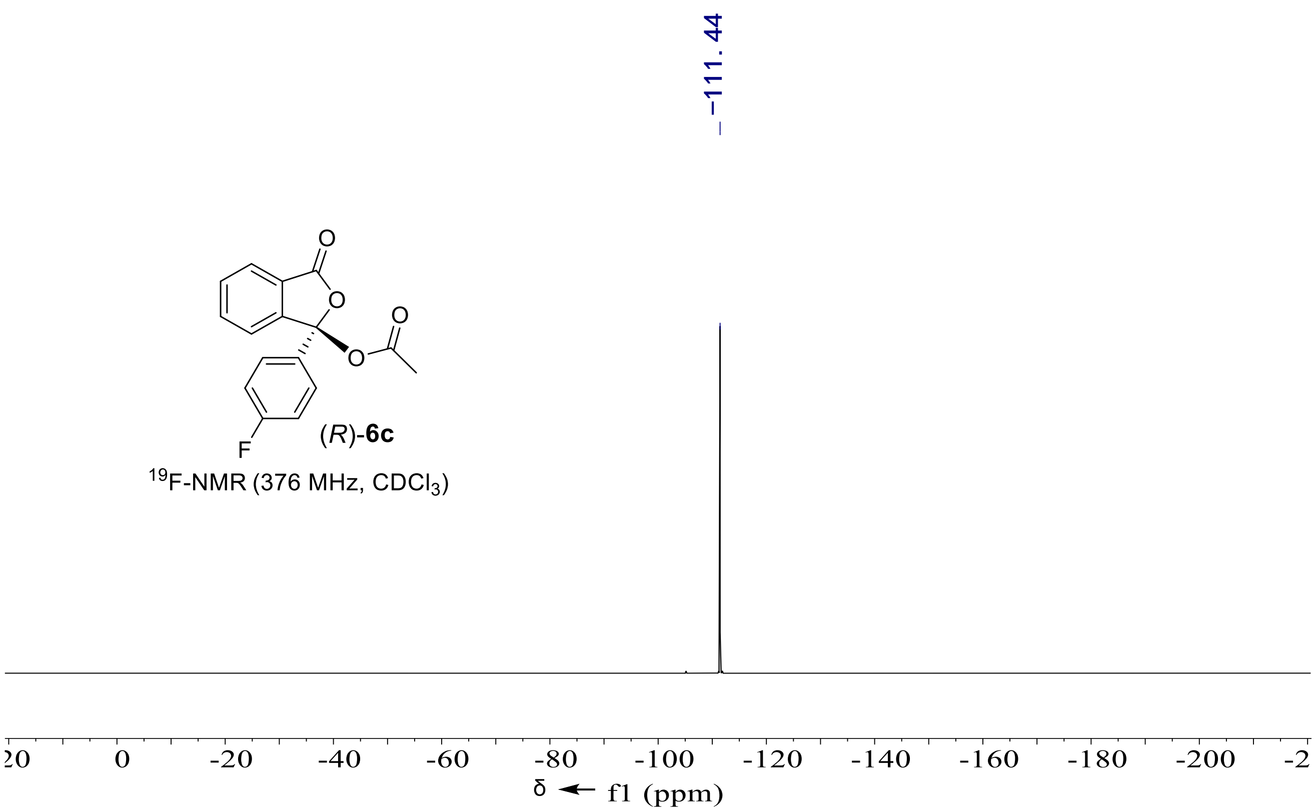

−111.44
(R)-6c
19F-NMR (376 MHz, CDCl3)
20
0
-20
-40
-60
-80
-100
-120
-140
-160
-180
-200
δ ← f1 (ppm)

(*R*)-6d: (*R*)-1-(4-chlorophenyl)-3-oxo-1,3-dihydroisobenzofuran-1-yl acetate.

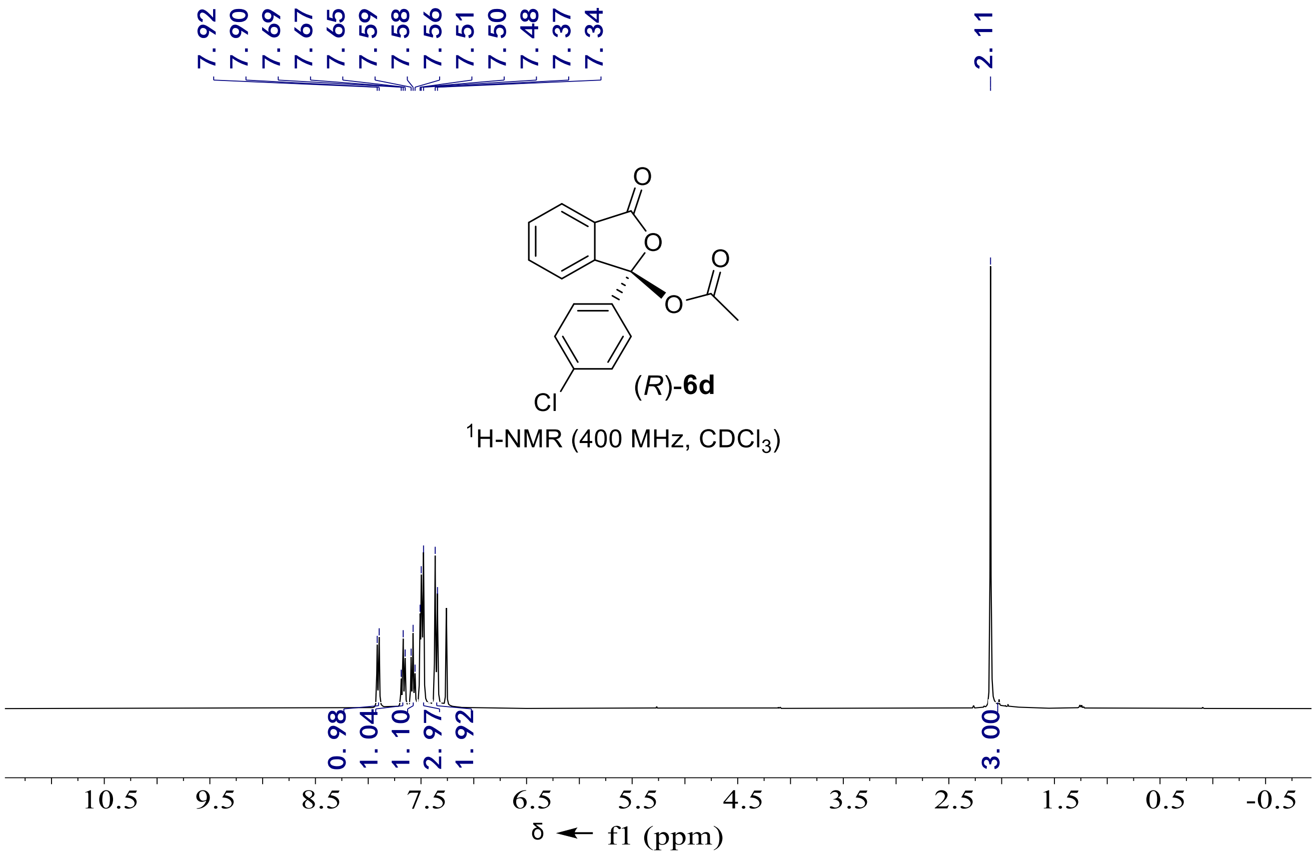

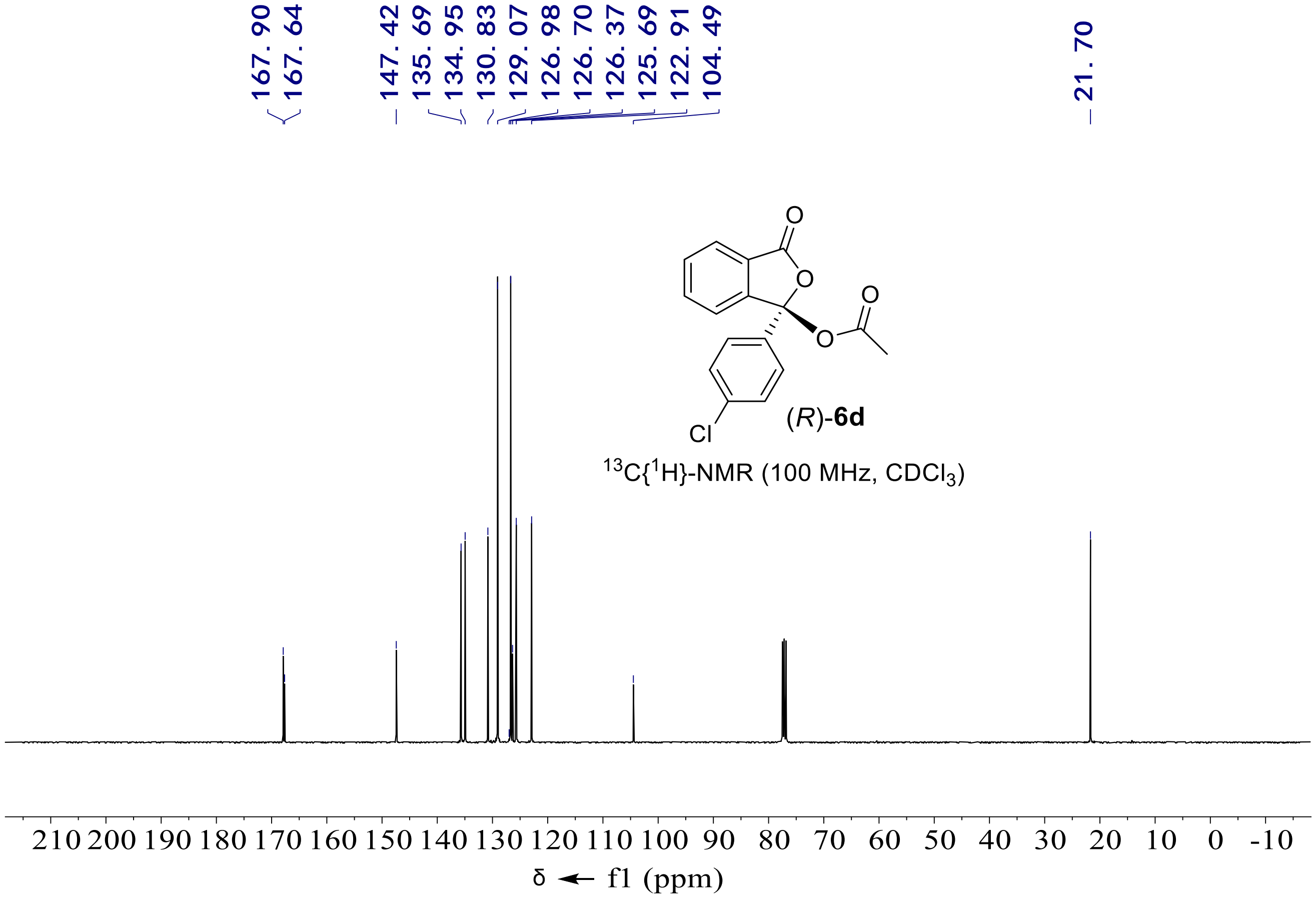

(*R*)-6e: (*R*)-1-(4-bromophenyl)-3-oxo-1,3-dihydroisobenzofuran-1-yl acetate.

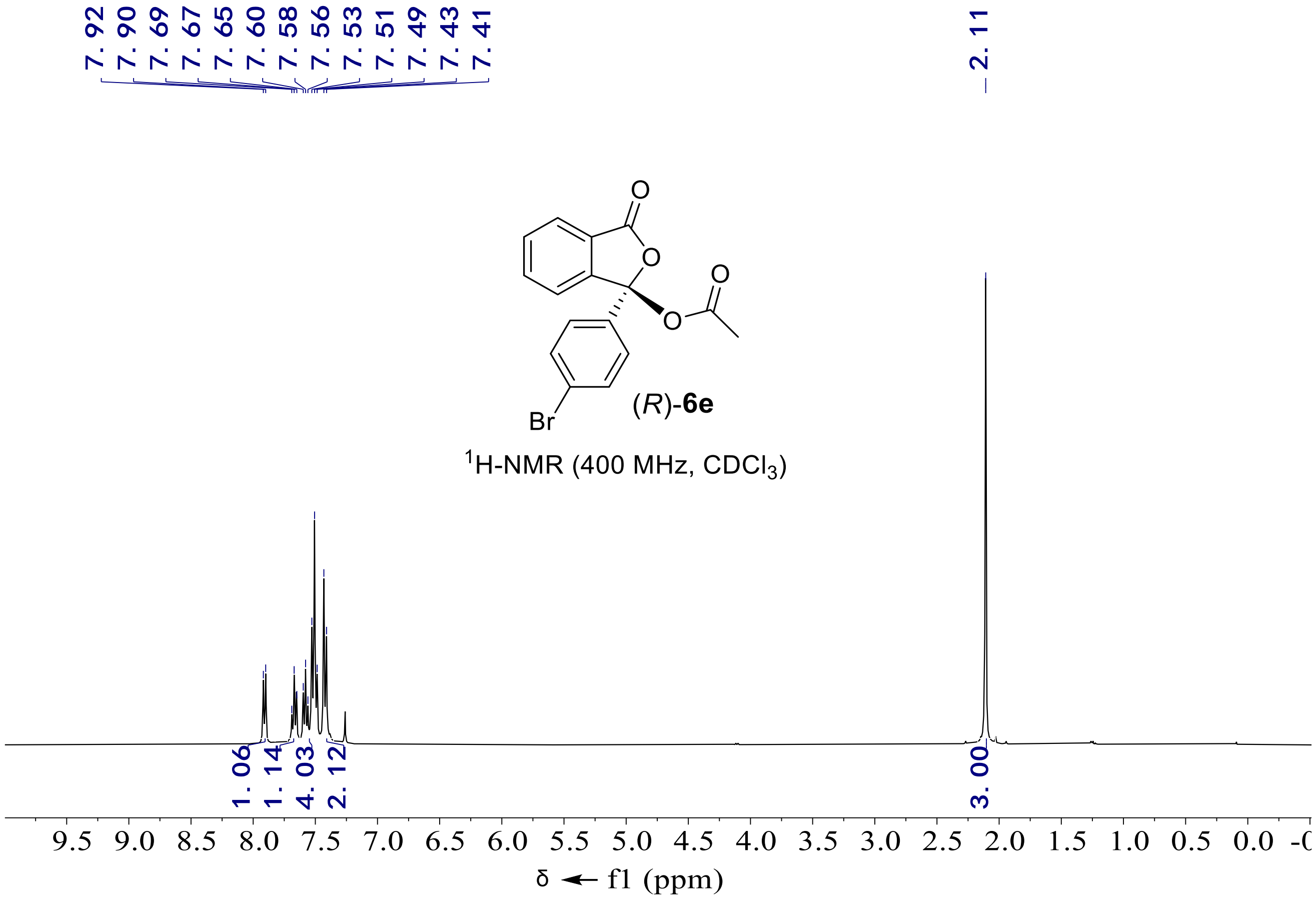

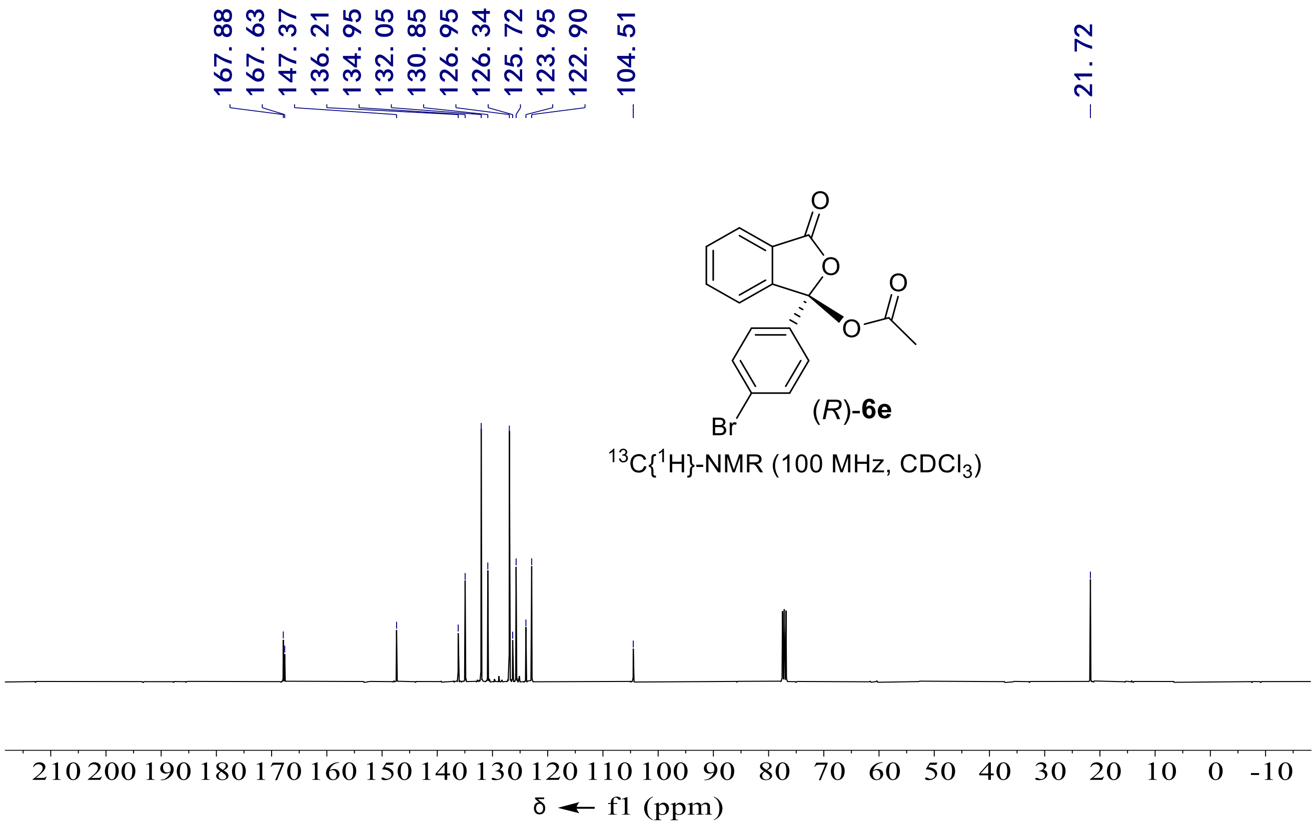

(*R*)-6f: (*R*)-3-oxo-1-(p-tolyl)-1,3-dihydroisobenzofuran-1-yl acetate.

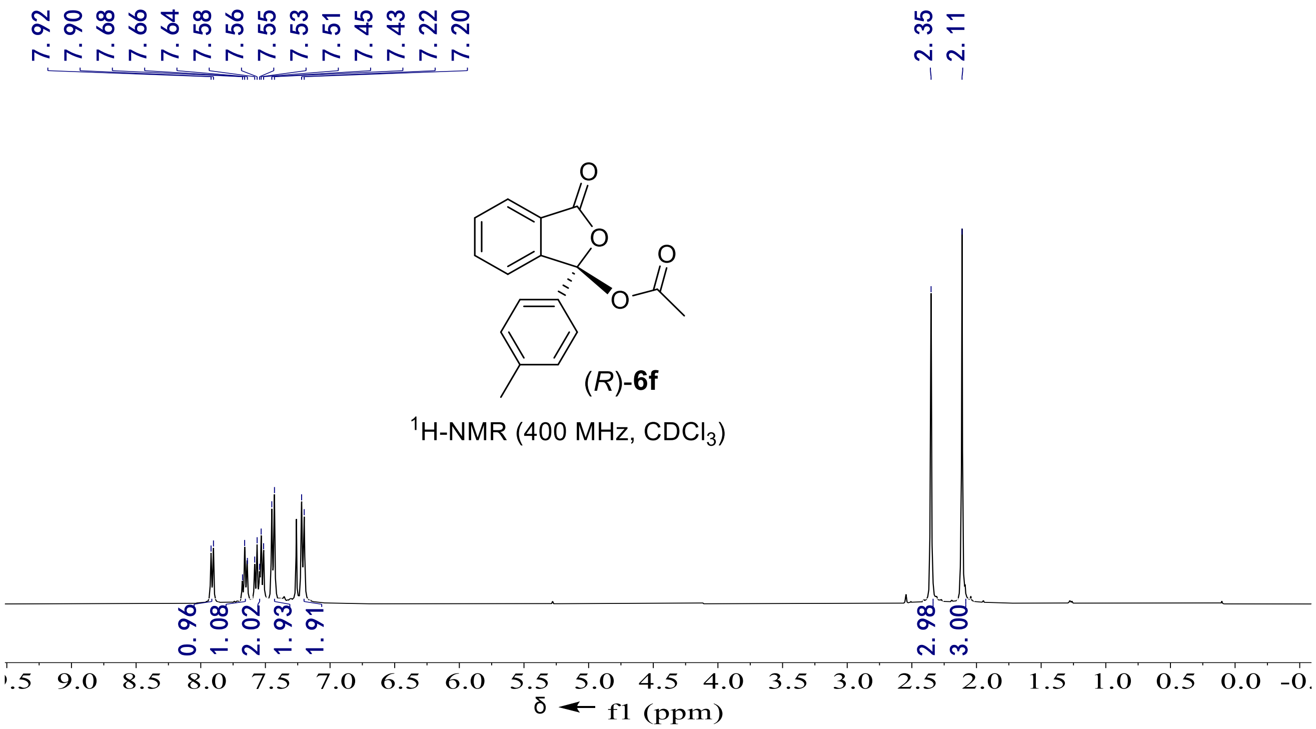

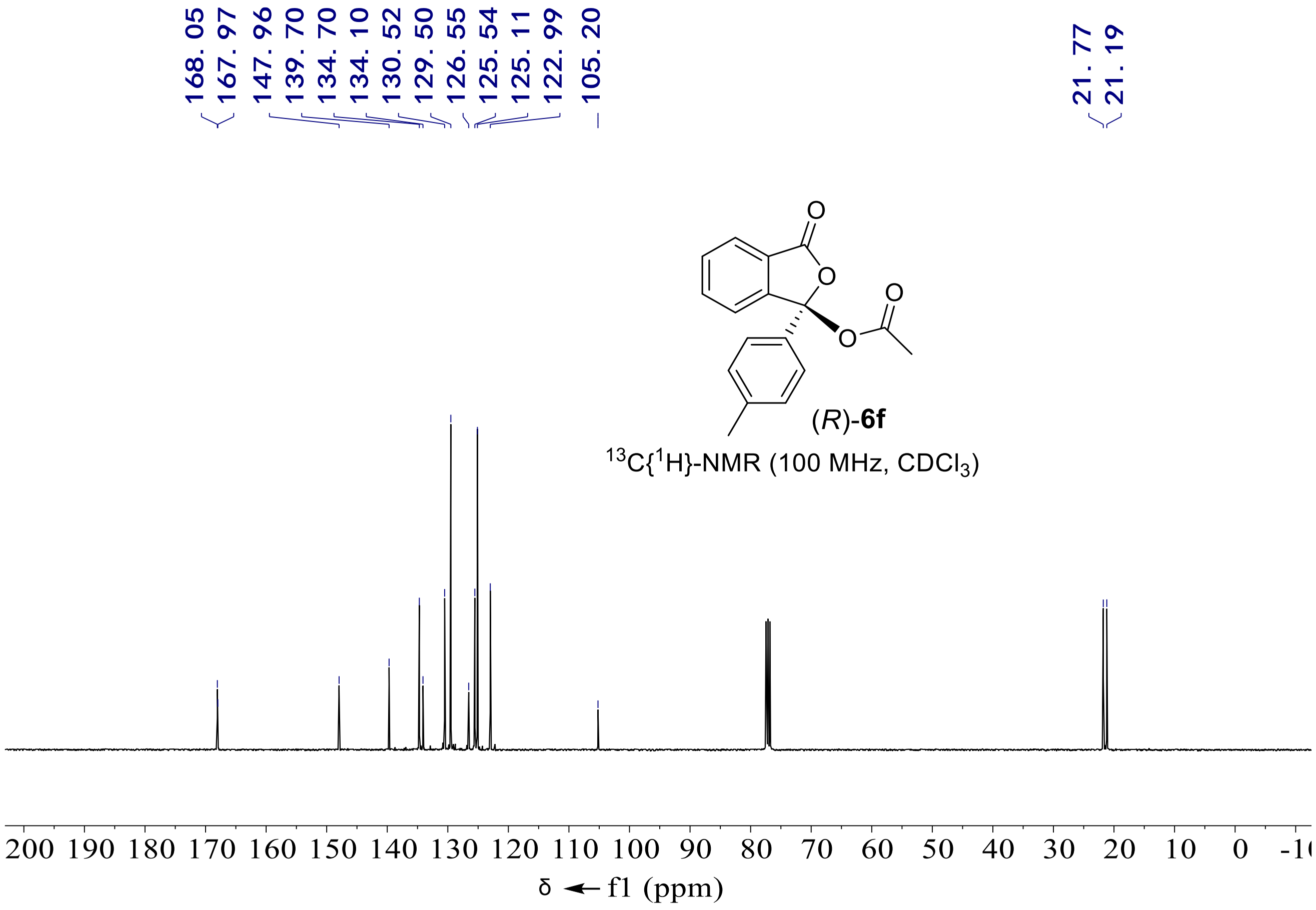

(***R***)**-6g:** (*R*)-1-(4-ethylphenyl)-3-oxo-1,3-dihydroisobenzofuran-1-yl acetate.

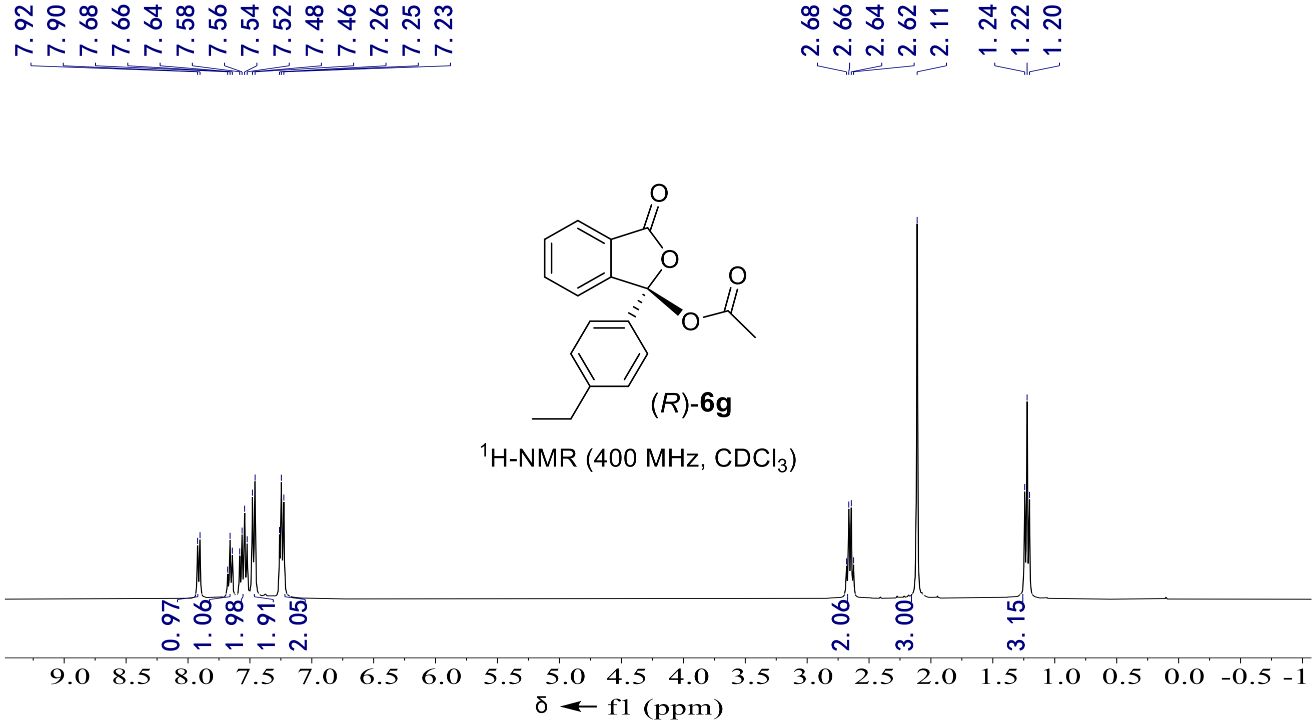

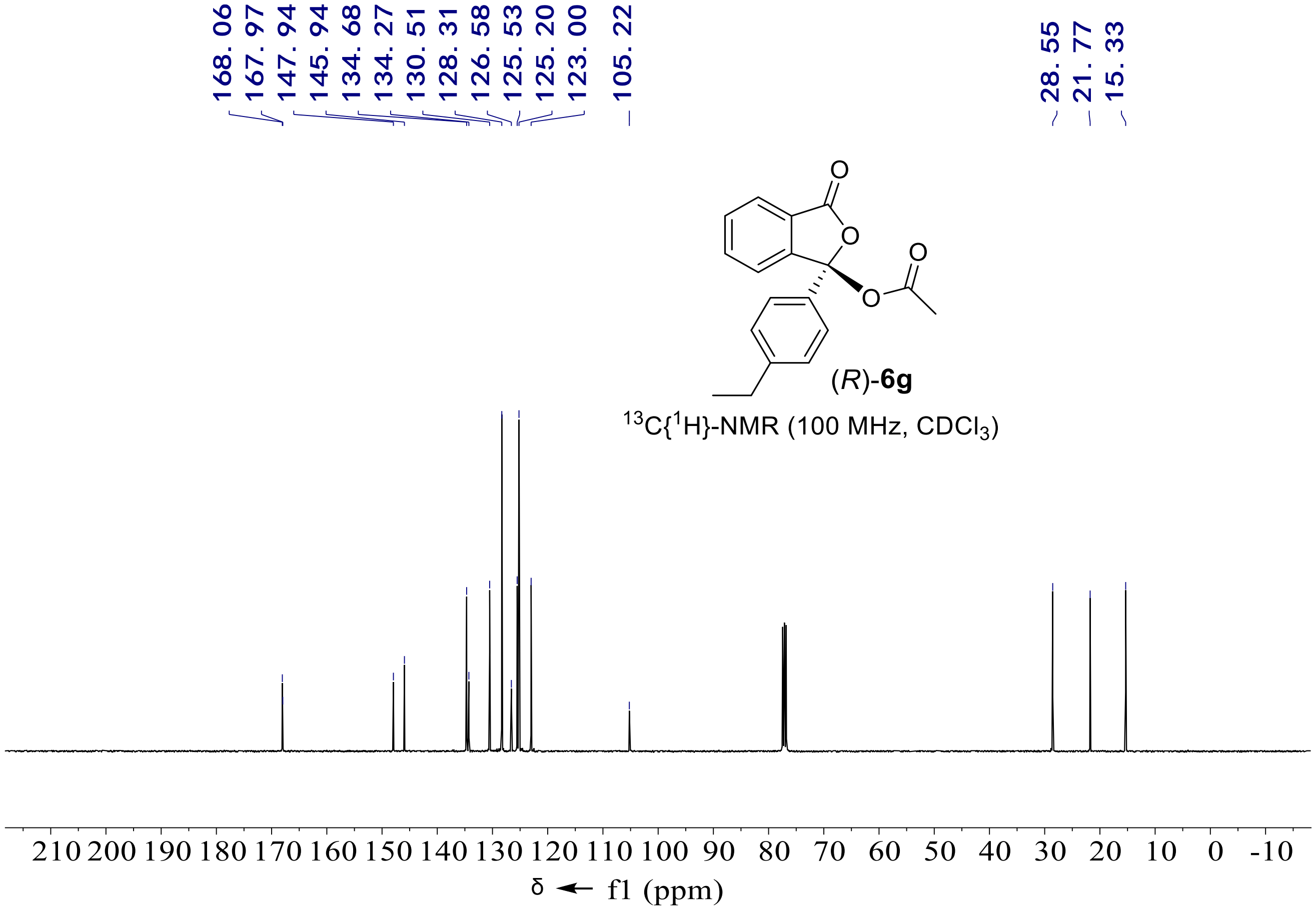

(*R*)-6h: (*R*)-3-oxo-1-(thiophen-2-yl)-1,3-dihydroisobenzofuran-1-yl acetate.

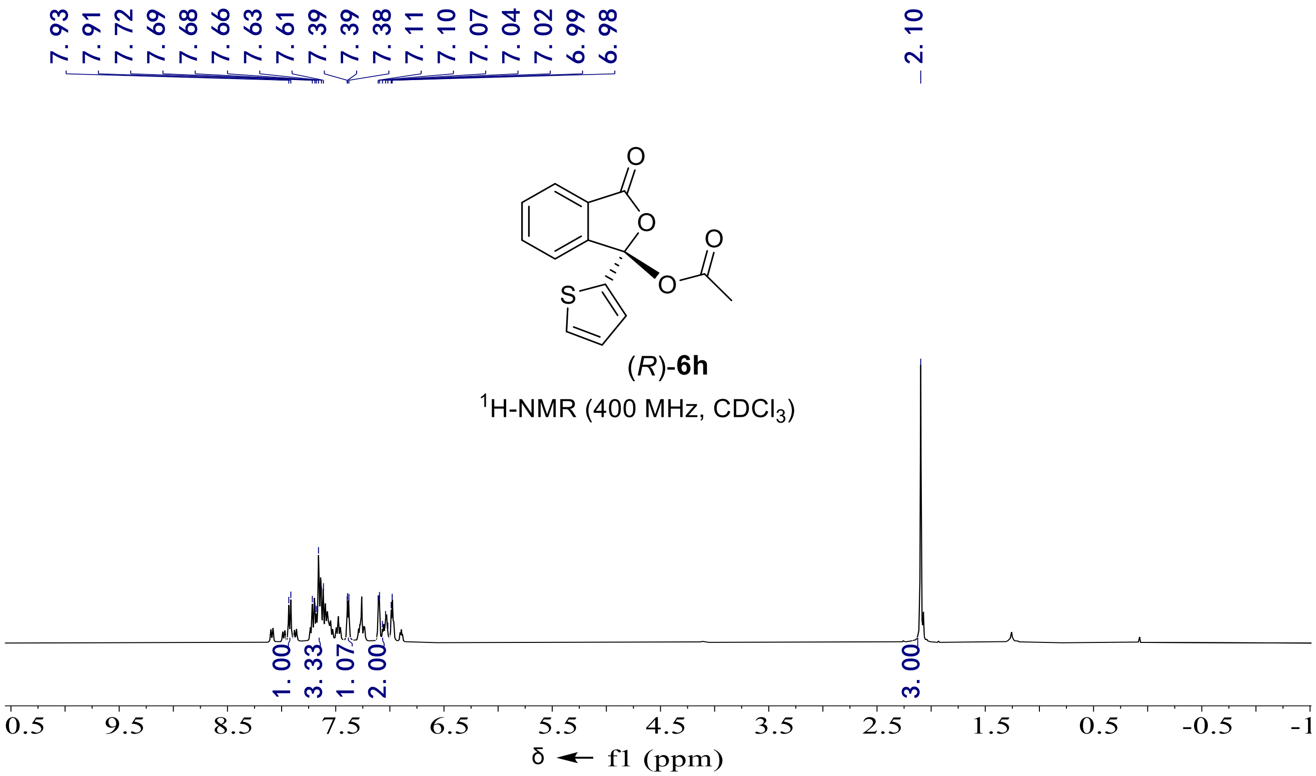

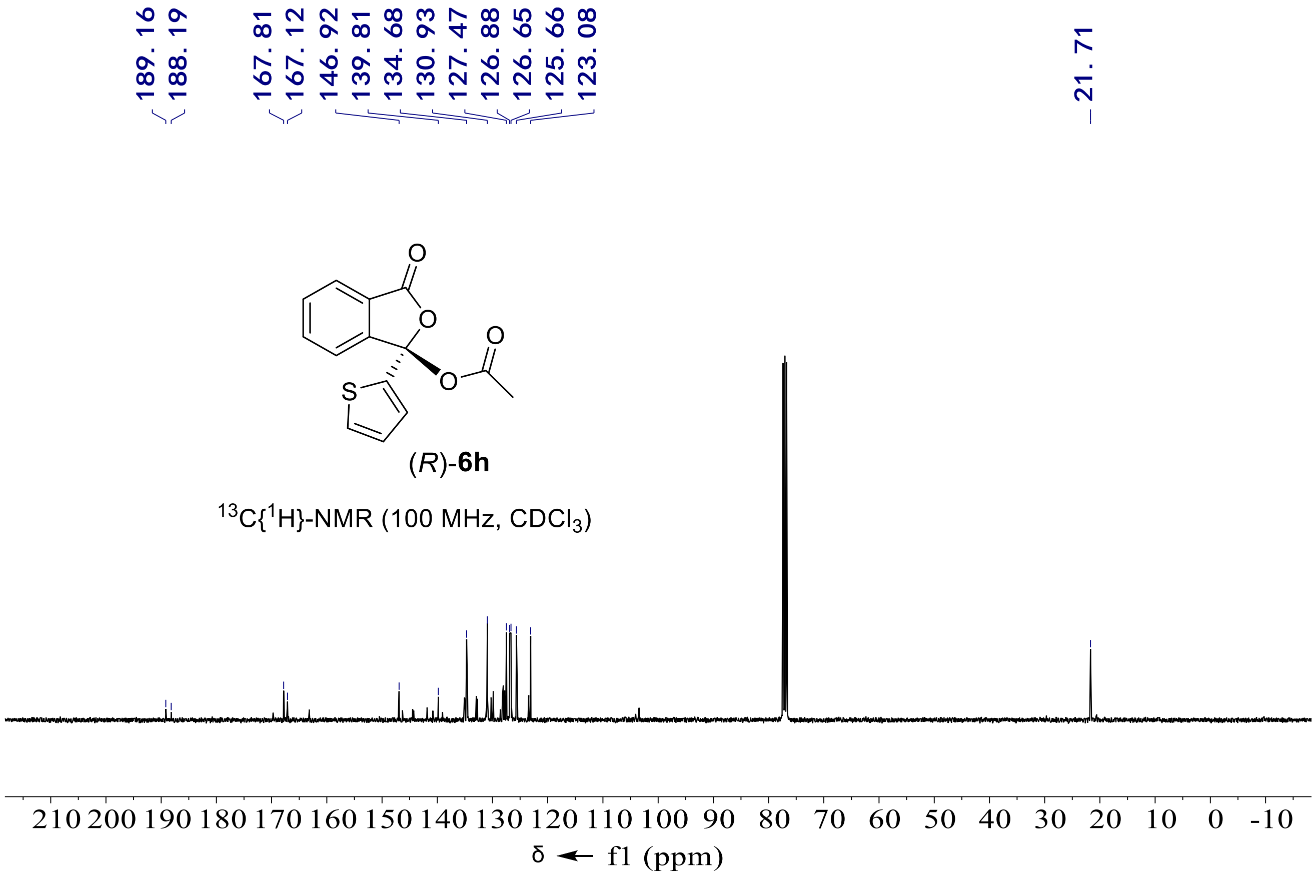

HPLC analyses of chiral products ((*R*)-3u-(*R*)-3w).

(*R*)-3u: (*R*)-3-oxo-1,3-dihydroisobenzofuran-1-yl 2-(1-(4-chlorobenzoyl)-5-methoxy-2-methyl-3a,7a-dihydro-1H-indol-3-yl)acetate. (HPLC: Chiralpak IC, detected at 210 nm, eluent: n-hexane/2-propanol = 95/5, flow rate = 1.0 mL/min, 25°C).

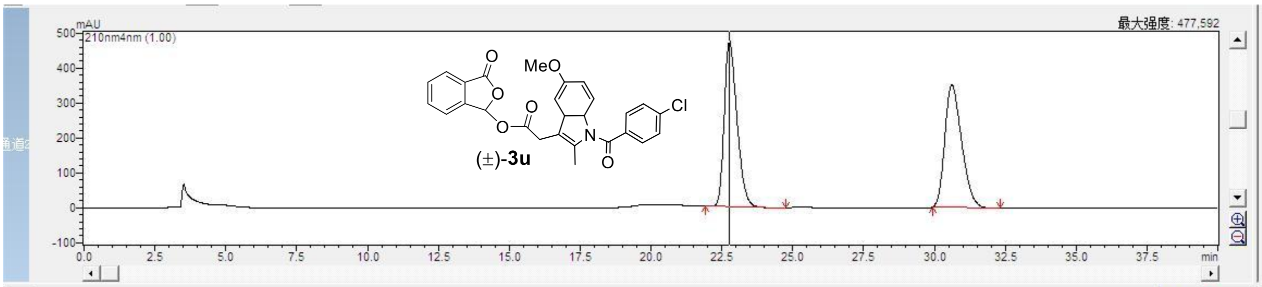

化合物表视图

ID#	名称	保留时间	峰#	面积	高度	面积%
1	RT22.764	22.764	1	14750053	474284	50.5061
2	RT30.608	30.608	2	14454429	350616	49.4939

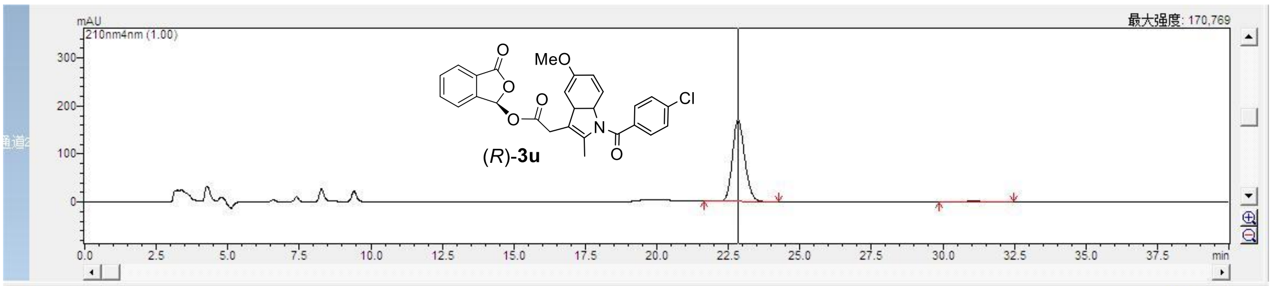

化合物表视图

ID#	名称	保留时间	峰#	面积	高度	面积%
1	RT22.836	22.836	1	5075417	169316	98.9488
2	RT30.699	30.699	2	53918	7493	1.0512

<u>**Translation of all characters (Chinese) in the above two frameworks to English is as follows:**</u>

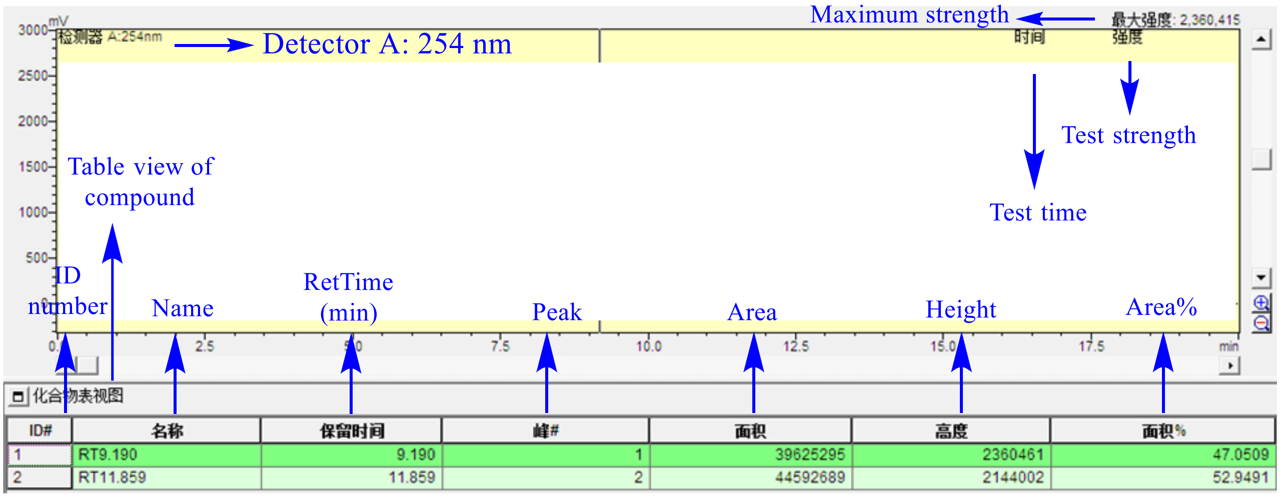

(*R*)-3v: (*R*)-3-oxo-1,3-dihydroisobenzofuran-1-yl 2-propylpentanoate. (HPLC: Chiralpak IC, detected at 210 nm, eluent: n-hexane/2-propanol = 80/20, flow rate = 1.0 mL/min, 25°C).

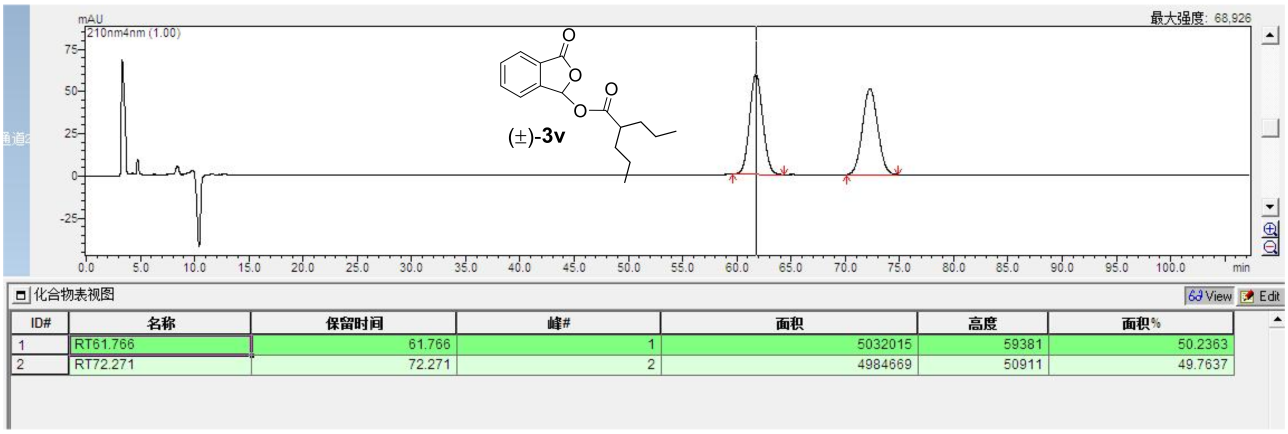

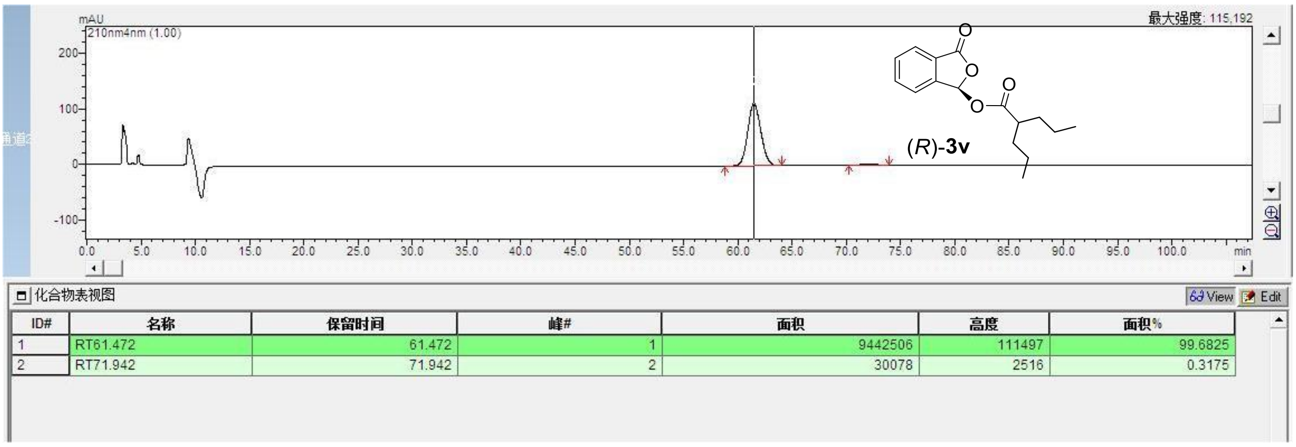

Translation of all characters (Chinese) in the above two frameworks to English is as follows:

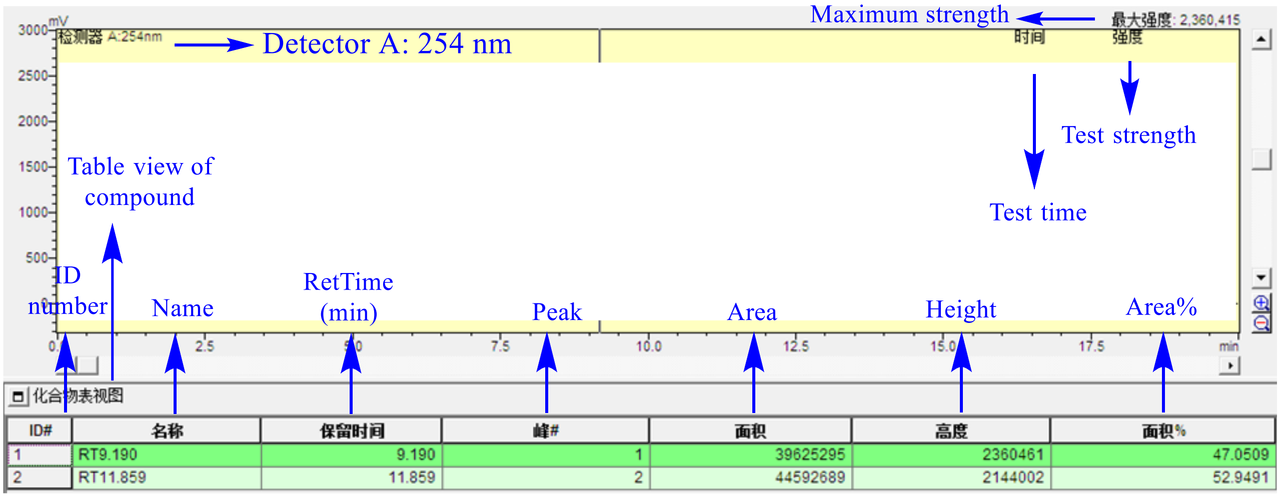

(*R*)-3w: (*R*)-3-oxo-1,3-dihydroisobenzofuran-1-yl 2-acetoxybenzoate. **(**HPLC: Chiralpak IC, detected at 210 nm, eluent: n-hexane/2-propanol = 80/20, flow rate = 1.0 mL/min, 25°C).

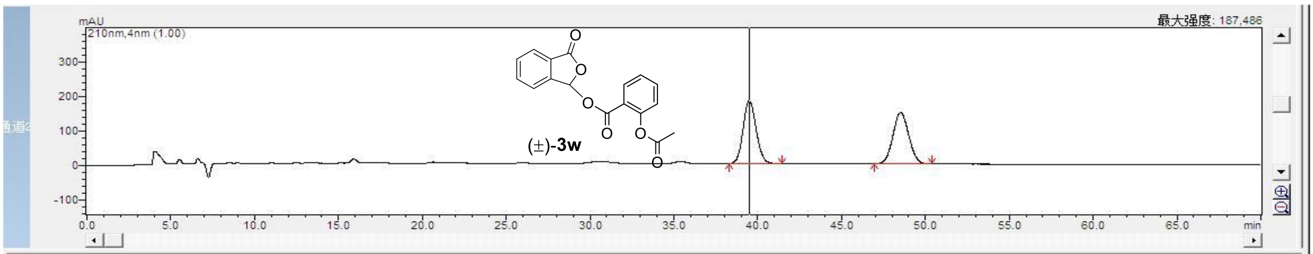

化合物表视图

ID#	名称	保留时间	峰#	面积	高度	面积%
1	RT39.489	39.489	1	9851979	182436	50.1449
2	RT48.514	48.514	2	9795049	149669	49.8551

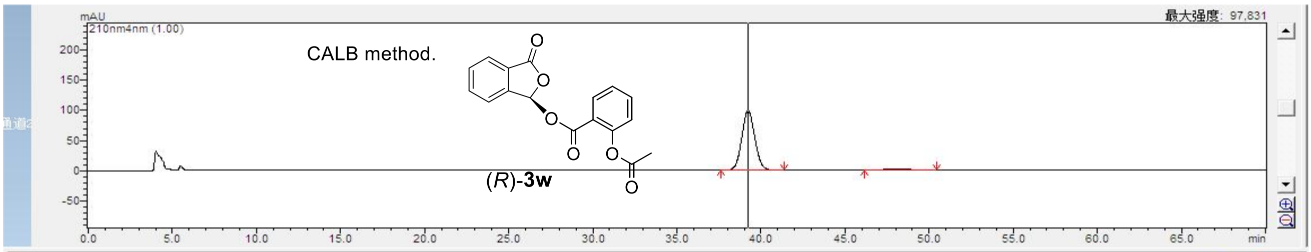

化合物表视图

ID#	名称	保留时间	峰#	面积	高度	面积%
1	RT39.220	39.220	1	5289993	98505	99.6266
2	RT48.108	48.108	2	19826	1735	0.3734

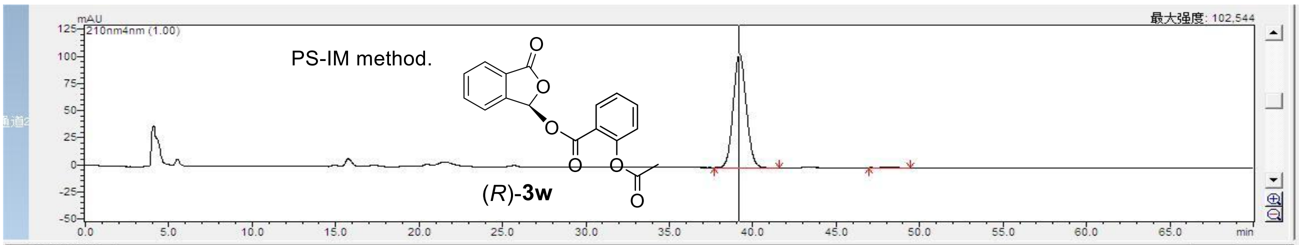

化合物表视图

ID#	名称	保留时间	峰#	面积	高度	面积%
1	RT39.198	39.198	1	5690835	105287	99.1619
2	RT48.171	48.171	2	48099	4185	0.8381

<u>Translation of all characters (Chinese) in the above two frameworks to English is as follows:</u>

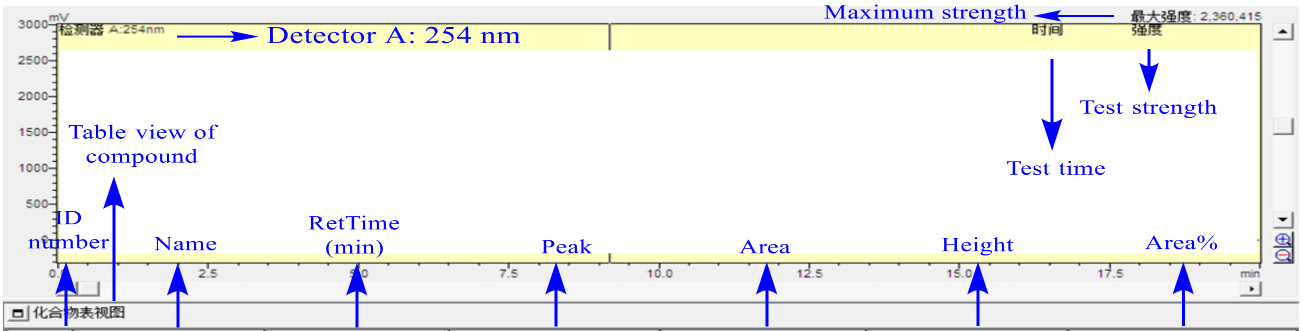

ID#	名称	保留时间	峰#	面积	高度	面积%
1	RT9.190	9.190	1	39625295	2360461	47.0509
2	RT11.859	11.859	2	44592689	2144002	52.9491

(*R*)-3x: (*R*)-5-oxo-2,5-dihydrofuran-2-yl acetate (HPLC: Chiralpak OJ-H, detected at 210 nm, eluent: n-hexane/2-propanol = 95/5, flow rate = 1.0 mL/min, 25°C).

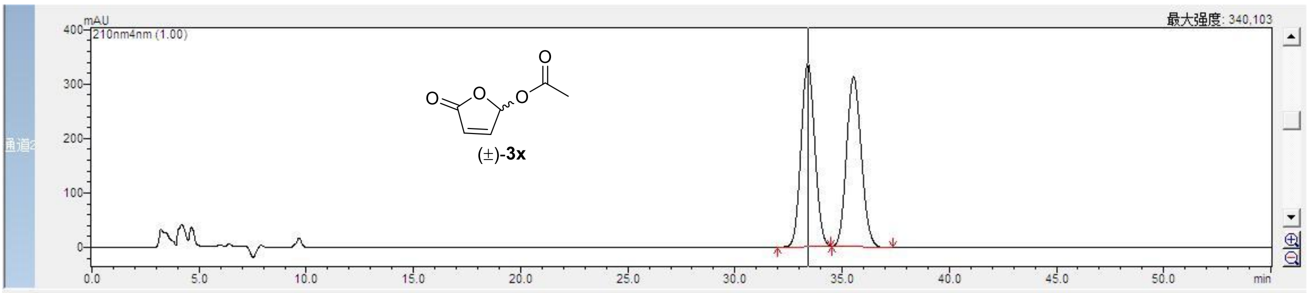

化合物表视图

ID#	名称	保留时间	峰#	面积	高度	面积%
1	RT33.389	33.389	1	14924001	336473	49.8868
2	RT35.523	35.523	2	14991755	312528	50.1132

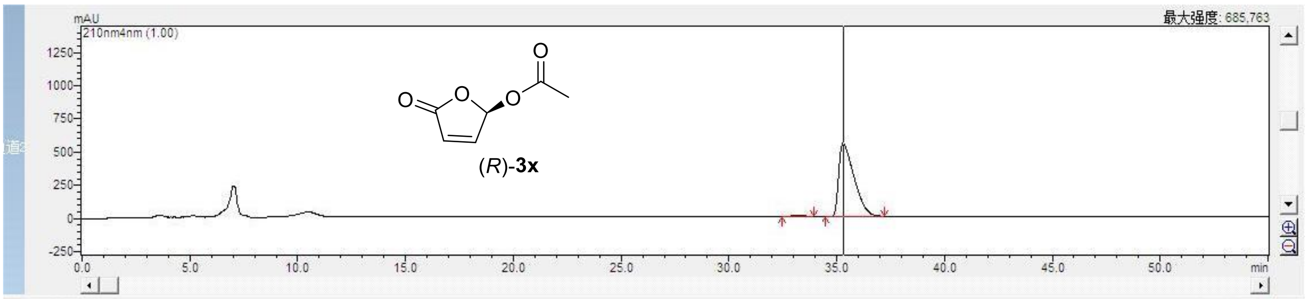

化合物表视图

ID#	名称	保留时间	峰#	面积	高度	面积%
1	RT24.718	33.219	1	69642	16592	0.2551
2	RT29.119	35.310	2	25920721	555046	99.7449

Translation of all characters (Chinese) in the above two frameworks to English is as follows:

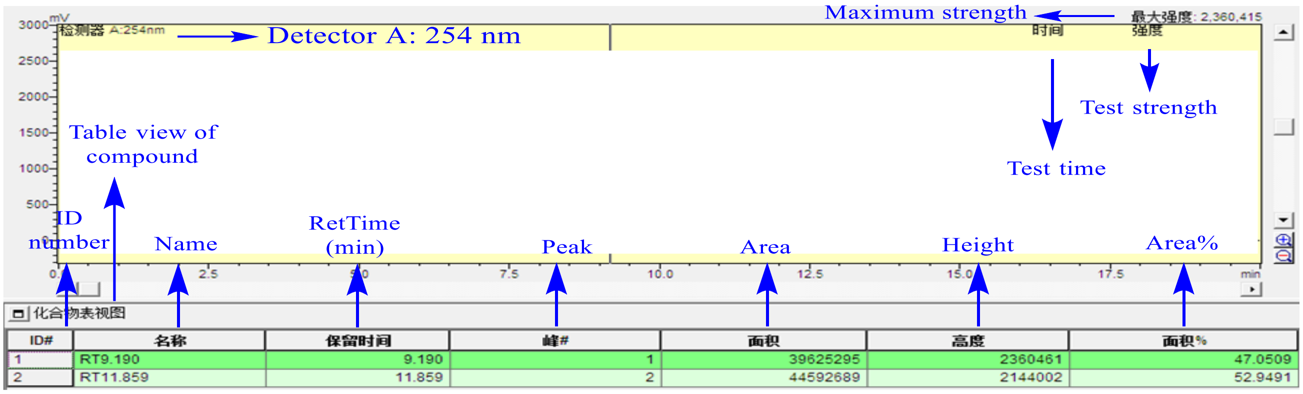

ID#	名称	保留时间	峰#	面积	高度	面积%
1	RT9.190	9.190	1	39625295	2360461	47.0509
2	RT11.859	11.859	2	44592689	2144002	52.9491

(*R*)-3y: (*R*)-chroman-2-yl acetate (HPLC: Chiracel IC, detected at 210 nm, eluent: n-hexane/2-propanol = 98/02, flow rate = 1.0 mL/min, 25°C).

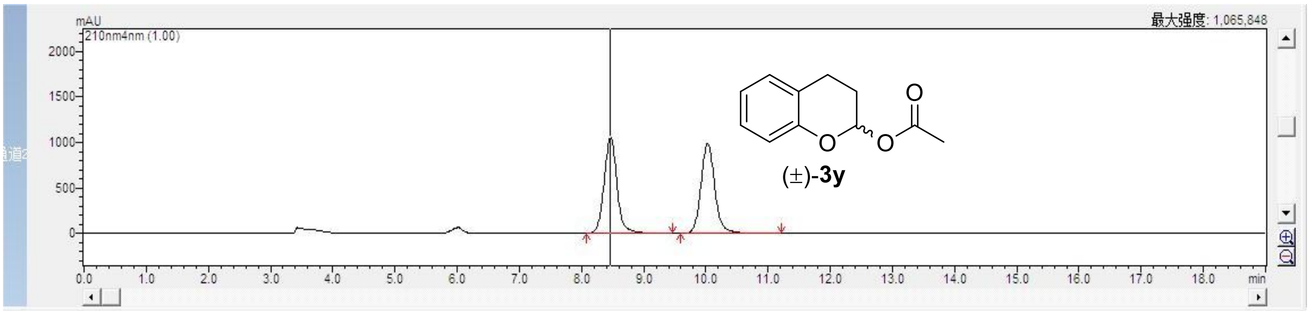

化合物表视图

ID#	名称	保留时间	峰#	面积	高度	面积%
1	RT8.463	8.463	1	15231301	1065069	49.8659
2	RT10.022	10.022	2	15313195	992921	50.1341

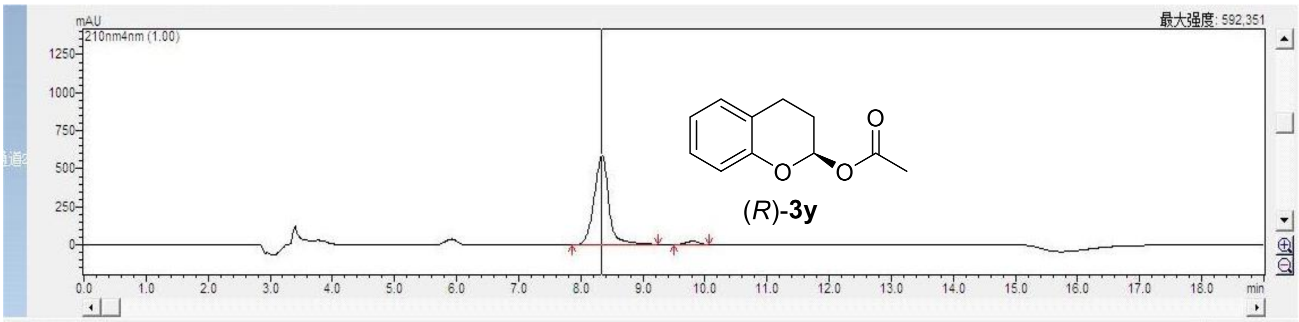

化合物表视图

ID#	名称	保留时间	峰#	面积	高度	面积%
1	RT8.337	8.337	1	9638561	591573	98.9540
2	RT9.816	9.816	2	101886	11897	1.0460

Translation of all characters (Chinese) in the above two frameworks to English is as follows:

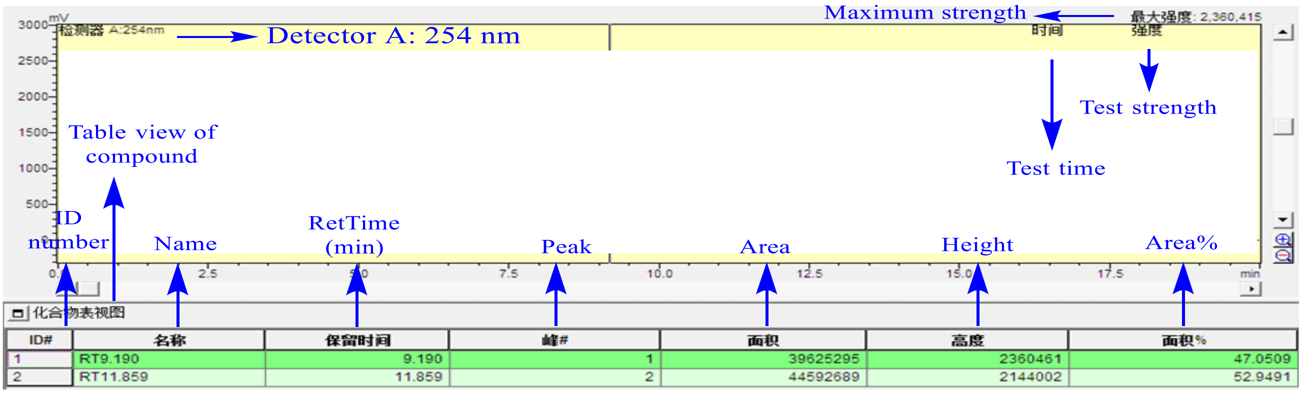

(***R***)**-3z: (** (***R***)**-6-hydroxy-3-oxo-1,3-dihydroisobenzofuran-1-yl acetate** (HPLC: Chiralpak IC, detected at 210 nm, eluent: n-hexane/2-propanol = 90/10, flow rate = 1.0 mL/min, 25°C).

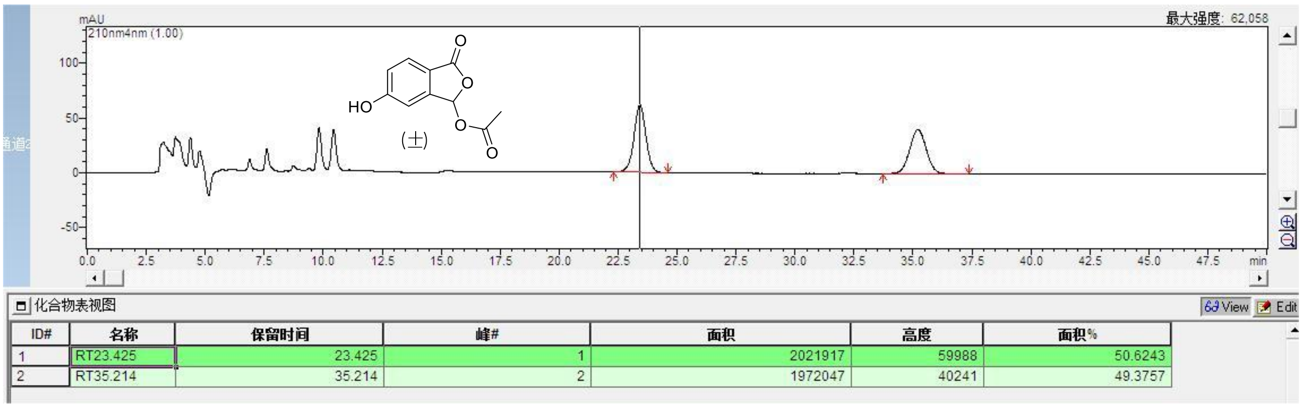

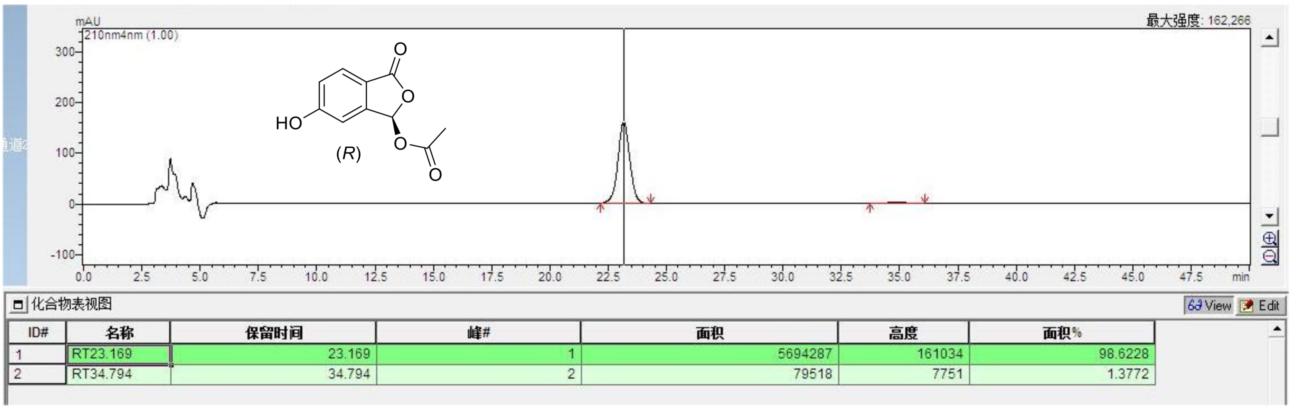

Translation of all characters (Chinese) in the above two frameworks to English is as follows:

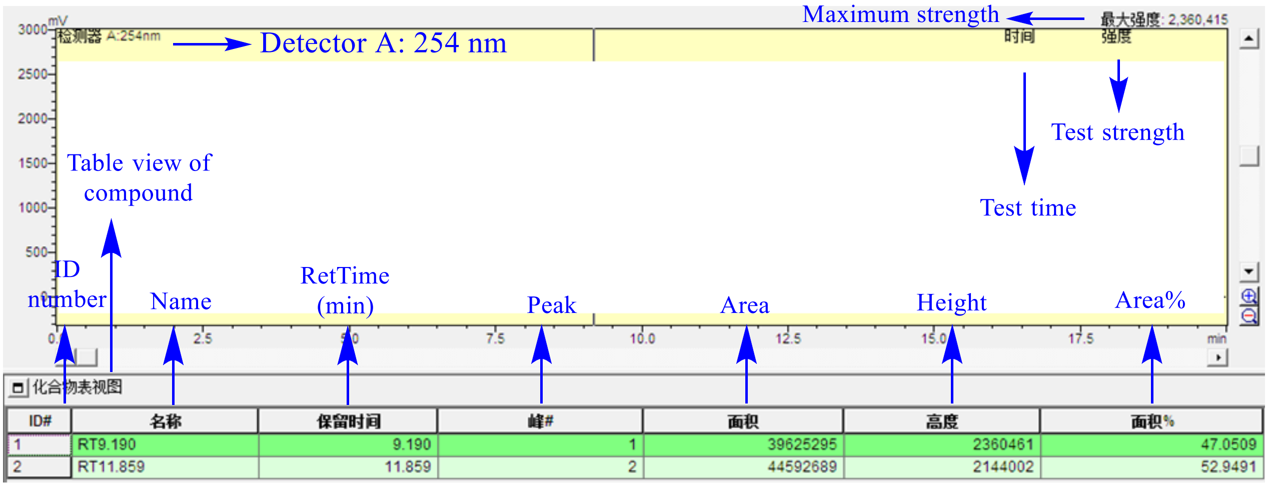

(*R*)-7: (*R*)-3-oxo-6-phenoxy-1,3-dihydroisobenzofuran-1-yl acetate.

(*R*)-3-oxo-6-phenoxy-1,3-dihydroisobenzofuran-1-yl acetate (HPLC: Chiralpak IC, detected at 210 nm, eluent: n-hexane/2-propanol = 90/10, flow rate = 1.0 mL/min, 25°C).

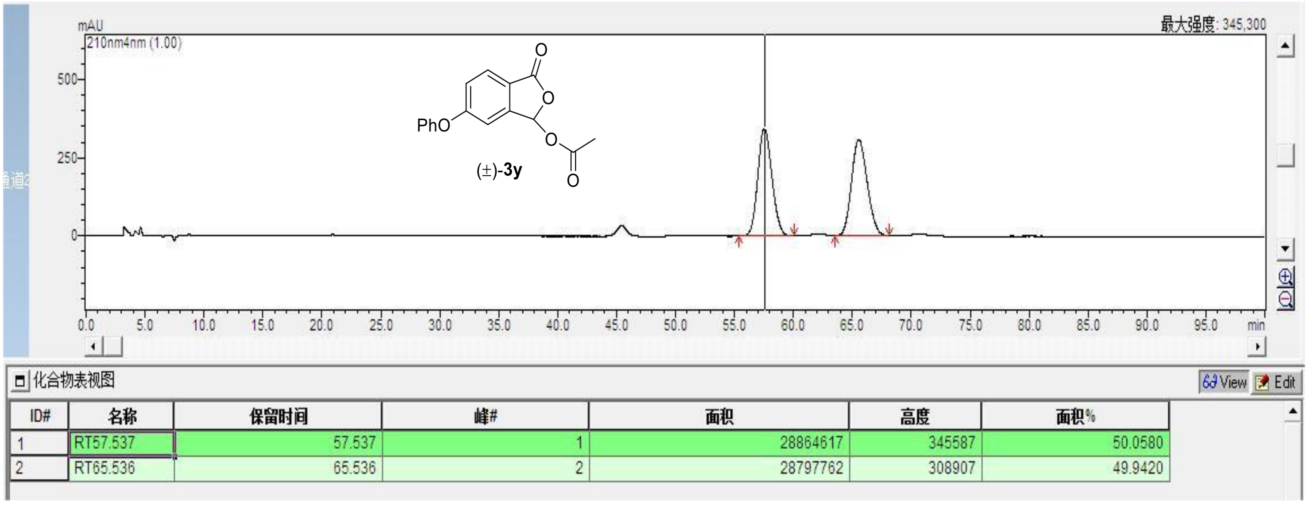

ID#	名称	保留时间	峰#	面积	高度	面积%
1	RT57.537	57.537	1	28864617	345587	50.0580
2	RT65.536	65.536	2	28797762	308907	49.9420

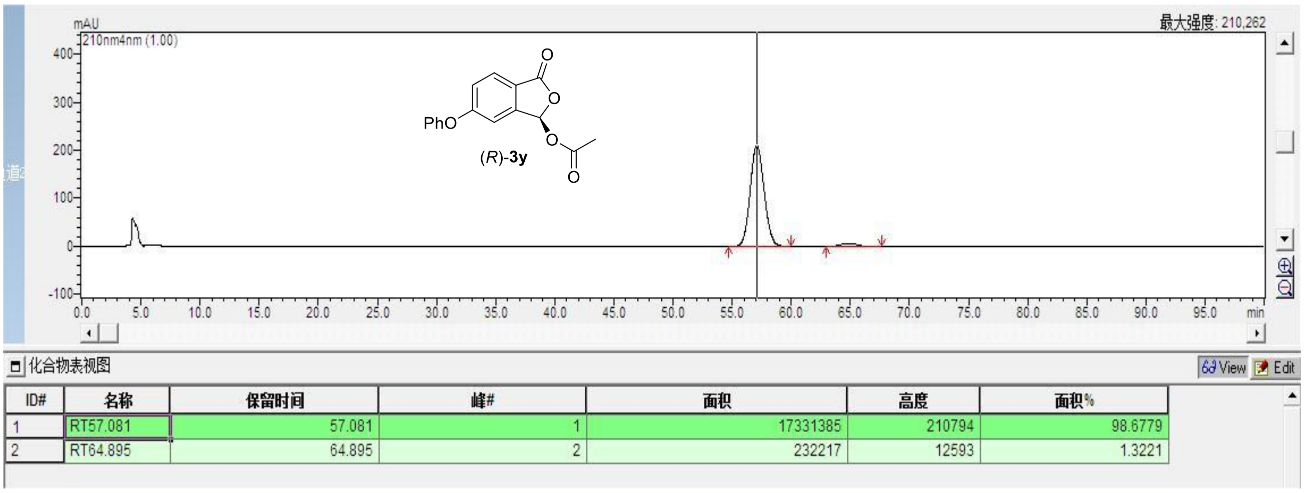

ID#	名称	保留时间	峰#	面积	高度	面积%
1	RT57.081	57.081	1	17331385	210794	98.6779
2	RT64.895	64.895	2	232217	12593	1.3221

<u>Translation of all characters (Chinese) in the above two frameworks to English is as follows:</u>

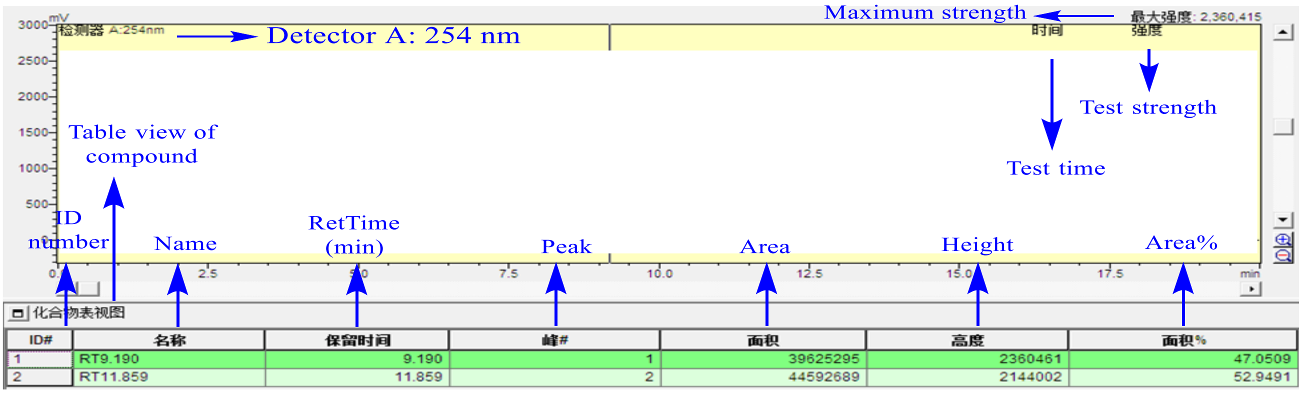

ID#	名称	保留时间	峰#	面积	高度	面积%
1	RT9.190	9.190	1	39625295	2360461	47.0509
2	RT11.859	11.859	2	44592689	2144002	52.9491

(*R*)-8: (*R*)-1-([1,1'-biphenyl]-4-yl)-3-oxo-1,3-dihydroisobenzofuran-1-yl acetate.

(*R*)-1-([1,1'-biphenyl]-4-yl)-3-oxo-1,3-dihydroisobenzofuran-1-yl acetate (HPLC: Chiralpak IC, detected at 210 nm, eluent: n-hexane/2-propanol = 90/10, flow rate = 1.0 mL/min, 25°C).

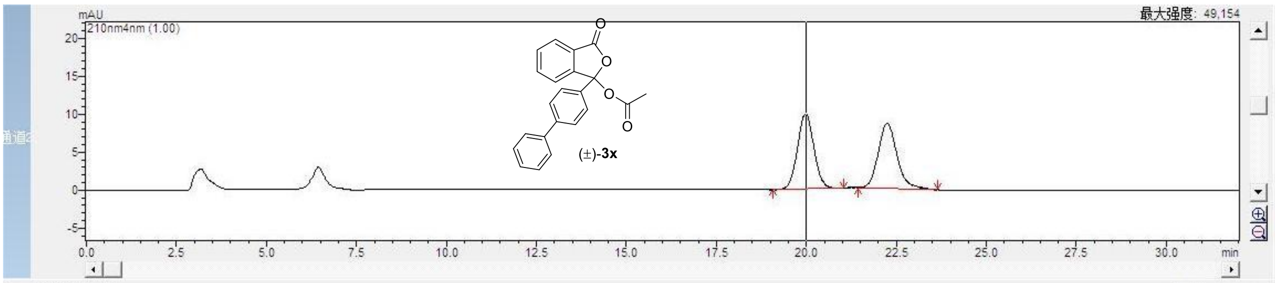

化合物表视图

ID#	名称	保留时间	峰#	面积	高度	面积%
1	RT19.986	19.986	1	312455	9875	49.9818
2	RT22.230	22.230	2	312682	8578	50.0182

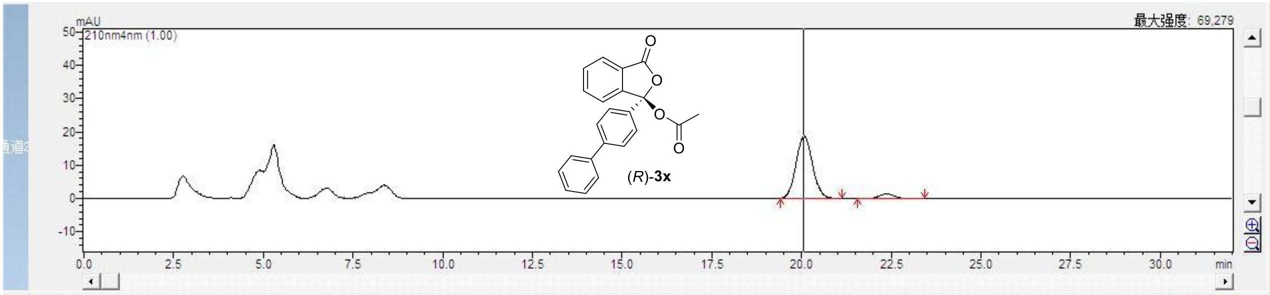

化合物表视图

ID#	名称	保留时间	峰#	面积	高度	面积%
1	RT20.064	20.064	1	598824	18726	96.9849
2	RT22.330	22.330	2	18617	1964	3.0151

<u>Translation of all characters (Chinese) in the above two frameworks to English is as follows:</u>

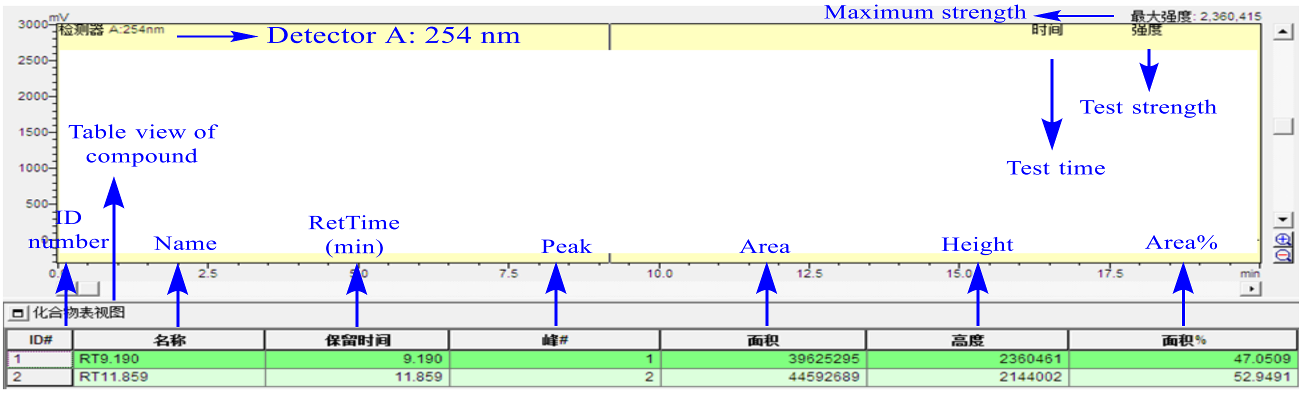

Characterizations of chiral products ((*R*)-3u-(*R*)-3w).

(*R*)-3u: (*R*)-3-oxo-1,3-dihydroisobenzofuran-1-yl 2-(1-(4-chlorobenzoyl)-5-methoxy-2-methyl-3a,7a-dihydro-1H-indol-3-yl)acetate.

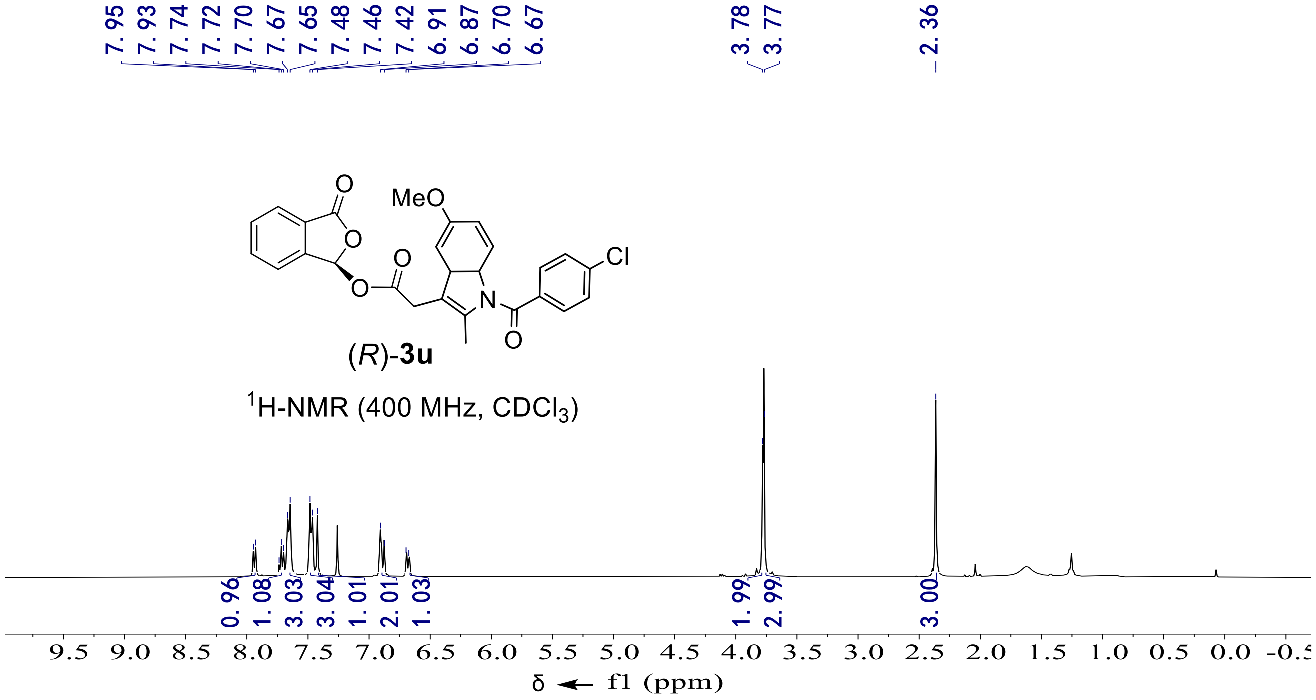

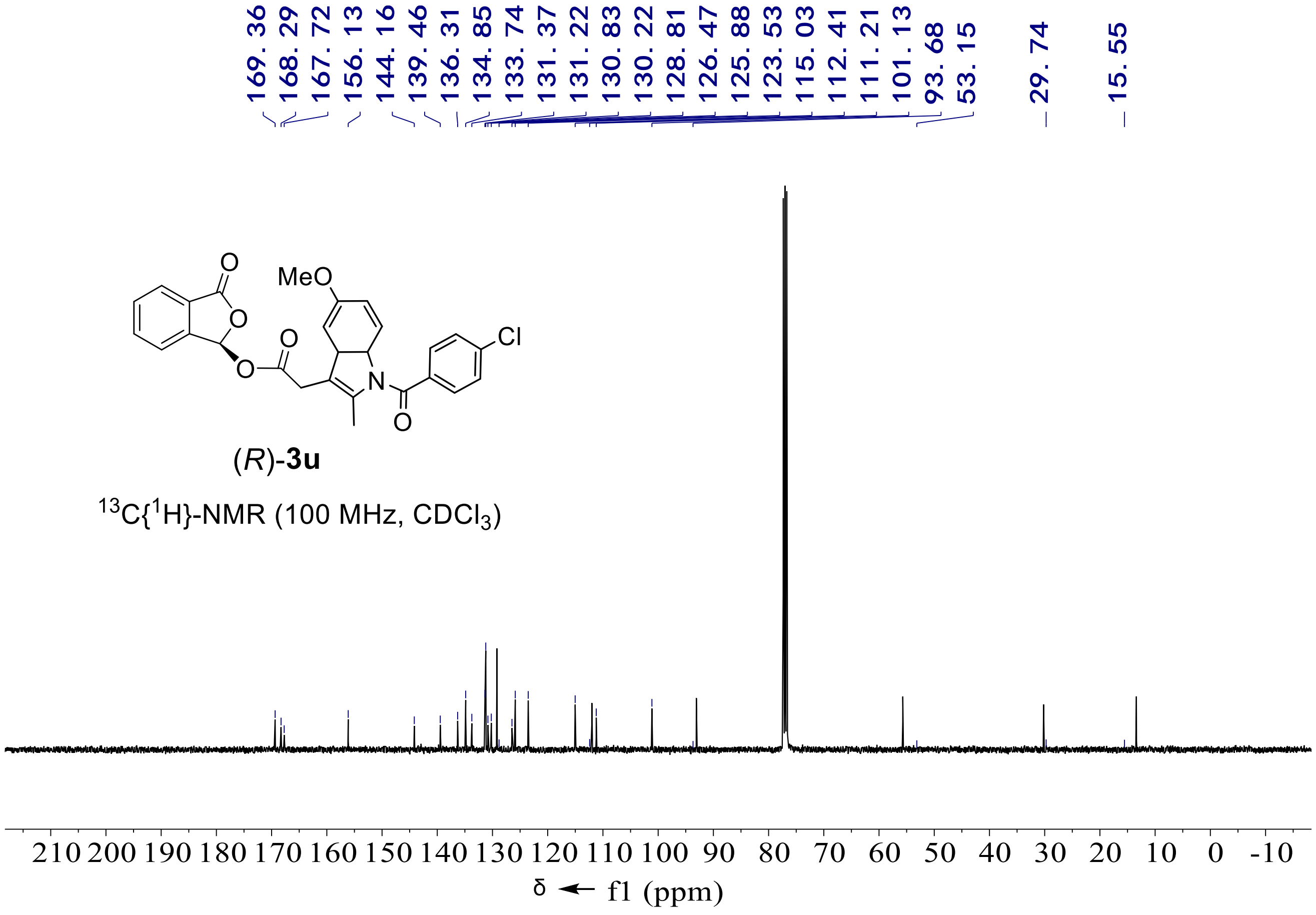

(*R*)-3v: (*R*)-3-oxo-1,3-dihydroisobenzofuran-1-yl 2-propylpentanoate.

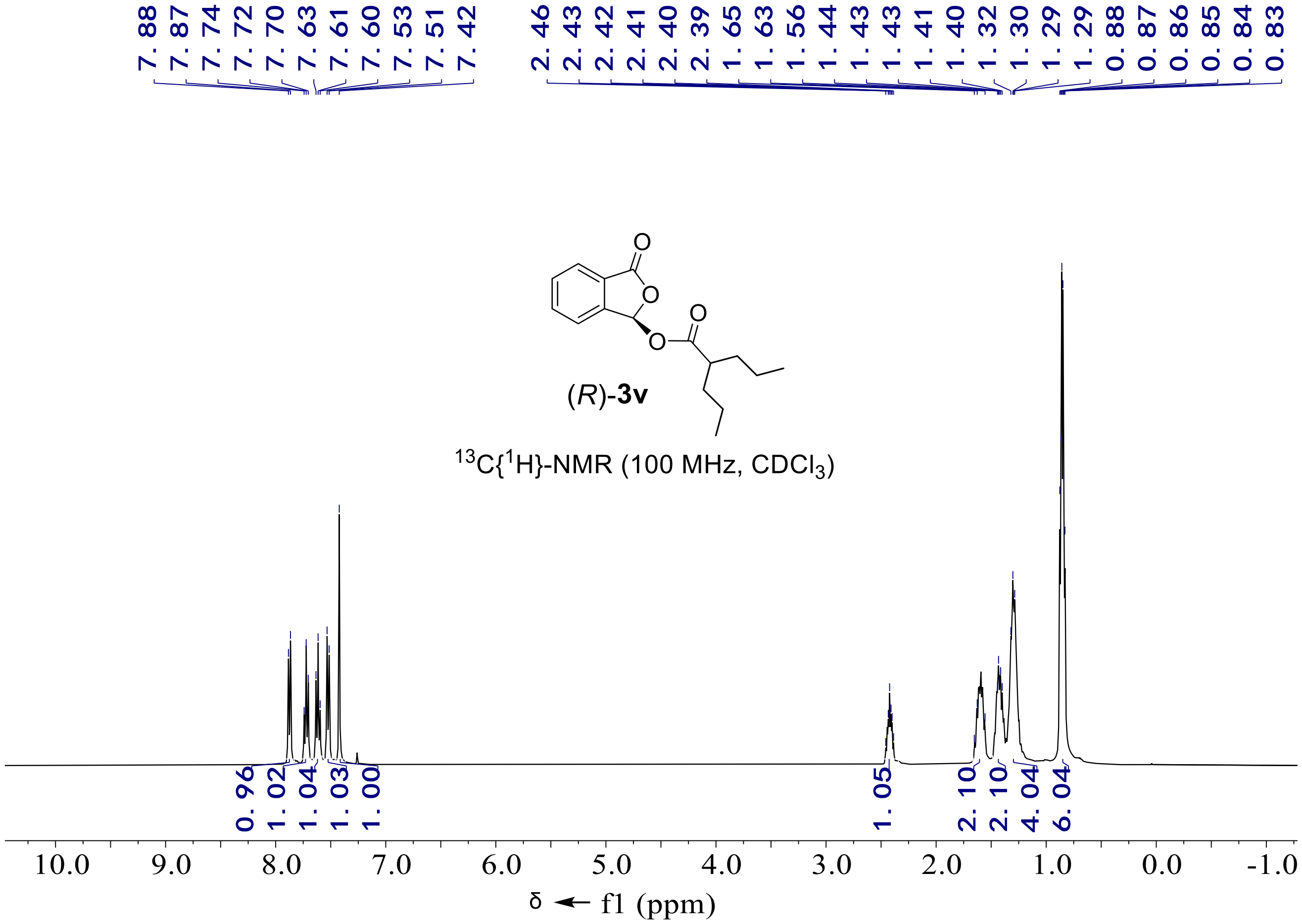

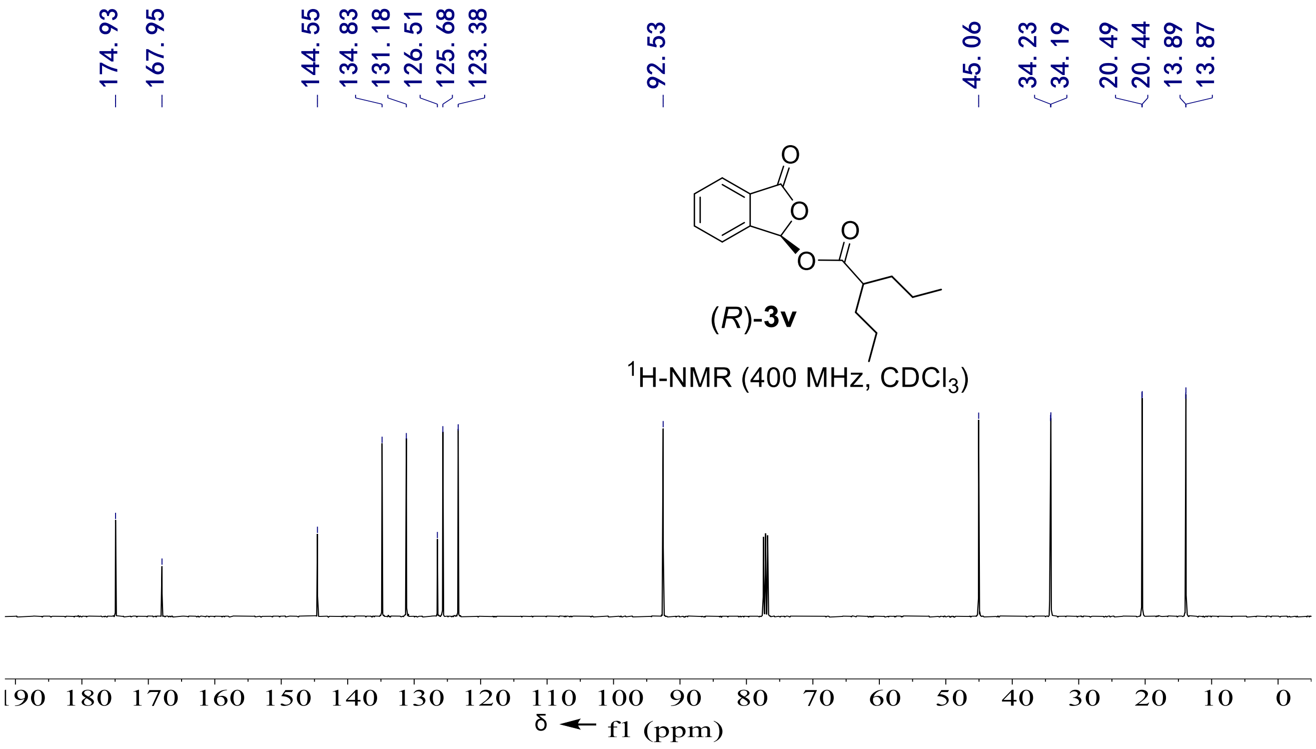

(*R*)-3w: (*R*)-3-oxo-1,3-dihydroisobenzofuran-1-yl 2-acetoxybenzoate.

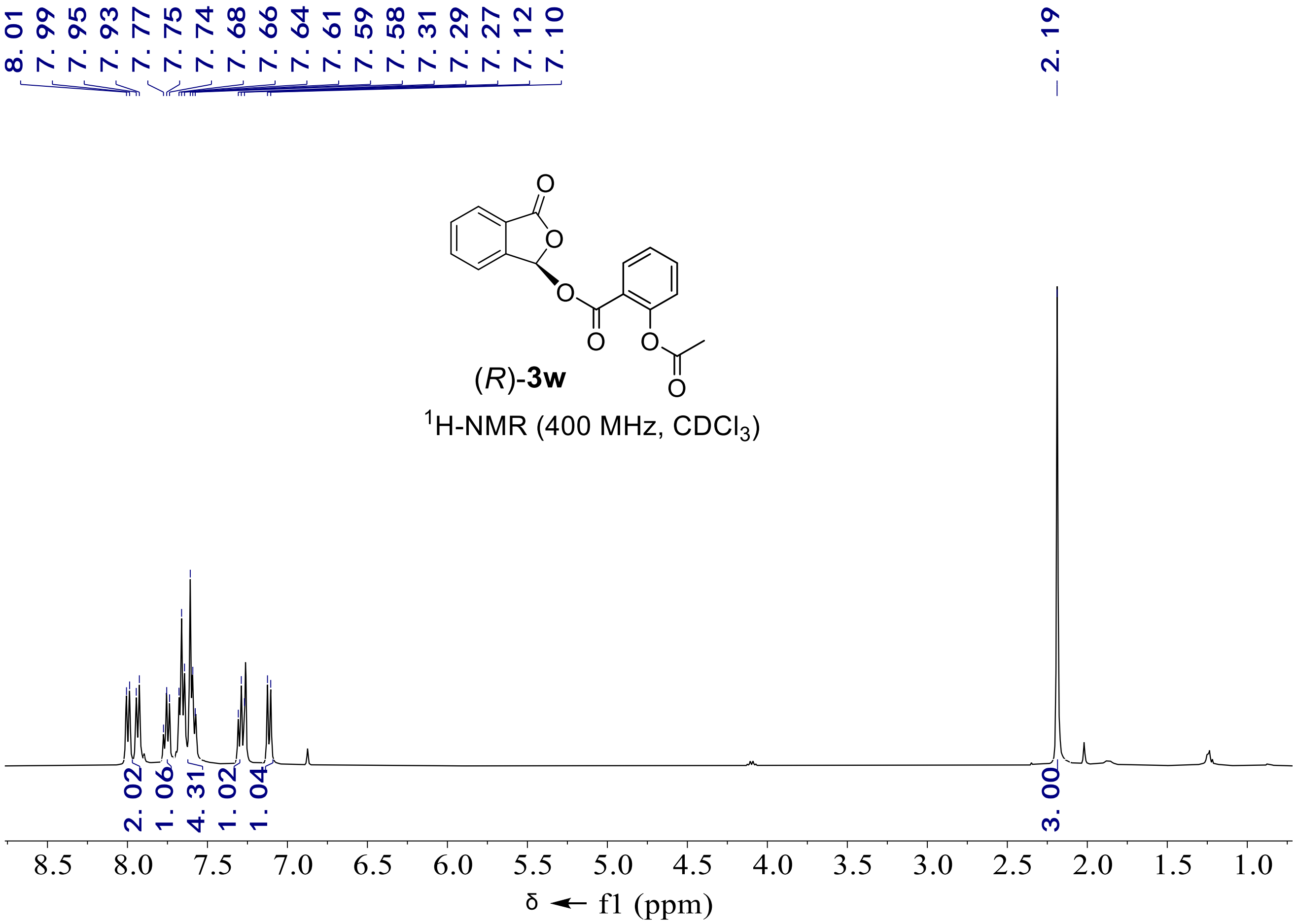

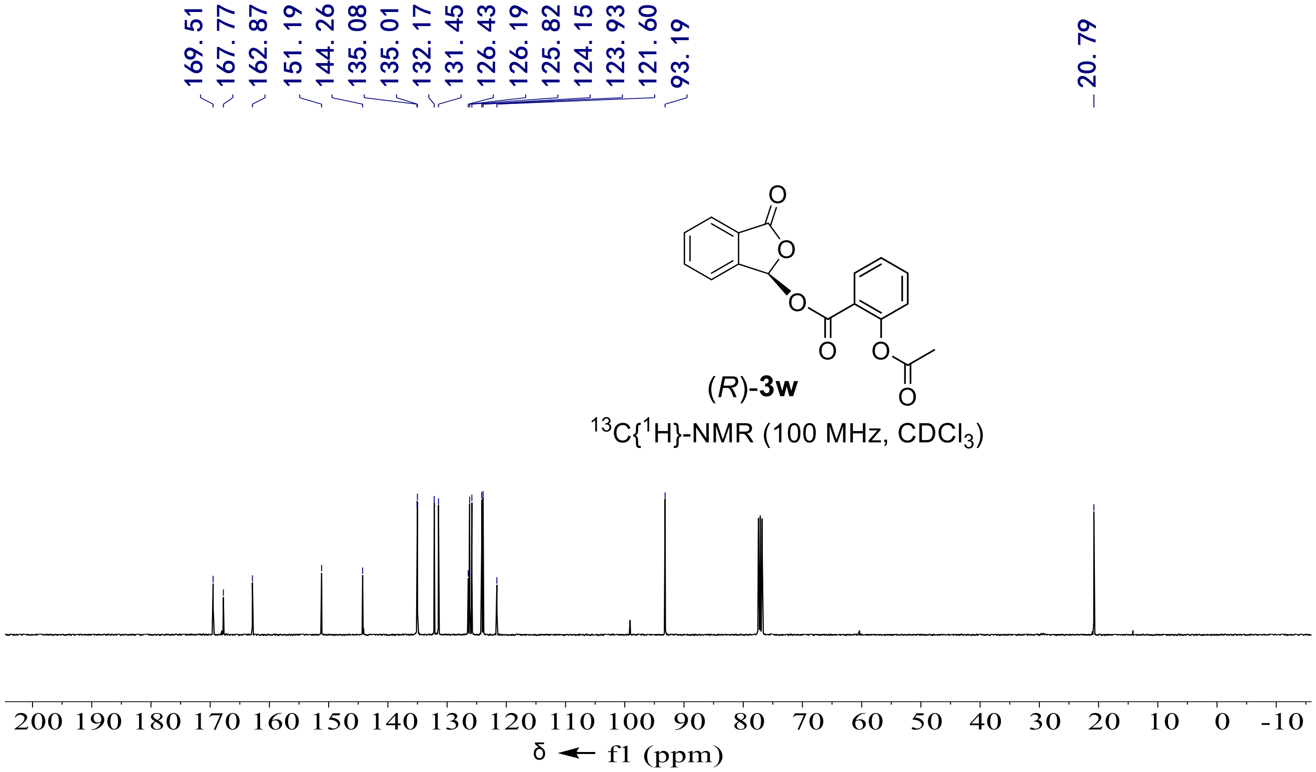

(*R*)-3x: (*R*)-5-oxo-2,5-dihydrofuran-2-yl acetate.

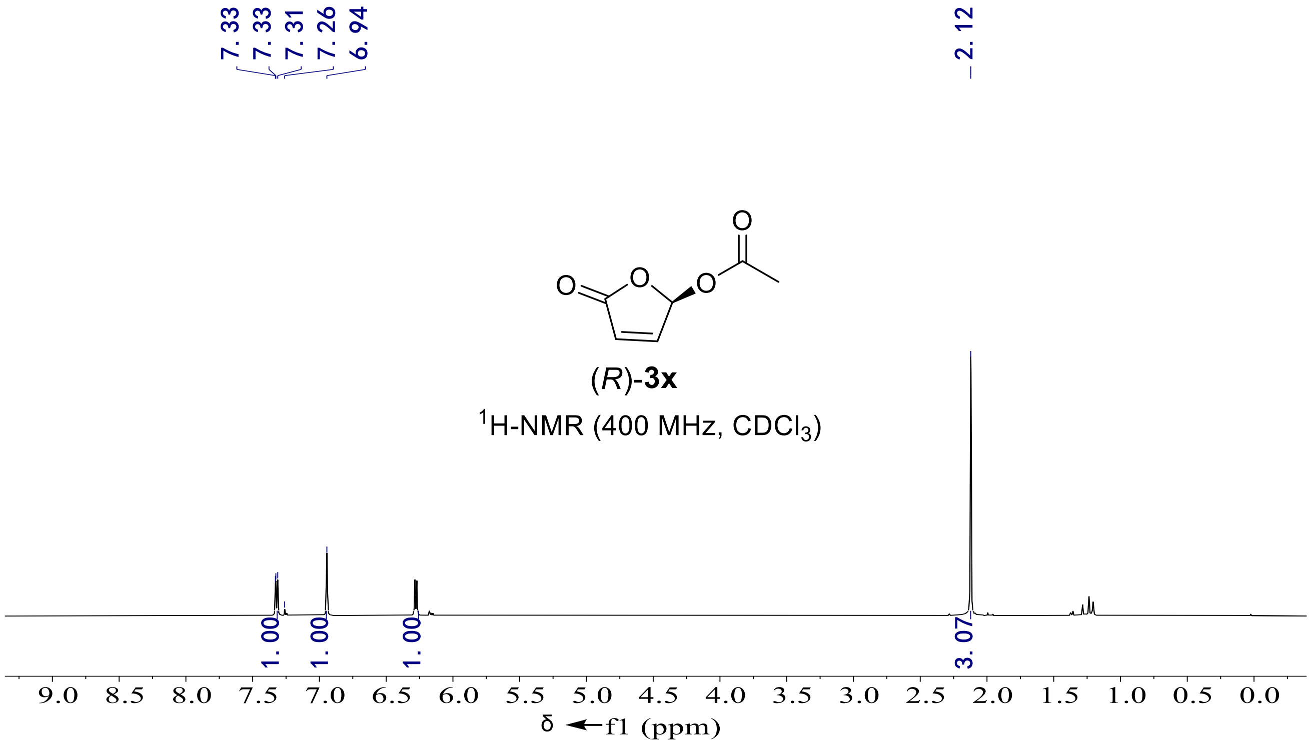

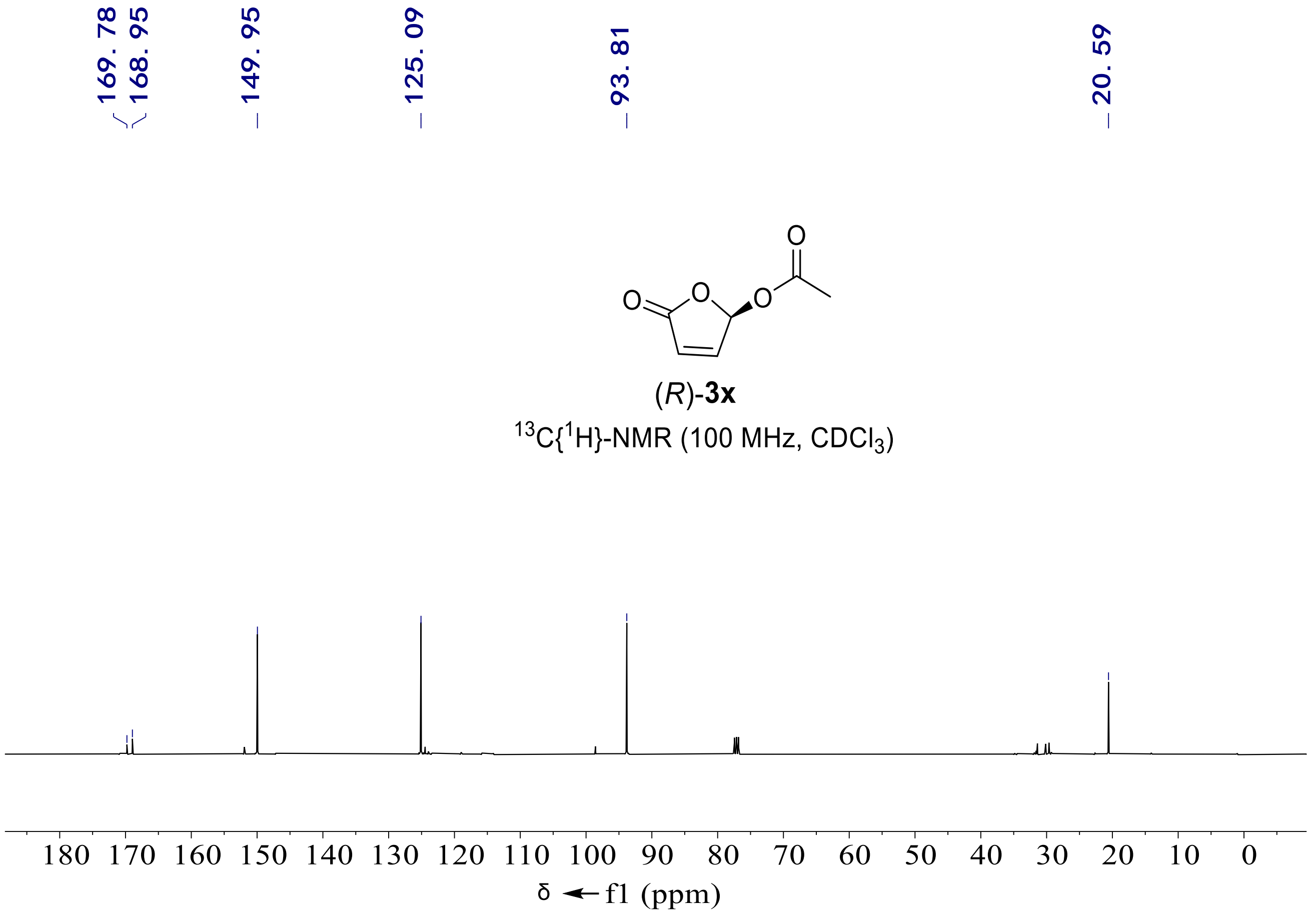

(*R*)-3y: (*R*)-chroman-2-yl acetate

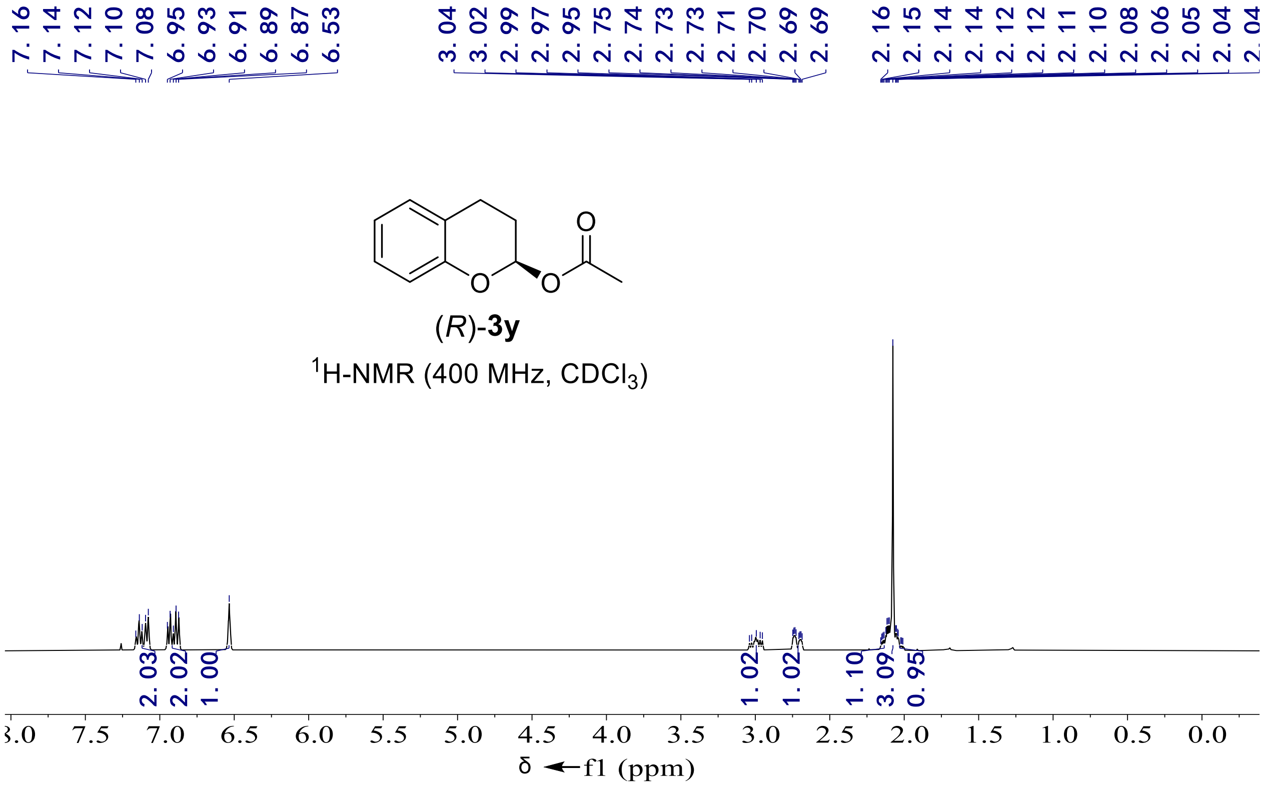

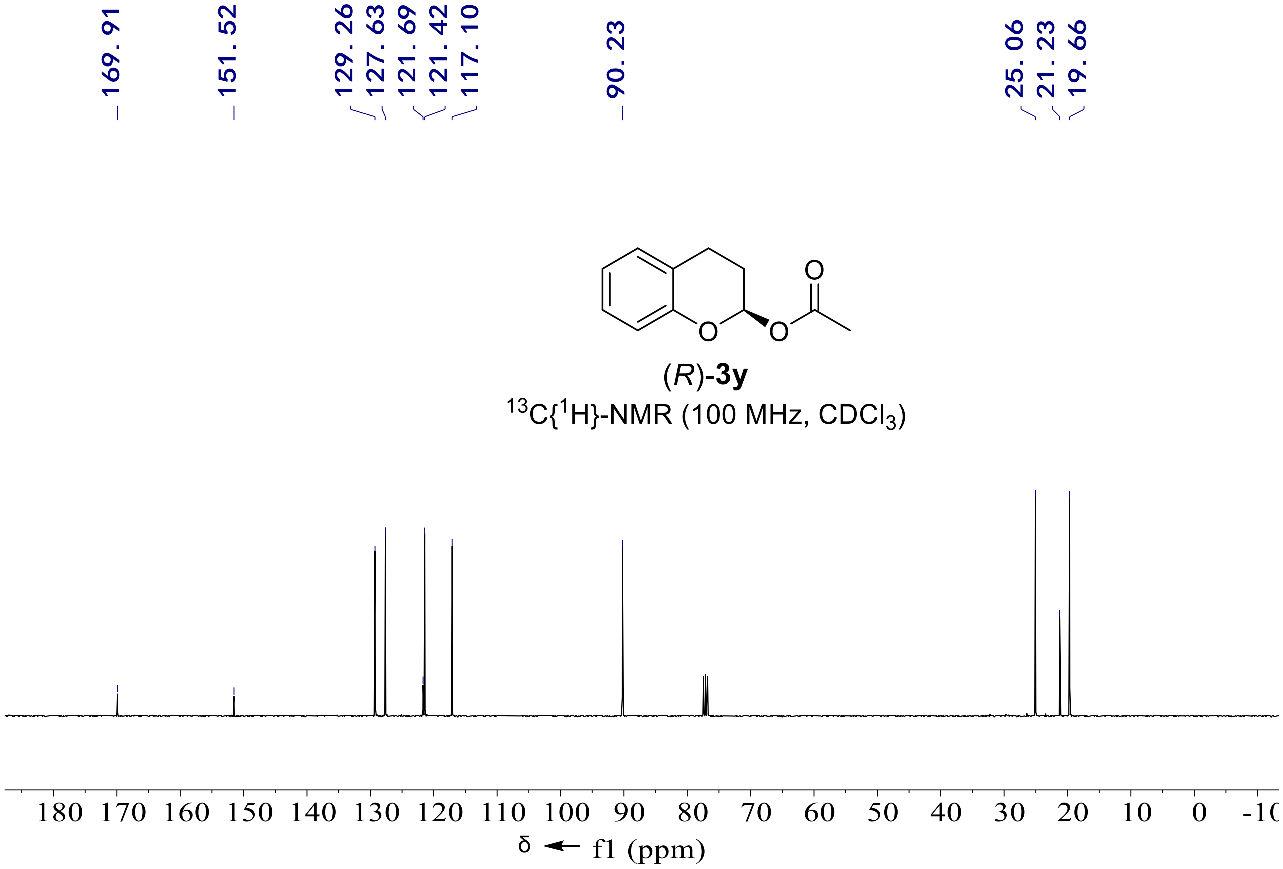

(*R*)-3z: **(*R*)-6-hydroxy-3-oxo-1,3-dihydroisobenzofuran-1-yl acetate.**

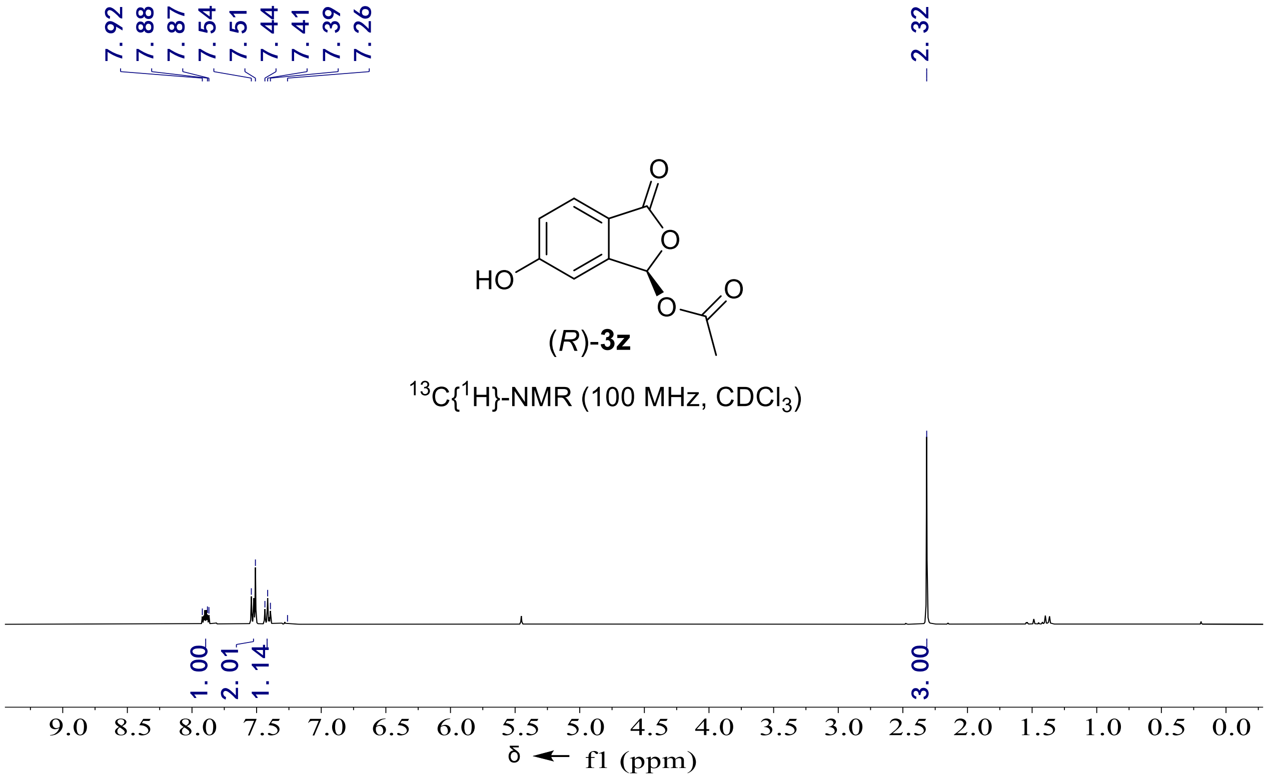

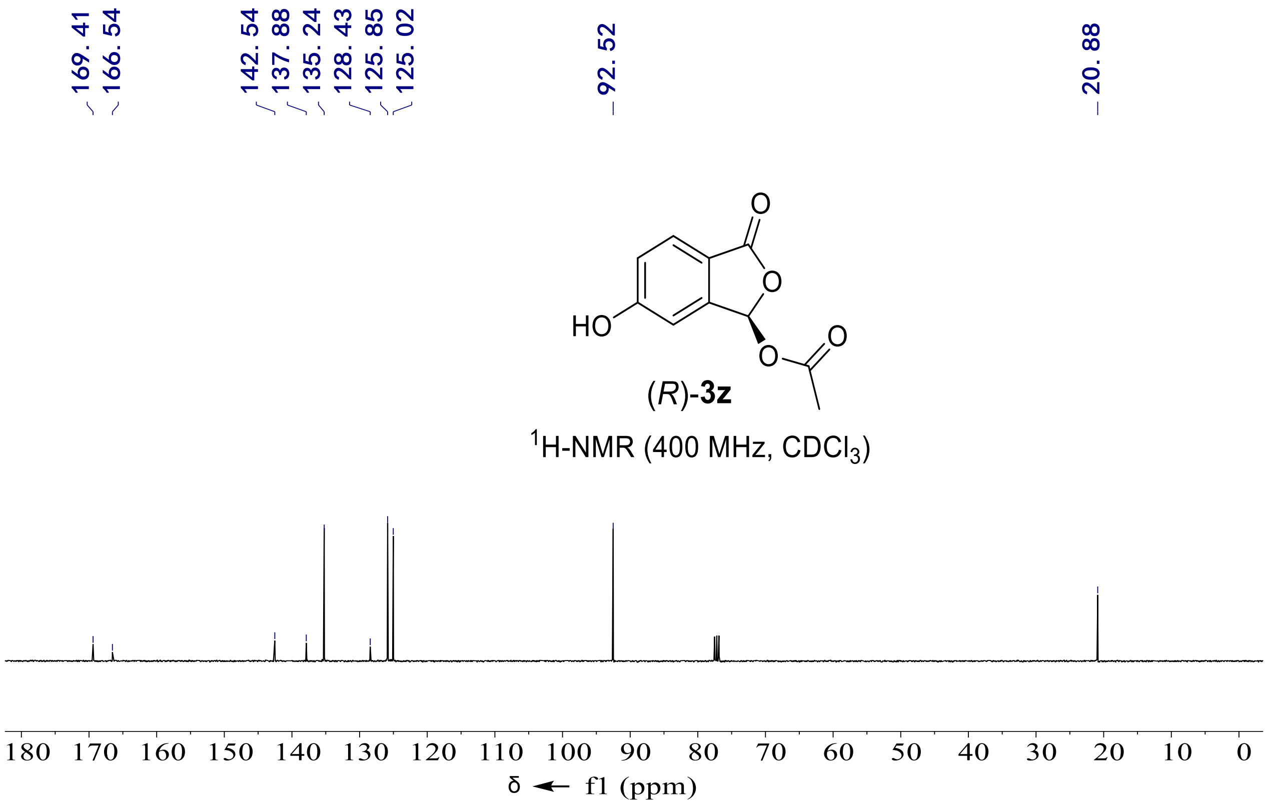

(*R*)-7: (*R*)-3-oxo-6-phenoxy-1,3-dihydroisobenzofuran-1-yl acetate

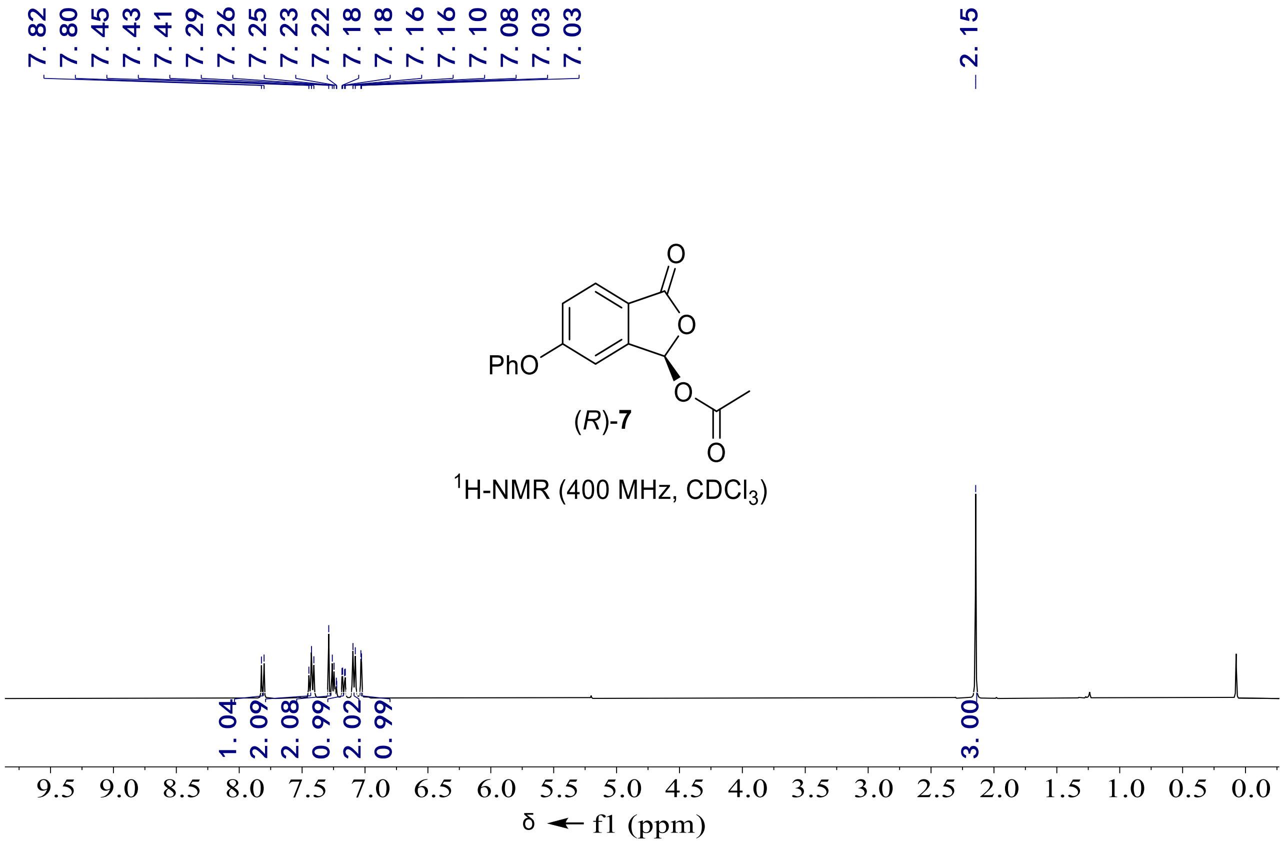

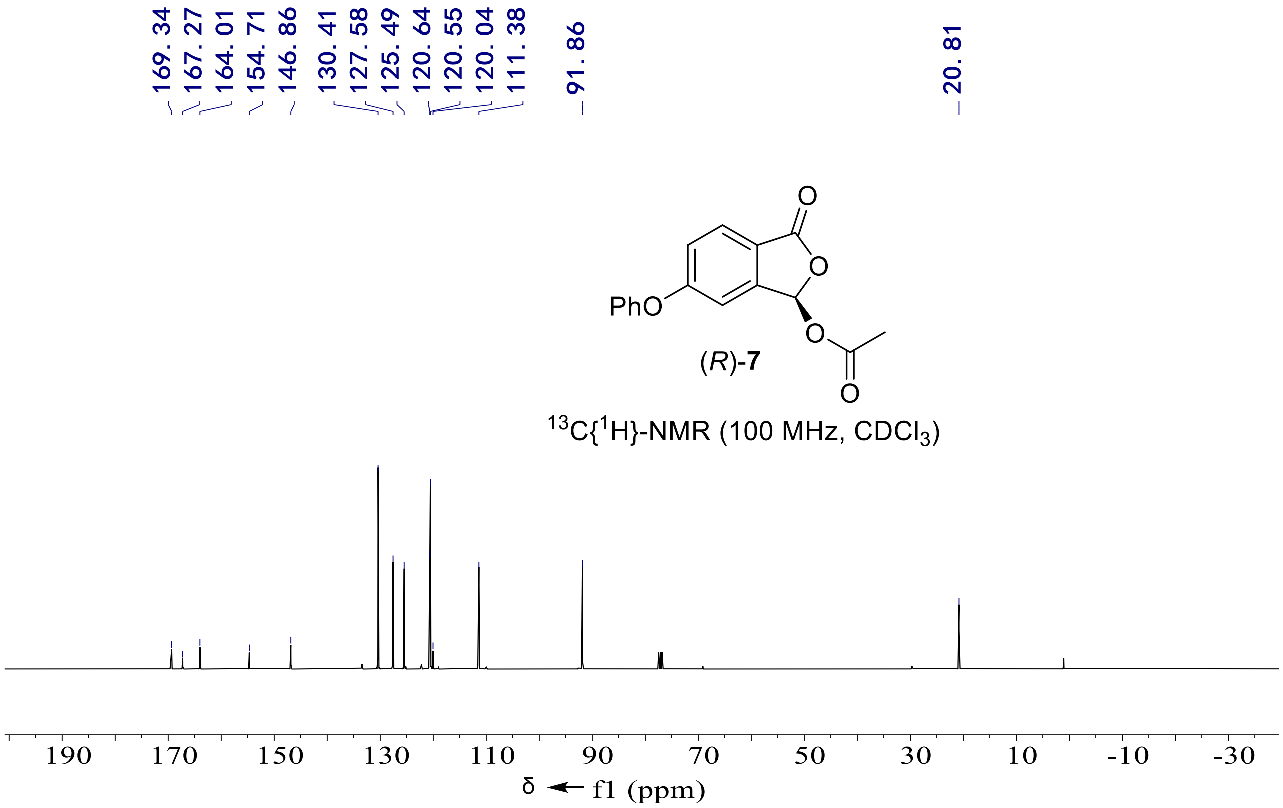

(*R*)-8: (*R*)-1-([1,1'-biphenyl]-4-yl)-3-oxo-1,3-dihydroisobenzofuran-1-yl acetate.

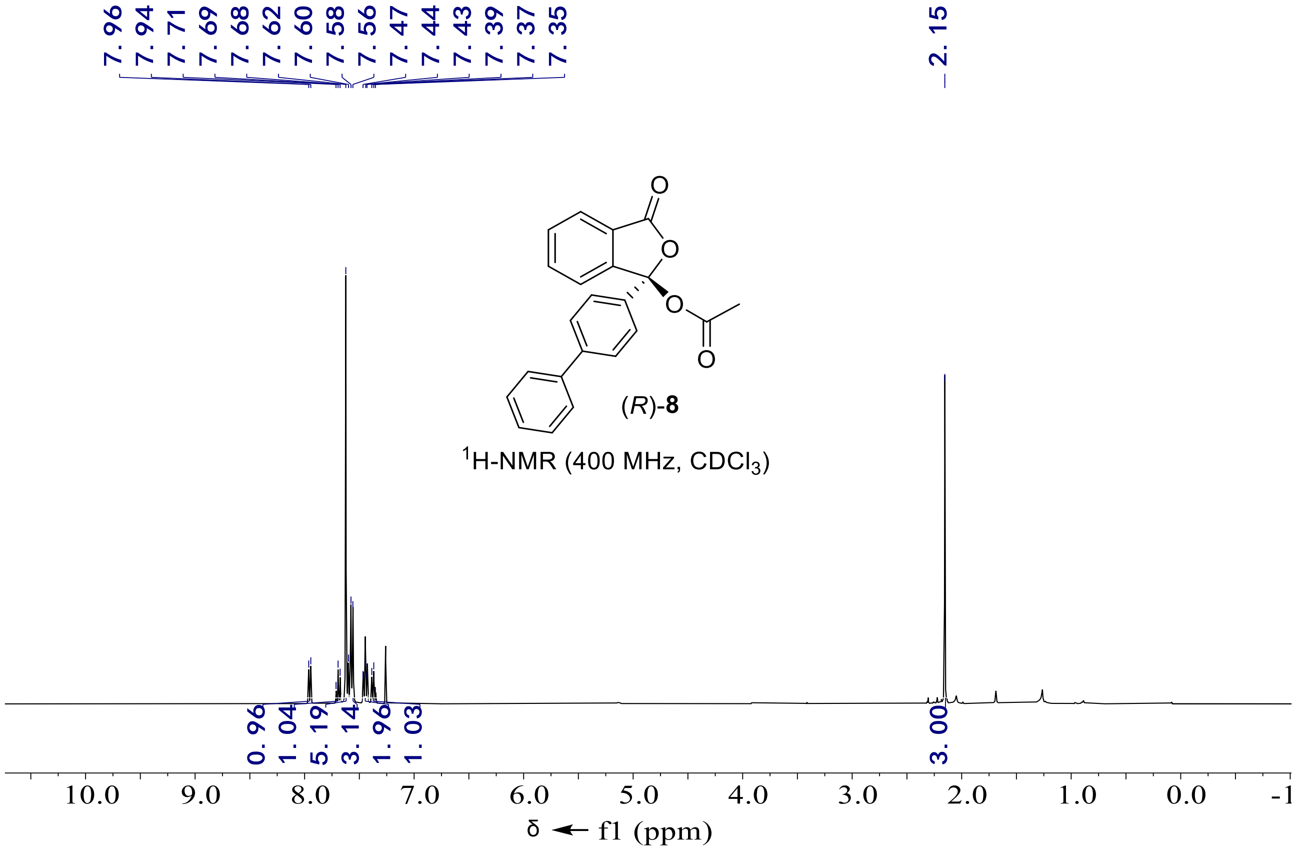

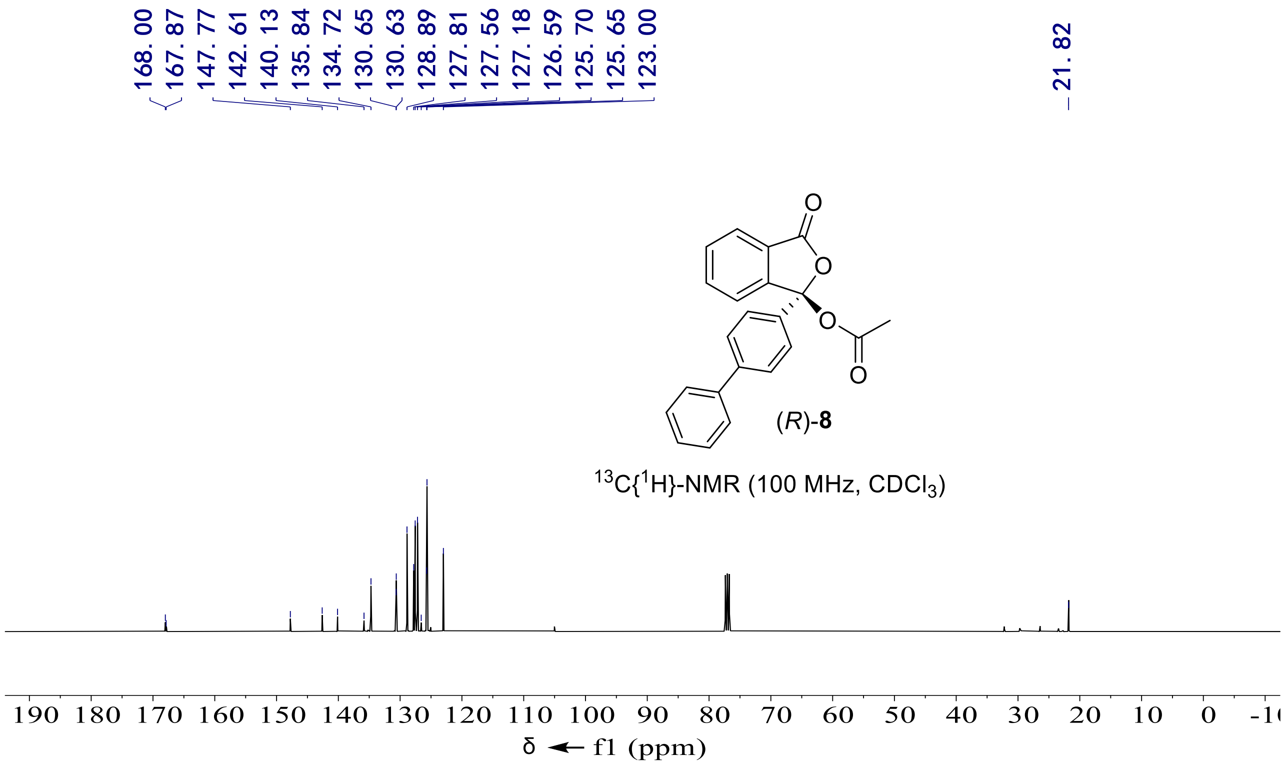

Supplementary Table 10. Reusability of the PDMS membrane (for the PDMS-modulated DKR process).

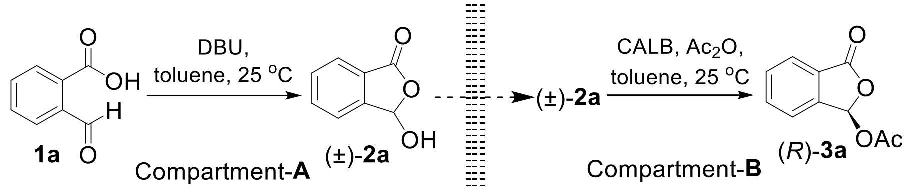

Entry	1	2	3	4	5	6
%Yield	90	88	86	83	81	68
%*ee*	99	99	98	98	97	95

Reaction conditions (The PDMS-modulated DKR process utilizes a 0.40 mm-thick PDMS membrane for its repeatability testing): In Compartment-**A**, **1a** (0.20 mmol), and DBU (0.20 mmol) in 2.0 mL of toluene, 25 °C, 36 h. In Compartment-**B**, Novozym-435 (CALB) (40.0 mg, 20 mg/0.1 mmol), and acetic anhydride (0.40 mmol, 2.0 equiv.) in 2.0 mL of toluene, 25 °C, 36 h. [b] Yields were determined by ^{1}H–NMR analysis in Compartment-**B**, and the %*ee* values were determined by chiral HPLC analysis.

Supplementary Figure 4. The SEM image of the PDMS membrane before and after the cycle.

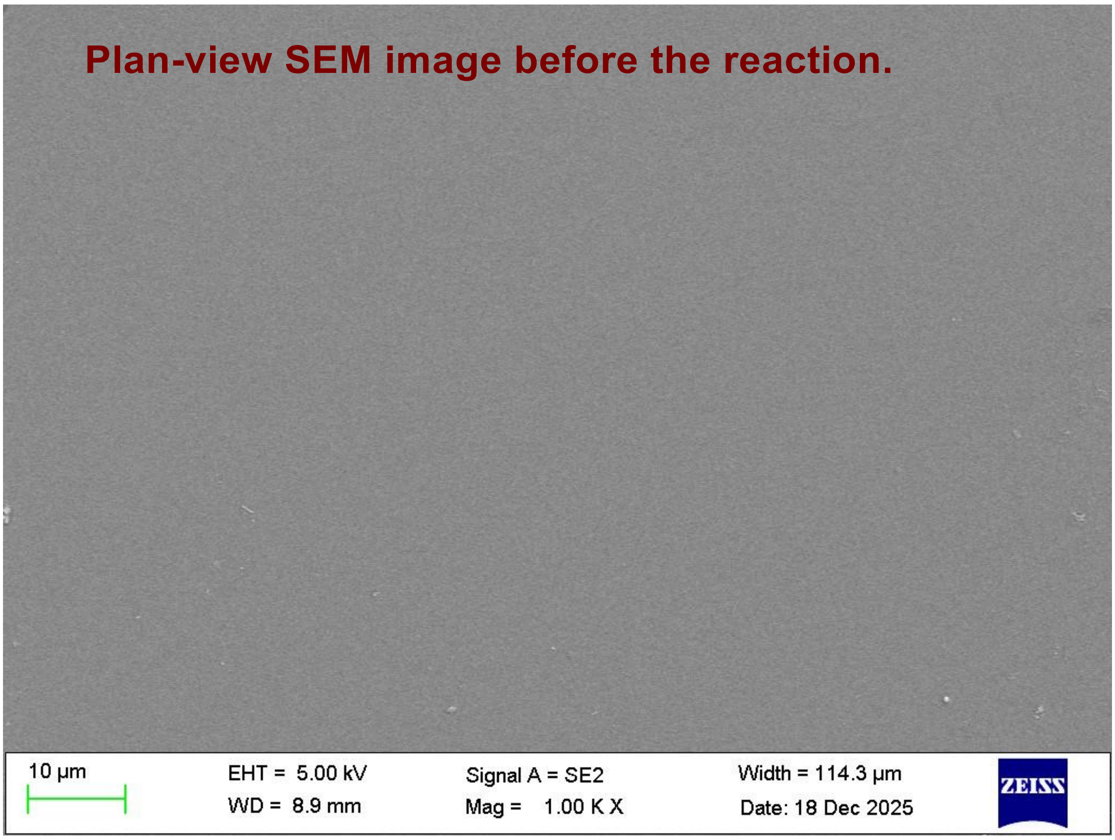

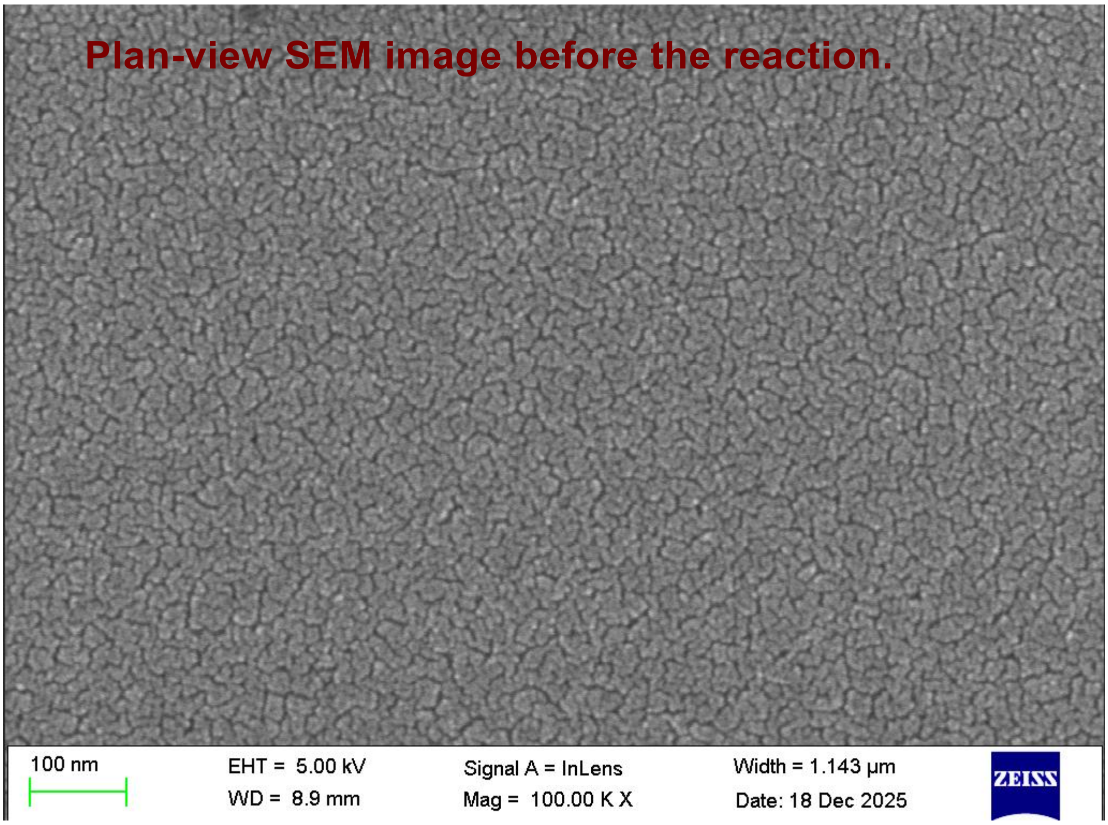

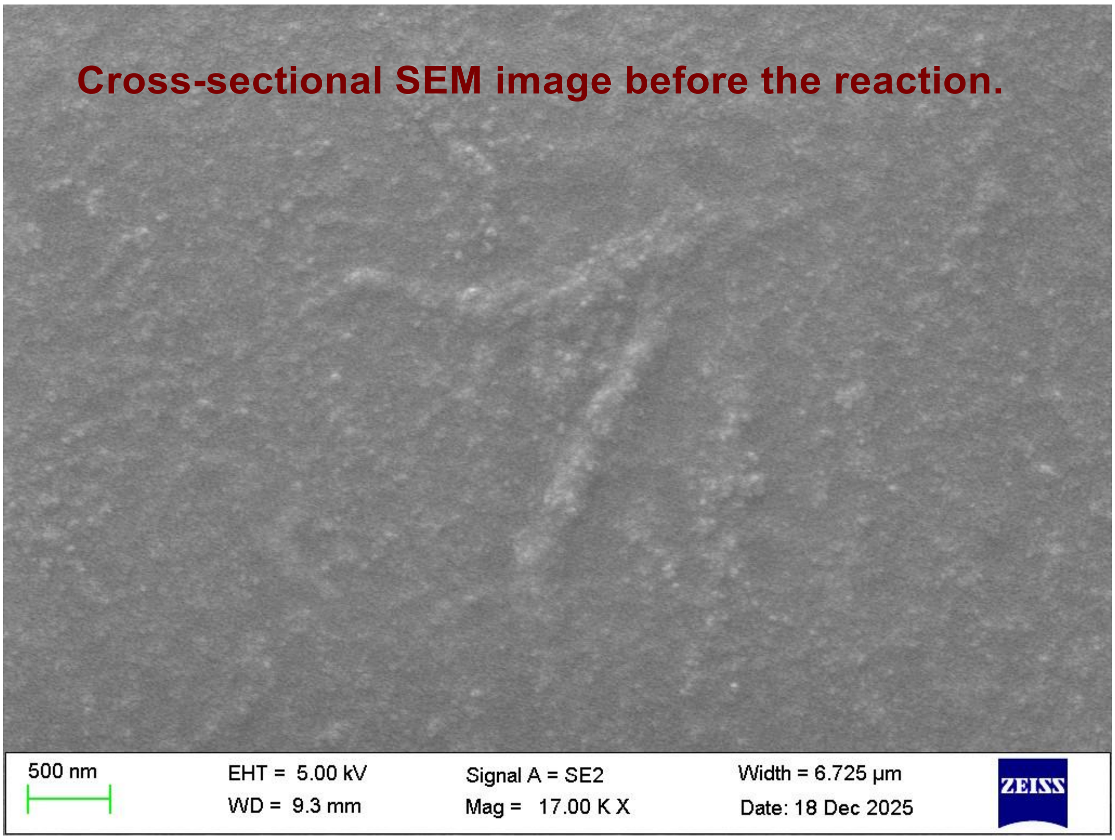
Cross-sectional SEM image before the reaction.
500 nm
EHT = 5.00 kV
WD = 9.3 mm
Signal A = SE2
Mag = 17.00 K X
Width = 6.725 µm
Date: 18 Dec 2025
ZEISS

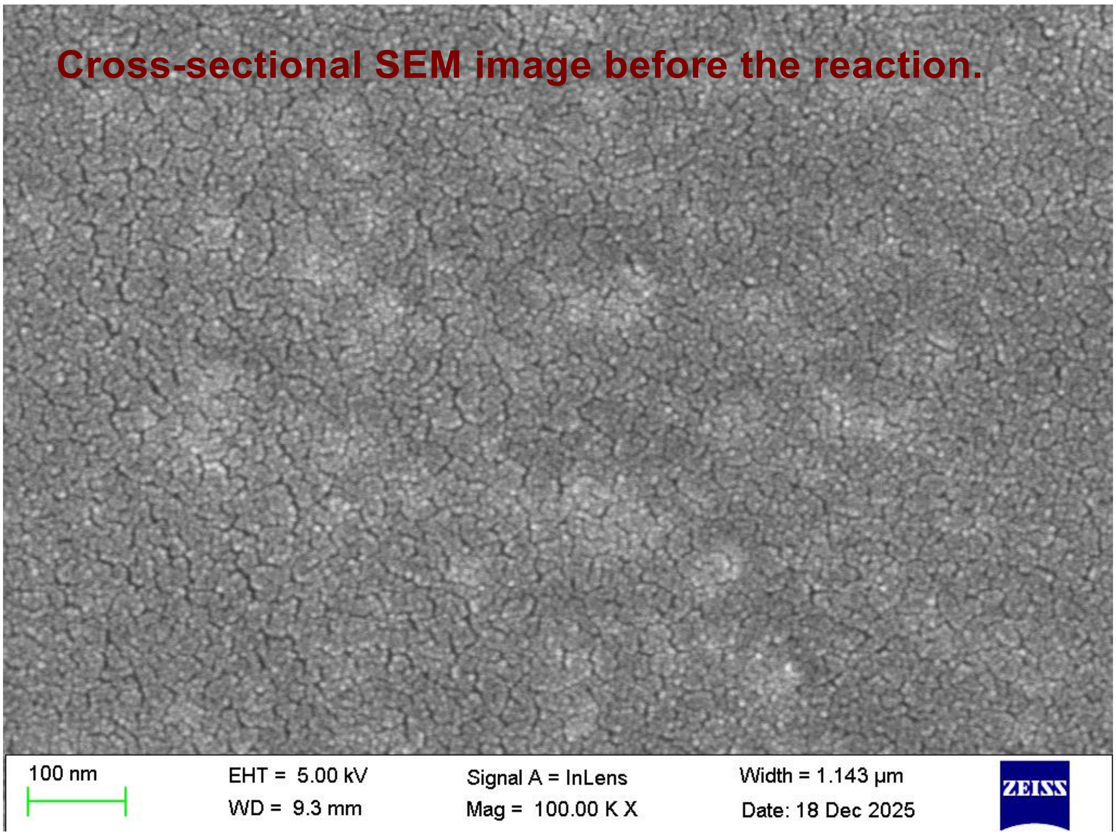
Cross-sectional SEM image before the reaction.
100 nm
EHT = 5.00 kV
WD = 9.3 mm
Signal A = InLens
Mag = 100.00 K X
Width = 1.143 µm
Date: 18 Dec 2025
ZEISS

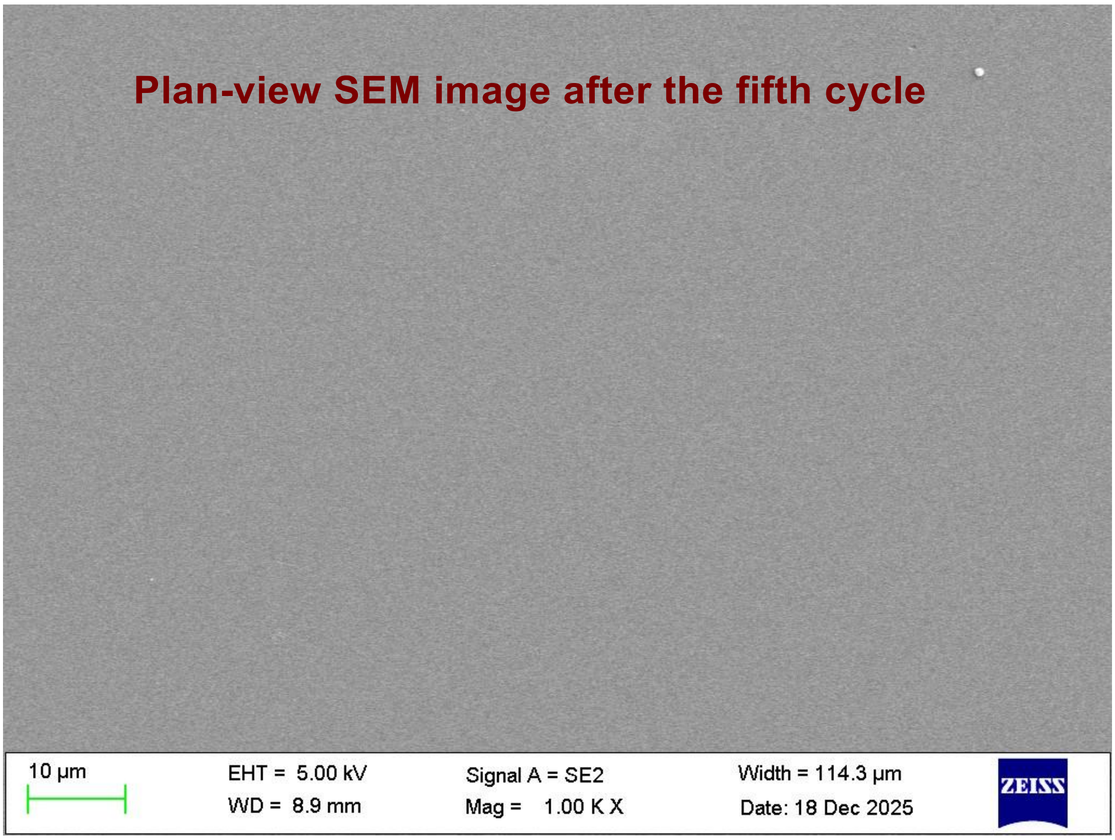
Plan-view SEM image after the fifth cycle
10 µm
EHT = 5.00 kV
WD = 8.9 mm
Signal A = SE2
Mag = 1.00 K X
Width = 114.3 µm
Date: 18 Dec 2025
ZEISS

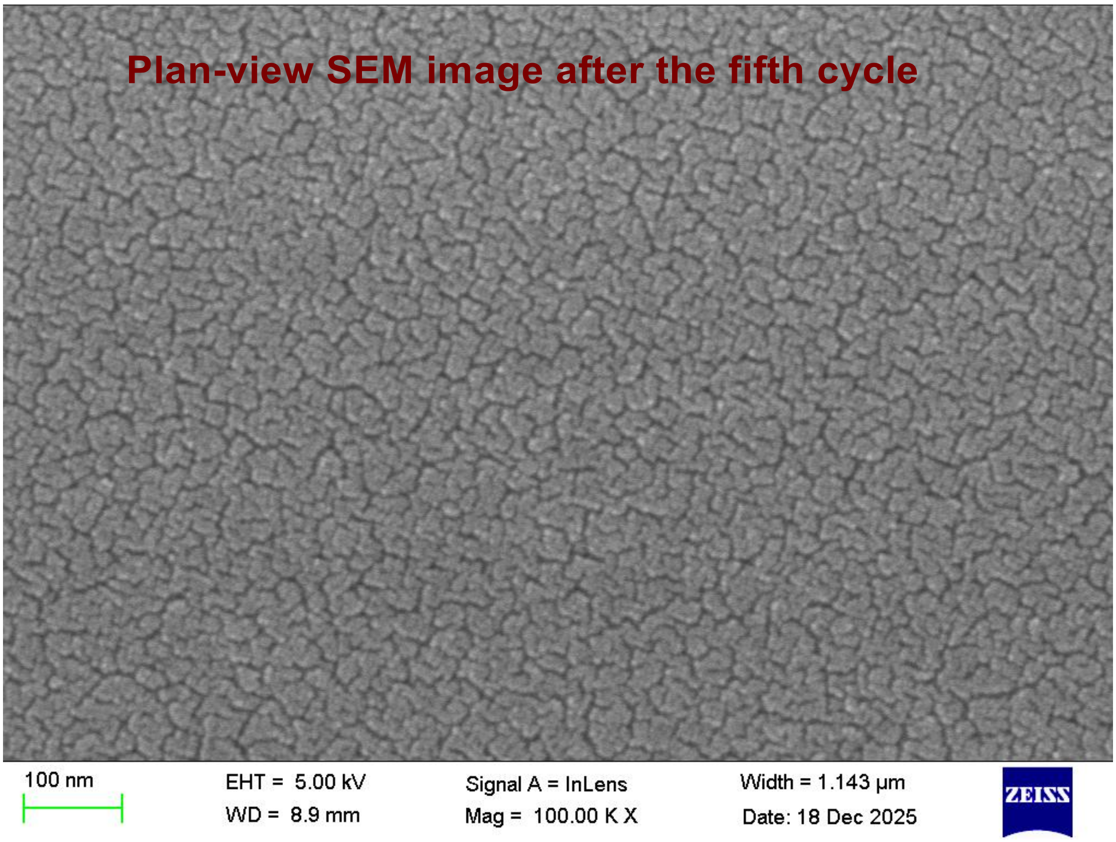
Plan-view SEM image after the fifth cycle
100 nm
EHT = 5.00 kV
WD = 8.9 mm
Signal A = InLens
Mag = 100.00 K X
Width = 1.143 µm
Date: 18 Dec 2025
ZEISS

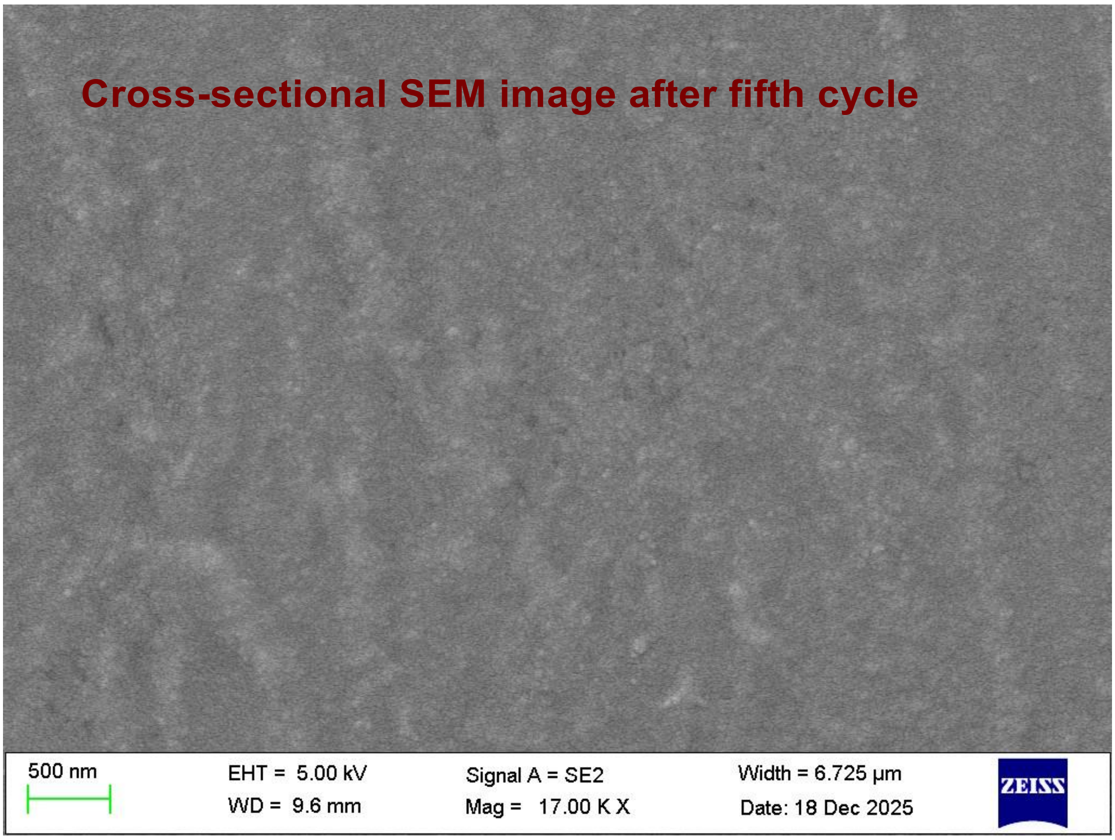
Cross-sectional SEM image after fifth cycle
500 nm
EHT = 5.00 kV
WD = 9.6 mm
Signal A = SE2
Mag = 17.00 K X
Width = 6.725 µm
Date: 18 Dec 2025
ZEISS

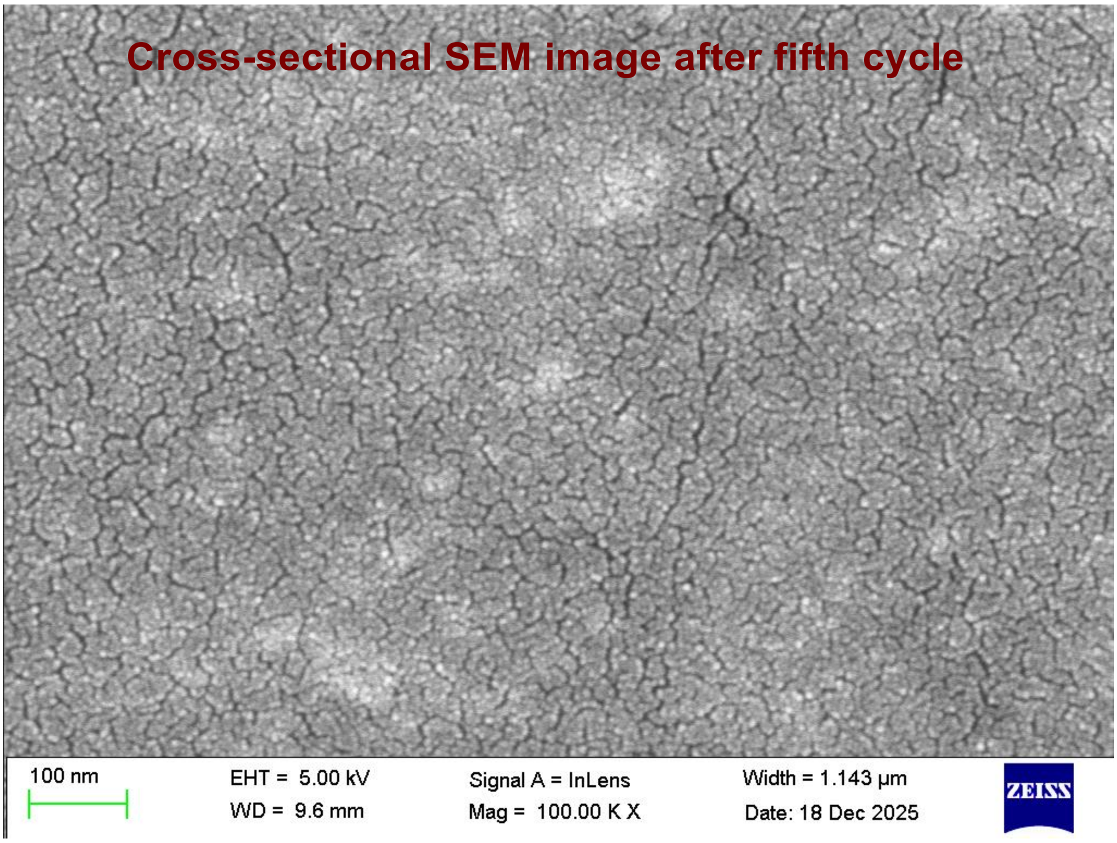
Cross-sectional SEM image after fifth cycle
100 nm
EHT = 5.00 kV
WD = 9.6 mm
Signal A = InLens
Mag = 100.00 K X
Width = 1.143 µm
Date: 18 Dec 2025
ZEISS

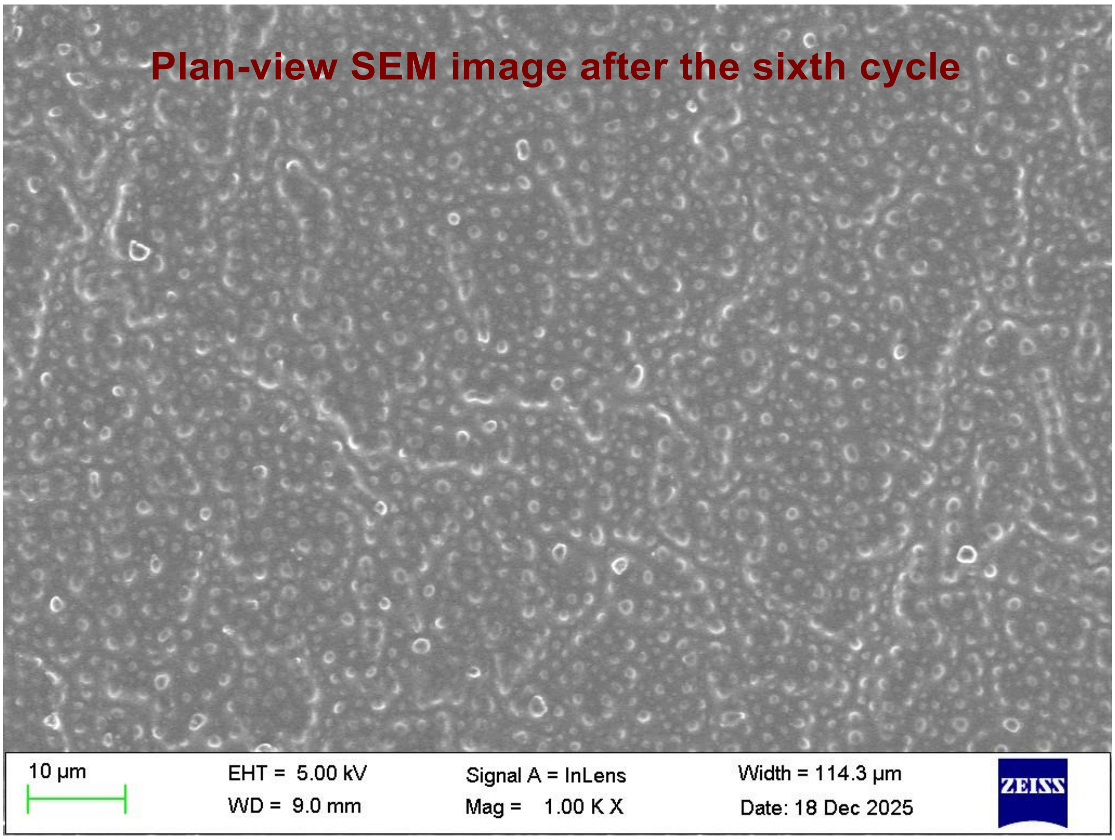
Plan-view SEM image after the sixth cycle
10 µm
EHT = 5.00 kV
WD = 9.0 mm
Signal A = InLens
Mag = 1.00 K X
Width = 114.3 µm
Date: 18 Dec 2025
ZEISS

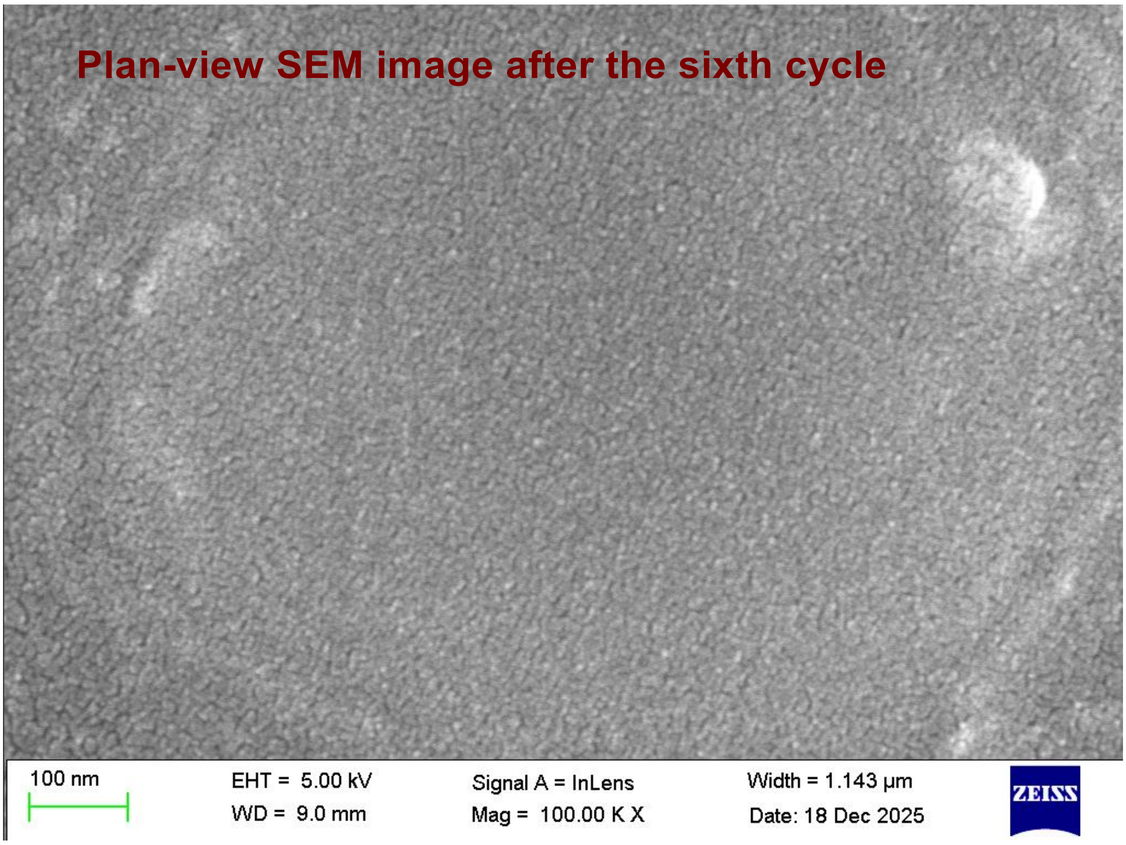
Plan-view SEM image after the sixth cycle
100 nm
EHT = 5.00 kV
WD = 9.0 mm
Signal A = InLens
Mag = 100.00 K X
Width = 1.143 µm
Date: 18 Dec 2025
ZEISS

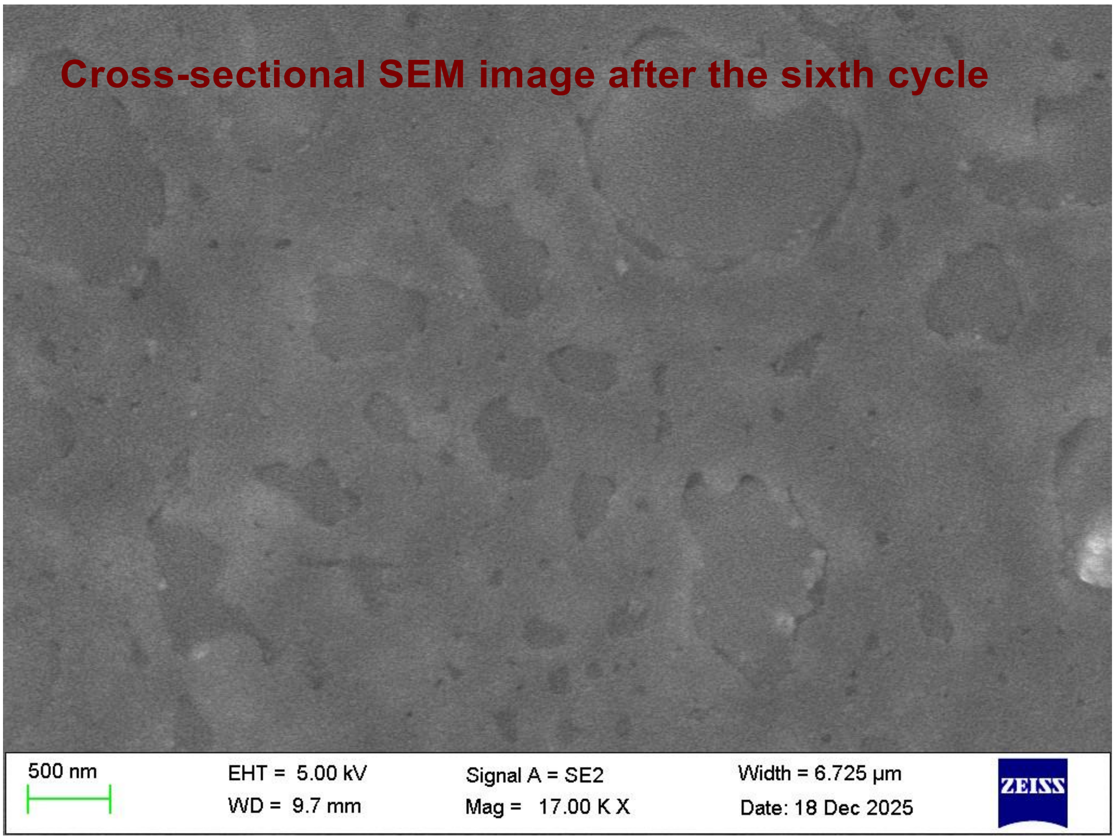
Cross-sectional SEM image after the sixth cycle
500 nm
EHT = 5.00 kV
WD = 9.7 mm
Signal A = SE2
Mag = 17.00 K X
Width = 6.725 µm
Date: 18 Dec 2025
ZEISS

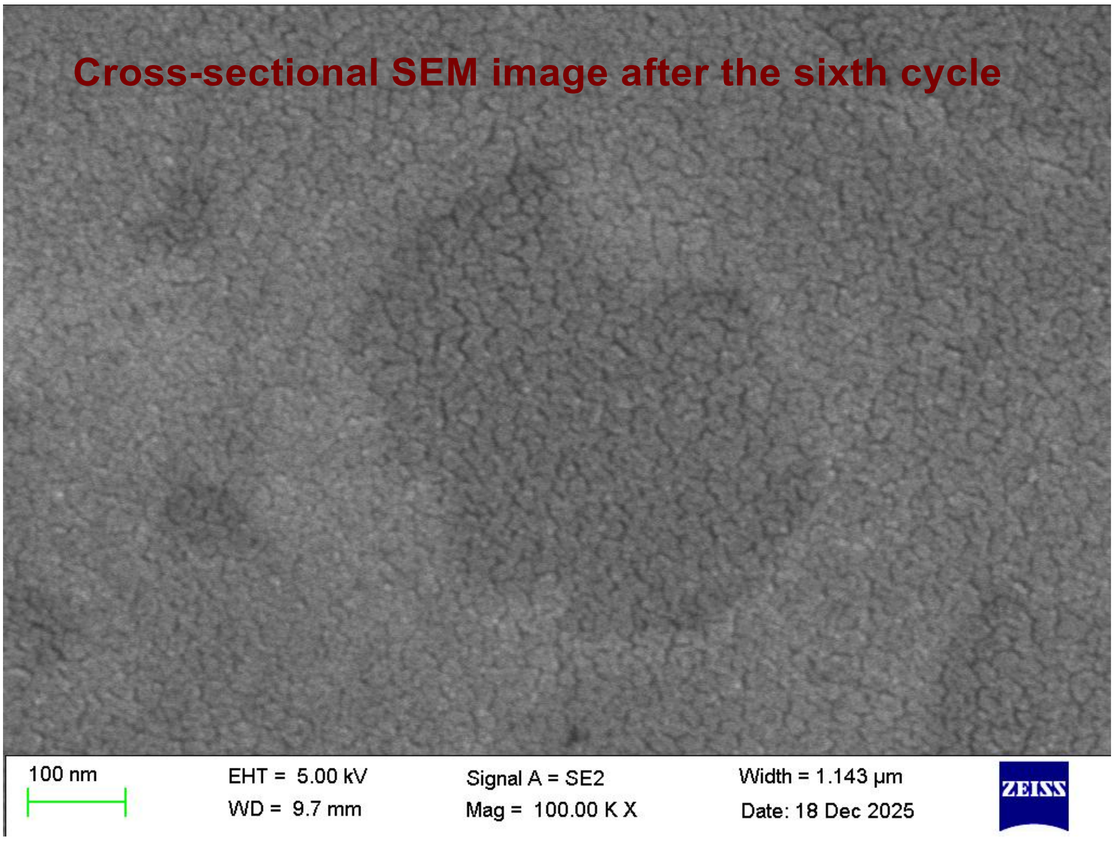
Cross-sectional SEM image after the sixth cycle
100 nm
EHT = 5.00 kV
WD = 9.7 mm
Signal A = SE2
Mag = 100.00 K X
Width = 1.143 µm
Date: 18 Dec 2025
ZEISS

Supplementary References:

1. Beck, D. E., Abdelmalak, M., Lv, W., Reddy, P. V. N., Tender, G. S., O'Neill, E., Agama, K., Marchand, C., Pommier, Y. & Cushman, M. Discovery of potent indenoisoquinoline topoisomerase I poisons lacking the 3-nitro toxicophore. *J. Med. Chem*. **58**, 3997–4015 (2015).
2. Shi, Y., Tan, X., Gao, S., Zhang, Y., Wang, J., Zhang, X. & Yin, Q. Direct Synthesis of Chiral NH Lactams via Ru-Catalyzed Asymmetric Reductive Amination/Cyclization Cascade of Keto Acids/Esters. *Org. Lett.* **22**, 2707-2713 (2020).
3. Agrawal, S. K., Majhi, P. K., Goodfellow, A. S., Tak, R. K., Cordes, D. B., McKay, A. P., Kasten, K., Bühl, M. & Smith, A. D. Synthesis of tetra-substituted 3-hydroxyphthalide esters by isothiourea-catalysed acylative dynamic kinetic resolution. *Angew. Chem. Int. Ed.* **63**, e202402909 (2024).
4. An, D., Yang, L., Liu, B., Wang, T. & Kan, C. Diffusion performance of fertilizer nutrient through polymer latex Film, *J. Agric. Food Chem.* **65**, 10868-10874 (2017).
5. Yang, L., An, D., Wang, T., Kan, C. & Jin, Y. Swelling and diffusion model of a hydrophilic film coating on controlled-release urea particles, *Particuology* **30**, 73–82 (2017).
6. McClelland, R. A. & Sørensen, P. E. Kinetics of the equilibration of 3-hydroxyphthalide and o-formylbenzoic acid. Hemiacetal breakdown with a carboxylic acid leaving group, *Can. J. Chem*. **64**, 1196-1200 (1986).
7. Cabordery, A., Toussaint, M., Azaroual, N., Bonte, J., Melnyk, P., Vaccher, C. & Foulon, C. Kinetics and mechanism of racemization of Tic-hydantoins, potent sigma-1 agonists, *Tetrahedron: Asymmetry* **22**, 125–133 (2011).
8. Danel, C., Azaroual, N., Brunel, A., Lannoy, D., Odou, P., Décaudin, B., Vermeersch, G., Bonte, J. & Vaccher, C. Configurational stability of 9-hydroxyrisperidone. Kinetics and mechanism of racemization, *Tetrahedron: Asymmetry* **20**, 1125–1131 (2009).
