# Supplementary material for: A membrane-modulated chemoenzymatic dynamic kinetic resolution for the synthesis of chiral phthalidyl esters
Source: Nat Commun. 2026 May 2;17:5950. doi: 10.1038/s41467-026-72684-2 (PMC13342490; doi:10.1038/s41467-026-72684-2)
Supplement: Supplementary file 2 — Transparent Peer Review file [file 41467_2026_72684_MOESM2_ESM.pdf]

nature portfolio

Peer Review File

A Membrane-Modulated Chemoenzymatic Dynamic Kinetic Resolution for the Synthesis of Chiral Phthalidyl Esters

Corresponding Author: Professor Guohua Liu

This file contains all reviewer reports in order by version, followed by all author rebuttals in order by version.

Version 0:

Reviewer comments:

Reviewer #1

(Remarks to the Author)
Chemoenzymatic dynamic kinetic resolution (DKR) is a powerful tool to combine chemocatalysis and biocatalysis for the construction of chiral skeletons from the racemic mixtures, however, compatibility of dual systems is the general issue, which has to be overcome. Herein, the authors developed a membrane-modulated strategy, which presents an alternative design to achieve chemoenzymatic DKR transformations by spatially separating racemization and resolution. Finally, the collaborative operation within a single step enables the DKR approach to allow the efficient synthesis of 3-hydroxyphthalide esters. The design is smart, and substrate scope is broad. Based on it, this reviewer supports the acceptance of the manuscript as publication in the high-impact journal of Nat. Commun. after minor revisions, by addressing the following concerns.
1. In Scheme 1A, the authors showed a significant decrease in the enantiomeric excess of the product when DBU is added stepwise in a one-pot reaction. This tendency could be plausibly attributed to DBU directly participating in a non-enzymatic background esterification. The authors hypothesize that the membrane prevents DBU, the substrate, and the acetate ester from coexisting in the same compartment, thereby suppressing this background reaction. However, in my opinion, this postulate requires further validation. Based on the provided description, while the substrate can permeate the membrane to access the DBU compartment, it remains unclear whether the acyl donor can also diffuse through the membrane. If the acyl donor is permeable, the background reaction could still occur. The authors should clarify how the membrane compartmentalization successfully enables the DKR transformation under these conditions.
2. This reviewer is concerned about the practical application of the products. It appears that after the chiral ester is de-esterified to the alcohol, the resulting compound is prone to racemization under basic conditions, leading to a loss of optical activity. The authors should consider this possibility, and provide evidence to demonstrate the potential for application of the chiral products.
3. Some related papers (J. Am. Chem. Soc. 2015, 490 137, 3996–4009; ACS Cent. Sci. 2024, 10, 2099–2110; Chin. Chem. Lett. 2024, 35, 109160; Chin. Chem. Lett. 2025, 36, doi.org/10.1016/j.cclet.2025.111943.) could be cited.

Reviewer #2

(Remarks to the Author)
This review was conducted with an Early Career Researcher participating in the co-review initiative of Nat. Commun. I, as the senior scientist, am responsible for the final version of this report, which is the result of a single evaluation by me and my coworker, our follow-up discussion, and the final writing of this report. According to the instructions, we do not give a specific recommendation to the editor for acceptance or rejection; rather, we critically analyze this work in terms of novelty, potential impact, and overall quality of the research, highlighting strengths and limitations.
As a general comment, I believe that the authors have made heavy use of an AI language model to write this manuscript. This is evident from the narrative, which is lengthy, repetitive, pompous, and also tries to “oversell” the novelty and impact of the work in some sections. I strongly recommend revising the entire manuscript using a more balanced and sober narrative that is more in line with academic writing.
In general, this is an excellent work in terms of development of the system, catalytic efficiency, and substrate scope covered. However, I have doubts regarding the real novelty and potential impact in the field in relation to the state of the art for the reasons explained below.

Comments

1. The introduction is very lengthy, repetitive, and difficult to follow. There is a lot of “unfocused” narrative. The key points are diluted in the lengthy text, making comprehension and grasping the main points very difficult. I suggest shortening the introduction and concisely discussing the state of the art (i.e., what the current limitations are and how this work can help to solve such limitations). There is no need to use six A4 pages to discuss all this. Half the length of the text would be more than enough. There is also a lot of emphasis on the discussion of compartmentalized dynamic kinetic resolution. Many statements are too “grand,” while the authors are essentially presenting a work based, in many parts, on already well-known technology. Lipases have been among the first enzymes used in biocatalysis; resolution of enantiomers with enzymes is a classic process, and DKR is almost 30 years old if we consider the work by Bäckvall or the first report by Williams and coworkers. The first chemoenzymatic DKR was accomplished with 1-phenylethanol using a lipase for the acyl-transfer reaction and a rhodium complex for racemization in 1996: (P. M. Dinh, J. A. Howarth, A. R. Hudnott, J. M. J. Williams, W. Harris, Tetrahedron Lett. 1996, 37, 7623–762). The use of membrane reactors in batch is also known.
Notably, similar types of DKR were conducted with DBU as a racemization catalyst in flow reactors with excellent efficiency. An example is P. Falus et al., Adv. Synth. Catal. 2016, 358, 1608–1617. Using two reactors in flow is another way to achieve compartmentalization, which is what the authors did in their paper using a membrane in a batch system. Therefore, conceptually, there is not so much novelty in compartmentalization of chemo- and biocatalysts. The authors should also mention flow-chemistry applications of DKR.
There is also another limitation. While a flow-reactor methodology can in principle deliver 99% conversion of the product in optically pure form, the batch solution with a membrane slows down significantly around 90% conversion due to diffusional limitations (i.e., the kinetics of diffusion at low concentration of remaining substrate becomes severely rate-limiting). The authors must mention this limitation. In a real-world application, incomplete conversion requires further isolation and purification steps, making the process less appealing.

2. The text is also redundant in the Results and Discussion. There are three main key points: 1) DBU influences the catalytic activity and, maybe, the enantioselectivity of CALB; 2) DBU can act as a catalyst for the esterification itself with no selectivity; 3) points 1 and 2 impede the classical DKR, and the only way to do it is through compartmentalization of the two catalysts.
These are three simple concepts to convey, yet the authors use very long text, repeating the same things several times. The text is redundant and rather pompous. A more concise and scientifically sober narrative would be advisable.

3. Page 2: the authors state: “However, the fundamental challenge of a conventional DKR process lies in synchronizing the two catalytic events of racemization and resolution in accordance with the core principles: (i) the racemization rate must exceed the resolution rate to ensure the selective and rapid transformation of the favored enantiomer (first limitation).”
This sentence is not conceptually correct in relation to DKR, in general. The racemization rate must be significantly higher than the rate for the conversion of the “wrong” (undesired) enantiomer by the enzyme. However, the racemization rate can be (and normally is) lower than the rate for the conversion of the desired enantiomer.
Citing from Verho and Bäckvall, J. Am. Chem. Soc. 2015, 137, 3996–4009:
“However, the design of a successful DKR system is far from simple, given that the following requirements must be fulfilled: (i) the KR must display a sufficient enantioselectivity (E value = kfast/kslow ≥ 20); (ii) the enzyme and the racemization catalyst must be compatible with one another; (iii) the rate of racemization (krac) must be at least 10 times faster than the enzyme-catalyzed reaction of the slow-reacting enantiomer (kslow); and (iv) the racemization catalyst must not react with the product formed from the resolution.”
In conclusion, there is no mention of kfast, strictly speaking.
I think what the authors mean is that, in their specific case, it is necessary that the racemization is faster than kfast because the racemization catalyst (DBU) can also catalyze the esterification but in a non-enantioselective manner, thus eroding the ee. In conclusion, the statement in the manuscript should be modified accordingly.

4. - Page 4: “In such systems, the substrate forms an unstable hemiacetal intermediate with the NHC catalyst, and the resulting energy difference between matched and mismatched isomers promotes efficient racemization of the unfavored enantiomer, ultimately delivering high DKR efficiency”.
NHC has never been introduced so far as acronym.

5. Page 6: “Beyond shielding, the selective permeability of the PDMS membrane leverages its dynamic mass-transfer capability, permitting the diffusion of targeted intermediate (3-hydroxyisobenzofuranone), while confining catalysts, substrates, and other components to their respective compartments”.
It would be beneficial for the paper and the readers to add to Figure 1 letters for each molecule and then add it in the text, 3-hydroxyisobenzofuranone (xxx).

6. Page 7: “As presented in Figure 1C, by utilizing the dynamic tautomeric equilibrium between 1a and 2a, a practical PDMS-modulated chemoenzymatic DKR process as a representative ...”
In Figure 1, the authors forgot to insert the numbers in the molecules for 1a and 2a, present for example in Table 1. Please add it to the table to render the comprehension easier.

7. Page 9: “Notably, these comparisons revealed that the second-step biocatalysis was severely suppressed in the presence of ...”
I guess the authors wanted to write the second biocatalytic step instead of second-step biocatalysis. Please rewrite to make it clear.

8 Page 9: “Finally, in the evaluation of an integrated PDMS-modulated DKR (II) based on the optimizations (see Supplementary Experimental and Table S2, SI), it was observed that the model reaction could steadily provide the final

(R)-3a with a 91% yield and 99% ee (entry 8), which was slightly superior to the outcomes obtained with other DBU loadings (entry 8 versus 10 entries 9 and 10)".
I don't think the results obtained in entries 8, 9 and 10 are statistically different. How would you explain this different outcome? From my perspective, it is just a normal experimental fluctuation of data. Statistical analysis from more data of same type of experiments would be needed to prove that.

9. - Page 10: "In addition, it was also found that the membrane thickness had a certain impact on the PDMS-modulated DKR process, in which the reaction using a 0.40 mm-thick membrane yielded the best results compared to the reactions with other membrane thicknesses (entry 8 versus entries 11 and 12)".
How would the authors explain this phenomenon?

10. Page 12: again the same mistake in my opinion: "To elucidate the unique advantage of independent compartmentalization in circumventing the conventional requirement that the racemization rate must exceed the resolution rate (i.e., krac > kfast to ensure continuous racemization of the unreacted enantiomer)."
This is not correct: krac >> kslow, but krac can also be equal to or smaller than kfast in a conventional DKR. It is the specific case of this DKR using DBU as the racemization catalyst that creates this additional requirement. It should be explained clearly.

11. Page 13: "Thus, the combination of this membrane-mediated device with the dynamic tautomerization facilitated by even any racemization base catalyst in Compartment-A was sufficient to sustain an efficient DKR process, thereby establishing a new mechanistic paradigm in which a concentration gradient, rather than relative rate control, drives the process."
I do not think that this is a new mechanistic paradigm. It is based on the law of diffusion, and racemization is based on the concept of entropy—very classical concepts from the last century and earlier that can be found in textbooks. Again, an exaggerated narrative, in my opinion.

12.- Page 15: "This equilibrium facilitates the transmembrane migration of racemic 2 while retaining 1 in Compartment-A".
Why is 1 retained? Do you have proof of the fact that 1 doesn't pass through the membrane? Did you try to put 1 in compartment B?

13. Page 19: "Third, for the racemic tetra-substituted 3-hydroxyphthalides (5a–5h), which was difficult to access by conventional chemoenzymatic strategy, the membrane-modulated DKR also exhibited remarkable tolerance, consistently furnishing the challenging chiral tetra-substituted 3-hydroxyphthalide esters ((R)-6a–(R)-6h) in acceptable yields with excellent enantioselectivities (Equation 3, Scheme 3)."
This limitation is attributable to CALB. Why does the DKR of tetra-substituted 3-hydroxyphthalides become easier with the membrane reactor if the catalytic rate depends only on the enzyme? Can the authors comment on that and provide an explanation?

14. Page 21, but also in other sections of the manuscript: "The independent compartmentalization maintains kinetically coupled yet isolated environments, sustaining a substrate concentration gradient that drives DKR forward without kinetic synchronization."
The use of the definition "kinetic synchronization" is not standard in DKR. The authors should better define what they mean by "kinetic synchronization." It would appear that "kinetic synchronization" is not possible in DKR with catalysts compartmentalization, while in contrast kinetic resolution and racemization must always run concurrently. In conclusion, either the authors can explain in a concrete way what this is about or such sentences must be removed from the manuscript.

15. Page S4: Why do you need a balloon filled with air? What happen if you close the system completely? Why did you filter the solution afterward? What is the solid part coming from? Only the enzyme?

16. Page S27: the two HPLC chromatograms don't testify the production of one of the enantiomer; the retention times are different between the racemic mixture and the enantioselective one (with only one peak).

17. Page S32: the two HPLC chromatograms don't testify the production of one of the enantiomer; the retention times are different between the racemic mixture and the enantioselective one (with only one peak).

18. Page S33: Why are there two different retention time for the same molecule? Please explain more carefully.

19. Page S40: In all the previous cases the R enantiomer is the first one; why is it the second peak on this specific example? Are you sure this is not the S enantiomer?

20. Page S68, S69: In all the previous cases the R enantiomer is the first one; why is it the second peak on this specific example? Are you sure this is not the S enantiomer?

21. Page S71: the two HPLC chromatogram don't testify the production of one of the enantiomer; the retention times are different between the racemic mixture and the reaction sample (with only one peak).

22. Page S80: the two HPLC chromatogram don't testify the production of one of the enantiomer; the retention times are different between the racemic mixture and the reaction sample (with only one peak).

Reviewer #3

(Remarks to the Author)
I co-reviewed this manuscript with one of the reviewers who provided the listed reports. This is part of the Nature Communications initiative to facilitate training in peer review and to provide appropriate recognition for Early Career Researchers who co-review manuscripts.

Reviewer #4

(Remarks to the Author)
Authors have presented a methodology for developing novel chemoenzymatic DKR procedures using DBU as racemization catalysts and CAL-B as selective acylation reagent, employing a PDMS membrane for the separation of both catalysts in order to avoid all the issues derived from the direct combination of these two catalysts. A wide set of 3-hydroxyphthalides are selectively resolved with good yields and excellent enantiomeric excesses, but the manuscritp has to be improved in certain aspects:
- Throughout the manuscript, the PDMS-mediated decoupling of chemo- and biocatalysis is presented as a conceptual advance that, as authors stated “bypasses fundamental DKR limitations” (pages 4–7). But the
- PDMS-thimble compartmentalization has been extensively used to resolve catalyst incompatibility, as shown in references from 29 to 38. Thus, aapart to increase the applicability of this type of systems by using a different reaction class, it is not bery clear what is conceptually new. A reformulation of this statement will be great.
- This approach has been studied only with one family of substrates (tetra-substituted 3-hydroxyphthalides presenting different substituents in the aromatic ring), practically one biocatalysts (CAL-B and one example with PS-IM) and only DBU used as racemisation base. The applicability of the study to other substrates will improve it in a decissive way.
- Authors compared in Table 1 a "classical" DKR with DBU for racemic 2a with the PDMS-modulated DKR. Apart that there is a reaction (III) in the table footnotes that does not appear in the Table body, this comparision is not very accurate as the "classical" DKR is performed in not optimal conditions. DBU is not the best racemisation catalyst for CAL-B and authors have indicated other tentative bases in which the DKR is performed (references 20-25). Clearly, the DBU-CAL-B classical DKR is not going to work in the same way as the novel methodology. Authors can also try to compare their process with a sequential DKR in which the DBU-racemization is conducted first and then the biocatalyzed acylation.
- Process has been scaled up to 4.0 mmol, which for this type of enzymes is not so high, but no studies on the PDMS stability and reusability for diferent cycles have been performed.
- The use of DBU in stoichiometric amounts can present some enviormental issues. In addition, the use of this base has shown certain side-reactions, have the authors observed any other product in the reaction medium?
- Finally, I am also missing some studies that are basic for the manuscript: Authors have not measured the diffusion rates through PDMS; There is no experimental calculation of the DBU-racemization kinetic and finally, there are no results on the retention of the DBU base in its compartment. Have been base-transfer observed?
Thus, the manuscript has to be improved in these ways to be suitable for publication in Nature Communications

No demonstration of DBU retention in Compartment-A

Version 1:

Reviewer comments:

Reviewer #1

(Remarks to the Author)
The author has made substantial and appropriate revisions, which significantly enhanced the quality of the manuscript. This reviewer supports its acceptance for publication in Nat. Commun. in current version.

Reviewer #2

(Remarks to the Author)
The authors did a good job revising the manuscript. Below are some remaining points:

1. Abstract: I recommend removing the word “synchronization” in the abstract, as it was done in some parts of the manuscript text. DKR is not about synchronization; it is about compatibility. In the DKR of the compounds mentioned in the manuscript, DBU alters the enantioselectivity of CALB. The membrane separation enables compatibility between DBU and CALB because of the physical separation. This clearly shows that what the authors addressed here is not synchronization between steps but compatibility: “However, its development has long been constrained by principal limitations, which need precise synchronization of racemization and resolution steps for rate matching.”

Change the sentence accordingly.

2. The same issue with “synchronization” applies on page 2, line 29: “However, the fundamental challenge of a conventional chemoenzymatic DKR process lies in synchronizing the two catalytic events of racemization.”

Modify the sentence by removing “synchronizing.” I commented on this also in the previous round of revision.

3. In the abstract: DKR is not a single-step process. It is a two-step process: racemization + acylation (in this case). If

concurrent, we talk about a one-stage, two-step process. If not concurrent (as in this work, because there is physical separation via a membrane), we talk about a two-stage, two-step process. Revise the sentence in the abstract accordingly: “By spatially separating racemization and resolution while enabling their collaborative operation within a single streamlined step (….)”.

4. In the abstract, there are again “grand” statements, such as: “(….) advancing chemoenzymatic DKR methodology but also provides a general and scalable platform with broad implications for pharmaceutical synthesis.”
This is not a “general” platform, and the implication is not so “broad.” I recommend making the statement in the abstract less bold.

5. Page 8, consider the sentence: “These findings demonstrate that the designed membrane-modulated DKR strategy could achieve the anticipated coordination of DBU-mediated racemization with CALB-catalyzed resolution in a two-stage manner, thereby enabling an efficient chemoenzymatic DKR to proceed that was previously incompatible under the conventional single-step chemoenzymatic conditions.”

“Single-step chemoenzymatic conditions” is incorrect. It is a two-step concurrent process. For terminology in cascade processes, see for example: Schrittwieser, J. H. et al. Artificial Biocatalytic Linear Cascades for Preparation of Organic Molecules. Chem. Rev. 118, 270–348 (2018).

Reviewer #4

(Remarks to the Author)
Authors have answered and addressed in deep all the questions and suggestions made by this refereee. They have included the study with a novel substrate (dihydrofuran), they have developed the PDMS stability and reusability study, and they have measured the diffusion rate of intermediate 2a through PDMS. All these modifications have been performed after a really extensive experimental work which is shown in the revised version. From my side, manuscript is suitable for publications with all these modifications.

Open Access This Peer Review File is licensed under a Creative Commons Attribution 4.0 International License, which permits use, sharing, adaptation, distribution and reproduction in any medium or format, as long as you give appropriate credit to the original author(s) and the source, provide a link to the Creative Commons license, and indicate if changes were made.
In cases where reviewers are anonymous, credit should be given to 'Anonymous Referee' and the source.
The images or other third party material in this Peer Review File are included in the article’s Creative Commons license, unless indicated otherwise in a credit line to the material. If material is not included in the article’s Creative Commons license and your intended use is not permitted by statutory regulation or exceeds the permitted use, you will need to obtain permission directly from the copyright holder.
To view a copy of this license, visit https://creativecommons.org/licenses/by/4.0/

Point-by-point response:

Responses to the comment of Reviewer 1

Reviewer #1 (Remarks to the Author):

Comments: Chemoenzymatic dynamic kinetic resolution (DKR) is a powerful tool to combine chemocatalysis and biocatalysis for the construction of chiral skeletons from the racemic mixtures, however, compatibility of dual systems is the general issue, which has to be overcome. Herein, the authors developed a membrane-modulated strategy, which presents an alternative design to achieve chemoenzymatic DKR transformations by spatially separating racemization and resolution. Finally, the collaborative operation within a single step enables the DKR approach to allow the efficient synthesis of 3-hydroxyphthalide esters. The design is smart, and substrate scope is broad. Based on it, this reviewer supports the acceptance of the manuscript as publication in the high-impact journal of Nat. Commun. after minor revisions, by addressing the following concerns.

1. In Scheme 1A, the authors showed a significant decrease in the enantiomeric excess of the product when DBU is added stepwise in a one-pot reaction. This tendency could be plausibly attributed to DBU directly participating in a non-enzymatic background esterification. The authors hypothesize that the membrane prevents DBU, the substrate, and the acetate ester from coexisting in the same compartment, thereby suppressing this background reaction. However, in my opinion, this postulate requires further validation. Based on the provided description, while the substrate can permeate the membrane to access the DBU compartment, it remains unclear whether the acyl donor can also diffuse through the membrane. If the acyl donor is permeable, the background reaction could still occur. The authors should clarify how the membrane compartmentalization successfully enables the DKR transformation under these conditions.
2. This reviewer is concerned about the practical application of the products. It appears that after the chiral ester is de-esterified to the alcohol, the resulting compound is prone to racemization under basic conditions, leading to a loss of optical activity. The authors should consider this possibility, and provide evidence to demonstrate the potential for application of the chiral products.
3. Some related papers (J. Am. Chem. Soc. 2015, 490 137, 3996−4009; ACS Cent. Sci. 2024, 10, 2099−2110; Chin. Chem. Lett. 2024, 35, 109160; Chin. Chem. Lett. 2025, 36, doi.org/10.1016/j.cclet.2025.111943.) could be cited.

Question 1:

In Scheme 1A, the authors showed a significant decrease in the enantiomeric excess of the product when DBU is added stepwise in a one-pot reaction. This tendency could be plausibly attributed to DBU directly participating in a non-enzymatic background

esterification. The authors hypothesize that the membrane prevents DBU, the substrate, and the acetate ester from coexisting in the same compartment, thereby suppressing this background reaction. However, in my opinion, this postulate requires further validation. Based on the provided description, while the substrate can permeate the membrane to access the DBU compartment, it remains unclear whether the acyl donor can also diffuse through the membrane. If the acyl donor is permeable, the background reaction could still occur. The authors should clarify how the membrane compartmentalization successfully enables the DKR transformation under these conditions.

Response: Thank you very much for your valuable comments, which have helped us further improve the quality and completeness of the manuscript. Yes, it is an important question to clarify the background reaction of non-enzymatic esterification in both compartments. According to the suggestion, we investigated the transmembrane permeation of DBU from Compartment-**A** to Compartment-**B** and the acyl donor (acetic anhydride) from Compartment-**B** to Compartment-**A** through two modes.

Regarding the issue of DBU permeation (from Compartment-**A** to Compartment-**B**), it was found that DBU could hardly permeate through the PDMS membrane to reach Compartment-**B**. GC analysis revealed that only 4.8% of DBU crossed the membrane into Compartment-**B** within 36 h, as following GC analysis in Figure S1a. This means that less than 1.0 mol% of DBU permeated into Compartment-**B**. Notably, only a trace amount of DBU in Compartment-**B** had a negligible effect on the resolution efficiency of the CALB enzyme, as proven by the control reactions (Please see Scheme 1B in the manuscript). Therefore, as the reviewer mentioned, the background reaction could only occur if the acyl donor (acetic anhydride) from Compartment-**B** permeated into Compartment-**A**.

Also, the GC analysis of DBU has been placed in the Supporting Information as Figure S1a, as follows.

Figure S1a. The GC analysis of DBU in the control experiment before reaction at Compartment-**A** *versus* the GC analysis of DBU in the control experiment after reaction for 36 h at Compartment-**B**. (Conditions. In compartment-**A**, DBU (0.20 mmol) in 2.0 mL of toluene, 25 °C, 36 h. In compartment-**B**, 2.0 mL of toluene, 25 °C, 36 h.)

The content ratio after reaction (the peak area of DBU after reaction for 36 h divided by the peak area of DBU before the reaction**)** = 5614366/116754976 = 4.8%**.**

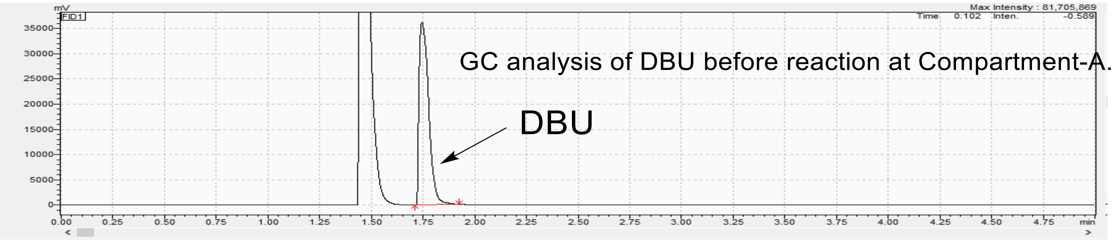

峰号	保留时间	面积	高度	标记	浓度	浓度单位	化合物ID号	化合物名	面积%
1	1.745	116754976	36120607	M	100.000				100.000
总计		116754976	36120607		100.000				100.000

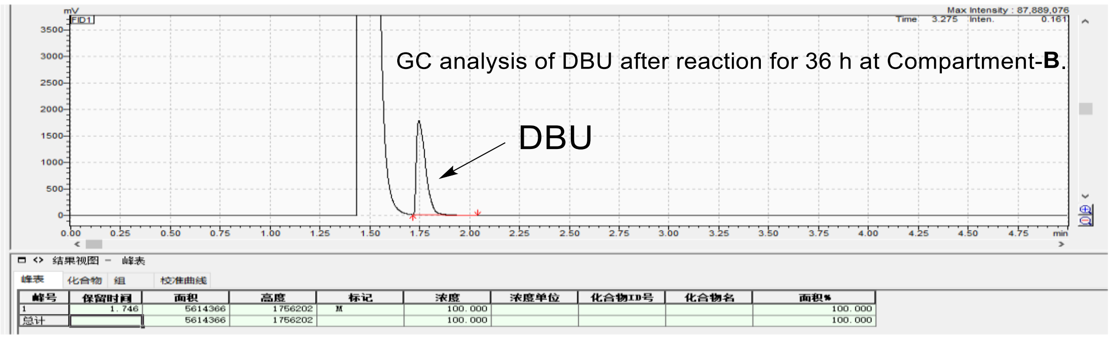

Regarding the permeation of the acyl donor (acetic anhydride) from Compartment-**B** to Compartment-**A**, we employed a similar approach to investigate the transfer of acetic anhydride into Compartment-**A**. The results indicated that the permeation of the acyl donor (acetic anhydride) into Compartment-**A** was also negligible. GC/MS analysis revealed that only 0.2% of acetic anhydride crossed the membrane into Compartment-**A,** as shown in the following Figure S1b. This means that approximately 0.8 mol% of acetic anhydride (less than 1.0 mol%) permeated into Compartment-**A** during the DKR process.

Figure S1b. The GC/MS analysis of acetic anhydride in the control experiment before reaction at Compartment-**B** *versus* the GC/MS analysis after reaction for 36 h at Compartment-**A**. (Conditions. In compartment-**A**, 2.0 mL of toluene, 25 °C, 36 h. In compartment-**B**, Novozym-435 (CALB) (40.0 mg), acetic anhydride (0.40 mmol) in 2.0 mL of toluene, 25 °C, 36 h.)

The content ratio after reaction (the peak area of acetic anhydride after reaction for 36 h at Compartment-**A** divided by the peak area of acetic anhydride before the reaction at Compartment-**B)** = 24848/10039269 = 0.2%.)

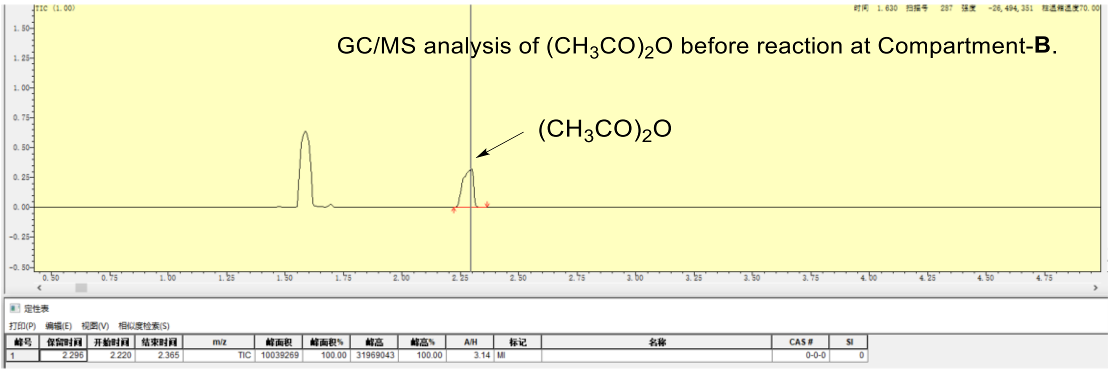

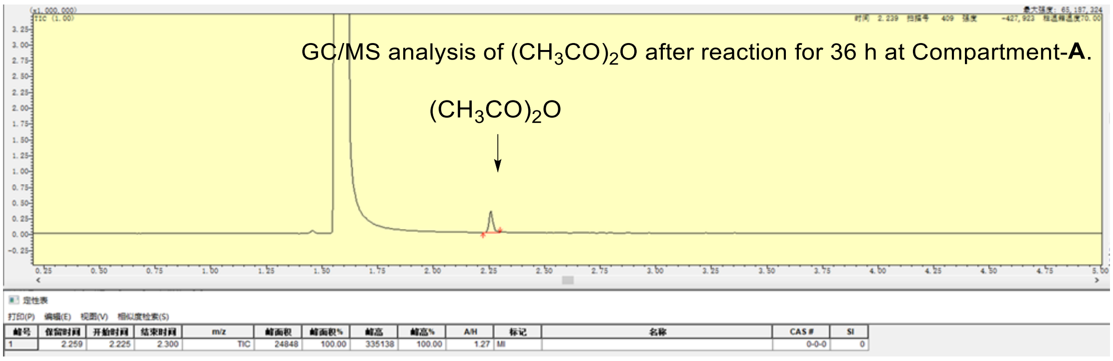

This observation should be primarily attributed to two key reasons. Firstly, in Compartment-**B**, acetic anhydride under conditions with the CALB enzyme present predominantly exists in the form of acetic acid because the enzyme requires acylation to form acetylated CALB for the enzyme-mediated resolution esterification (ref. 46. *J. Mol. Graph. Model.* **57**, 9–19 (2015)). In other words, in Compartment-**B**, acetic anhydride was largely consumed and converted into acetic acid in the presence of CALB enzyme, as proven by a GC/MS analysis as following Figure. Secondly, due to its high polarity and water solubility, acetic acid is unable to permeate the hydrophobic, lipid-soluble PDMS membrane.

Figure. The GC/MS analysis of acetic anhydride and acetic acid in the control experiment after reaction at Compartment-**B** (Conditions. In compartment-**A**, **2a** (0.20 mmol), DBU (0.20 mmol) in 2.0 mL of toluene, 25 °C. In compartment-**B**, Novozym-435 (CALB) (40.0 mg), and acetic anhydride (0.40 mmol) in 2.0 mL of toluene, 25 °C, 36 h.)

The ratio of acetic anhydride to acetic acid after reaction = 7271101/1233853 ≈ **1/6**

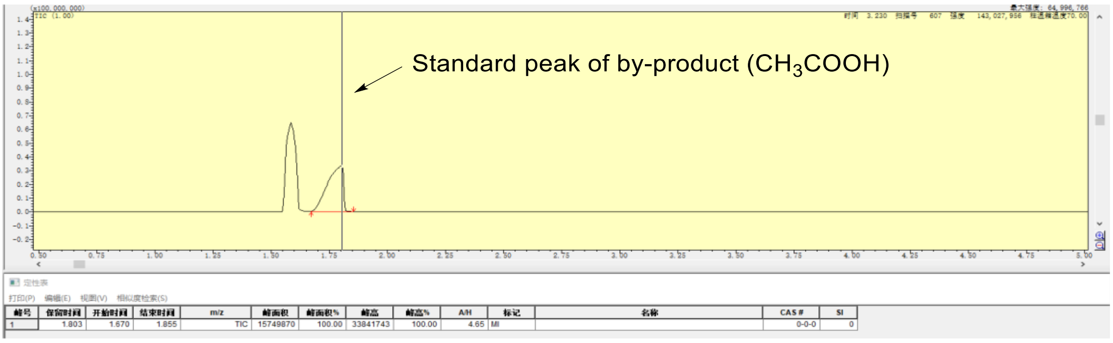

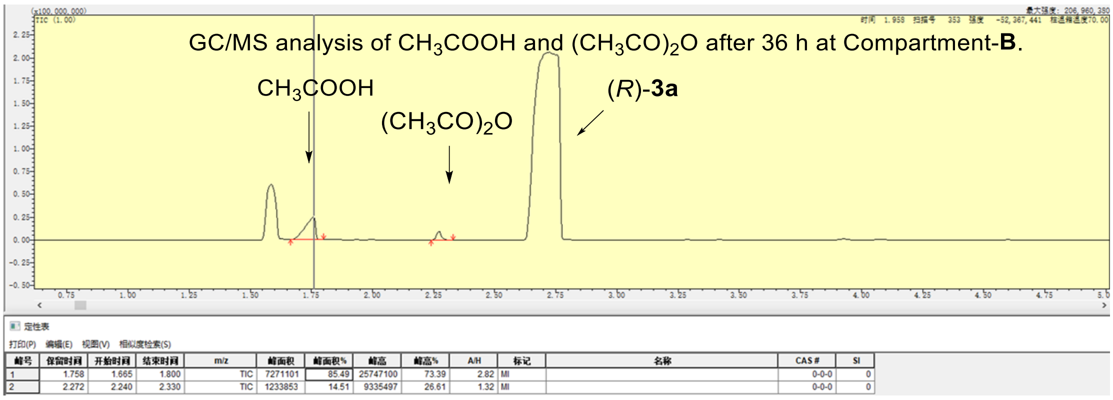

The above results demonstrated that the background reaction *via* the permeation of acetic anhydride into Compartment-**A** was also negligible, as proven by the control reactions, as the following Scheme. It was observed that in the presence of 1 mol% acetic anhydride (which exceeds the 0.8 mol% acetic anhydride content detected in the above GC/MS analysis), only a trace amount (less than 2%) of the racemic side product **3a** was formed. This further confirmed that the permeation of acetic anhydride into Compartment-**A** had a negligible effect on the reaction.

Scheme. Control experiments for the comparative traditional single-step DKR process

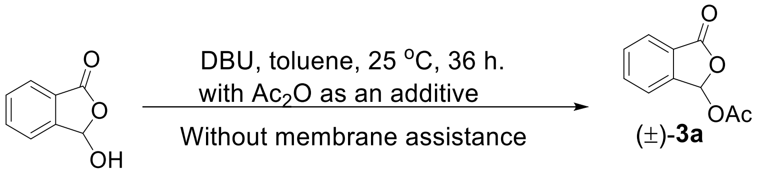

(±)-2a

2.0 equivalents of Ac_2O (200 mol%): 68% yield, 3% *ee.*
0.01 equivalents of Ac_2O (1 mol%): less than 2% yield, 0% *ee.*

Also, the statement has been revised in the revised manuscript as follows.

Revise (page 8, line 9): "Finally, in the evaluation of an integrated PDMS-modulated DKR (**II**) based on the optimizations (see Supplementary Experimental and Table S2, SI), it was observed that the model reaction could steadily provide the final (*R*)-**3a** with a 91% yield and 99% *ee* (entry 8)."

To: "Finally, in the evaluation of an integrated PDMS-modulated DKR (**II**) based on the optimizations (see Supplementary Experimental and Table S2, SI), it was observed that the model reaction could steadily provide the final (*R*)-**3a** with a 91% yield and 99% *ee* (entry 8), in which the permeation of 4.8% DBU from Compartment-**A** to Compartment-**B** and 0.2% acetic anhydride from Compartment-**B** to Compartment-**A** had a negligible impact on the enantioselectivity of the product, as confirmed by GC/MS analysis (see Supplementary Figure S1, SI)."

Question 2:

This reviewer is concerned about the practical application of the products. It appears that after the chiral ester is de-esterified to the alcohol, the resulting compound is prone to racemization under basic conditions, leading to a loss of optical activity. The authors should consider this possibility, and provide evidence to demonstrate the potential for application of the chiral products.

Response: Yes, we agree with the reviewer's perspective. The de-esterified chiral alcohols can readily undergo a ring-opening reaction under alkaline conditions, leading to a dynamic tautomeric equilibrium and loss of optical activity, which significantly limits the practical application potential of these chiral products. Of course, exploring the application of chiral esterified products might be another story. According to the reviewer's suggestion, in this study, we attempted to derivatize the chiral esterified products via two approaches, as the following Scheme. One approach involves direct functionalization on the aromatic ring, with a specific example being the functionalization of hydroxy-containing substrate (*R*)-**3z** as follows. The other approach involves directly functionalizing the chiral quaternary carbon center of (*R*)-**6e**, with an example being the use of coupling reagents to perform derivatization via carbon-carbon coupling. The two examples above illustrate the possibilities for

expanding the utility of the chiral products, thereby demonstrating their broader application potential.

Scheme. The derivations of chiral products.

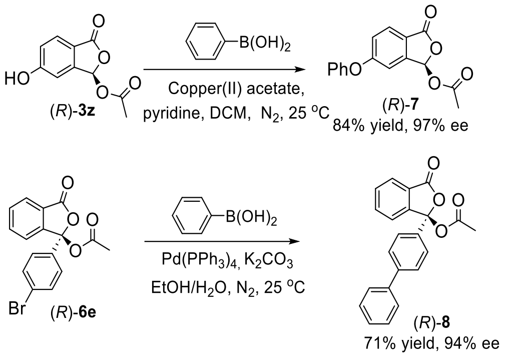

Additionally, the extended products had been incorporated into Scheme 4, and the corresponding description has been updated in the revised manuscript as follows.

Add (page 20, line 2): "In addition to the preparation of chiral phthalide prodrugs, the obtained chiral products could also be further derivatized to synthesize their analogues (Equation 2-3 in Scheme 4). It was found that (*R*)-**3z** could afford product (*R*)-**7** through an etherification reaction, while (*R*)-**6e** yielded the corresponding coupling product (*R*)-**8** through a coupling reaction (see Supplementary Experimental and Figs. S8-S9, SI).[58-60]"

Revise (page 19, Scheme 4):

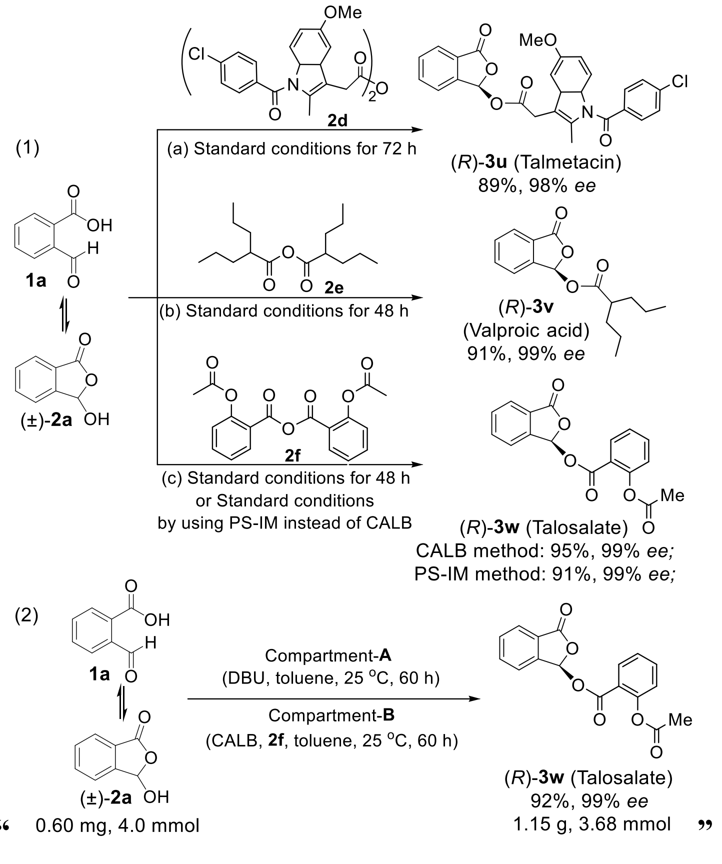

"   "

<u>To:</u>

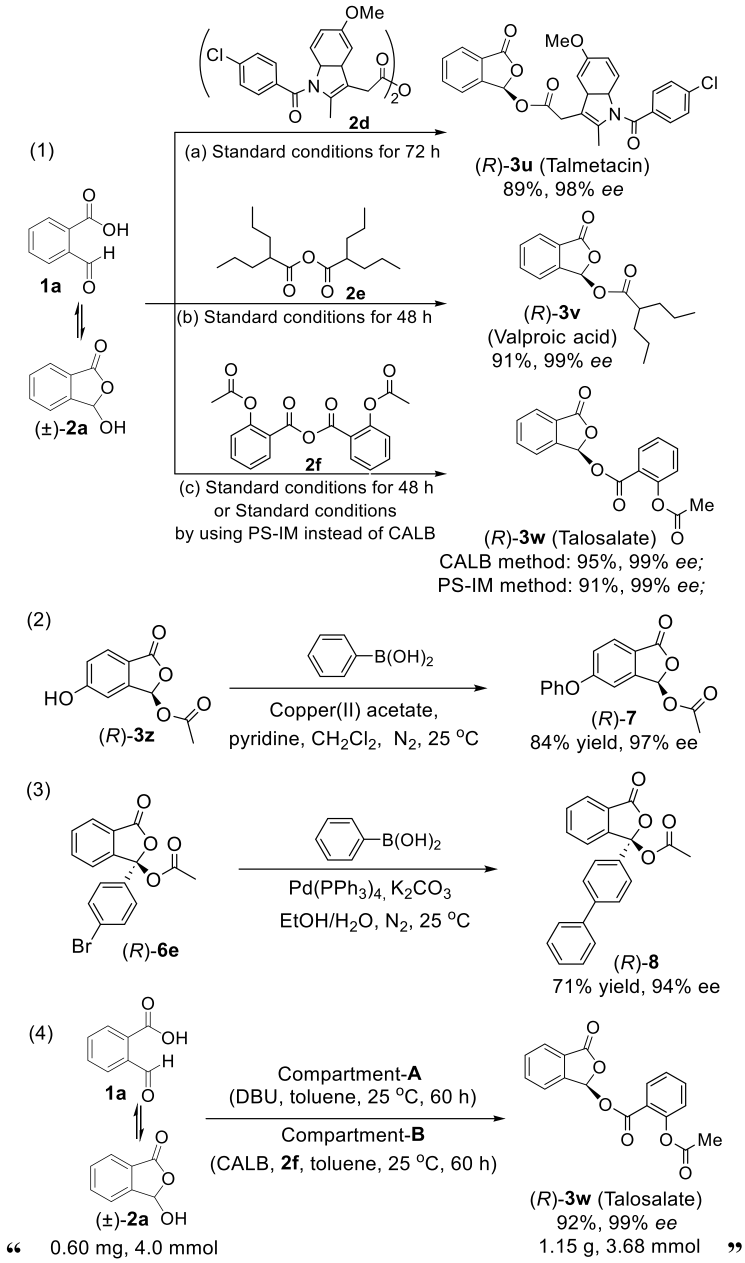

<u>Also,</u> the derivatization synthesis procedures of products (*R*)-**7** and (*R*)-**8** have been provided in the Experimental Section of the Supporting Information as follows.

<u>6.3 General procedure for the preparation of products (*R*)-7 and (*R*)-8.</u>

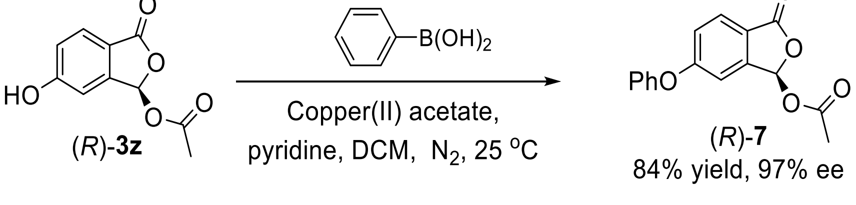

Under nitrogen protection, (*R*)-**3z** (0.20 mol), phenylboronic acid (0.40 mol), pyridine (0.80 mol), and copper(II) acetate monohydrate (0.20 mol) were successively added to

dichloromethane (10 mL). The mixture was stirred at 25 °C for 48 h. After completion, the reaction was quenched with 2 N dilute hydrochloric acid, and the solids were removed by filtration. The combined organic phases were extracted with CH_2Cl_2 (5 mL × 3), dried over anhydrous magnesium sulfate, filtered, and concentrated under reduced pressure. The crude product was purified by silica gel column chromatography to afford the target product (*R*)-**7** (48.3 mg, 0.17 mmol) in 84% yield.

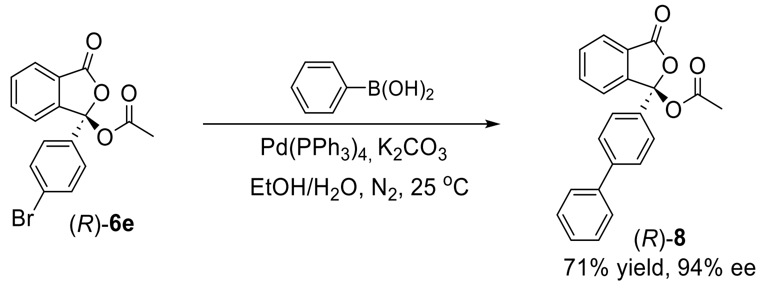

Under nitrogen protection, (*R*)-**6e** (0.20 mmol), phenylboronic acid (0.40 mmol), $Pd(PPh_3)_4$ (0.06 mmol), and potassium carbonate (0.20 mmol) were added to a mixture of ethanol/water (1:3, v/v, 10 mL). The reaction was stirred at 25 °C for 48 h. After completion, the mixture was poured into water and extracted with CH_2Cl_2 (5 mL × 3). The combined organic phases were dried over anhydrous magnesium sulfate, filtered, and concentrated under reduced pressure. The crude product was purified by silica gel column chromatography to afford the target product (*R*)-**8** (48.8 mg, 0.14 mmol) in 71% yield.

Also, the characterization data of (*R*)-**7** and (*R*)-**8** have been provided in the Supporting Information as Figures S8-S9 as follows.

Figure. Characterizations of derivatized chiral products.

(*R*)-7: (*R*)-3-oxo-6-phenoxy-1,3-dihydroisobenzofuran-1-yl acetate

(*R*)-3-oxo-6-phenoxy-1,3-dihydroisobenzofuran-1-yl acetate (HPLC: Chiralpak IC, detected at 210 nm, eluent: n-hexane/2-propanol = 90/10, flow rate = 1.0 mL/min, 25°C).

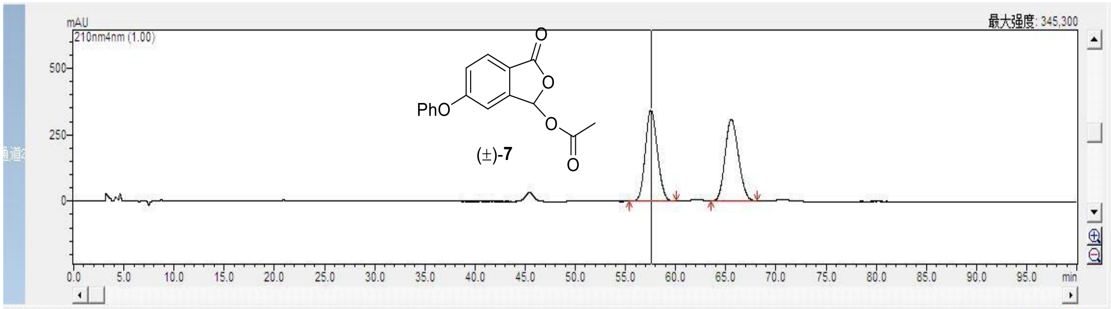

化合物表视图

ID#	名称	保留时间	峰#	面积	高度	面积%
1	RT57.537	57.537	1	28864617	345587	50.0580
2	RT65.536	65.536	2	28797762	308907	49.9420

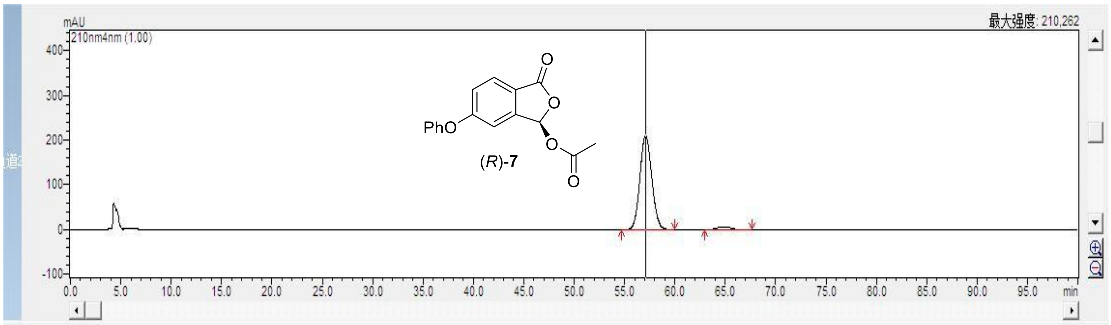

化合物表视图

ID#	名称	保留时间	峰#	面积	高度	面积%
1	RT57.081	57.081	1	17331385	210794	98.6779
2	RT64.895	64.895	2	232217	12593	1.3221

<u>(*R*)-8: (*R*)-1-([1,1'-biphenyl]-4-yl)-3-oxo-1,3-dihydroisobenzofuran-1-yl acetate.</u>

(*R*)-1-([1,1'-biphenyl]-4-yl)-3-oxo-1,3-dihydroisobenzofuran-1-yl acetate **(**HPLC: Chiralpak IC, detected at 210 nm, eluent: n-hexane/2-propanol = 90/10, flow rate = 1.0 mL/min, 25°C).

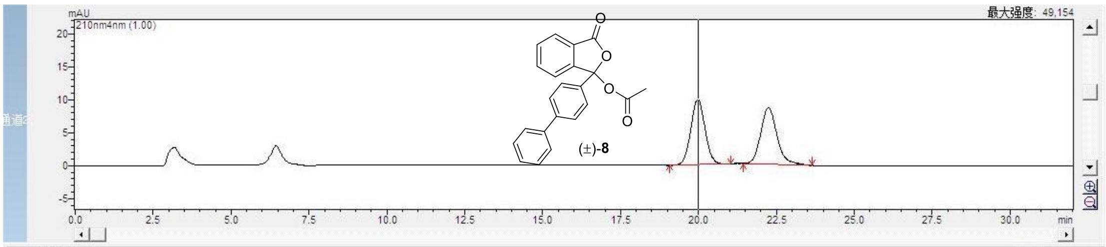

化合物表视图

ID#	名称	保留时间	峰#	面积	高度	面积%
1	RT19.986	19.986	1	312455	9875	49.9818
2	RT22.230	22.230	2	312682	8578	50.0182

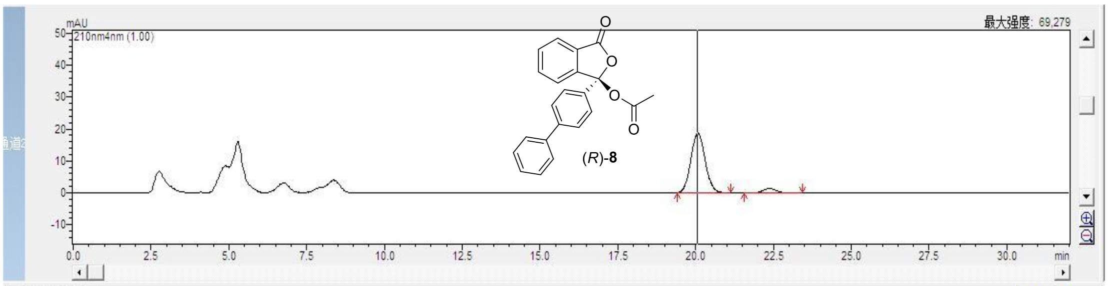

化合物表视图

ID#	名称	保留时间	峰#	面积	高度	面积%
1	RT20.064	20.064	1	598824	18726	96.9849
2	RT22.330	22.330	2	18617	1964	3.0151

<u>Also, the references have been cited as follows</u>

58. Evans, D. A., Katz, J. L. & West. T. R. Synthesis of diaryl ethers through the copper-promoted arylation of phenols with arylboronic acids. An expedient synthesis of thyroxine. *Tetrahedron Lett.* **39**, 2937-2940 (1998).

59. Elumalai, V. & Hansen, J. H. Synthesis of 5,7-diarylindoles via Suzuki–Miyaura coupling in water, *Org. Biomol. Chem.* **19**, 10343–10347 (2021).

60. Sun, Z., Zang, Q., Luo, Q., Lv, C., Cao, F., Song, Q., Zhao, R., Zhang, Y. & Wong, W. Emission enhancement and high sensitivity of a p-conjugated dye towards pressure: the synergistic effect of supramolecular interactions and H-aggregation, *Chem. Commun.* **55**, 4735-4738 (2019).

Question 3:

Some related papers (J. Am. Chem. Soc. 2015, 490 137, 3996−4009; ACS Cent. Sci. 2024, 10, 2099−2110; Chin. Chem. Lett. 2024, 35, 109160; Chin. Chem. Lett. 2025, 36, doi.org/10.1016/j.cclet.2025.111943.) could be cited.

Response: According to the reviewer's suggestion, the literature on chemoenzymatic dynamic kinetic resolution has been cited, and the statements have also been revised as follows.

The statement has been revised in the revised manuscript as follows.

Revise (page 1, line 7): "However, the fundamental challenge of a conventional DKR process lies in synchronizing the two catalytic events of racemization and resolution in accordance with the core principles: (i) the KR must display a sufficient enantioselectivity (E value = $k_{fast}/k_{slow} \geq 20$), and the rate of racemization (k_{rac}) must be at least 10 times faster than the enzyme-catalyzed reaction of the slow reacting enantiomer (k_{slow}) (first limitation), and (ii) the enzyme and the racemization catalyst must be compatible with one another, and the racemization catalyst must not react with the product formed from the resolution) (second limitation)."

To: "However, the fundamental challenge of a conventional DKR process lies in synchronizing the two catalytic events of racemization and resolution in accordance with the core principles (Figure 1A):[9-12] (i) the KR must display a sufficient enantioselectivity (E value = $k_{fast}/k_{slow} \geq 20$), and the rate of racemization (k_{rac}) must be at least 10 times faster than the enzyme-catalyzed reaction of the slow reacting enantiomer (k_{slow}) (first limitation), and (ii) the enzyme and the racemization catalyst must be compatible with one another, and the racemization catalyst must not react with the product formed from the resolution) (second limitation)."

Add references (ref. 9-12): "

9. Verho, O. & Backvall, J. E. Chemoenzymatic dynamic kinetic resolution: A powerful tool for the preparation of enantiomerically pure alcohols and amines, *J. Am. Chem. Soc.* **137**, 3996–4009 (2015).
10. Wang, K., Wang, W., Lou, D. K., Zhang, J., Chi, C. L., Backvall, J. E., Sheng, X. & Zhu, C. Overcoming the limitations of transition-metal catalysis in the chemoenzymatic dynamic kinetic resolution (DKR) of atropisomeric bisnaphthols, *ACS Cent. Sci.* **10**, 2099−2110 (2024).
11. Zhang, J., Zheng, Z. Y. & Zhu, C. Stereochemical editing: Catalytic racemization of secondary alcohols and amines, *Chin. Chem. Lett.* **35**, 109160 (2024).

12. Lou, D. K., Wu, H. Z. & Zhu, C. Design and construction of difunctionalized C-N atropisomers via lipase-catalyzed desymmetrization and kinetic resolution of N-naphthol-carbazoles, *Chin. Chem. Lett.* doi.org/10.1016/j.cclet.2025.111943."

Responses to the comment of Reviewer 2

Reviewer #2 (Remarks to the Author):

Comments: This review was conducted with an Early Career Researcher participating in the co-review initiative of Nat. Commun. I, as the senior scientist, am responsible for the final version of this report, which is the result of a single evaluation by me and my coworker, our follow-up discussion, and the final writing of this report. According to the instructions, we do not give a specific recommendation to the editor for acceptance or rejection; rather, we critically analyze this work in terms of novelty, potential impact, and overall quality of the research, highlighting strengths and limitations. As a general comment, I believe that the authors have made heavy use of an AI language model to write this manuscript. This is evident from the narrative, which is lengthy, repetitive, pompous, and also tries to "oversell" the novelty and impact of the work in some sections. I strongly recommend revising the entire manuscript using a more balanced and sober narrative that is more in line with academic writing.

In general, this is an excellent work in terms of development of the system, catalytic efficiency, and substrate scope covered. However, I have doubts regarding the real novelty and potential impact in the field in relation to the state of the art for the reasons explained below.

Comments

1. The introduction is very lengthy, repetitive, and difficult to follow. There is a lot of "unfocused" narrative. The key points are diluted in the lengthy text, making comprehension and grasping the main points very difficult. I suggest shortening the introduction and concisely discussing the state of the art (i.e., what the current limitations are and how this work can help to solve such limitations). There is no need to use six A4 pages to discuss all this. Half the length of the text would be more than enough. There is also a lot of emphasis on the discussion of compartmentalized dynamic kinetic resolution. Many statements are too "grand," while the authors are essentially presenting a work based, in many parts, on already well-known technology. Lipases have been among the first enzymes used in biocatalysis; resolution of enantiomers with enzymes is a classic process, and DKR is almost 30 years old if we consider the work by Bäckvall or the first report by Williams and coworkers. The first chemoenzymatic DKR was accomplished with 1-phenylethanol using a lipase for the acyl-transfer reaction and a rhodium complex for racemization in 1996: (P. M. Dinh, J. A. Howarth, A. R. Hudnott, J. M. J. Williams, W. Harris, Tetrahedron Lett. 1996, 37, 7623–762). The use of membrane reactors in batch is also known. Notably, similar types of DKR were conducted with DBU as a racemization catalyst in flow reactors with excellent efficiency. An example is P. Falus et al., Adv. Synth. Catal. 2016, 358, 1608–1617. Using two

reactors in flow is another way to achieve compartmentalization, which is what the authors did in their paper using a membrane in a batch system. Therefore, conceptually, there is not so much novelty in compartmentalization of chemo- and biocatalysts. The authors should also mention flow-chemistry applications of DKR. There is also another limitation. While a flow-reactor methodology can in principle deliver 99% conversion of the product in optically pure form, the batch solution with a membrane slows down significantly around 90% conversion due to diffusional limitations (i.e., the kinetics of diffusion at low concentration of remaining substrate becomes severely rate-limiting). The authors must mention this limitation. In a real-world application, incomplete conversion requires further isolation and purification steps, making the process less appealing.

2. The text is also redundant in the Results and Discussion. There are three main key points: 1) DBU influences the catalytic activity and, maybe, the enantioselectivity of CALB; 2) DBU can act as a catalyst for the esterification itself with no selectivity; 3) points 1 and 2 impede the classical DKR, and the only way to do it is through compartmentalization of the two catalysts.
   These are three simple concepts to convey, yet the authors use very long text, repeating the same things several times. The text is redundant and rather pompous. A more concise and scientifically sober narrative would be advisable.
3. Page 2: the authors state: "However, the fundamental challenge of a conventional DKR process lies in synchronizing the two catalytic events of racemization and resolution in accordance with the core principles: (i) the racemization rate must exceed the resolution rate to ensure the selective and rapid transformation of the favored enantiomer (first limitation)." This sentence is not conceptually correct in relation to DKR, in general. The racemization rate must be significantly higher than the rate for the conversion of the "wrong" (undesired) enantiomer by the enzyme. However, the racemization rate can be (and normally is) lower than the rate for the conversion of the desired enantiomer. Citing from Verho and Bäckvall, J. Am. Chem. Soc. 2015, 137, 3996−4009: "However, the design of a successful DKR system is far from simple, given that the following requirements must be fulfilled: (i) the KR must display a sufficient enantioselectivity (E value = kfast/kslow ≥ 20); (ii) the enzyme and the racemization catalyst must be compatible with one another; (iii) the rate of racemization (krac) must be at least 10 times faster than the enzyme-catalyzed reaction of the slow-reacting enantiomer (kslow); and (iv) the racemization catalyst must not react with the product formed from the resolution."
   In conclusion, there is no mention of kfast, strictly speaking. I think what the authors mean is that, in their specific case, it is necessary that the racemization is faster than kfast because the racemization catalyst (DBU) can also catalyze the esterification but in a non-enantioselective manner, thus eroding the ee. In conclusion, the

statement in the manuscript should be modified accordingly.

4. - Page 4: “In such systems, the substrate forms an unstable hemiacetal intermediate with the NHC catalyst, and the resulting energy difference between matched and mismatched isomers promotes efficient racemization of the unfavored enantiomer, ultimately delivering high DKR efficiency”. NHC has never been introduced so far as acronym.
5. Page 6: “Beyond shielding, the selective permeability of the PDMS membrane leverages its dynamic mass-transfer capability, permitting the diffusion of targeted intermediate (3-hydroxyisobenzofuranone), while confining catalysts, substrates, and other components to their respective compartments”.
   It would be beneficial for the paper and the readers to add to Figure 1 letters for each molecule and then add it in the text, 3-hydroxyisobenzofuranone (xxx).
6. Page 7: “As presented in Figure 1C, by utilizing the dynamic tautomeric equilibrium between 1a and 2a, a practical PDMS-modulated chemoenzymatic DKR process as a representative ...” In Figure 1, the authors forgot to insert the numbers in the molecules for 1a and 2a, present for example in Table 1. Please add it to the table to render the comprehension easier.
7. Page 9: “Notably, these comparisons revealed that the second-step biocatalysis was severely suppressed in the presence of ...” I guess the authors wanted to write the second biocatalytic step instead of second-step biocatalysis. Please rewrite to make it clear.
8. Page 9: “Finally, in the evaluation of an integrated PDMS-modulated DKR (II) based on the optimizations (see Supplementary Experimental and Table S2, SI), it was observed that the model reaction could steadily provide the final (R)-3a with a 91% yield and 99% ee (entry 8), which was slightly superior to the outcomes obtained with other DBU loadings (entry 8 versus 10 entries 9 and 10)”.
   I don’t think the results obtained in entries 8, 9 and 10 are statistically different. How would you explain this different outcome? From my perspective, it is just a normal experimental fluctuation of data. Statistical analysis from more data of same type of experiments would be needed to prove that.
9. Page 10: “In addition, it was also found that the membrane thickness had a certain impact on the PDMS-modulated DKR process, in which the reaction using a 0.40 mm-thick membrane yielded the best results compared to the reactions with other membrane thicknesses (entry 8 versus entries 11 and 12)”. How would the authors explain this phenomenon?
10. Page 12: again the same mistake in my opinion: “To elucidate the unique advantage of independent compartmentalization in circumventing the conventional requirement that the racemization rate must exceed the resolution rate (i.e., krac > kfast to ensure continuous racemization of the unreacted enantiomer).” This is not

correct: krac >> kslow, but krac can also be equal to or smaller than kfast in a conventional DKR. It is the specific case of this DKR using DBU as the racemization catalyst that creates this additional requirement. It should be explained clearly.

11. Page 13: "Thus, the combination of this membrane-mediated device with the dynamic tautomerization facilitated by even any racemization base catalyst in Compartment-A was sufficient to sustain an efficient DKR process, thereby establishing a new mechanistic paradigm in which a concentration gradient, rather than relative rate control, drives the process." I do not think that this is a new mechanistic paradigm. It is based on the law of diffusion, and racemization is based on the concept of entropy—very classical concepts from the last century and earlier that can be found in textbooks. Again, an exaggerated narrative, in my opinion.
12. Page 15: "This equilibrium facilitates the transmembrane migration of racemic 2 while retaining 1 in Compartment-A".
    Why is 1 retained? Do you have proof of the fact that 1 doesn't pass through the membrane? Did you try to put 1 in compartment B?
13. Page 19: "Third, for the racemic tetra-substituted 3-hydroxyphthalides (5a–5h), which was difficult to access by conventional chemoenzymatic strategy, the membrane-modulated DKR also exhibited remarkable tolerance, consistently furnishing the challenging chiral tetra-substituted 3-hydroxyphthalide esters ((R)-6a–(R)-6h) in acceptable yields with excellent enantioselectivities (Equation 3, Scheme 3)." This limitation is attributable to CALB. Why does the DKR of tetra-substituted 3-hydroxyphthalides become easier with the membrane reactor if the catalytic rate depends only on the enzyme? Can the authors comment on that and provide an explanation?
14. Page 21, but also in other sections of the manuscript: "The independent compartmentalization maintains kinetically coupled yet isolated environments, sustaining a substrate concentration gradient that drives DKR forward without kinetic synchronization." The use of the definition "kinetic synchronization" is not standard in DKR. The authors should better define what they mean by "kinetic synchronization." It would appear that "kinetic synchronization" is not possible in DKR with catalysts compartmentalization, while in contrast kinetic resolution and racemization must always run concurrently. In conclusion, either the authors can explain in a concrete way what this is about or such sentences must be removed from the manuscript.
15. Page S4: Why do you need a balloon filled with air? What happen if you close the system completely? Why did you filter the solution afterward? What is the solid part coming from? Only the enzyme?
16. Page S27: the two HPLC chromatograms don't testify the production of one of the

enantiomer; the retention times are different between the racemic mixture and the enantioselective one (with only one peak).

17. Page S32: the two HPLC chromatograms don't testify the production of one of the enantiomer; the retention times are different between the racemic mixture and the enantioselective one (with only one peak).
18. Page S33: Why are there two different retention time for the same molecule? Please explain more carefully.
19. Page S40: In all the previous cases the R enantiomer is the first one; why is it the second peak on this specific example? Are you sure this is not the S enantiomer?
20. Page S68, S69: In all the previous cases the R enantiomer is the first one; why is it the second peak on this specific example? Are you sure this is not the S enantiomer?
21. Page S71: the two HPLC chromatogram don't testify the production of one of the enantiomer; the retention times are different between the racemic mixture and the reaction sample (with only one peak).
22. Page S80: the two HPLC chromatogram don't testify the production of one of the enantiomer; the retention times are different between the racemic mixture and the reaction sample (with only one peak).

Question 1:

The introduction is very lengthy, repetitive, and difficult to follow. There is a lot of "unfocused" narrative. The key points are diluted in the lengthy text, making comprehension and grasping the main points very difficult. I suggest shortening the introduction and concisely discussing the state of the art (i.e., what the current limitations are and how this work can help to solve such limitations). There is no need to use six A4 pages to discuss all this. Half the length of the text would be more than enough. There is also a lot of emphasis on the discussion of compartmentalized dynamic kinetic resolution. Many statements are too "grand," while the authors are essentially presenting a work based, in many parts, on already well-known technology. Lipases have been among the first enzymes used in biocatalysis; resolution of enantiomers with enzymes is a classic process, and DKR is almost 30 years old if we consider the work by Bäckvall or the first report by Williams and coworkers. The first chemoenzymatic DKR was accomplished with 1-phenylethanol using a lipase for the acyl-transfer reaction and a rhodium complex for racemization in 1996: (P. M. Dinh, J. A. Howarth, A. R. Hudnott, J. M. J. Williams, W. Harris, Tetrahedron Lett. 1996, 37, 7623–762). The use of membrane reactors in batch is also known. Notably, similar types of DKR were conducted with DBU as a racemization catalyst in flow reactors with excellent efficiency. An example is P. Falus et al., Adv. Synth. Catal. 2016, 358, 1608–1617. Using two reactors in flow is another way to achieve compartmentalization, which is what the authors did in their paper using a membrane in a batch system.

Therefore, conceptually, there is not so much novelty in the compartmentalization of chemo- and biocatalysts. The authors should also mention flow-chemistry applications of DKR. There is also another limitation. While a flow-reactor methodology can in principle deliver 99% conversion of the product in optically pure form, the batch solution with a membrane slows down significantly around 90% conversion due to diffusional limitations (i.e., the kinetics of diffusion at low concentration of remaining substrate becomes severely rate-limiting). The authors must mention this limitation. In a real-world application, incomplete conversion requires further isolation and purification steps, making the process less appealing.

Response: Thank you very much for your valuable comments, which have helped us further improve the quality. According to the reviewer's suggestions, we have shortened the Introduction and streamlined the discussion, reducing its length from seven pages to less than five pages. Please see the revised Introduction as follows. In addition, concerning the question about the flow-chemistry applications of DKR, we have also stated its advantages and provided a comparative analysis of the limitations of the membrane-mediated approach, and references have been cited as follows.

Revise Introduction (page 2, line 1): "The selective preparation of enantiomerically pure compounds remains a central objective in modern synthetic chemistry, driven by the profound influence of chirality on the biological, pharmacological, and functional properties of target molecules.[1-3] As shown in Figure 1, classical kinetic resolution (KR) methodology is intrinsically restricted to a theoretical maximum yield of 50%. By contrast, dynamic kinetic resolution (DKR) integrates KR with an *in-situ* racemization process, enabling complete (theoretical 100%) conversion of racemic substrates into a single enantiomer (Figure 1A).[4-6] Notably, a chemoenzymatic DKR strategy not only overcomes the intrinsic yield limitation of enzymatic KR but also preserves the enzyme's exquisite stereoselectivity, offering a distinct advantage in the preparation of enantiopure products.[7-10] Over the past decades, the chemoenzymatic DKR has evolved into a powerful platform for the synthesis of a wide range of optically pure pharmaceuticals and fine chemicals, with research efforts largely focused on the development of robust racemization systems in combination with highly selective resolving enzymes.[11-12] However, the fundamental challenge of a conventional chemoenzymatic DKR process lies in synchronizing the two catalytic events of racemization and resolution in accordance with the core principles:[13-17] (i) the KR must display a sufficient enantioselectivity (E value = $k_{\text{fast}}/\text{k}_{\text{slow}} \geq 20$), and the rate of racemization (k_{rac}) must be at least 10 times faster than the enzyme-catalyzed reaction of the slow reacting enantiomer (k_{slow}) (first limitation), and (ii) the enzyme and the racemization catalyst must be compatible with one another, and the racemization

catalyst must not react with the product formed from the resolution) (second limitation). These stringent requirements substantially narrow the scope of applicable systems. The challenge becomes even more pronounced in chemoenzymatic DKR, where additional factors, such as reaction parameters and the nature of the racemization catalyst, can inadvertently cause enzyme deactivation.[13-17] Therefore, the development of innovative strategies that simultaneously overcome the primary limitation requiring racemization to outpace resolution and address the secondary limitation of chemoenzymatic incompatibility is urgently needed."

<u>To:</u> "Kinetic resolution (KR) is intrinsically limited to a theoretical maximum yield of 50%, whereas dynamic kinetic resolution (DKR) enables complete (theoretical 100%) conversion of racemic substrates into a single enantiomer, as shown in Figure 1.[1-2] Over the past decades, the chemoenzymatic DKR has evolved into a powerful platform for the synthesis of a wide range of optically pure pharmaceuticals and fine chemicals, which not only overcomes the intrinsic yield limitation of enzymatic KR but also preserves the enzyme's exquisite stereoselectivity.[3-8] However, the fundamental challenge of a conventional chemoenzymatic DKR process lies in synchronizing the two catalytic events of racemization and resolution in accordance with the core principles (Figure 1A):[9-12] (i) the KR must display a sufficient enantioselectivity (E value = $k_{\text{fast}}/k_{\text{slow}} \geq 20$), and the rate of racemization (k_{rac}) must be at least 10 times faster than the enzyme-catalyzed reaction of the slow reacting enantiomer (k_{slow}) (first limitation), and (ii) the enzyme and the racemization catalyst must be compatible with one another, and the racemization catalyst must not react with the product formed from the resolution (second limitation). The challenge becomes even more pronounced in chemoenzymatic DKR, where additional factors, such as reaction parameters and the nature of the racemization catalyst, can inadvertently cause enzyme deactivation.[13-17] Therefore, the development of innovative strategies that simultaneously overcome the primary limitation of racemization and resolution rates and address the secondary limitation of chemoenzymatic incompatibility is urgently needed."

<u>Revise Introduction (page 3, line 1):</u> "Chiral phthalides constitute a class of bioactive structural motifs broadly distributed in natural products, holding substantial promise for applications in natural product synthesis and medicinal chemistry.[18-19] Recent advances in their asymmetric synthesis have predominantly focused on purely chemical DKR strategies based on core principles,[20-25] in which racemization and resolution proceed in a cooperative manner within the conventional DKR systems. Considerable efforts have been devoted to elucidating and optimizing the racemization step to enhance both the reactivity and enantioselectivity of DKR processes. Representative examples reported by Smith and co-workers employed an isothiourea catalyst to selectively stabilize the transition state of the (*R*)-isomer *via* O···S chalcogen bonding,

thereby lowering its activation barrier and enabling racemization of the (*S*)-isomer within a DKR framework.[20] Similarly, a chiral 4-aryl-pyridine-N-oxide organocatalyst was shown to lower the barrier of the favorable (*S*)-isomer through a hydrogen-bonding network and enable the racemization of the unreacted (*R*)-isomer.[21] In contrast, Zhang's bicyclic imidazole-based catalyst achieved DKR through conformational control of the transition state *via* CH–π interactions at exceptionally low temperatures (–80 °C), effectively suppressing non-selective racemization.[22] By comparison, carbene-modulated DKR processes predominantly exploit steric effects to create a chiral environment. In such systems, the substrate forms an unstable hemiacetal intermediate with the *N*-heterocyclic carbene (NHC) organic catalysts, and the resulting energy difference between matched and mismatched isomers promotes efficient racemization of the unfavored enantiomer, ultimately delivering high DKR efficiency.[23-24] Recently, a chemoenzymatic DKR approach has been explored for the synthesis of chiral phthalides, where extensive lipase screening afforded only a relatively narrow substrate scope.[25] Notably, the strict adherence to the DKR principles inherently requires extensive and often laborious optimization of reaction conditions to finely control the sensitive racemization process, thereby severely limiting its practical applicability. Therefore, establishing a general strategy that decouples the cooperative racemization-resolution in conventional DKR into two independent stages may offer a powerful means to overcome the limitations of conventional DKR."

To: "Chiral phthalides, a class of bioactive structural motifs widely distributed in natural products, have attracted extensive interest,[18-19] and recent advances in their asymmetric synthesis have largely centered on purely chemical DKR strategies.[20–25] These studies primarily focus on elucidating and optimizing the cooperative interactions between racemization and resolution steps in order to enhance both the reactivity and enantioselectivity of DKR processes in accordance with core principles. For example, Smith and co-workers employed an isothiourea catalyst to selectively stabilize the transition state of the (*R*)-isomer *via* O···S chalcogen bonding, thereby lowering its activation barrier and enabling racemization of the (*S*)-isomer for an efficient DKR process.[20] In contrast, Zhang's bicyclic imidazole-based catalyst achieved efficient DKR through conformational control of the transition state *via* CH–π interactions at exceptionally low temperatures, effectively suppressing non-selective racemization.[22] Despite these successful examples, achieving an effective DKR process still requires extensive and often laborious optimization to finely control the sensitive racemization step. Therefore, establishing a general strategy that decouples the cooperative racemization-resolution into two independent stages offers a new possibility to overcome the limitations of conventional DKR."

Revise Introduction (page 4, line 10): "Polydimethylsiloxane (PDMS) membranes, distinguished by their unique hydrophobicity, chemical stability, and tunable permeability, serve as effective physical barriers and have been widely employed in diverse catalytic transformations.[26-28] The pioneering studies by the Gröger group and others have demonstrated the remarkable utility of PDMS membranes as thimbles in diverse chemoenzymatic two-step sequential enantioselective processes.[29-38] In their approach, a PDMS thimble mediates the diffusion of organic intermediates from an oxidation zone to a reduction zone, enabling a Wacker oxidation/enzymatic reduction cascade.[29] While effective, this strategy still requires removing the thimble after the initial oxidation to prevent catalyst incompatibility. As noted by the authors, failure to remove the catalyst inevitably leads to enzyme deactivation, severely compromising catalytic efficiency.[29-31] Building on these insights, and inspired by the elegant work of the Hérault and Bressy groups on physical separation of enantiomeric products,[39] in this context, we introduce a membrane-modulated DKR strategy that potentially bypasses the fundamental constraints of conventional DKR. In our design, by decoupling the cooperative racemization–resolution process into two independent stages (blue box versus brown-red box in the strategy of Figure 1B), the strategy used in a PDMS-modulated process fulfills three collaborative roles to overcome the intrinsic challenges of mandatory rate matching and complex interactions between racemization and resolution of conventional DKR: (i) physical shielding, (ii) selective permeability, and (iii) independent compartmentalization. The shielding function prevents direct contact between racemization chemocatalysts and resolution biocatalysts, thereby eliminating the incompatibility that constitutes the second principal limitation of conventional DKR systems. Remarkably, even if a racemization chemocatalyst partially interferes with the resolution step in a conventional DKR setting, an efficient DKR process is still achieved through this membrane-modulated strategy, substantially broadening the scope of usable racemization chemocatalysts. Beyond shielding, the selective permeability of the PDMS membrane leverages its dynamic mass-transfer capability, permitting the diffusion of targeted intermediate ((±)-**2a**), while confining catalysts, substrates, and other components to their respective compartments. This feature eliminates the strict necessity of laborious rate-ratio tuning, as the continuous consumption of intermediates inherently biases the reversible equilibria toward product formation. Notably, an efficient DKR process can be realized with only a single effective racemization step, thereby dramatically simplifying the most time-intensive aspect of DKR optimization. The most transformative feature is the independent compartmentalization afforded by the membrane, which establishes two isolated yet kinetically coupled environments. This spatial segregation maintains a continuous concentration gradient of intermediates between the two sides of the membrane, which drives the cascade forward without the need for kinetic

synchronization, thereby overcoming the first principal limitation of DKR systems. Evidently, as long as an appropriate concentration gradient is maintained, an efficient DKR becomes achievable without rate matching, representing a conceptual advance that removes the constraints of conventional DKR and unlocks a far broader spectrum of racemization–resolution pairings than previously possible. As presented in Figure 1C, by utilizing the dynamic tautomeric equilibrium between **1a** and **2a,** a practical PDMS-modulated chemoenzymatic DKR process as a representative solution is established, in which two independent compartments (**A** and **B**) are created, separated by a PDMS membrane. Compartment-**A** accommodates a base (e.g., DBU: 1,8-diazabicyclo[5.4.0]undec-7-ene) serving as a racemization chemocatalyst,[40-42] while Compartment-**B** houses a lipase functioning as a resolution biocatalyst (e.g., CALB, Novozym-435).[43-47] This practical PDMS-modulated chemoenzymatic DKR process ensures complete spatial separation of DBU and CALB while orchestrating the collaborative interplay between DBU-mediated racemization and CALB-mediated resolution. As envisioned, this membrane-modulated DKR strategy furnishes a powerful methodology that grants access to otherwise inaccessible enantioenriched phthalide derivatives, reducing the practical requirement to that of a simple kinetic resolution and thereby achieving possible complete (theoretical 100%) conversion of racemic substrates into a single enantiomer."

To: "Polydimethylsiloxane (PDMS) membranes, as effective physical barriers, have been widely employed in diverse catalytic transformations.[26-28] The pioneering studies by the Gröger group and others have demonstrated the remarkable utility of PDMS membranes as thimbles in diverse chemoenzymatic two-step sequential enantioselective processes.[29-37] Although the method is effective, the membrane-mediated DKR process still faces the issue of reduced chiral product conversion due to membrane diffusion limitations. Inspired by the advantage of the continuous-flow DKR approach in achieving quantitative chiral conversion,[38] and combining the feature of physical separation of enantiomeric products in a membrane-mediated KR process,[39] a membrane-modulated DKR strategy is expected to decouple racemization and resolution into two independent stages, thereby overcoming the limitations of conventional batch DKR processes. In our design, by decoupling the cooperative racemization–resolution process into two independent stages (blue box versus brown-red box in the strategy of Figure 1B), the strategy used in a PDMS-modulated process fulfills three collaborative roles to overcome the intrinsic challenges of mandatory rate matching and complex interactions between racemization and resolution of conventional DKR: (i) physical shielding, (ii) selective permeability, and (iii) independent compartmentalization. The shielding function prevents direct contact between racemization chemocatalysts and resolution biocatalysts, thereby eliminating

the incompatibility that constitutes the second principal limitation of conventional DKR systems. The selective permeability of the PDMS membrane leverages its dynamic mass-transfer capability, permitting the diffusion of targeted intermediate ((±)-**2a**), while confining catalysts, substrates, and other components to their respective compartments, thereby simplifying the most time-intensive aspect of DKR optimization. The independent compartmentalization establishes two isolated environments and maintains a continuous concentration gradient of intermediates between the two sides of the membrane, thereby driving the cascade forward and overcoming the first principal limitation of DKR systems. As presented in Figure 1C, by utilizing the dynamic tautomeric equilibrium between **1** and **2,** a practical PDMS-modulated chemoenzymatic DKR process as a representative solution is established. Compartment-**A** accommodates a base (e.g., DBU: 1,8-diazabicyclo[5.4.0]undec-7-ene) serving as a racemization chemocatalyst,[40-42] while Compartment-**B** houses a lipase functioning as a resolution biocatalyst (e.g., CALB, Novozym-435).[43-47] This DKR process ensures complete spatial separation of DBU and CALB while orchestrating the collaborative interplay between DBU-mediated racemization and CALB-mediated resolution. As envisioned, this membrane-modulated DKR strategy furnishes a powerful methodology that grants access to otherwise inaccessible enantioenriched phthalide derivatives, reducing the practical requirement to that of a simple kinetic resolution and thereby achieving possible complete conversion of racemic substrates into a single enantiomer."

Regarding the question about "The authors should also mention flow-chemistry applications of DKR, and a flow-reactor methodology can in principle deliver 99% conversion of the product in optically pure form, the batch solution with a membrane slows down significantly around 90% conversion due to diffusional limitations (i.e., the kinetics of diffusion at low concentration of remaining substrate becomes severely rate-limiting)", we have also added the advantage of the continuous-flow DKR approach and provided a comparative analysis of the limitations of the membrane-mediated approach as follows.

Revise (page 4, line 10): "The pioneering studies by the Gröger group and others have demonstrated the remarkable utility of PDMS membranes as thimbles in diverse chemoenzymatic two-step sequential enantioselective processes.[29-37]"

To: "The pioneering studies by the Gröger group and others have demonstrated the remarkable utility of PDMS membranes as thimbles in diverse chemoenzymatic two-step sequential enantioselective processes.[29-37] Although the method is effective, the membrane-mediated DKR process still faces the issue of reduced chiral product conversion due to membrane diffusion limitations (i.e., the kinetics of diffusion at low concentrations of the remaining substrate become severely rate-limiting). Inspired by

the advantage of the continuous-flow DKR approach in achieving quantitative chiral conversion,[38] and combining the feature of physical separation of enantiomeric products in a membrane-mediated KR process,[39] a membrane-modulated DKR strategy is expected to decouple racemization and resolution into two independent stages, thereby overcoming the limitations of conventional batch DKR processes.

In addition, the relevant literature has also been cited accordingly as ref. 38, as follows.

Add ref. 38: "38. de Miranda, A. S., de M. Silva, M. V., Dias, F. C., de Souza, S. P., Leão, R. A. C. & de Souza, R. O. M. A. Continuous flow dynamic kinetic resolution of *rac*-1-phenylethanol using a single packed-bed containing immobilized CAL-B lipase and $VOSO_4$ as racemization catalysts, *React. Chem. Eng.* **2**, 375-381 (2017)."

Question 2:

The text is also redundant in the Results and Discussion. There are three main key points: 1) DBU influences the catalytic activity and, maybe, the enantioselectivity of CALB; 2) DBU can act as a catalyst for the esterification itself with no selectivity; 3) points 1 and 2 impede the classical DKR, and the only way to do it is through compartmentalization of the two catalysts. These are three simple concepts to convey, yet the authors use very long text, repeating the same things several times. The text is redundant and rather pompous. A more concise and scientifically sober narrative would be advisable.

Response: According to the reviewer's suggestion, we have replaced those inappropriately long sentences with more concise statements. Please see the revised manuscript as follows.

Revise (page 9, line 5): "In the first type of evaluation, the traditional single-step reaction performed without membrane assistance revealed that, despite extensive optimization, increasing the molar amount of DBU consistently improved the reaction yield but invariably led to a pronounced erosion of enantioselectivity (Scheme 1A). This observation indicated that, beyond catalyzing racemization through the ring-opening/closing equilibrium, DBU might also act as a base catalyst to promote esterification. Supporting evidence was provided by a control experiment, in which omission of CALB and addition of two equivalents of DBU relative to the substrate furnished **3a** in 46% yield but without enantioselectivity. This comparison demonstrated the additional roles of DBU in the DKR process. One pathway led to the conformational deactivation of CALB during the DKR process,[48-50] while the other facilitated a base-mediated esterification process,[40] with both factors contributing to the inherent incompatibility of this traditional DKR system. Notably, in contrast to the 91% yield and 99% *ee* obtained in the PDMS-modulated chemoenzymatic DKR process (entry 8, Table 1), this result conversely confirmed the superiority of the

membrane's separation and selective permeation functions. The key advantage of the PDMS-modulated strategy lies in its capacity to effectively decouple the racemization and chiral resolution stages, thereby enabling these intrinsically incompatible steps to proceed independently."

To: "In the first type of evaluation, the traditional single-step reaction performed without membrane assistance revealed that, despite extensive optimization, increasing the molar amount of DBU consistently improved the reaction yield but invariably led to a pronounced erosion of enantioselectivity (Scheme 1A). This observation indicates that: 1) DBU influences the catalytic activity and potentially the enantioselectivity of CALB;[48-50] 2) DBU itself can function as a non-selective catalyst for the esterification reaction.[40] Supporting evidence was provided by a control experiment, in which omission of CALB and addition of two equivalents of DBU relative to the substrate furnished **3a** in 46% yield but without enantioselectivity. Furthermore, compared to the 91% yield and 99% *ee* achieved by the designed PDMS-modulated DKR process (Entry 8, Table 1), these experimental data conversely confirmed that the efficient DKR could only be attained through spatial compartmentalization of the two catalysts, thereby demonstrating the advantage of the PDMS-modulated strategy."

Question 3:

Page 2: the authors state: "However, the fundamental challenge of a conventional DKR process lies in synchronizing the two catalytic events of racemization and resolution in accordance with the core principles: (i) the racemization rate must exceed the resolution rate to ensure the selective and rapid transformation of the favored enantiomer (first limitation)." This sentence is not conceptually correct in relation to DKR, in general. The racemization rate must be significantly higher than the rate for the conversion of the "wrong" (undesired) enantiomer by the enzyme. However, the racemization rate can be (and normally is) lower than the rate for the conversion of the desired enantiomer. Citing from Verho and Bäckvall, J. Am. Chem. Soc. 2015, 137, 3996−4009: "However, the design of a successful DKR system is far from simple, given that the following requirements must be fulfilled: (i) the KR must display a sufficient enantioselectivity (E value = $k_{fast}/k_{slow} \geq 20$); (ii) the enzyme and the racemization catalyst must be compatible with one another; (iii) the rate of racemization (krac) must be at least 10 times faster than the enzyme-catalyzed reaction of the slow-reacting enantiomer (kslow); and (iv) the racemization catalyst must not react with the product formed from the resolution." In conclusion, there is no mention of k_{fast}, strictly speaking. I think what the authors mean is that, in their specific case, it is necessary that the racemization is faster than k_{fast} because the racemization catalyst (DBU) can also catalyze the esterification but in a non-enantioselective manner, thus eroding the ee. In conclusion, the statement in the manuscript should be modified accordingly.

Response: Thank you very much for your valuable comments, which have helped us further improve the quality. According to the reviewer's suggestion, we have revised the statement based on the report by Verho and Bäckvall (*J. Am. Chem. Soc.* **2015**, 137, 3996–4009), and integrated the reported (i) and (iii) into our presented (i), and (ii) and (iv) into our presented (ii) to maintain the continuity of the manuscript as follows. Additionally, the references have been cited (Please see the answer to Question 3 for the above reviewer 1).

Revise (page 2, line 7): "However, the fundamental challenge of a conventional DKR process lies in synchronizing the two catalytic events of racemization and resolution in accordance with the core principles: (i) the racemization rate must exceed the resolution rate to ensure the selective and rapid transformation of the favored enantiomer (first limitation), and (ii) the racemization catalyst must function without interfering with the resolution process or reacting with the chiral product(s) (second limitation)."

To: "However, the fundamental challenge of a conventional DKR process lies in synchronizing the two catalytic events of racemization and resolution in accordance with the core principles (Figure 1A):[9-12] (i) the KR must display a sufficient enantioselectivity (E value = $k_{fast}/k_{slow} \geq 20$), and the rate of racemization (k_{rac}) must be at least 10 times faster than the enzyme-catalyzed reaction of the slow reacting enantiomer (k_{slow}) (first limitation), and (ii) the enzyme and the racemization catalyst must be compatible with one another, and the racemization catalyst must not react with the product formed from the resolution) (second limitation)."

Question 4:

- Page 4: "In such systems, the substrate forms an unstable hemiacetal intermediate with the NHC catalyst, and the resulting energy difference between matched and mismatched isomers promotes efficient racemization of the unfavored enantiomer, ultimately delivering high DKR efficiency". NHC has never been introduced so far as acronym.

Response: According to the suggestion, we have shortened the introduction by removing the statement in this section. Please see the response to Question 1 above.

Question 5

Page 6: "Beyond shielding, the selective permeability of the PDMS membrane leverages its dynamic mass-transfer capability, permitting the diffusion of targeted intermediate (3-hydroxyisobenzofuranone), while confining catalysts, substrates, and other components to their respective compartments". It would be beneficial for the paper and the readers to add to Figure 1 letters for each molecule and then add it in the

text, 3-hydroxyisobenzofuranone (xxx).

<u>Response</u>: According to the reviewer's suggestion, we have added labels to each molecule in Figure 1 and referenced the Figure in the text when discussing 3-hydroxyisobenzofuran-1-one.

<u>Revise (page 5, line 10):</u> "The selective permeability of the PDMS membrane leverages its dynamic mass-transfer capability, permitting <u>the diffusion of targeted intermediate (3-hydroxyisobenzofuranone)</u>, while confining catalysts, substrates, and other components to their respective compartments."

<u>To:</u> "The selective permeability of the PDMS membrane leverages its dynamic mass-transfer capability, permitting t<u>he diffusion of targeted intermediate ((±)-**2a**)</u>, while confining catalysts, substrates, and other components to their respective compartments."

<u>Additionally, Figure 1 has been modified as follows.</u>

<u>Revise (page 3, Figure 1):</u>

"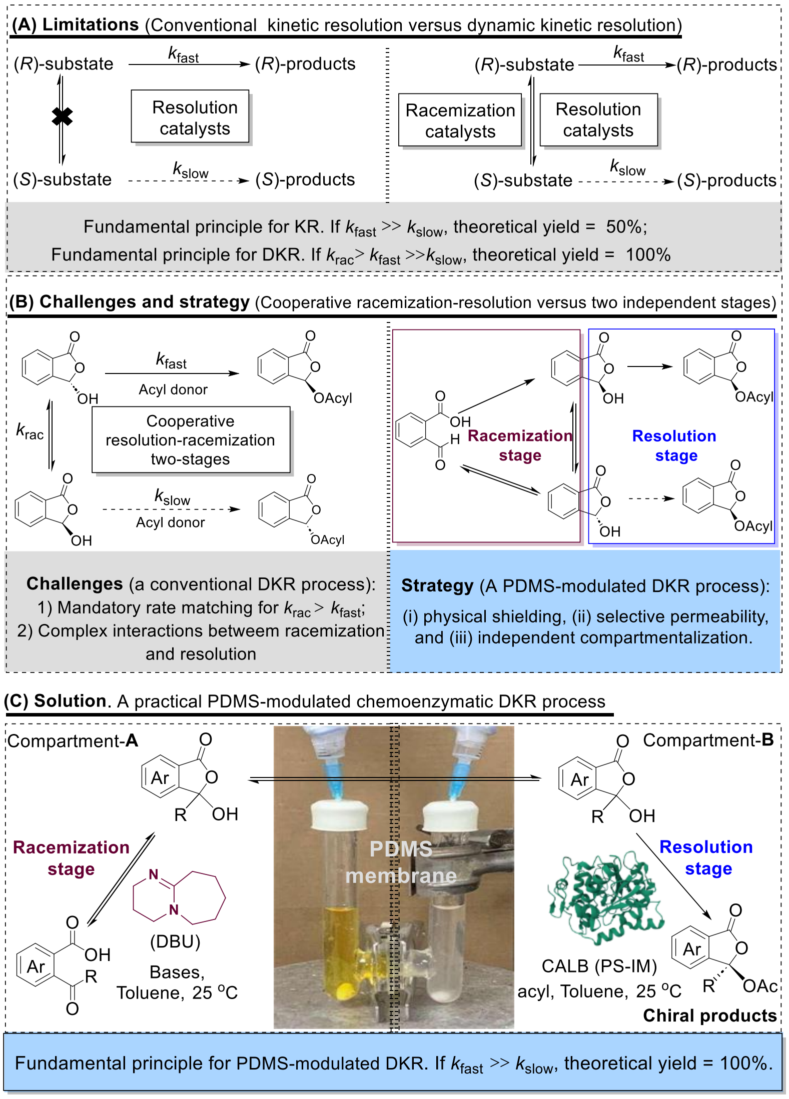

"

<u>To:</u>

“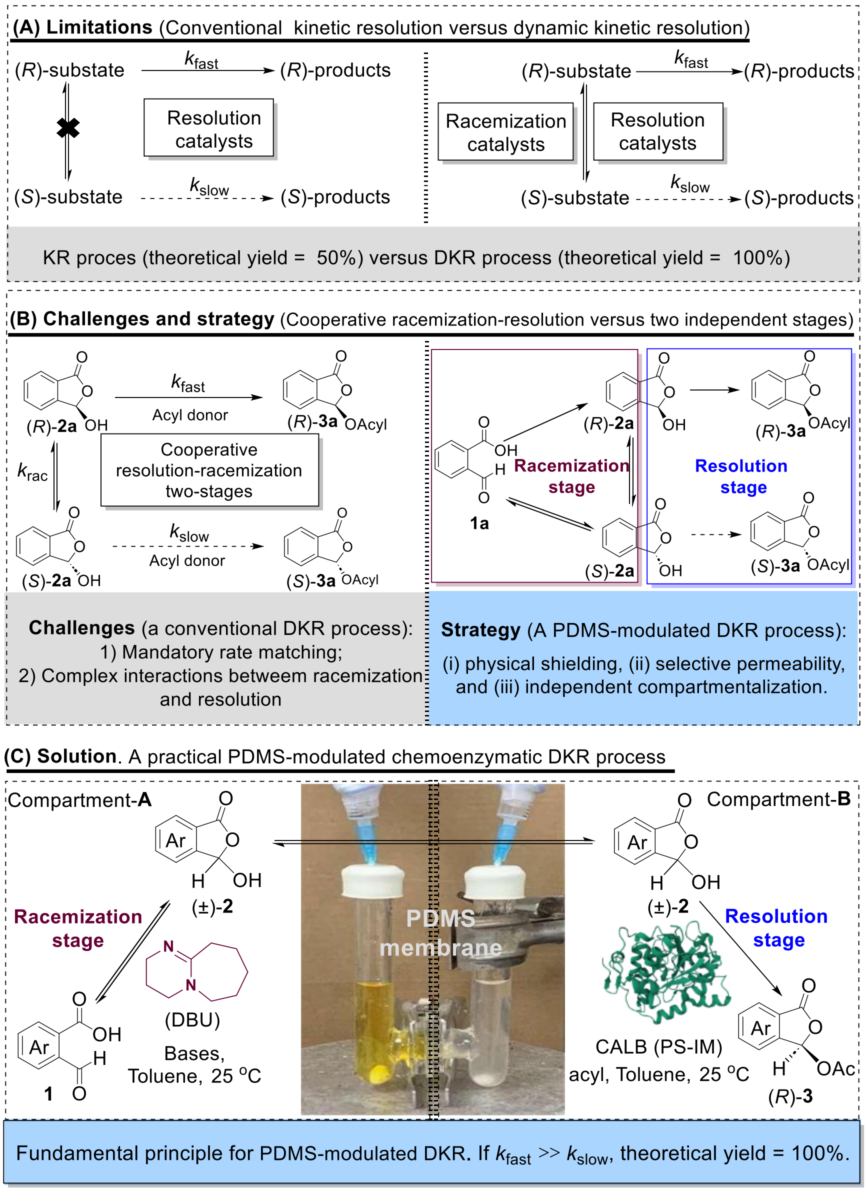

”

Question 6

Page 7: “As presented in Figure 1C, by utilizing the dynamic tautomeric equilibrium between 1a and 2a, a practical PDMS-modulated chemoenzymatic DKR process as a representative ...” In Figure 1, the authors forgot to insert the numbers in the molecules for 1a and 2a, present for example in Table 1. Please add it to the table to render the comprehension easier.

Response: According to the reviewer’s suggestion, we have added labels to each molecule in Figure 1. Please see our response to Question 5 above. Also, the statement has been revised as follows.

Revise (page 5, line 11): “As presented in Figure 1C, by utilizing the dynamic tautomeric equilibrium between **1a** and **2a**, a practical PDMS-modulated chemoenzymatic DKR process as a representative solution is established.”

To: "As presented in Figure 1C, by utilizing the dynamic tautomeric equilibrium between **1** and **2**, a practical PDMS-modulated chemoenzymatic DKR process as a representative solution is established."

Question 7

Page 9: "Notably, these comparisons revealed that the second-step biocatalysis was severely suppressed in the presence of ..." I guess the authors wanted to write the second biocatalytic step instead of second-step biocatalysis. Please rewrite to make it clear.

Explanation: According to the reviewer's suggestion, we have rewritten this sentence to improve its clarity. Please see the revised manuscript as follows.

Revise (page 8, line 1): "Notably, these comparisons revealed that the second-step biocatalysis was severely suppressed in the presence of bases due to a deactivation effect of bases impacting CALB, thereby disclosing the incompatible nature of the conventional DKR process that originated from the mismatch between base-mediated racemization and CALB-mediated resolution."

To: "Notably, these comparisons revealed that the conventional two-step cascade DKR process was severely suppressed in the presence of bases due to a deactivation effect of bases impacting CALB, thereby disclosing the incompatible nature of the conventional DKR process that originated from the mismatch between base-mediated racemization and CALB-mediated resolution."

Question 8

Page 9: "Finally, in the evaluation of an integrated PDMS-modulated DKR (II) based on the optimizations (see Supplementary Experimental and Table S2, SI), it was observed that the model reaction could steadily provide the final (R)-3a with a 91% yield and 99% ee (entry 8), which was slightly superior to the outcomes obtained with other DBU loadings (entry 8 versus entries 9 and 10)". I don't think the results obtained in entries 8, 9 and 10 are statistically different. How would you explain this different outcome? From my perspective, it is just a normal experimental fluctuation of data. Statistical analysis from more data of same type of experiments would be needed to prove that.

Response: Yes, we agree with the reviewer's comment. According to the reviewer's suggestion, we repeated the experiment with tightly controlled reaction time. The results showed that the yield of (*R*)-**3a** with 1.2 or 0.8 equivalents of DBU was consistent with our initial findings, which likely falls within the range of normal experimental variation, as the reviewer mentioned. However, a yield drop from 89% to 81% was observed when using 0.5 equivalents of DBU instead of 0.8 equivalents, whereas the yield decreased from 90% to 85% when 1.5 equivalents were used in place

of 1.2 equivalents. This indicates that the effect is only noticeable when comparing a wider range of loadings. As we expected, an excess of DBU leads to the excessive ring-opening of the cyclization intermediate for the racemization, thereby compromising the final yield. Conversely, an insufficient amount of DBU is unable to promote an effective cyclization process for the racemization, also resulting in yield reduction. Therefore, we have replaced the data for 1.2 and 0.8 equivalents of DBU in Table 1 with the data for 1.5 and 0.5 equivalents of DBU, respectively, thereby elucidating the rationale for the optimization based on variations in DBU catalyst loading. Please see the revised Table 1 as follows.

Revise (Table 1, page 7):

"**Table 1.** Comparative reaction study.[a]

Entry	Type	Catalyst(s)/solvent(s)	%Yield of (*R*)-**3a**[b]	%*ee*) of (*R*)-**3a**[b]
1	**I**	CALB/toluene	48	99
8	**II**	DBU/toluene + CALB/toluene	91	99
9 [c]	**II**	DBU/toluene + CALB/toluene	90	99
10 [d]	**II**	DBU/toluene + CALB/toluene	89	99
12 [f]	**II**	DBU/toluene + CALB/toluene	87	99

[a] Reaction conditions: For reaction (**I**), **1a** (0.20 mmol), base (0.20 mmol), and/or additive in 2.0 mL of solvent (s), 25 °C, 4 h. For reaction (**II**), Reaction conditions. **2a** (0.20 mmol), Novozym-435 (CALB) (40.0 mg, 20 mg/0.1 mmol), and acetic anhydride (0.40 mmol, 2.0 equiv.) in 2.0 mL of toluene, 25 °C, 36 h. For reaction (**III**) by using a 0.40 mm-thick PDMS membrane; In Compartment-**A**, **1a** (0.20 mmol), base (0.20 mmol), and/or additive in 2.0 mL of solvent (s), 25 °C, 4 h. In compartment-**B**, Novozym-435 (CALB) (40.0 mg, 20 mg/0.1 mmol), and acetic anhydride (0.40 mmol, 2.0 equiv.) in 2.0 mL of toluene, 25 °C, 36 h. [b] Yields were determined by ^{1}H–NMR analysis, and the %*ee* values were determined by chiral HPLC analysis. [c] Data were obtained using 1.20 equivalents of DBU in Compartment-**A**. [d] Data were obtained using 0.80 equivalents of DBU in Compartment-**A.** [e] Data were obtained using a 0.20 mm thick PDMS membrane. [f] Data were obtained using a 0.60 mm thick PDMS membrane."

To:

"**Table 1.** Comparative reaction study.[a]

Entry	Type	Catalyst(s)/solvent(s)	%Yield of (*R*)-**3a**[b]	%*ee*) of (*R*)-**3a**[b]
1	**I**	CALB/toluene	48	99
8	**II**	DBU/toluene + CALB/toluene	91	99
10 [d]	**II**	DBU/toluene + CALB/toluene	85	99
11 [e]	**II**	DBU/toluene + CALB/toluene	81	99
12 [f]	**II**	DBU/toluene + CALB/toluene	87	99

[a] Reaction conditions: For reaction (**I**), **1a** (0.20 mmol), base (0.20 mmol), and/or additive in 2.0 mL of solvent (s), 25 °C, 4 h. For reaction (**II**), Reaction conditions. **2a**

(0.20 mmol), Novozym-435 (CALB) (40.0 mg, 20 mg/0.1 mmol), and acetic anhydride (0.40 mmol, 2.0 equiv.) in 2.0 mL of toluene, 25 °C, 36 h. For reaction (**III**) by using a 0.40 mm-thick PDMS membrane; In Compartment-**A**, **1a** (0.20 mmol), base (0.20 mmol), and/or additive in 2.0 mL of solvent (s), 25 °C, 4 h. In compartment-**B**, Novozym-435 (CALB) (40.0 mg, 20 mg/0.1 mmol), and acetic anhydride (0.40 mmol, 2.0 equiv.) in 2.0 mL of toluene, 25 °C, 36 h. [b] Yields were determined by ^{1}H–NMR analysis, and the %*ee* values were determined by chiral HPLC analysis. [c] Data were obtained using 1.0 equivalents of NEt_3 instead of DBU in Compartment-**A** and PS-IM (PS-IM: lipases from *Burkholderia* (formerly Pseudomonas) *cepacia*) instead of CALB in Compartment-**B**. [d] Data were obtained using 1.50 equivalents of DBU in Compartment-**A**. [e] Data were obtained using 0.50 equivalents of DBU in Compartment-**A.** [f] Data were obtained using a 0.20 mm thick PDMS membrane. [g] Data were obtained using a 0.60 mm thick PDMS membrane.**”**

Question 9

Page 10: “In addition, it was also found that the membrane thickness had a certain impact on the PDMS-modulated DKR process, in which the reaction using a 0.40 mm-thick membrane yielded the best results compared to the reactions with other membrane thicknesses (entry 8 versus entries 11 and 12)”. How would the authors explain this phenomenon?

Response: In membrane-mediated catalytic reactions with the membrane size fixed, the membrane thickness influences the reaction outcome. The primary reason is the transmembrane process of intermediates. A thinner membrane accelerates this process, whereas a thicker one hinders it and may even lead to pore blockage. Our experimental results under strictly controlled timing confirmed this: when using a 0.20 mm thick PDMS membrane, although the transmembrane rate increased, a small amount of the chiral product permeated into Compartment-**A**, leading to a certain degree of reduction in yield. Conversely, a relatively thick membrane with 0.60 mm PDMS membrane caused a certain degree of hindrance, thereby reducing the efficiency of intermediate transport and consequently decreasing the yield after transmembrane passage.

Question 10

Page 12: again the same mistake in my opinion: “To elucidate the unique advantage of independent compartmentalization in circumventing the conventional requirement that the racemization rate must exceed the resolution rate (i.e., krac > kfast to ensure continuous racemization of the unreacted enantiomer).” This is not correct: krac >> kslow, but krac can also be equal to or smaller than kfast in a conventional DKR. It is the specific case of this DKR using DBU as the racemization catalyst that creates this additional requirement. It should be explained clearly.

Response: According to the reviewer’s suggestion, we have revised this statement

following the core principles in a conventional chemoenzymatic DKR process, as the reviewer mentioned (Please see the response to Question 3 above), as follows.

Revise (page 10, line 5): **"**To elucidate the unique advantage of independent compartmentalization in circumventing the conventional requirement that the racemization rate must exceed the resolution rate (i.e., $k_{rac} > k_{fast}$ to ensure continuous racemization of the unreacted enantiomer), a series of bases with intrinsically low racemization activities were subsequently evaluated, as shown in Scheme 1C.**"**

To: **"**To elucidate the advantage of independent compartmentalization in a PDMS-modulated DKR process, a series of bases with intrinsically low racemization activities were subsequently evaluated, as shown in Scheme 1C.**"**

Question 11

Page 13: "Thus, the combination of this membrane-mediated device with the dynamic tautomerization facilitated by even any racemization base catalyst in Compartment-A was sufficient to sustain an efficient DKR process, thereby establishing a new mechanistic paradigm in which a concentration gradient, rather than relative rate control, drives the process." I do not think that this is a new mechanistic paradigm. It is based on the law of diffusion, and racemization is based on the concept of entropy—very classical concepts from the last century and earlier that can be found in textbooks. Again, an exaggerated narrative, in my opinion.

Response: According to the reviewer's suggestion, we have corrected the statements as follows.

Revise (page 11, line 5): **"**Thus, the combination of this membrane-mediated device with the dynamic tautomerization facilitated by even any racemization base catalyst in Compartment-**A** was sufficient to sustain an efficient DKR process, thereby establishing a new mechanistic paradigm in which a concentration gradient, rather than relative rate control, drives the process.**"**

To: **"**Thus, the combination of this membrane-mediated device with the dynamic tautomerization facilitated by even any racemization base catalyst in Compartment-**A** was sufficient to sustain an efficient DKR process, thereby developing a new approach in which a concentration gradient, rather than relative rate control, drives the process.**"**

Question 12

Page 15: "This equilibrium facilitates the transmembrane migration of racemic 2 while retaining 1 in Compartment-A". Why is 1 retained? Do you have proof of the fact that 1 doesn't pass through the membrane? Did you try to put 1 in compartment B?

Response: Substrates **1** are primarily retained because the membrane is hydrophobic and organic. The water-soluble substrate **1** has difficulty permeating the membrane and entering Compartment-**B** due to its high polarity and hydrophilicity.

Scheme. Control diffusion experiment (Conditions. In compartment-**A**, **1a** (0.20 mmol) and DBU (0.20 mmol) in 2.0 mL of toluene, 25 °C, 36 h. In compartment-**B**, 2.0 mL of toluene, 25 °C, 36 h.)

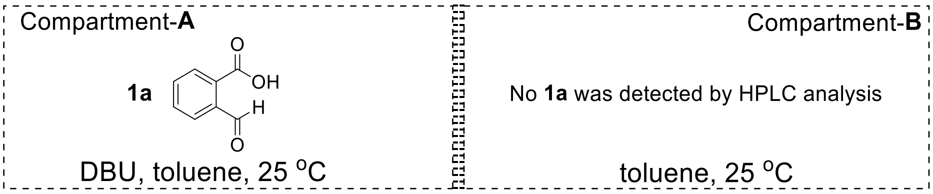

To demonstrate this claim that substrates do not pass through the membrane, we conducted control experiments where Compartment-**A** contained **1a** in the presence of DBU, while Compartment-**B** was kept blank, as illustrated in the above Scheme (Conditions. In compartment-**A**, **1a** (0.20 mmol) and DBU (0.20 mmol) in 2.0 mL of toluene, 25 °C, 36 h. In compartment-**B**, 2.0 mL of toluene, 25 °C, 36 h.). The results showed that **1a** was not detected, as confirmed by HPLC analysis, thereby confirming that **1a** in Compartment-**A** could not permeate through the membrane into Compartment-**B**.

In addition, according to the reviewer's suggestion, we also conducted a control experiment by placing **1a** in Compartment-**B** under the standard reaction conditions (Conditions: In Compartment-**A**, DBU (0.20 mmol) in 2.0 mL of toluene, 25 °C, 36 h. In Compartment-**B**, **1a** (0.20 mmol), Novozym-435 (CALB) (40.0 mg, 20 mg/0.1 mmol), and acetic anhydride (0.40 mmol, 2.0 equiv.) in 2.0 mL of toluene, 25 °C, 36 h.), and found that a 69% yield of the target product was obtained without any change in enantioselectivity (99% *ee*). A yield of 69% was still achieved, indicating the presence of a certain degree of dynamic tautomeric equilibrium between **1a** and **2a**, as we mentioned in the manuscript **(page 7, line 3**: Owing to the pronounced dynamic tautomeric equilibrium between 2-formylbenzoic acid (**1a**) and 3-hydroxyisobenzofuran-1(3H)-one (**2a**), with **2a** predominating through a ring-opening pathway under basic conditions). Notably, a yield still greater than the 48% obtained in the KR process for the target product (entry 1 in Table 1) demonstrates three phenomena. First, the presence of a certain degree of dynamic tautomeric equilibrium could overcome the interference of the base (DBU) in conventional KR processes, allowing the enantioselectivity to remain unaffected in Compartment-**B**. Second, the yield exceeding the 50% resolution limit of traditional KR processes indicates that the membrane enables the unconverted (*S*)-configured cyclic substrate **2a** to cross the membrane into Compartment-**A**, where it undergoes base-catalyzed racemization, thereby achieving a yield greater than the theoretical maximum of conventional KR processes. These comparisons illustrate that the advantage of the PDMS-modulated DKR process lies in its ability to decouple the cooperative racemization–resolution process into two independent stages. Third, a small portion of **1a** can directly react with

the anhydride, resulting in the formation of the corresponding acylated byproduct. This side reaction is responsible for the reduced yield compared to the standard membrane-mediated DKR process.

Question 13

Page 19: "Third, for the racemic tetra-substituted 3-hydroxyphthalides (**5a**–**5h**), which was difficult to access by conventional chemoenzymatic strategy, the membrane-modulated DKR also exhibited remarkable tolerance, consistently furnishing the challenging chiral tetra-substituted 3-hydroxyphthalide esters ((*R*)-**6a**–(*R*)-**6h**) in acceptable yields with excellent enantioselectivities (Equation 3, Scheme 3)." This limitation is attributable to CALB. Why does the DKR of tetra-substituted 3-hydroxyphthalides become easier with the membrane reactor if the catalytic rate depends only on the enzyme? Can the authors comment on that and provide an explanation?

Response: Yes, the improved feasibility of the DKR process for tetra-substituted 3-hydroxyphthalides was primarily attributed to the PDMS-modulated DKR strategy, which enabled the decoupling of the cooperative racemization–resolution process into two independent stages. This approach effectively prevents DBU from interfering with the activity and enantioselectivity of the CALB enzyme, as the two components are spatially segregated in separate compartments. The compartmentalization strategy ensures that the base-catalyzed racemization and enzyme-mediated resolution occur in isolated environments, eliminating their mutual deactivation as the base (DBU) can alter the chiral microenvironment of the enzyme (e.g., by disrupting or interfering with the folding of the chiral cavity, leading to reduced activity and/or diminished enantioselectivity).

In conventional one-pot reaction systems, it was well-known that the presence of DBU base could induce structural folding or conformational changes in CALB, leading to the loss of its catalytic activity and/or partial loss of enantioselective activity. This explained why racemic tetra-substituted 3-hydroxyphthalides exhibited poor yields and low *ee* values in traditional one-pot systems. As reported in the literature (ref. 24: *J. Org. Chem.* 2023, **88**, 3897–3902), the only reported example of a racemic tetra-substituted 3-hydroxyphthalide yielded the product (*R*)-**6a** with merely 79% yield and 61% *ee*. In contrast, the PDMS-based strategy spatially separated the racemization and resolution steps, ensuring that DBU did not compromise the CALB performance. As a result, the DKR process became more efficient and afforded product (*R*)-**6a** in 84% yield and 99% *ee*.

Question 14

Page 21, but also in other sections of the manuscript: "The independent

compartmentalization maintains kinetically coupled yet isolated environments, sustaining a substrate concentration gradient that drives DKR forward without kinetic synchronization." The use of the definition "kinetic synchronization" is not standard in DKR. The authors should better define what they mean by "kinetic synchronization." It would appear that "kinetic synchronization" is not possible in DKR with catalysts compartmentalization, while in contrast kinetic resolution and racemization must always run concurrently. In conclusion, either the authors can explain in a concrete way what this is about or such sentences must be removed from the manuscript.

Response: According to the reviewer's suggestion, we have removed the statements about "kinetic synchronization". The statement has been revised as follows.

Revise (page 21, line 4): **"**The independent compartmentalization maintains kinetically coupled yet isolated environments, sustaining a substrate concentration gradient that drives DKR forward without kinetic synchronization**"**

To: **"**The independent compartmentalization creates mutually isolated environments and maintains a concentration gradient of intermediates across the membrane, thereby enabling the DKR to proceed continuously and effectively.**"**

Question 15

Page S4: Why do you need a balloon filled with air? What happen if you close the system completely? Why did you filter the solution afterward? What is the solid part coming from? Only the enzyme?

Response: Yes, we used an air balloon during the catalytic process. An important reason is to maintain equilibrium on both sides of the membrane, so that the concentration difference acts as a key driving force rather than the pressure difference, thereby promoting the equilibrium of intermediates across the membrane and enabling an efficient racemization through the DKR process. Another reason is that local conditions, particularly significant fluctuations in humidity, lead to some degree of yield variation in repeated catalytic experiments. To overcome this error, we used an air-filled balloon to exclude the influence of such factors and ensure data reproducibility.

Actually, when an air balloon was not used during the catalytic process, the results remained the same under normal weather conditions. However, when the system was completely sealed, both the pressure difference and the concentration difference simultaneously affected the equilibrium across the membrane, influencing the transport of intermediates. This leads to a decrease in yield from 91% to 84% within 36 h, while the enantioselectivity remains unchanged. Filtration is a conventional post-treatment step aimed at separating a small amount of insoluble solids, which in this process primarily include the enzyme and a trace amount of insoluble acetate in toluene. The purpose of this routine post-treatment step is to facilitate the subsequent purification of the product through column chromatography.

<u>Question 16</u>

Page S27: the two HPLC chromatograms don’t testify the production of one of the enantiomer; the retention times are different between the racemic mixture and the enantioselective one (with only one peak).

<u>Response</u>: Yes, we have re-run the product sample by HPLC to correct this discrepancy. Now, the retention time of the reaction sample was consistent with that of the racemic mixture as follows. Please refer to the revised Supporting Information.

<u>Revise (SI page S27):</u>

“**(*R*)-3c: (*R*)-5-fluoro-3-oxo-1,3-dihydroisobenzofuran-1-yl acetate** (HPLC: Chiralpak IC, detected at 210 nm, eluent: n-hexane/2-propanol = 80/20, flow rate = 1.0 mL/min, 25°C).

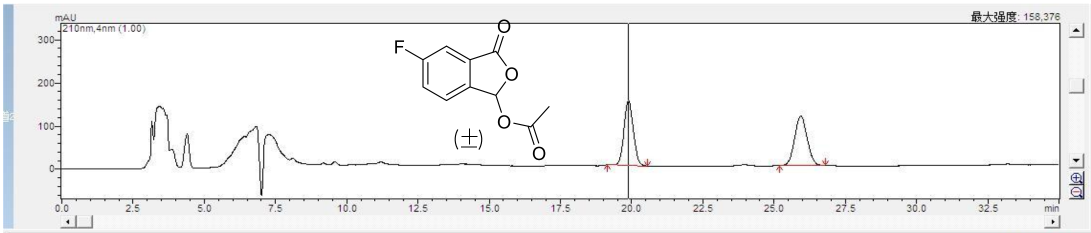

化合物表视图

ID#	名称	保留时间	峰#	面积	高度	面积%
1	RT19.882	19.882	1	3391177	149138	49.1634
2	RT25.928	25.928	2	3506597	114493	50.8366

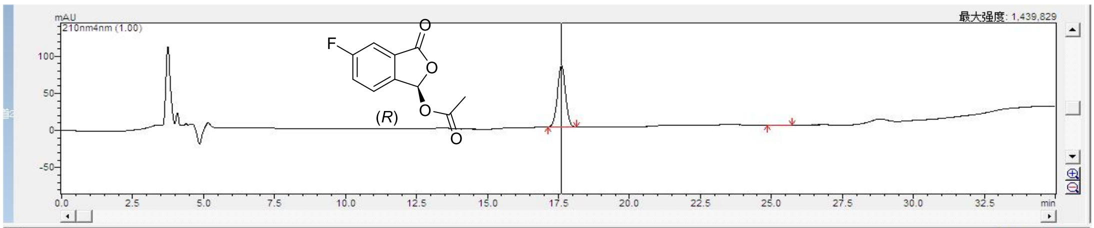

化合物表视图

ID#	名称	保留时间	峰#	面积	高度	面积%
1	RT17.601	17.601	1	1633003	82355	99.1933
2	RT25.159	25.159	2	13280	959	0.8067

”

<u>To:</u>

“**(*R*)-3c: (*R*)-5-fluoro-3-oxo-1,3-dihydroisobenzofuran-1-yl acetate** (HPLC: Chiralpak IC, detected at 210 nm, eluent: n-hexane/2-propanol = 80/20, flow rate = 1.0 mL/min, 25°C).

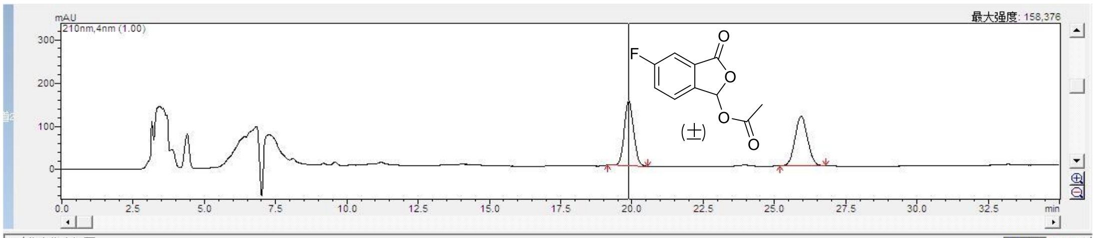

化合物表视图

ID#	名称	保留时间	峰#	面积	高度	面积%
1	RT19.882	19.882	1	3391177	149138	49.1634
2	RT25.928	25.928	2	3506597	114493	50.8366

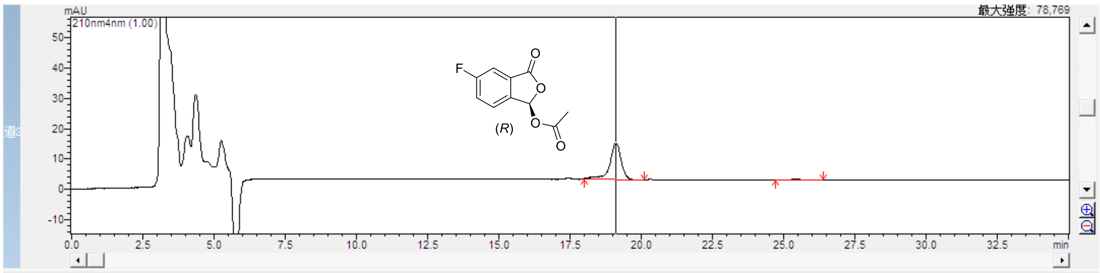

化合物表视图

ID#	名称	保留时间	峰#	面积	高度	面积%
1	RT19.104	19.104	1	322753	12025	99.5984
2	RT25.324	25.324	2	1301	253	0.4016

”

Question 17

Page S32: the two HPLC chromatograms don't testify the production of one of the enantiomer; the retention times are different between the racemic mixture and the enantioselective one (with only one peak).

Response: Yes, we have re-run the product sample by HPLC to correct this discrepancy by adjusting the eluent composition from n-hexane/2-propanol = 80/20 to n-hexane/2-propanol = 90/10. Now, the retention time of the reaction sample was consistent with that of the racemic mixture as follows. Please refer to the revised Supporting Information.

Revise (Figures on page S32):

"(*R*)-3h: (*R*)-4-bromo-3-oxo-1,3-dihydroisobenzofuran-1-yl acetate (HPLC: Chiralpak AD-H, detected at 210 nm, eluent: n-hexane/2-propanol = 80/20, flow rate = 1.0 mL/min, 25°C).

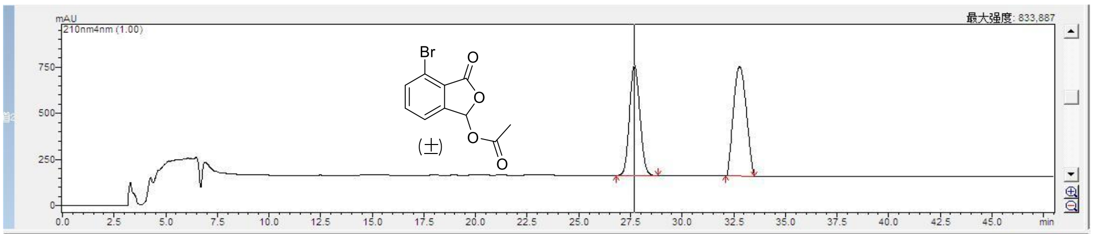

化合物表视图

ID#	名称	保留时间	峰#	面积	高度	面积%
1	RT27.687	27.687	1	20788157	594408	45.0827
2	RT32.782	32.782	2	25323021	590347	54.9173

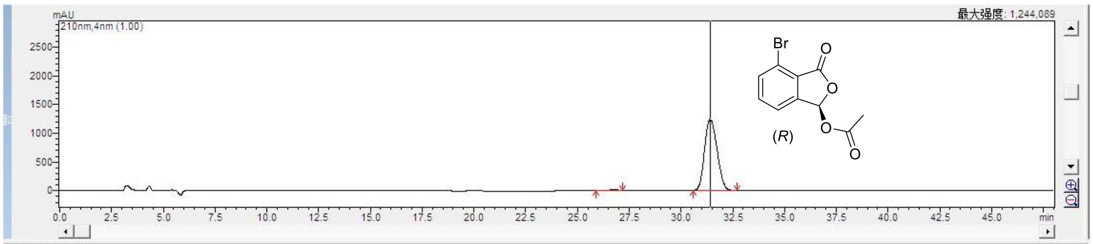

化合物表视图

ID#	名称	保留时间	峰#	面积	高度	面积%
1	RT26.754	26.754	1	445733	14613	0.8511
2	RT31.404	31.404	2	51925644	1237412	99.1489

”

To:

“(*R*)-3h: (*R*)-4-bromo-3-oxo-1,3-dihydroisobenzofuran-1-yl acetate** (HPLC: Chiralpak AD-H, detected at 210 nm, eluent: n-hexane/2-propanol = 90/10, flow rate = 1.0 mL/min, 25°C).

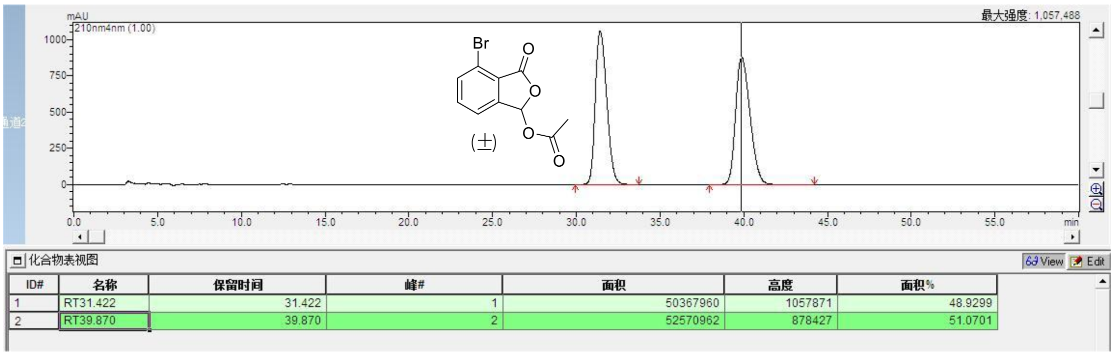

ID#	名称	保留时间	峰#	面积	高度	面积%
1	RT31.422	31.422	1	50367960	1057871	48.9299
2	RT39.870	39.870	2	52570962	878427	51.0701

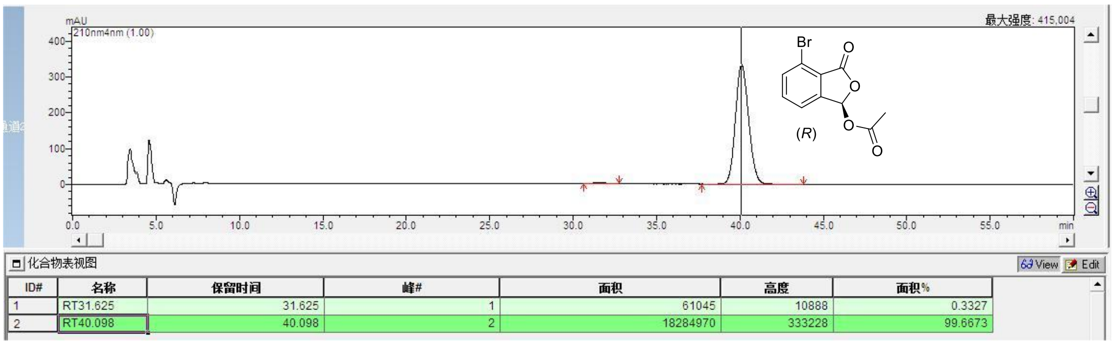

ID#	名称	保留时间	峰#	面积	高度	面积%
1	RT31.625	31.625	1	61045	10888	0.3327
2	RT40.098	40.098	2	18284970	333228	99.6673

Question 18

Page S33: Why are there two different retention time for the same molecule? Please explain more carefully.

Response: Yes, the same molecule should exhibit identical retention times. However, in practice, when synthesizing the second compound, students often confirmed the identical chiral compound *via* TLC and ^{1}H NMR, but they typically do not run an HPLC analysis for the racemate of the corresponding chiral product. Instead, they directly refer to the HPLC data of the original racemic compound. Due to the time interval (sometimes around a month), the retention time of the column typically shifts, leading to variations in retention times. We are sorry for this error. Therefore, we have re-run the product sample by HPLC in a continuous time sequence to correct this discrepancy. Now, the retention time of the reaction sample was nearly consistent with that of the racemic mixture as follows. Please refer to the revised Supporting Information.

Revise (Figures on page S33)

“(*R*)-**3i**: (*R*)-5-bromo-3-oxo-1,3-dihydroisobenzofuran-1-yl acetate (HPLC: Chiralpak IC, detected at 210 nm, eluent: n-hexane/2-propanol = 80/20, flow rate = 1.0 mL/min, 25°C)

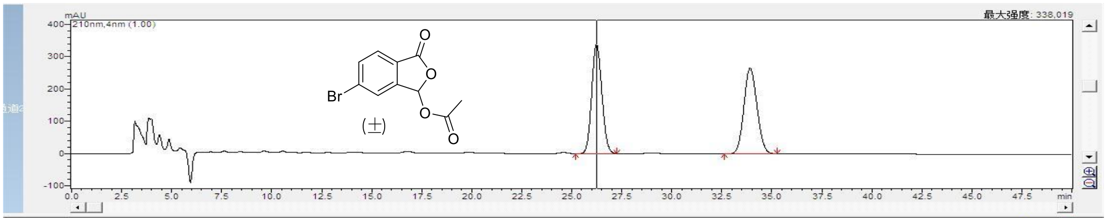

化合物表视图

ID#	名称	保留时间	峰#	面积	高度	面积%
1	RT26.231	26.231	1	12034308	338275	49.8946
2	RT33.915	33.915	2	12085162	265626	50.1054

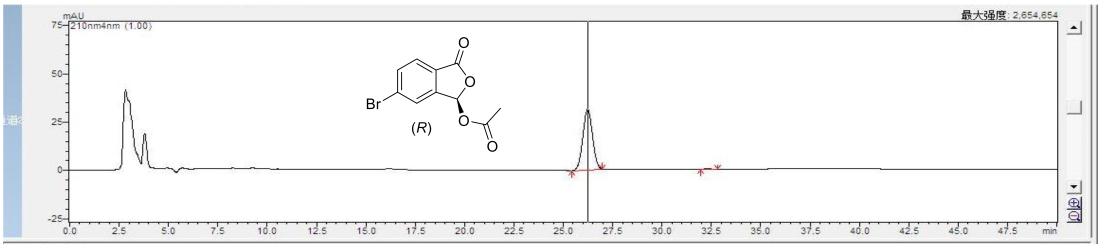

化合物表视图

ID#	名称	保留时间	峰#	面积	高度	面积%
1	RT26.230	26.230	1	1073421	31453	99.8635
2	RT32.427	32.427	2	1467	0	0.1365

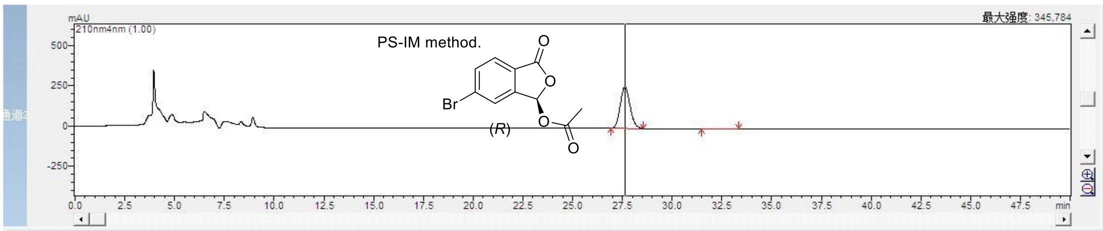

化合物表视图

ID#	名称	保留时间	峰#	面积	高度	面积%
1	RT27.621	27.621	1	8810863	257278	99.2430
2	RT32.062	32.062	2	67207	1839	0.7570

”

To:

“(*R*)-3i: (*R*)-5-bromo-3-oxo-1,3-dihydroisobenzofuran-1-yl acetate (HPLC: Chiralpak IC, detected at 210 nm, eluent: n-hexane/2-propanol = 80/20, flow rate = 1.0 mL/min, 25°C).

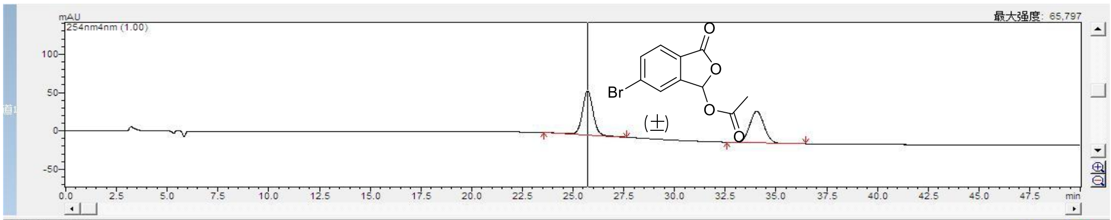

化合物表视图

ID#	名称	保留时间	峰#	面积	高度	面积%
1	RT25.710	25.710	1	2103762	57804	51.0145
2	RT34.045	34.045	2	2020090	41099	48.9855

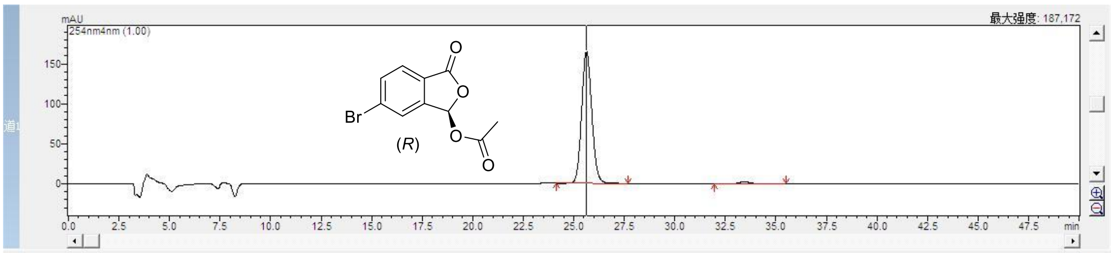

化合物表视图

ID#	名称	保留时间	峰#	面积	高度	面积%
1	RT25.624	25.624	1	5792132	166085	98.9649
2	RT33.344	33.344	2	60579	5956	1.0351

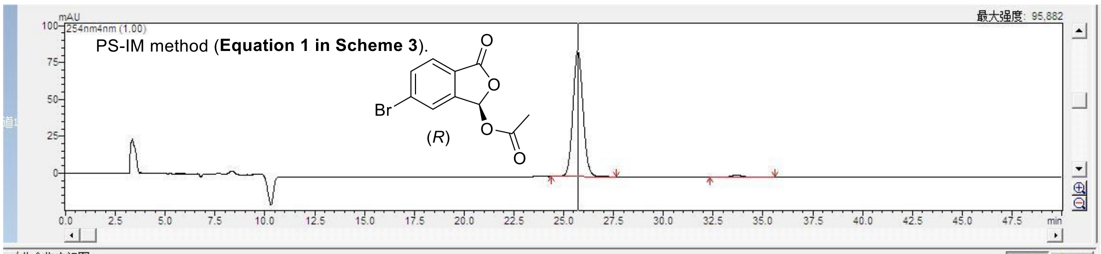

化合物表视图

ID#	名称	保留时间	峰#	面积	高度	面积%
1	RT25.703	25.703	1	2987290	85394	98.3092
2	RT33.471	33.471	2	51377	5193	1.6908

”

Question 19

Page S40: In all the previous cases the R enantiomer is the first one; why is it the second peak on this specific example? Are you sure this is not the S enantiomer?

Response: Yes, due to the nature of the chiral column packing, the elution order of different chiral compounds in HPLC is usually unpredictable, which is a common phenomenon in chiral HPLC analysis. To validate the rationality of this, we also replicated literature data using the chiral catalyst reported in a prior study and obtained comparable results with the same configuration (Please refer to literature data in *Angew. Chem. Int. Ed.* **63**, e202402909 (2024)), thereby confirming that it was (*R*)-configuration rather than the S enantiomer as follows.

(*R*)-3p: (*R*)-6-methyl-3-oxo-1,3-dihydroisobenzofuran-1-yl acetate. (HPLC: Chiralpak AD-H, detected at 210 nm, eluent: n-hexane/2-propanol = 80/20, flow rate = 1.0 mL/min, 25°C).

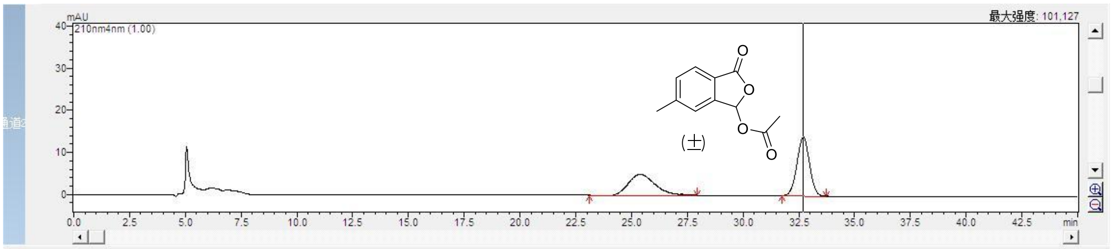

化合物表视图

ID#	名称	保留时间	峰#	面积	高度	面积%
1	RT25.424	25.323	1	432387	5116	49.9207
2	RT32.696	32.186	2	433761	12579	50.0793

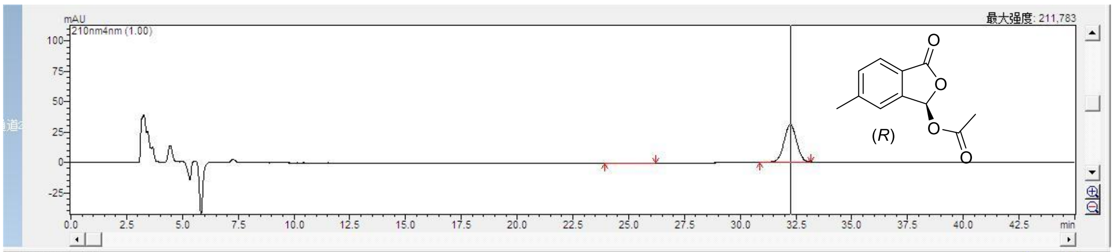

化合物表视图

ID#	名称	保留时间	峰#	面积	高度	面积%
1	RT25.213	25.213	1	6627	285	0.5551
2	RT32.249	32.249	2	1187153	30785	99.4449

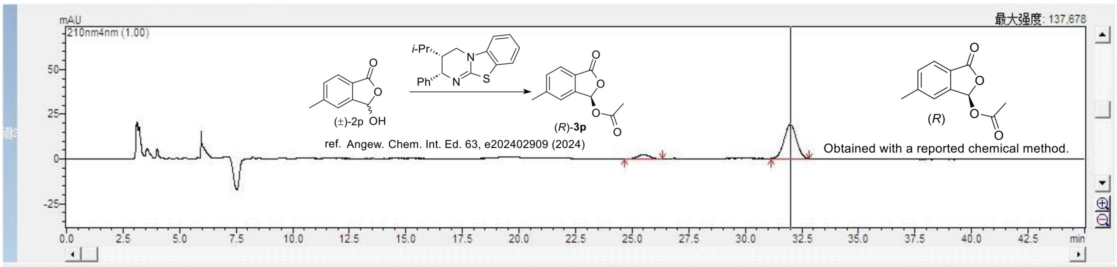

化合物表视图

ID#	名称	保留时间	峰#	面积	高度	面积%
1	RT25.047	25.047	1	114042	2413	13.5614
2	RT31.995	31.995	2	726887	19197	86.4386

Additionally, similar evidence was also observed for **(*R*)-3h** as follows.

(*R*)-3h: (*R*)-4-bromo-3-oxo-1,3-dihydroisobenzofuran-1-yl acetate (HPLC: Chiralpak AD-H, detected at 210 nm, eluent: n-hexane/2-propanol = 80/20, flow rate = 1.0 mL/min, 25°C).

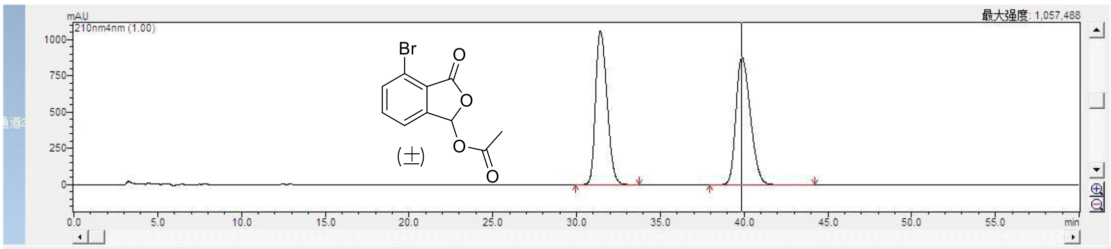

化合物表视图

ID#	名称	保留时间	峰#	面积	高度	面积%
1	RT31.422	31.422	1	50367960	1057871	48.9299
2	RT39.870	39.870	2	52570962	878427	51.0701

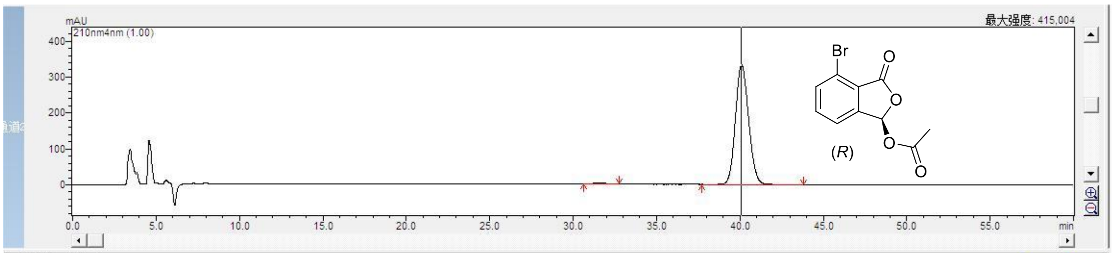

化合物表视图

ID#	名称	保留时间	峰#	面积	高度	面积%
1	RT31.625	31.625	1	61045	10888	0.3327
2	RT40.098	40.098	2	18284970	333228	99.6673

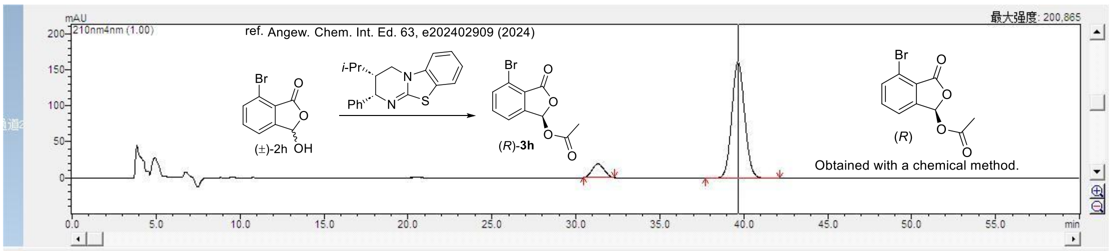

化合物表视图

ID#	名称	保留时间	峰#	面积	高度	面积%
1	RT31.376	31.376	1	496450	35997	5.4850
2	RT39.659	39.659	2	8554565	161189	94.5150

”

Question 20

Page S68, S69: In all the previous cases the R enantiomer is the first one; why is it the second peak on this specific example? Are you sure this is not the S enantiomer?

Response: Yes, the reviewer is right. To further confirm the elution order, we employed two methods. For the chiral product (*R*)-**6d** (page S68), we also replicated literature data using the chiral catalyst reported in prior study and obtained comparable results with the same configuration (Please refer to literature data in *Angew. Chem. Int. Ed.* **63**, e202402909 (2024)), thereby confirming that it was (*R*)-configuration rather than the S enantiomer as follows.

For the chiral product (*R*)-**6e** (page S69), we adjusted the eluent composition from n-hexane/2-propanol = 80/20 to n-hexane/2-propanol = 95/5. The reduction in solvent polarity allowed sufficient retention time to accurately determine the peak elution order. Upon re-running the product sample by HPLC, we confirmed the situation described by the reviewer. We have now replaced the original data with this new data. Please refer to the revised Supporting Information provided below.

Revise (Figures on page S68):

"(*R*)-6d: (*R*)-1-(4-chlorophenyl)-3-oxo-1,3-dihydroisobenzofuran-1-ylacetate. (HPLC: Chiralpak IC, detected at 210 nm, eluent: n-hexane/2-propanol = 80/20, flow rate = 1.0 mL/min, 25°C).

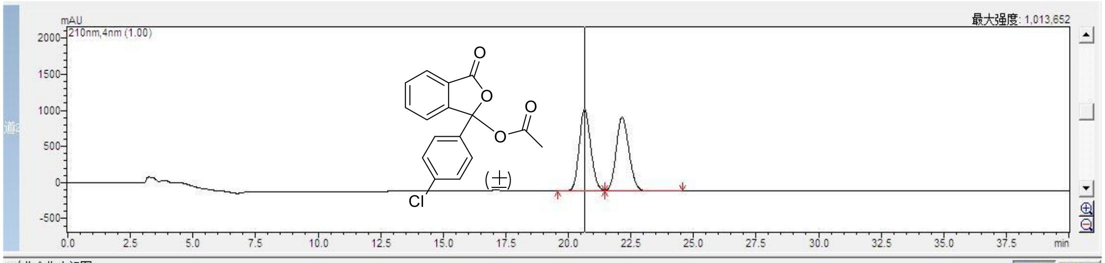

化合物表视图

ID#	名称	保留时间	峰#	面积	高度	面积%
1	RT20.645	20.645	1	36795724	1134863	50.1769
2	RT22.141	22.141	2	36536306	1028316	49.8231

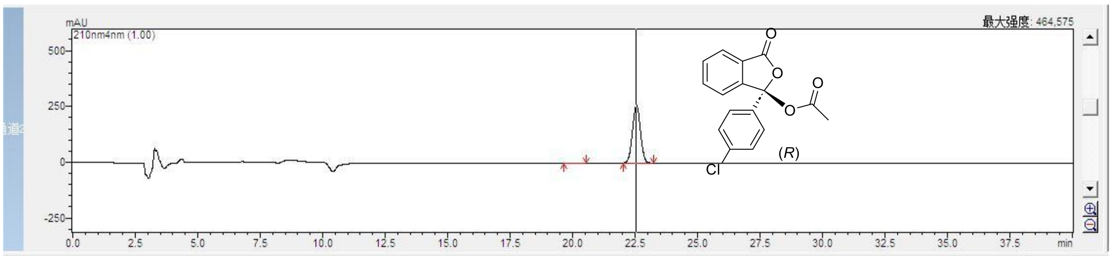

化合物表视图

ID#	名称	保留时间	峰#	面积	高度	面积%
1	RT20.010	20.010	1	22456	786	0.1679
2	RT22.538	22.538	2	13350249	452377	99.8321

To:

"

(*R*)-6d: (*R*)-1-(4-chlorophenyl)-3-oxo-1,3-dihydroisobenzofuran-1-ylacetate. (HPLC: Chiralpak OD-H, detected at 210 nm, eluent: n-hexane/2-propanol = 90/10, flow rate = 1.0 mL/min, 25°C).

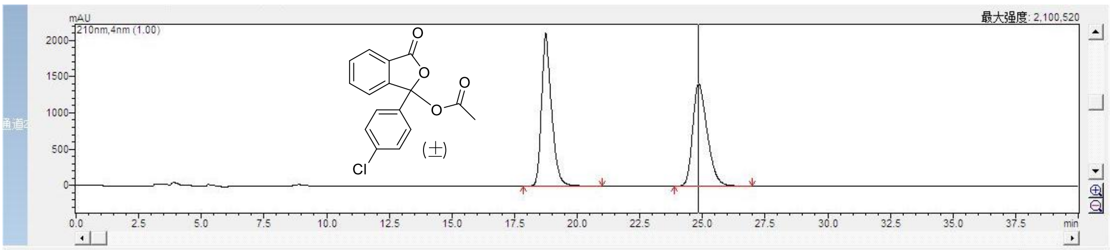

□化合物表视图

ID#	名称	保留时间	峰#	面积	高度	面积%
1	RT18.736	18.736	1	57995681	2106932	50.5461
2	RT24.848	24.848	2	56742429	1409660	49.4539

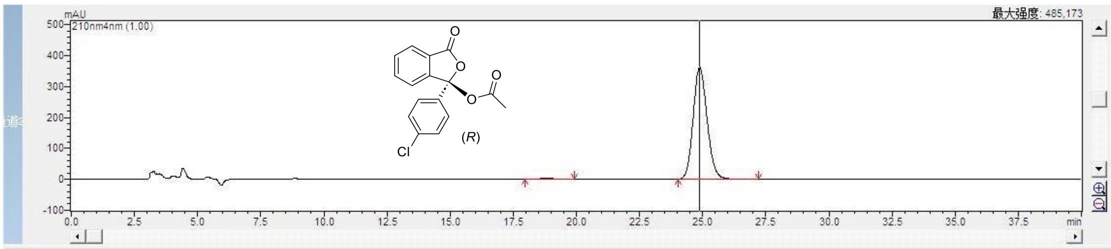

□化合物表视图

ID#	名称	保留时间	峰#	面积	高度	面积%
1	RT18.750	18.750	1	28830	9791	0.2073
2	RT24.879	24.879	2	13875902	361862	99.7927

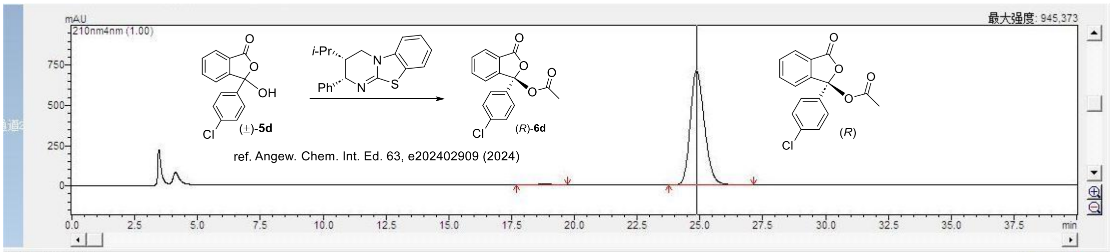

□化合物表视图

ID#	名称	保留时间	峰#	面积	高度	面积%
1	RT18.737	18.737	1	436100	73534	1.5392
2	RT24.864	24.864	2	27896522	708952	98.4608

”

Revise (Figures on page S69):

“(*R*)-6e:(R)-1-(4-bromophenyl)-3-oxo-1,3-dihydroisobenzofuran-1-ylacetate. (HPLC: Chiralpak IC, detected at 210 nm, eluent: n-hexane/2-propanol = 80/20, flow rate = 1.0 mL/min, 25°C).

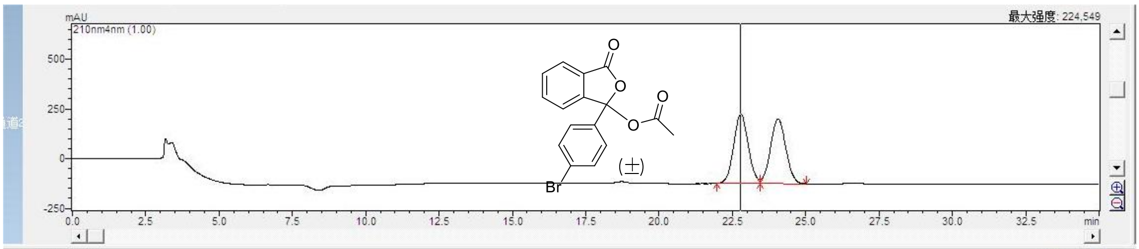

□化合物表视图

ID#	名称	保留时间	峰#	面积	高度	面积%
1	RT22.781	22.781	1	12089065	349500	50.5469
2	RT24.049	24.049	2	11827447	326513	49.4531

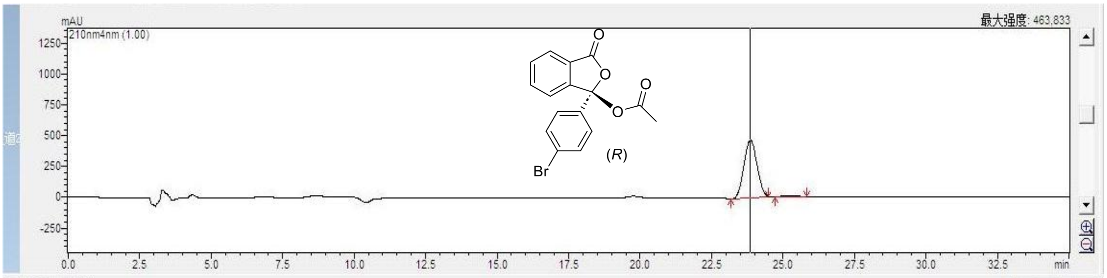

化合物表视图

ID#	名称	保留时间	峰#	面积	高度	面积%
1	RT23.851	23.851	1	15003116	469126	99.2991
2	RT25.168	25.168	2	105899	19447	0.7009

”

To:

“(*R*)-6e:(R)-1-(4-bromophenyl)-3-oxo-1,3-dihydroisobenzofuran-1-ylacetate. (HPLC: Chiralpak IC, detected at 210 nm, eluent: n-hexane/2-propanol = 95/5, flow rate = 1.0 mL/min, 25°C).

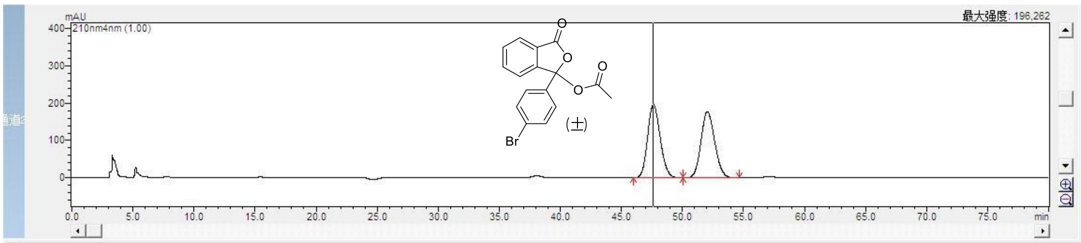

化合物表视图

ID#	名称	保留时间	峰#	面积	高度	面积%
1	RT47.619	47.619	1	14160042	196268	50.4830
2	RT52.048	52.048	2	13889077	177266	49.5170

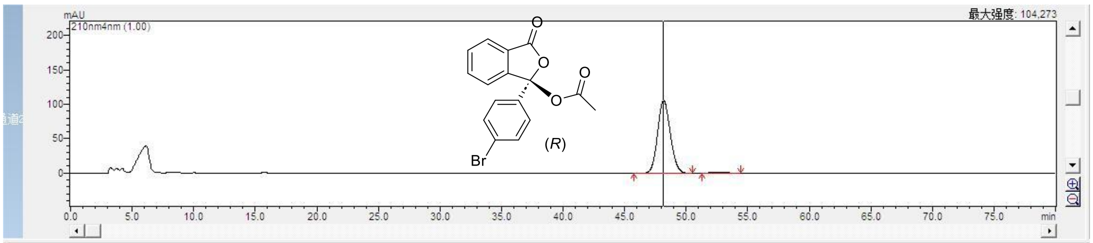

化合物表视图

ID#	名称	保留时间	峰#	面积	高度	面积%
1	RT48.161	48.161	1	7627053	106084	99.5445
2	RT52.623	52.623	2	34898	3247	0.4555

”

Question 21

Page S71: the two HPLC chromatogram don’t testify the production of one of the enantiomer; the retention times are different between the racemic mixture and the reaction sample (with only one peak).

Response: Yes, we adjusted the eluent composition from n-hexane/2-propanol = 80/20 to n-hexane/2-propanol = 95/5. The reduction in solvent polarity allowed sufficient retention time to accurately determine the peak elution order. Therefore, we have re-run the product sample by HPLC to correct this discrepancy. Now, the retention time of the reaction sample was consistent with that of the racemic mixture as follows. Please refer to the revised Supporting Information.

<u>Revise (Figures on page S71):</u> "(*R*)-6g: (*R*)-1-(4-ethylphenyl)-3-oxo-1,3-dihydroisobenzofuran-1-yl acetate. (HPLC: Chiralpak IC, detected at 210 nm, eluent: <u>n-hexane/2-propanol = 80/20</u>, flow rate = 1.0 mL/min, 25°C).

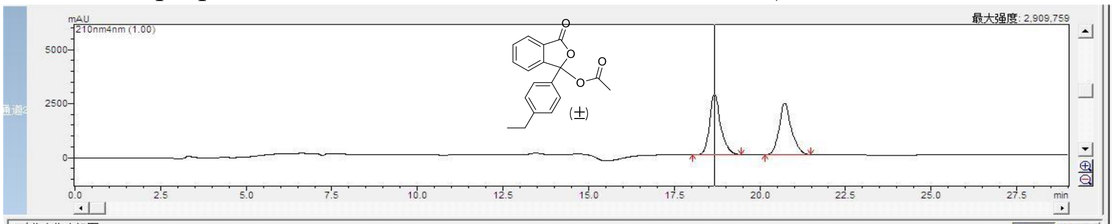

化合物表视图

ID#	名称	保留时间	峰#	面积	高度	面积%
1	RT18.664	18.664	1	63175415	2775949	51.6018
2	RT20.718	20.718	2	59253356	2384054	48.3982

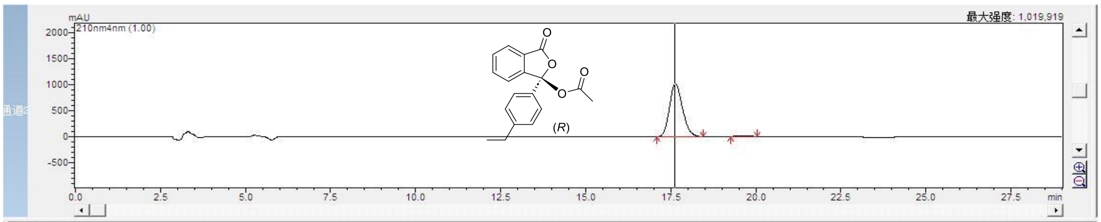

化合物表视图

ID#	名称	保留时间	峰#	面积	高度	面积%
1	RT17.630	17.630	1	27035415	1019590	99.1679
2	RT19.644	19.644	2	226847	37736	0.8321

"

<u>To:</u> "(*R*)-6g: (*R*)-1-(4-ethylphenyl)-3-oxo-1,3-dihydroisobenzofuran-1-ylacetate. (HPLC: Chiralpak IC, detected at 210 nm, eluent: <u>n-hexane/2-propanol = 95/5,</u> flow rate = 1.0 mL/min, 25°C).

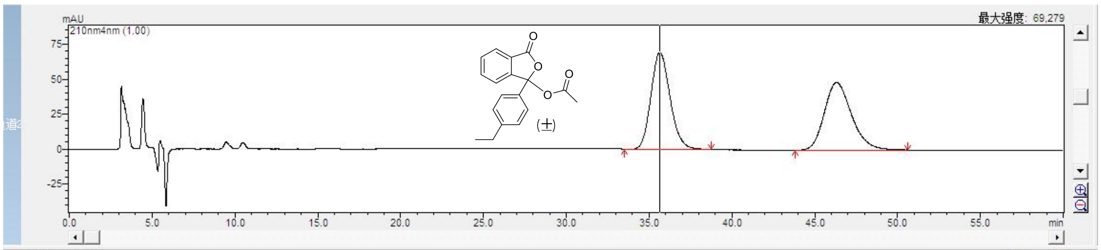

化合物表视图

ID#	名称	保留时间	峰#	面积	高度	面积%
1	RT35.661	35.661	1	5593101	69493	50.2864
2	RT46.319	46.319	2	5529393	48330	49.7136

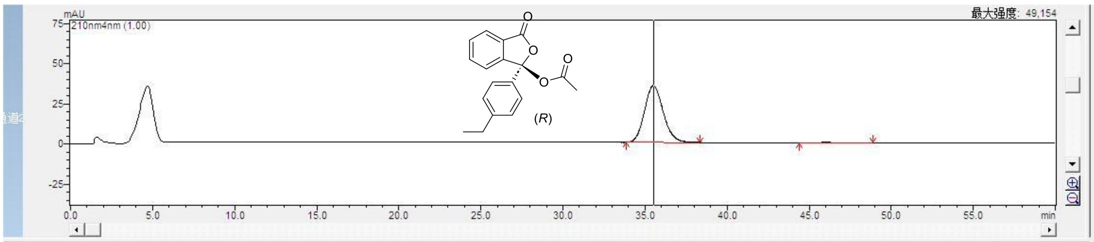

化合物表视图

ID#	名称	保留时间	峰#	面积	高度	面积%
1	RT35.505	35.505	1	2819595	35321	99.5058
2	RT46.177	46.177	2	14003	889	0.4942

"

<u>Question 22</u>

Page S80: the two HPLC chromatogram don't testify the production of one of the enantiomer; the retention times are different between the racemic mixture and the reaction sample (with only one peak).

<u>Response:</u> Yes, we have re-run the product sample by HPLC to correct this discrepancy

by adjusting the eluent composition from n-hexane/2-propanol = 80/20 to n-hexane/2-propanol = 95/5. Now, the retention time of the reaction sample was consistent with that of the racemic mixture as follows. Please refer to the revised Supporting Information.

Revise (Figures on page S71): “(*R*)-3u: (*R*)-3-oxo-1,3-dihydroisobenzofuran-1-yl 2-(1-(4-chlorobenzoyl)-5-methoxy-2-methyl-3a,7a-dihydro-1H-indol-3-yl)acetate. **(**HPLC: Chiralpak IC, detected at 210 nm, eluent: n-hexane/2-propanol = 80/20, flow rate = 1.0 mL/min, 25°C).

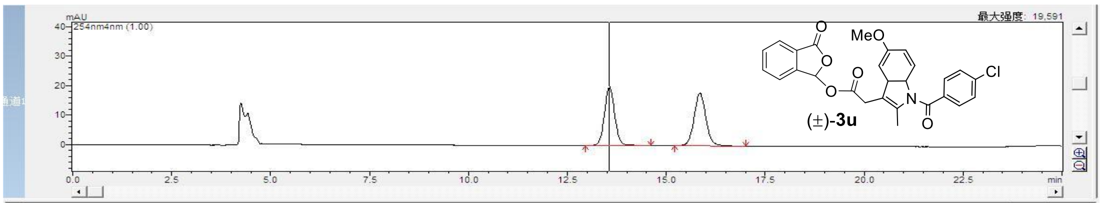

化合物表视图

ID#	名称	保留时间	峰#	面积	高度	面积%
1	RT13.558	13.558	1	360860	19960	49.3905
2	RT15.844	15.844	2	369765	17830	50.6095

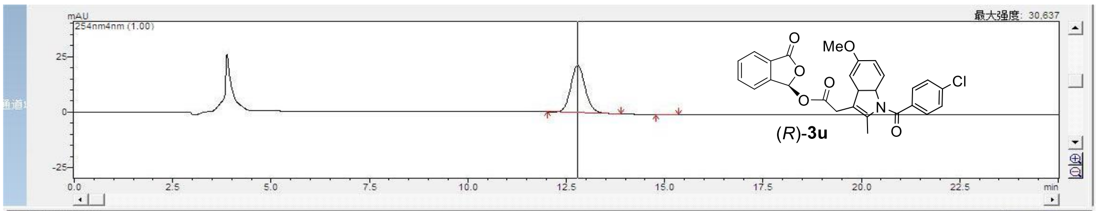

化合物表视图

ID#	名称	保留时间	峰#	面积	高度	面积%
1	RT12.786	12.786	1	545556	21351	99.9568
2	RT14.787	14.787	2	236	1	0.0432

”

To: “(*R*)-3u: (*R*)-3-oxo-1,3-dihydroisobenzofuran-1-yl 2-(1-(4-chlorobenzoyl)-5-methoxy-2-methyl-3a,7a-dihydro-1H-indol-3-yl)acetate. **(**HPLC: Chiralpak IC, detected at 210 nm, eluent: n-hexane/2-propanol = 95/5, flow rate = 1.0 mL/min, 25°C).

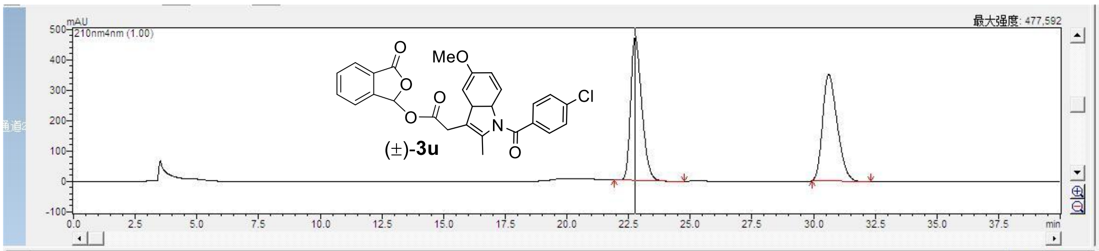

化合物表视图

ID#	名称	保留时间	峰#	面积	高度	面积%
1	RT22.764	22.764	1	14750053	474284	50.5061
2	RT30.608	30.608	2	14454429	350616	49.4939

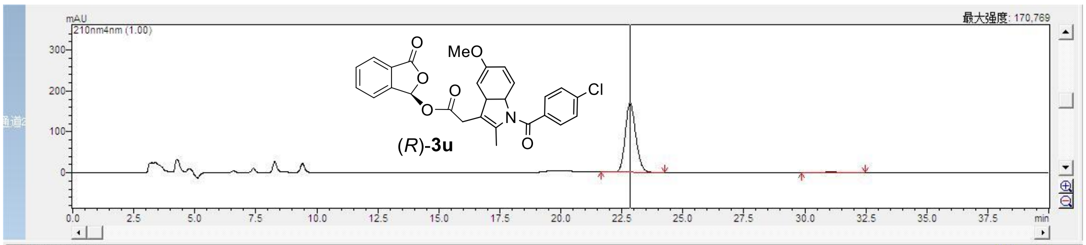

化合物表视图

ID#	名称	保留时间	峰#	面积	高度	面积%
1	RT22.836	22.836	1	5075417	169316	98.9488
2	RT30.699	30.699	2	53918	7493	1.0512

”

Responses to the comment of Reviewer 3

Reviewer: 3
Reviewer #3 (Remarks to the Author):
I co-reviewed this manuscript with one of the reviewers who provided the listed reports. This is part of the Nature Communications initiative to facilitate training in peer review and to provide appropriate recognition for Early Career Researchers who co-review manuscripts.

Response: Thank you very much for your valuable comments, which have helped us further improve the quality. Please refer to the corresponding reply. Also, see the revised manuscript and the revised Supporting Information.

Responses to the comment of Reviewer 4

Reviewer #4 (Remarks to the Author): Comments:
Authors have presented a methodology for developing novel chemoenzymatic DKR procedures using DBU as racemization catalysts and CAL-B as selective acylation reagent, employing a PDMS membrane for the separation of both catalysts in order to avoid all the issues derived from the direct combination of these two catalysts. A wide set of 3-hydroxyphthalides are selectively resolved with good yields and excellent enantiomeric excesses, but the manuscript has to be improved in certain aspects:

1. Throughout the manuscript, the PDMS-mediated decoupling of chemo- and biocatalysis is presented as a conceptual advance that, as authors stated "bypasses fundamental DKR limitations" (pages 4–7). But the PDMS-thimble compartmentalization has been extensively used to resolve catalyst incompatibility, as shown in references from 29 to 38. Thus, aapart to increase the applicability of this type of systems by using a different reaction class, it is not bery clear what is conceptually new. A reformulation of this statement will be great.
2. - This approach has been studied only with one family of substrates (tetra-substituted 3-hydroxyphthalides presenting different substituents in the aromatic ring), practically one biocatalysts (CAL-B and one example with PS-IM) and only DBU used as racemisation base. The applicability of the study to other substrates will improve it in a decissive way.
3. - Authors compared in Table 1 a "classical" DKR with DBU for racemic 2a with the PDMS-modulated DKR. Apart that there is a reaction (III) in the table footnotes that does not appear in the Table body, this comparision is not very accurate as the "classical" DKR is performed in not optimal conditions. DBU is not the best racemisation catalyst for CAL-B and authors have indicated other tentative bases in which the DKR is performed (references 20-25). Clearly, the DBU-CAL-B classical DKR is not going to work in the same way as the novel methodology. Authors can also try to compare their process with a sequential DKR in which the DBU-racemization is conducted first and then the biocatalyzed acylation.
4. - Process has been scaled up to 4.0 mmol, which for this type of enzymes is not so high, but no studies on the PDMS stability and reusability for diferent cycles have been performed.
5. - The use of DBU in stoichiometric amounts can present some enviormental issues. In addition, the use of this base has shown certain side-reactions, have the authors observed any other product in the reaction medium?
6. - Finally, I am also missing some studies that are basic for the manuscript: Authors have not measured the diffusion rates through PDMS; There is no experimental calculation of the DBU-racemization kinetic and finally, there are no results on the retention of the DBU base in its compartment. Have been base-transfer observed?
7. Thus, the manuscript has to be improved in these ways to be suitable for publication in Nature Communications, No demonstration of DBU retention in Compartment-A.

Question 1:

Throughout the manuscript, the PDMS-mediated decoupling of chemo- and biocatalysis is presented as a conceptual advance that, as authors stated "bypasses fundamental DKR limitations" (pages 4–7). But the - PDMS-thimble compartmentalization has been extensively used to resolve catalyst incompatibility, as shown in references from 29 to 38. Thus, apart to increase the applicability of this type of systems by using a different reaction class, it is not very clear what is conceptually new. A reformulation of this statement will be great.

Response: Yes, we agree with this opinion of the reviewer. According to the suggestion, we have revised the statements to demonstrate the characteristics of a membrane-modulated DKR strategy as follows.

Revise (page 4, line 15): "Building on these advantages, and inspired by the elegant work on physical separation of enantiomeric products,[39] we introduce a membrane-modulated DKR strategy that bypasses the constraints of conventional DKR."

To: "Inspired by the advantage of the continuous-flow DKR approach in achieving quantitative chiral conversion,[38] and combining the feature of physical separation of enantiomeric products in a membrane-mediated KR process,[39] a membrane-modulated DKR strategy is expected to decouple racemization and resolution into two independent stages, thereby overcoming the limitations of conventional batch DKR processes."

Question 2:

This approach has been studied only with one family of substrates (tetra-substituted 3-hydroxyphthalides presenting different substituents in the aromatic ring), practically one biocatalysts (CAL-B and one example with PS-IM) and only DBU used as racemisation base. The applicability of the study to other substrates will improve it in a decissive way.

Response: Yes, the reviewer is right that the applicability of the study to other substrates will expand its potential application scope. According to the suggestion, we have investigated the feasibility of other substrates. It was found that the PDMS-modulated DKR process was also applicable to a five-membered dihydrofuran derivative, affording (*R*)-**3x** in 81% yield with 99% ee in the following Scheme. In addition to the construction of five-membered heterocyclic (*R*)-**3x**, it was also found that this type of PDMS-modulated DKR process is suitable for the construction of six-membered heterocyclic (*R*)-**3y**, as shown below. Additionally, the relevant statements have been revised in the manuscript, the characterizations have been provided in the Supporting Information, and the references have been cited as follows.

Scheme. The PDMS-modulated DKR of **1s.**

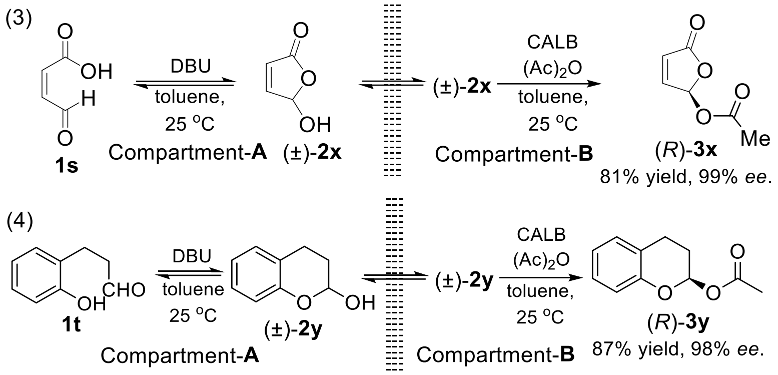

<u>Revise (page 17, line 3):</u> **"**Second, for heteroaryl substrate **2r**, which could not be transformed by conventional DKR methods, the membrane-modulated process proceeded smoothly with both CALB and PS-IM, affording the desired products in high yield and excellent enantioselectivity (Equation 2, Scheme 3).**"**

<u>To:</u> **"**Second, for heteroaryl substrate **2r**, which could not be transformed by conventional DKR methods, the membrane-modulated process proceeded smoothly with both CALB and PS-IM, affording the desired products in high yield and excellent enantioselectivity (Equation 2, Scheme 3). <u>Furthermore, it was also found that the PDMS-modulated DKR process was suitable for other types of substrates, in which a five-membered dihydrofuran derivative (*R*)-**3x** and a six-membered dihydropyran derivative (*R*)-**3y** could be stably obtained in high yield and with excellent enantioselectivity (Equations 3 and 4, Scheme 3).[57]</u>**"**

<u>Revise (page 16, Scheme 3):</u>

"

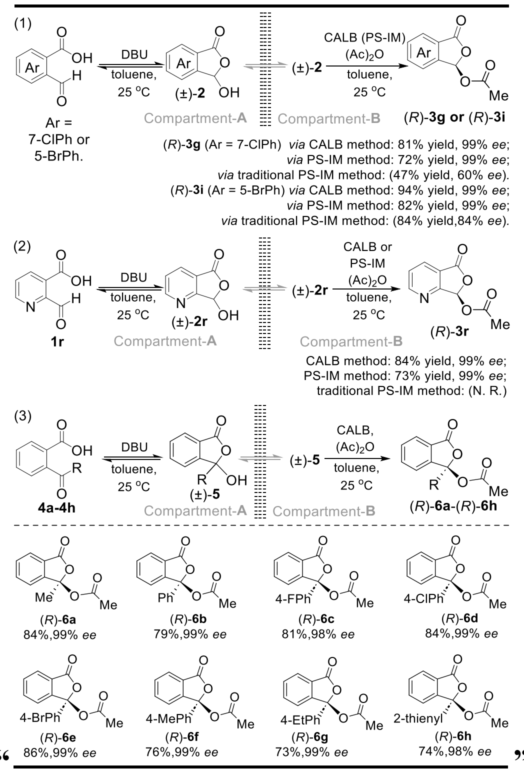

"

To:“

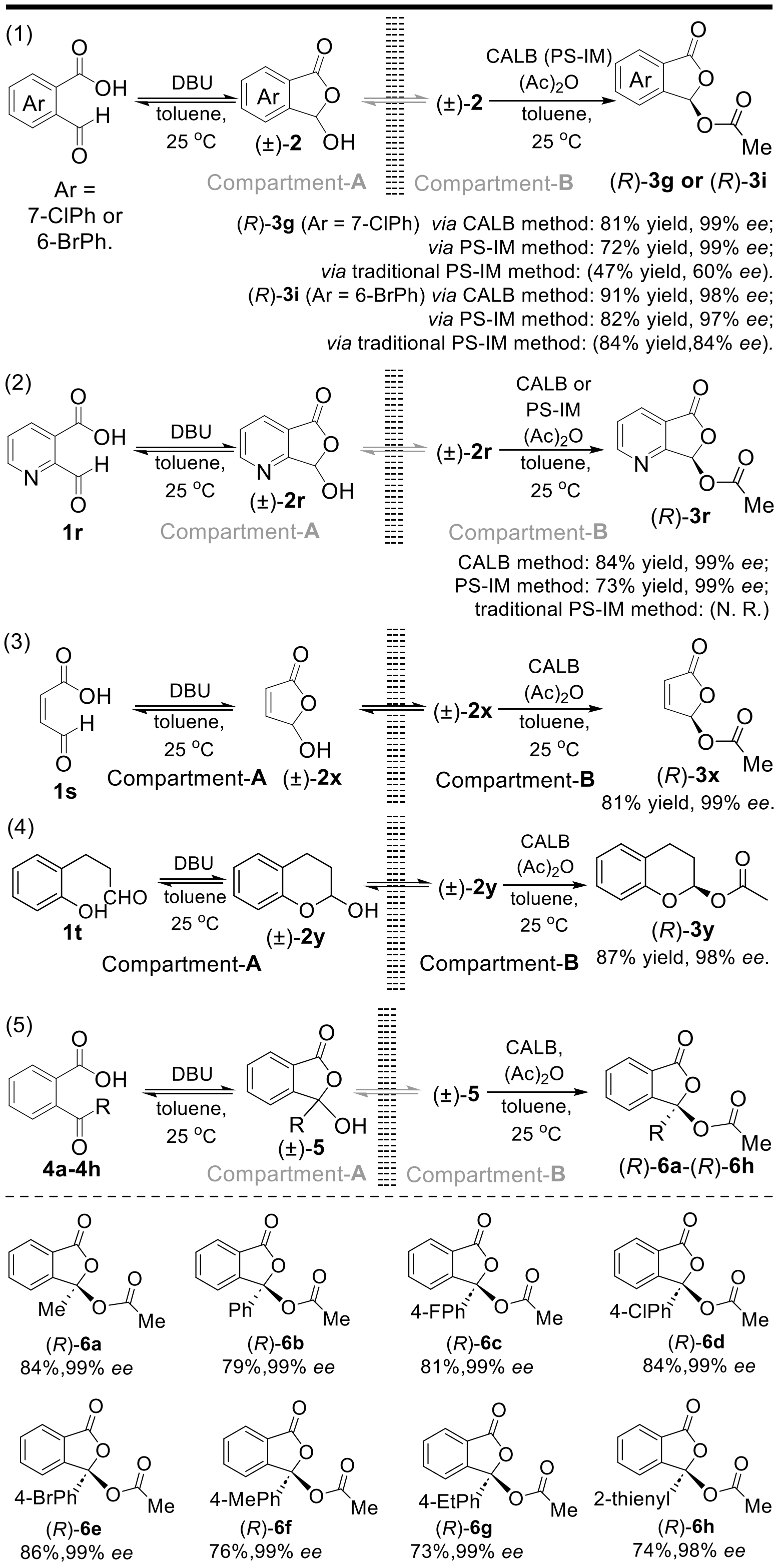

”

Also, the references have been cited in the manuscript as ref. 57, as follows

Add reference: “57. Glazier, D. A., Schroeder, J. M., Liu, J. & Tang, W. Organocatalyst-mediated dynamic kinetic enantioselective acylation of 2-chromanols, *Adv. Synth. Catal.* **360**, 4646-4649 (2018).

Also, the characterizations have been provided in the Supporting Information as follows.

(*R*)-3x: (*R*)-5-oxo-2,5-dihydrofuran-2-yl acetate (HPLC: Chiralpak OJ-H, detected at 210 nm, eluent: n-hexane/2-propanol = 95/5, flow rate = 1.0 mL/min, 25°C).

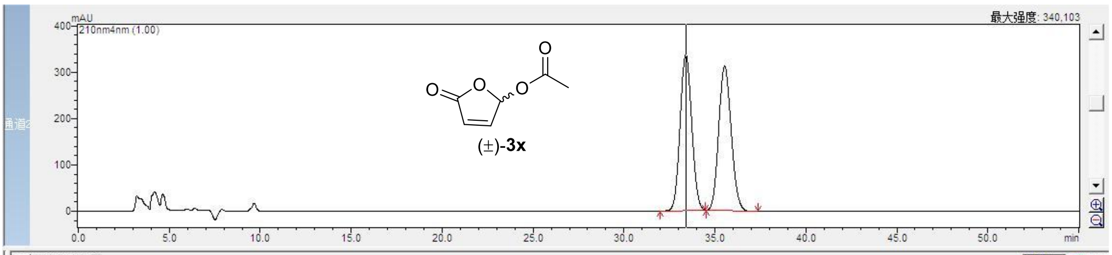

化合物表视图

ID#	名称	保留时间	峰#	面积	高度	面积%
1	RT33.389	33.389	1	14924001	336473	49.8868
2	RT35.523	35.523	2	14991755	312528	50.1132

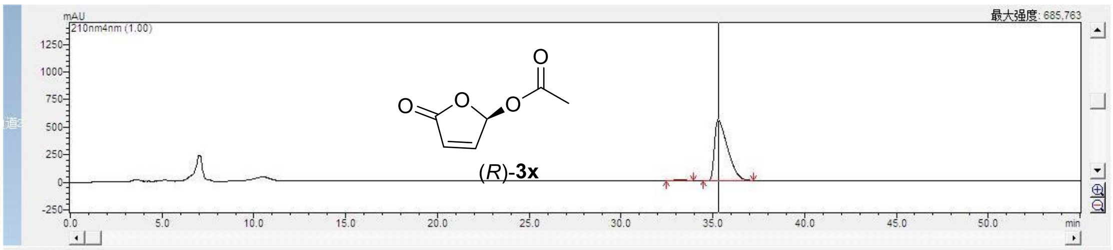

化合物表视图

ID#	名称	保留时间	峰#	面积	高度	面积%
1	RT24.718	33.219	1	69642	16592	0.2551
2	RT29.119	35.310	2	25920721	555046	99.7449

(*R*)-3x: (*R*)-5-oxo-2,5-dihydrofuran-2-yl acetate.

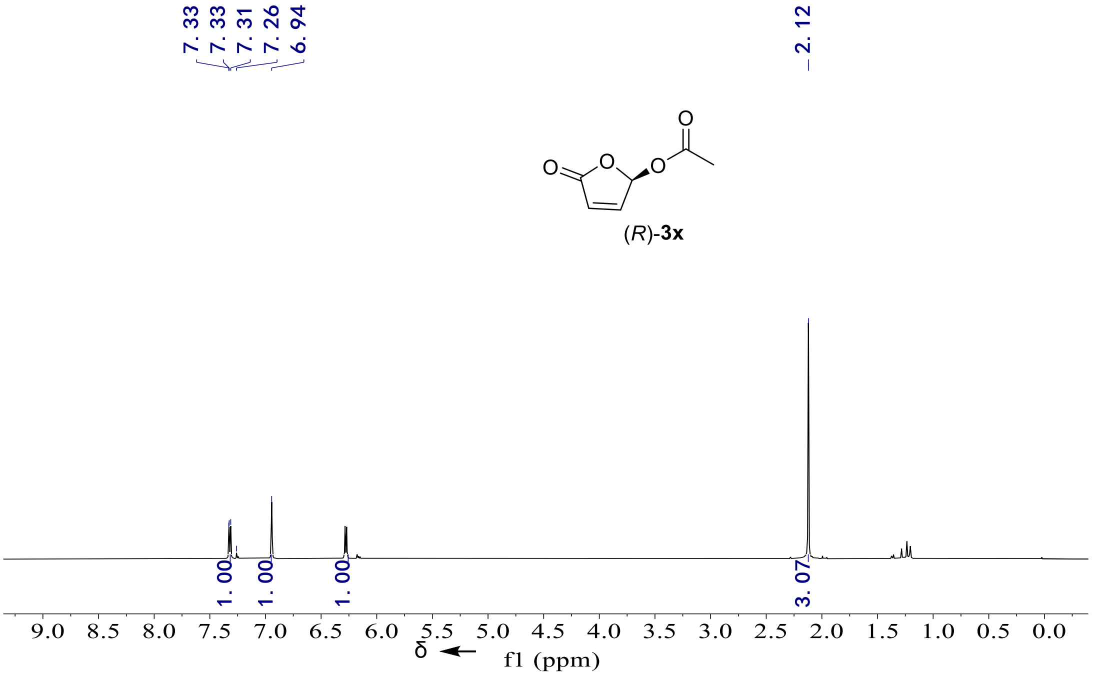

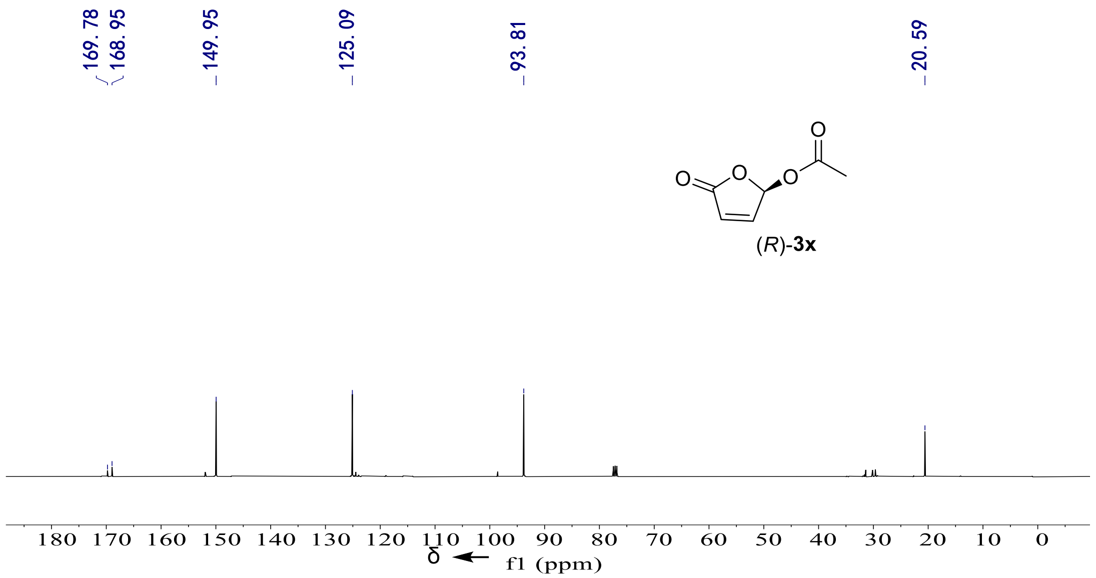

(*R*)-3y: (*R*)-chroman-2-yl acetate (HPLC: Chiracel IC, detected at 210 nm, eluent: n-hexane/2-propanol = 98/02, flow rate = 1.0 mL/min, 25°C).

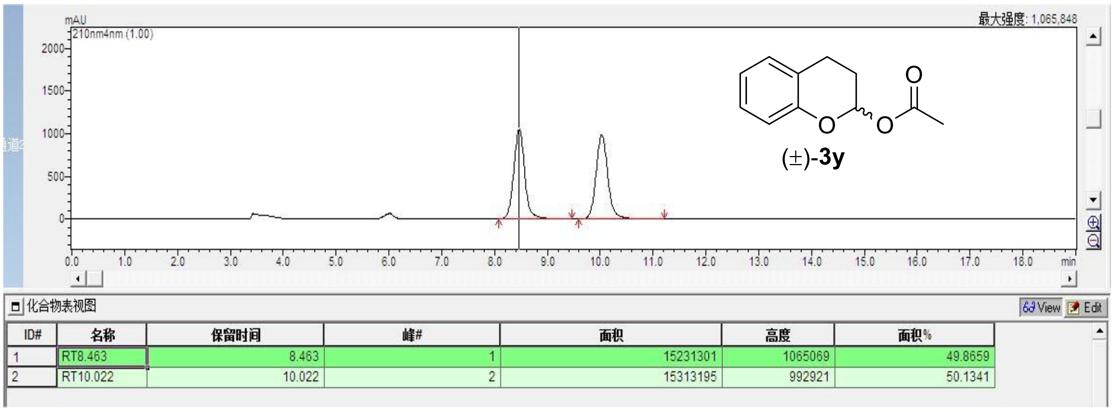

ID#	名称	保留时间	峰#	面积	高度	面积%
1	RT8.463	8.463	1	15231301	1065069	49.8659
2	RT10.022	10.022	2	15313195	992921	50.1341

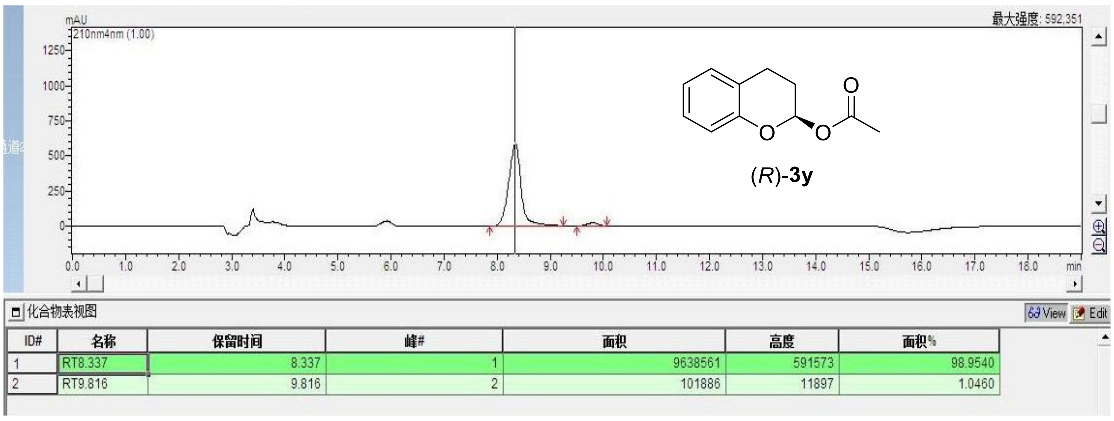

ID#	名称	保留时间	峰#	面积	高度	面积%
1	RT8.337	8.337	1	9638561	591573	98.9540
2	RT9.816	9.816	2	101886	11897	1.0460

(*R*)-3y: (*R*)-chroman-2-yl acetate

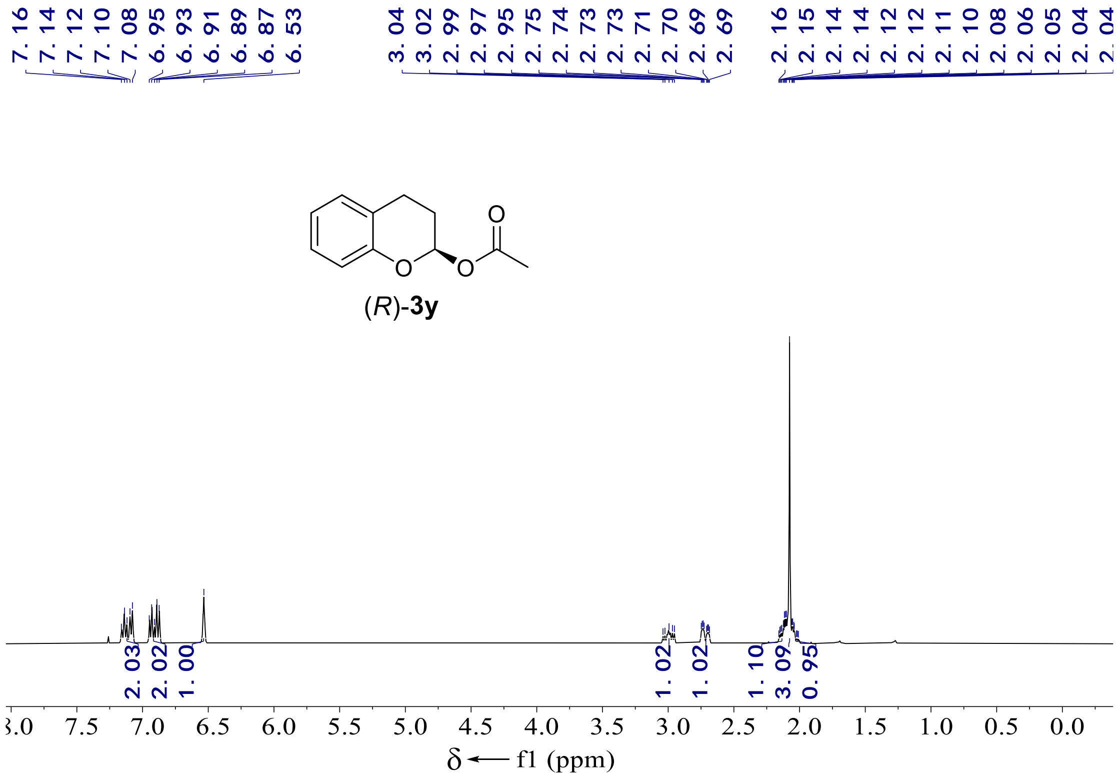

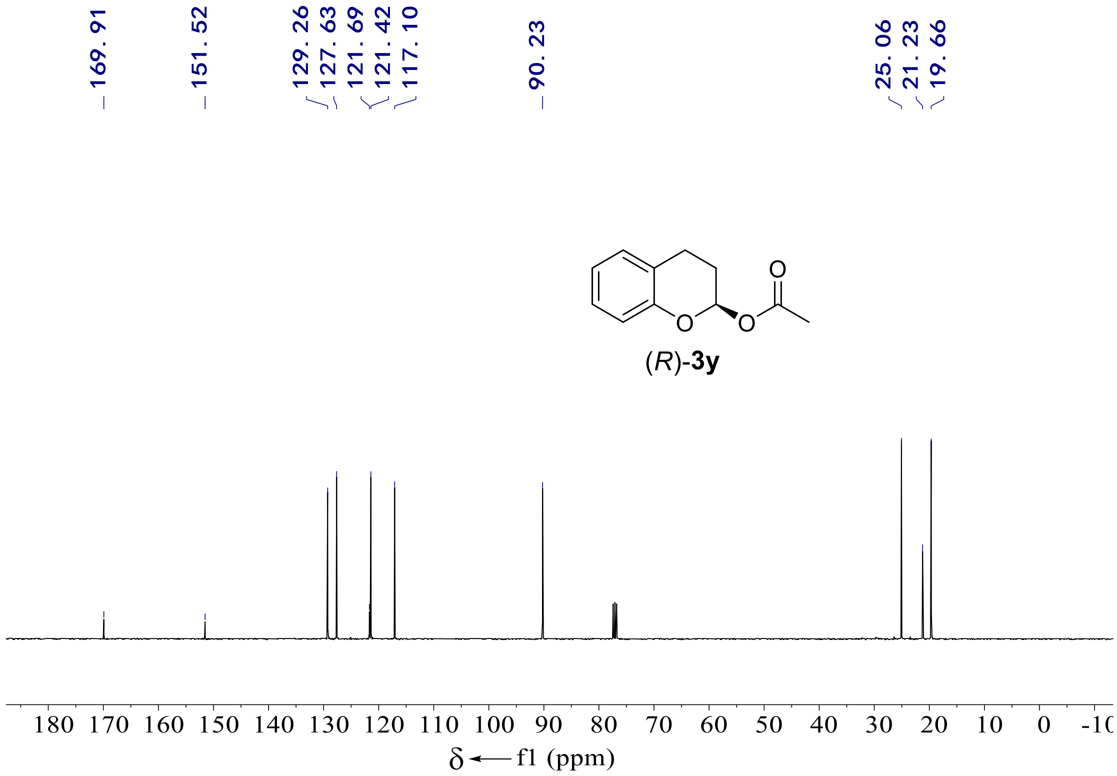

Question 3:

Authors compared in Table 1 a "classical" DKR with DBU for racemic 2a with the PDMS-modulated DKR. Apart that there is a reaction (III) in the table footnotes that does not appear in the Table body, this comparision is not very accurate as the "classical" DKR is performed in not optimal conditions. DBU is not the best racemisation catalyst for CAL-B and authors have indicated other tentative bases in which the DKR is performed (references 20-25). Clearly, the DBU-CAL-B classical DKR is not going to work in the same way as the novel methodology. Authors can also try to compare their process with a sequential DKR in which the DBU-racemization is conducted first and then the biocatalyzed acylation.

Response: Thank you very much for your valuable comments, which have helped us further improve the quality and completeness of the manuscript. We are sorry for the error in the table footnote regarding Reaction (**III**), which actually refers to Reaction (**II**). Now we have corrected this error (Please refer to the revised Table 1 below).

Regarding the issue that "this comparison is not very accurate as the 'classical' DKR is performed in not optimal conditions," the reviewer is right that DBU is not the best racemisation catalyst for CALB. Actually, we have already carried out the comparison of a two-step sequential DKR process with the reported optimal conditions in **Scheme 1A** (The comparative DKR process is actually a two-step sequential dynamic kinetic resolution, which, to a certain extent, implies that DBU-catalyzed racemization is performed first, followed by biocatalyzed acylation in a one-pot manner within the same reaction system. Please refer to the comparison of conventional DBU-promoted DKR in Scheme 1A for the single-step KR, the DBU-mediated DKR, as well as the DKR processes with different DBU loadings). These experiments illustrate that, due to the inherent deactivation of CALB enzyme by DBU, attempting a traditional one-pot DKR process under any of these conditions results in poor outcomes (significantly lower yield and/or enantioselectivity). This is because, in the traditional DKR, the DBU-racemization and CALB-catalyzed resolution stages are forced to proceed simultaneously in a one-pot manner. Notably, unless the two stages are separated, a DKR under any of these reaction sequences will either suppress the reaction yield or result in reduced ee values.

According to the reviewer's suggestions, we also utilized the optimal PS-IM as the best biocatalyst and the optimal triethylamine as racemization catalyst (**ref. 25**. *J. Org. Chem.* 2023, 88, 3897-3902) to compare the catalytic performance in our PDMS-modulated DKR process. It was found that the reaction could steadily provide the final (*R*)-**3a** with an 88% yield and 99% *ee*, which was better than the results (82% yield and 96% ee) of the reported outcomes. We have now added the data to Table 1 and revised the corresponding statements in the manuscript as follows.

Revise (page 8, line 9): "Finally, in the evaluation of an integrated PDMS-modulated DKR (**II**) based on the optimizations (see Supplementary Experimental and Table S2, SI), it was observed that the model reaction could steadily provide the final (*R*)-**3a** with a 91% yield and 99% *ee* (entry 8), in which the permeation of less than 4.8% DBU from Compartment-**A** to Compartment-**B** and 0.2% acetic anhydride from Compartment-**B** to Compartment-**A** had a negligible impact on the enantioselectivity of the product, as confirmed by GC analysis (see Supplementary Figure S1, SI). Furthermore, this result was also superior to the outcomes obtained with other DBU loadings (entry 8 versus entries 9 and 10)."

To: "Finally, in the evaluation of an integrated PDMS-modulated DKR (**II**) based on the optimizations (see Supplementary Experimental and Table S2, SI), it was observed that the model reaction could steadily provide the final (*R*)-**3a** with a 91% yield and 99% *ee* (entry 8) , in which the permeation of less than 4.8% DBU from Compartment-**A** to Compartment-**B** and 0.2% acetic anhydride from Compartment-**B** to Compartment-**A** had a negligible impact on the enantioselectivity of the product, as confirmed by GC analysis (see Supplementary Figure S1, SI). Such a result outperformed the reported outcomes when using triethylamine as the racemization catalyst and PS-IM as the biocatalyst (entry 8 versus entry 9).[25] Furthermore, this result was also superior to the outcomes obtained with other DBU loadings (entry 8 versus entries 10 and 11)."

Revise (page 7, Table 1): "

Entry	Type	Catalyst(s)/solvent(s)	%Yield of (*R*)-**3a**^{b}	%*ee*) of (*R*)-**3a**^{b}
1	**I**	CALB/toluene	48	99
2	**I**	CALB/toluene + DBU	68	3
3	**I**	CALB/toluene + pyridine	59	27
4	**I**	CALB/toluene + piperidine	61	13
5	**I**	CALB/toluene + Et_3N	53	21
6	**I**	CALB/toluene + $NaHCO_3$	55	46
7	**I**	CALB/toluene + K_2CO_3	58	45
8	**II**	DBU/toluene + CALB/toluene	91	99
9^c	**II**	DBU/toluene + CALB/toluene	85	99
10^d	**II**	DBU/toluene + CALB/toluene	81	99
11^e	**II**	DBU/toluene + CALB/toluene	83	99
12^f	**II**	DBU/toluene + CALB/toluene	87	99

^{a} Reaction conditions: For reaction (**I**), **1a** (0.20 mmol), base (0.20 mmol), and/or additive in 2.0 mL of solvent (s), 25 °C, 4 h. For reaction (**II**), Reaction conditions. **2a** (0.20 mmol), Novozym-435

(CALB) (40.0 mg, 20 mg/0.1 mmol), and acetic anhydride (0.40 mmol, 2.0 equiv.) in 2.0 mL of toluene, 25 °C, 36 h. For reaction (**III**) by using a 0.40 mm-thick PDMS membrane; In Compartment-**A**, **1a** (0.20 mmol), base (0.20 mmol), and/or additive in 2.0 mL of solvent (s), 25 °C, 4 h. In compartment-**B**, Novozym-435 (CALB) (40.0 mg, 20 mg/0.1 mmol), and acetic anhydride (0.40 mmol, 2.0 equiv.) in 2.0 mL of toluene, 25 °C, 36 h. [b] Yields were determined by ^{1}H–NMR analysis in Compartment-**B**, and the %*ee* values were determined by chiral HPLC analysis. [c] Data were obtained using 1.50 equivalents of DBU in Compartment-**A**. [d] Data were obtained using 0.50 equivalents of DBU in Compartment-**A.** [e] Data were obtained using a 0.20 mm thick PDMS membrane. [f] Data were obtained using a 0.60 mm thick PDMS membrane."

To:

"

Entry	Type	Catalyst(s)/solvent(s)	%Yield of (*R*)-**3a**[b]	%*ee*) of (*R*)-**3a**[b]
1	**I**	CALB/toluene	48	99
2	**I**	CALB/toluene + DBU	68	3
3	**I**	CALB/toluene + pyridine	59	27
4	**I**	CALB/toluene + piperidine	61	13
5	**I**	CALB/toluene + Et_3N	53	21
6	**I**	CALB/toluene + $NaHCO_3$	55	46
7	**I**	CALB/toluene + K_2CO_3	58	45
8	**II**	DBU/toluene + CALB/toluene	91	99
9 [c]	**II**	NEt_3/toluene + PS-IM/toluene	88	99
10 [d]	**II**	DBU/toluene + CALB/toluene	85	99
11 [e]	**II**	DBU/toluene + CALB/toluene	81	99
12 [f]	**II**	DBU/toluene + CALB/toluene	83	99
13 [g]	**II**	DBU/toluene + CALB/toluene	87	99

[a] Reaction conditions: For reaction (**I**), **1a** (0.20 mmol), base (0.20 mmol), and/or additive in 2.0 mL of solvent (s), 25 °C, 4 h. For reaction (**II**) by using a 0.40 mm-thick PDMS membrane; In Compartment-**A**, **1a** (0.20 mmol), base (0.20 mmol), and/or additive in 2.0 mL of solvent (s), 25 °C, 4 h. In compartment-**B**, Novozym-435 (CALB) (40.0 mg, 20 mg/0.1 mmol), and acetic anhydride (0.40 mmol, 2.0 equiv.) in 2.0 mL of toluene, 25 °C, 36 h. [b] Yields were determined by ^{1}H–NMR analysis in Compartment-**B**, and the %*ee* values were determined by chiral HPLC analysis. [c] Data were obtained using 1.0 equivalents of NEt_3 instead of DBU in Compartment-**A** and PS-IM instead of CALB in Compartment-**B**. [d] Data were obtained using 1.50 equivalents of DBU in Compartment-**A**. [e] Data were obtained using 0.50 equivalents of DBU in Compartment-**A.** [f] Data were obtained using a 0.20 mm thick PDMS membrane. [g] Data were obtained using a 0.60 mm thick PDMS membrane."

Question 4:

Process has been scaled up to 4.0 mmol, which for this type of enzymes is not so high, but no studies on the PDMS stability and reusability for different cycles have been performed.

Response: According to the reviewer's suggestion, the stability and reusability of the PDMS membrane were investigated. It was found that the PDMS membrane could be used for five cycles. By comparing SEM images taken after the first and fifth cycles, it was observed that their morphology showed no significant changes, thereby demonstrating good stability and reusability. The statements in the manuscript have been revised. Also, the reusability of the PDMS membrane and the SEM image of the PDMS membrane before and after the fifth cycle have been placed in the Supporting Information as follows.

The statement has been revised in the revised manuscript as follows.

Revise (page 20, line 11): **"**It was observed that the DKR of **2a** (4.0 mmol) delivered 1.15 g of the optically pure talosalate (*R*)-**3k** in 92% yield with 99% ee upon extended reaction time (Equation 2 in Scheme 4)**"**

To: **"**It was observed that the DKR of **2a** (4.0 mmol) delivered 1.15 g of the optically pure talosalate (*R*)-**3k** in 92% yield with 99% ee upon extended reaction time (Equation 2 in Scheme 4). Furthermore, it was also found that the PDMS membrane could be reused five times while still maintaining 81% yield and 97% *ee*, as confirmed by the SEM images (see Supplementary Experimental, Table S4 and Fig. S10, SI).**"**

Also, the reusability of the PDMS membrane had been provided in the Supporting Information as Table S4 as follows.

Table S4. Reusability of the PDMS membrane (for the PDMS-modulated DKR process).

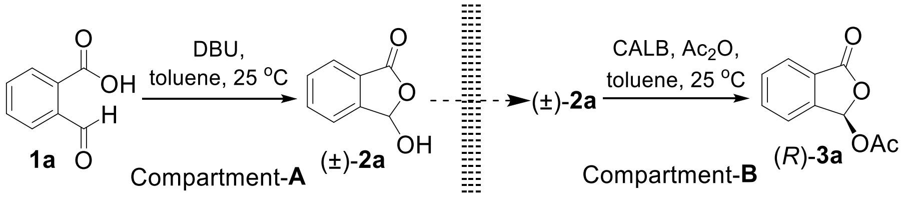

Entry	1	2	3	4	5	6
%Yield	90	88	86	83	81	68
%*ee*	99	99	98	98	97	95

Reaction conditions (The PDMS-modulated DKR process utilizes a 0.40 mm-thick

PDMS membrane for its repeatability testing): In Compartment-**A**, **1a** (0.20 mmol), and DBU (0.20 mmol) in 2.0 mL of toluene, 25 °C, 36 h. In compartment-**B**, Novozym-435 (CALB) (40.0 mg, 20 mg/0.1 mmol), and acetic anhydride (0.40 mmol, 2.0 equiv.) in 2.0 mL of toluene, 25 °C, 36 h. [b] Yields were determined by ^{1}H–NMR analysis in Compartment-**B**, and the %*ee* values were determined by chiral HPLC analysis.

In addition, the general procedure for evaluating the reusability of the PDMS membrane had been provided in the experimental section of the Supporting Information as follows.

7. General procedure for the reusability of the PDMS membrane.

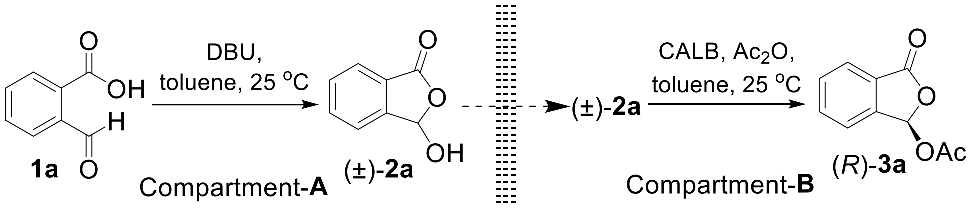

A typical procedure was as follows. In compartment-**A**, **1a** (0.20 mmol) and DBU (0.20 mmol) were introduced into 2.0 mL of toluene. In compartment-**B**, Novozym-435 (CALB) (40.0 mg, 20 mg/0.1 mmol) and acetic anhydride (0.40 mmol, 2.0 equiv.) were introduced into 2.0 mL of a mixture of toluene. A balloon filled with air (P = 1 atm) was connected to the top of both compartments. Both reactions in each compartment were stirred at 25 °C for 36 h. After this, the resulting solution in both compartments was removed, and the membrane in the reaction device was reused for the next cycle. The resulting solution was filtered, and toluene was removed using a rotary evaporator. The remaining solution was extracted with EtOAc (3 × 3 mL), and the collected organic phase was rinsed with brine (5 mL). After drying the organic layer over anhydrous Na_2SO_4, filtration of the drying agent, and evaporation of the solvent under vacuum, the crude product was purified by silica gel flash column chromatography, affording (*R*)-**3a**. The enantiomeric excess (*ee*) values were determined using HPLC analysis with a Photo-Diode Array detector and a Daicel chiral cell column (Φ 0.46 × 25 cm).

In addition, the SEM image of the PDMS membrane before and after the fifth and sixth cycle has been provided in the Supporting Information as Figure S10 as follows.

Figure S10. The SEM image of the PDMS membrane before and after the fifth and sixth cycles.

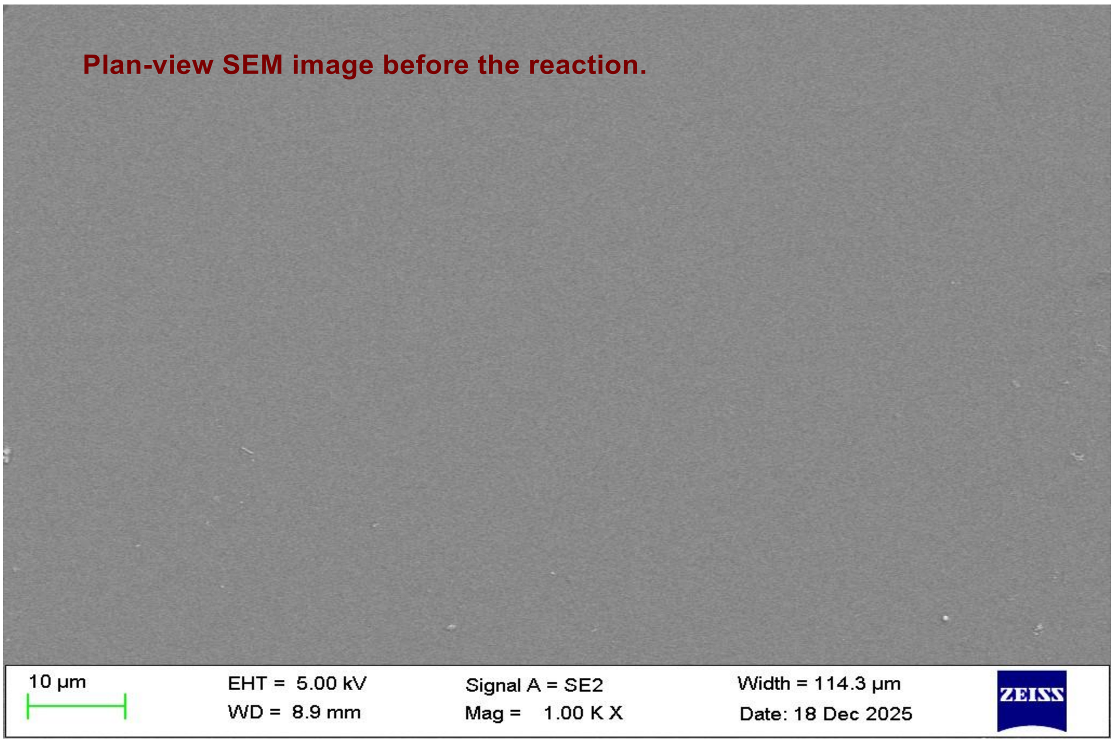

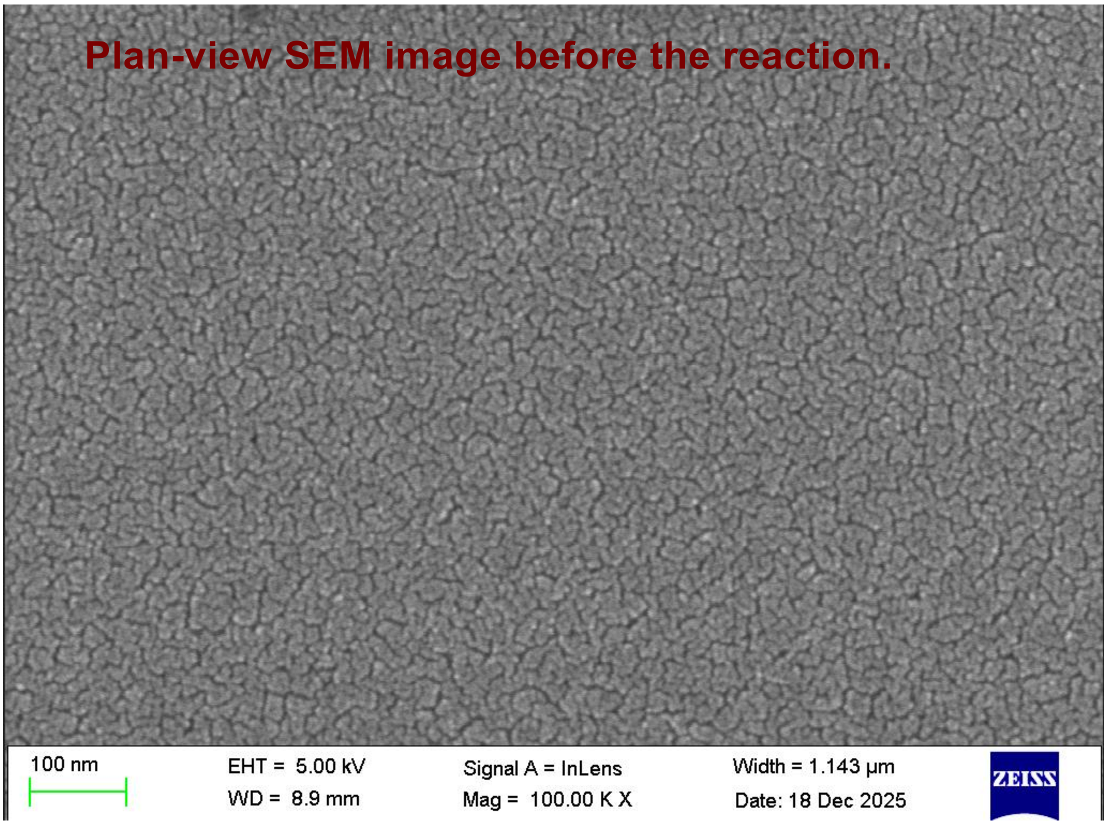

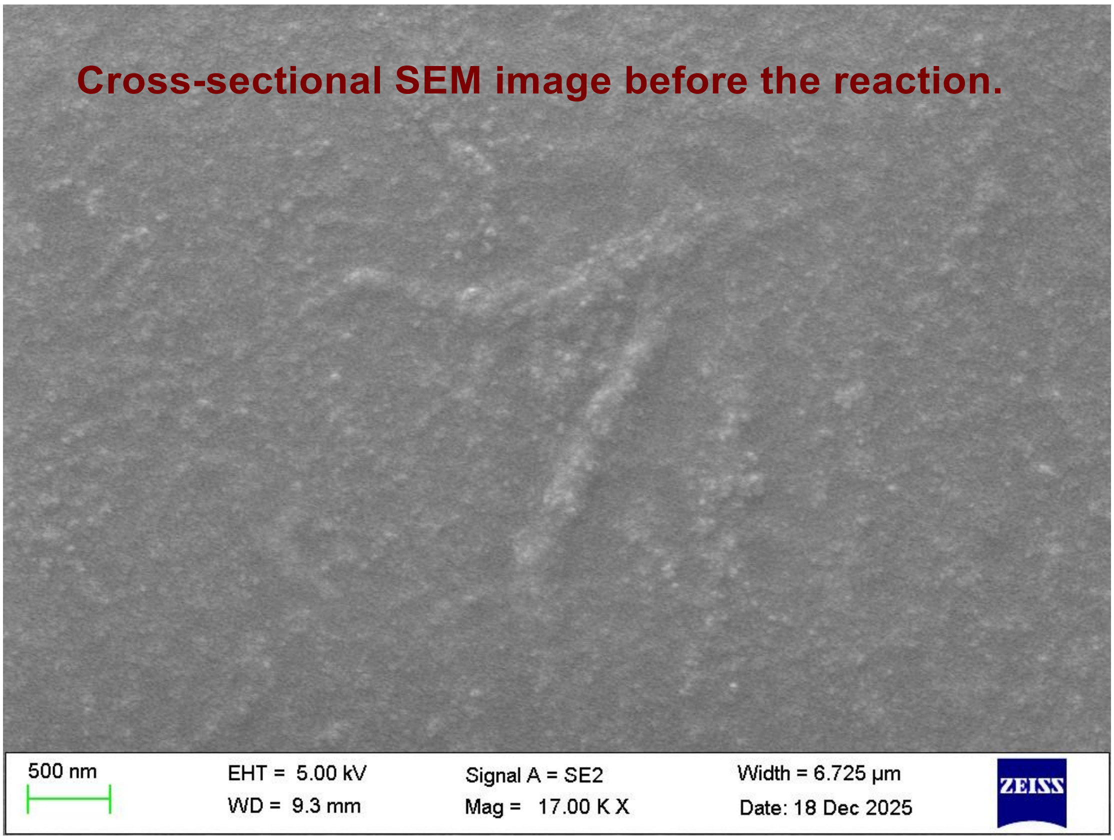
Cross-sectional SEM image before the reaction.
500 nm
EHT = 5.00 kV
WD = 9.3 mm
Signal A = SE2
Mag = 17.00 K X
Width = 6.725 µm
Date: 18 Dec 2025
ZEISS

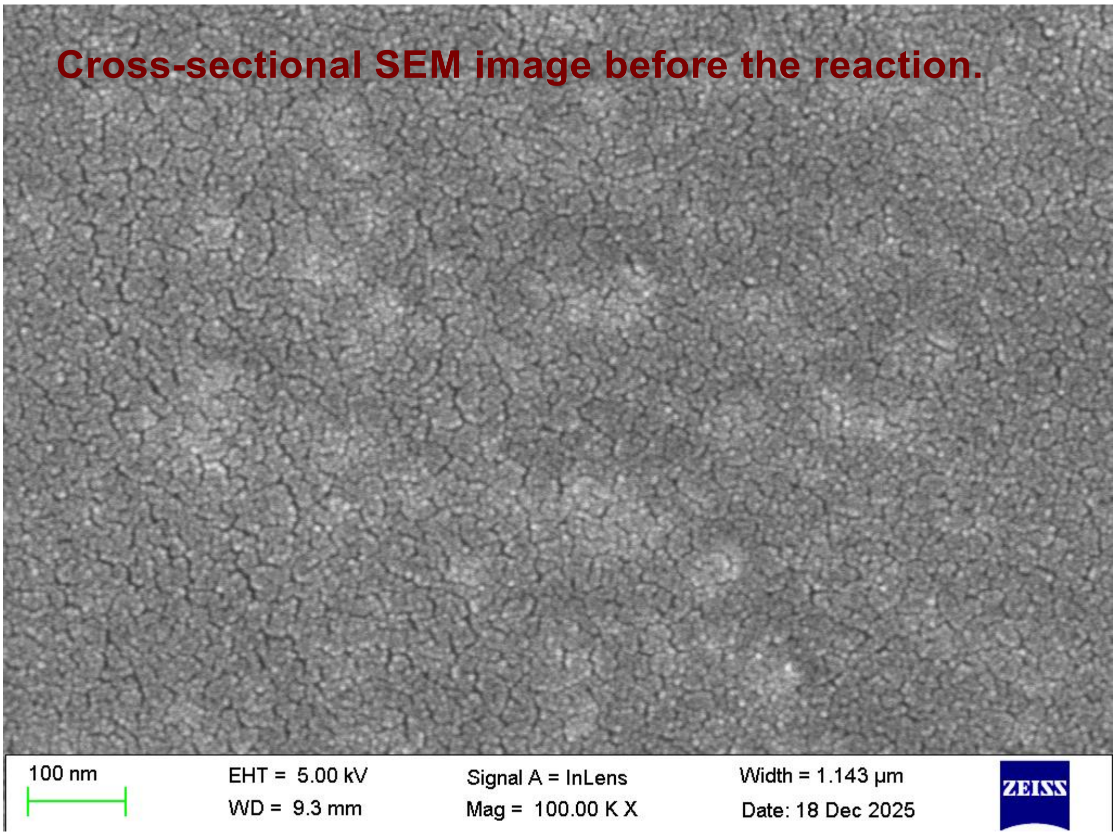
Cross-sectional SEM image before the reaction.
100 nm
EHT = 5.00 kV
WD = 9.3 mm
Signal A = InLens
Mag = 100.00 K X
Width = 1.143 µm
Date: 18 Dec 2025
ZEISS

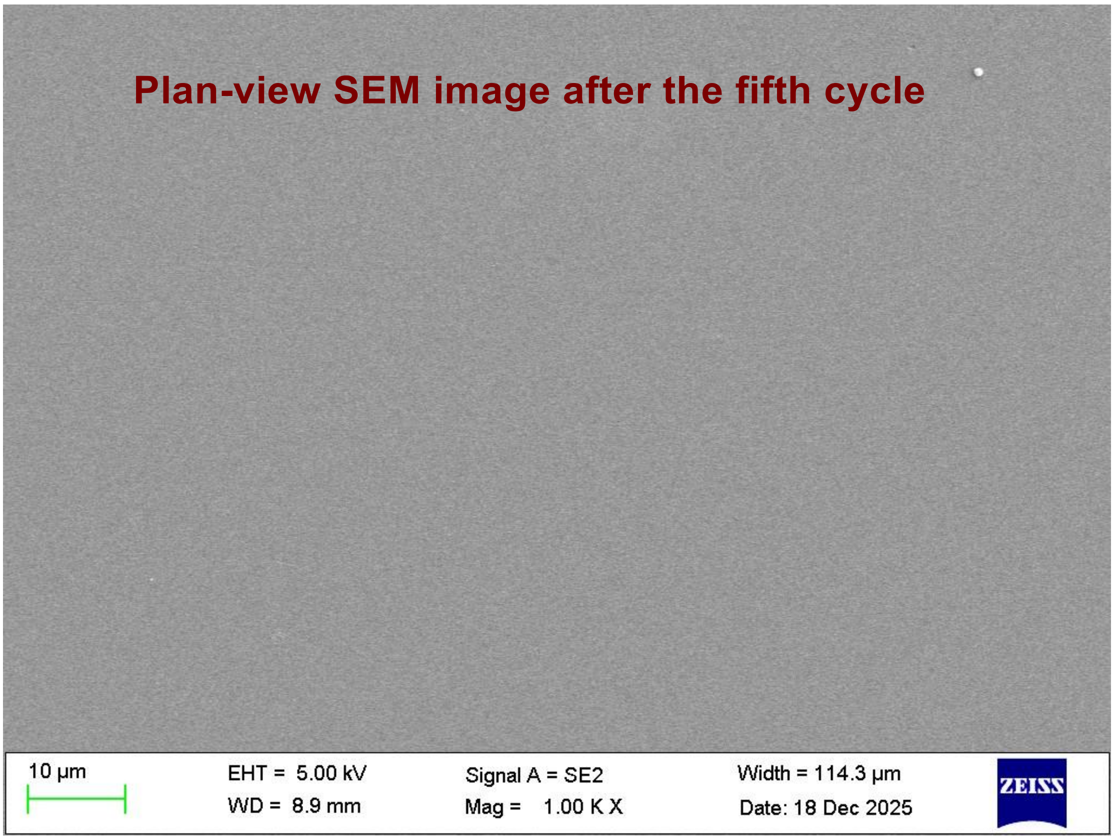
Plan-view SEM image after the fifth cycle
10 µm
EHT = 5.00 kV
WD = 8.9 mm
Signal A = SE2
Mag = 1.00 K X
Width = 114.3 µm
Date: 18 Dec 2025
ZEISS

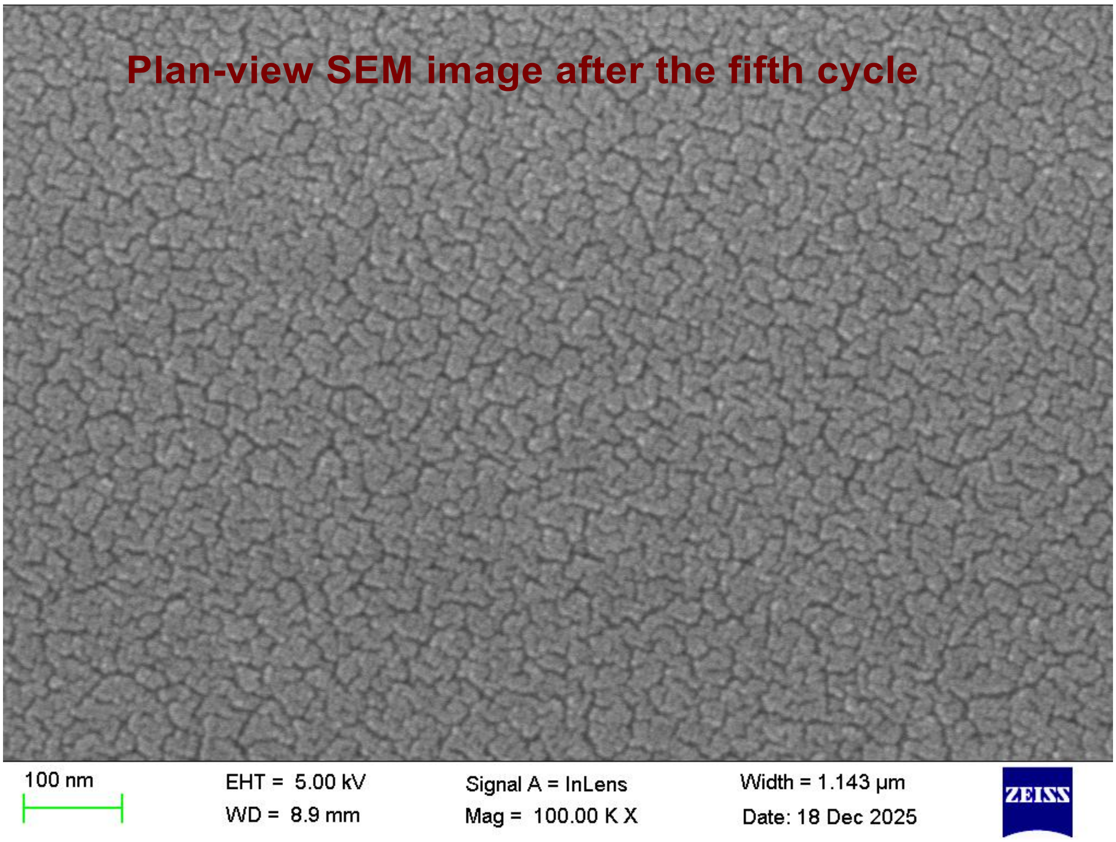
Plan-view SEM image after the fifth cycle
100 nm
EHT = 5.00 kV
WD = 8.9 mm
Signal A = InLens
Mag = 100.00 K X
Width = 1.143 µm
Date: 18 Dec 2025
ZEISS

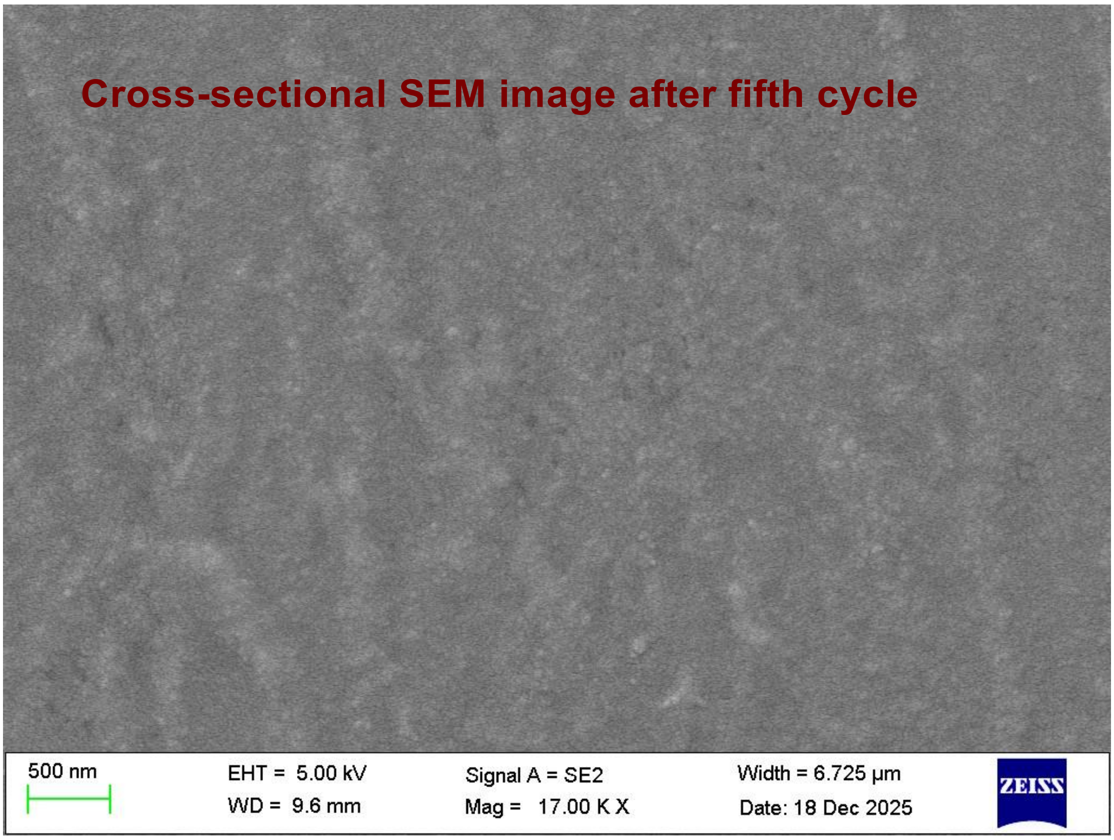
Cross-sectional SEM image after fifth cycle
500 nm
EHT = 5.00 kV
WD = 9.6 mm
Signal A = SE2
Mag = 17.00 K X
Width = 6.725 µm
Date: 18 Dec 2025

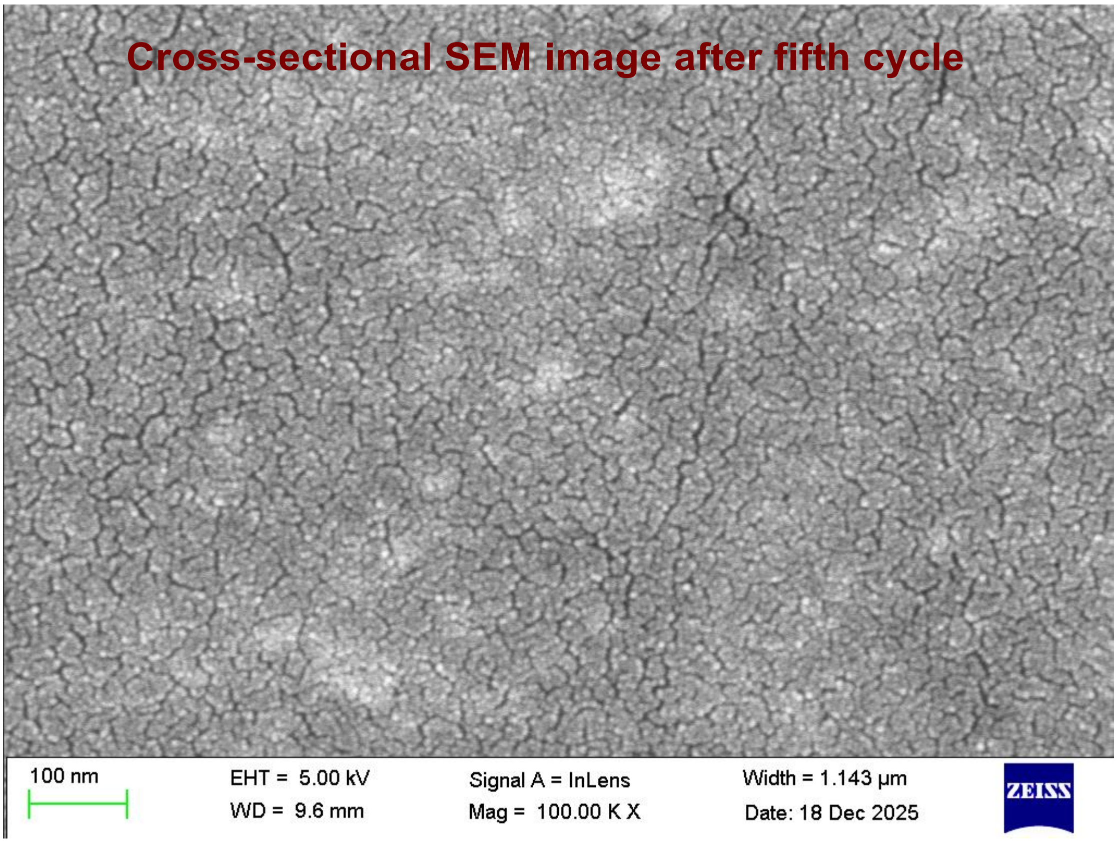
Cross-sectional SEM image after fifth cycle
100 nm
EHT = 5.00 kV
WD = 9.6 mm
Signal A = InLens
Mag = 100.00 K X
Width = 1.143 µm
Date: 18 Dec 2025

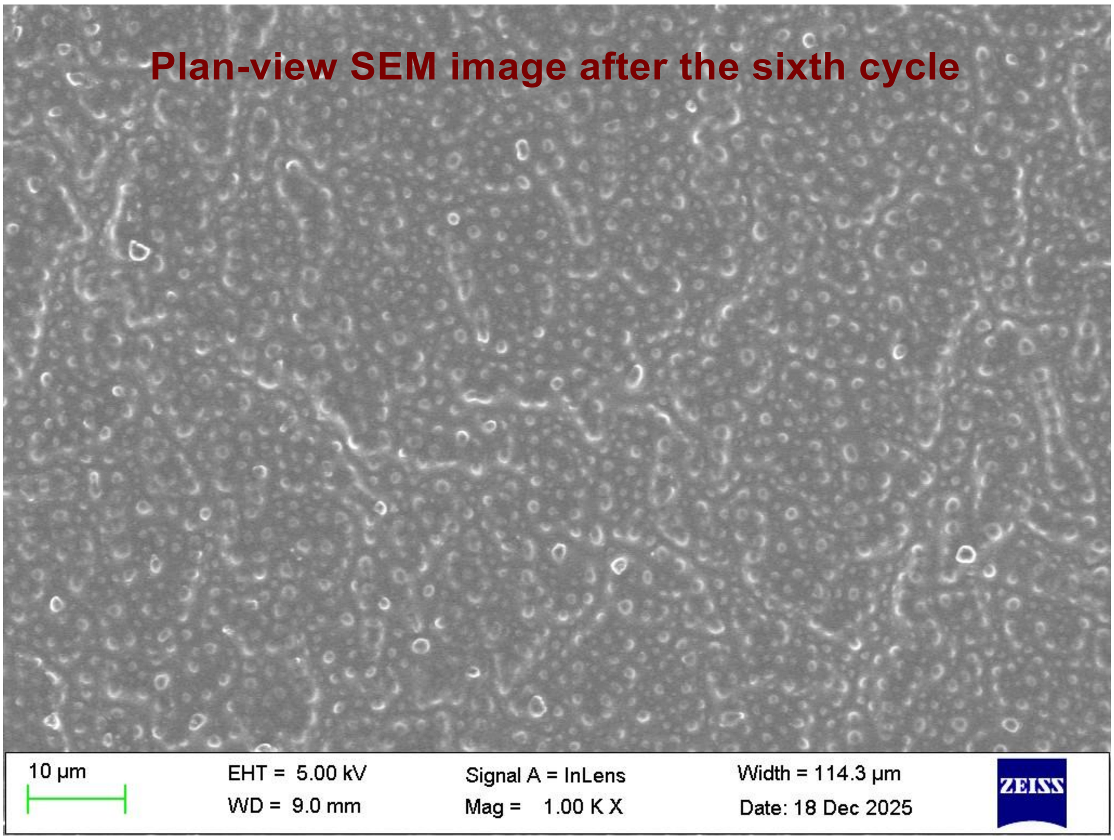
Plan-view SEM image after the sixth cycle
10 µm
EHT = 5.00 kV
WD = 9.0 mm
Signal A = InLens
Mag = 1.00 K X
Width = 114.3 µm
Date: 18 Dec 2025
ZEISS

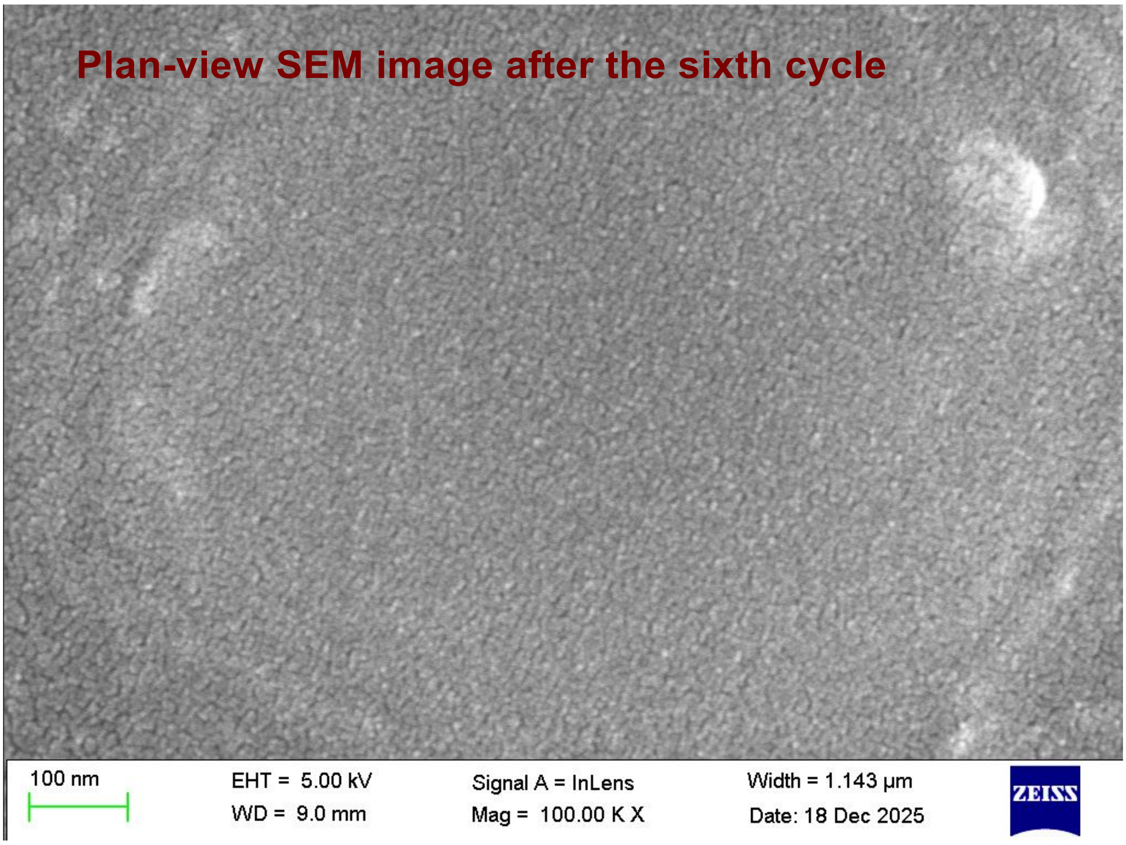
Plan-view SEM image after the sixth cycle
100 nm
EHT = 5.00 kV
WD = 9.0 mm
Signal A = InLens
Mag = 100.00 K X
Width = 1.143 µm
Date: 18 Dec 2025
ZEISS

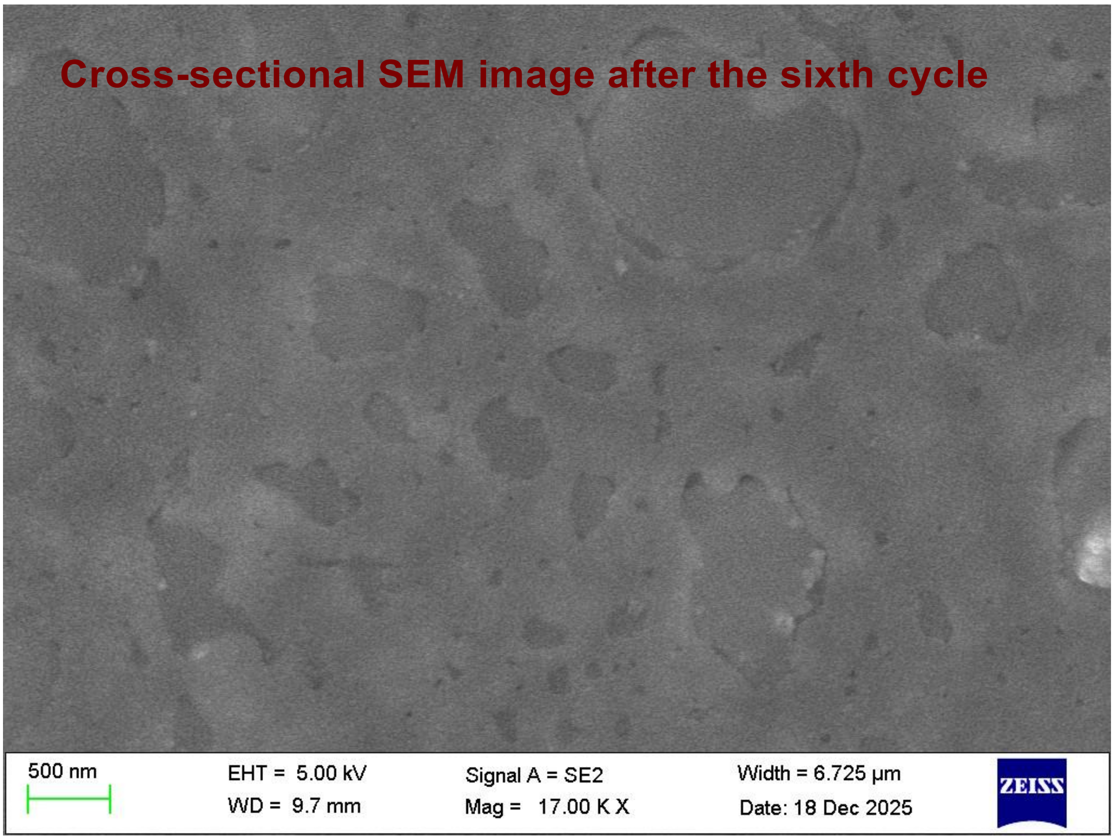
Cross-sectional SEM image after the sixth cycle
500 nm
EHT = 5.00 kV
WD = 9.7 mm
Signal A = SE2
Mag = 17.00 K X
Width = 6.725 µm
Date: 18 Dec 2025
ZEISS

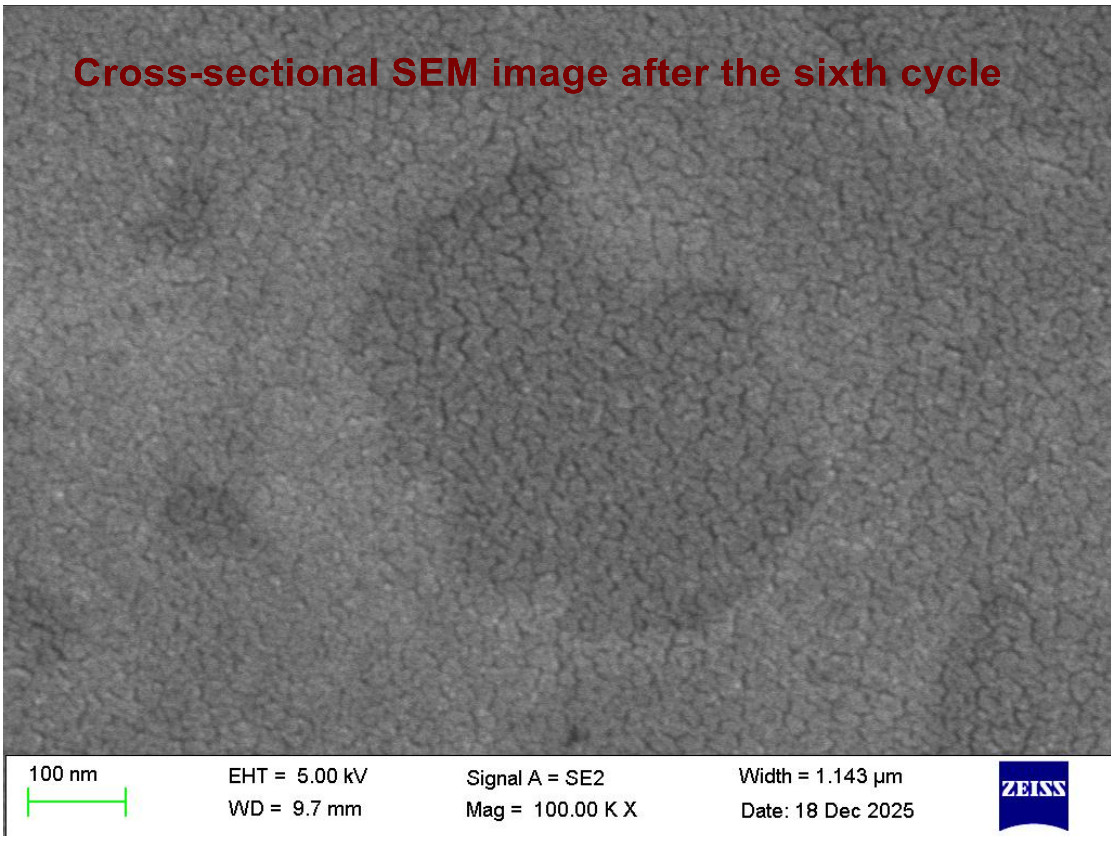
Cross-sectional SEM image after the sixth cycle
100 nm
EHT = 5.00 kV
WD = 9.7 mm
Signal A = SE2
Mag = 100.00 K X
Width = 1.143 µm
Date: 18 Dec 2025
ZEISS

Question 5:

The use of DBU in stoichiometric amounts can present some enviormental issues. In addition, the use of this base has shown certain side-reactions, have the authors observed any other product in the reaction medium?

Response: The reviewer is right that the use of stoichiometric amounts of DBU may raise certain environmental concerns. Fortunately, in our system, apart from generating trace amounts of racemized products (please refer to entry 8 in Table 1 of the manuscript), no other byproducts were observed.

Question 6:

Finally, I am also missing some studies that are basic for the manuscript: Authors have not measured the diffusion rates through PDMS; There is no experimental calculation of the DBU-racemization kinetic and finally, there are no results on the retention of the DBU base in its compartment. Have been base-transfer observed?

Response: According to the suggestion, we have measured the diffusion rate of intermediate **2a** through PDMS. It was found that the diffusion rate of intermediate **2a** through PDMS is 6.35×10^{-12} m^2/s. Furthermore, the statement has been revised, the procedure for measuring the diffusion rate of intermediate **2a** through PDMS has been incorporated into the Supporting Information, and the references have been cited, as detailed below.

The statement in the manuscript has been revised as follows.

Revise (page 13, line 5): **"**In the second resolution stage, **2** diffuses through the PDMS membrane into Compartment-**B**, where the CALB-mediated selective esterification of (*R*)-**2** to give (*R*)-**3**, leaving (*S*)-**2** (blue-labeled) to accumulate.**"**

To: **"**In the second resolution stage, **2** diffuses through the PDMS membrane into Compartment-**B**, with a diffusion rate of 6.35×10^{-12} m^2/s determined *via* the controlled experiments (see Supplementary Experimental and Figs. S2, SI).[52-53] Concurrently, the CALB-mediated selective esterification of (*R*)-**2** occurs in Compartment-**B**, yielding the target product (*R*)-**3**, while the unreacted intermediate (*S*)-**2** (blue-labeled) accumulates.**"**

Also, the measurement procedure for the diffusion rate of **2a** through PDMS incorporated into the Supporting Information as Figure S2 was as follows.

Add Figure S2 in Supporting Information:

"Figure S2. Measurement of the diffusion coefficient of 2a through PDMS.

1.1 Measurement principle of the diffusion rate.[4-5]

The Time-Lag Method is a classical technique for determining the diffusion coefficient (D) by analyzing the transient diffusion data of a substance permeating through a thin

film. The theoretical basis of this method lies in the fact that the diffusion of a substance through a film requires a transition period from initiation to the establishment of a steady state. This transition time is directly related to the diffusion coefficient and the film thickness. The formula for calculating the diffusion coefficient (D) is:

$D = L^2 / 6\theta$

Where:

- D is the diffusion coefficient (m^2/s).
- L is the film thickness (m).
- θ is the time-lag, which is the time required from the start of diffusion until the steady-state diffusion stage is reached (s).

1.2 Determination of the diffusion rate of the intermediate **2a**

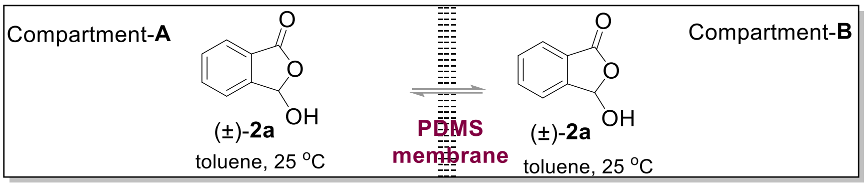

Objective: To quantify the diffusion coefficient (D) of the key intermediate compound **2a** through a defined PDMS, providing essential kinetic parameters for understanding its mass transport behavior.

Step 1: Conduct the diffusion experiment and collect data

The experimental setup referenced from the article is used. A PDMS membrane is securely fixed between the two compartments (Compartment-**A:** 2.0 mL of toluene containing **2a** (0.20 mmol, 30.0 mg), and Compartment-**B:** 2.0 mL of pure toluene). The experiment is conducted under constant temperature (25°C) with stirring at a fixed speed of 150 rpm to minimize boundary layer effects. At specific time intervals, a 10 μL sample is withdrawn from Compartment-**B** and analyzed by Gas Chromatography (GC/MS) to quantify the mass of **2a** that has diffused across the membrane. The collected data were summarized in a table as shown below.

**Time (h)**	**Mass of 2a (mg) in Compartment-B**
0	0.0
20	0.0
40	0.0
60	0.0
70	0.0
75	0.1
80	0.43

100	0.81
120	1.34
140	1.78
160	2.33
180	2.93
200	3.41
220	3.94
240	4.50

Step 2: Plot the diffusion profile

Figure. Mass of **2a** diffused to the Compartment-**B** versus time.

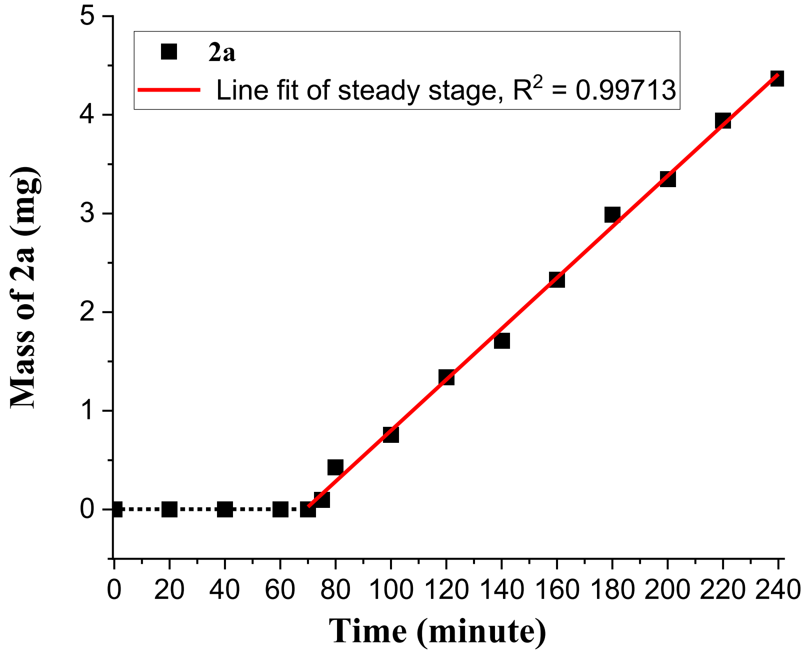

Step 3: Determine the time-lag (θ) from the Figure

The plotted curve typically shows two phases. (1) Initial Transient Phase: As the compound penetrates and diffuses through the membrane, the mass in the receptor compartment is very low. The curve is initially flat and close to the time axis. (2) Steady-State Phase**:** Diffusion reaches a steady state, and the mass in the receptor compartment increases linearly with time. The linear portion of the curve from the steady-state phase is extrapolated backwards until it intersects the time axis. The value of time at this intersection point is defined as the Time-Lag (θ) (defined as the time required from the start of diffusion until the steady-state diffusion stage is reached).

For the curve corresponding to compound **2a** (above Figure), the time-lag (θ) is estimated to be **70** minutes (1.17 h).

Step 4: Calculate the diffusion coefficient (D)

The obtained time-lag (θ) and the known membrane thickness (L) are substituted directly into the time-lag equation:

$D = L^2 / 6\theta$

Sample 2a Calculation:

- $L = 400\ \mu m = 400 \times 10^{-6}\ m = 4 \times 10^{-4}\ m$
- $\theta = 70\ (minute) \times 60 = 4200\ s$

$D = (4 \times 10^{-4}\ m)^2 / (6 \times 4200\ s) = (1.6 \times 10^{-7}\ m^2) / (25200\ s) \approx 6.35 \times 10^{-12}\ m^2/s$. ”

Also, the references have been cited in the manuscript as ref. 52 and 53, as follows

Add reference: “52. An, D., Yang, L., Liu, B., Wang, T. & Kan, C. Diffusion performance of fertilizer nutrient through polymer latex Film, *J. Agric. Food Chem.* **65**, 10868-10874 (2017).

53. Yang, L., An, D., Wang, T., Kan, C. & Jin, Y. Swelling and diffusion model of a hydrophilic film coating on controlled-release urea particles, *Particuology* **30**, 73–82 (2017).”

Regarding the concern that " There is no experimental calculation of the DBU-racemization kinetics", we had also investigated the experimental calculation of the DBU-racemization kinetics. Due to the inherent instability of series **2a** compounds (attributed to dynamic reversible ring-opening/closing tautomerism), it is difficult to effectively and accurately determine the content of the two enantiomeric peaks under the attempted chiral HPLC conditions. Therefore, we used (*S*)-**2x** as an example to explore the DBU-mediated racemization kinetics. It was found that the rate constant and half-life for the DBU-mediated racemization were determined to be 0.707 h^{-1} and 0.980 h, respectively, which were comparable to the reported results.[54-56] Also, the statement has been revised, the procedure for the experimental calculation of the DBU-racemization kinetics of (*S*)-**2x** has been incorporated into the Supporting Information, and the references have been cited, as detailed below.

The statement in the manuscript has been revised as follows.

Revise (page 14, line 2): **“**In this process, the DBU-mediated racemization proceeds through a reversible ring-opening/closing mechanism, regenerating racemic **2** for subsequent CALB resolution in Compartment-**B**.”

To: **“**In this process, the DBU-mediated racemization proceeds through a reversible ring-opening/closing mechanism, regenerating racemic **2** for subsequent CALB resolution in Compartment-**B**. Controlled comparative kinetic tests of racemization in Compartment-**A** revealed that the DBU significantly influenced the racemization process. The corresponding rate constant and half-life for the DBU-mediated racemization were determined to be 0.707 h^{-1} and 0.980 h, respectively, calculated using a reported method (see Supplementary Experimental and Figs. S3, SI).[54-56]**”**

Also, the procedure for the experimental calculation of the DBU-racemization kinetics of (*S*)-**2x** had been incorporated into the Supporting Information as Figure S3, as follows.

Figure S3. Measurement of the DBU-racemization kinetic parameters.

1.1 Reaction modeling principle. [6-8]

The DBU-catalyzed racemization is modeled as a first-order irreversible reaction: (*S*) → Racemate. The kinetic analysis relies on monitoring the decrease in enantiomeric excess (*ee*) over time. Its kinetic parameters are solved using the following mathematical model:

(1) Calculation of enantiomeric excess (ee%):

The concentrations of the R- and S-enantiomers are determined separately using HPLC. The ee% is calculated using the formula:

$ee\% = (([R]-[S])/([R]+[S])) \times 100$

(2) Determination of rate constant (k_{rac}) and half-life ($t_{1/2rac}$):

A plot of ln(ee) versus time is constructed. The slope of this linear plot gives the racemization rate constant, k_{rac}. The half-life of racemization is then calculated from the rate constant using the equation:

$t_{1/2rac} = (\ln 2)/k_{rac}$

1.2 Determination of DBU-induced racemization kinetic parameters.

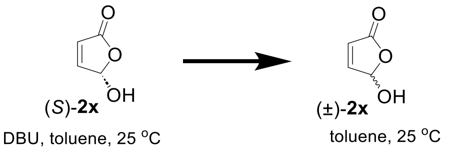

Objective: To quantitatively determine the kinetic parameters (rate constant k_{rac} and half-life $t_{1/2rac}$) of the DBU-catalyzed racemization using compound **2x** as a model substrate, establishing a standard protocol for evaluating chiral stability under basic conditions.

Step 1: Reaction system configuration and collect data

(1) Base: 1,8-Diazabicyclo[5.4.0]undec-7-ene (DBU)
(2) Chiral Substrate: Compound 2x (0.20 mmol, 30.0 mg)
(3) Solvent: 2.0 mL toluene
(4) Conditions: Constant temperature (25°C) with stirring at 150 rpm.

Step 2: Calculation of enantiomeric excess (ee%)

(1) Solution preparation: Precisely weigh and dissolve 0.20 mmol of compound 2x in 2.0 mL of toluene.

(2) Reaction initiation: Add an equimolar amount of the base DBU (1,8-diazabicyclo[5.4.0]undec-7-ene) to the solution to initiate racemization. Start the timer immediately and maintain a constant stirring speed.

(3) Sampling and analysis: At specific, predetermined time intervals, withdraw a 100 μL aliquot from the reaction mixture.

(4) HPLC analysis: Analyze each aliquot using High-Performance Liquid Chromatography (HPLC) with a chiral stationary phase to separate and quantify the R- and S-enantiomers of 2x.

(5) Calculation: For each time point, calculate the enantiomeric excess (ee%) using the formula based on the concentrations ([R] and [S]) determined by HPLC:

a) ee% = [([R] - [S]) / ([R] + [S])] × 100

The collected data were summarized in a table as shown below.

**Time (h)**	***%ee* of 2x**
0.0	98
0.5	83
1.0	56
2.0	28
3.0	12
4.0	8

(*S*)-3x: (*S*)-5-hydroxyfuran-2(5H)-one (HPLC: Chiralpak OJ-H, detected at 210 nm, eluent: n-hexane/2-propanol = 95/5, flow rate = 1.0 mL/min, 25°C).

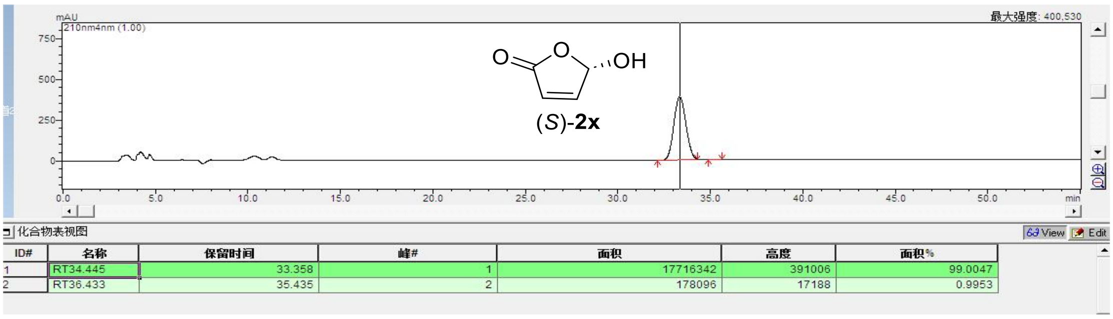

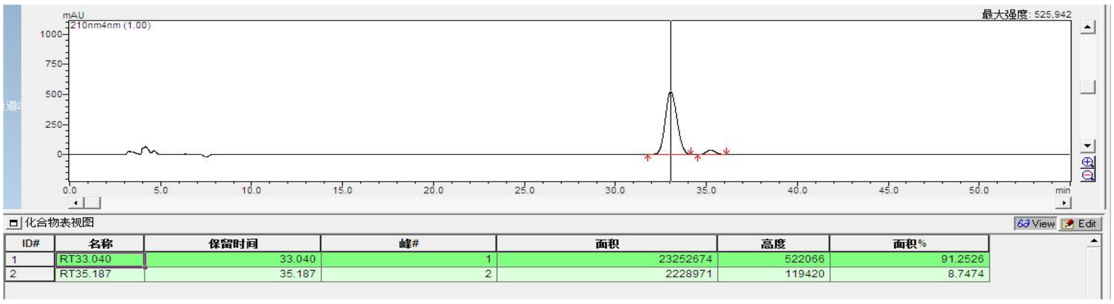

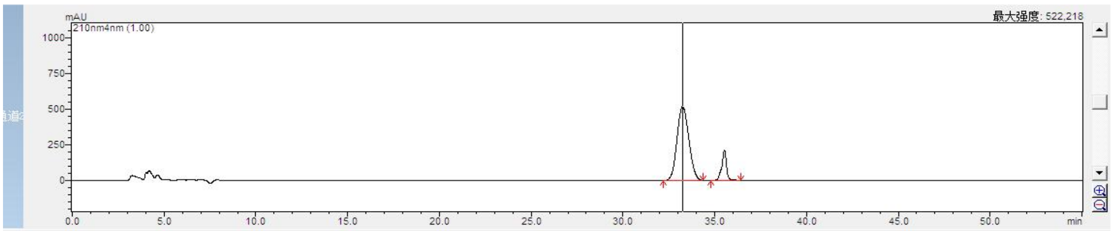

化合物表视图

ID#	名称	保留时间	峰#	面积	高度	面积%
1	RT33.242	33.242	1	23193000	517645	78.0533
2	RT35.370	35.370	2	6521304	234355	21.9467

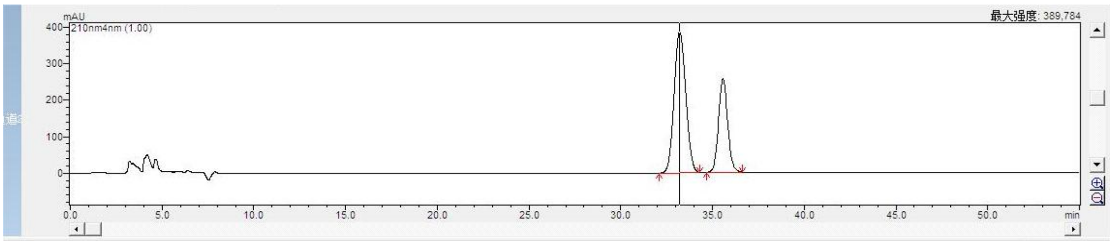

化合物表视图

ID#	名称	保留时间	峰#	面积	高度	面积%
1	RT33.209	33.209	1	17079772	386695	64.3601
2	RT35.357	35.357	2	9458038	264278	35.6399

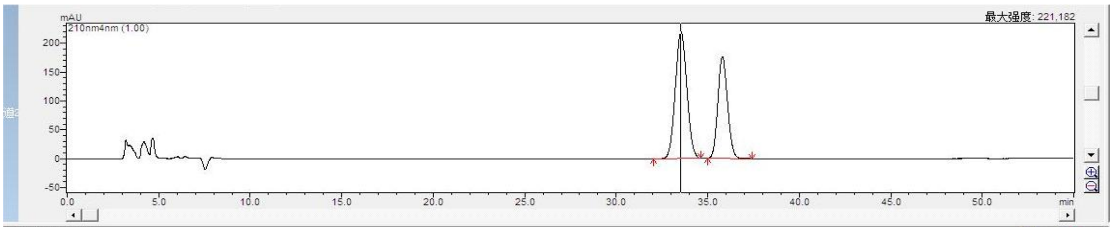

化合物表视图

ID#	名称	保留时间	峰#	面积	高度	面积%
1	RT33.533	33.533	1	9671812	219547	56.0636
2	RT35.671	35.671	2	7579683	181736	43.9364

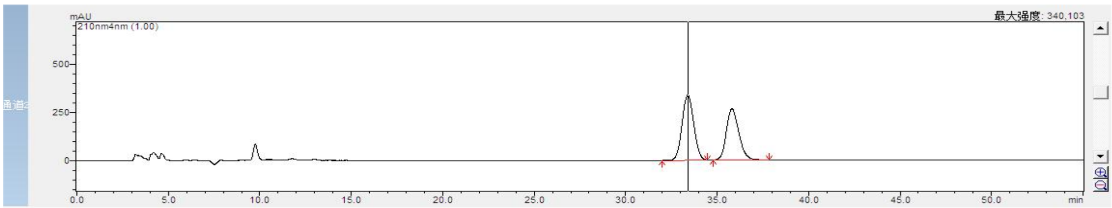

化合物表视图

ID#	名称	保留时间	峰#	面积	高度	面积%
1	RT33.389	33.389	1	14924001	336473	54.1481
2	RT35.523	35.523	2	12637466	289903	45.8519

Step 3: Determination of the racemization rate constant (k_{rac})

(1) Data transformation: Calculate the natural logarithm (ln) of the ee% values obtained in Step 2 for each corresponding time point.

(2) Plot generation: Construct a graph of ln(ee) versus time.

(3) Linear regression: Perform a linear fit on the data points. The racemization is modeled as a first-order reaction, and the data should fit the equation:

$$\ln(ee) = -k_{rac} \times t$$

where t is time, and k_{rac} is a rate constant.

(4) Slope analysis: The slope of the resulting straight line is equal to the negative value of the racemization rate constant, $-k_{rac}$.

Figure. Influence of DBU on the rate constant of the racemization of **2x** at 25 °C, with time on the x-axis and ln(ee) on the y-axis.

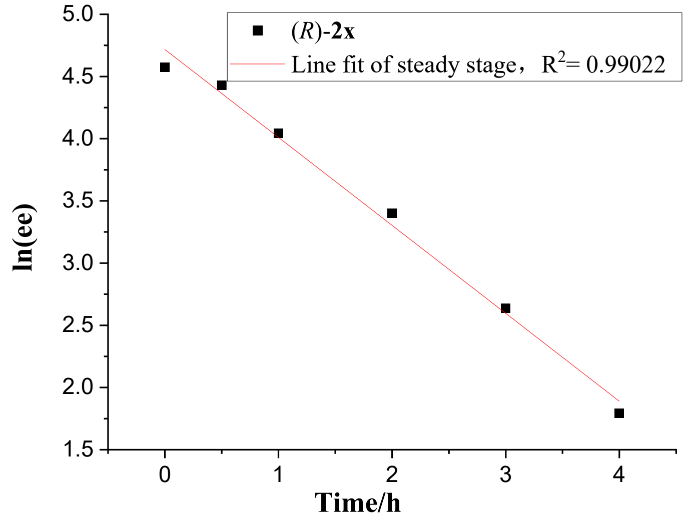

For the rate constant corresponding to compound **2x** (above Figure), the $\boldsymbol{k}_{\mathbf{rac}}$ is estimated to be 0.70703 (h^{-1}).

Step 4: Calculation of the half-life ($t_{1/2\text{rac}}$)

(1) Parameter application: Once the racemization rate constant (k_{rac}) is determined from the slope in Step 3, it is used in the standard first-order kinetics half-life formula.

(2) Half-Life calculation: Calculate the half-life of racemization, which is the time required for the enantiomeric excess (ee%) to decrease to 50% of its initial value, using the equation:

$t_{1/2\text{rac}} = \ln 2/k_{\text{rac}}$

Sample calculation:

- $k_{\text{rac}} = 0.70703\ h^{-1}$

$t_{1/2\ \text{rac}} = \ln 2/k_{\text{rac}} = 0.693147/0.70703\ h^{-1} \approx 0.980\ h$

Also, the references have been cited in the manuscript as ref. 54-56, as follows

Add reference: "54. Cabordery, A., Toussaint, M., Azaroual, N., Bonte, J., Melnyk, P., Vaccher, C. & Foulon, C. Kinetics and mechanism of racemization of Tic-hydantoins, potent sigma-1 agonists, *Tetrahedron: Asymmetry* **22**, 125–133 (2011).

55. Danel, C., Azaroual, N., Brunel, A., Lannoy, D., Odou, P., Décaudin, B., Vermeersch, G., Bonte, J. & Vaccher, C. Configurational stability of 9-hydroxyrisperidone. Kinetics and mechanism of racemization, *Tetrahedron: Asymmetry* **20**, 1125–1131 (2009). "

56. McClelland, R. A. & Sørensen, P. E. Kinetics of the equilibrationof 3-hydroxyphthalide and o-formylbenzoic acid. Hemiacetal breakdown with a carboxylic acid leaving group, *Can. J. Chem*. **64**, 1196-1200 (1986).

Regarding the question of "there are no results on the retention of the DBU base in its compartment. Have been base-transfer observed?", we have also conducted corresponding control experiments. These experiments provided evidence for the retention of the DBU base in its Compartment-**A** and investigated the potential transmembrane diffusion of DBU. Please refer to the response to the following Question 7.

Question 7:

Thus, the manuscript has to be improved in these ways to be suitable for publication in Nature Communications, No demonstration of DBU retention in Compartment-**A**.

Response: According to the suggestion, we investigated the transmembrane permeation of DBU from Compartment-**A** to Compartment-**B** to illustrate its retention in Compartment-**A** (Please see the response to Question 1 of reviewer 1). GC analysis revealed that only 4.8% of DBU crossed the membrane into Compartment-**B** within 36 h, meaning that less than 1.0 mol% of DBU permeated into Compartment-**B** as follows. Notably, only a trace amount of DBU in Compartment-**B** had a negligible effect on the enantioselectivity of the CALB enzyme, as proven by the control reactions (Please see Scheme 1B in the manuscript).

Also, the GC analysis of DBU has been placed in the Supporting Information as Figure S1a, as follows.

Figure S1a. The GC analysis of DBU in the control experiment before reaction at Compartment-**A** *versus* the GC analysis of DBU in the control experiment after reaction for 36 h at Compartment-**B**. (Conditions. In compartment-**A**, DBU (0.20 mmol) in 2.0 mL of toluene, 25 °C, 36 h. In compartment-**B**, 2.0 mL of toluene, 25 °C, 36 h.)

The content ratio after reaction (the peak area of DBU after reaction for 36 h divided by the peak area of DBU before the reaction**)** = 5614366/116754976 = 4.8%.

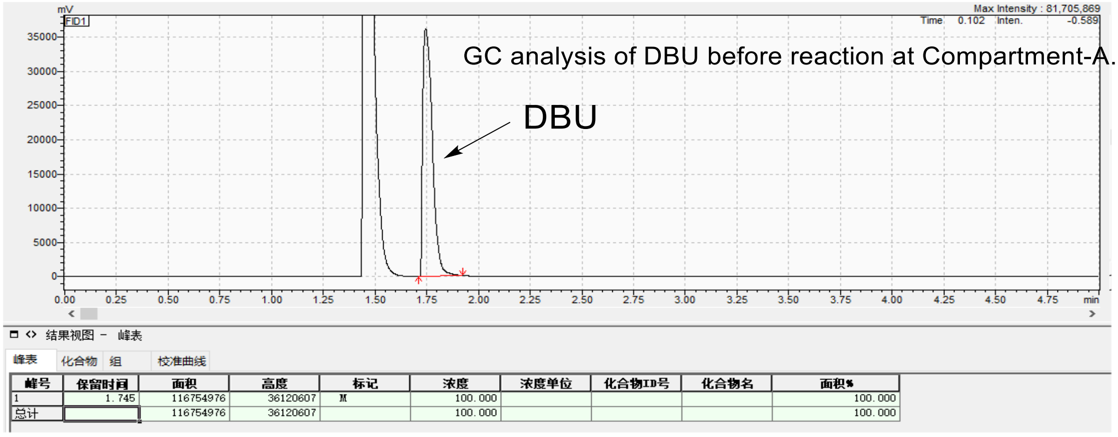

峰号	保留时间	面积	高度	标记	浓度	浓度单位	化合物ID号	化合物名	面积%
1	1.745	116754976	36120607	M	100.000				100.000
总计		116754976	36120607		100.000				100.000

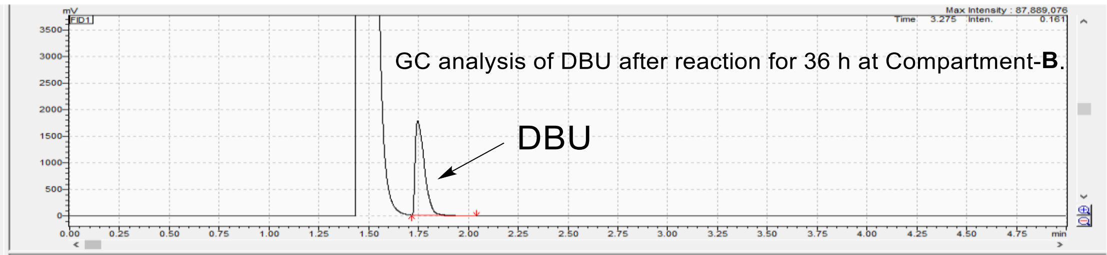

结果视图 - 峰表

峰表 化合物 组 校准曲线

峰号	保留时间	面积	高度	标记	浓度	浓度单位	化合物ID号	化合物名	面积%
1	1.746	5614366	1756202	M	100.000				100.000
总计		5614366	1756202		100.000				100.000

Also, the statement has been revised in the revised manuscript as follows.

Revise (page 8, line 9): “Finally, in the evaluation of an integrated PDMS-modulated DKR (**II**) based on the optimizations (see Supplementary Experimental and Table S2, SI), it was observed that the model reaction could steadily provide the final (*R*)-**3a** with a 91% yield and 99% *ee* (entry 8).”

To: “Finally, in the evaluation of an integrated PDMS-modulated DKR (**II**) based on the optimizations (see Supplementary Experimental and Table S2, SI), it was observed that the model reaction could steadily provide the final (*R*)-**3a** with a 91% yield and 99% *ee* (entry 8), in which the permeation of 4.8% DBU from Compartment-**A** to Compartment-**B** and 0.2% acetic anhydride from Compartment-**B** to Compartment-**A** had a negligible impact on the enantioselectivity of the product, as confirmed by GC/MS analysis (see Supplementary Figure S1, SI).

Point-by-point response:

Responses to the comment of Reviewer 2

Reviewer #2 (Remarks to the Author):

Comments:

The authors did a good job revising the manuscript. Below are some remaining points:

1. Abstract: I recommend removing the word "synchronization" in the abstract, as it was done in some parts of the manuscript text. DKR is not about synchronization; it is about compatibility. In the DKR of the compounds mentioned in the manuscript, DBU alters the enantioselectivity of CALB. The membrane separation enables compatibility between DBU and CALB because of the physical separation. This clearly shows that what the authors addressed here is not synchronization between steps but compatibility: "However, its development has long been constrained by principal limitations, which need precise synchronization of racemization and resolution steps for rate matching." Change the sentence accordingly.

2. The same issue with "synchronization" applies on page 2, line 29: "However, the fundamental challenge of a conventional chemoenzymatic DKR process lies in synchronizing the two catalytic events of racemization." Modify the sentence by removing "synchronizing." I commented on this also in the previous round of revision.

3. In the abstract: DKR is not a single-step process. It is a two-step process: racemization + acylation (in this case). If concurrent, we talk about a one-stage, two-step process. If not concurrent (as in this work, because there is physical separation via a membrane), we talk about a two-stage, two-step process. Revise the sentence in the abstract accordingly: "By spatially separating racemization and resolution while enabling their collaborative operation within a single streamlined step (….)".

4. In the abstract, there are again "grand" statements, such as: "(….) advancing chemoenzymatic DKR methodology but also provides a general and scalable platform with broad implications for pharmaceutical synthesis." This is not a "general" platform, and the implication is not so "broad." I recommend making the statement in the abstract less bold.

5. Page 8, consider the sentence: "These findings demonstrate that the designed membrane-modulated DKR strategy could achieve the anticipated coordination of DBU-mediated racemization with CALB-catalyzed resolution in a two-stage manner, thereby enabling an efficient chemoenzymatic DKR to proceed that was previously incompatible under the conventional single-step chemoenzymatic conditions." "Single-step chemoenzymatic conditions" is incorrect. It is a two-step concurrent process. For terminology in cascade processes, see for example: Schrittwieser, J. H. et al. Artificial

Biocatalytic Linear Cascades for Preparation of Organic Molecules. Chem. Rev. 118, 270–348 (2018).

Question 1:

1. Abstract: I recommend removing the word “synchronization” in the abstract, as it was done in some parts of the manuscript text. DKR is not about synchronization; it is about compatibility. In the DKR of the compounds mentioned in the manuscript, DBU alters the enantioselectivity of CALB. The membrane separation enables compatibility between DBU and CALB because of the physical separation. This clearly shows that what the authors addressed here is not synchronization between steps but compatibility: “However, its development has long been constrained by principal limitations, which need precise synchronization of racemization and resolution steps for rate matching.” Change the sentence accordingly.

Response: According to the reviewer’s suggestion, we have removed the term “synchronization” and revised the statement as follows.

Revise (page 1, abstract): “Chemoenzymatic dynamic kinetic resolution (DKR) offers a powerful bridge between chemocatalysis and biocatalysis for the preparation of chiral molecules. However, its development has long been constrained by principal limitations, which need precise synchronization of racemization and resolution steps for rate matching.”

To: “Chemoenzymatic dynamic kinetic resolution (DKR) offers a powerful bridge between chemocatalysis and biocatalysis for the preparation of chiral molecules. However, its broader application has been limited by the incompatibility between racemization and resolution catalysts, where mutual interference often compromises catalytic activity and/or enantioselectivity.”

Question 2:

2. The same issue with “synchronization” applies on page 2, line 29: “However, the fundamental challenge of a conventional chemoenzymatic DKR process lies in synchronizing the two catalytic events of racemization.” Modify the sentence by removing “synchronizing.” I commented on this also in the previous round of revision.

Response: According to the reviewer’s suggestion, we have replaced “synchronizing” with “compatibility.” Please see the revised manuscript as follows.

Revise (page 2, line 29): “However, the fundamental challenge of a conventional chemoenzymatic DKR process lies in synchronizing the two catalytic events of racemization and resolution in accordance with the core principles (Figure 1A):[9-12].”

To: "However, the fundamental challenge of a conventional chemoenzymatic DKR process lies in achieving compatibility between the two catalytic events of racemization and resolution in accordance with the core principles (Figure 1A):[9-12]"

Question 3:

3. In the abstract: DKR is not a single-step process. It is a two-step process: racemization + acylation (in this case). If concurrent, we talk about a one-stage, two-step process. If not concurrent (as in this work, because there is physical separation via a membrane), we talk about a two-stage, two-step process. Revise the sentence in the abstract accordingly: "By spatially separating racemization and resolution while enabling their collaborative operation within a single streamlined step (….)".

Response: According to the reviewer's suggestion, we have adopted the description "a two-stage, two-step process" and revised the statement as follows.

Revise (page 1, abstract): "By spatially separating racemization and resolution while enabling their collaborative operation within a single streamlined step, this approach dramatically enhances the typically low efficiency of conventional DKR, allowing the efficient synthesis of tetra-substituted 3-hydroxyphthalide esters that are challenging to access by traditional methods, and greatly expanding the scope of chiral phthalide preparation."

To: "By spatially separating racemization and resolution while enabling their collaborative operation within a two-stage, two-step process, this approach dramatically enhances the typically low efficiency of conventional DKR, allowing the efficient synthesis of tetra-substituted 3-hydroxyphthalide esters that are challenging to access by traditional methods, and greatly expanding the scope of chiral phthalide preparation."

Question 4:

4. In the abstract, there are again "grand" statements, such as: "(….) advancing chemoenzymatic DKR methodology but also provides a general and scalable platform with broad implications for pharmaceutical synthesis." This is not a "general" platform, and the implication is not so "broad." I recommend making the statement in the abstract less bold.

Response: According to the reviewer's suggestion, we have revised the statement as follows.

Revise (page 1, abstract): "This membrane-modulated strategy not only streamlines the typically laborious optimization required in conventional DKR for advancing

chemoenzymatic DKR methodology but also provides a general and scalable platform with broad implications for pharmaceutical synthesis."

To: "This membrane-modulated strategy not only streamlines the typically laborious optimization required in conventional DKR for developing an alternative chemoenzymatic DKR approach but also provides a useful platform with the potential for pharmaceutical synthesis."

Question 5

5. Page 8, consider the sentence: "These findings demonstrate that the designed membrane-modulated DKR strategy could achieve the anticipated coordination of DBU-mediated racemization with CALB-catalyzed resolution in a two-stage manner, thereby enabling an efficient chemoenzymatic DKR to proceed that was previously incompatible under the conventional single-step chemoenzymatic conditions." "Single-step chemoenzymatic conditions" is incorrect. It is a two-step concurrent process. For terminology in cascade processes, see for example: Schrittwieser, J. H. et al. Artificial Biocatalytic Linear Cascades for Preparation of Organic Molecules. Chem. Rev. 118, 270–348 (2018).

Response: According to the reviewer's suggestion, we have revised the statement as follows.

Revise (Page 8): "These findings demonstrate that the designed membrane-modulated DKR strategy could achieve the anticipated coordination of DBU-mediated racemization with CALB-catalyzed resolution in a two-stage manner, thereby enabling an efficient chemoenzymatic DKR to proceed that was previously incompatible under the conventional single-step chemoenzymatic conditions."

To: "These findings demonstrate that the designed membrane-modulated DKR strategy could achieve the anticipated coordination of DBU-mediated racemization with CALB-catalyzed resolution in a two-stage, two-step process, thereby enabling an efficient chemoenzymatic DKR to proceed that was previously incompatible under the conventional one-stage two-step chemoenzymatic conditions."

Responses to the comment of Reviewer 1

Reviewer #1 (Remarks to the Author):

Comments: The author has made substantial and appropriate revisions, which significantly enhanced the quality of the manuscript. This reviewer supports its acceptance for publication in Nat. Commun. in current version.

Response: We sincerely thank you for your valuable comments provided previously, which have greatly helped improve the quality of our manuscript.

Responses to the comment of Reviewer 4

Reviewer #4 (Remarks to the Author):

Comments:

Authors have answered and addressed in deep all the questions and suggestions made by this refereee. They have included the study with a novel substrate (dihydrofuran), they have developed the PDMS stability and reusability study, and they have measured the diffusion rate of intermediate 2a through PDMS. All these modifications have been performed after a really extensive experimental work which is shown in the revised version. From my side, manuscript is suitable for publications with all these modifications.

Response: We sincerely thank you for your valuable comments provided previously, which have greatly helped improve the quality of our manuscript.
